# 25th Annual Computational Neuroscience Meeting: CNS-2016

**DOI:** 10.1186/s12868-016-0283-6

**Published:** 2016-08-18

**Authors:** Tatyana O. Sharpee, Alain Destexhe, Mitsuo Kawato, Vladislav Sekulić, Frances K. Skinner, Daniel K. Wójcik, Chaitanya Chintaluri, Dorottya Cserpán, Zoltán Somogyvári, Jae Kyoung Kim, Zachary P. Kilpatrick, Matthew R. Bennett, Kresimir Josić, Irene Elices, David Arroyo, Rafael Levi, Francisco B. Rodriguez, Pablo Varona, Eunjin Hwang, Bowon Kim, Hio-Been Han, Tae Kim, James T. McKenna, Ritchie E. Brown, Robert W. McCarley, Jee Hyun Choi, James Rankin, Pamela Osborn Popp, John Rinzel, Alejandro Tabas, André Rupp, Emili Balaguer-Ballester, Matias I. Maturana, David B. Grayden, Shaun L. Cloherty, Tatiana Kameneva, Michael R. Ibbotson, Hamish Meffin, Veronika Koren, Timm Lochmann, Valentin Dragoi, Klaus Obermayer, Maria Psarrou, Maria Schilstra, Neil Davey, Benjamin Torben-Nielsen, Volker Steuber, Huiwen Ju, Jiao Yu, Michael L. Hines, Liang Chen, Yuguo Yu, Jimin Kim, Will Leahy, Eli Shlizerman, Justas Birgiolas, Richard C. Gerkin, Sharon M. Crook, Atthaphon Viriyopase, Raoul-Martin Memmesheimer, Stan Gielen, Yuri Dabaghian, Justin DeVito, Luca Perotti, Anmo J. Kim, Lisa M. Fenk, Cheng Cheng, Gaby Maimon, Chang Zhao, Yves Widmer, Simon Sprecher, Walter Senn, Geir Halnes, Tuomo Mäki-Marttunen, Daniel Keller, Klas H. Pettersen, Ole A. Andreassen, Gaute T. Einevoll, Yasunori Yamada, Moira L. Steyn-Ross, D. Alistair Steyn-Ross, Jorge F. Mejias, John D. Murray, Henry Kennedy, Xiao-Jing Wang, Alexandra Kruscha, Jan Grewe, Jan Benda, Benjamin Lindner, Laurent Badel, Kazumi Ohta, Yoshiko Tsuchimoto, Hokto Kazama, B. Kahng, Nicoladie D. Tam, Luca Pollonini, George Zouridakis, Jaehyun Soh, DaeEun Kim, Minsu Yoo, S. E. Palmer, Viviana Culmone, Ingo Bojak, Andrea Ferrario, Robert Merrison-Hort, Roman Borisyuk, Chang Sub Kim, Taro Tezuka, Pangyu Joo, Young-Ah Rho, Shawn D. Burton, G. Bard Ermentrout, Jaeseung Jeong, Nathaniel N. Urban, Petr Marsalek, Hoon-Hee Kim, Seok-hyun Moon, Do-won Lee, Sung-beom Lee, Ji-yong Lee, Yaroslav I. Molkov, Khaldoun Hamade, Wondimu Teka, William H. Barnett, Taegyo Kim, Sergey Markin, Ilya A. Rybak, Csaba Forro, Harald Dermutz, László Demkó, János Vörös, Andrey Babichev, Haiping Huang, Sergio Verduzco-Flores, Filipa Dos Santos, Peter Andras, Christoph Metzner, Achim Schweikard, Bartosz Zurowski, James P. Roach, Leonard M. Sander, Michal R. Zochowski, Quinton M. Skilling, Nicolette Ognjanovski, Sara J. Aton, Michal Zochowski, Sheng-Jun Wang, Guang Ouyang, Jing Guang, Mingsha Zhang, K. Y. Michael Wong, Changsong Zhou, Peter A. Robinson, Paula Sanz-Leon, Peter M. Drysdale, Felix Fung, Romesh G. Abeysuriya, Chris J. Rennie, Xuelong Zhao, Yoonsuck Choe, Huei-Fang Yang, Yuanyuan Mi, Xiaohan Lin, Si Wu, Joscha Liedtke, Manuel Schottdorf, Fred Wolf, Yoriko Yamamura, Jeffery R. Wickens, Timothy Rumbell, Julia Ramsey, Amy Reyes, Danel Draguljić, Patrick R. Hof, Jennifer Luebke, Christina M. Weaver, Hu He, Xu Yang, Hailin Ma, Zhiheng Xu, Yuzhe Wang, Kwangyeol Baek, Laurel S. Morris, Prantik Kundu, Valerie Voon, Everton J. Agnes, Tim P. Vogels, William F. Podlaski, Martin Giese, Pradeep Kuravi, Rufin Vogels, Alexander Seeholzer, William Podlaski, Rajnish Ranjan, Tim Vogels, Joaquin J. Torres, Fabiano Baroni, Roberto Latorre, Bart Gips, Eric Lowet, Mark J. Roberts, Peter de Weerd, Ole Jensen, Jan van der Eerden, Abdorreza Goodarzinick, Mohammad D. Niry, Alireza Valizadeh, Aref Pariz, Shervin S. Parsi, Julia M. Warburton, Lucia Marucci, Francesco Tamagnini, Jon Brown, Krasimira Tsaneva-Atanasova, Florence I. Kleberg, Jochen Triesch, Bahar Moezzi, Nicolangelo Iannella, Natalie Schaworonkow, Lukas Plogmacher, Mitchell R. Goldsworthy, Brenton Hordacre, Mark D. McDonnell, Michael C. Ridding, Martin Zapotocky, Daniel Smit, Coralie Fouquet, Alain Trembleau, Sakyasingha Dasgupta, Isao Nishikawa, Kazuyuki Aihara, Taro Toyoizumi, Daniel T. Robb, Nick Mellen, Natalia Toporikova, Rongxiang Tang, Yi-Yuan Tang, Guangsheng Liang, Seth A. Kiser, James H. Howard, Julia Goncharenko, Sergej O. Voronenko, Tosif Ahamed, Greg Stephens, Pierre Yger, Baptiste Lefebvre, Giulia Lia Beatrice Spampinato, Elric Esposito, Marcel Stimberg et Olivier Marre, Hansol Choi, Min-Ho Song, SueYeon Chung, Dan D. Lee, Haim Sompolinsky, Ryan S. Phillips, Jeffrey Smith, Alexandra Pierri Chatzikalymniou, Katie Ferguson, N. Alex Cayco Gajic, Claudia Clopath, R. Angus Silver, Padraig Gleeson, Boris Marin, Sadra Sadeh, Adrian Quintana, Matteo Cantarelli, Salvador Dura-Bernal, William W. Lytton, Andrew Davison, Luozheng Li, Wenhao Zhang, Dahui Wang, Youngjo Song, Sol Park, Ilhwan Choi, Hee-sup Shin, Hannah Choi, Anitha Pasupathy, Eric Shea-Brown, Dongsung Huh, Terrence J. Sejnowski, Simon M. Vogt, Arvind Kumar, Robert Schmidt, Stephen Van Wert, Steven J. Schiff, Richard Veale, Matthias Scheutz, Sang Wan Lee, Júlia Gallinaro, Stefan Rotter, Leonid L. Rubchinsky, Chung Ching Cheung, Shivakeshavan Ratnadurai-Giridharan, Safura Rashid Shomali, Majid Nili Ahmadabadi, Hideaki Shimazaki, S. Nader Rasuli, Xiaochen Zhao, Malte J. Rasch, Jens Wilting, Viola Priesemann, Anna Levina, Lucas Rudelt, Joseph T. Lizier, Richard E. Spinney, Mikail Rubinov, Michael Wibral, Ji Hyun Bak, Jonathan Pillow, Yuan Zaho, Il Memming Park, Jiyoung Kang, Hae-Jeong Park, Jaeson Jang, Se-Bum Paik, Woochul Choi, Changju Lee, Min Song, Hyeonsu Lee, Youngjin Park, Ergin Yilmaz, Veli Baysal, Mahmut Ozer, Daniel Saska, Thomas Nowotny, Ho Ka Chan, Alan Diamond, Christoph S. Herrmann, Micah M. Murray, Silvio Ionta, Axel Hutt, Jérémie Lefebvre, Philipp Weidel, Renato Duarte, Abigail Morrison, Jung H. Lee, Ramakrishnan Iyer, Stefan Mihalas, Christof Koch, Mihai A. Petrovici, Luziwei Leng, Oliver Breitwieser, David Stöckel, Ilja Bytschok, Roman Martel, Johannes Bill, Johannes Schemmel, Karlheinz Meier, Timothy B. Esler, Anthony N. Burkitt, Robert R. Kerr, Bahman Tahayori, Max Nolte, Michael W. Reimann, Eilif Muller, Henry Markram, Antonio Parziale, Rosa Senatore, Angelo Marcelli, K. Skiker, M. Maouene, Samuel A. Neymotin, Alexandra Seidenstein, Peter Lakatos, Terence D. Sanger, Rosemary J. Menzies, Campbell McLauchlan, Sacha J. van Albada, David J. Kedziora, Samuel Neymotin, Cliff C. Kerr, Benjamin A. Suter, Gordon M. G. Shepherd, Juhyoung Ryu, Sang-Hun Lee, Joonwon Lee, Hyang Jung Lee, Daeseob Lim, Jisung Wang, Heonsoo Lee, Nam Jung, Le Anh Quang, Seung Eun Maeng, Tae Ho Lee, Jae Woo Lee, Chang-hyun Park, Sora Ahn, Jangsup Moon, Yun Seo Choi, Juhee Kim, Sang Beom Jun, Seungjun Lee, Hyang Woon Lee, Sumin Jo, Eunji Jun, Suin Yu, Felix Goetze, Pik-Yin Lai, Seonghyun Kim, Jeehyun Kwag, Hyun Jae Jang, Marko Filipović, Ramon Reig, Ad Aertsen, Gilad Silberberg, Claudia Bachmann, Simone Buttler, Heidi Jacobs, Kim Dillen, Gereon R. Fink, Juraj Kukolja, Daniel Kepple, Hamza Giaffar, Dima Rinberg, Steven Shea, Alex Koulakov, Jyotika Bahuguna, Tom Tetzlaff, Jeanette Hellgren Kotaleski, Tim Kunze, Andre Peterson, Thomas Knösche, Minjung Kim, Hojeong Kim, Ji Sung Park, Ji Won Yeon, Sung-Phil Kim, Jae-Hwan Kang, Chungho Lee, Andreas Spiegler, Spase Petkoski, Matias J. Palva, Viktor K. Jirsa, Maria L. Saggio, Silvan F. Siep, William C. Stacey, Christophe Bernar, Oh-hyeon Choung, Yong Jeong, Yong-il Lee, Su Hyun Kim, Mir Jeong, Jeungmin Lee, Jaehyung Kwon, Jerald D. Kralik, Jaehwan Jahng, Dong-Uk Hwang, Jae-Hyung Kwon, Sang-Min Park, Seongkyun Kim, Hyoungkyu Kim, Pyeong Soo Kim, Sangsup Yoon, Sewoong Lim, Choongseok Park, Thomas Miller, Katie Clements, Sungwoo Ahn, Eoon Hye Ji, Fadi A. Issa, JeongHun Baek, Shigeyuki Oba, Junichiro Yoshimoto, Kenji Doya, Shin Ishii, Thiago S. Mosqueiro, Martin F. Strube-Bloss, Brian Smith, Ramon Huerta, Michal Hadrava, Jaroslav Hlinka, Hannah Bos, Moritz Helias, Charles M. Welzig, Zachary J. Harper, Won Sup Kim, In-Seob Shin, Hyeon-Man Baek, Seung Kee Han, René Richter, Julien Vitay, Frederick Beuth, Fred H. Hamker, Kelly Toppin, Yixin Guo, Bruce P. Graham, Penelope J. Kale, Leonardo L. Gollo, Merav Stern, L. F. Abbott, Leonid A. Fedorov, Martin A. Giese, Mohammad Hovaidi Ardestani, Mohammad Javad Faraji, Kerstin Preuschoff, Wulfram Gerstner, Margriet J. van Gendt, Jeroen J. Briaire, Randy K. Kalkman, Johan H. M. Frijns, Won Hee Lee, Sophia Frangou, Ben D. Fulcher, Patricia H. P. Tran, Alex Fornito, Stephen V. Gliske, Eugene Lim, Katherine A. Holman, Christian G. Fink, Jinseop S. Kim, Shang Mu, Kevin L. Briggman, H. Sebastian Seung, the EyeWirers, Detlef Wegener, Lisa Bohnenkamp, Udo A. Ernst, Anna Devor, Anders M. Dale, Glenn T. Lines, Andy Edwards, Aslak Tveito, Espen Hagen, Johanna Senk, Markus Diesmann, Maximilian Schmidt, Rembrandt Bakker, Kelly Shen, Gleb Bezgin, Claus-Christian Hilgetag, Sacha Jennifer van Albada, Haoqi Sun, Olga Sourina, Guang-Bin Huang, Felix Klanner, Cornelia Denk, Katharina Glomb, Adrián Ponce-Alvarez, Matthieu Gilson, Petra Ritter, Gustavo Deco, Maria A. G. Witek, Eric F. Clarke, Mads Hansen, Mikkel Wallentin, Morten L. Kringelbach, Peter Vuust, Guido Klingbeil, Erik De Schutter, Weiliang Chen, Yunliang Zang, Sungho Hong, Akira Takashima, Criseida Zamora, Andrew R. Gallimore, Dennis Goldschmidt, Poramate Manoonpong, Philippa J. Karoly, Dean R. Freestone, Daniel Soundry, Levin Kuhlmann, Liam Paninski, Mark Cook, Jaejin Lee, Yonatan I. Fishman, Yale E. Cohen, James A. Roberts, Luca Cocchi, Yann Sweeney, Soohyun Lee, Woo-Sung Jung, Youngsoo Kim, Younginha Jung, Yoon-Kyu Song, Frédéric Chavane, Karthik Soman, Vignesh Muralidharan, V. Srinivasa Chakravarthy, Sabyasachi Shivkumar, Alekhya Mandali, B. Pragathi Priyadharsini, Hima Mehta, Catherine E. Davey, Braden A. W. Brinkman, Tyler Kekona, Fred Rieke, Michael Buice, Maurizio De Pittà, Hugues Berry, Nicolas Brunel, Michael Breakspear, Gary Marsat, Jordan Drew, Phillip D. Chapman, Kevin C. Daly, Samual P. Bradle, Sat Byul Seo, Jianzhong Su, Ege T. Kavalali, Justin Blackwell, LieJune Shiau, Laure Buhry, Kanishka Basnayake, Sue-Hyun Lee, Brandon A. Levy, Chris I. Baker, Timothée Leleu, Ryan T. Philips, Karishma Chhabria

**Affiliations:** 1 Computational Neurobiology Laboratory, The Salk Institute for Biological Studies, San Diego, CA USA; 2 UNIC, CNRS, Gif sur Yvette, France; 3 The European Institute for Theoretical Neuroscience (EITN), Paris, France; 4 ATR Computational Neuroscience Laboratories, 2-2 Hikaridai, Seika-cho, Soraku-gun, Kyoto, 619-0288 Japan; 5 Krembil Research Institute, University Health Network, Toronto, ON M5T 2S8 Canada; 6 Department of Physiology, University of Toronto, Toronto, ON M5S 1A8 Canada; 7 Department of Medicine (Neurology), University of Toronto, Toronto, ON M5T 2S8 Canada; 8 Department of Neurophysiology, Nencki Institute of Experimental Biology, Warsaw, Poland; 9 Department of Theory, Wigner Research Centre for Physics of the Hungarian Academy of Sciences, Budapest, 1121 Hungary; 10 Department of Mathematical Sciences, KAIST, Daejoen, 34141 Republic of Korea; 11 Department of Mathematics, University of Houston, Houston, TX 77004 USA; 12 Department of Biochemistry & Cell Biology and Institute of Biosciences and Bioengineering, Rice University, Houston, TX 77005 USA; 13 Department of Biology and Biochemistry, University of Houston, Houston, TX 77004 USA; 14 Grupo de Neurocomputación Biológica, Dpto. de Ingeniería Informática, Escuela Politécnica Superior, Universidad Autónoma de Madrid, Madrid, Spain; 15 Department of Biological Sciences, University of Southern California, Los Angeles, CA USA; 16 Center for Neuroscience, Korea Institute of Science and Technology, Hwarang-ro 14-gil 5, Seongbuk-gu, Seoul, 02792 South Korea; 17 Department of Neuroscience, University of Science and Technology, 217 Gajeong-ro, Yuseong-gu, Daejon, 34113 South Korea; 18 Department of Psychology, Yonsei University, 50 Yonsei-ro, Seodaemun-gu, Seoul, 03722 South Korea; 19 Department of Psychiatry, Kyung Hee University Hospital at Gangdong, 892, Dongnam-ro, Gangdong-gu, Seoul, 05278 South Korea; 20 Department of Psychiatry, Veterans Administration Boston Healthcare System and Harvard Medical School, Brockton, MA 02301 USA; 21 Center for Neural Science, New York University, New York, NY 10003 USA; 22 Courant Institute of Mathematical Sciences, New York University, New York, NY 10012 USA; 23 Faculty of Science and Technology, Bournemouth University, Bournemouth, England, UK; 24 Heidelberg University, Baden-Württemberg, Germany; 25 Heidelberg University, Baden-Württemberg, Germany; 26 Bernstein Center for Computational Neuroscience, Heidelberg-Mannheim, Baden-Württemberg, Germany; 27 National Vision Research Institute, Australian College of Optometry, Carlton, 3053 Australia; 28 NeuroEngineering Laboratory, Dept. Electrical & Electronic Engineering, University of Melbourne, Parkville, VIC 3010 Australia; 29 Centre for Neural Engineering, University of Melbourne, Parkville, 3010 Australia; 30 Department of Physiology, Monash University, Melbourne, 3800 Australia; 31 ARC Centre of Excellence for Integrative Brain Function, Dept. Optometry and Vision Sciences, University of Melbourne, Parkville, 3010 Australia; 32 Institute of Software Engineering and Theoretical Computer Science, Technische Universitaet Berlin, 10587 Berlin, Germany; 33 Bernstein Center for Computational Neuroscience Berlin, Humboldt-Universitaet zu Berlin, 10115 Berlin, Germany; 34 Department of Neurobiology and Anatomy, University of Texas-Houston Medical School, Houston, TX 77030 USA; 35 Centre for Computer Science and Informatics Research, University of Hertfordshire, Hatfield, AL10 9AB UK; 36 School of Life Science and the Collaborative Innovation Center for Brain Science, Fudan University, Shanghai, 200438 China; 37 LinyiHospitalof TraditionalChineseMedicine, 211 Jiefang Road, Lanshan, Linyi, 276000 Shandong Province China; 38 Department of Neuroscience, Yale University School of Medicine, New Haven, CT 06520 USA; 39 Department of Neurosurgery, Huashan Hospital, Shanghai Medical College, Fudan University, Shanghai, China; 40 Department of Applied Mathematics, University of Washington, Seattle, WA 98195 USA; 41 Amazon.com lnc, Seattle, WA 98108 USA; 42 Department of Electrical Engineering, University of Washington, Seattle, WA 98195 USA; 43 School of Life Science, Arizona State University, Tempe, AZ 85287 USA; 44 School of Mathematical and Statistical Sciences, Arizona State University, Tempe, AZ 85287 USA; 45 Donders Institute for Brain, Cognition and Behaviour, Radboud University Nijmegen (Medical Centre), Nijmegen, The Netherlands; 46 Department for Biophysics, Faculty of Science, Radboud University Nijmegen, Nijmegen, The Netherlands; 47 Department for Neuroinformatics, Faculty of Science, Radboud University Nijmegen, Nijmegen, The Netherlands; 48 Center for Theoretical Neuroscience, Columbia University, New York, NY USA; 49 Department of Neurology Pediatrics, Baylor College of Medicine, Houston, TX 77030 USA; 50 Department of Computational and Applied Mathematics, Rice University, Houston, TX 77005 USA; 51 Physics Department, Texas Southern University, 3100 Cleburne St, Houston, TX 77004 USA; 52 Laboratory of Integrative Brain Function, The Rockefeller University, New York, NY 10065 USA; 53 Department of Physiology, University of Bern, 3012 Bern, Switzerland; 54 Department of Biology, University of Fribourg, 1700 Fribourg, Switzerland; 55 Department of Mathematical Sciences and Technology, Norwegian University of Life Sciences, Ås, Norway; 56 NORMENT, Institute of Clinical Medicine, University of Oslo, Oslo, Norway; 57 The Blue Brain Project, École Polytechnique Fédérale de Lausanne (EPFL), Lausanne, Switzerland; 58 Letten Centre and Glialab, Dept. of Molecular Medicine, Inst. of Basic Medical Sciences, University of Oslo, Oslo, Norway; 59 Centre for Molecular Medicine Norway, University of Oslo, Oslo, Norway; 60 Department of Physics, University of Oslo, Oslo, Norway; 61 IBM Research - Tokyo, Tokyo, Japan; 62 School of Engineering, University of Waikato, Hamilton, 3240 New Zealand; 63 Department of Psychiatry, Yale School of Medicine, New Haven, CT 06511 USA; 64 INSERM U846, Stem Cell and Brain Research Institute, Bron Cedex, France; 65 NYU-ECNU Institute of Brain and Cognitive Science, NYU Shanghai, Shanghai, China; 66 Bernstein Center for Computational Neuroscience, 10115 Berlin, Germany; 67 Institute for Physics, Humboldt-Universität zu Berlin, 12489 Berlin, Germany; 68 Institute for Neurobiology, Eberhardt Karls Universität Tübingen, Tübingen, Germany; 69 Bernstein Center for Computational Neuroscience, Munich, Germany; 70 Riken Brai Science Institute, 2-1 Hirosawa, Wako, Saitama Japan; 71 Department of Physics and Astronomy, Seoul National University, Seoul, 08826 Korea; 72 Department of Biological Sciences, University of North Texas, Denton, TX 76203 USA; 73 College of Technology, the University of Houston, Houston, TX 77204 USA; 74 Departments of Engineering Technology, Computer Science, and Electrical and Computer Engineering, University of Houston, Houston, TX 77204 USA; 75 Biological Cybernetics, School of Electrical and Electronic Engineering, Yonsei University, Shinchon, Seoul, 120-749 South Korea; 76 Committee on Computational Neuroscience, University of Chicago, Chicago, IL USA; 77 Department of Organismal Biology and Anatomy, University of Chicago, Chicago, IL USA; 78 School of Psychology, University of Reading, Reading, Berkshire RG1 6AY UK; 79 School of Computing and Mathematics, Plymouth University, Plymouth, PL4 8AA UK; 80 Department of Physics, Chonnam National University, Gwangju, 61186 Republic of Korea; 81 Faculty of Library, Information and Media Science, University of Tsukuba, Tsukuba, 305-0821 Japan; 82 Physics, POSTECH, Pohang, 37673 Republic of Korea; 83 Department of Mathematics, University of Pittsburgh, Pittsburgh, PA 15260 USA; 84 Department of Bio and Brain Engineering/Program of Brain and Cognitive Engineering, Korea Advanced Institute of Science and Technology (KAIST), Daejeon, 34141 South Korea; 85 Department of Biological Sciences, Carnegie Mellon University, Pittsburgh, PA 15213 USA; 86 Center for the Neural Basis of Cognition, Pittsburgh, PA 15213 USA; 87 Inst. of Pathological Physiology, First Faculty of Medicine, Charles University in Prague, 128 53 Prague, Czech Republic; 88 Czech Technical University in Prague, Zikova 1903/4, 166 36 Prague, Czech Republic; 89 Korea Science Academy of KAIST, Busan, 10547 South Korea; 90 Department of Mathematics and Statistics, Georgia State University, Atlanta, GA 30303 USA; 91 Department of Neurobiology and Anatomy, Drexel University, Philadelphia, PA 19129 USA; 92 Department of Mathematical Sciences, Indiana University – Purdue University, Indianapolis, IN 46202 USA; 93 LBB, ETH Zürich, 8051 Zurich, Switzerland; 94 RIKEN Brain Science Institute, Wako-shi, Saitama, Japan; 95 Computational Neuroscience Unit, Okinawa Institute of Science and Technology, Okinawa, 1919-1 Japan; 96 School of Computing and Mathematics, Keele University, Newcastle-under-Lyme, ST5 5BG UK; 97 Science and Technology Research Institute, University of Hertfordshire, Hatfield, UK; 98 Institute for Robotics and Cognitive Systems, University of Luebeck, Luebeck, Germany; 99 Department of Psychiatry, University of Luebeck, Schleswig-Holstein, Luebeck, Germany; 100 Neuroscience Graduate Program, University of Michigan, Ann Arbor, MI 48109 USA; 101 Center for the Study of Complex Systems, University of Michigan, Ann Arbor, MI 48109 USA; 102 Department of Physics, University of Michigan, Ann Arbor, MI 48109 USA; 103 Biophysics Program, University of Michigan, Ann Arbor, MI 48109 USA; 104 Department of Molecular, Cellular, and Developmental Biology, University of Michigan, Ann Arbor, MI 48109 USA; 105 Department of Physics, University of Michigan, Ann Arbor, MI 48109 USA; 106 Department of Physics, Shaanxi Normal University, Xi’An City, ShaanXi Province China; 107 Department of Physics and Centre for Nonlinear Studies, Institute of Computational and Theoretical Studies, Hong Kong Baptist University, Kowloon Tong, Hong Kong; 108 State Key Laboratory of Cognitive Neuroscience and Learning, Beijing Normal University, Beijing, China; 109 Department of Physics, Hong Kong University of Science and Technology, Clear Water Bay, Hong Kong; 110 Beijing Computational Science Research Center, Beijing, 100084 People’s Republic of China; 111 Research Centre, HKBU Institute of Research and Continuing Education, Shenzhen, China; 112 School of Physics, University of Sydney, Sydney, NSW 2006 Australia; 113 Center for Integrative Brain Function, University of Sydney, Sydney, NSW 2006 Australia; 114 Department of Computer Science & Engineering, Texas A&M University, College Station, TX 77845 USA; 115 Research Center for Information Technology Innovation, Academia Sinica, Taipei, Taiwan; 116 State Key Lab of Cognitive Neuroscience & Learning, IDG/McGovern Institute for Brain Research, Beijing Normal University, Beijing, 100875 China; 117 Max Planck Institute for Dynamics and Self-Organization, Göttingen, Germany; 118 Bernstein Center for Computational Neuroscience, Göttingen, Germany; 119 Neurobiology Research Unit, Okinawa Institute of Science and Technology, Onna-son, Okinawa, 904-0412 Japan; 120 Computational Biology Center, IBM Research, Thomas J. Watson Research Center, Yorktown Heights, NY 10598 USA; 121 Department of Mathematics, Franklin and Marshall College, Lancaster, PA 17604 USA; 122 Fishberg Department of Neuroscience and Friedman Brain Institute, Icahn School of Medicine at Mount Sinai, New York, NY 10029 USA; 123 Department of Anatomy and Neurobiology, Boston University School of Medicine, Boston, MA 02118 USA; 124 Institute of Microelectronics, Tsinghua University, Beijing, 100081 China; 125 School of Software, Beijing Institute of Technology, Beijing, 100083 China; 126 Department of Psychiatry, University of Cambridge, Cambridge, CB2 0QQ UK; 127 Department of Biomedical Engineering, Ulsan National Institute of Science and Technology, Ulsan, South Korea; 128 Departments of Radiology and Psychiatry, Icahn School of Medicine at Mount Sinai, New York City, NY 10029 USA; 129 Centre for Neural Circuits and Behaviour, University of Oxford, Oxford, OX1 3SR UK; 130 Centre for Neural Circuits and Behaviour, University of Oxford, Oxford, UK; 131 Section Computational Sensomotorics, CIN & HIH, Department of Cognitive Neurology, University Clinic Tübingen, Tübingen, Germany; 132 Lab. Neuro en Psychofysiologie, Dept. Neuroscience, KU Leuven, Louvain, Belgium; 133 Laboratory of Computational Neuroscience, EPF Lausanne, Lausanne, Switzerland; 134 Centre for Neural Circuits and Behaviour, University of Oxford, Oxford, UK; 135 The Blue Brain Project, EPF Lausanne, Lausanne, Switzerland; 136 Departamento de Electromagnetismo y Física de la Materia, and Institute “Carlos I” for Theoretical and Computational Physics, University of Granada, Granada, Spain; 137 School of Psychological Sciences, Faculty of Biomedical and Psychological Sciences, Monash University, Parkville, Australia; 138 Faculty of Psychology and Neuroscience, Maastricht University, 6200 MD Maastricht, The Netherlands; 139 Department of Physics, Institute for Advanced Studies in Basic Sciences (IASBS), Zanjan, 45137-66731 Iran; 140 Center for Research in Climate Change and Global Warming (CRCC), Institute for Advanced Studies in Basic Sciences (IASBS), Zanjan, 45137-66731 Iran; 141 School of Cognitive Sciences, Institute for Research in Fundamental Sciences (IPM), Tehran, Iran; 142 Department of Physics, Institute for Advanced Studies in Basic Sciences, Zanjan, Iran; 143 School of Cognitive Sciences, Institute for Studies in Theoretical Physics and Mathematics, Niavaran, Tehran, Iran; 144 Bristol Centre for Complexity Sciences, University of Bristol, Bristol, BS8 1TR UK; 145 Department of Engineering Mathematics, University of Bristol, Bristol, BS8 1UB UK; 146 School of Physiology and Pharmacology, University of Bristol, Bristol, BS8 1TD UK; 147 Medical School, University of Exeter, Exeter, EX4 4PE UK; 148 School of Physiology and Pharmacology, University of Bristol, Bristol, BS8 1TD UK; 149 Medical School, University of Exeter, Exeter, EX4 4PE UK; 150 Department of Mathematics, University of Exeter, Exeter, EX4 4QF UK; 151 Frankfurt Institute for Advanced Studies, 60438 Frankfurt am Main, Hessen, Germany; 152 Computational and Theoretical Neuroscience Laboratory, School of Information Technology and Mathematical Sciences, University of South Australia, Adelaide, Australia; 153 School of Mathematical Sciences, University of Nottingham, Nottingham, UK; 154 Frankfurt Institute for Advanced Studies, Goethe-Universität, Frankfurt am Main, Germany; 155 Robinson Research Institute, School of Medicine, University of Adelaide, Adelaide, Australia; 156 Institute of Physiology of the Czech Academy of Sciences, Prague, Czech Republic; 157 Institute of Biophysics and Informatics, First Faculty of Medicine, Charles University in Prague, Prague, Czech Republic; 158 IBPS, Neuroscience Paris Seine, CNRS UMR8246, Inserm U1130, UPMC UM 119, Université Pierre et Marie Curie, Paris, France; 159 IBM Research - Tokyo, Tokyo, Japan; 160 RIKEN Brain Science Institute, Tokyo, Japan; 161 The University of Tokyo, Tokyo, Japan; 162 Department of Mathematics, Computer Science and Physics, Roanoke College, Salem, VA 24153 USA; 163 Department of Pediatrics, University of Louisville, Louisville, KY 40208 USA; 164 Department of Biology, Washington and Lee University, Lexington, VA 24450 USA; 165 Department of Psychology, Washington University in St. Louis, St. Louis, MO 63130 USA; 166 Department of Psychological Sciences, Texas Tech University, Lubbock, TX 79409 USA; 167 The Department of Veteran Affairs, District of Columbia VA Medical Center, Washington, DC 20420 USA; 168 Department of Psychology, The Catholic University of America, Washington, DC 20064 USA; 169 Department of Physics, Humboldt University, 10099 Berlin, Germany; 170 Biological Physics Theory Unit, Okinawa Institute of Science and Technology, Okinawa, 904-0495 Japan; 171 Department of Physics and Astronomy, Vrije Universiteit Amsterdam, Amsterdam, The Netherlands; 172 Institut de la Vision, INSERM UMRS 968, CNRS UMR 7210, Paris, France; 173 Bernstein Center Freiburg, Institute of Biology III, University of Freiburg, 79100 Freiburg, Germany; 174 fourMs group, Dept. Musicology, University of Oslo, 0371 Oslo, Norway; 175 Center for Brain Science, Harvard University, Cambridge, MA 02138 USA; 176 Department of Electrical and Systems Engineering, University of Pennsylvania, Philadelphia, PA 19104 USA; 177 Edmond and Lily Safra Center for Brain Sciences, Hebrew University, 91904 Jerusalem, Israel; 178 NINDS, NIH, Bethesda, MD 20892 USA; 179 Department of Physics, University of New Hampshire, Durham, NH 03824 USA; 180 Krembil Research Institute, University Health Network, Toronto, ON Canada; 181 Department of Physiology, University of Toronto, Toronto, ON Canada; 182 Department of Neuroscience, Yale School of Medicine, New Haven, CT 06520 USA; 183 Department of Medicine (Neurology), University of Toronto, Toronto, ON Canada; 184 Department of Neuroscience, Physiology and Pharmacology, University College London, London, UK; 185 Department of Bioengineering, Imperial College London, London, UK; 186 Metacell LLC, San Diego, CA USA; 187 State University of New York Downstate Medical Center, Brooklyn, NY USA; 188 Neuroinformatics group Unité de Neurosciences, Information et Complexité, CNRS, Gif sur Yvette, France; 189 State Key Laboratory of Cognitive Neuroscience & Learning, IDG/McGovern Institute for Brain Research, Beijing Normal University, Beijing, 100875 China; 190 School of System Science, Beijing Normal University, Beijing, 100875 China; 191 Bio and Brain Engineering, KAIST, Daejeon, 34141 Republic of Korea; 192 Center for Cognition and Sociality, IBS, Daejeon, 34047 Republic of Korea; 193 Department of Biological Structure, University of Washington, Seattle, WA 98195 USA; 194 UW Institute for Neuroengineering, University of Washington, Seattle, WA 98195 USA; 195 The Salk Institute for Biological Studies, La Jolla, CA 92037 USA; 196 Division of Biological Sciences, University of California at San Diego, La Jolla, CA 92095 USA; 197 BrainLinks-BrainTools, Cluster of Excellence, University of Freiburg, Freiburg, Germany; 198 Faculty of Biology and Bernstein Center Freiburg, University of Freiburg, Freiburg, Germany; 199 Department of Computational Biology, Royal Institute of Technology Stockholm, Stockholm, Sweden; 200 Center for Neural Engineering, Department of Engineering Science and Mechanics, The Pennsylvania State University, University Park, PA 16802 USA; 201 Departments of Neurosurgery and Physics, The Pennsylvania State University, University Park, PA 16802 USA; 202 National Institute for Physiological Sciences, Okazaki, Aichi Japan; 203 Department of Computer Science, Tufts University, Medford, MA USA; 204 Program of Brain and Cognitive Engineering, Korea Advanced Institute of Science and Technology (KAIST), Daejeon, South Korea; 205 KAIST Institute for Health Science and Technology, Korea Advanced Institute of Science and Technology (KAIST), Daejeon, South Korea; 206 Bernstein Center Freiburg & Faculty of Biology, University of Freiburg, Baden-Württember, Freiburg, 79194 Germany; 207 School of Physics, University of Sydney, Sydney, NSW Australia; 208 Department of Mathematical Sciences, Indiana University-Purdue University Indianapolis, Indianapolis, IN USA; 209 Stark Neurosciences Research Institute, Indiana University School of Medicine, Indianapolis, IN USA; 210 School of Cognitive Sciences, Institute for Research in Fundamental Sciences (IPM), Tehran, 19395-5746 Iran; 211 School of ECE, College of Engineering, University of Tehran, Tehran, 14155-6619 Iran; 212 RIKEN Brain Science Institute, Wako, Saitama 351-0198 Japan; 213 Department of Physics, University of Guilan, Rasht, 41335-1914 Iran; 214 School of Physics, Institute for Research in Fundamental Sciences (IPM), Tehran, 19395-5531 Iran; 215 Max-Planck-Institute for Dynamics and Self-Organization, 37077 Göttingen, Germany; 216 Bernstein Center for Computational Neuroscience, University of Göttingen, 37075 Göttingen, Germany; 217 IST Austria, 3400 Klosterneuburg, Austria; 218 BCCN & MPI for Dynamics and Self-Organization, 37077 Göttingen, Germany; 219 Dept. of Non-linear Dynamics, Max Planck Institute for Dynamics and Self-Organization, Göttingen, Germany; 220 School of Civil Engineering, The University of Sydney, Sydney, NSW Australia; 221 Complex Systems Research Group, Faculty of Engineering & IT, The University of Sydney, Sydney, NSW 2006 Australia; 222 Janelia Research Campus, Howard Hughes Medical Institute, Ashburn, VA 20147 USA; 223 Department of Psychiatry, University of Cambridge, Cambridge, UK; 224 MEG Unit, Brain Imaging Center, Goethe University, 60528 Frankfurt am Main, Germany; 225 Department of Nonlinear Dynamics, Max Planck Institute for Dynamics & Self-Organization, Göttingen, Germany; 226 Department of Physics & Lewis-Sigler Institute for Integrative Genomics, Princeton University, Princeton, NJ 08544 USA; 227 Department of Psychology & Princeton Neuroscience Institute, Princeton University, Princeton, NJ 08544 USA; 228 Department of Neurobiology and Behavior, Stony Brook University, Stony Brook, NY 11794 USA; 229 Department of Applied Mathematics and Statistics, Stony Brook University, Stony Brook, NY 11794 USA; 230 Institute for Advanced Computational Science, Stony Brook University, Stony Brook, NY 11794 USA; 231 Graduate School of Life Science, University of Hyogo, 3-2-1 Koto, Kamigori, Ako, Hyogo 678-1297 Japan; 232 Department of Nuclear Medicine, Radiology and Psychiatry, Yonsei University College of Medicine, Department of Cognitive Science, Yonsei University, 50 Yonsei-ro, Sinchon-dong Seodaemoon-gu, Seoul, 120-752 Republic of Korea; 233 Program of Brain and Cognitive Engineering, Korea Advanced Institute of Science and Technology, Daejeon, 34141 Republic of Korea; 234 Program of Brain and Cognitive Engineering, KAIST, Daejeon, 34141 Republic of Korea; 235 Department of Biomedical Engineering, Bülent Ecevit University, 67100 Zonguldak, Turkey; 236 Department of Electrical and Electronics Engineering, Bülent Ecevit University, 67100 Zonguldak, Turkey; 237 School of Engineering and Informatics, Sussex Neuroscience, University of Sussex, Falmer, Brighton, BN1 9QJ UK; 238 School of Engineering and Informatics, University of Sussex, Falmer, Brighton BN1 9QJ UK; 239 Research Center Neurosensory Science, Carl-von-Ossietzky University Oldenburg, Oldenburg, Germany; 240 The Laboratory for Investigative Neurophysiology (The LINE), Department of Clinical Neurosciences and Department of Radiology, University Hospital Center and University of Lausanne, 1011 Lausanne, Switzerland; 241 Deutscher Wetterdienst, 63067 Offenbach, Germany; 242 Krembil Research Institute, University Health Network, Toronto, ON M5T 2S8 Canada; 243 Institute of Advanced Simulation (IAS-6) & Institute of Neuroscience and Medicine (INM-6) & JARA BRAIN Institute I, Jülich Research Center, 52425 Jülich, Germany; 244 Bernstein Center Freiburg, Albert-Ludwig University of Freiburg, Freiburg im Breisgau 79104, Germany; 245 Institute for Adaptive and Neural Computation, School of Informatics, University of Edinburgh, Edinburgh, EH8 9AB UK; 246 Institute of Cognitive Neuroscience, Faculty of Psychology, Ruhr-University Bochum, 44801 Bochum, Germany; 247 Simulation Laboratory Neuroscience – Bernstein Facility for Simulation and Database Technology, Institute for Advanced Simulation, Jülich Aachen Research Alliance, Jülich Research Center, Jülich, Germany; 248 Allen Institute for Brain Science, Seattle, WA 98109 USA; 249 Kirchhoff-Institute for Physics, University of Heidelberg, Heidelberg, Germany; 250 NeuroEngineering Laboratory, Electrical & Electronic Engineering, The University of Melbourne, Parkville, VIC 3010 Australia; 251 IBM Research, Melbourne, Australia; 252 Monash Institute of Medical Engineering, Monash University, Melbourne, Australia; 253 National Vision Research Institute, Melbourne, Australia; 254 School of Mathematical Sciences, University of Nottingham, Nottingham, UK; 255 Blue Brain Project, École Polytechnique fédérale de Lausanne (EPFL), Geneva, Switzerland; 256 Department of Information and Electrical Engineering, University of Salerno, 84084 Fisciano, SA Italy; 257 Department of Information and Electrical Engineering and Applied Mathematics, University of Salerno, Fisciano, SA 81100 Italy; 258 Laboratory of Neural Computation, Istituto Italiano di Tecnologia, Rovereto, TN 38068 Italy; 259 LIST Laboratory, FST, Abdelmalek Essaadi’s University, Tangier, Morocco; 260 Department of Computer Science, ENSAT, Abdelmalek Essaadi’s University, Tangier, Morocco; 261 Department Physiology & Pharmacology, SUNY Downstate, Brooklyn, NY 11203 USA; 262 Department Neuroscience, Yale University School of Medicine, New Haven, CT USA; 263 Department of Chemical & Biomedical Engineering, Tandon School of Engineering, NYU, Brooklyn, NY USA; 264 Nathan Kline Institute for Psychiatric Research, Orangeburg, NY USA; 265 Department Biomedical Engineering, University of Southern California, Los Angeles, CA USA; 266 Div Neurology, Child Neurology and Movement Disorders, Children’s Hospital Los Angeles, Los Angeles, CA USA; 267 Department Neurology, Kings County Hospital Center, Brooklyn, NY 11203 USA; 268 Department of Physiology & Pharmacology, SUNY Downstate Medical Center, Brooklyn, NY 11023 USA; 269 Complex Systems Group, School of Physics, University of Sydney, Sydney, NSW 2006 Australia; 270 Institute of Neuroscience and Medicine (INM-6), Jülich Research Centre and JARA, Jülich, Germany; 271 Department Physiology, Northwestern University, Chicago, IL 60611 USA; 272 Department of Neuroscience, Physiology & Pharmacology, University College London, London, WC1E6BT UK; 273 Brain and Cognitive Science, Seoul National University, Seoul, 151-742 Republic of Korea; 274 Department of Brain and Cognitive Sciences, Seoul National University, Seoul, 151-742 Korea; 275 Department of Brain and Cognitive Neuroscience, Seoul National University, Gwanak-gu, South Korea; 276 Department of Brain and Cognitive Sciences, Seoul National University, Seoul, 08826 South Korea; 277 Physics Department, Pohang University of Science and Technology, Pohang, South Korea; 278 Department of Physics, Inha University, Namgu, Incheon, 22212 Korea; 279 Departments of Neurology, Ewha Womans University School of Medicine, Seoul, Korea; 280 Department of Medical Science, Ewha Womans University School of Medicine, Seoul, Korea; 281 Department of Electronics Engineering, Ewha Womans University College of Engineering, Seoul, Korea; 282 Brain & Cognitive Sciences, Ewha Womans University College of Scranton, Seoul, Korea; 283 Department of Electronics Engineering, Ewha Womans University, Seoul, 120-750 Korea; 284 Department of Neurology, Ewha Womans University, Seoul, 120-750 Korea; 285 Department of Physics, National Central University, Chung-Li, Taiwan, ROC; 286 Taiwan International Graduate Program for Molecular Science and Technology, Institute for Atomic and Molecular Sciences, Academia Sinica, Taipei, Taiwan, ROC; 287 Department of Brain and Cognitive Engineering, Korea University, Seoul, Korea; 288 Bernstein Center Freiburg, Freiburg, Germany; 289 Faculty of Biology, University of Freiburg, 79104 Freiburg, Germany; 290 Instituto de Neurociencias de Alicante, University of Alicante, Alicante, Spain; 291 Department of Neuroscience, Karolinska Institute, Stockholm, 17177 Sweden; 292 Dept. of Computational Science and Technology, School of Computer Science and Communication, KTH Royal Institute of Technology, 10040 Stockholm, Sweden; 293 Institute of Neuroscience and Medicine (INM-6) and Institute for Advanced Simulation (IAS-6) and JARA BRAIN Institute I, Jülich Research Centre, 52425 Jülich, Germany; 294 Faculty of Health, Medicine and Life Science, School for Mental Health and Neuroscience (MHeNS),Alzheimer Centre Limburg, Maastricht University Medical Centre, PO Box 616, 6200 MD Maastricht, The Netherlands; 295 Department of Radiology &Athinoula A. Martinos Center for Biomedical Imaging, Massachusetts General Hospital, Harvard Medical School, Boston, MA 02114 USA; 296 Department of Cognitive Neuroscience, Faculty of Psychology and Neuroscience, Maastricht University, PO BOX 616, 6200 MD Maastricht, The Netherlands; 297 Cognitive Neuroscience, Inst. of Neuroscience and Medicine (INM-3), Jülich Research Centre, Jülich, Germany; 298 Department of Neurology, University Hospital of Cologne, Cologne, Germany; 299 Computational Neuroscience, Bernstein Center Freiburg, 79104 Freiburg, Germany; 300 Cold Spring Harbor Laboratory, Cold Spring Harbor, NY 11724 USA; 301 NYU Neuroscience Institute, New York, NY 10016 USA; 302 Institute of Neuroscience and Medicine (INM-6), Institute for Advanced Simulation (IAS-6) and JARA BRAIN Institute I, Jülich Research Centre, Jülich, Germany; 303 Computational Brain Science, Dept. of Computational Science and Technology, School of Computer Science and Communication, KTH, Royal Institute of Technology, Stockholm, Sweden; 304 Max Planck Institute for Human Cognitive and Brain Sciences, Leipzig, Germany; 305 Institute of Biomedical Engineering and Informatics, Ilmenau University of Technology, Ilmenau, Germany; 306 Department of Medicine, University of Melbourne, Melbourne, Australia; 307 Division of IoT and Robotics Convergence Research, DGIST, Daegu, 42988 Korea; 308 Department of Human Factors Engineering, Ulsan National Institute of Science and Technology, Ulsan, 689-798 South Korea; 309 Department of Human and Systems Engineering, Ulsan National Institute of Science and Technology, Ulsan, South Korea; 310 INSERM UMR 1106 Institut de Neurosciences des Systèmes - Aix-Marseille Université, Marseille, France; 311 Aix-Marseille Université, CNRS, ISM UMR 7287, 13288 Marseille, France; 312 Neuroscience Center, University of Helsinki, 00014 Helsinki, Finland; 313 Dept of Neurology, Dept of Biomedical Engineering, University of Michigan, Ann Arbor, MI 48109 USA; 314 Program of Brain and Cognitive Engineering, College of Engineering, Korea Advanced Institute of Science and Technology (KAIST), Daejeon, 34141 South Korea; 315 Program of Brain Engineering, College of Engineering, Korea Advanced Institute of Science and Technology (KAIST), Daejeon, 34141 Republic of Korea; 316 Division of Computational Mathematics, National Institute for Mathematical Sciences (NIMS), Daejeon, 34047 South Korea; 317 Program of Brain and Cognitive Engineering, Korea Advanced Institute of Science and Technology (KAIST), Daejeon, 34141 South Korea; 318 Department of Mathematics, North Carolina A&T State University, Greensboro, NC 27411 USA; 319 Department of Biology, East Carolina University, Greenville, NC 27858 USA; 320 Department of Mathematics, East Carolina University, Greenville, NC 27858 USA; 321 David Geffen School of Medicine, UCLA, Los Angeles, CA 90095 USA; 322 Graduate School of Informatics, Kyoto University, Yoshidahonmachi 36-1, Sakyo, Kyoto, Japan; 323 Neural Computation Unit, Okinawa Institute of Science and Technology Graduate University, 1919-1 Tancha, Onna-son, Kunigami-gun, Okinawa Japan; 324 Graduate School of Information Science, Nara Institute of Science and Technology, 8916-5 Takayama, Ikoma, Nara Japan; 325 University of California San Diego, La Jolla, CA USA; 326 Biocenter University of Würzburg, Würzburg, Germany; 327 School of Life Sciences, Arizona State University, Tempe, AZ USA; 328 Department of Cybernetics, Faculty of Electrical Engineering, Czech Technical University in Prague, 166 27 Prague, Czech Republic; 329 Department of Nonlinear Dynamics and Complex Systems, Institute of Computer Science, The Czech Academy of Sciences, 182 07 Prague, Czech Republic; 330 National Institute of Mental Health, 250 67 Klecany, Czech Republic; 331 Department of Physics, Faculty 1, RWTH Aachen University, 52074 Aachen, Germany; 332 Departments of Neurology and Physiology, Medical College of Wisconsin, Milwaukee, WI 53226 USA; 333 College of Engineering & Applied Science, University of Wisconsin-Milwaukee, Milwaukee, WI 53211 USA; 334 Department of Physics, Chungbuk National University, Cheongju, Chungbuk 28644 Republic of Korea; 335 Korea Basic Science Institute, Cheongju, Chungbuk 28119 Republic of Korea; 336 Department of Computer Science, Chemnitz University of Technology, Chemnitz, Germany; 337 Bernstein Center for Computational Neuroscience, Charité University Medicine, Berlin, Germany; 338 Department of Mathematics, Drexel University, Philadelphia, PA 19104 USA; 339 National Vision Research Institute, Australian College of Optometry, Carlton, VIC 3053 Australia; 340 Centre for Neural Engineering, University of Melbourne, Parkville, VIC 3010 Australia; 341 Computational and Theoretical Neuroscience Laboratory, School of Information Technology and Mathematical Sciences, University of South Australia, Mawson Lakes, SA 5095 Australia; 342 Computing Science & Mathematics, School of Natural Sciences, University of Stirling, Stirling, FK9 4LA UK; 343 Systems Neuroscience Group, QIMR Berghofer Medical Research Institute, Brisbane, QLD 4006 Australia; 344 Faculty of Medicine, Technion, Haifa, Israel; 345 Department of Neuroscience and Department of Physiology and Cellular Biophysics, Columbia University, New York, NY USA; 346 Section for Computational Sensomotorics, Dept. Cognitive Neurology, CIN&HIH, Tübingen, Germany; 347 GTC, International Max Planck Research School, University of Tübingen, Tübingen, Germany; 348 Section Computational Sensomotorics, Department of Cognitive Neurology, CIN & HIH, 72076 Tübingen, Germany; 349 IMPRS for Cognitive and Systems Neuroscience, University Clinic Tübingen, 72076 Tübingen, Germany; 350 School of Life Sciences, Brain Mind Institute and School of Computer and Communication Sciences, Ecole Polytechnique Federal de Lausanne (EPFL), 1015 Lausanne, Switzerland; 351 Geneva Finance Research Institute (GFRI) and Swiss Center for Affective Sciences (CISA), University of Geneva, 1211 Geneva, Switzerland; 352 ENT-Department, Leiden University Medical Centre, Leiden, 2300 RC The Netherlands; 353 Leiden Institute for Brain and Cognition, 2300 RC Leiden, The Netherlands; 354 Department of Psychiatry, Icahn School of Medicine at Mount Sinai, New York, NY 10029 USA; 355 Monash Institute of Cognitive and Clinical Neurosciences, Monash University, Clayton, VIC 3168 Australia; 356 Department of Neurology, University of Michigan, Ann Arbor, MI 48104 USA; 357 Department of Biomedical Engineering, University of Michigan, Ann Arbor, MI 48104 USA; 358 Department of Physics, Ohio Wesleyan University, Delaware, OH 43015 USA; 359 Department of Physics, Towson University, Towson, MD 21252 USA; 360 Neuroscience Program, Ohio Wesleyan University, Delaware, OH 43015 USA; 361 Department of Structure and Function of Neural Networks, Korea Brain Research Institute, Daegu, 41068 Republic of Korea; 362 Princeton Neuroscience Institute, Princeton University, Princeton, NJ 08544 USA; 363 Circuit Dynamics and Connectivity Unit, National Institute of Neurological Disorders and Stroke, National Institutes of Health, Bethesda, MD 20824 USA; 364 Computer Science Department, Princeton University, Princeton, NJ 08544 USA; 365 Brain Research Institute, University of Bremen, 28334 Bremen, Germany; 366 Institute for Neurophysics, University of Bremen, 28334 Bremen, Germany; 367 NORMENTa, Institute of Clinical Medicine, University of Oslo, Oslo, Norway; 368 Department of Neurosciences, University of California San Diego, La Jolla, CA USA; 369 Department of Radiology, University of California San Diego, La Jolla, CA USA; 370 Biocomputation Research Group, University of Hertfordshire, Hatfield, UK; 371 Department of Physics, University of Oslo, Oslo, Norway; 372 Simula Research Laboratory and Center for Cardiological Innovation, Oslo, Norway; 373 Multimodal Imaging Laboratory, UC San Diego, La Jolla, CA USA; 374 Department of Psychiatry, Psychotherapy and Psychosomatics, Medical Faculty, RWTH Aachen University, 52074 Aachen, Germany; 375 Department of Physics, Faculty 1, RWTH Aachen University, 52074 Aachen, Germany; 376 Institute of Neuroscience and Medicine (INM-6) and Institute for Advanced Simulation (IAS-6), and JARA BRAIN Institute I, Jülich Research Centre, Jülich, Germany; 377 Rotman Research Institute, Baycrest, Toronto, ON M6A 2E1 Canada; 378 McConnell Brain Imaging Centre, Montreal Neurological Institute, McGill University, Montreal, QC Canada; 379 Department of Computational Neuroscience, University Medical Center Eppendorf, Hamburg, Germany; 380 Department of Health Sciences, Boston University, Boston, MA USA; 381 Department of Psychiatry, Psychotherapy and Psychosomatics, Medical Faculty, RWTH Aachen University, Aachen, Germany; 382 Department of Physics, Faculty 1, RWTH Aachen University, Aachen, Germany; 383 Energy Research Institute @ NTU (ERI@N), Interdisciplinary Graduate School, Nanyang Technological University, Singapore, 639798 Singapore; 384 Fraunhofer IDM @ NTU, Nanyang Technological University, Singapore, 639798 Singapore; 385 School of Electrical and Electronic Engineering, Nanyang Technological University, Singapore, 639798 Singapore; 386 Future Mobility Research Lab, A Joint Initiative of BMW Group & NTU, Nanyang Technological University, Singapore, 639798 Singapore; 387 School of Computer Engineering, Nanyang Technological University, Singapore, 639798 Singapore; 388 Center for Brain and Cognition, Universitat Pompeu Fabra, 08018 Barcelona, Spain; 389 Minerva Research Group Brain Modes, Max Planck Institute for Human Cognitive and Brain Sciences, 04103 Leipzig, Germany; 390 Dept. of Neurology, Charité - University Medicine, 10117 Berlin, Germany; 391 Bernstein Focus State Dependencies of Learning & Bernstein Center for Computational Neuroscience, 10115 Berlin, Germany; 392 Berlin School of Mind and Brain & Mind and Brain Institute, Humboldt University, 10117 Berlin, Germany; 393 Catalan Institution for Advanced Studies (ICREA), Universitat Barcelona, 08010 Barcelona, Spain; 394 Center for Brain Cognition, Universitat Pompeu Fabra, Barcelona, Spain; 395 Center for Music in the Brain, Aarhus University & Royal Academy of Music, Aarhus/Aalborg, Denmark; 396 Faculty of Music, University of Oxford, Oxford, UK; 397 Department of Psychology and Behavioural Sciences, Aarhus University, Aarhus, Denmark; 398 Center of Functionally Integrative Neuroscience, Aarhus University, Aarhus, Denmark; 399 Department of Psychiatry, University of Oxford, Oxford, UK; 400 Computational Neuroscience Unit, Okinawa Institute of Science and Technology, 1919-1 Tancha, Onna-son, Kunigami-gun, Okinawa 904-0495 Japan; 401 Computational Neuroscience Unit, Okinawa Institute of Science and Technology, Okinawa, 904-0411 Japan; 402 Computational Neuroscience Unit, Okinawa Institute of Science and Technology Graduate University, Onna-son, Okinawa Japan; 403 Computational Neuroscience Unit, Okinawa Institute of Science and Technology Graduate University, Onna-son, Okinawa, 904-0495 Japan; 404 Computational Neuroscience Unit, Okinawa Institute of Science and Technology Graduate University, Okinawa, 904-0895 Japan; 405 Champalimaud Neuroscience Programme, Champalimaud Center for the Unknown, Lisbon, Portugal; 406 Center of Biorobotics, Mærsk Mc-Kinney Møller Institute, University of Southern Denmark, Odense, Denmark; 407 Riken Brain Science Institute, 2-1 Hirosawa, Wako, Saitama Japan; 408 IBM, IBM Research - Tokyo, Tokyo, 103-8510 Japan; 409 Department of Medicine, The University of Melbourne, Parkville, VIC 3010 Australia; 410 Department of Statistics, Columbia University, New York, NY USA; 411 Swinburne University of Technology, Hawthorn, VIC 3122 Australia; 412 Department of Electrical and Electronic Engineering, The University of Melbourne, Parkville, VIC 3010 Australia; 413 Department of Otorhinolaryngology – Head and Neck Surgery, University of Pennsylvania, Philadelphia, PA 19104 USA; 414 Department of Neurology, Albert Einstein College of Medicine, Bronx, NY 10461 USA; 415 Systems Neuroscience Group, QIMR Berghofer Medical Research Institute, Herston, QLD 4006 Australia; 416 Department of Bioengineering, Imperial College London, London, UK; 417 Department of Physics, POSTECH, Pohang, 37673 South Korea; 418 Center for Neuroscience, KIST, Seoul, 02792 South Korea; 419 Department of Industrial and Management Engineering, POSTECH, Pohang, 37673 South Korea; 420 Center for Neuroscience, Korea Institute of Science and Technology, Seoul, South Korea; 421 Department of Psychiatry, VA Boston Healthcare System & Harvard Medical School, Brockton, MA USA; 422 Department of Neuroscience, University of Science and Technology, Daejon, South Korea; 423 Center for Neuroscience, Korea Institute of Science and Technology, Seoul, 02792 Korea; 424 Program in Nano Science and Technology, Seoul National University, Seoul, 08826 Korea; 425 Department of Neuroscience, University of Science and Technology, Daejon, 34113 Korea; 426 Center for Neural Science, New York University, 4 Washington Place, 10003, New York NY USA; 427 Institut de Neuroscienes de la Timone (INT), CNRS & Aix-Marseille University, 27 Boulevard Jean Moulin, 13005 Marseille, France; 428 Department of Biotechnology, Indian Institute of Technology Madras, Chennai, Tamil Nadu 600036 India; 429 Department of Electrical and Electronic Engineering, University of Melbourne, Parkville, VIC 3010 Australia; 430 Centre for Neural Engineering, University of Melbourne, Parkville, VIC 3010 Australia; 431 Department of Physiology and Biophysics, University of Washington, Seattle, WA 98195 USA; 432 Howard Hughes Medical Institute, University of Washington, Seattle, WA 98195 USA; 433 Allen Institute for Brain Science, Seattle, WA 98109 USA; 434 Department of Neurobiology, University of Chicago, Chicago, IL 60637 USA; 435 Project-Team BEAGLE, INRIA Rhône-Alpes, 69603 Villeurbanne, France; 436 Department of Statistics, University of Chicago, Chicago, IL 60637 USA; 437 Systems Neuroscience Group, QIMR Berghofer Medical Research Institute, Brisbane, QLD 4006 Australia; 438 Department of Biology, West Virginia University, Morgantown, WV 26506 USA; 439 Department of Mathematics, University of Texas at Arlington, Arlington, TX 76019 USA; 440 Department of Neuroscience, University of Texas Southwestern Medical Center, Dallas, TX 75390 USA; 441 Department of Mathematics, University of Houston, Clear Lake, Houston, TX 77059 USA; 442 Department of Computational Neurosciences, University of Lorraine, 54600 Nancy, France; 443 Computational Neurosciences Laboratory, Ecole Polytechnique Federale de Lausanne, 1015 Lausanne, Switzerland; 444 Program of Brain and Cognitive Engineering, Korea Advanced Institute of Science and Technology (KAIST), Daejeon, 34141 Republic of Korea; 445 Laboratory of Brain and Cognition, National Institute of Mental Health, National Institutes of Health, Bethesda, MD 20892 USA; 446 Institute of Industrial Science, the University of Tokyo, Tokyo, Japan

## Abstract

A1 Functional advantages of cell-type heterogeneity in neural circuits

Tatyana O. Sharpee

A2 Mesoscopic modeling of propagating waves in visual cortex

Alain Destexhe

A3 Dynamics and biomarkers of mental disorders

Mitsuo Kawato

F1 Precise recruitment of spiking output at theta frequencies requires dendritic h-channels in multi-compartment models of oriens-lacunosum/moleculare hippocampal interneurons

Vladislav Sekulić, Frances K. Skinner

F2 Kernel methods in reconstruction of current sources from extracellular potentials for single cells and the whole brains

Daniel K. Wójcik, Chaitanya Chintaluri, Dorottya Cserpán, Zoltán Somogyvári

F3 The synchronized periods depend on intracellular transcriptional repression mechanisms in circadian clocks.

Jae Kyoung Kim, Zachary P. Kilpatrick, Matthew R. Bennett, Kresimir Josić

O1 Assessing irregularity and coordination of spiking-bursting rhythms in central pattern generators

Irene Elices, David Arroyo, Rafael Levi, Francisco B. Rodriguez, Pablo Varona

O2 Regulation of top-down processing by cortically-projecting parvalbumin positive neurons in basal forebrain

Eunjin Hwang, Bowon Kim, Hio-Been Han, Tae Kim, James T. McKenna, Ritchie E. Brown, Robert W. McCarley, Jee Hyun Choi

O3 Modeling auditory stream segregation, build-up and bistability

James Rankin, Pamela Osborn Popp, John Rinzel

O4 Strong competition between tonotopic neural ensembles explains pitch-related dynamics of auditory cortex evoked fields

Alejandro Tabas, André Rupp, Emili Balaguer-Ballester

O5 A simple model of retinal response to multi-electrode stimulation

Matias I. Maturana, David B. Grayden, Shaun L. Cloherty, Tatiana Kameneva, Michael R. Ibbotson, Hamish Meffin

O6 Noise correlations in V4 area correlate with behavioral performance in visual discrimination task

Veronika Koren, Timm Lochmann, Valentin Dragoi, Klaus Obermayer

O7 Input-location dependent gain modulation in cerebellar nucleus neurons

Maria Psarrou, Maria Schilstra, Neil Davey, Benjamin Torben-Nielsen, Volker Steuber

O8 Analytic solution of cable energy function for cortical axons and dendrites

Huiwen Ju, Jiao Yu, Michael L. Hines, Liang Chen, Yuguo Yu

O9 *C. elegans* interactome: interactive visualization of Caenorhabditis elegans worm neuronal network

Jimin Kim, Will Leahy, Eli Shlizerman

O10 Is the model any good? Objective criteria for computational neuroscience model selection

Justas Birgiolas, Richard C. Gerkin, Sharon M. Crook

O11 Cooperation and competition of gamma oscillation mechanisms

Atthaphon Viriyopase, Raoul-Martin Memmesheimer, Stan Gielen

O12 A discrete structure of the brain waves

Yuri Dabaghian, Justin DeVito, Luca Perotti

O13 Direction-specific silencing of the *Drosophila* gaze stabilization system

Anmo J. Kim, Lisa M. Fenk, Cheng Lyu, Gaby Maimon

O14 What does the fruit fly think about values? A model of olfactory associative learning

Chang Zhao, Yves Widmer, Simon Sprecher,Walter Senn

O15 Effects of ionic diffusion on power spectra of local field potentials (LFP)

Geir Halnes, Tuomo Mäki-Marttunen, Daniel Keller, Klas H. Pettersen,Ole A. Andreassen, Gaute T. Einevoll

O16 Large-scale cortical models towards understanding relationship between brain structure abnormalities and cognitive deficits

Yasunori Yamada

O17 Spatial coarse-graining the brain: origin of minicolumns

Moira L. Steyn-Ross, D. Alistair Steyn-Ross

O18 Modeling large-scale cortical networks with laminar structure

Jorge F. Mejias, John D. Murray, Henry Kennedy, Xiao-Jing Wang

O19 Information filtering by partial synchronous spikes in a neural population

Alexandra Kruscha, Jan Grewe, Jan Benda, Benjamin Lindner

O20 Decoding context-dependent olfactory valence in *Drosophila*

Laurent Badel, Kazumi Ohta, Yoshiko Tsuchimoto, Hokto Kazama

P1 Neural network as a scale-free network: the role of a hub

B. Kahng

P2 Hemodynamic responses to emotions and decisions using near-infrared spectroscopy optical imaging

Nicoladie D. Tam

P3 Phase space analysis of hemodynamic responses to intentional movement directions using functional near-infrared spectroscopy (fNIRS) optical imaging technique

Nicoladie D.Tam, Luca Pollonini, George Zouridakis

P4 Modeling jamming avoidance of weakly electric fish

Jaehyun Soh, DaeEun Kim

P5 Synergy and redundancy of retinal ganglion cells in prediction

Minsu Yoo, S. E. Palmer

P6 A neural field model with a third dimension representing cortical depth

Viviana Culmone, Ingo Bojak

P7 Network analysis of a probabilistic connectivity model of the Xenopus tadpole spinal cord

Andrea Ferrario, Robert Merrison-Hort, Roman Borisyuk

P8 The recognition dynamics in the brain

Chang Sub Kim

P9 Multivariate spike train analysis using a positive definite kernel

Taro Tezuka

P10 Synchronization of burst periods may govern slow brain dynamics during general anesthesia

Pangyu Joo

P11 The ionic basis of heterogeneity affects stochastic synchrony

Young-Ah Rho, Shawn D. Burton, G. Bard Ermentrout, Jaeseung Jeong, Nathaniel N. Urban

P12 Circular statistics of noise in spike trains with a periodic component

Petr Marsalek

P14 Representations of directions in EEG-BCI using Gaussian readouts

Hoon-Hee Kim, Seok-hyun Moon, Do-won Lee, Sung-beom Lee, Ji-yong Lee, Jaeseung Jeong

P15 Action selection and reinforcement learning in basal ganglia during reaching movements

Yaroslav I. Molkov, Khaldoun Hamade, Wondimu Teka, William H. Barnett, Taegyo Kim, Sergey Markin, Ilya A. Rybak

P17 Axon guidance: modeling axonal growth in T-Junction assay

Csaba Forro, Harald Dermutz, László Demkó, János Vörös

P19 Transient cell assembly networks encode persistent spatial memories

Yuri Dabaghian, Andrey Babichev

P20 Theory of population coupling and applications to describe high order correlations in large populations of interacting neurons

Haiping Huang

P21 Design of biologically-realistic simulations for motor control

Sergio Verduzco-Flores

P22 Towards understanding the functional impact of the behavioural variability of neurons

Filipa Dos Santos, Peter Andras

P23 Different oscillatory dynamics underlying gamma entrainment deficits in schizophrenia

Christoph Metzner, Achim Schweikard, Bartosz Zurowski

P24 Memory recall and spike frequency adaptation

James P. Roach, Leonard M. Sander, Michal R. Zochowski

P25 Stability of neural networks and memory consolidation preferentially occur near criticality

Quinton M. Skilling, Nicolette Ognjanovski, Sara J. Aton, Michal Zochowski

P26 Stochastic Oscillation in Self-Organized Critical States of Small Systems: Sensitive Resting State in Neural Systems

Sheng-Jun Wang, Guang Ouyang, Jing Guang, Mingsha Zhang, K. Y. Michael Wong, Changsong Zhou

P27 Neurofield: a C++ library for fast simulation of 2D neural field models

Peter A. Robinson, Paula Sanz-Leon, Peter M. Drysdale, Felix Fung, Romesh G. Abeysuriya, Chris J. Rennie, Xuelong Zhao

P28 Action-based grounding: Beyond encoding/decoding in neural code

Yoonsuck Choe, Huei-Fang Yang

P29 Neural computation in a dynamical system with multiple time scales

Yuanyuan Mi, Xiaohan Lin, Si Wu

P30 Maximum entropy models for 3D layouts of orientation selectivity

Joscha Liedtke, Manuel Schottdorf, Fred Wolf

P31 A behavioral assay for probing computations underlying curiosity in rodents

Yoriko Yamamura, Jeffery R. Wickens

P32 Using statistical sampling to balance error function contributions to optimization of conductance-based models

Timothy Rumbell, Julia Ramsey, Amy Reyes, Danel Draguljić, Patrick R. Hof, Jennifer Luebke, Christina M. Weaver

P33 Exploration and implementation of a self-growing and self-organizing neuron network building algorithm

Hu He, Xu Yang, Hailin Ma, Zhiheng Xu, Yuzhe Wang

P34 Disrupted resting state brain network in obese subjects: a data-driven graph theory analysis

Kwangyeol Baek, Laurel S. Morris, Prantik Kundu, Valerie Voon

P35 Dynamics of cooperative excitatory and inhibitory plasticity

Everton J. Agnes, Tim P. Vogels

P36 Frequency-dependent oscillatory signal gating in feed-forward networks of integrate-and-fire neurons

William F. Podlaski, Tim P. Vogels

P37 Phenomenological neural model for adaptation of neurons in area IT

Martin Giese, Pradeep Kuravi, Rufin Vogels

P38 ICGenealogy: towards a common topology of neuronal ion channel function and genealogy in model and experiment

Alexander Seeholzer, William Podlaski, Rajnish Ranjan, Tim Vogels

P39 Temporal input discrimination from the interaction between dynamic synapses and neural subthreshold oscillations

Joaquin J. Torres, Fabiano Baroni, Roberto Latorre, Pablo Varona

P40 Different roles for transient and sustained activity during active visual processing

Bart Gips, Eric Lowet, Mark J. Roberts, Peter de Weerd, Ole Jensen, Jan van der Eerden

P41 Scale-free functional networks of 2D Ising model are highly robust against structural defects: neuroscience implications

Abdorreza Goodarzinick, Mohammad D. Niry, Alireza Valizadeh

P42 High frequency neuron can facilitate propagation of signal in neural networks

Aref Pariz, Shervin S. Parsi, Alireza Valizadeh

P43 Investigating the effect of Alzheimer’s disease related amyloidopathy on gamma oscillations in the CA1 region of the hippocampus

Julia M. Warburton, Lucia Marucci, Francesco Tamagnini, Jon Brown, Krasimira Tsaneva-Atanasova

P44 Long-tailed distributions of inhibitory and excitatory weights in a balanced network with eSTDP and iSTDP

Florence I. Kleberg, Jochen Triesch

P45 Simulation of EMG recording from hand muscle due to TMS of motor cortex

Bahar Moezzi, Nicolangelo Iannella, Natalie Schaworonkow, Lukas Plogmacher, Mitchell R. Goldsworthy, Brenton Hordacre, Mark D. McDonnell, Michael C. Ridding, Jochen Triesch

P46 Structure and dynamics of axon network formed in primary cell culture

Martin Zapotocky, Daniel Smit, Coralie Fouquet, Alain Trembleau

P47 Efficient signal processing and sampling in random networks that generate variability

Sakyasingha Dasgupta, Isao Nishikawa, Kazuyuki Aihara, Taro Toyoizumi

P48 Modeling the effect of riluzole on bursting in respiratory neural networks

Daniel T. Robb, Nick Mellen, Natalia Toporikova

P49 Mapping relaxation training using effective connectivity analysis

Rongxiang Tang, Yi-Yuan Tang

P50 Modeling neuron oscillation of implicit sequence learning

Guangsheng Liang, Seth A. Kiser, James H. Howard, Jr., Yi-Yuan Tang

P51 The role of cerebellar short-term synaptic plasticity in the pathology and medication of downbeat nystagmus

Julia Goncharenko, Neil Davey, Maria Schilstra, Volker Steuber

P52 Nonlinear response of noisy neurons

Sergej O. Voronenko, Benjamin Lindner

P53 Behavioral embedding suggests multiple chaotic dimensions underlie *C. elegans* locomotion

Tosif Ahamed, Greg Stephens

P54 Fast and scalable spike sorting for large and dense multi-electrodes recordings

Pierre Yger, Baptiste Lefebvre, Giulia Lia Beatrice Spampinato, Elric Esposito, Marcel Stimberg et Olivier Marre

P55 Sufficient sampling rates for fast hand motion tracking

Hansol Choi, Min-Ho Song

P56 Linear readout of object manifolds

SueYeon Chung, Dan D. Lee, Haim Sompolinsky

P57 Differentiating models of intrinsic bursting and rhythm generation of the respiratory pre-Bötzinger complex using phase response curves

Ryan S. Phillips, Jeffrey Smith

P58 The effect of inhibitory cell network interactions during theta rhythms on extracellular field potentials in CA1 hippocampus

Alexandra Pierri Chatzikalymniou, Katie Ferguson, Frances K. Skinner

P59 Expansion recoding through sparse sampling in the cerebellar input layer speeds learning

N. Alex Cayco Gajic, Claudia Clopath, R. Angus Silver

P60 A set of curated cortical models at multiple scales on Open Source Brain

Padraig Gleeson, Boris Marin, Sadra Sadeh, Adrian Quintana, Matteo Cantarelli, Salvador Dura-Bernal, William W. Lytton, Andrew Davison, R. Angus Silver

P61 A synaptic story of dynamical information encoding in neural adaptation

Luozheng Li, Wenhao Zhang, Yuanyuan Mi, Dahui Wang, Si Wu

P62 Physical modeling of rule-observant rodent behavior

Youngjo Song, Sol Park, Ilhwan Choi, Jaeseung Jeong, Hee-sup Shin

P64 Predictive coding in area V4 and prefrontal cortex explains dynamic discrimination of partially occluded shapes

Hannah Choi, Anitha Pasupathy, Eric Shea-Brown

P65 Stability of FORCE learning on spiking and rate-based networks

Dongsung Huh, Terrence J. Sejnowski

P66 Stabilising STDP in striatal neurons for reliable fast state recognition in noisy environments

Simon M. Vogt, Arvind Kumar, Robert Schmidt

P67 Electrodiffusion in one- and two-compartment neuron models for characterizing cellular effects of electrical stimulation

Stephen Van Wert, Steven J. Schiff

P68 STDP improves speech recognition capabilities in spiking recurrent circuits parameterized via differential evolution Markov Chain Monte Carlo

Richard Veale, Matthias Scheutz

P69 Bidirectional transformation between dominant cortical neural activities and phase difference distributions

Sang Wan Lee

P70 Maturation of sensory networks through homeostatic structural plasticity

Júlia Gallinaro, Stefan Rotter

P71 Corticothalamic dynamics: structure, number of solutions and stability of steady-state solutions in the space of synaptic couplings

Paula Sanz-Leon, Peter A. Robinson

P72 Optogenetic versus electrical stimulation of the parkinsonian basal ganglia. Computational study

Leonid L. Rubchinsky, Chung Ching Cheung, Shivakeshavan Ratnadurai-Giridharan

P73 Exact spike-timing distribution reveals higher-order interactions of neurons

Safura Rashid Shomali, Majid Nili Ahmadabadi, Hideaki Shimazaki, S. Nader Rasuli

P74 Neural mechanism of visual perceptual learning using a multi-layered neural network

Xiaochen Zhao, Malte J. Rasch

P75 Inferring collective spiking dynamics from mostly unobserved systems

Jens Wilting, Viola Priesemann

P76 How to infer distributions in the brain from subsampled observations

Anna Levina, Viola Priesemann

P77 Influences of embedding and estimation strategies on the inferred memory of single spiking neurons

Lucas Rudelt, Joseph T. Lizier, Viola Priesemann

P78 A nearest-neighbours based estimator for transfer entropy between spike trains

Joseph T. Lizier, Richard E. Spinney, Mikail Rubinov, Michael Wibral, Viola Priesemann

P79 Active learning of psychometric functions with multinomial logistic models

Ji Hyun Bak, Jonathan Pillow

P81 Inferring low-dimensional network dynamics with variational latent Gaussian process

Yuan Zaho, Il Memming Park

P82 Computational investigation of energy landscapes in the resting state subcortical brain network

Jiyoung Kang, Hae-Jeong Park

P83 Local repulsive interaction between retinal ganglion cells can generate a consistent spatial periodicity of orientation map

Jaeson Jang, Se-Bum Paik

P84 Phase duration of bistable perception reveals intrinsic time scale of perceptual decision under noisy condition

Woochul Choi, Se-Bum Paik

P85 Feedforward convergence between retina and primary visual cortex can determine the structure of orientation map

Changju Lee, Jaeson Jang, Se-Bum Paik

P86 Computational method classifying neural network activity patterns for imaging data

Min Song, Hyeonsu Lee, Se-Bum Paik

P87 Symmetry of spike-timing-dependent-plasticity kernels regulates volatility of memory

Youngjin Park, Woochul Choi^,^ Se-Bum Paik

P88 Effects of time-periodic coupling strength on the first-spike latency dynamics of a scale-free network of stochastic Hodgkin-Huxley neurons

Ergin Yilmaz, Veli Baysal, Mahmut Ozer

P89 Spectral properties of spiking responses in V1 and V4 change within the trial and are highly relevant for behavioral performance

Veronika Koren, Klaus Obermayer

P90 Methods for building accurate models of individual neurons

Daniel Saska, Thomas Nowotny

P91 A full size mathematical model of the early olfactory system of honeybees

Ho Ka Chan, Alan Diamond, Thomas Nowotny

P92 Stimulation-induced tuning of ongoing oscillations in spiking neural networks

Christoph S. Herrmann, Micah M. Murray, Silvio Ionta, Axel Hutt, Jérémie Lefebvre

P93 Decision-specific sequences of neural activity in balanced random networks driven by structured sensory input

Philipp Weidel, Renato Duarte, Abigail Morrison

P94 Modulation of tuning induced by abrupt reduction of SST cell activity

Jung H. Lee, Ramakrishnan Iyer, Stefan Mihalas

P95 The functional role of VIP cell activation during locomotion

Jung H. Lee, Ramakrishnan Iyer, Christof Koch, Stefan Mihalas

P96 Stochastic inference with spiking neural networks

Mihai A. Petrovici, Luziwei Leng, Oliver Breitwieser, David Stöckel, Ilja Bytschok, Roman Martel, Johannes Bill, Johannes Schemmel, Karlheinz Meier

P97 Modeling orientation-selective electrical stimulation with retinal prostheses

Timothy B. Esler, Anthony N. Burkitt, David B. Grayden, Robert R. Kerr, Bahman Tahayori, Hamish Meffin

P98 Ion channel noise can explain firing correlation in auditory nerves

Bahar Moezzi, Nicolangelo Iannella, Mark D. McDonnell

P99 Limits of temporal encoding of thalamocortical inputs in a neocortical microcircuit

Max Nolte, Michael W. Reimann, Eilif Muller, Henry Markram

P100 On the representation of arm reaching movements: a computational model

Antonio Parziale, Rosa Senatore, Angelo Marcelli

P101 A computational model for investigating the role of cerebellum in acquisition and retention of motor behavior

Rosa Senatore, Antonio Parziale, Angelo Marcelli

P102 The emergence of semantic categories from a large-scale brain network of semantic knowledge

K. Skiker, M. Maouene

P103 Multiscale modeling of M1 multitarget pharmacotherapy for dystonia

Samuel A. Neymotin, Salvador Dura-Bernal, Alexandra Seidenstein, Peter Lakatos, Terence D. Sanger, William W. Lytton

P104 Effect of network size on computational capacity

Salvador Dura-Bernal, Rosemary J. Menzies, Campbell McLauchlan, Sacha J. van Albada, David J. Kedziora, Samuel Neymotin, William W. Lytton, Cliff C. Kerr

P105 NetPyNE: a Python package for NEURON to facilitate development and parallel simulation of biological neuronal networks

Salvador Dura-Bernal, Benjamin A. Suter, Samuel A. Neymotin, Cliff C. Kerr, Adrian Quintana, Padraig Gleeson, Gordon M. G. Shepherd, William W. Lytton

P107 Inter-areal and inter-regional inhomogeneity in co-axial anisotropy of Cortical Point Spread in human visual areas

Juhyoung Ryu, Sang-Hun Lee

P108 Two bayesian quanta of uncertainty explain the temporal dynamics of cortical activity in the non-sensory areas during bistable perception

Joonwon Lee, Sang-Hun Lee

P109 Optimal and suboptimal integration of sensory and value information in perceptual decision making

Hyang Jung Lee, Sang-Hun Lee

P110 A Bayesian algorithm for phoneme Perception and its neural implementation

Daeseob Lim, Sang-Hun Lee

P111 Complexity of EEG signals is reduced during unconsciousness induced by ketamine and propofol

Jisung Wang, Heonsoo Lee

P112 Self-organized criticality of neural avalanche in a neural model on complex networks

Nam Jung, Le Anh Quang, Seung Eun Maeng, Tae Ho Lee, Jae Woo Lee

P113 Dynamic alterations in connection topology of the hippocampal network during ictal-like epileptiform activity in an in vitro rat model

Chang-hyun Park, Sora Ahn, Jangsup Moon, Yun Seo Choi, Juhee Kim, Sang Beom Jun, Seungjun Lee, Hyang Woon Lee

P114 Computational model to replicate seizure suppression effect by electrical stimulation

Sora Ahn, Sumin Jo, Eunji Jun, Suin Yu, Hyang Woon Lee, Sang Beom Jun, Seungjun Lee

P115 Identifying excitatory and inhibitory synapses in neuronal networks from spike trains using sorted local transfer entropy

Felix Goetze, Pik-Yin Lai

P116 Neural network model for obstacle avoidance based on neuromorphic computational model of boundary vector cell and head direction cell

Seonghyun Kim, Jeehyun Kwag

P117 Dynamic gating of spike pattern propagation by Hebbian and anti-Hebbian spike timing-dependent plasticity in excitatory feedforward network model

Hyun Jae Jang, Jeehyun Kwag

P118 Inferring characteristics of input correlations of cells exhibiting up-down state transitions in the rat striatum

Marko Filipović, Ramon Reig, Ad Aertsen, Gilad Silberberg, Arvind Kumar

P119 Graph properties of the functional connected brain under the influence of Alzheimer’s disease

Claudia Bachmann, Simone Buttler, Heidi Jacobs, Kim Dillen, Gereon R. Fink, Juraj Kukolja, Abigail Morrison

P120 Learning sparse representations in the olfactory bulb

Daniel Kepple, Hamza Giaffar, Dima Rinberg, Steven Shea, Alex Koulakov

P121 Functional classification of homologous basal-ganglia networks

Jyotika Bahuguna,Tom Tetzlaff, Abigail Morrison, Arvind Kumar, Jeanette Hellgren Kotaleski

P122 Short term memory based on multistability

Tim Kunze, Andre Peterson, Thomas Knösche

P123 A physiologically plausible, computationally efficient model and simulation software for mammalian motor units

Minjung Kim, Hojeong Kim

P125 Decoding laser-induced somatosensory information from EEG

Ji Sung Park, Ji Won Yeon, Sung-Phil Kim

P126 Phase synchronization of alpha activity for EEG-based personal authentication

Jae-Hwan Kang, Chungho Lee, Sung-Phil Kim

P129 Investigating phase-lags in sEEG data using spatially distributed time delays in a large-scale brain network model

Andreas Spiegler, Spase Petkoski, Matias J. Palva, Viktor K. Jirsa

P130 Epileptic seizures in the unfolding of a codimension-3 singularity

Maria L. Saggio, Silvan F. Siep, Andreas Spiegler, William C. Stacey, Christophe Bernard, Viktor K. Jirsa

P131 Incremental dimensional exploratory reasoning under multi-dimensional environment

Oh-hyeon Choung, Yong Jeong

P132 A low-cost model of eye movements and memory in personal visual cognition

Yong-il Lee, Jaeseung Jeong

P133 Complex network analysis of structural connectome of autism spectrum disorder patients

Su Hyun Kim, Mir Jeong, Jaeseung Jeong

P134 Cognitive motives and the neural correlates underlying human social information transmission, gossip

Jeungmin Lee, Jaehyung Kwon, Jerald D. Kralik, Jaeseung Jeong

P135 EEG hyperscanning detects neural oscillation for the social interaction during the economic decision-making

Jaehwan Jahng, Dong-Uk Hwang, Jaeseung Jeong

P136 Detecting purchase decision based on hyperfrontality of the EEG

Jae-Hyung Kwon, Sang-Min Park, Jaeseung Jeong

P137 Vulnerability-based critical neurons, synapses, and pathways in the *Caenorhabditis elegans* connectome

Seongkyun Kim, Hyoungkyu Kim, Jerald D. Kralik, Jaeseung Jeong

P138 Motif analysis reveals functionally asymmetrical neurons in *C. elegans*

Pyeong Soo Kim, Seongkyun Kim, Hyoungkyu Kim, Jaeseung Jeong

P139 Computational approach to preference-based serial decision dynamics: do temporal discounting and working memory affect it?

Sangsup Yoon, Jaehyung Kwon, Sewoong Lim, Jaeseung Jeong

P141 Social stress induced neural network reconfiguration affects decision making and learning in zebrafish

Choongseok Park, Thomas Miller, Katie Clements, Sungwoo Ahn, Eoon Hye Ji, Fadi A. Issa

P142 Descriptive, generative, and hybrid approaches for neural connectivity inference from neural activity data

JeongHun Baek, Shigeyuki Oba, Junichiro Yoshimoto, Kenji Doya, Shin Ishii

P145 Divergent-convergent synaptic connectivities accelerate coding in multilayered sensory systems

Thiago S. Mosqueiro, Martin F. Strube-Bloss, Brian Smith, Ramon Huerta

P146 Swinging networks

Michal Hadrava, Jaroslav Hlinka

P147 Inferring dynamically relevant motifs from oscillatory stimuli: challenges, pitfalls, and solutions

Hannah Bos, Moritz Helias

P148 Spatiotemporal mapping of brain network dynamics during cognitive tasks using magnetoencephalography and deep learning

Charles M. Welzig, Zachary J. Harper

P149 Multiscale complexity analysis for the segmentation of MRI images

Won Sup Kim, In-Seob Shin, Hyeon-Man Baek, Seung Kee Han

P150 A neuro-computational model of emotional attention

René Richter, Julien Vitay, Frederick Beuth, Fred H. Hamker

P151 Multi-site delayed feedback stimulation in parkinsonian networks

Kelly Toppin, Yixin Guo

P152 Bistability in Hodgkin–Huxley-type equations

Tatiana Kameneva, Hamish Meffin, Anthony N. Burkitt, David B. Grayden

P153 Phase changes in postsynaptic spiking due to synaptic connectivity and short term plasticity: mathematical analysis of frequency dependency

Mark D. McDonnell, Bruce P. Graham

P154 Quantifying resilience patterns in brain networks: the importance of directionality

Penelope J. Kale, Leonardo L. Gollo

P155 Dynamics of rate-model networks with separate excitatory and inhibitory populations

Merav Stern, L. F. Abbott

P156 A model for multi-stable dynamics in action recognition modulated by integration of silhouette and shading cues

Leonid A. Fedorov, Martin A. Giese

P157 Spiking model for the interaction between action recognition and action execution

Mohammad Hovaidi Ardestani, Martin Giese

P158 Surprise-modulated belief update: how to learn within changing environments?

Mohammad Javad Faraji, Kerstin Preuschoff, Wulfram Gerstner

P159 A fast, stochastic and adaptive model of auditory nerve responses to cochlear implant stimulation

Margriet J. van Gendt, Jeroen J. Briaire, Randy K. Kalkman, Johan H. M. Frijns

P160 Quantitative comparison of graph theoretical measures of simulated and empirical functional brain networks

Won Hee Lee, Sophia Frangou

P161 Determining discriminative properties of fMRI signals in schizophrenia using highly comparative time-series analysis

Ben D. Fulcher, Patricia H. P. Tran, Alex Fornito

P162 Emergence of narrowband LFP oscillations from completely asynchronous activity during seizures and high-frequency oscillations

Stephen V. Gliske, William C. Stacey, Eugene Lim, Katherine A. Holman, Christian G. Fink

P163 Neuronal diversity in structure and function: cross-validation of anatomical and physiological classification of retinal ganglion cells in the mouse

Jinseop S. Kim, Shang Mu, Kevin L. Briggman, H. Sebastian Seung, the EyeWirers

P164 Analysis and modelling of transient firing rate changes in area MT in response to rapid stimulus feature changes

Detlef Wegener, Lisa Bohnenkamp, Udo A. Ernst

P165 Step-wise model fitting accounting for high-resolution spatial measurements: construction of a layer V pyramidal cell model with reduced morphology

Tuomo Mäki-Marttunen, Geir Halnes, Anna Devor, Christoph Metzner, Anders M. Dale, Ole A. Andreassen, Gaute T. Einevoll

P166 Contributions of schizophrenia-associated genes to neuron firing and cardiac pacemaking: a polygenic modeling approach

Tuomo Mäki-Marttunen, Glenn T. Lines, Andy Edwards, Aslak Tveito, Anders M. Dale, Gaute T. Einevoll, Ole A. Andreassen

P167 Local field potentials in a 4 × 4 mm^2^ multi-layered network model

Espen Hagen, Johanna Senk, Sacha J. van Albada, Markus Diesmann

P168 A spiking network model explains multi-scale properties of cortical dynamics

Maximilian Schmidt, Rembrandt Bakker, Kelly Shen, Gleb Bezgin, Claus-Christian Hilgetag, Markus Diesmann, Sacha Jennifer van Albada

P169 Using joint weight-delay spike-timing dependent plasticity to find polychronous neuronal groups

Haoqi Sun, Olga Sourina, Guang-Bin Huang, Felix Klanner, Cornelia Denk

P170 Tensor decomposition reveals RSNs in simulated resting state fMRI

Katharina Glomb, Adrián Ponce-Alvarez, Matthieu Gilson, Petra Ritter, Gustavo Deco

P171 Getting in the groove: testing a new model-based method for comparing task-evoked vs resting-state activity in fMRI data on music listening

Matthieu Gilson, Maria AG Witek, Eric F. Clarke, Mads Hansen, Mikkel Wallentin, Gustavo Deco, Morten L. Kringelbach^,^ Peter Vuust

P172 STochastic engine for pathway simulation (STEPS) on massively parallel processors

Guido Klingbeil, Erik De Schutter

P173 Toolkit support for complex parallel spatial stochastic reaction–diffusion simulation in STEPS

Weiliang Chen, Erik De Schutter

P174 Modeling the generation and propagation of Purkinje cell dendritic spikes caused by parallel fiber synaptic input

Yunliang Zang, Erik De Schutter

P175 Dendritic morphology determines how dendrites are organized into functional subunits

Sungho Hong, Akira Takashima, Erik De Schutter

P176 A model of Ca^2+^/calmodulin-dependent protein kinase II activity in long term depression at Purkinje cells

Criseida Zamora, Andrew R. Gallimore, Erik De Schutter

P177 Reward-modulated learning of population-encoded vectors for insect-like navigation in embodied agents

Dennis Goldschmidt, Poramate Manoonpong, Sakyasingha Dasgupta

P178 Data-driven neural models part II: connectivity patterns of human seizures

Philippa J. Karoly, Dean R. Freestone, Daniel Soundry, Levin Kuhlmann, Liam Paninski, Mark Cook

P179 Data-driven neural models part I: state and parameter estimation

Dean R. Freestone, Philippa J. Karoly, Daniel Soundry, Levin Kuhlmann, Mark Cook

P180 Spectral and spatial information processing in human auditory streaming

Jaejin Lee, Yonatan I. Fishman, Yale E. Cohen

P181 A tuning curve for the global effects of local perturbations in neural activity: Mapping the systems-level susceptibility of the brain

Leonardo L. Gollo, James A. Roberts, Luca Cocchi

P182 Diverse homeostatic responses to visual deprivation mediated by neural ensembles

Yann Sweeney, Claudia Clopath

P183 Opto-EEG: a novel method for investigating functional connectome in mouse brain based on optogenetics and high density electroencephalography

Soohyun Lee, Woo-Sung Jung, Jee Hyun Choi

P184 Biphasic responses of frontal gamma network to repetitive sleep deprivation during REM sleep

Bowon Kim, Youngsoo Kim, Eunjin Hwang, Jee Hyun Choi

P185 Brain-state correlate and cortical connectivity for frontal gamma oscillations in top-down fashion assessed by auditory steady-state response

Younginha Jung, Eunjin Hwang, Yoon-Kyu Song, Jee Hyun Choi

P186 Neural field model of localized orientation selective activation in V1

James Rankin, Frédéric Chavane

P187 An oscillatory network model of Head direction and Grid cells using locomotor inputs

Karthik Soman, Vignesh Muralidharan, V. Srinivasa Chakravarthy

P188 A computational model of hippocampus inspired by the functional architecture of basal ganglia

Karthik Soman, Vignesh Muralidharan, V. Srinivasa Chakravarthy

P189 A computational architecture to model the microanatomy of the striatum and its functional properties

Sabyasachi Shivkumar, Vignesh Muralidharan, V. Srinivasa Chakravarthy

P190 A scalable cortico-basal ganglia model to understand the neural dynamics of targeted reaching

Vignesh Muralidharan, Alekhya Mandali, B. Pragathi Priyadharsini, Hima Mehta, V. Srinivasa Chakravarthy

P191 Emergence of radial orientation selectivity from synaptic plasticity

Catherine E. Davey, David B. Grayden, Anthony N. Burkitt

P192 How do hidden units shape effective connections between neurons?

Braden A. W. Brinkman, Tyler Kekona, Fred Rieke, Eric Shea-Brown, Michael Buice

P193 Characterization of neural firing in the presence of astrocyte-synapse signaling

Maurizio De Pittà, Hugues Berry, Nicolas Brunel

P194 Metastability of spatiotemporal patterns in a large-scale network model of brain dynamics

James A. Roberts, Leonardo L. Gollo, Michael Breakspear

P195 Comparison of three methods to quantify detection and discrimination capacity estimated from neural population recordings

Gary Marsat, Jordan Drew, Phillip D. Chapman, Kevin C. Daly, Samual P. Bradley

P196 Quantifying the constraints for independent evoked and spontaneous NMDA receptor mediated synaptic transmission at individual synapses

Sat Byul Seo, Jianzhong Su, Ege T. Kavalali, Justin Blackwell

P199 Gamma oscillation via adaptive exponential integrate-and-fire neurons

LieJune Shiau, Laure Buhry, Kanishka Basnayake

P200 Visual face representations during memory retrieval compared to perception

Sue-Hyun Lee, Brandon A. Levy, Chris I. Baker

P201 Top-down modulation of sequential activity within packets modeled using avalanche dynamics

Timothée Leleu, Kazuyuki Aihara

Q28 An auto-encoder network realizes sparse features under the influence of desynchronized vascular dynamics

Ryan T. Philips, Karishma Chhabria, V. Srinivasa Chakravarthy

## A1 Functional advantages of cell-type heterogeneity in neural circuits

### Tatyana O. Sharpee^1^

#### ^1^Computational Neurobiology Laboratory, The Salk Institute for Biological Studies, San Diego, CA, USA

##### **Correspondence**: Tatyana O. Sharpee - sharpee@snl.salk.edu

*BMC Neuroscience* 2016, **17(Suppl 1)**:A1

Neural circuits are notorious for the complexity of their organization. Part of this complexity is related to the number of different cell types that work together to encode stimuli. I will discuss theoretical results that point to functional advantages of splitting neural populations into subtypes, both in feedforward and recurrent networks. These results outline a framework for categorizing neuronal types based on their functional properties. Such classification scheme could augment classification schemes based on molecular, anatomical, and electrophysiological properties.

## A2 Mesoscopic modeling of propagating waves in visual cortex

### Alain Destexhe^1,2^

#### ^1^UNIC, CNRS, Gif sur Yvette, France; ^2^The European Institute for Theoretical Neuroscience (EITN), Paris, France

##### **Correspondence**: Alain Destexhe - destexhe@unic.cnrs-gif.fr

*BMC Neuroscience* 2016, **17(Suppl 1)**:A2

Propagating waves are large-scale phenomena widely seen in the nervous system, in both anesthetized and awake or sleeping states. Recently, the presence of propagating waves at the scale of microns–millimeters was demonstrated in the primary visual cortex (V1) of macaque monkey. Using a combination of voltage-sensitive dye (VSD) imaging in awake monkey V1 and model-based analysis, we showed that virtually every visual input is followed by a propagating wave (Muller et al., Nat Comm 2014). The wave was confined within V1, and was consistent and repeatable for a given input. Interestingly, two propagating waves always interact in a suppressive fashion, and sum sublinearly. This is in agreement with the general suppressive effect seen in other circumstances in V1 (Bair et al., J Neurosci 2003; Reynaud et al., J Neurosci 2012).

To investigate possible mechanisms for this suppression we have designed mean-field models to directly integrate the VSD experiments. Because the VSD signal is primarily caused by the summed voltage of all membranes, it represents an ideal case for mean-field models. However, usual mean-field models are based on neuronal transfer functions such as the well-known sigmoid function, or functions estimated from very simple models. Any error in the transfer function may result in wrong predictions by the corresponding mean-field model. To palliate this caveat, we have obtained semi-analytic forms of the transfer function of more realistic neuron models. We found that the same mathematical template can capture the transfer function for models such as the integrate-and-fire (IF) model, the adaptive exponential (AdEx) model, up to Hodgkin–Huxley (HH) type models, all with conductance-based inputs.

Using these transfer functions we have built “realistic” mean-field models for networks with two populations of neurons, the regular-spiking (RS) excitatory neurons, showing spike frequency adaptation, and the fast-spiking (FS) inhibitory neurons. This mean-field model can reproduce the propagating waves in V1, due to horizontal interactions, as shown previously using IF networks. This mean-field model also reproduced the suppressive interactions between propagating waves. The mechanism of suppression was based on the preferential recruitment of inhibitory cells over excitatory cells by afferent activity, which acted through the conductance-based shunting effect of the two waves onto one another. The suppression was negligible in networks with identical models for excitatory and inhibitory cells (such as IF networks). This suggests that the suppressive effect is a general phenomenon due to the higher excitability of inhibitory neurons in cortex, in line with previous models (Ozeki et al., Neuron 2009).

Work done in collaboration with Yann Zerlaut (UNIC) for modeling, Sandrine Chemla and Frederic Chavane (CNRS, Marseille) for in vivo experiments. Supported by CNRS and the European Commission (Human Brain Project).

## A3 Dynamics and biomarkers of mental disorders

### Mitsuo Kawato^1^

#### ^1^ATR Computational Neuroscience Laboratories, 2-2 Hikaridai, Seika-cho, Soraku-gun, Kyoto 619-0288, Japan

##### **Correspondence**: Mitsuo Kawato - kawato@hip.atr.co.jp

*BMC Neuroscience* 2016, **17(Suppl 1)**:A3

Current diagnoses of mental disorders are made in a categorical way, as exemplified by DSM-5, but many difficulties have been encountered in such categorical regimes: the high percentage of comorbidities, usage of the same drug for multiple disorders, the lack of any validated animal model, and the situation where no epoch-making drug has been developed in the past 30 years. NIMH started RDoC (research domain criterion) to overcome these problems [1], and some successful results have been obtained, including common genetic risk loci [2] and common neuroanatomical changes for multiple disorders [3] as well as psychosis biotypes [4].

In contrast to the currently dominant molecular biology approach, which basically assumes one-to-one mapping between genes and disorders, I postulate the following dynamics-based view of psychiatric disorders. Our brain is a nonlinear dynamical system that can generate spontaneous spatiotemporal activities. The dynamical system is characterized by multiple stable attractors, only one of which corresponds to a healthy or typically developed state. The others are pathological states.

The most promising research approach within the above dynamical view is to combine resting-state functional magnetic resonance imaging, machine learning, big data, and sophisticated neurofeedback. Yahata et al. developed an ASD biomarker using only 16/9730 functional connections, and it did not generalize to MDD or ADHD but moderately to schizophrenia [5]. Yamashita’s regression model of working memory ability from functional connections [6] generalized to schizophrenia and reproduced the severity of working-memory deficits of four psychiatric disorders (in preparation).

With the further development of machine learning algorithms and accumulation of reliable datasets, we hope to obtain a comprehensive landscape of many psychiatric and neurodevelopmental disorders. Guided by this full-spectrum structure, a tailor-made neurofeedback therapy should be optimized for each patient [7].

**References**

1. Insel T, Cuthbert B, Garvey M., et al. Research domain criteria (RDoC): toward a new classification framework for research on mental disorders. Am J Psychiatry. 2010;167:748–51.
2. Cross-disorder group of the psychiatric genomics consortium: identification of risk loci with shared effects on five major psychiatric disorders: a genome-wide analysis. Lancet. 2013;381:1371–9.
3. Goodkind M, et al. Identification of a common neurobiological substrate for mental illness. JAMA Psychiatry. 2015;72:305–15.
4. Clementz BA, et al. Identification of distinct psychosis biotypes using brain-based biomarkers. Am J Psychiatry. 2016;173:373–84.
5. Yahata N, Morimoto J, Hashimoto R, Lisi G, Shibata K, Kawakubo Y, Kuwabara H, Kuroda M, Yamada T, Megumi F, Imamizu H, Nanez JE, Takahashi H, Okamoto Y, Kasai K, Kato N, Sasaki Y, Watanabe T, Kawato M: A small number of abnormal brain connections predicts adult autism spectrum disorder. Nature Commun. 2016;7:11254. doi:10.1038/ncomms11254.
6. Yamashita M, Kawato M, Imamizu H. Predicting learning plateau of working memory from whole-brain intrinsic network connectivity patterns. Sci Rep. 2015;5(7622). doi:10.1038/srep07622.
7. ATR Brain Information Communication Research Laboratory Group. DecNef  Project. Available at http://www.cns.atr.jp/decnefpro/ (2016).

## F1 Precise recruitment of spiking output at theta frequencies requires dendritic h-channels in multi-compartment models of oriens-lacunosum/moleculare hippocampal interneurons

### Vladislav Sekulić^1,2^, Frances K. Skinner^1,2,3^

#### ^1^Krembil Research Institute, University Health Network, Toronto, Ontario, Canada, M5T 2S8; ^2^Department of Physiology, University of Toronto, Toronto, Ontario, Canada, M5S 1A8; ^3^ Department of Medicine (Neurology), University of Toronto, Toronto, Ontario, Canada, M5T 2S8

##### **Correspondence**: Vladislav Sekulić - vlad.sekulic@utoronto.ca

*BMC Neuroscience* 2016, **17(Suppl 1)**:F1

The theta rhythm (4–12 Hz) is a prominent network oscillation observed in the mammalian hippocampus and is correlated with spatial navigation and mnemonic processing. Inhibitory interneurons of the hippocampus fire action potentials at specific phases of the theta rhythm, pointing to distinct functional roles of interneurons in shaping this rhythmic activity. One hippocampal interneuron type, the oriens-lacunosum/moleculare (O-LM) cell, provides direct feedback inhibition and regulation of pyramidal cell activity in the CA1 region. O-LM cells express the hyperpolarization-activated, mixed-cation current (*I*_h_) and, in vitro, demonstrate spontaneous firing at theta that is impaired upon blockade of *I*_h_. Work using dynamic clamp has shown that in the presence of frequency-modulated artificial synaptic inputs, O-LM cells exhibit a spiking resonance at theta frequencies that is not dependent on *I*_h_ [1]. However, due to the somatic injection limitation of dynamic clamp, the study could not examine the potential contributions of putative dendritic *I*_h_ or the integration of dendritically-located synaptic inputs. To overcome this, we have used a database of previously developed multi-compartment computational models of O-LM cells [2].

We situated our OLM cell models in an in vivo-like context by injecting Poisson-based synaptic background activities throughout their dendritic arbors. Excitatory and inhibitory synaptic weights were tuned to produce similar baseline activity prior to modulation of the inhibitory synaptic process at various frequencies (2–30 Hz). We found that models with dendritic inputs expressed enhanced resonant firing at theta frequencies compared to models with somatic inputs. We then performed detailed analyses on the outputs of the models with dendritic inputs to further elucidate these results with respect to *I*_h_ distributions. The ability of the models to be recruited at the modulated input frequencies was quantified using the rotation number, or average number of spikes across all input cycles. Models with somatodendritic *I*_h_ were recruited at >50 % of the input cycles for a wider range of theta frequencies (3–9 Hz) compared to models with somatic *I*_h_ only (3–4 Hz). Models with somatodendritic *I*_h_ also exhibited a wider range of theta frequencies for which phase-locked output (vector strength >0.75) was observed (4–12 Hz), compared to models with somatic *I*_h_ (3–5 Hz). Finally, the phase of firing of models with somatodendritic *I*_h_ given 8–10 Hz modulated input was delayed 180–230° relative to the time of release from inhibitory synaptic input.

O-LM cells receive phasic inhibitory inputs at theta frequencies from a subpopulation of parvalbumin-positive GABAergic interneurons in the medial septum (MS) timed to the peak of hippocampal theta, as measured in the stratum pyramidale layer [3]. Furthermore, O-LM cells fire at the trough of hippocampal pyramidal layer theta in vivo [4], an approximate 180˚ phase delay from the MS inputs, corresponding to the phase delay in our models with somatodendritic *I*_h_. Our results suggest that, given dendritic synaptic inputs, O-LM cells require somatodendritic *I*_h_ channel expression to be precisely recruited during the trough of hippocampal theta activity. Our strategy of leveraging model databases that encompass experimental cell type-specificity and variability allowed us to reveal critical biophysical factors that contribute to neuronal function within in vivo-like contexts.

**Acknowledgements:** Supported by NSERC of Canada, an Ontario Graduate Scholarship, and the SciNet HPC Consortium.

**References**

1. Kispersky TJ, Fernandez FR, Economo MN, White JA. Spike resonance properties in hippocampal O-LM cells are dependent on refractory dynamics. J Neurosci. 2012;32(11):3637–51.
2. Sekulić V, Lawrence JJ, Skinner FK. Using multi-compartment ensemble modeling as an investigative tool of spatially distributed biophysical balances: application to hippocampal oriens-lacunosum/moleculare (O-LM) cells. PLOS One. 2014;9(10):e106567.
3. Borhegyi Z, Varga V, Szilágyi, Fabo D, Freund TF. Phase segregation of medial septal GABAergic neurons during hippocampal theta activity. J Neurosci. 2004;24(39):8470–9.
4. Varga C, Golshani P, Soltesz I. Frequency-invariant temporal ordering of interneuronal discharges during hippocampal oscillations in awake mice. Proc Natl Acad Sci USA. 2012;109(40):E2726–34.

## F2 Kernel methods in reconstruction of current sources from extracellular potentials for single cells and the whole brains

### Daniel K. Wójcik^1^, Chaitanya Chintaluri^1^, Dorottya Cserpán^2^, Zoltán Somogyvári^2^

#### ^1^Department of Neurophysiology, Nencki Institute of Experimental Biology, Warsaw, Poland; ^2^Department of Theory, Wigner Research Centre for Physics of the Hungarian Academy of Sciences, Budapest, H-1121, Hungary

##### **Correspondence:** Daniel K. Wójcik - d.wojcik@nencki.gov.pl

*BMC Neuroscience* 2016, **17(Suppl 1)**:F2

Extracellular recordings of electric potential, with a century old history, remain a popular tool for investigations of brain activity on all scales, from single neurons, through populations, to the whole brains, in animals and humans, in vitro and in vivo [1]. The specific information available in the recording depends on the physical settings of the system (brain + electrode). Smaller electrodes are usually more selective and are used to capture local information (spikes from single cells or LFP from populations) while larger electrodes are used for subdural recordings (on the cortex, ECoG), on the scalp (EEG) but also as depth electrodes in humans (called SEEG). The advantages of extracellular electric potential are the ease of recording and its stability. Its problem is interpretation: since electric field is long range one can observe neural activity several millimeters from its source [2–4]. As a consequence every recording reflects activity of many cells, populations and regions, depending on which level we focus. One way to overcome this problem is to reconstruct the distribution of current sources (CSD) underlying the measurement [5], typically done to identify activity on systems level from multiple LFP on regular grids [6].

We recently proposed a kernel-based method of CSD estimation from multiple LFP recordings from arbitrarily placed probes (i.e. not necessarily on a grid) which we called kernel Current Source Density method (kCSD) [7]. In this overview we present the original proposition as well as two recent developments, skCSD (single cell kCSD) and kESI (kernel Electrophysiological Source Imaging). skCSD assumes that we know which part of the recorded signal comes from a given cell and we have access to the morphology of the cell. This could be achieved by patching a cell, driving it externally while recording the potential on a multielectrode array, injecting a dye, and reconstructing the morphology. In this case we know that the sources must be located on the cell and this information can be successfully used in estimation. In kESI we consider simultaneous recordings with subdural ECoG (strip and grid electrodes) and with depth electrodes (SEEG). Such recordings are taken on some epileptic patients prepared for surgical removal of epileptogenic zone. When MR scan of the patient head is taken and the positions of the electrodes are known as well as the brain’s shape, the idea of kCSD can be used to bound the possible distribution of sources facilitating localization of the foci.

**Acknowledgements:** Polish Ministry for Science and Higher Education (grant 2948/7.PR/2013/2), Hungarian Scientific Research Fund (Grant OTKA K113147), National Science Centre, Poland (Grant 2015/17/B/ST7/04123).

**References**

1. Buzsáki G, Anastassiou CA, Koch C. The origin of extracellular fields and currents—EEG, ECoG, LFP and spikes. Nat Rev Neurosci. 2012;13:407–20.
2. Hunt MJ, Falinska M, Łęski S, Wójcik DK, Kasicki S. Differential effects produced by ketamine on oscillatory activity recorded in the rat hippocampus, dorsal striatum and nucleus accumbens. J Psychopharmacol. 2011;25:808–21.
3. Lindén H, Tetzlaff T, Potjans TC, Pettersen KH, Gruen S, Diesmann M, Einevoll GT. Modeling the spatial reach of the LFP. Neuron. 2011;72:859–72..
4. Łęski S, Lindén H, Tetzlaff T, Pettersen KH, Einevoll GT. Frequency dependence of signal power and spatial reach of the local field potential. PLoS Comput Biol. 2013;9:e1003137.
5. Wójcik DK. Current source density (CSD) analysis. In: Jaeger D, Jung R, editors. Encyclopedia of computational neuroscience. SpringerReference. Berlin: Springer; 2013.
6. Mitzdorf U. Current source-density method and application in cat cerebral cortex: investigation of evoked potentials and EEG phenomena. Physiol Rev. 1985;65:37–100.
7. Potworowski J, Jakuczun W, Łęski S, Wójcik DK. Kernel current source density method. Neural Comput. 2012;24:541–75.

## F3 The synchronized periods depend on intracellular transcriptional repression mechanisms in circadian clocks

### Jae Kyoung Kim^1^, Zachary P. Kilpatrick^2^, Matthew R. Bennett^3^, Kresimir Josić^2,4^

#### ^1^Department of Mathematical Sciences, KAIST, Daejoen 34141, Republic of Korea; ^2^Department of Mathematics, University of Houston, Houston, TX 77004, USA; ^3^Department of Biochemistry and Cell Biology and Institute of Biosciences and Bioengineering, Rice University, Houston, TX 77005, USA; ^4^Department of Biology and Biochemistry, University of Houston, Houston, TX 77004, USA

##### **Correspondence:** Jae Kyoung Kim - jaekkim@kaist.ac.kr

*BMC Neuroscience* 2016, **17(Suppl 1)**:F2

In mammals, circadian (~24 h) rhythms are mainly regulated by a master circadian clock located in the suprachiasmatic nucleus (SCN) [1]. The SCN consists of ~20,000 neurons, each of which generates own rhythms via intracellular transcriptional negative feedback loop involving PER-CRY and BMAL1-CLOCK. These individual rhythms of each neuron are synchronized through intercellular coupling via neurotransmitters including VIP [2]. In this talk, I will discuss that the synchronized periods via coupling signal strongly depend on the mechanism of intracellular transcription repression [3–4]. Specifically, using mathematical modeling and phase response curve analysis, we find that the synchronized period of SCN stays close to the population mean of cells’ intrinsic periods (~24 h) if transcriptional repression occurs via protein sequestration. However, the synchronized period is far from the population mean when repression occurs via Hill-type regulation (e.g. phosphorylation-based repression). These results reveal the novel relationship between two major functions of the SCN-*intracellular* rhythm generation and *intercellular* synchronization of rhythms. Furthermore, this relationship provides an explanation for why the protein sequestration is commonly used in circadian clocks of multicellular organisms, which have a coupled master clock, but not in unicellular organisms [4].

**Acknowledgements:** This work was funded by the National Institutes of Health, through the joint National Science Foundation/National Institute of General Medical Sciences Mathematical Biology Program grant No. R01GM104974 (to M.R.B. and K.J.), National Science Foundation grants Nos. DMS-1311755 (to Z.P.K.) and DMS-1122094 (to K.J.), the Robert A. Welch Foundation grant No. C-1729 (to M.R.B.), National Science Foundation grant No. DMS-0931642 to the Mathematical Biosciences Institute (to J.K.K.), KAIST Research Allowance Grant G04150020 (to J.K.K) and the TJ Park Science Fellowship of POSCO TJ Park Foundation G01160001 (to J.K.K).

**References**

1. Dibner C, Schibler U, Albrecht U. The mammalian circadian timing system: organization and coordination of central and peripheral clocks. Annu Rev Physiol. 2010;72:517–49.
2. Welsh DK, Takahashi JS, Kay SA. Suprachiasmatic nucleus: cell autonomy and network properties. Annu Rev Physiol. 2010;72:551.
3. Kim JK, Kilpatrick ZP, Bennett MR, Josić K. Molecular mechanisms that regulate the coupled period of the mammalian circadian clock. Biophys J. 2014;106(9):2071–81.
4. Kim JK. Protein sequestration vs Hill-type repression in circadian clock models (in revision).

## O1 Assessing irregularity and coordination of spiking-bursting rhythms in central pattern generators

### Irene Elices^1^, David Arroyo^1^, Rafael Levi^1,2^, Francisco B. Rodriguez^1^, Pablo Varona^1^

#### ^1^Grupo de Neurocomputación Biológica, Dpto. de Ingeniería Informática, Escuela Politécnica Superior, Universidad Autónoma de Madrid, Spain; ^2^Department of Biological Sciences, University of Southern California, CA, USA

##### **Correspondence:** Irene Elices - irene.elices@uam.es

*BMC Neuroscience* 2016, **17(Suppl 1)**:O1

Found in all nervous systems, central pattern generators (CPGs) are neural circuits that produce flexible rhythmic motor patterns. Their robust and highly coordinated spatio-temporal activity is generated in the absence of rhythmic input. Several invertebrate CPGs are among the best known neural circuits, as their neurons and connections have been identified and mapped. The crustacean pyloric CPG is one of these flagship neural networks [1, 2]. Experimental and computational studies of CPGs typically examine their rhythmic output in periodic spiking-bursting regimes. Aiming to understand the fast rhythm negotiation of CPG neurons, here we present experimental and theoretical analyses of the pyloric CPG activity in situations where irregular yet coordinated rhythms are produced. In particular, we focus our study in the context of two sources of rhythm irregularity: intrinsic damage in the preparation, and irregularity induced by ethanol. The analysis of non-periodic regimes can unveil important properties of the robust dynamics controlling rhythm coordination in this system.

Adult male and female shore crabs (*Carcinus maenas*) were used for the experimental recordings. The isolated stomatrogastric ganglion was kept in *Carcinus maenas* saline. Membrane potentials were recorded intracellularly from the LP and PD cells, two mutually inhibitory neurons that form a half-center oscillator in the pyloric CPG. Extracellular electrodes allowed monitoring the overall CPG rhythm. Conductance-based models of the pyloric CPG neurons and their associated graded synapses as described in [3, 4] were also used in this dual experimental and theoretical study.

Irregularity and coordination of the CPG rhythms were analyzed using measures characterizing the cells’ instantaneous waveform, period, duty cycle, plateau, hyperpolarization and temporal structure of the spiking activity, as well as measures describing instantaneous phases among neurons in the irregular rhythms and their variability. Our results illustrate the strong robustness of the circuit to keep LP/PD phase relationships in intrinsic and induced irregularity conditions while allowing a large variety of burst waveforms, durations and hyperpolarization periods in these neurons. In spite of being electrically coupled to the pacemaker cell of the circuit, the PD neurons showed a wide flexibility to participate with larger burst durations in the CPG rhythm (and larger increase in variability), while the LP neuron was more restricted in sustaining long bursts in the conditions analyzed. The conductance-based models were used to explain the role of asymmetry in the dynamics of the neurons and synapses to shape the irregular activity observed experimentally. Taking into account the overall experimental and model analyses, we discuss the presence of preserved relationships in the non-periodic but coordinated bursting activity of the pyloric CPG, and their role in the fast rhythm negotiating properties of this circuit.

**Acknowledgements:** We acknowledge support from MINECO DPI2015-65833-P, TIN2014-54580-R, TIN-2012-30883 and ONRG grant N62909-14-1-N279.

**References**

1. Marder E, Calabrese RL. Principles of rhythmic motor pattern generation. Physiol Rev. 1996;76:687–717.
2. Selverston AI, Rabinovich MI, Abarbanel HDI, Elson R, Szücs A, Pinto RD, Huerta R, Varona P. Reliable circuits from irregular neurons: a dynamical approach to understanding central pattern generators. J Physiol. 2000;94:357–74.
3. Latorre R, Rodríguez FB, Varona P. Neural signatures: multiple coding in spiking-bursting cells. Biol Cybern. 2006;95:169–83.
4. Elices I, Varona P. Closed-loop control of a minimal central pattern generator network. Neurocomputing. 2015;170:55–62.

## O2 Regulation of top-down processing by cortically-projecting parvalbumin positive neurons in basal forebrain

### Eunjin Hwang^1^, Bowon Kim^1,2^, Hio-Been Han^1,3^, Tae Kim^4^, James T. McKenna^5^, Ritchie E. Brown^5^, Robert W. McCarley^5^, Jee Hyun Choi^1,2^

#### ^1^Center for Neuroscience, Korea Institute of Science and Technology, Hwarang-ro 14-gil 5, Seongbuk-gu, Seoul 02792, South Korea; ^2^Department of Neuroscience, University of Science and Technology, 217 Gajeong-ro, Yuseong-gu, Daejon 34113, South Korea; ^3^Department of Psychology, Yonsei University, 50 Yonsei-ro, Seodaemun-gu, Seoul 03722, South Korea; ^4^Department of Psychiatry, Kyung Hee University Hospital at Gangdong, 892, Dongnam-ro, Gangdong-gu, Seoul 05278, South Korea; ^5^Department of Psychiatry, Veterans Administration Boston Healthcare System and Harvard Medical School, Brockton, MA 02301, USA

##### **Correspondence:** Jee Hyun Choi - jeechoi@kist.re.kr

*BMC Neuroscience* 2016, **17(Suppl 1)**:O2

Particular behaviors are associated with different spatio-temporal patterns of cortical EEG oscillations. A recent study suggests that the cortically-projecting, parvalbumin-positive (PV+) inhibitory neurons in the basal forebrain (BF) play an important role in the state-dependent control of cortical oscillations, especially ~40 Hz gamma oscillations [1]. However, the cortical topography of the gamma oscillations which are controlled by BF PV+ neurons and their relationship to behavior are unknown. Thus, in this study, we investigated the spatio-temporal patterns and the functional role of the cortical oscillations induced or entrained by BF PV+ neurons by combining optogenetic stimulation of BF PV+ neurons with high-density EEG [2, 3] in channelrhodopsin-2 (ChR2) transduced PV-cre mice. First, we recorded the spatio-temporal responses in the cortex with respect to the stimulation of BF PV+ neurons at various frequencies. The topographic response patterns were distinctively different depending on the stimulation frequencies, and most importantly, stimulation of BF PV+ neurons at 40 Hz (gamma band frequency) induced a preferential enhancement of gamma band oscillations in prefrontal cortex (PFC) with a statistically significant increase in intracortical connectivity within PFC. Second, optogenetic stimulation of BF PV+ neurons was applied while the mice were exposed to auditory stimuli (AS) at 40 Hz. The time delay between optogenetic stimulation and AS was tested and the phase response to the AS was characterized. We found that the phase responses to the click sound in PFC were modulated by the optogenetic stimulation of BF PV+ neurons. More specifically, the advanced activation of BF PV+ neurons by π/2 (6.25 ms) with respect to AS sharpened the phase response to AS in PFC, while the anti-phasic activation (π, 12.5 ms) blunted the phase response. Interestingly, like PFC, the primary auditory cortex (A1) also showed sharpened phase response for the π/2 advanced optogenetic BF PV+ neuron activation during AS. Considering that no direct influence of BF PV+ neurons on A1 was apparent in the response to stimulation of BF PV+ neurons alone, the sharpened phase response curve of A1 suggests a top-down influence of the PFC. This result implies that the BF PV+ neurons may participate in regulating the top-down influence that PFC exerts on primary sensory cortices during attentive behaviors, and supports the idea that the modulating activities of BF PV+ neurons might be a potential target for restoring top-down cognitive functions as well as abnormal frontal gamma oscillations associated with psychiatric disorders.

**Acknowledgements:** This research was supported by the Department of Veterans Affairs, the Korean National Research Council of Science & Technology (No. CRC-15-04-KIST), NIMH R01 MH039683 and Basic Science Research Program through the National Research Foundation of Korea (NRF) funded by the Ministry of Education (2015R1D1A1A01059119). The contents of this report do not represent the views of the US Department of Veterans Affairs or the United States government.

**References**

1. Kim T, et al. Cortically projecting basal forebrain parvalbumin neurons regulate cortical gamma band oscillations. Proc Natl Acad Sci. 2015;112(11):3535–40.
2. Choi JH, et al. High resolution electroencephalography in freely moving mice. J Neurophysiol .2010;104(3):1825–34.
3. Lee M, et al. High-density EEG recordings of the freely moving mice using polyimide-based microelectrode. J Vis Exp. 2011;47. http://www.jove.com/details.php?id=2562. doi:10.3791/2562.

## O3 Modeling auditory stream segregation, build-up and bistability

### James Rankin^1^, Pamela Osborn Popp^1^, John Rinzel^1,2^

#### ^1^Center for Neural Science, New York University, New York 10003, NY; ^2^Courant Institute of Mathematical Sciences, New York University, New York 10012, NY

##### **Correspondence:** James Rankin - james.rankin@nyu.edu

*BMC Neuroscience* 2016, **17(Suppl 1)**:O3

**Fig. 1:**
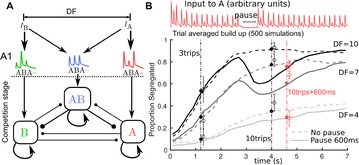
**A** Model schematic: tone inputs I_A_ and I_B_ elicit pulsatile responses in A1, which are pooled as inputs to a three-population competition network. Central unit AB encodes integrated, peripheral units A and B encode segregated. Mutual inhibition between units and recurrent excitation are incorporated with adaptation and noise. **B** A1 inputs show early initial adaptation, also if a pause is present. Build-up function shows proportion segregated increasing over time, here shown for three tone-frequency differences, DF, with no pause (*dashed*) or with a pause (*solid curves*). Time-snapshots from model (*filled circles*) agree with data (*empty circles* with SEM *error bars*, N = 8)

With neuromechanistic modelling and psychoacoustic experiments we study the perceptual dynamics of auditory streaming (cocktail party problem). The stimulus is a sequence of two interleaved tones, A and B in a repeating triplet pattern: ABA_ABA_ (‘_’ is a silent gap). Initially, subjects hear a single integrated pattern, but after some seconds they hear segregated A_A_A_ and _B___B__ streams (build-up of streaming segregation). For long presentations, build-up is followed by irregular alternations between integrated and segregated (auditory bistability). We recently presented [1] the first neuromechanistic model of auditory bistability; it incorporates common competition mechanisms of mutual inhibition, slow adaptation and noise [2]. Our competition network is formulated to reside downstream of primary auditory cortex (A1). Neural responses in macaque A1 to triplet sequences [3] encode stimulus features and provide the inputs to our network (Fig. 1A). In our model recurrent excitation with an NMDA-like timescale links responses across gaps between tones and between triplets. It captures the dynamics of perceptual alternations and the stimulus feature dependence of percept durations. To account for build-up we incorporate early adaptation of A1 responses [3] (Fig. 1B, upper). Early responses in A1 are broadly tuned and do not reflect the frequency difference between the tones; later responses show a clear tonotopic dependence. This adaptation biases the initial percept towards integration, but occurs faster (~0.5 s) than the gradual build-up process (~5–10 s). The low initial probability of segregation gradually builds up to the stable probability of later bistable alternations (Fig. 1B, lower). During build-up, a pause in presentation may cause partial reset to integrated [4]. Our extended model shows this behavior assuming that after a pause A1 responses recover on the timescale of early adaptation. Moreover, the modeling results agree with our psychoacoustic experiments (compare filled and open circles in Fig. 1B, lower).

**Conclusions** For the first time, we offer an explanation of the discrepancy in the timescales of early A1 responses and the more gradual build-up process. Recovery of A1 responses can explain resetting for stimulus pauses. Our model offers, to date, the most complete account of the early and late dynamics for auditory streaming in the triplet paradigm.

**References**

1. Rankin J, Sussman E, Rinzel J. Neuromechanistic model of auditory bistability. PLoS Comput Biol. 2015;11:e1004555.
2. Shpiro A, Moreno-Bote R, Rubin N, Rinzel J. Balance between noise and adaptation in competition models of perceptual bistability. J Comp Neurosci. 2009;27:37–54.
3. Micheyl C, Tian B, Carlyon R, Rauschecker J. Perceptual organization of tone sequences in the auditory cortex of awake macaques. Neuron. 2005;48:139–48.
4. Beauvois MW, Meddis R. Time decay of auditory stream biasing. Percept Psychophys. 1997;59:81–6.

## O4 Strong competition between tonotopic neural ensembles explains pitch-related dynamics of auditory cortex evoked fields

### Alejandro Tabas^1^, André Rupp^2,†^, Emili Balaguer-Ballester^1,3,†^

#### ^1^Faculty of Science and Technology, Bournemouth University, Bournemouth, England, UK; ^2^Heidelberg University, Baden-Württemberg, Germany; ^3^Bernstein Center for Computational Neuroscience, Heidelberg-Mannheim, Baden-Württemberg, Germany

##### **Correspondence:** Alejandro Tabas - atabas@bournemouth.ac.uk

^†^Equal contribution

*BMC Neuroscience* 2016, **17(Suppl 1)**:O4

Auditory evoked fields (AEFs) observed in MEG experiments systematically present a transient deflection known as the N100 m, elicited around 100 ms after the tone onset in the antero-lateral Heschl’s Gyrus. The exact N100m’s latency is correlated with the perceived pitch of a wide range of stimulus [1, 2], suggesting that the transient component reflects the processing of pitch in auditory cortex. However, the biophysical substrate of such precise relationship remains an enigma. Existing models of pitch, focused on perceptual phenomena, did not explain the mechanism generating cortical evoked fields during pitch processing in biophysical detail. In this work, we introduce a model of interacting neural ensembles describing, for the first time to our knowledge, how cortical pitch processing gives rise to observed human neuromagnetic responses and why its latency strongly correlates with pitch.

To provide a realistic cortical input, we used a recent model of the auditory periphery and realistic subcortical processing stages. Subcortical processing was based on a delay-and-multiply operation carried out in cochlear nucleus and inferior colliculus [3], resulting in realistic patterns of neural activation in response to the stimulus periodicities. Subcortical activation is transformed into a tonotopic receptive-field-like representation [4] by a novel cortical circuit composed by functional blocks characterised by a best frequency. Each block consist of an excitatory and an inhibitory population, modelled using mean-field approximations [5]. Blocks interact with each other through local AMPA- and NMDA-driven excitation and GABA-driven global inhibition [5].

**Fig. 2:**
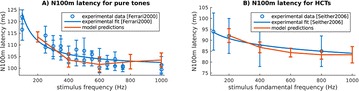
N100 m predictions in comparison with available data [1, 2] for a range of pure tones (**A**) and HCTs (**B**)

The excitation-inhibition competition of the cortical model describes a general pitch processing mechanism that explains the N100m deflection as a transient state in the cortical dynamics. The deflection is rapidly triggered by a rise in the activity elicited by the subcortical input, peaks after the inhibition overcomes the input, and stabilises when model dynamics reach equilibrium, around 100 ms after onset. As a direct consequence of the connectivity structure among blocks, the time necessary for the system to reach equilibrium depends on the encoded pitch of the tone. The model quantitatively predicts observed latencies of the N100m in agreement with available empirical data [1, 2] in a series of stimuli (see Fig. 2), suggesting that the mechanism potentially accounts for the N100 m dynamics.

**References**

1. Seither-Preisler A, Patterson R, Krumbholz K, Seither S, Lütkenhöner B. Evidence of pitch processing in the N100 m component of the auditory evoked field. Hear Res. 2006;213(1–2):88–98.
2. Roberts TP, Ferrari P, Stufflebeam SM, Poeppel D. Latency of the auditory evoked neuromagnetic field components: stimulus dependence and insights toward perception. J Clin Neurophysiol. 2000;17(2):114–29.
3. Meddis R, O’Mard LP. Virtual pitch in a computational physiological model. J Acoust Soc Am. 2006;6:3861–9.
4. Balaguer-Ballester E, Clark, N. Understanding pitch perception as a hierarchical process with top-down modulation. PLoS Comput Biol. 2009;5(3):e1000301.
5. Wong K-F, Wang X-J. A recurrent network mechanism of time integration in perceptual decisions. J Neurosci. 2006;26(4):1314–28.

## O5 A simple model of retinal response to multi-electrode stimulation

### Matias I. Maturana^1,2^, David B. Grayden^2,3^, Shaun L. Cloherty^4^, Tatiana Kameneva^2^, Michael R. Ibbotson^1,5^, Hamish Meffin^1,5^

#### ^1^National Vision Research Institute, Australian College of Optometry, 3053, Australia; ^2^NeuroEngineering Laboratory, Dept. Electrical & Electronic Eng., University of Melbourne, 3010, Australia; ^3^Centre for Neural Engineering, University of Melbourne, 3010, Australia; ^4^Department of Physiology, Monash University, 3800, Australia; ^5^ARC Centre of Excellence for Integrative Brain Function, Department Optometry and Vision Sciences, University of Melbourne, 3010, Australia

##### **Correspondence:** Hamish Meffin - hmeffin@unimelb.edu.au

*BMC Neuroscience* 2016, **17(Suppl 1)**:O5

Retinal implants can restore vision to patients suffering photoreceptor loss by stimulating surviving retinal ganglion cells (RGCs) via an array of microelectrodes implanted within the eye [1]. However, the acuity offered by existing devices is low, limiting the benefits to patients. Improvements may come by increasing the number of electrodes in new devices and providing patterned vision, which necessitates stimulation using multiple electrodes simultaneously. However, simultaneous stimulation poses a number of problems due to cross-talk between electrodes and uncertainty regarding the resulting activation pattern.

Here, we present a model and methods for estimating the responses of RGCs to simultaneous electrical stimulation. Whole cell in vitro patch clamp recordings were obtained from 25 RGCs with various morphological types in rat retina. The retinae were placed onto an array of 20 stimulating electrodes. Biphasic current pulses with 500 µs phase duration and 50 µs interphase gap were applied simultaneously to all electrodes at a frequency of 10 Hz, with the amplitude of current on each electrode sampled independently from a Gaussian distribution.

**Fig. 3:**
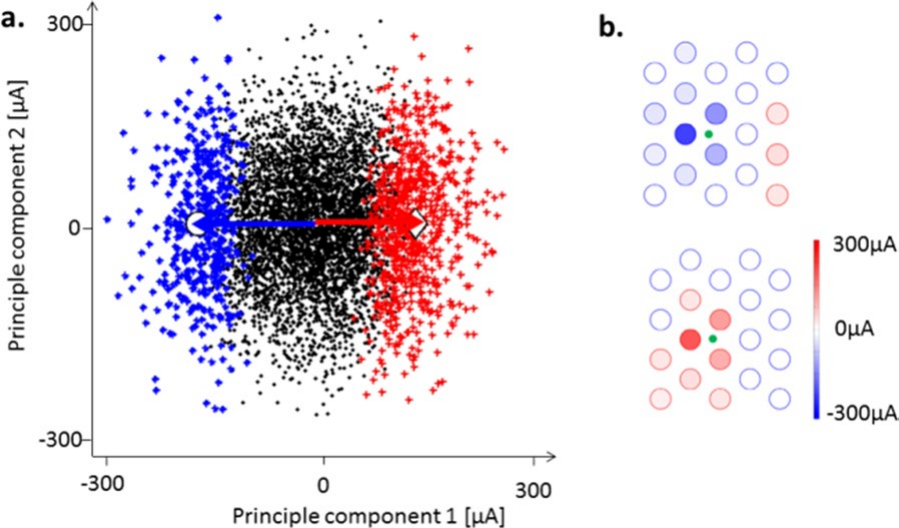
**a** Spike triggered covariance showing the full set of stimuli (*black dots*) projected onto the first two principle components. Stimuli causing a spike formed two clusters: net cathodic first pulses (*blue*) and net anodic first pulse (*red*). **b** Electrical receptive fields superimposed on the electrode array are shown for the cathodic first (*blue*) and anodic first clusters (*red*)

A linear-nonlinear model was fit to the responses of each RGC using spike-triggered covariance analyses on 80 % of the recorded data. The analysis revealed a single significant principle component corresponding to the electrical receptive field for each cell, with the second largest principle component having negligible effect on the neural response (Fig. 3a). This indicates that interactions between electrodes are approximately linear in their influence on the cells’ responses.

Furthermore, the spike-triggered ensemble showed two clusters (red and blue in Fig. 3a) corresponding to stimulation that had a net effect that was either anodic first or cathodic first. The electrical receptive fields for both anodic first and cathodic first stimulation were highly similar (Fig. 3b). They consisted of a small number (1–4) of electrodes that were close to the cell body (green dot).

The remaining 20 % of data were used to validate the model. The average model prediction root-mean-square error was 7 % over the 25 cells. The accuracy of the model indicates that the linear-nonlinear model is appropriate to describe the responses of RGCs to electrical stimulation.

**Acknowledgements:** This research was supported by the Australian Research Council (ARC). MI, HM, and SC acknowledge support through the Centre of Excellence for Integrative Brain Function (CE140100007), TK through ARC Discovery Early Career Researcher Award (DE120102210) and HM and TK through the ARC Discovery Projects funding scheme (DP140104533).

**Reference**

1. Hadjinicolaou AE, Meffin H, Maturana M, Cloherty SL, Ibbotson MR. Prosthetic vision: devices, patient outcomes and retinal research. Clin Exp Optom. 2015;98(5):395–410.

## O6 Noise correlations in V4 area correlate with behavioral performance in visual discrimination task

### Veronika Koren^1,2^, Timm Lochmann^1,2^, Valentin Dragoi^3^, Klaus Obermayer^1,2^

#### ^1^Institute of Software Engineering and Theoretical Computer Science, Technische Universitaet Berlin, Berlin, 10587, Germany; ^2^ Bernstein Center for Computational Neuroscience Berlin, Humboldt-Universitaet zu Berlin, Berlin, 10115, Germany; ^3^Department of Neurobiology and Anatomy, University of Texas-Houston Medical School, Houston, TX 77030, USA

##### **Correspondence:** Veronika Koren - veronika.koren@bccn-berlin.de

*BMC Neuroscience* 2016, **17(Suppl 1)**:O6

Linking sensory coding and behavior is a fundamental question in neuroscience. We have addressed this issue in behaving monkey visual cortex (areas V1 and V4) while animals were trained to perform a visual discrimination task in which two successive images were either rotated with respect to each other or were the same. We hypothesized that the animal’s performance in the visual discrimination task depends on the quality of stimulus coding in visual cortex. We tested this hypothesis by investigating the functional relevance of neuronal correlations in areas V1 and V4 in relation to behavioral performance. We measured two types of correlations: noise (spike count) correlations and correlations in spike timing. Surprisingly, both methods showed that correct responses are associated with significantly higher correlations in V4, but not V1, during the delay period between the two stimuli. This suggests that pair-wise interactions during the spontaneous activity preceding the arrival of the stimulus sets the stage for subsequent stimulus processing and importantly influences behavioral performance.

Experiments were conducted in 2 adult monkeys that were previously trained for the task. After 300 ms of fixation, the target stimulus, consisting of a naturalistic stimulus, is shown for 300 ms, and after a random delay period (500–1200 ms), a test stimulus is shown for 300 ms. The test can either be identical to the target stimulus (match) or rotated with respect to the target (non-match). Monkey responded by pressing a button and was rewarded for a correct response with fruit juice. Two linear arrays with 16 recording channels each were used to record population activity in areas V1 and V4. The difficulty of the task is calibrated individually to have 70 % correct responses on average. The analysis is conducted on non-match condition, comparing activity in trials with correct responses with trials where the monkey responded incorrectly. Noise correlations were assessed as pair-wise correlations of spike counts (method 1) and of spike timing (method 2). For method 1, z-scores of spike counts of binned spike trains are computed in individual trials. r_sc is computed as Pearson correlation coefficient of z-scores in all available trials, balanced across correct/incorrect condition. For the method 2, cross-correlograms were computed, from which the cross-correlograms from shuffled trials are subtracted. Resulting function was summed around zero lag and normalized with sum of autocorrelograms [1].

While firing rates of single units or of the population did not significantly change for correct and incorrect responses, noise correlations during the delay period were significantly higher in V4 pairs, computed with both r_sc method (p = 0.0005 in monkey 1, sign-rank test) and with r_ccg method (p = 0.0001 and p = 0.0280 in monkey 1 and 2, respectively, 50 ms integration window). This result is robust to changes in the length of the bin (method 1) and to the length of the summation window (method 2). In agreement with [2], we confirm the importance of spontaneous activity preceding the stimulus on performance and suggest that higher correlations in V4 might be beneficial for successful read-out and reliable transmission of the information downstream.

**References**

1. Bair W, Zohary E, Newsome WT. Correlated firing in macaque visual area MT: time scales and relationship to behavior. J Neurosci. 2001; 21(5):1676–97.
2. Gutnisky DA, Beaman CB, Lew SE, Dragoi V. Spontaneous fluctuations in visual cortical responses influence population coding accuracy. Cereb Cortex. 2016;1–19.
3. Cohen MR, Maunsell JH. Attention improves performance primarily by reducing interneuronal correlations. Nat Neurosci. 2009;12(12):1594–1600.
4. Nienborg HR, Cohen MR, Cumming BG. Decision-related activity in sensory neurons: correlations among neurons and with behavior. Annu Rev Neurosci. 2012;35:463–83.

## O7 Input-location dependent gain modulation in cerebellar nucleus neurons

### Maria Psarrou^1^, Maria Schilstra^1^, Neil Davey^1^, Benjamin Torben-Nielsen^1^, Volker Steuber^1^

#### Centre for Computer Science and Informatics Research, University of Hertfordshire, Hatfield, AL10 9AB, UK

##### **Correspondence:** Maria Psarrou - m.psarrou@herts.ac.uk

*BMC Neuroscience* 2016, **17(Suppl 1)**:O7

Gain modulation is a brain-wide principle of neuronal computation that describes how neurons integrate inputs from different presynaptic sources. A gain change is a multiplicative operation that is defined as a change in the sensitivity (or slope of the response amplitude) of a neuron to one set of inputs (driving input) which results from the activity of a second set of inputs (modulatory input) [1, 2].

Different cellular and network mechanisms have been proposed to underlie gain modulation [2–4]. It is well established that input features such as synaptic noise and plasticity can contribute to multiplicative gain changes [2–4]. However, the effect of neuronal morphology on gain modulation is relatively unexplored. Neuronal inputs to the soma and dendrites are integrated in a different manner: whilst dendritic saturation can introduce a strong non-linear relationship between dendritic excitation and somatic depolarization, the relationship between somatic excitation and depolarization is more linear. The non-linear integration of dendritic inputs can enhance the multiplicative effect of shunting inhibition in the presence of noise [3].

Neurons in the cerebellar nuclei (CN) provide the main gateway from the cerebellum to the rest of the brain. Understanding how inhibitory inputs from cerebellar Purkinje cells interact with excitatory inputs from mossy fibres to control output from the CN is at the center of understanding cerebellar computation. In the present study, we investigated the effect of inhibitory modulatory input on CN neuronal output when the excitatory driving input was delivered at different locations in the CN neuron. We used a morphologically realistic conductance based CN neuron model [5] and examined the change in output gain in the presence of distributed inhibitory input under two conditions: (a) when the excitatory input was confined to one compartment (the soma or a dendritic compartment) and, (b), when the excitatory input was distributed across particular dendritic regions at different distances from the soma. For both of these conditions, our results show that the arithmetic operation performed by inhibitory synaptic input depends on the location of the excitatory synaptic input. In the presence of distal dendritic excitatory inputs, the inhibitory input has a multiplicative effect on the CN neuronal output. In contrast, excitatory inputs at the soma or proximal dendrites close to the soma undergo additive operations in the presence of inhibitory input. Moreover, the amount of the multiplicative gain change correlates with the distance of the excitatory inputs from the soma, with increasing distances from the soma resulting in increased gain changes and decreased additive shifts along the input axis. These results indicate that the location of synaptic inputs affects in a systematic way whether the input undergoes a multiplicative or additive operation.

**References**

1. Salinas E, Sejnowski TJ. Gain modulation in the central nervous system: where behavior, neurophysiology, and computation meet. Neuroscientist. 2001;7(5):430–40.
2. Silver RA. Neuronal arithmetic. Nat Rev Neurosci. 2010;11(7):474–89.
3. Prescott SA, De Koninck Y. Gain control of firing rate by shunting inhibition: roles of synaptic noise and dendritic saturation. Proc Natl Acad Sci USA. 2003;100(4):2076–81.
4. Rothman J, Cathala L, Steuber V, Silver RA. Synaptic depression enables neuronal gain control. Nature. 2009;475:1015–18.
5. Steuber V, Schultheiss NW, Silver RA, De Schutter E, Jaeger D. Determinants of synaptic integration and heterogeneity in rebound firing explored with data-driven models of deep cerebellar nucleus cells. J Comput Neurosci. 2011;30(3):633–58.

## O8 Analytic solution of cable energy function for cortical axons and dendrites

### Huiwen Ju^1^, Jiao Yu^2^, Michael L. Hines^3^, Liang Chen^4^ and Yuguo Yu^1^

#### ^1^School of Life Science and the Collaborative Innovation Center for Brain Science, Fudan University, Shanghai, 200438, China; ^2^Linyi Hospital of Traditional Chinese Medicine, 211 Jiefang Road, Lanshan, Linyi, Shandong Province, 276000, China; ^3^Department of Neuroscience, Yale University School of Medicine, New Haven, CT 06520, USA; ^4^Department of Neurosurgery, Huashan Hospital, Shanghai Medical College, Fudan University, Shanghai, China

##### **Correspondence:** Yuguo Yu - yuyuguo@fudan.edu.cn

*BMC Neuroscience* 2016, **17(Suppl 1)**:O8

Accurate estimation of action potential (AP)-related metabolic cost is essential for understanding energetic constraints on brain connections and signaling processes. Most previous energy estimates of the AP were obtained using the Na^+^-counting method [1, 2], which seriously limits accurate assessment of metabolic cost of ionic currents that underlie AP generation. Moreover, the effects of axonal geometry and ion channel distribution on energy consumption related to AP propagation have not been systematically investigated.

To address these issues, we return to the cable theory [3] that underlies our HH-type cortical axon model [4], which was constructed based on experimental measurements. Based on the cable equation that describes how ion currents flow along the cable as well as analysis of the electrochemical energy in the equivalent circuit, we derived the electrochemical energy function for the cable model,

$$ \begin{aligned} \frac{{\partial^{2} E}}{\partial x\partial t} & = I_{Na} \left( {V - V_{Na} } \right) + I_{K} \left( {V - V_{K} } \right) + I_{L} \left( {V - V_{L} } \right) - \frac{1}{2\pi a}i_{a} \frac{\partial V}{\partial x} \\ & = g_{Na}^{\hbox{max} } m^{3} h\left( {V\left( {x,t} \right) - V_{Na} } \right)^{2} + g_{K}^{\hbox{max} } n^{4} \left( {V\left( {x,t} \right) - V_{K} } \right)^{2} \\ & \quad + g_{L} \left( {V\left( {x,t} \right) - V_{L} } \right)^{2} + G_{a} \left( {\frac{\partial V}{\partial x}} \right)^{2} \\ \end{aligned} $$

where g_Na_^max^ (in a range of 50–650 mS/cm^2^), g_K_^max^ (5–100 mS/cm^2^), and g_L_ = 0.033 mS/cm^2^ are the maximal sodium, maximal potassium, and leak conductance per unit membrane area, respectively; and V_Na_ = 60, V_K_ = −90 V_L_ = −70 mV are the reversal potentials of the sodium, potassium, and leak channels, respectively. The gate variables m, h, and n are dimensionless activation and inactivation variables, which describe the activation and inactivation processes of the sodium and potassium channels [4]. This equation describes the AP-related energy consumption rate per unit membrane area (cm^2^/s) at any axonal distance and any time. The individual terms on the right-hand side of the equation represent the contributions of the sodium, potassium, leak, and axial currents, respectively. Then we employed the cable energy function to calculate energy consumption for unbranched axons and axons with several degrees of branching (branching level, BL). Calculations based on this function distinguish between the contributions of each item toward total energy consumption.

Our analytical approach predicts an inhomogeneous distribution of metabolic cost along an axon with either uniformly or nonuniformly distributed ion channels. The results show that the Na+-counting method severely underestimates energy cost in the cable model by 20–70 %. AP propagation along axons that differ in length may require over 15 % more energy per unit of axon area than that required by a point model. However, actual energy cost can vary greatly depending on axonal branching complexity, ion channel density distributions, and AP conduction states. We also infer that the metabolic rate (i.e. energy consumption rate) of cortical axonal branches as a function of spatial volume exhibits a 3/4 power law relationship.

**Acknowledgements:** Dr. Yu thanks for the support from the National Natural Science Foundation of China (31271170, 31571070), Shanghai program of Professor of Special Appointment (Eastern Scholar SHH1140004).

**References**

1. Alle H, Roth A, Geiger JR. Energy-efficient action potentials in hippocampal mossy fibers. Science. 2009;325(5946):1405–8.
2. Carter BC, Bean BP. Sodium entry during action potentials of mammalian neurons: incomplete inactivation and reduced metabolic efficiency in fast-spiking neurons. Neuron. 2009;64(6):898–909.
3. Rall W. Cable theory for dendritic neurons. In: Methods in neuronal modeling. MIT Press; 1989. p. 9–92.
4. Yu Y, Hill AP, McCormick DA. Warm body temperature facilitates energy efficient cortical action potentials. PLoS Comput Biol. 2012;8(4):e1002456.

## O9 *C. elegans* interactome: interactive visualization of *Caenorhabditis elegans* worm neuronal network

### Jimin Kim^1^, Will Leahy^2^, Eli Shlizerman^1,3^

#### ^1^Department of Applied Mathematics, University of Washington, Seattle, WA 98195, USA; ^2^Amazon.com Inc., Seattle, WA 98108, USA; ^3^Department of Electrical Engineering, University of Washington, Seattle, WA 98195, USA

##### **Correspondence:** Eli Shlizerman - shlizee@uw.edu

*BMC Neuroscience* 2016, **17(Suppl 1)**:O9

Modeling neuronal systems involves incorporating the two layers: a static map of neural connections (connectome), and biophysical processes that describe neural responses and interactions. Such a model is called the ‘dynome’ of a neuronal system as it integrates a dynamical system with the static connectome. Being closer to reproducing the activity of a neuronal system, investigation of the dynome has more potential to reveal neuronal pathways of the network than the static connectome [1]. However, since the two layers of the dynome are considered simultaneously, novel tools have to be developed for the dynome studies. Here we present a visualization methodology, called `interactome’, that allows to explore the dynome of a neuronal system interactively and in real-time, by viewing the dynamics overlaid on a graph representation of the connectome.

**Fig. 4:**
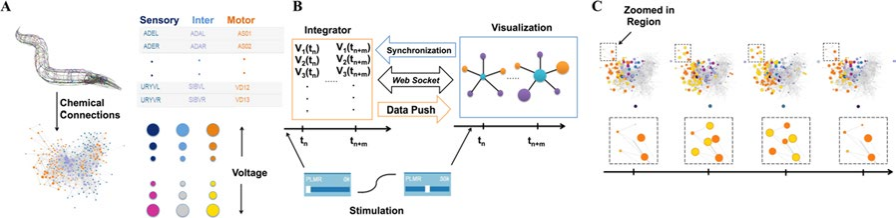
**A** Visualization of *C. elegans* dynome, **B** communication diagram between the dynome and the layout, **C** snapshots of visualization of *C. elegans* during the PLM/AVB excitations (forward crawling)

We apply our methodology to the nervous system of *Caenorhabditis elegans* (*C. elegans*) worm, which connectome is almost fully resolved [2], and a computational model of neural dynamics and interactions (gap and synaptic) based on biophysical experimental findings was recently introduced [3]. Integrated together, *C. elegans* dynome defines a unique set of neural dynamics of the worm. To visualize the dynome, we propose a dynamic force-directed graph layout of the connectome. The layout is implemented using D3 visualization platform [4], and is designed to communicate with an integrator of the dynome. The two-way communication protocol between the layout and the integrator allows for stimulating (injecting current) into any subset of neurons at any time point (Fig. 4B). It also allows for simultaneously viewing the response of the network on top of the layout visualized by resizing graph nodes (neurons) according to their voltage. In addition, we support structural changes in the connectome, such as ablation of neurons and connections.

Our visualization and communication protocols thereby display the stimulated network in an interactive manner and permit to explore different regimes that the stimulations induce. Indeed, with the interactome we are able to recreate various experimental scenarios, such as stimulation of forward crawling (PLM/AVB neurons and/or ablation of AVB) and show that its visualization assists in identifying patterns of neurons in the stimulated network. As connectomes and dynomes of additional neuronal systems are being resolved, the interactome will enable exploring their functionality and inference to its underlying neural pathways [5].

**References**

1. Kopell NJ, Gritton HJ, Whittingon MA, Kramer MA. Beyond the connectome: the dynome. Neuron. 2014;83(6):1319–28.
2. Varshney LR, Chen BL, Paniagua E, Hall DH, Chkolvski DB. Structural properties of the caenorhabditis elegans neuronal network. PLoS Comput Biol. 2011;7(2):e1001066.
3. Kunert J, Shlizerman E, Kutz JN. Low-dimensional functionality of complex network dynamics: neurosensory integration in the *Caenorhabditis elegans* connectome. Phys Rev E. 2014;89(5):052805.
4. Bostock M, Ogievetsky V, Heer J. D3 data-driven documents. IEEE. 2011;17(12):2301–9.
5. Kim J, Leahy W, Shlizerman E. *C. elegans* interactome: interactive visualization of *Caenorhabditis elegans* worm neuronal network. 2016 (in submission).

## O10 Is the model any good? Objective criteria for computational neuroscience model selection

### Justas Birgiolas^1^, Richard C. Gerkin^1^, Sharon M. Crook^1,2^

#### ^1^School of Life Science, Arizona State University, Tempe, AZ 85287, USA; ^2^School of Mathematical and Statistical Sciences, Arizona State University, Tempe, AZ, 85287, USA

##### **Correspondence:** Justas Birgiolas - justas@asu.edu

*BMC Neuroscience* 2016, **17(Suppl 1)**:O10

Objectively evaluating and selecting computational models of biological neurons is an ongoing challenge in the field. Models vary in morphological detail, channel mechanisms, and synaptic transmission implementations. We present the results of an automated method for evaluating computational models against property values obtained from published cell electrophysiology studies. Seven published deterministic models of olfactory bulb mitral cells were selected from ModelDB [1] and simulated using NEURON’s Python interface [2]. Passive and spike properties in response to step current stimulation pulses were computed using the NeuronUnit [3] package and compared to their respective, experimentally obtained means of olfactory bulb mitral cell properties found in the NeuroElectro database [4].

**Fig. 5:**
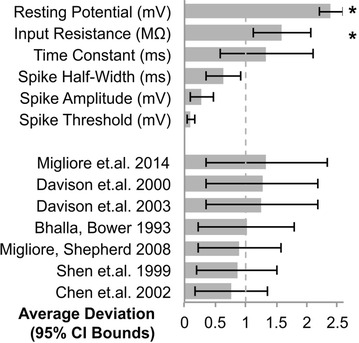
The average deviations of models and cell electrophysiology properties as measured in multiples of the 95 % CI bounds of experimental data means. *Dashed line* represents 1 CI bound threshold. *Top rows* show average deviations across all models for each cell property. *Bottom rows* show deviations across all cell properties for each model

Results reveal that across all models, the resting potential and input resistance property means deviated the most from their experimentally measured means (R_input_*ttest* p = 0.02, V_rest_*Wilcoxon*-*test* p = 0.01). The time constant, spike half-width, spike amplitude, and spike threshold properties, in the order of decreasing average deviation, matched well with experimental data (p > 0.05) (Fig. 5 top).

In three models, the property deviations were, on average, outside the 95 % CI of the experimental means (Fig. 5 bottom), but these averages were not significant (*t test* p > 0.05). All other models were within the 95 % CI, while the model of Chen et al. had the lowest deviation [5].

Overall, the majority of these olfactory bulb mitral cell models display some properties that are not significantly different from their experimental means. However, the resting potential and input resistance properties significantly differ from the experimental values. We demonstrate that NeuronUnit provides an objective method for evaluating the fitness of computational neuroscience cell models against publicly available data.

**Acknowledgements:** The work of JB, RG, and SMC was supported in part by R01MH1006674 from the National Institutes of Health.

**References**

1. Hines ML, Morse T, Migliore M, Carnevale NT, Shepherd GM. ModelDB: a database to support computational neuroscience. J Comput Neurosci. 2004;17(1):7–11.
2. Hines M, Davison AP, Muller E. NEURON and Python. Front Neuroinform. 2009;3:1.
3. Omar C, Aldrich J, Gerkin RC. Collaborative infrastructure for test-driven scientific model validation. In: Companion proceedings of the 36th international conference on software engineering. ACM; 2014. p. 524–7.
4. Tripathy SJ, Savitskaya J, Burton SD, Urban NN, Gerkin RC. NeuroElectro: a window to the world’s neuron electrophysiology data. Front Neuroinform. 2014;8.
5. Chen WR, Shen GY, Shepherd GM, Hines ML, Midtgaard J. Multiple modes of action potential initiation and propagation in mitral cell primary dendrite. J Neurophysiol. 2002;88(5):2755–64.

## O11 Cooperation and competition of gamma oscillation mechanisms

### Atthaphon Viriyopase^1,2,3^, Raoul-Martin Memmesheimer^1,3,4^, and Stan Gielen^1,2^

#### ^1^Donders Institute for Brain, Cognition and Behaviour, Radboud University Nijmegen (Medical Centre), The Netherlands; ^2^Department for Biophysics, Faculty of Science, Radboud University Nijmegen, The Netherlands; ^3^Department for Neuroinformatics, Faculty of Science, Radboud University Nijmegen, The Netherlands; ^4^Center for Theoretical Neuroscience, Columbia University, New York, NY, USA

##### **Correspondence:** Atthaphon Viriyopase - a.viriyopase@science.ru.nl

*BMC Neuroscience* 2016, **17(Suppl 1)**:O11

Two major mechanisms that underlie gamma oscillations are InterNeuronal Gamma (“ING”), which is related to tonic excitation of reciprocally coupled inhibitory interneurons (I-cells), and Pyramidal InternNeuron Gamma (“PING”), which is mediated by coupled populations of excitatory pyramidal cells (E-cells) and I-cells. ING and PING are thought to serve different biological functions. Using computer simulations and analytical methods, we [1] therefore investigate which mechanism (ING or PING) will dominate the dynamics of a network when ING and PING interact and how the dominant mechanism may switch.

**Fig. 6:**
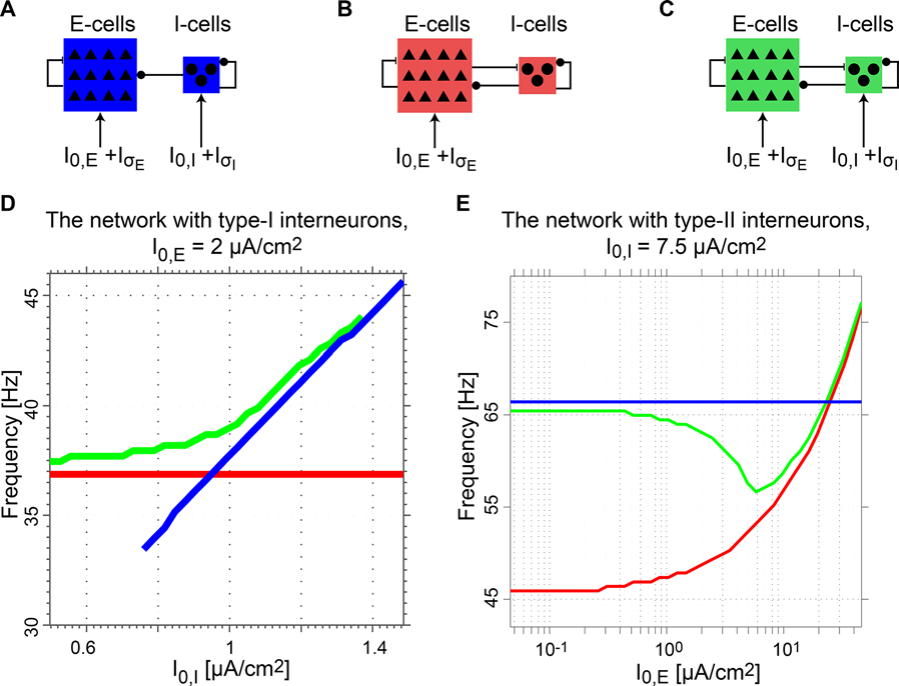
Oscillations in full and reduced networks of reciprocally coupled pyramidal cells and interneurons. **A**, **B** Illustrate topologies of reduced networks that generate “pure” ING and “pure” PING, respectively, while **C** highlights the topology of a “full” network that could in principle generate either ING or PING oscillations or mixtures of both. **D**, **E** Frequency of pure ING-rhythm generated by the reduced network in **A** (*blue line*), pure PING-rhythm generated by the reduced network in **b** (*red line*), and rhythms generated by the full network in **C** (*green line*) as a function of mean current to I-cells I_0,I_ and as function of mean current to E-cells I_0,E_, respectively. **D** Results for networks with type-I interneurons while **E** shows results for networks with type-II interneurons. Pyramidal cells are modeled as type-I Hodgkin–Huxley neurons

We find that ING and PING oscillations compete: The mechanism generating the higher oscillation frequency “wins”. It determines the frequency of the network oscillations and suppresses the other mechanism. The network oscillation frequency (green lines corresponding to the network topology given in Fig. 6C) corresponding to the network with type-I-phase-response-curve interneurons and type-II-phase-response-curve interneurons is plotted in Fig. 6D, E, respectively. We explain our simulation results by a theoretical model that allows a full theoretical analysis.

Our study suggests experimental approaches to decide whether oscillatory activity in networks of interacting excitatory and inhibitory neurons is dominated by ING or PING oscillations and whether the participating interneurons belong to class I or II. Consider as an example networks with type-I interneurons where the external drive to the E-cells, I_0,E_, is kept constant while the external drive to the I-cells, I_0,I_, is varied. For both ING and PING dominated oscillations the frequency of the rhythm increases when I_0,I_ increases (cf. Fig. 6D). Observing such an increase does therefore *not* allow to determine the underlying mechanism. However, the absolute value of the first derivative of the frequency with respect to I_0,I_ allows a distinction, as it is much smaller for PING than for ING (cf. Fig. 6D). In networks with type-II interneurons, the non-monotonic dependence near the ING-PING transition may be a characteristic hallmark to detect the oscillation character (and the interneuron type): Decrease (increase) of the frequency when increasing I_0,E_ indicates ING (PING), cf. Fig. 6E. These theoretical predictions are in line with experimental evidence [2].

**References**

1. Viriyopase A, Memmesheimer RM, Gielen S. Cooperation and competition of gamma oscillation mechanisms. J Neurophysiol. 2016.
2. Craig MT, McBain CJ. Fast gamma oscillations are generated intrinsically in CA1 without the involvement of fast-spiking basket cells. J Neurosci. 2015;35(8):3616–24.

## O12 A discrete structure of the brain waves

### Yuri Dabaghian^1,2^, Justin DeVito^1^, Luca Perotti^3^

#### ^1^Department of Neurology Pediatrics, Baylor College of Medicine, Houston, TX 77030, USA; ^2^Department of Computational and Applied Mathematics, Rice University, Houston, TX, 77005, USA; ^3^Physics Department, Texas Southern University, 3100 Cleburne St, Houston, TX 77004, USA

##### **Correspondence:** Yuri Dabaghian - dabaghian@rice.edu

*BMC Neuroscience* 2016, **17(Suppl 1)**:O12

A physiological interpretation of the biological rhythms, e.g., of the local field potentials (LFP) depends on the mathematical and computational approaches used for its analysis. Most existing mathematical methods of the LFP studies are based on braking the signal into a combination of simpler components, e.g., into sinusoidal harmonics of Fourier analysis or into wavelets of the Wavelet Analysis. However, a common feature of all these methods is that their prime components are presumed from the onset, and the goal of the subsequent analysis reduces to identifying the combination that best reproduces the original signal.

We propose a fundamentally new method, based on a number of deep theorems of complex function theory, in which the prime components of the signal are not presumed a priori, but discovered empirically [1]. Moreover, the new method is more flexible and more sensitive to the signal’s structure than the standard Fourier method.

Applying this method reveals a fundamentally new structure in the hippocampal LFP signals in rats in mice. In particular, our results suggest that the LFP oscillations consist of a superposition of a small, discrete set of frequency modulated oscillatory processes, which we call “oscillons”. Since these structures are discovered empirically, we hypothesize that they may capture the signal’s actual physical structure, i.e., the pattern of synchronous activity in neuronal ensembles. Proving this hypothesis will help enormously to advance a principal, theoretical understanding of the neuronal synchronization mechanisms. We anticipate that it will reveal new information about the structure of the LFP and other biological oscillations, which should provide insights into the underlying physiological phenomena and the organization of brains states that are currently poorly understood, e.g., sleep and epilepsy.

**Acknowledgements:** The work was supported by the NSF 1422438 grant and by the Houston Bioinformatics Endowment Fund.

**Reference**

1. Perotti L, DeVito J, Bessis D, Dabaghian Y, Dabaghian Y, Brandt VL, Frank LM. Discrete spectra of brain rhythms (in submisison).

## O13 Direction-specific silencing of the *Drosophila* gaze stabilization system

### Anmo J. Kim^1,†^, Lisa M. Fenk^1,†^, Cheng Lyu^1^, Gaby Maimon^1^

#### ^1^Laboratory of Integrative Brain Function, The Rockefeller University, New York, NY 10065, USA

##### **Correspondence:** Anmo J. Kim - anmo.kim@gmail.com

^†^ Authors contributed equally

*BMC Neuroscience* 2016, **17(Suppl 1)**:O13

Many animals, including insects and humans, stabilize the visual image projected onto their retina by following a rotating landscape with their head or eyes. This stabilization reflex, also called the optomotor response, can pose a problem, however, when the animal intends to change its gaze. To resolve this paradox, von Holst and Mittelstaedt proposed that a copy of the motor command, or efference copy, could be routed into the visual system to transiently silence this stabilization reflex when an animal changes its gaze [1]. Consistent with this idea, we recently demonstrated that a single identified neuron associated with the optomotor response receives silencing motor-related inputs during rapid flight turns, or saccades, in tethered, flying *Drosophila* [2].

**Fig. 7:**
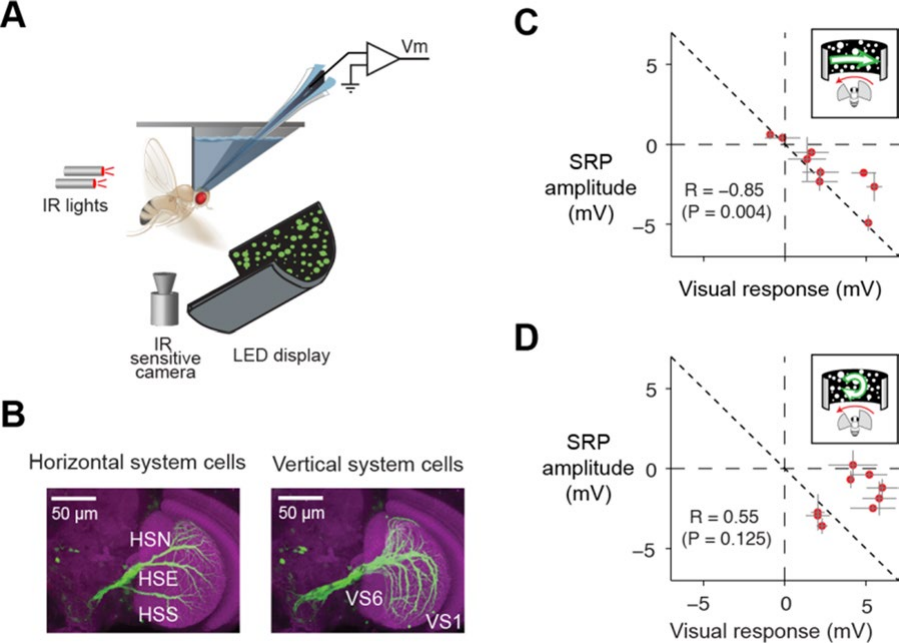
The amplitudes of saccade-related potentials (SRPs) to HS and VS cells are strongly correlated with each cell’s visual sensitivity to rightward yaw motion stimuli. **A** Experimental apparatus. **B** Maximal-intensity z-projections of the lobula plate to visualize HS- or VS-cell neurites that are marked by a GAL4 enhancer trap line. **C**, **D** The amplitude of saccade-related potentials (SRPs) were inversely correlated with visual responses, when measured under rightward yaw motion stimuli, but not under clockwise roll motion stimuli. Each sample point corresponds to each cell type. *Error bars* indicate SEM

Here, we expand on these results by comprehensively recording from a group of optomotor-mediating visual neurons in the fly visual system: three horizontal system (HS) and six vertical system (VS) cells. We found that the amplitude of motor-related inputs to each HS and VS cell correlates strongly with the strength of each cell’s visual sensitivity to rotational motion stimuli around the primary turn axis, but not to the other axes (Fig. 7). These results support the idea that flies send rotation-axis-specific efference copies to the visual system during saccades—silencing the stabilization reflex only for a specific axis, but leaving the others intact. This is important because saccades consist of stereotyped banked turns, which involve body rotations around all three primary axes of rotation. If the gaze stabilization system is impaired for only one of these axes, then the fly is expected to attempt to maintain gaze stability, through a combination of head and body movements, for the other two. This prediction is consistent with behavioral measurements of head and body kinematics during saccades in freely flying blow flies [3]. Together, these studies provide an integrative model of how efference copies counteract a specific aspect of visual feedback signals to tightly control the gaze stabilization system.

**References**

1. von Holst E, Mittelstaedt H. The principle of reafference. Naturwissenschaften.1950;37:464–76.
2. Kim AJ, Fitzgerald JK, Maimon G. Cellular evidence for efference copy in *Drosophila* visuomotor processing. Nat Neurosci. 2015;18:1247–55.
3. Schilstra C, van Hateren JH. Stabilizing gaze in flying blowflies. Nature. 1998;395:654.

## O14 What does the fruit fly think about values? A model of olfactory associative learning

### Chang Zhao^1^, Yves Widmer^2^, Simon Sprecher^2^, Walter Senn^1^

#### ^1^Department of Physiology, University of Bern, Bern, 3012, Switzerland; ^2^Department of Biology, University of Fribourg, Fribourg, 1700, Switzerland

##### **Correspondence:** Chang Zhao - zhao@pyl.unibe.ch

*BMC Neuroscience* 2016, **17(Suppl 1)**:O14

Associative learning in the fruit fly olfactory system has been studied from the molecular to the behavior level [1, 2]. Fruit flies are able to associate conditional stimuli such as odor with unconditional aversive stimuli such as electrical shocks, or appetitive stimuli such as sugar or water. The mushroom body in the fruit fly brain is considered to be crucial for olfactory learning [1, 2]. The behavioral experiments show that the learning can not be explained simply by an additive Hebbian (i.e. correlation-based) learning rule. Instead, it depends on the timing between the conditional and unconditional stimulus presentation. Yarali and colleagues suggested a dynamic model on the molecular level to explain event timing in associative learning [3]. Here, we present new experiments together with a simple phenomenological model for learning that shows that associative olfactory learning in the fruit fly represents value learning that is incompatible with Hebbian learning.

In our model, the information of the conditional odor stimulus is conveyed by Kenyon cells from the projection neurons to the mushroom output neurons; the information of the unconditional shock stimulus is represented by dopaminergic neurons to the mushroom output neurons through direct or indirect pathways. The mushroom body output neurons encode the internal value (v) of the odor (o) by synaptic weights (w) that conveys the odor information, v = w∙o. The synaptic strength is updated according to the value learning rule, Δw = *η*(s − v)õ, where s represents the (internal) strength of the shock stimulus, õ represents the synaptic odor trace, and *η* is the learning rate. The value associated with the odor determines the probability of escaping from that odor. This simple model reproduces the behavioral data and shows that olfactory conditioning in the fruit fly is in fact value learning. In contrast to the prediction of Hebbian learning, the escape probability for repeated odor-shock pairings is much lower than the escape probability for a single pairing with a correspondingly stronger shock.

**References**

1. Aso Y, Sitaraman D, Ichinose T, Kaun KR, Vogt K, Belliart-Gurin G, Plaais PY, Robie AA, Yamagata N, Schnaitmann C, Rowell WJ, Johnston RM, Ngo TB, Chen N, Korff W, Nitabach MN, Heberlein U, Preat T, Branson KM, Tanimoto H, Rubin GM: Mushroom body output neurons encode valence and guide memory-based action selection in Drosophila. ELife. 2014;3:e04580.
2. Heisenberg M. Mushroom body memoir: from maps to models. Nat Rev Neurosci. 2003;4:266–75.
3. Yarali A, Nehrkorn J, Tanimoto H, Herz AVM. Event timing in associative learning: from biochemical reaction dynamics to behavioural observations. PLoS One. 2012;7(3):e32885.

## O15 Effects of ionic diffusion on power spectra of local field potentials (LFP)

### Geir Halnes^1^, Tuomo Mäki-Marttunen^2^, Daniel Keller^3^, Klas H. Pettersen^4,5^,Ole A. Andreassen^2^, Gaute T. Einevoll^1,6^

#### ^1^Department of Mathematical Sciences and Technology, Norwegian University of Life Sciences, Ås, Norway; ^2^NORMENT, Institute of Clinical Medicine, University of Oslo, Oslo, Norway; ^3^The Blue Brain Project, École Polytechnique Fédérale de Lausanne (EPFL), Lausanne, Switzerland; ^4^Letten Centre and Glialab, Department of Molecular Medicine, Instotute of Basic Medical Sciences, University of Oslo, Oslo, Norway; ^5^Centre for Molecular Medicine Norway, University of Oslo, Oslo, Norway; ^6^Department of Physics, University of Oslo, Oslo, Norway

##### **Correspondence:** Geir Halnes - geir.halnes@nmbu.no

*BMC Neuroscience* 2016, **17(Suppl 1)**:O15

The local field potential (LFP) in the extracellular space (ECS) of the brain, is a standard measure of population activity in neural tissue. Computational models that simulate the relationship between the LFP and its underlying neurophysiological processes are commonly used in the interpretation such measurements. Standard methods, such as volume conductor theory [1], assume that ionic diffusion in the ECS has negligible impact on the LFP. This assumption could be challenged during endured periods of intense neural signalling, under which local ion concentrations in the ECS can change by several millimolars. Such concentration changes are indeed often accompanied by shifts in the ECS potential, which may be partially evoked by diffusive currents [2]. However, it is hitherto unclear whether putative diffusion-generated potential shifts are too slow to be picked up in LFP recordings, which typically use electrode systems with cut-off frequencies at ~0.1 Hz.

To explore possible effects of diffusion on the LFP, we developed a hybrid simulation framework: (1) The NEURON simulator was used to compute the ionic output currents from a small population of cortical layer-5 pyramidal neurons [3]. The neural model was tuned so that simulations over ~100 s of biological time led to shifts in ECS concentrations by a few millimolars, similar to what has been seen in experiments [2]. (2) In parallel, a novel electrodiffusive simulation framework [4] was used to compute the resulting dynamics of the potential and ion concentrations in the ECS, accounting for the effect of electrical migration as well as diffusion. To explore the relative role of diffusion, we compared simulations where ECS diffusion was absent with simulations where ECS diffusion was included.

**Fig. 8:**
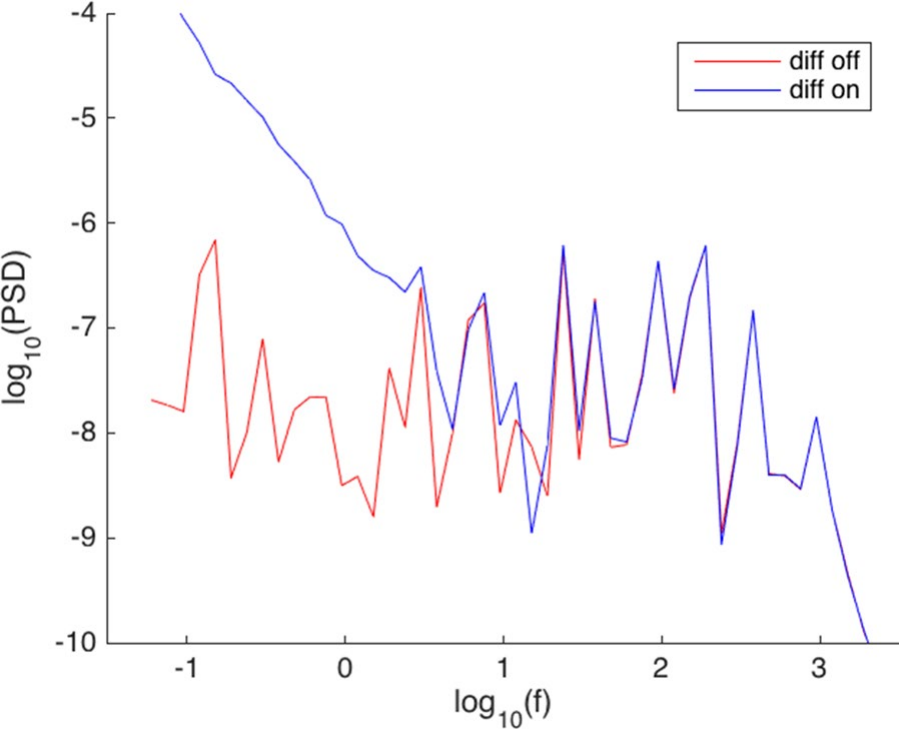
Power spectrum of ECS potential in a simulation including ECS diffusion (*blue line*) and a simulation without ECS diffusion (*red line*). Units for frequency and power are Hz and mV^2^/Hz, respectively

Our key findings were: (i) ECS diffusion shifted the local potential by up to ~0.2 mV. (ii) The power spectral density (PSD) of the diffusion-evoked potential shifts followed a 1*/f*^*2*^ power law. (iii) Diffusion effects dominated the PSD of the ECS potential for frequencies up to ~10 Hz (Fig. 8). We conclude that for large, but physiologically realistic ECS concentration gradients, diffusion could affect the ECS potential well within the frequency range considered in recordings of the LFP.

**References**

1. Holt G, Koch C. Electrical interactions via the extracellular potential near cell bodies. J Comput Neurosci. 1999;6:169–84.
2. Dietzel I, Heinemann U, Lux H. Relations between slow extracellular potential changes, glial potassium buffering, and electrolyte and cellular volume changes during neuronal hyperactivity in cat. Glia. 1989;2:25–44.
3. Hay E, Hill S, Schürmann F, Markram H, Segev I. Models of neocortical layer 5b pyramidal cells capturing a wide range of dendritic and perisomatic active properties. PLoS Comput Biol. 2011;7(7):e1002107.
4. Halnes G, Østby I, Pettersen KH, Omholt SW, Einevoll GT: Electrodiffusive model for astrocytic and neuronal ion concentration dynamics. PLoS Comput Biol. 2013;9(12):e1003386.

## O16 Large-scale cortical models towards understanding relationship between brain structure abnormalities and cognitive deficits

### Yasunori Yamada^1^

#### ^1^IBM Research - Tokyo, Japan

##### **Correspondence:** Yasunori Yamada - ysnr@jp.ibm.com

*BMC Neuroscience* 2016, **17(Suppl 1)**:O16

**Fig. 9:**
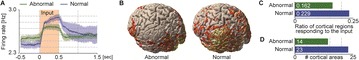
Responses to input to the left V1 in the two cortical models with normal/abnormal structural connectivity. **A** Average firing rates. **B**–**D** Cortical regions and cortical areas that significantly responded to the input

Brain connectivity studies have revealed fundamental properties of normal brain network organization [1]. In parallel, they have reported structural connectivity abnormalities in brain diseases such as Alzheimer’s disease (AD) [1, 2]. However, how these structural abnormalities affect information processing and cognitive functions involved in brain diseases is still poorly understood. To deepen our understanding of this causal link, I developed two large-scale cortical models with *normal* and *abnormal* structural connectivity of diffusion tensor imaging on aging *APOE*-4 non-carriers and carriers in the USC Multimodal Connectivity Database [2, 3]. The possession of the *APOE*-4 allele is one of the major risk factors in developing later AD, and it has known abnormalities in structural connectivity characterized by lower network communication efficiency in terms of local interconnectivity and balance of integration and interconnectivity [2]. The two cortical models share other parameters and consist of 2.4 million spiking neurons and 4.8 billion synaptic connections. First, I demonstrate the biological relevance of the models by confirming that they reproduce normal patterns of cortical spontaneous activities in terms of the following distinctive properties observed in vivo [4]: low firing rates of individual neurons that approximate log-normal distributions, irregular spike trains following a Poisson distribution, a network balance between excitation and inhibition, and greater depolarization of the average membrane potentials. Next, to investigate how the difference in structural connectivity affects cortical information processing, I compare cortical response properties to an input during spontaneous activity between the cortical models. The results show that the cortical model with the abnormal structural connectivity decreased the degree of cortical response as well as the number of cortical regions responding to the input (Fig. 9), suggesting that the structural connectivity abnormality observed in *APOE*-4 carriers might reduce cortical information propagation and lead to negative effects in information integration. Indeed, imaging studies support this suggestion by reporting structural abnormality with lower network communication efficiency observed in the structural connectivity of both *APOE*-4 carriers and AD patients [1, 2]. This computational approach allowing for manipulations and detailed analyses that are difficult or impossible in human studies can help to provide a causal understanding of how cognitive deficits in patients with brain diseases are associated with their underlying structural abnormalities.

**Acknowledgements:** This research was partially supported by the Japan Science and Technology Agency (JST) under the Strategic Promotion of Innovative Research and Development Program.

**References**

1. Stam CJ. Modern network science of neurological disorders. Nat Rev Neurosci. 2014;15(10):683–695.
2. Brown JA, Terashima KH, Burggren AC, Ercoli LM, Miller KJ, Small GW, Bookheimer SY. Brain network local interconnectivity loss in aging *APOE*-4 allele carriers. Proc Natl Acad Sci USA. 2011;108(51):20760–5.
3. Brown JA, Rudie JD, Bandrowski A, van Horn JD, Bookheimer SY. The UCLA multimodal connectivity database: a web-based platform for brain connectivity matrix sharing and analysis. Front Neuroinform. 2012;6(28).
4. Ikegaya Y, Sasaki T, Ishikawa D, Honma N, Tao K, Takahashi N, Minamisawa G, Ujita S, Matsuki N. Interpyramid spike transmission stabilizes the sparseness of recurrent network activity. Cereb Cortex. 2013;23(2):293–304.

## O17 Spatial coarse-graining the brain: origin of minicolumns

### Moira L. Steyn-Ross^1^, D. Alistair Steyn-Ross^1^

#### ^1^School of Engineering, University of Waikato, Hamilton 3240, New Zealand

##### **Correspondence:** Moira L. Steyn-Ross - msr@waikato.ac.nz

*BMC Neuroscience* 2016, **17(Suppl 1)**:O17

The seminal experiments of Mountcastle [1] over 60 years ago established the existence of cortical minicolumns: vertical column-like arrays of approximately 80–120 neurons aligned perpendicular to the pial surface, penetrating all six cortical layers. Minicolumns have been proposed as the fundamental unit for cortical organisation. Minicolumn formation is thought to rely on gene expression and thalamic activity, but exactly why neurons cluster into columns of diameter 30–50 μm containing approximately 100 neurons is not known.

In this presentation we describe a mechanism for the formation of minicolumns via gap-junction diffusion-mediated coupling in a network of spiking neurons. We use our recently developed method of cortical “reblocking” (spatial coarse-graining) [2] to derive neuronal dynamics equations at different spatial scales. We are able to show that for sufficiently strong gap-junction coupling, there exists a minimum block size over which neural activity is expected to be coherent. This coherence region has cross-sectional area of order (40–60 μm)^2^, consistent with the areal extent of a minicolumn. Our scheme regrids a 2D continuum of spiking neurons using a spatial rescaling theory, established in the 1980s, that systematically eliminates high-wave-number modes [3]. The rescaled neural equations describe the bulk dynamics of a larger block of neurons giving “true” (rather than mean-field) population activity, encapsulating the inherent dynamics of a continuum of spiking neurons stimulated by incoming signals from neighbors, and buffeted by ion-channel and synaptic noise.

Our method relies on a perturbative expansion. In order for this coarse-graining expansion to converge, we require not only a sufficiently strong level of inhibitory gap-junction coupling, but also a sufficiently large blocking ratio *B*. The latter condition establishes a lower bound for the smallest “cortical block”: the smallest group of neurons that can respond to input as a collective and cooperative unit. We find that this minimum block-size ratio lies between 4 and 6. In order to relate this 2D geometric result to the 3D extent of a 3-mm-thick layered cortex, we project the cortex onto a horizontal surface and count the number of neurons contained within each *l* × *l* grid micro-cell. Setting *l* ≈ 10 μm and assuming an average of one interneuron per grid cell, a blocking ratio at the mid-value *B* = 5 implies that the side-length of a coherent “macro-cell” will be *L* = *Bl* = 50 μm containing ~25 inhibitory plus 100 excitatory neurons (assuming an *i* to *e* abundance ratio of 1:4) in cross-sectional area *L*^2^. Thus the minicolumn volume will contain roughly 125 neurons. We argue that this is the smallest diffusively-coupled population size that can support cooperative dynamics, providing a natural mechanism defining the functional extent of a minicolumn.

We propose that minicolumns might form in the developing brain as follows: Inhibitory neurons migrate horizontally from the ganglionic eminence to form a dense gap-junction coupled substrate that permeates all layers of the cortex [4]. Progenitor excitatory cells ascend vertically from the ventricular zone, migrating through the inhibitory substrate of the cortical plate. Thalamic input provides low-level stimulus to activate spiking activity throughout the network. Inhibitory diffusive coupling allows a “coarse graining” such that neurons within a particular areal extent respond collectively to the same input. The minimum block size prescribed by the coarse graining imposes constraints on minicolumn geometry, leading to the spontaneous emergence of cylindrical columns of coherent activity, each column centered on an ascending chain of excitatory neurons and separated from neighboring chains by an annular surround of inhibition. This smallest aggregate is preferentially activated during early brain development, and activity-based plasticity then leads to the formation of tangible structural columns.

**References**

1. Mountcastle VB. Modality and topographic properties of single neurons of cat’s somatic sensory cortex. J Neurophysiol. 1957;20(4):408–34.
2. Steyn-Ross ML, Steyn-Ross DA. From individual spiking neurons to population behavior: Systematic elimination of short-wavelength spatial modes. Phys Rev E. 2016;93(2):022402.
3. Steyn-Ross ML, Gardiner CW. Adiabatic elimination in stochastic systems III. Phys Rev A. 1984;29(5):2834–44.
4. Jones EG. Microcolumns in the cerebral cortex. Proc Natl Acad Sci USA. 2000;97(10):5019–21.

## O18 Modeling large-scale cortical networks with laminar structure

### Jorge F. Mejias^1^, John D. Murray^2^, Henry Kennedy^3^, and Xiao-Jing Wang^1,4^

#### ^1^Center for Neural Science, New York University, New York, NY, 10003, USA; ^2^Department of Psychiatry, Yale School of Medicine, New Haven, CT, 06511, USA; ^3^INSERM U846, Stem Cell and Brain Research Institute, Bron Cedex, France; ^4^NYU-ECNU Institute of Brain and Cognitive Science, NYU Shanghai, Shanghai, China

##### **Correspondence:** Jorge F. Mejias - jorge.f.mejias@gmail.com

*BMC Neuroscience* 2016, **17(Suppl 1)**:O18

Visual cortical areas in the macaque are organized according to an anatomical hierarchy, which is defined by specific patterns of anatomical projections in the feedforward and feedback directions [1, 2]. Recent macaque studies also suggest that signals ascending through the visual hierarchy are associated with gamma rhythms, and top-down signals with alpha/low beta rhythms [3–5]. It is not clear, however, how oscillations presumably originating at local populations can give rise to such frequency-specific large-scale interactions in a mechanistic way, or the role that anatomical projections patterns might have in this.

To address this question, we build a large-scale cortical network model with laminar structure, grounding our model on a recently obtained anatomical connectivity matrix with weighted directed inter-areal projections and information about their laminar origin. The model involves several spatial scales—local or intra-laminar microcircuit, inter-laminar circuits, inter-areal interactions and large-scale cortical network—and a wide range of temporal scales—from slow alpha oscillations to gamma rhythms. At any given level, the model is constrained anatomically and then tested against electrophysiological observations, which provides useful information on the mechanisms modulating the oscillatory activity at different scales. As we ascend through the local to the inter-laminar and inter-areal levels, the model allows us to explore the sensory-driven enhancement of gamma rhythms, the inter-laminar phase-amplitude coupling, the relationship between alpha waves and local inhibition, and the frequency-specific inter-areal interactions in the feedforward and feedback directions [3, 4], revealing a possible link with the predictive coding framework.

When we embed our modeling framework into the anatomical connectivity matrix of 30 areas (which includes novel areas not present in previous studies [2, 6]), the model gives insight into the mechanisms of large-scale communication across the cortex, accounts for an anatomical and functional segregation of FF and FB interactions, and predicts the emergence of functional hierarchies, which recent studies have found in macaque [4] and human [5]. Interestingly, the functional hierarchies observed experimentally are highly dynamic, with areas moving across the hierarchy depending on the behavioral context [4]. In this regard, our model provides a strong prediction: we propose that these *hierarchical jumps* are triggered by laminar-specific modulations of input into cortical areas, suggesting a strong link between hierarchy dynamics and context-dependent computations driven by specific inputs.

**References**

1. Felleman DJ, Van Essen DC. Distributed hierarchical processing in the primate cerebral cortex. Cereb Cortex. 1991;1(1):1–47.
2. Markov NT, Vezoli J, Chameau P, Falchier A, Quilodran R, Huissoud C, Lamy C, Misery P, Giroud P, Ullman S, et al. Anatomy of hierarchy: feedforward and feedback pathways in macaque visual cortex. J Comp Neurol. 2014;522:225–259.
3. van Kerkoerle T, Self MW, Dagnino B, Gariel-Mathis MA, Poort J, van der Togt C, Roelfsema PR. Alpha and gamma oscillations characterize feedback and feedforward processing in monkey visual cortex. Proc Natl Acad Sci USA. 2014;111;14332–41.
4. Bastos AM, Vezoli J, Bosman CA, Schoffelen JM, Oostenveld R, Dowdall JR, De Weerd P, Kennedy H, Fries P. Visual areas exert feedforward and feedback influences through distinct frequency channels. Neuron. 2015;85:390–401.
5. Michalareas G, Vezoli J, van Pelt S, Schoffelen JM, Kennedy H, Fries. Alpha–beta and gamma rhythms subserve feedback and feedforward influences among human visual cortical areas. Neuron. 2016;89:384–97.
6. Chaudhuri R, Knoblauch K, Gariel MA, Kennedy H, Wang XJ. A large-scale circuit mechanism for hierarchical dynamical processing in the primate cortex. Neuron. 2015;88:419–31.

## O19 Information filtering by partial synchronous spikes in a neural population

### Alexandra Kruscha^1,2^, Jan Grewe^3,4^, Jan Benda^3,4^ and Benjamin Lindner^1,2^

#### ^1^Bernstein Center for Computational Neuroscience, Berlin, 10115, Germany; ^2^Institute for Physics, Humboldt-Universität zu Berlin, Berlin, 12489, Germany; ^3^Institue for Neurobiology, Eberhardt Karls Universität Tübingen, Germany; ^4^Bernstein Center for Computational Neuroscience, Munich, Germany

##### **Correspondence:** Alexandra Kruscha - alexandra.kruscha@bccn-berlin.de

*BMC Neuroscience* 2016, **17(Suppl 1)**:O19

Synchronous firing of neurons is a prominent feature in many brain areas. Here, we are interested in the information transmission by the synchronous spiking output of a noisy neuronal population, which receives a common time-dependent sensory stimulus. Earlier experimental [1] and theoretical [2] work revealed that synchronous spikes encode preferentially fast (high-frequency) components of the stimulus, i.e. synchrony can act as an information filter. In these studies a rather strict measure of synchrony was used: the entire population has to fire within a short time window. Here, we generalize the definition of the synchronous output, for which only a certain fraction γ of the population needs to be active simultaneously—a setup that seems to be of more biological relevance. We characterize the information transfer in dependence of this fraction and the population size, by the spectral coherence function between the stimulus and the partial synchronous output. We present two different analytical approaches to derive this frequency-resolved measure (one that is more suited for small population sizes, while the second one is applicable to larger populations). We show that there is a critical synchrony fraction, namely the probability at which a single neuron spikes within the predefined time window, which maximizes the information transmission of the synchronous output. At this value, the partial synchronous output acts as a low-pass filter, whereas deviations from this critical fraction lead to a more and more pronounced band-pass filtering effect. We confirm our analytical findings by numerical simulations for the leaky integrate-and-fire neuron. We also show that these findings are supported by experimental recordungs of P-Units electroreceptors of weakly electric fish, where the filtering effect of the synchronous output occurs in real neurons as well.

**Acknowledgement:** This work was supported by Bundesministerium für Bildung und Forschung Grant 01GQ1001A and DFG Grant 609788-L1 1046/2-1.

**References**

1. Middleton JW, Longtin A, Benda J, Maler L. Postsynaptic receptive field size and spike threshold determine encoding of high-frequency information via sensitivity to synchronous presynaptic activity. J Neurophysiol. 2009;101:1160–70.
2. Sharafi N, Benda J, Lindner B. Information filtering by synchronous spikes in a neural population. J Comp Neurosc. 2013;34:285–301.

## O20 Decoding context-dependent olfactory valence in *Drosophila*

### Laurent Badel^1^, Kazumi Ohta^1^, Yoshiko Tsuchimoto^1^, Hokto Kazama^1^

#### ^1^RIKEN Brain Science Institute, 2-1 Hirosawa, Wako, 351-0198, Japan

##### **Correspondence:** Laurent Badel - laurent@brain.riken.jp

*BMC Neuroscience* 2016, **17(Suppl 1)**:O20

Many animals rely on olfactory cues to make perceptual decisions and navigate the environment. In the brain, odorant molecules are sensed by olfactory receptor neurons (ORNs), which convey olfactory information to the central brain in the form of sequences of action potentials. In many organisms, axons of ORNs expressing the same olfactory receptor converge to one or a few glomeruli in the first central region (the antennal lobe in insects and the olfactory bulb in fish and mammals) where they make contact with their postsynaptic targets. Therefore, each glomerulus can be considered as a processing unit that relays information from a specific type of receptor. Because different odorants recruit different sets of glomeruli, and most glomeruli respond to a wide array of odors, olfactory information at this stage of processing is contained in spatiotemporal patterns of glomerular activity. How these patterns are decoded by the brain to guide odor-evoked behavior, however, remains largely unknown.

In *Drosophila*, attraction and aversion to specific odors have been linked to the activation of one or a few glomeruli (reviewed in [1]) in the antennal lobe (AL). These observations suggest a “labeled-line” coding strategy, in which individual glomeruli convey signals of specific ethological relevance, and their activation triggers the execution of hard-wired behavioral programs. However, because these studies used few odorants, and a small fraction of glomeruli were tested, it is unclear how the results generalize to broader odor sets, and whether similar conclusions hold for each of the ~50 glomeruli of the fly AL. Moreover, how compound signals from multiple glomeruli are integrated is poorly understood.

Here, we combine optical imaging, behavioral and statistical techniques to address these questions systematically. Using two-photon imaging, we monitor Ca^2+^ activity in the AL in response to 84 odors. We next screen behavioral responses to the same odorants. Comparing these data allows us to formulate a decoding model describing how olfactory behavior is determined by glomerular activity patterns in a quantitative manner. We find that a weighted sum of normalized glomerular responses recapitulates the observed behavior and predicts responses to novel odors, suggesting that odor valence is not determined solely by the activity a few privileged glomeruli. This conclusion is supported by genetic silencing and optogenetic activation of individual ORN types, which are found to evoke modest biases in behavior in agreement with model predictions. Finally, we test the model prediction that the relative valence of a pair of odors depends on the identity of other odors presented in the same experiment. We find that the relative valence indeed changes, and may even switch, suggesting that perceptual decisions can be modulated by the olfactory context. Surprisingly, our model correctly captured both the direction and the magnitude of the observed changes. These results indicate that the valence of olfactory stimuli is decoded from AL activity by pooling contributions over a large number of glomeruli, and highlight the ability of the olfactory system to adapt to the statistics of its environment, similarly to the visual and auditory systems.

**Reference**

1. Li Q, Liberles SD. Aversion and attraction through olfaction. Curr Biol. 2015;25(3):R120–9.

## P1 Neural network as a scale-free network: the role of a hub

### B. Kahng^1^

#### ^1^Department of Physics and Astronomy, Seoul National University, 08826, Korea

##### **Correspondence:** B. Kahng - bkahng@snu.ac.kr

*BMC Neuroscience* 2016, **17(Suppl 1)**:P1

Recently, increasing attention has been drawn to human neuroscience in network science communities. This is because recent fMRI and anatomical experiments have revealed that neural networks of normal human brain are scale-free networks. Thus, accumulated knowledges in a broad range of network sciences can be naturally applied to neural networks to understand functions and properties of normal and disordered human brain networks. Particularly, the degree exponent value of the human neural network constructed from the fMRI data turned out to be approximately two. This value has particularly important meaning in scale-free networks, because the number of connections to neighbors of a hub becomes largest and thus functional role of the hub becomes extremely important. In this talk, we present the role of the hub in pattern recognition and dynamical problems in association with neuroscience.

## P2 Hemodynamic responses to emotions and decisions using near-infrared spectroscopy optical imaging

### Nicoladie D. Tam^1^

#### ^1^Department of Biological Sciences, University of North Texas, Denton, TX 76203, USA

##### **Correspondence:** Nicoladie D. Tam - nicoladie.tam@unt.edu

*BMC Neuroscience* 2016, **17(Suppl 1)**:P2

This study focuses on the relationship between the emotional response, decision and the hemodynamic responses in the prefrontal cortex. This is based on the computational emotional model that hypothesizes the emotional response is proportional to the discrepancy between the expectancy and the actuality. Previous studies had shown that emotional responses are related to decisions [1, 2]. Specifically, the emotional responses of happy [3], sad [4], angry [5], jealous [6] emotions are proportional to the discrepancy between what one wants and what one gets [1, 3–7].

**Methods** Human subjects are asked to perform the classical behavioral economic experiment called Ultimatum Game (UG) [8]. This experimental paradigm elicits the interrelationship between decision and emotion in human subjects [3–6]. The hemodynamic responses of the prefrontal cortex were recorded while the subjects performed the UG experiment.

**Results** The results showed that the hemodynamic response, which corresponds to the neural activation and deactivation based on the metabolic activities of the neural tissues, are proportional to the emotional intensity and the discrepancy between the expectancy and the actuality. This validates the hypothesis of the proposed emotional theory [9–11] that the intensity of emotion is proportional to the disparity between the expected and the actual outcomes. These responses are also related to the fairness perception [7], with respect to the survival functions [9, 10] similar to the responses established for happy [1] emotion, and for fairness [12] experimentally. This is consistent with the computational relationship between decision and fairness [13].

**References**

1. Tam ND. Quantification of happy emotion: dependence on decisions. Psychol Behav Sci. 2014;3(2):68–74.
2. Tam ND. Rational decision-making process choosing fairness over monetary gain as decision criteria. Psychol Behav Sci. 2014;3(6–1):16–23.
3. Tam ND. Quantification of happy emotion: Proportionality relationship to gain/loss. Psychol Behav Sci. 2014;3(2):60–7.
4. Tam ND: Quantitative assessment of sad emotion. *Psychol Behav Sci* 2015, 4(2):36-43.
5. Tam DN. Computation in emotional processing: quantitative confirmation of proportionality hypothesis for angry unhappy emotional intensity to perceived loss. Cogn Comput. 2011;3(2):394–415.
6. Tam ND, Smith KM. Cognitive computation of jealous emotion. Psychol Behav Sci. 2014;3(6–1):1–7.
7. Tam ND. Quantification of fairness perception by including other-regarding concerns using a relativistic fairness-equity model. Adv Soc Sci Research J. 2014;1(4):159–69.
8. von Neumann J, Morgenstern O, Rubinstein A. Theory of games and economic behavior. Princeton: Princeton University Press; 1953.
9. Tam D. EMOTION-I model: A biologically-based theoretical framework for deriving emotional context of sensation in autonomous control systems. Open Cybern Syst J. 2007;1:28–46.
10. Tam D. EMOTION-II model: a theoretical framework for happy emotion as a self-assessment measure indicating the degree-of-fit (congruency) between the expectancy in subjective and objective realities in autonomous control systems. Open Cybern Syst J. 2007;1:47–60.
11. Tam ND. EMOTION-III model. A theoretical framework for social empathic emotions in autonomous control systems. Open Cybern Syst J. 2016 (in press).
12. Tam ND: Quantification of fairness bias in relation to decisions using a relativistic fairness-equity model. *Adv in Soc Sci Research J* 2014, 1(4):169-178.
13. Tam ND. A decision-making phase-space model for fairness assessment. Psychol Behav Sci. 2014;3(6–1):8–15.

## P3 Phase space analysis of hemodynamic responses to intentional movement directions using functional near-infrared spectroscopy (fNIRS) optical imaging technique

### Nicoladie D. Tam^1^, Luca Pollonini^2^, George Zouridakis^3^

#### ^1^Department of Biological Sciences, University of North Texas, Denton, TX 76203, USA; ^2^College of Technology, the University of Houston, TX, 77204, USA; ^3^Departments of Engineering Technology, Computer Science, and Electrical and Computer Engineering, University of Houston, Houston, TX, 77204, USA

##### **Correspondence:** Nicoladie D. Tam - nicoladie.tam@unt.edu

*BMC Neuroscience* 2016, **17(Suppl 1)**:P3

We aim to extract the intentional movement directions of the hemodynamic signals recorded from noninvasive optical imaging technique, such that a brain-computer-interface (BCI) can be built to control a wheelchair based on the optical signals recorded from the brain. Real-time detection of neurodynamic signals can be obtained using functional near-infrared spectroscopy (fNIRS), which detects both oxy-hemoglobin (oxy-Hb) and deoxy-hemoglobin (deoxy-Hb) levels in the underlying neural tissues. In addition to the advantage of real-time monitoring of hemodynamic signals using fNIRS over fMRI (functional magnetic resonance imaging), fNIRS also can detect brain signals of human subjects in motion without any movement artifacts. Previous studies had shown that hemodynamic responses are correlated with the movement directions based on the temporal profiles of the oxy-Hb and deoxy-Hb levels [1–5]. In this study, we will apply a phase space analysis to the hemodynamic response to decode the movement directions instead of using the temporal analysis in the previous studies.

**Methods** In order to decode the movement directions, human subjects were asked to execute two different orthogonal directional movements in the front-back and right-left directions while the optical hemodynamic responses were recorded in the motor cortex of the dominant hemisphere. We aim to decode the intentional movement directions without a priori any assumption on how arm movement directions are correlated with the hemodynamic signals. Therefore, we used the phase space analysis to determine how the trajectories of oxy-Hb and deoxy-Hb are related to each other during these arm movements.

**Results** The results show that there are subpopulations of cortical neurons that are task-related to the intentional movement directions. Specifically, using phase space analysis of the oxy-Hb and deoxy-Hb levels, opposite movement direction is represented by the different hysteresis of the trajectories in opposite direction in the phase space. Since oxy-Hb represents the oxygen delivery and deoxy-Hb represents the oxygen extraction by the underlying brain tissues, the phase space analysis provides a means to differentiate the movement direction by the ratio between oxygen delivery and oxygen extraction. In other words, the oxygen demands in the subpopulation of neurons in the underlying tissue differ depending on the movement direction. This also corresponds to the opposite patterns of neural activation and deactivation during execution of opposite movement directions. Thus, phase space analysis can be used as an analytical tool to differentiate different movement directions based on the trajectory of the hysteresis with respect to the hemodynamic variables.

**References**

1. Tam ND, Zouridakis G. Optical imaging of motor cortical activation using functional near-infrared spectroscopy. BMC Neurosci. 2012;13(Suppl 1):P27.
2. Tam ND, Zouridakis G. Optical imaging of motor cortical hemodynamic response to directional arm movements using near-infrared spectroscopy. Int J Biol Eng. 2013;3(2):11–17.
3. Tam ND, Zouridakis G. Decoding of movement direction using optical imaging of motor cortex. BMC Neurosci. 2013; P380.
4. Tam ND, Zouridakis G. Temporal decoupling of oxy- and deoxy-hemoglobin hemodynamic responses detected by functional near-infrared spectroscopy (fNIRS). J Biomed Eng Med Imaging. 2014;1(2):18–28.
5. Tam ND, Zouridakis G. Decoding movement direction from motor cortex recordings using near-infrared spectroscopy. In: Infrared spectroscopy: theory, developments and applications. Hauppauge: Nova Science; 2014.

## P4 Modeling jamming avoidance of weakly electric fish

### Jaehyun Soh^1^, DaeEun Kim^1^

#### ^1^Biological Cybernetics, School of Electrical and Electronic Engineering, Yonsei University, Shinchon, Seoul, 120-749, South Korea

##### **Correspondence:** DaeEun Kim - daeeun@yonsei.ac.kr

*BMC Neuroscience* 2016, **17(Suppl 1)**:P4

Weakly electric fish use electric field generated by the electric organ in the tail of the fish. They detect objects by sensing the electric field with electroreceptors on the fish’s body surface. Obstacles in the vicinity of the fish distort the electric field generated by the fish and the fish detect this distortion to recognize environmental situations. Generally, weakly electric fish produce species-dependent electric organ discharge (EOD) signals. Frequency bands of the fish’s signals include a variety of frequencies, 50–600 Hz or higher than 800 Hz. The EOD signals can be disturbed by similar frequency signals emitted by neighboring weakly electric fish. They change their EOD frequencies to avoid jamming signals when they detect the interference of signals. This is called jamming avoidance response (JAR).

**Fig. 10:**
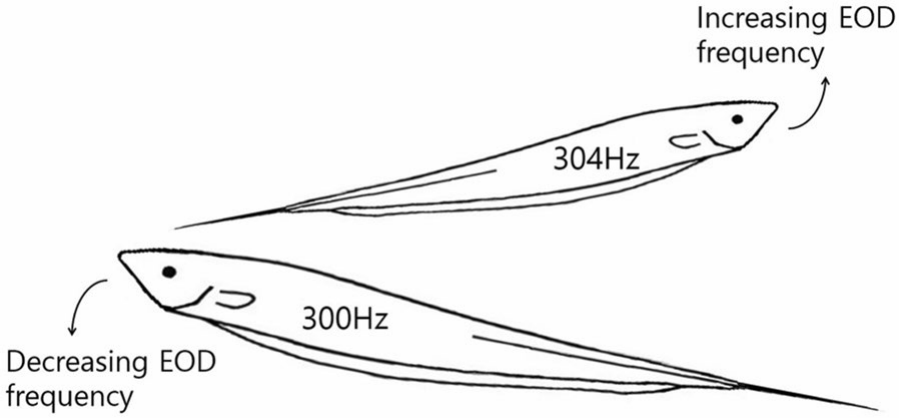
Jamming avoidance response

Electroreceptors of the fish read other electric fish’s EOD while they sense their own EOD. Therefore, when two weakly electric fish are close enough and they sense similar frequencies, their sensing ability by EOD is impaired because of signal jamming [1, 2]. The fish lowers its EOD frequency in response to the jamming signals when a slightly higher frequency of signals are detected and otherwise, raises its EOD. This response is shown in Fig. 10. The fish shift their EOD frequency almost immediately without trial and error.

The method of how to avoid jamming has been studied for a long time, but the corresponding neural mechanisms have not been revealed yet so far. The JAR of *Eigenmannia* can be analyzed by Lissajous graphs which consist of amplitude modulations and differential phase modulations. Relative intensity of signals at each skin can show that the signal frequency is higher than its own signal frequency or lower [3].

We suggest an algorithm of jamming avoidance for EOD signals, especially for wave-type fish. We explore the diagram of amplitude modulation versus phase modulation, and analyze the shape over the graph. The phase differences or amplitude differences will contribute to the estimation of the signal jamming situation. From that, the jammed signal frequency can be detected and so it can guide the jamming avoidance response. It can provide a special measure to predict the jamming avoidance response. However, what type of neural structure is available in weakly electric fish is an open question. We need further study on this subject.

**Acknowledgements:** This work was supported by the National Research Foundation of Korea (NRF) grant funded by the Korea government (MEST) (No. 2014R1A2A1A11053839).

**References**

1. Heiligenberg W. Electrolocation of objects in the electric *fish eigenmannia* (*rhamphichthyidae, gymnotoidei*). J Comp Physiol. 1973;87(2):137–64.
2. Heiligenberg W. Principles of electrolocation and jamming avoidance in electric fish. Berlin: Springer; 1977.
3. Heiligenberg W. Neural nets in electric fish. Cambridge: MIT Press; 1991.

## P5 Synergy and redundancy of retinal ganglion cells in prediction

### Minsu Yoo^1^, S. E. Palmer^1,2^

#### ^1^Committee on Computational Neuroscience, University of Chicago, Chicago, IL, USA; ^2^Department of Organismal Biology and Anatomy, University of Chicago, Chicago, IL, USA

##### **Correspondence:** Minsu Yoo - minsu@uchicago.edu

*BMC Neuroscience* 2016, **17(Suppl 1)**:P5

Recent work has shown that retina ganglion cells (RGC) of salamanders predict future sensory information [1]. It has also been shown that these RGC’s carry significant information about the future state of their own population firing patterns [2]. From the perspective of downstream neurons in the visual system that do not have independent access to the visual scene, the correlations in the RGC firing, itself, may be important for predicting the future visual input. In this work, we explore the structure of the generalized correlation in firing patterns in the RGC, with a particular focus on coding efficiency. From the perspective of efficient neural coding, we might expect neurons to code for their own future state independently (decorrelation across cells), and to have very little predictive information extending forward in time (decorrelation in the time domain).

**Fig. 11:**
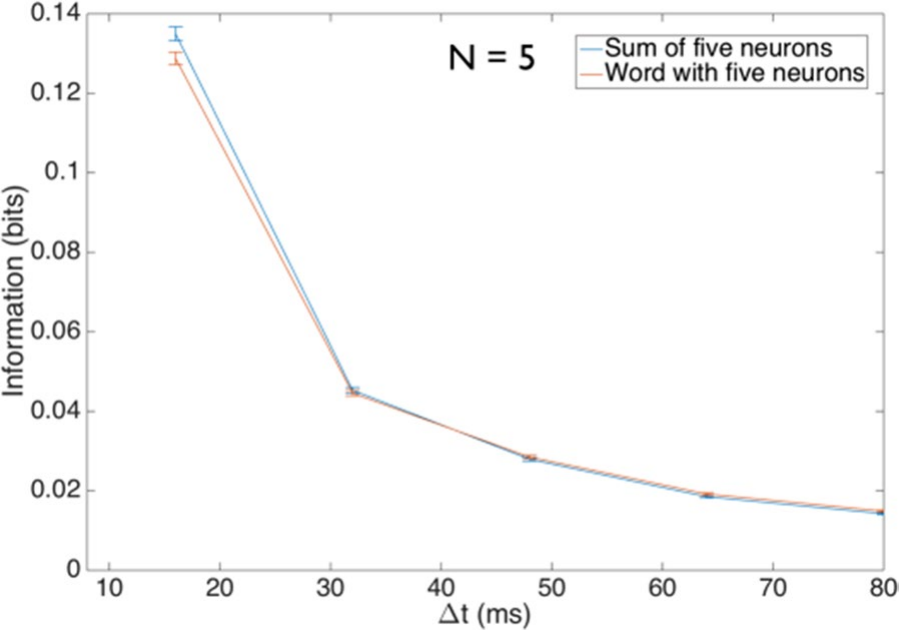
Predictive information in the retinal response is coded for independently. *Red* the mutual information between the binary population firing patterns at times t and t + Δt, for 1000 randomly selected groups of 5 cells from our 31-cell population. Time is binned in 16.67 ms bins, and the (rare) occurrence of two spikes in a bin is recorded as a ‘1’. *Blue* the sum of the mutual information between a single cell response at time t and the future response of the group at time t + Δt. Error bars indicate the standard error of the mean across groups. All information quantities are corrected for finite-size effects using quadratic extrapolation [3]

In this work, we quantify whether neurons in the retina code for their own future input independently, redundantly, or synergistically, and how long these correlations persist in time. We use published extracellular multi-electrode data from the salamander retina in response to repeated presentations of a natural movie [1]. We find significant mutual information in the population firing that is almost entirely independent except at very short time delays, where the code is weakly redundant (Fig. 11). We also find that the information persists to delays of up to a few 100 ms. In addition, we find that individual neurons vary widely in the amount of predictive information they carry about the future population firing state. This heterogeneity may contribute to the diversity of predictive information we find across groups in this experiment.

The results in this study may provide useful information for building a model of the RGC population that can explain why redundant coding is only observed at short delays, or what makes one RGC more predictive than another. Building this type of model will illustrate how the retina represents the future.

**References**

1. Palmer SE, Marre O, Berry MJ, Bialek W. Predictive information in a sensory population. Proc. Natl. Acad. Sci. 2015;112:6908–13.
2. Salisbury J, Palmer SE. Optimal prediction and natural scene statistics in the retina. ArXiv150700125 Q-Bio [Internet]. 2015 [cited 2016 Feb 25]; Available from: http://arxiv.org/abs/1507.00125.
3. Panzeri S, Senatore R, Montemurro MA, Petersen RS. Correcting for the sampling bias problem in spike train information measures. J. Neurophysiol. 2007;98:1064–72.

## P6 A neural field model with a third dimension representing cortical depth

### Viviana Culmone^1^, Ingo Bojak^1^

#### ^1^School of Psychology, University of Reading, Reading, Berkshire, RG1 6AY, UK

##### **Correspondence:** Viviana Culmone - v.culmone@pgr.reading.ac.uk

*BMC Neuroscience* 2016, **17(Suppl 1)**:P6

**Fig. 12:**
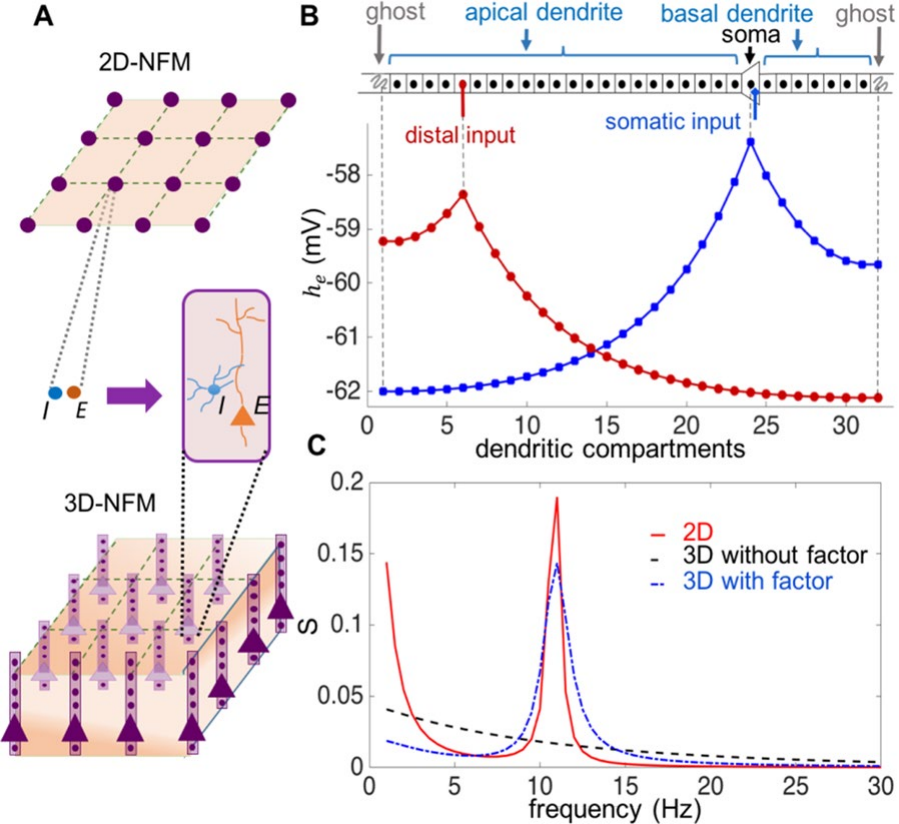
**A** The 3D-NFM adds a dendritic dimension to the 2D one [1]. One single macrocolumn has inhibitory (I) and excitatory (E) subpopulations. **B** (*Top*) Discretization of the dendrite. (*Bottom*) Equilibrium membrane potential along the dendrite for two different synaptic inputs. **C** PSDs of h_e_ for the 2D- and 3D-NFM. Increasing the synaptic input recovers the lost alpha rhythm

Neural field models (NFMs) characterize the average properties of neural ensembles as a continuous excitable medium. So far, NFMs have largely ignored the extension of the dendritic tree, and its influence on the neural dynamics [1]. As shown in Fig. 12A, we implement a 3D-NFM, including the dendritic extent through the cortical layers, starting from a well-known 2D-NFM [2]. We transform the equation for the average membrane potential *h*_*e*_ for the point-like soma in the 2D-NFM [2] to a full cable equation form (added parts in bold):

$$ \begin{aligned} \tau_{e} \frac{{\partial h_{e} (x,z,t)}}{\partial t} & = - \left[ {h_{e} (x,z,t) - h_{e}^{r} } \right] +\varvec{\lambda}^{2} \frac{{\varvec{\partial }^{2} \varvec{h}_{\varvec{e}} (\varvec{x},\varvec{z},\varvec{t})}}{{\varvec{\partial z}^{2} }} \\ & \quad + \varvec{f}_{{\varvec{syn}}} \sum\limits_{k} {\psi_{ke} (h_{e} )I_{ke} (x,z,t)} \\ \end{aligned} $$

The 3D-NFM is modeled considering the dendritic tree as a single linear cable. Figure 12B shows the resulting resting potential along the extended dendrite for synaptic input in two different locations. Naively keeping the parameters of the 2D-NFM for the 3D-NFM results in a power spectral density (PSD) without an alpha rhythm resonance, see Fig. 12C. However, increasing the synaptic input by a factor ***f***_***syn***_ can compensate for the dispersion along the dendrite and recovers the peak in the alpha band. We study the influence of varying the distribution of synaptic inputs along the dendritic (vertical) dimension and of changing the (horizontal) area of the simulated cortical patch. We also provide an outlook on how to compare our results with local field potential recordings from real cortical tissues. We expect that 3D-NFMs will be used widely in the future for describing such experimental data, and that the methods used to extend the specific 2D-NFM used here [2] will generalize to other 2D-NFMs.

**References**

1. Spruston N. Pyramidal neurons: dendritic structure and synaptic integration. Nat Rev Neurosci. 2008;9:206–221.
2. Bojak I, Liley DTJ. Modeling the effects of anesthesia on the electroencephalogram. Phys Rev E. 2005;71:041902.

## P7 Network analysis of a probabilistic connectivity model of the Xenopus tadpole spinal cord

### Andrea Ferrario^1^, Robert Merrison-Hort^1^, Roman Borisyuk^1^

#### ^1^School of Computing and Mathematics, Plymouth University, Plymouth, PL4 8AA, United Kingdom

##### **Correspondence:** Andrea Ferrario - andrea.ferrario@plymouth.ac.uk

*BMC Neuroscience* 2016, **17(Suppl 1)**:P7

Our previous results [1, 2] describe a computational anatomical model of the Xenopus tadpole spinal cord which includes about 1400 neurons of seven types allocated on two sides of the body. This model is based on a developmental approach, where axon growth is simulated and synapses are created (with some probability) when axons cross dendrites. A physiological model of spiking neurons with the generated connectivity of about 85,000 synapses produces a very reliable swimming pattern of anti-phase oscillations in response to simulated sensory input [2].

Using the developmental model we generate 100 different sets of synaptic connections (“connectomes”), and use this information to create a generalized probabilistic model. The probabilistic model provides a new way to easily generate tadpole connectomes and, remarkably, these connectomes produce similar simulated physiological behavior to those generated using the more complex developmental approach (e.g. they swim when stimulated). Studying these generated connectivity graphs allows us to analyze the structure of connectivity in a typical tadpole spinal cord.

Many complex neuronal networks have been found to have “small world” properties, including those in the nematode worm *C. elegans* [3, 6], cat and macaque cortex and the human brain [4]. Small world networks are classified between regular and random networks, and are characterized by a high value of the clustering coefficient *C* and a relatively small value of the average path length *L*, when compared with Erdős-Rényi and degree matched graphs of a similar size. We used graph theory tools to calculate the strongly connected component of each network, which was then used to measure *C* and *L*. For the degree-matched network, these computations have been based on finding the probabilistic generating function [5]. By comparing these measures with those of degree matched random graphs, we found that tadpole’s network can be considered a small world graph. This is also true for the sub-graph consisting only of neurons on one side of the body, which displays properties very similar to those of the *C. elegans* network. Another important subgraph, comprising only the two main neuron types in the central pattern generator (CPG) network also shows small world properties, but is less similar to the *C. elegans* network.

Our approach allows us to study the general properties of the architecture of the tadpole spinal cord, even though in reality the actual network varies from individual to individual (unlike in *C. elegans*). This allows us to develop ideas about the organizing principles of the network, as well as to make predictions about the network’s functionality that can be tested first in computer simulations and later in real animal experiments. In this work we combine several graph theory techniques in a novel way to analyze the structure of a complex neuronal network where not all biological details are known. We believe that this approach can be applied widely to analyze other animals’ nervous systems.

**References**

1. Borisiuk R, al Azad AK, Conte D, Roberts A, Soffe SR. A developmental approach to predicting neuronal connectivity from small biological datasets: a gradient-based neuron growth model. PloS One. 2014;9(2):e89461.
2. Roberts A, Conte A, Hull M, Merrison-Hort R, al Azad AK, Buhl E, Borisyuk R, Soffe SR. Can simple rules control development of a pioneer vertebrate neuronal network generating behavior? J Neurosci. 2014;34(2):608–21.
3. Watts DJ, Strogatz SH. Collective dynamics of ‘small-world’ networks. Nature. 1998;440–2.
4. Kaiser M. A tutorial in connectome analysis: topological and spatial features of brain networks. NeuroImage. 2011;892–907.
5. Newman MEJ, Strogatz SH, Watts DJ. Random graphs with arbitrary degree distribution and their applications. Phys. Rev. 2001;E64:026118.
6. Vershney LR, Chen BL, Paniagua E, Hall DH Chklovskii DB. Structural properties of the *Caenorhabditis elegans* neuronal network. PloS Comput Biol. 2011;7(2):e1001066.

## P8 The recognition dynamics in the brain

### Chang Sub Kim^1^

#### ^1^Department of Physics, Chonnam National University, Gwangju, 61186, Republic of Korea

##### **Correspondence:** Chang Sub Kim - cskim@jnu.ac.kr

*BMC Neuroscience* 2016, **17(Suppl 1)**:P8

Over the years an extensive research endeavor has been given to understanding the brain’s cognitive function in a unified principle and to providing a formulation of the corresponding computational scheme of the brain [1]. The explored free-energy principle (FEP) claims that the brain’s operation on perception, learning, and action rests on brain’s internal mechanism of trying to avoid aberrant events encountering in its habitable environment. The theoretical measure for this biological process has been suggested to be the informational free-energy (IFE). The computational actualization of the FEP is carried out via the gradient descent method (GDM) in machine learning theory.

The information content of the cognitive processes is encoded in the biophysical matter as spatiotemporal patterns of the neuronal correlates of the external causes. Therefore, any realistic attempt to account for the brain function must conform to the physics laws and the underlying principles. Notwithstanding the grand simplicity, however, the FEP framework embraces some extra-physical constructs. Two major such extra-physical constructs are the generalized motions, which are non-Newtonian objects, and the GDM in executing the brain’s computational mechanism of perception and active inference. The GDM is useful in finding mathematical solutions in the optimal problems, but not derived from a physics principle.

In this work, we cast the FEP in the brain science into the framework of the principle of least action (PLA) in physics [2]. The goal is to remove the extra-physical constructs embedded in the FEP and to reformulate the GDM within the standard mechanics arena. Previously, we suggested setting up the minimization scheme of the IFE in the Lagrange mechanics formalism [3] which contained only primitive results. In the present formulation we specify the IFE as the information-theoretic Lagrangian and thus formally define the informational action (IA) as time-integral of the IFE. Then, the PLA prescribes that the viable brain minimizes the IA when encountering uninhabitable events by selecting an optimal path among all possible dynamical configurations in the brain’s neuronal network. Specifically, the minimization yields the mechanistic equations of motion of the brain states, which are inverting algorithms of sensory inputs to infer their external causes. The obtained Hamilton–Jacobi–Bellman-type equation prescribes the brain’s recognition dynamics which do not require the extra-physical concept of higher order motions. Finally, a neurobiological implementation of the algorithm is presented which complies with the hierarchical, operative structure of the brain. In doing so, we adopt the local field potential and the local concentration of ions in the Hodgkin–Huxley model as the effective brain states [4]. Thus, the brain’s recognition dynamics is operatively implemented in a neuro-centric picture. We hope that our formulation, conveying a wealth of structure as an interpretive and mechanistic description of explaining how the brain’s cognitive function may operate, will provide with a helpful guidance for future simulation.

**References**

1. Friston K. The free-energy principle: a unified brain theory? Nat Reivew Neurosci. 2010;11:127–38.
2. Landau LP. Classical mechanics. 2nd ed. NewYork: Springer; 1998.
3. Kim CS. The adaptive dynamics of brains: Lagrangian formulation. Front Neurosci Conf Abstr Neuroinform. 2010. doi:10.3389/conf.fnins.2010.13.00046.
4. Hodgkin A, Huxley A. A quantitative description of membrane current and its application to conduction and excitation in nerve. J Physiol. 1952;117:500–44.

## P9 Multivariate spike train analysis using a positive definite kernel

### Taro Tezuka^1^

#### ^1^Faculty of Library, Information and Media Science, University of Tsukuba, Tsukuba, 305-0821, Japan

##### **Correspondence:** Taro Tezuka - tezuka@slis.tsukuba.ac.jp

*BMC Neuroscience* 2016, **17(Suppl 1)**:P9

Multivariate spike trains, obtained by recording multiple neurons simultaneously, is a key to uncovering information representation in the brain [1]. Other expressions used to refer to the same type of data include “multi-neuron spike train” [2] and “parallel spike train’” [3]. One approach to analyze spike trains is to use kernel methods, which are known to be among the most powerful machine learning methods. Kernel methods rely on defining a symmetric positive-definite kernel suited to the given data. This work proposes a general way of extending kernels on univariate (or single-unit) spike trains to multivariate spike trains.

In this work, the mixture kernel, which naturally extends a kernel defined on univariate spike trains, is proposed and evaluated. There are many univariate spike train kernels proposed [4–9], and the mixture kernel is applicable to any of these kernels. Considered abstractly, a multivariate spike train is a set of time points at which different types of events occurred. In other words, it is a sample taken from a marked point process. The method proposed in this paper is therefore applicable to other data with the same structure.

The mixture kernel is defined as a linear combination of symmetric positive-definite kernels on the components of the target data structure, in this case univariate spike trains. The name “mixture kernel” derives from the common use of the word “mixture” to indicate a linear combination in physics and machine learning, for example in Gaussian mixture models. One can prove that the mixture kernel is symmetric positive-definite if coefficient matrix of the mixture is a symmetric positive-semidefinite matrix.

The performance of the mixture kernel was evaluated by kernel ridge regression for estimating the value of the parameter for generating synthetic spike train data, and also the stimulus given to the animal as the spike trains were recorded. For synthetic data, multivariate spike trains were generated using homogenous Poisson processes. For real data, the pvc-3 data set [2] in the CRCNS (Collaborative Research in Computational Neuroscience) data sharing website was used, which is a 10-unit multivariate spike trains recorded from the primary visual cortex of a cat.

**Acknowledgement:** This work was supported in part by JSPS KAKENHI Grant Numbers 21700121, 25280110, and 25540159.

**References**

1. Gerstner W, Kistler WM, Naud R, Paninski L. Neuronal dynamics. Cambridge: Cambridge University Press; 2014.
2. Blanche T. Multi-neuron recordings in primary visual cortex, CRCNS.org; 2009.
3. Grun S, Rotter S. Analysis of parallel spike trains. Berlin: Springer; 2010.
4. Paiva A, Park IM, Principe JC. A reproducing kernel Hilbert space framework for spike train signal processing, Neural Comput. 2009;21(2):424–49.
5. Park IM, Seth S, Rao M, Principe JC. Strictly positive definite spike train kernels for point process divergences. Neural Comput. 2012;24:2223–50.
6. Park IM, Seth S, Paiva A, Li L, Principe JC. Kernel methods on spike train space for neuroscience: a tutorial. Signal Process Mag. 2013;30(4):149–60.
7. Li L, Park IM, Brockmeier AJ, Chen B, Seth S, Francis JT, Sanchez JC, Principe JC. Adaptive inverse control of neural spatiotemporal spike patterns with a reproducing kernel Hilbert space (RKHS) framework. IEEE Trans Neural Syst Rehabil Eng. 2013;21(4):532–43.
8. Shpigelman L, Singer Y, Paz R, Vaadia E. Spikernels: embedding spiking neurons in inner product spaces. Adv Neural Inf Process Syst. 2003;15:125–32.
9. Eichhorn J, Tolias A, Zien A, Kuss M, Rasmussen CE, Weston J, Logothetis N, Scholkopf B. Prediction on spike data using kernel algorithms. Adv Neural Inf Process Syst. 2004;16:1367–74.

## P10 Synchronization of burst periods may govern slow brain dynamics during general anesthesia

### Pangyu Joo^1^

#### ^1^Physics, POSTECH, Pohang, 37673, Republic of Korea

##### **Correspondence:** Pangyu Joo - pangyu32@postech.ac.kr

*BMC Neuroscience* 2016, **17(Suppl 1)**:P10

**Fig. 13:**
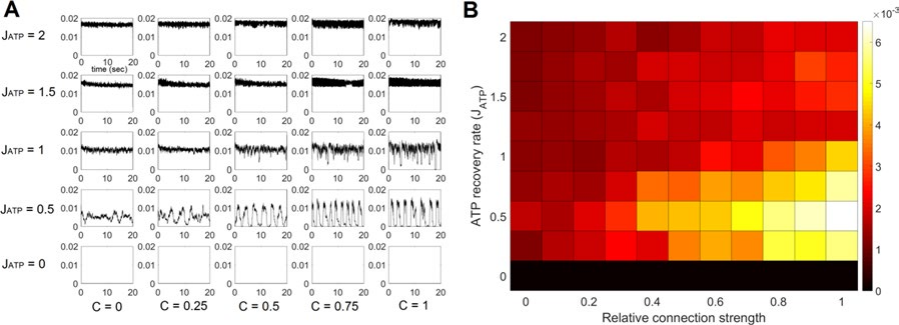
**A** The convoluted signal with different ATP recovery rates (J_ATP_) and relative connection strengths (C). **B** Standard deviation of the convoluted signals

Researchers have utilized electroencephalogram (EEG) as an important key to study brain dynamics in general anesthesia. Representative features of EEG in deep anesthesia are slow wave oscillation and burst suppression [1], and they have so different characteristics that they seem to have different origins. Here, we propose that the two feature may be a different aspect of same phenomenon and show that the slow oscillation could arise from partial synchronization of bursting periods. To model the synchronization of burst periods, modified version of Ching’s model of burst suppression [2] is used. 20 pyramidal neurons and 20 fast spiking neurons are divided into 10 areas composed of 2 pyramidal and 2 fast spiking neurons so that each area exhibit burst suppression behavior independently. Then, all the pyramidal neurons are all to all connected and the connection strength modulates the amount of synchronization of burst periods. The action potentials of pyramidal neurons are substituted by 1 when the action potential larger than 0, and all other case 0. Then they are averaged over the neurons and convoluted with 50 ms square function to see the collective activity of the neurons. As shown in Fig. 13A, At high level of ATP recovery rate (J_ATP_ > 1), there are no suppression period so that slow oscillation does not appear regardless of synchronization. At low level of ATP recovery rate (J_ATP_ = 0.5), we can observe that the slow oscillation appears with increasing amplitude and finally become burst suppression as relative connection strength increases (Fig. 13B). When the ATP recovery rate is 0, then the pyramidal neurons do not fire at all. These results suggest that the burst period synchronization model could explain some important features of EEG during general anesthesia: the increasing slow oscillation amplitude as anesthesia deepen, significantly high activity in bursting period, and the peak max phase amplitude coupling in deep anesthesia.

**References**

1. Purdon PL, Pierce ET, Mukamel EA, et al. Electroencephalogram signatures of loss and recovery of consciousness from propofol. PNAS. 2013;110(12):E1142–51.
2. Ching S, Purdon PL, Vijayan S, Kopell NJ, Brown EN. A neurophysiological–metabolic model for burst suppression. PNAS. 2012;109(8):3095–100.

## P11 The ionic basis of heterogeneity affects stochastic synchrony

### Young-Ah Rho^1,4^, Shawn D. Burton2,3, G. Bard Ermentrout^1,3^, Jaeseung Jeong^4^, Nathaniel N. Urban^2,3^

#### ^1^Department of Mathematics, University of Pittsburgh, Pittsburgh, PA, USA 15260; ^2^Department of Biological Sciences, Carnegie Mellon University, Pittsburgh, PA, USA 15213; ^3^Center for the Neural Basis of Cognition, Pittsburgh, PA, USA 15213; ^4^Department of Bio and Brain Engineering/Program of Brain and Cognitive Engineering, Korea Advanced Institute of Science and Technology (KAIST), Daejeon, South Korea 34141

##### **Correspondence:** Young-Ah Rho - yarho75@gmail.com

*BMC Neuroscience* 2016, **17(Suppl 1)**:P11

Synchronization in neural oscillations is a prominent feature of neural activity and thought to play an important role in neural coding. Theoretical and experimental studies have described several mechanisms for synchronization based on coupling strength and correlated noise input. In the olfactory systems, recurrent and lateral inhibition mediated by dendrodendritic mitral cell–granule cell synapses are critical for synchronization, and intrinsic biophysical heterogeneity reduce the ability to synchronize. In our previous study, a simple phase model was used to examine how physiological heterogeneity in biophysical properties and firing rates across neurons affects correlation-induced synchronization (stochastic synchrony). It has showed that heterogeneity in the firing rates and in the shapes of the phase response curves (PRCs) reduced output synchrony. In this study, we extend the previous phase model to a conductance based model to examine how the density of specific ion channels in mitral cells impacts on stochastic synchrony. A recent study revealed that mitral cells are highly heterogeneous in the expression of the sag current, a hyperpolarization-activated inward current (Angelo, 2011). The variability in the sag contributes to the diversity of mitral cells and thus we wanted to know how this variability influences synchronization. Mitral cell oscillations and bursting are also regulated by an inactivating potassium current (I_A_). Based on these ion channels, we examined the effect of changing the current densities (g_A_, g_H_) on diversity of PRCs and of synchrony. In order to identify oscillatory patterns of bursting and repetitive spiking across g_A_ and g_H_ to the model, two parameter bifurcation analysis was performed in the presence and absence of noise. Increasing g_H_ alone reduces the region of bursting, but does not completely eliminate bursting, and PRCs changed much more with respect to g_A_ than g_H_. Focusing on varying g_A_, we next examined a role of g_A_ density and firing rate in stochastic synchrony by introducing the fluctuating correlated input resembling the shared presynaptic drives. We found that heterogeneity in A-type current mainly influenced on stochastic synchrony as we predicted in PRCs investigated theoretically, and diversity in firing rate alone didn’t account for it. In addition, heterogeneous population with respect to g_A_, given decent amount of g_A_ density, showed better stochastic synchrony than homogeneous population in same firing rate.

## P12 Circular statistics of noise in spike trains with a periodic component

### Petr Marsalek^1,2^

#### ^1^Institute of Pathological Physiology, First Faculty of Medicine, Charles University in Prague, 128 53, Czech Republic; ^2^Czech Technical University in Prague, Zikova 1903/4, 166 36, Czech Republic

##### **Correspondence:** Petr Marsalek - petr.marsalek@lf1.cuni.cz

*BMC Neuroscience* 2016, **17(Suppl 1)**:P12

**Introduction** We estimate parameters of the inter-spike interval distributions in binaural neurons of the mammalian sound localization neural circuit, neurons of the lateral and medial superior olive [1]. We present equivalent descriptions of spike time probabilities using both standard and circular statistics. We show that the difference between sine function and beta density in the circular domain is negligible.

**Fig. 14:**
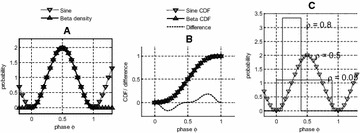
Comparison of circular probability density functions of sine and beta density. **A** Beta density with parameters a = b = 3.3818, matches closely that of the sine function, used as a probability density function (PDF). Beta density with parameters a = b = 3 *solid line*, is matched by sine function y = 1.05 − 1.1 cos(2π x/1.1). **B** Cumulative distribution function (CDF) is shown for these densities together with the difference between the two CDFs multiplied by 100 to visualize the comparison of the two distributions. **C** For testing different vector strengths we use uniform distributions with pre-set vector strengths (ρ = 0.8, 0.5 and 0.08)

**Results** Estimation of the spike train probability density function parameters is presented in relation to harmonic and complex sound input. The resulting densities are expressed analytically with the use of harmonic and Bessel functions. Parameter fits are verified by numerical simulations of spike trains (Fig. 14).

**Conclusions** We use analytical techniques, where it is possible. We calculate the one-to-one correspondence of vector strength parameters and parameters of circular distributions used for description of data. We show here introductory figure of our paper with the two representative circular densities. We also use experimental data [2, 3] and simulated data to compare them with these theoretical distributions.

**Acknowledgements:** Supported by the PRVOUK program no. 205024 at the Charles University in Prague. I acknowledge contributions to the analytical computations by Ondrej Pokora and simulation in Matlab by Peter G. Toth.

**References**

1. Bures Z, Marsalek P. On the precision of neural computation with interaural level differences in the lateral superior olive. Brain Res. 2013;1536:16–26.
2. Joris P, Carney L, Smith P, Yin T. Enhancement of neural synchronization in the anteroventral cochlear nucleus. I. Responses to tones at the characteristic frequency. J Neurophysiol. 1994;71(3):1022–36.
3. Joris P, Smith P, Yin T. Enhancement of neural synchronization in the anteroventral cochlear nucleus. II. Responses in the tuning curve tail. J Neurophysiol. 1994;71(3):1037–51.

## P14 Representations of directions in EEG-BCI using Gaussian readouts

### Hoon-Hee Kim^1,2^, Seok-hyun Moon^3^, Do-won Lee^3^, Sung-beom Lee^3^, Ji-yong Lee^3^, Jaeseung Jeong^1,2^

#### ^1^Department of Bio and Brain Engineering and ^2^Program of Brain and Cognitive Engineering, College of Engineering, Korea Advanced Institute of Science and Technology (KAIST), Daejeon, South Korea, 34141; ^3^Korea Science Academy of KAIST, Busan, South Korea, 10547

##### **Correspondence:** Jaeseung Jeong - jsjeong@kaist.ac.kr

*BMC Neuroscience* 2016, **17(Suppl 1)**:P14

**Fig. 15:**
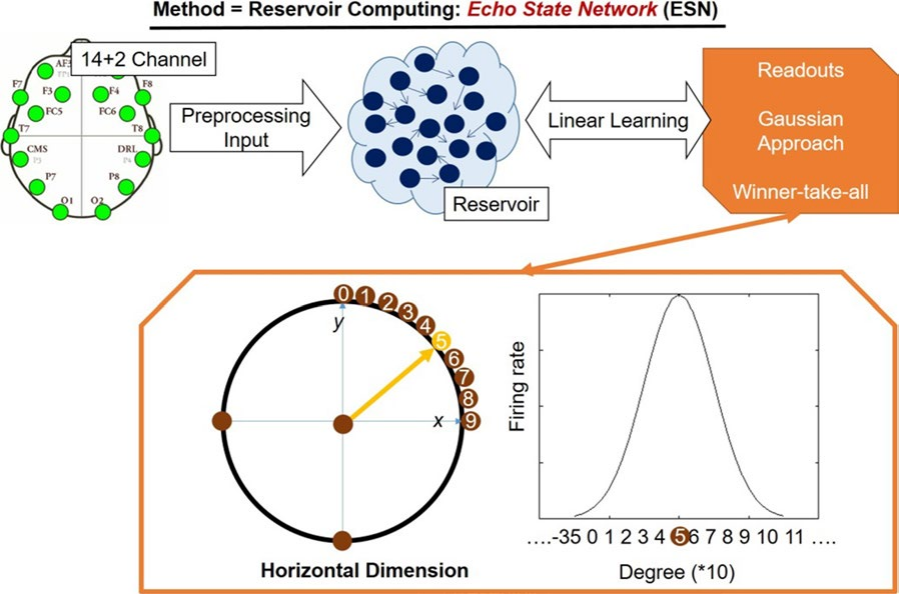
Design of recurrent neural networks and readouts

EEG (electroencephalography) is one of most useful neuroimaging technology and best options for BCI (Brain-Computer Interface) because EEG has portable size, wireless and well-wearing design in any situations. The key objective of BCI is physical control of machine such as cursor movement in screen and robot movement [1, 2]. In previously study, the motor imagery had used for represent of direction to movement [1, 2]. For example, the left hand imagery mapping to move the left, the right hand imagery mapping to move the right and both hand imagery mapping to move the forward. In this study, however, we considered only brain signals when a subject thinks directions to movements not motor imageries. We designed the recurrent neural networks which consist of 300–10,000 artificial linear neurons using Echo State Networks paradigm [3]. We also recorded EEG signals using Emotiv EPOC+ which has 16 channels (AF3, F7, F3, FC5, T7, P7, O1, O2, P8, T8, FC6, F4, F8, AF4 and two of reference). All raw data of channels were normalized and then used inputs to recurrent neural networks. For representation of directions, we had built Gaussian readouts which has preferred directions and fitted the Gaussian functions (Fig. 15). The firing rate of readout were high when the subject thought preferred direction. However, when the subject thought not preferred direction, the firing rate of readout slightly low down. For implement these readouts, all of neuros in recurrent neural networks had linearly connected to all readouts and weights of these connections were trained by linear learning rules. In result, we considered 5 healthy subjects and recorded EEG signals for each directions. The readouts were showed well Gaussian fitted direction preference. In this study, we considered only two dimensions but many situations of BCI has three dimensional space. Therefore, our study which using Gaussian readouts should be extended to three dimensional version.

**References**

1. Chae Y, Jeong J, Jo S. Toward brain-actuated humanoid robots: asynchronous direct control using an EEG-based BCI. IEEE Trans Robot. 2012;28(5):1131–44.
2. LaFleur K, Cassady K, Doud A, Shades K, Rogin E, He B. Quadcopter control in three-dimensional space using a noninvasive motor imagery-based brain–computer interface. J Neural Eng. 2013;10(4):046003.
3. Jaeger H, Haas H. Harnessing nonlinearity predicting chaotic systems and saving energy in wireless communication. Science. 2004;304(5667):78–80.

## P15 Action selection and reinforcement learning in basal ganglia during reaching movements

### Yaroslav I. Molkov^1^, Khaldoun Hamade^2^, Wondimu Teka^3^, William H. Barnett^1^, Taegyo Kim^2^, Sergey Markin^2^, Ilya A. Rybak^2^

#### ^1^Department of Mathematics and Statistics, Georgia State University, Atlanta, GA 30303, USA; ^2^Department of Neurobiology and Anatomy, Drexel University, Philadelphia, PA 19129, USA; ^3^Department of Mathematical Sciences, Indiana University – Purdue University, Indianapolis, IN 46202, USA

##### **Correspondence:** Yaroslav I. Molkov - ymolkov@gsu.edu

*BMC Neuroscience* 2016, **17(Suppl 1)**:P15

The basal ganglia (BG) comprise a number of interconnected nuclei that are collectively involved in a wide range of motor and cognitive behaviors. The commonly accepted theory is that the BG play a pivotal role in action selection and reinforcement learning facilitated by the activity of dopaminergic neurons of substantia nigra pars compacta (SNc). These dopaminergic neurons encode prediction errors when reward outcomes exceed or fall below anticipated values. The BG gate appropriate behaviors from multiple moto-cortical command candidates arriving at the striatum (BG’s input nuclei) but suppress competing inappropriate behaviors. The selected motor action is realized when the internal segment of the globus pallidus (GPi) (BG’s output nuclei) disinhibits thalamic neurons corresponding to the gated behavior. The BG network performs motor command selection through the facilitation of the appropriate behavior via the “direct” striatonigral (GO) pathway and inhibition of competing behaviors by the “indirect” striatopallidal (NOGO) pathway.

Several modeling studies have showed plausibility of the above concept in simplified cases, e.g. for binary action selection in response to a binary cue. However, in these previous models, the possible actions/behaviors were represented in an abstract way, and did not have a detailed implementation as specific neuronal patterns actuating the muscular-skeletal apparatus. To address these details, the motor system in the present study includes a 2D-biomechanical arm model in the horizontal plane to simulate realistic reaching movements. The arm consists of two segments (upper arm and forearm) and has two joints (shoulder and elbow) controlled by four monoarticular (flexor and extensor at each joint) and two bi-articular (shoulder and elbow flexor, and shoulder and elbow extensor) muscles. The neural component of the model includes the BG, the thalamus, the motor cortex, and spinal circuits. The low-level spinal circuitry contains six motoneurons (each controlling one muscle), and receives proprioceptor feedback from muscles. Cortical neurons provide inputs to the spinal network. Their activity is calculated by solving an inverse problem (inverting the internal model) based on the initial position of the arm, reaching distance and direction.

In the model, reaching movements in different directions were used as a set of possible behaviors. We simulated movements in response to a sensory cue defining the target arm position. The cortex generated signals corresponding to the cue and all possible motor commands and delivered these signals to the BG. The resulting neuronal patterns in the motor cortex were calculated as a convolution of the thalamic activity and all possible motor commands. The function of BG was to establish the association between the cue and the appropriate action(s) by adjusting weights of plastic corticostriatal projections through reinforcement learning. The BG model contained an exploratory mechanism, operating through the subthalamic nucleus (STN) that allowed the model to constantly seek better cue-action associations that deliver larger rewards. Reinforcement learning relied on the SNc dopaminergic signal that measured trial-to-trial changes in the reward value, defined by performance errors.

Using this model, we simulated several learning tasks in the conditions of different unexpected perturbations. When a perturbation was introduced, the model was capable of quickly switching away from pre-learned associations and learning novel cue-action associations. The analysis of the model reveals several features, that can have general importance for brain control of movements: (1) potentiation of the cue-NOGO projections is crucial for quick destruction of preexisting cue-action associations; (2) the synaptic scaling (the decay of the cortical-striatal synaptic weights in the absence of dopamine-mediated potentiation/depression) has a relatively short time-scale (10–20 trials); (3) quick learning is associated with a relatively poor accuracy of the resultant movement. We suggest that BG may be involved in a quick search for behavioral alternatives when the conditions change, but not in the learning of skilled movements that require good precision.

## P17 Axon guidance: modeling axonal growth in T-junction assay

### Csaba Forro^1^, Harald Dermutz^1^,László Demkó^1^, János Vörös^1^

#### ^1^LBB, ETH Zürich, Zürich, 8051, Switzerland

##### **Correspondence:** Csaba Forro - forro@biomed.ee.ethz.ch

*BMC Neuroscience* 2016, **17(Suppl 1)**:P17

The current field of neuroscience investigates the brain at scales varying from the whole organ, to brain slices and down to the single cell level. The technological advances miniaturization of electrode arrays has enabled the investigation of neural networks comprising several neurons by recording electrical activity from every individual cell in the network. This level of complexity is key in the study of the core principles at play in the machinery of the brain. Indeed, it is the first layer of complexity above the single cell that is still tractable for the human scientist without needing to resort to a ‘Big Data’ approach. In light of this, we strive to create topologically well-defined neural networks, akin to mathematical directed graphs, as a model systems in order to study the basic mechanisms emerging in networks of increasing complexity and varying topology. This approach will also yield statistically sound and reproducible observations, something which is sought after in neuroscience [1].

**Fig. 16:**
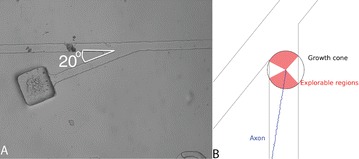
**A** The T-junction assay with an entry angle of 20°. The axon is expected to prefer a right-turn at this angle. **B** A simple model is constructed where the direction of growth of the axon is proportional to area (*red*) it can explore

The first step in realizing such a well-defined neural network is to reliably control the guidance of individual axons in order to connect the network of cells in a controlled way. For this purpose, we present a method consisting of obstacles forcing the axon to turn one way or the other. The setup is made of PolyDiMethylSiloxane (PDMS) which is microstructured by ways of state of the art photolithography procedures. Two tunnels of 5 µ height are patterned into a block of 100 µ thick PDMS and connected in the shape of a T-junction (Fig. 16). Primary cortical neurons are inserted via entry holes at the base of the tunnels. The entry angle of the bottom tunnel (“vertical part of the T”) into the junction is varied between 20° (steep entry) and 90° (vertical entry). We observe that the axons prefer to turn towards the smaller angle. We show how this observed angular selectivity in axon guidance can be explained by a simple model and how this principle can be used to create topologically well-defined neural networks (Fig. 16B).

**Reference**

1. Button KS, et al. Power failure: why small sample size undermines the reliability of neuroscience. Nat Rev Neurosci. 2013;14(5):365–76.

## P19 Transient cell assembly networks encode persistent spatial memories

### Yuri Dabaghian^1,2^, Andrey Babichev^1,2^

#### ^1^Department of Neurology Pediatrics, Baylor College of Medicine, Houston, TX 77030, USA; ^2^Department of Computational and Applied Mathematics, Rice University, Houston, TX, 77005, USA

##### **Correspondence:** Yuri Dabaghian - dabaghian@rice.edu

*BMC Neuroscience* 2016, **17(Suppl 1)**:P19

The reliability of our memories is nothing short of remarkable. Thousands of neurons die every day, synaptic connections appear and disappear, and the networks formed by these neurons constantly change due to various forms of synaptic plasticity. How can the brain develop a reliable representation of the world, learn and retain memories despite, or perhaps because of, such complex dynamics? Here we consider the specific case of spatial navigation in mammals, which is based on mental representations of their environments—cognitive maps—provided by the network of the hippocampal place cells—neurons that become active only in a particular region of the environment, known as their respective place fields. Experiments suggest that the hippocampal map is fundamentally topological, i.e., more similar to a subway map than to a topographical city map, and hence amenable to analysis by topological methods [1]. By simulating the animal’s exploratory movements through different environments we studied how stable topological features of space get represented by assemblies of simulated neurons operating under a wide range of conditions, including variations in the place cells’ firing rate, the size of the place fields, the number of cells in the population [2,3]. In this work, we use methods from Algebraic Topology to understand how the dynamic connections between hippocampal place cells influence the reliability of spatial learning. We find that although the hippocampal network is highly transient, the overall spatial map encoded by the place cells is stable.

**Acknowledgements:** The work was supported by the NSF 1422438 grant and by the Houston Bioinformatics Endowment Fund.

**References**

1. Dabaghian Y, Brandt VL, Frank LM. Reconceiving the hippocampal map as a topological template. eLife. 2014. doi:10.7554/eLife.03476.
2. Dabaghian Y, Mémoli F, Frank L, Carlsson G. A topological paradigm for hippocampal spatial map formation using persistent homology. PLoS Comput Biol. 2012;8:e1002581.
3. Arai M, Brandt V, Dabaghian Y. The effects of theta precession on spatial learning and simplicial complex dynamics in a topological model of the hippocampal spatial map. PLoS Comput Biol. 2014;10:e1003651.

## P20 Theory of population coupling and applications to describe high order correlations in large populations of interacting neurons

### Haiping Huang^1^

#### ^1^RIKEN Brain Science Institute, Wako-shi, Saitama, Japan

##### **Correspondence:** Haiping Huang - physhuang@gmail.com

*BMC Neuroscience* 2016, **17(Suppl 1)**:P20

Correlations among neurons spiking activities play a prominent role in deciphering the neural code. Various models were proposed to understand the pairwise correlations in the population activity. Modeling these correlations sheds light on the functional organization of the nervous system. In this study, we interpret correlations in terms of population coupling, a concept recently proposed to understand the multi-neuron firing patterns of the visual cortex of mouse and monkey [1]. We generalize the population coupling to its higher order (PC2), characterizing the relationship of pairwise firing with the population activity. We derive the practical dimensionality reduction method for extracting the low dimensional representation parameters, and test our method on different types of neural data, including ganglion cells in the salamander retina onto which a repeated natural movie was projected [2], and layer 2/3 as well as layer 5 cortical cells in the medial prefrontal cortex (MPC) of behaving rats [3].

For the retinal data, by considering the correlation between the pairwise firing activity and the global population activity, i.e., the second order population coupling, the three-cell correlation could be predicted partially (64.44 %), which suggests that PC2 acts as a key circuit variable for third order correlations. The interaction matrix revealed here may be related to the found overlapping modular structure of retinal neuron interactions [4]. In this structure, neurons interact locally with their adjacent neurons, and in particular this feature is scalable and applicable for larger networks.

About 94.79 % of three-cell correlations are explained by PC2 in the MPC circuit. The PC2 matrix shows clear hubs’ structure in the cortical circuit. Some neuron interacts strongly with a large portion of neurons in the population, and such neurons may play a key role in shaping the collective spiking behavior during the working memory task. The hubs and non-local effects are consistent with findings reported in the original experimental paper [3].

**Acknowledgements:** We are grateful to Shigeyoshi Fujisawa and Michael J Berry for sharing us the cortical and retinal data, respectively. We also thank Hideaki Shimazaki and Taro Toyoizumi for stimulating discussions. This work was supported by the program for Brain Mapping by Integrated Neurotechnologies for Disease Studies (Brain/MINDS) from Japan Agency for Medical Research and development, AMED.

**References**

1. Okun M, Steinmetz NA, Cossell L, Iacaruso MF, Ko H, Bartho P, et al. Diverse coupling of neurons to populations in sensory cortex. Nature. 2015;521:511–15.
2. Tkacik G, Marre O, Amodei D, Schneidman E, Bialek W, Berry II MJB. Searching for collective behavior in a large network of sensory neurons. PLoS Comput Biol. 2014;10:e1003408.
3. Fujisawa S, Amarasingham A, Harrison MT, Buzsaki G. Behavior-dependent short-term assembly dynamics in the medial prefrontal cortex. Nat Neurosci. 2008;11:823–33.
4. Ganmor E, Segev R, Schneidman E. The architecture of functional interaction networks in the retina. J Neurosci. 2011;31(8):3044–54.

## P21 Design of biologically-realistic simulations for motor control

### Sergio Verduzco-Flores^1^

#### ^1^Computational Neuroscience Unit, Okinawa Institute of Science and Technology, Okinawa 1919-1, Japan

##### **Correspondence:** Sergio Verduzco-Flores - sergio.verduzco@oist.jp

*BMC Neuroscience* 2016, **17(Suppl 1)**:P21

Several computational models of motor control, although apparently feasible, fail when simulated in 3-dimensional space with redundant manipulators [1, 2]. Moreover, it has become apparent that the details of musculoskeletal simulations, such as the muscle model used, can fundamentally affect the conclusions of a computational study [3].

There would be great benefits from being able to test theories involving motor control within a simulation framework that brings realism in the musculoskeletal model, and in the networks that control movements. In particular, it would be desirable to have: (1) a musculoskeletal model considered to be research-grade within the biomechanics community, (2) afferent information provided by standard models of the spindle afferent and the Golgi tendon organ, (3) muscle stimulation provided by a spiking neural network that follows the basic known properties of the spinal cord, and (4) a cerebellar network as part of adaptive learning.

Creating this type of model is only now becoming practical, not only due to faster computers, but due to properly validated musculoskeletal models and simulation platforms from the biomechanics community, as well as mature software and simulations techniques from the computational neuroscience community. We show how these can be harnessed in order to create simulations that are grounded both by physics and by neural implementation. This pairing of computational neuroscience and biomechanics is sure to bring further insights into the workings of the central nervous system.

**References**

1. Gielen S. Review of models for the generation of multi-joint movements in 3D. In: Sternad D, editor. Progress in motor control. New-York: Springer; 2009.
2. Verduzco-Flores SO, O’Reilly RC. How the credit assignment problems in motor control could be solved after the cerebellum predicts increases in error. Front Comput Neurosci. 2015;9:39.
3. Gribble PL, Ostry DJ, Sanguineti V, Laboissière R. Are complex control signals required for human arm movement? J Neurophysiol. 1998;79:1409–24.

## P22 Towards understanding the functional impact of the behavioural variability of neurons

### Filipa Dos Santos^1^, Peter Andras^1^

#### ^1^School of Computing and Mathematics, Keele University, Newcastle-under-Lyme, ST5 5BG, UK

##### **Correspondence:** Filipa Dos Santos - f.d.s.brandao@keele.ac.uk

*BMC Neuroscience* 2016, **17(Suppl 1)**:P22

**Fig. 17:**
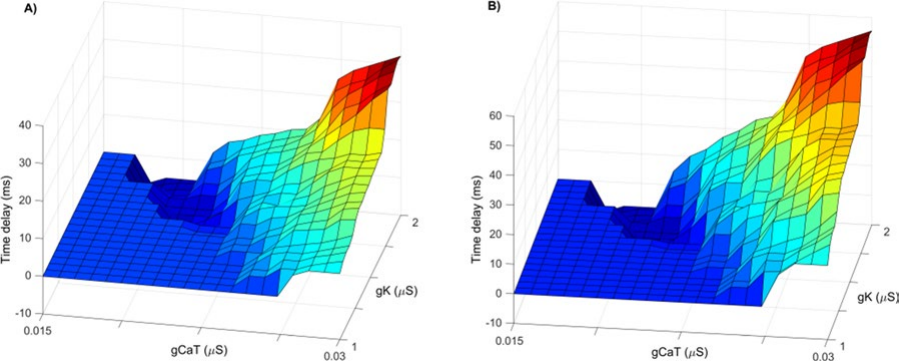
The time distances between the first and second spikes of the simulated PD neurons as a function of the g_K_ and g_CaT_ conductances of the neuron with variable conductances. **A** first spikes. **B** Second spikes. The PD neuron with fixed conductances had g_K_ = 1.5768 μS and g_CaT_ = 0.0225 μS

The same neuron may play different functional roles in the neural circuits to which it belongs. For example, neurons in the Tritonia pedal ganglia may participate in variable phases of the swim motor rhythms [1]. While such neuronal functional variability is likely to play a major role the delivery of the functionality of neural systems, it is difficult to study it in most nervous systems. We work on the pyloric rhythm network of the crustacean stomatogastric ganglion (STG) [2]. Typically network models of the STG treat neurons of the same functional type as a single model neuron (e.g. PD neurons), assuming the same conductance parameters for these neurons and implying their synchronous firing [3, 4]. However, simultaneous recording of PD neurons shows differences between the timings of spikes of these neurons. This may indicate functional variability of these neurons. Here we modelled separately the two PD neurons of the STG in a multi-neuron model of the pyloric network. Our neuron models comply with known correlations between conductance parameters of ionic currents. Our results reproduce the experimental finding of increasing spike time distance between spikes originating from the two model PD neurons during their synchronised burst phase. The PD neuron with the larger calcium conductance generates its spikes before the other PD neuron. Larger potassium conductance values in the follower neuron imply longer delays between spikes, see Fig. 17.

Neuromodulators change the conductance parameters of neurons and maintain the ratios of these parameters [5]. Our results show that such changes may shift the individual contribution of two PD neurons to the PD-phase of the pyloric rhythm altering their functionality within this rhythm. Our work paves the way towards an accessible experimental and computational framework for the analysis of the mechanisms and impact of functional variability of neurons within the neural circuits to which they belong.

**References**

1. Hill ES, Vasireddi SK, Bruno AM, Wang J, Frost WN. Variable neuronal participation in stereotypic motor programs. PLoS One. 2012;7:1–11.
2. Bucher D, Johnson CD, Marder E. Neuronal morphology and neuropil structure in the stomatogastric ganglion. J Comp Neurol. 2007;501:185–205.
3. Soto-Treviño C, Rabbah P, Marder E, Nadim F. Computational model of electrically coupled, intrinsically distinct pacemaker neurons. J Neurophysiol. 2005;94:590–604.
4. Golowasch J, Casey M, Abbott LF, Marder E. Network stability from activity-dependent regulation of neuronal conductances. Neural Comput. 1999;11:1079–96.
5. Khorkova O, Golowasch J. Neuromodulators, not activity, control coordinated expression of ionic currents. J Neurosci. 2007;27:8709–18.

## P23 Different oscillatory dynamics underlying gamma entrainment deficits in schizophrenia

### Christoph Metzner^1^, Achim Schweikard^2^, Bartosz Zurowski^3^

#### ^1^Science and Technology Research Institute, University of Hertfordshire, Hatfield, United Kingdom; ^2^Institute for Robotics and Cognitive Systems, University of Luebeck, Luebeck, Germany; ^3^Department of Psychiatry, University of Luebeck, Schleswig–Holstein, Luebeck, Germany

##### **Correspondence:** Filipa Dos Santos - c.metzner@herts.ac.uk

*BMC Neuroscience* 2016, **17(Suppl 1)**:P23

In recent years, a significant amount of biomarkers and endophenotypic signatures of psychiatric illnesses have been identified, however, only a very limited number of computational models in support thereof have been described so far [1]. Furthermore, the few existing computational models typically only investigate one possible mechanism in isolation, disregarding the potential multifactoriality of the network behaviour [2]. Here we describe a computational instantiation of an endophenotypic finding for schizophrenia, an impairment in gamma entrainment in auditory click paradigms [3].

We used a model of primary auditory cortex from Beeman [4] and simulated a click entrainment paradigm with stimulation at 40 Hz, to investigate gamma entrainment deficits, and at 30 Hz as a control condition. We explored the multifactoriality by performing an extensive parameter search (approx. 4000 simulations). We focused on synaptic and connectivity parameters of the fast spiking inhibitory interneurons in the model (i.e. number and strength of and, GABAergic decay times at I-to-E and I-to-I connections, independently). We performed a time–frequency analysis of simulated EEG signals and extracted the power in the 40 Hz and the 30 Hz band, respectively. Using the power in the 40 Hz band for 40 Hz stimulation we identified regions in the parameter space showing strong reductions in gamma entrainment. For these we calculated cycle-averaged EEG signals and spike time histograms of both network populations, in order to explore the dynamics underlying the reduction in gamma power.

We find three regions in the parameter space which show strong reductions in gamma power. These three regions, however, have very different parameter settings and show very different oscillatory dynamics. The first, which produces the strongest reduction, is characterised by a strong prolongation of decay times at I-to-E synapses and strong and numerous I-to-E connections. Cycle-averaged spike histograms show a broadening of distributions which indicate that the overall synchrony is reduced, leading to the strong reduction in gamma power. However, this parameter setting also produced a strong reduction of power in the 30 Hz control condition, which is not seen experimentally. The second region, is characterized by prolonged I-to-I decay times together with numerous and strong I-to-I connectivity. Here, a second peak appears in the cycle-average spike histogram of the excitatory population, which leads to a loss of synchrony and thus a reduction in gamma power. The third parameter region, is also characterized by prolonged I-to-I decay times. Moreover, it is associated with a reduction in I-to-I connection numbers and strengths together with strong I-to-E connections. Here, we found that in every second cycle, the spike histogram of the inhibitory neurons showed two peaks, one at the beginning and one in the middle of the cycle. This second peak then inhibited the excitatory neurons’ response to the next stimulation. Hence, the EEG signal showed beat-skipping, i.e. every second gamma peak was suppressed, resulting in a decrease in gamma power.

Performing an extensive parameter search in an in silico instantiation of an endophenotypic finding for schizophrenia, we have identified distinct regions of the parameter space that give rise to analogous network level behaviour found in schizophrenic patients using electrophysiology [3]. However, the oscillatory dynamics underlying this behaviour substantially differ across regions. These regions might correspond to different subtypes of schizophrenic patients and hence, subtypes of what might have different targets for alleviating the deficits because of their differences in underlying dynamics.

**References**

1. Siekmeier P. Computational modeling of psychiatric illnesses via well-defined neurophysiological and neurocognitive biomarkers. Neurosci Biobehav Rev. 2015;57:365–80.
2. Pavão, R, Tort ABL, Amaral OB. Multifactoriality in psychiatric disorders: a computational study of schizophrenia. Schizophrenia Bull. 2015;41(4):980–88.
3. Kwon JS, O’Donnell BF, Wallenstein GV, Greene RW, Hirayasu Y, Nestor PG, Hasselmo ME, Potts GF, Shenton ME, McCarley RW. Gamma frequency-range abnormalities to auditory stimulation in schizophrenia. Arch Gen Psychiatry. 1999;56(11):1001–5.
4. Beeman D. A modeling study of cortical waves in primary auditory cortex. BMC Neurosci. 2013;14(Suppl. 1):P23.

## P24 Memory recall and spike frequency adaptation

### James P. Roach^1^, Leonard M. Sander^2,3^, Michal R. Zochowski^2,3,4^

#### ^1^Neuroscience Graduate Program, University of Michigan, Ann Arbor, MI 48109, USA; ^2^Center for the Study of Complex Systems, University of Michigan, Ann Arbor, MI 48109, USA; ^3^Department of Physics, University of Michigan, Ann Arbor, MI 48109, USA; ^4^Biophysics Program, University of Michigan, Ann Arbor, MI 48109, USA

##### **Correspondence:** James P. Roach - roachjp@umich.edu

*BMC Neuroscience* 2016, **17(Suppl 1)**:P24

In the brain, representations of the external world are encoded by patterns of neural activity. It is critical that representations be stable, but still easily moved between. This phenomenon has been modeled at the network level as auto associative memory. In auto associative network models, such as the Hopfield network, representations, or memories, are stored within synaptic weights and form stable fixed points, or attractors [1]. Spike frequency adaptation (SFA) provides a biologically plausible mechanism for switching between stabile fixed points in the Hopfield network. In the present work we show that for low levels of SFA networks will stabilize in a representation that corresponds to the nearest memory activity space, regardless of strength. In networks with higher levels of SFA only the pattern corresponding to the strongest memory, or a global minimum in activity space. The effects of SFA are similar to fast, or thermodynamic noise, but also allows for deterministic destabilization of memories leading to periodic activation of memories through time. We argue that control of SFA level is a universal mechanism for network-wide attractor selectivity. SFA is tightly regulated by the neurotransmitter acetylcholine (ACh) and can be changed on behaviorally relevant timescales. To support this claim we demonstrate that SFA controls selectivity of spatial attractors in a biophysical model of cholinergic modulation in cortical networks [2, 3]. This model produces localized bumps of firing. A region with enhanced recurrent excitation acts as an attractor for the bump location and selectivity for these regions is quickly diminishes as SFA level increases [3]. When multiple spatial attractors of varying strengths are stored in a network moderate increases SFA level will lead to the weak attractors being destabilized and activity localizing within the strongest attractor. This effect is qualitatively similar to the effects of SFA in the Hopfield network. These results indicate that ACh controls memory recall and perception within the cortex by regulation of SFA and explain the important role cholinergic modulation plays in cognitive functions such as attention and memory consolidation [4].

**Acknowledgements:** JPR was supported by an NSF Graduate Research Fel- lowship Program under Grant No. DGE 1256260 and a UM Rackham Merit Fellowship. MRZ and LMS were supported by NSF PoLS 1058034.

**References**

1. Hopfield JJ. Neural networks and physical systems with emergent collective computational abilities. PNAS. 1982;79: 2554–8.
2. Stiefel KM, Gutkin BS, Sejnowski TJ. The effects of cholinergic neuromodulation on neuronal phase-response curves of modeled cortical neurons. J Comp Neurosci. 2008;26:289–301.
3. Roach JP, Ben-Jacob E, Sander LM, Zochowski MR. Formation and dynamics of waves in a cortical model of cholinergic modulation. PLoS Comput Biol. 2015;11(8): e1004449.
4. Hasselmo ME, Sarter M. Modes and models of forebrain cholinergic neuromodulation of cognition. Neuropsychopharmacology. 2011;36:52–73.

## P25 Stability of neural networks and memory consolidation preferentially occur near criticality

### Quinton M. Skilling^1^, Nicolette Ognjanovski^2^, Sara J. Aton^2^, Michal Zochowski^1,3^

#### ^1^Biophysics Program, University of Michigan, Ann Arbor, MI 48109 USA; ^2^Department of Molecular, Cellular, and Developmental Biology, University of Michigan, Ann Arbor, MI, 48109 USA; ^3^Department of Physics, University of Michigan, Ann Arbor, MI 48109 USA

##### **Correspondence:** Michal Zochowski - michalz@umich.edu

*BMC Neuroscience* 2016, **17(Suppl 1)**:P25

Dynamic neural representations underlie cognitive processing and are an outcome of complex interactions of network structural properties and cellular dynamics. We have developed a new framework to study dynamics of network representations during rapid memory formation in the hippocampus in response to contextual fear conditioning (CFC) [1]. Experimentally, this memory paradigm is achieved by exposing mice to foot shocks while in a novel environment and later testing for behavioral responses when reintroduced to that environment. We employ the average minimum distance (AMD) functional connectivity algorithm to spiking data recorded before, during, and after CFC using implanted stereotrodes. Comparing changes in functional connectivity using cosine similarity, we find that stable functional representations correlate well with animal performance in learning. Using extensive computer simulations, we show that the most robust changes compared to baseline occur when the system resides near criticality. We attribute these results to emergence of long-range correlations during the initial process of memory formation. Furthermore, we have developed a generic model using a generalized Hopfield framework to link formation of novel memory representation to functional stability changes. The network initially stores a single representation, which is to exemplify biologically already stored (old) memories, and is then presented a new representation by freezing a randomly chosen fraction of nodes from a novel pattern. We show that imposing fractional input of the new representation may partially stabilize this representation near the phase transition (critical) point. We further show that invoking synaptic plasticity rules may fully stabilize this new representation only when the dynamics of the network reside near criticality. Taken together these results show, for the first time, that only when the network is at criticality can it stabilize novel memory representations, the dynamical regime which also yields an increase of network stability. Furthermore, our results match well experimental data observed from CFC experiments.

**Reference**

1. Ognjanovski N, Maruyama D, Lashner N, Zochowski M, Aton SJ. CA1 hippocampal network activity changes during sleep-dependent memory consolidation. Front Syst Neurosci. 2014;8:61.

## P26 Stochastic oscillation in self-organized critical states of small systems: sensitive resting state in neural systems

### Sheng-Jun Wang^1,2^, Guang Ouyang^2^, Jing Guang^3^, Mingsha Zhang^3^, K. Y. Michael Wong^4^, Changsong Zhou^2,5,6^

#### ^1^Department of Physics, Shaanxi Normal University, Xi’An City, ShaanXi Province, China; ^2^Department of Physics and Centre for Nonlinear Studies, Institute of Computational and Theoretical Studies, Hong Kong Baptist University, Kowloon Tong, Hong Kong; ^3^State Key Laboratory of Cognitive Neuroscience and Learning, Beijing Normal University, Beijing, China; ^4^Department of Physics, Hong Kong University of Science and Technology, Clear Water Bay, Hong Kong; ^5^Beijing Computational Science Research Center, Beijing 100084, People’s Republic of China; ^6^Research Centre, HKBU Institute of Research and Continuing Education, Shenzhen, China

##### **Correspondence:** Changsong Zhou - cszhou@hkbu.edu.hk

*BMC Neuroscience* 2016, **17(Suppl 1)**:P26

Self-organized critical states (SOCs) and stochastic oscillations (SOs) are simultaneously observed in neural systems [1], which appears to be theoretically contradictory since SOCs are characterized by scale-free avalanche sizes but oscillations indicate typical scales. Here, we show that SOs can emerge in SOCs of small size systems due to temporal correlation between large avalanches at the finite-size cutoff, resulting from the accumulation-release process in SOCs. In contrast, the critical branching process without accumulation-release dynamics cannot exhibit oscillations. The reconciliation of SOCs and SOs is demonstrated both in the sandpile model and robustly in biologically plausible neuronal networks. The oscillations can be suppressed if external inputs eliminate the prominent slow accumulation process, providing a potential explanation of the widely studied Berger effect or event-related desynchronization in neural response. The features of neural oscillations and suppression are confirmed during task processing in monkey eye-movement experiments. Our results suggest that finite-size, columnar neural circuits may play an important role in generating neural oscillations around the critical states, potentially enabling functional advantages of both SOCs and oscillations for sensitive response to transient stimuli. The results have been published in [2].

**Acknowledgements:** This work was partially supported by Hong Kong Baptist University Strategic Development Fund, NSFCRGC Joint Research Scheme HKUST/NSFC/12-13/01 (or N-HKUST 606/12), RGC (Grants No. 604512, No. 605813, and No. 12302914), NSFC (Grants No.11275027, No. 11328501, and No. 11305098), and the Fundamental Research Funds for the Central Universities (Grant No. GK201302008).

**References**

1. Gireesh E, Plenz D, Neuronal avalanches organize as nested theta-and beta/gamma-oscillations during development of cortical layer 2/3. Proc Natl Acad Sci USA. 2008;105:7576–81.
2. Wang SJ, Ouyang G, Guang J, Zhang MS, Wong KYM, Zhou CS. Stochastic oscillation in self-organized critical states of small systems: sensitive resting state in neural systems. Phys Rev Lett. 2016;116:018101.

## P27 Neurofield: a C++ library for fast simulation of 2D neural field models

### Peter A. Robinson^1,2^, Paula Sanz-Leon^1,2^, Peter M. Drysdale^1,2^, Felix Fung^1,2^, Romesh G. Abeysuriya^3^, Chris J. Rennie^1,2^, Xuelong Zhao^1,2^

#### ^1^School of Physics, University of Sydney, Sydney, New South Wales, 2006, Australia; ^2^Center for Integrative Brain Function, University of Sydney, Sydney, New South Wales, 2006, Australia

##### **Correspondence:** Paula Sanz-Leon - paula.sanz-leon@sydney.edu.au

*BMC Neuroscience* 2016, **17(Suppl 1)**:P27

Neural field theory [1] has addressed numerous questions regarding brain dynamics and its interactions across many scales, becoming a highly flexible and unified framework for the study and prediction experimental observables of the electrical activity of the brain. These include EEG spectra [2, 3], evoked response potentials, age-related changes to the physiology of the brain [4], epileptic seizures [5, 6], and synaptic plasticity phenomena [7]. However, numerical simulations of neural field models are not widely available despite their extreme usefulness in cases where analytic solutions are less tractable. This work introduces the features of *NeuroField*, a research-ready library applicable to simulate a wide range of neural field based systems involving multiple structures (e.g., cortex, cortex and thalamic nuclei, and basal ganglia). The link between a given neural field model, its mathematical representation (i.e., a delay-partial differential equations system with spatial periodic boundary conditions) and its computational implementation is described. The resulting computational model has the capability to represent from spatially extended to neural-mass-like systems, and it has been extensively validated against analytical solutions and against experiment [1–10]. To illustrate its flexibility, a range of simulations modeling a variety of arousal-, sleep- and epilepsy-state phenomena is presented [8, 9]. *NeuroField* has been written using object-oriented programming in C++ and is bundled together with MATLAB routines for quantitative offline analysis, such as spectral and dynamic spectral analysis.

**References**

1. Robinson PA, Rennie CJ, Wright JJ. Propagation and stability of waves of electrical activity in the cortex. Phys Rev E. 1997;56:826–40.
2. Robinson PA, Rennie CJ, Wright JJ, Bahramali H, Gordon E, Rowe D. Prediction of electroencephalographic spectra from neurophysiology. Phys Rev E. 2001;63:021903.
3. Robinson PA, Rennie CJ, Rowe DL, O’Connor SC. Estimation of multiscale neurophysiologic parameters by electroencephalographic means. Hum Brain Mapp. 2004;23:53–72.
4. van Albada SJ, Kerr CC, Chiang AKI, Rennie CJ, Robinson PA. Neurophysiological changes with age probed by inverse modeling of EEG spectra. Clin Neurophysiol. 2010;121:21–38.
5. Robinson PA, Rennie CJ, Rowe DL. Dynamics of large-scale brain activity in normal arousal states and epileptic seizures. Phys Rev E. 2002; 65:041924.
6. Breakspear M, Roberts JA, Terry JR, Rodrigues S, Mahant N, Robinson PA. A unifying explanation of primary generalized seizures through nonlinear brain modeling and bifurcation analysis. Cereb Cortex. 2006;16:1296–1313.
7. Fung PK, Haber AL, Robinson PA. Neural field theory of large-scale synaptic plasticity in the cerebral cortex. J Theor Biol. 2013; 318:44–57.
8. Abeysuriya RG, Rennie CJ, Robinson PA. Physiologically based arousal state estimation and dynamics. J Neurosci Methods. 2015; 253:55–69.
9. Robinson, PA, Postnova, S, Abeysuriya, RG, Kim, JK, Roberts, JA, McKenzie-Sell L, Karanjai, A, Kerr, CC, Fung, F, Anderson, R, Breakspear, MJ, Drysdale, PM, Fulcher, BD, Phillips, AKJ, Rennie, CJ, Yin G. Chapter 5: a multiscale “working brain” model. In: Validating neurocomputational models of of neurological and psychiatric disorders. Paris: Springer; 2015.
10. O’Connor SC, Robinson PA. Spatially uniform and nonuniform analysis of electroencephalographic dynamics, with application to the topography of the alpha rhythm. Phys Rev E. 2004;70:110–9.

## P28 Action-based grounding: Beyond encoding/decoding in neural code

### Yoonsuck Choe^1^, Huei-Fang Yang^2^

#### ^1^Department of Computer Science & Engineering, Texas A&M University, College Station, TX, 77845, USA; ^2^Research Center for Information Technology Innovation, Academia Sinica, Taipei, Taiwan

##### **Correspondence:** Yoonsuck Choe - choe@tamu.edu

*BMC Neuroscience* 2016, **17(Suppl 1)**:P28

**Fig. 18:**
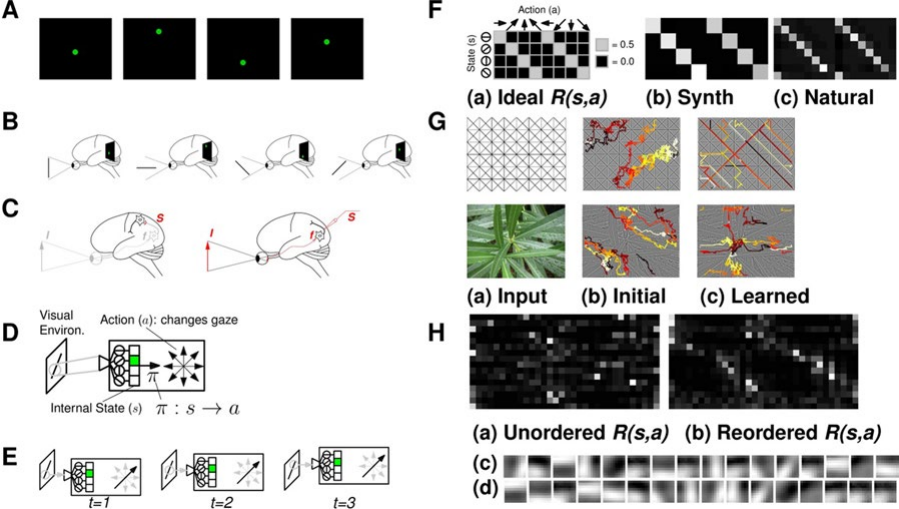
Concept (**A**–**E**) and simulation results (**F**–**H**). **A** Four activities without any clear meaning. **b** Activities in A are V1 response to oriented lines. **C** Comparison of brain’s view of spikes (*left*; apparently intractable) and scientist’s view of spikes (*right*; decoding possible). **D** Visuomotor agent set up. **E** Invariance principle. **F** Ideal state(s)-action(a) mapping *R*(*s*, *a*) (a), learned *R*(*s*, *a*) (b: synthetic input), learned *R*(*s*, *a*) (c: natural input). **G** Input (a), initial gaze trajectory (b), and learned gaze trajectory (c). **H** Learned state-action mapping (a: unordered; b: reordered rows), and learned receptive fields (c: unordered; d: reordered as b) [1]

How can we decode the neural activation patterns (Fig. 18A)? This is a key question in neuroscience. We as scientists have the luxury of controlling the stimulus, based on which we can find the meaning of the spikes (Fig. 18C-right). However, as shown in Fig. 18A (and C-left), the problem seems intractable from the point of view of the brain itself since neurons deeply embedded in the brain do not have direct access to the stimulus. In [1] and related work, we showed that the decoding problem seems intractable only because we left out the motor system from the picture. Figure 18D shows how motor action can help processes deeply embedded in the brain can understand the meaning of the spikes by generating motor behavior and observing the resulting change in the neural spikes. Here, a key principle is to generate motion that keeps the neural spike pattern invariant over time (Fig. 18E), which allows the following to coincide (1) the property of the motion (diagonal movement) and (2) the encoded property of the input (45° orientation). Using reinforcement learning, we showed that the invariance criterion leads to near optimal state-action mapping for synthetic and natural image inputs (Fig. 18F, G), where the encoded property of the input is mapped to congruent motor action. Furthermore, we showed that the receptive fields can be learned simultaneously with the state-action mapping (Fig. 18H). The main lesson we learned is that the encoding/decoding framework in neural code can lead to a dead end unless the problem is posed from the perspective of the brain itself; and the motor system can play an important role in the shaping of the sensory/perceptual primitives (also see [2]).

**References**

1. Choe Y, Yang HF, Misra N. Motor system’s role in grounding, receptive field development, and shape recognition. In: 7th IEEE international conference on development and learning (ICDL 2008). IEEE. p. 67–72.
2. Salinas E. How behavioral constraints may determine optimal sensory representations. PLoS Biol. 2006;4(12):e387.

## P29 Neural computation in a dynamical system with multiple time scales

### Yuanyuan Mi^1,†^, Xiaohan Lin^1,†^, Si Wu^1^

#### ^1^State Key Lab of Cognitive Neuroscience & Learning, IDG/McGovern Institute for Brain Research, Beijing Normal University, Beijing 100875, China

##### **Correspondence:** Si Wu - wusi@bnu.edu.cn

^†^ Y.M. and X.L. contributed equally to this work

*BMC Neuroscience* 2016, **17(Suppl 1)**:P29

The brain performs computation by updating its internal states in response to external inputs. Neurons, synapses, and the circuits are the fundamental units for implementing brain functions. At the single neuron level, a neuron integrates synaptic inputs and generates spikes if its membrane potential crosses the threshold. At the synapse level, neurons interact with each other to enhance or depress their responses. At the network level, the topology of neuronal connection pattern shapes the overall population activity. These fundamental computation units of different levels encompass rich short-term dynamics, for example, spike-frequency adaptation (SFA) at single neurons [1], short-term facilitation (STF) and depression (STD) at neuronal synapses [2]. These dynamical features typically expand a broad range of time scale and exhibit large diversity in different brain regions. Although they play a vital part in the rise of various brain functions, it remains unclear what is the computational benefit for the brain to have such variability in short-term dynamics.

**Fig. 19:**
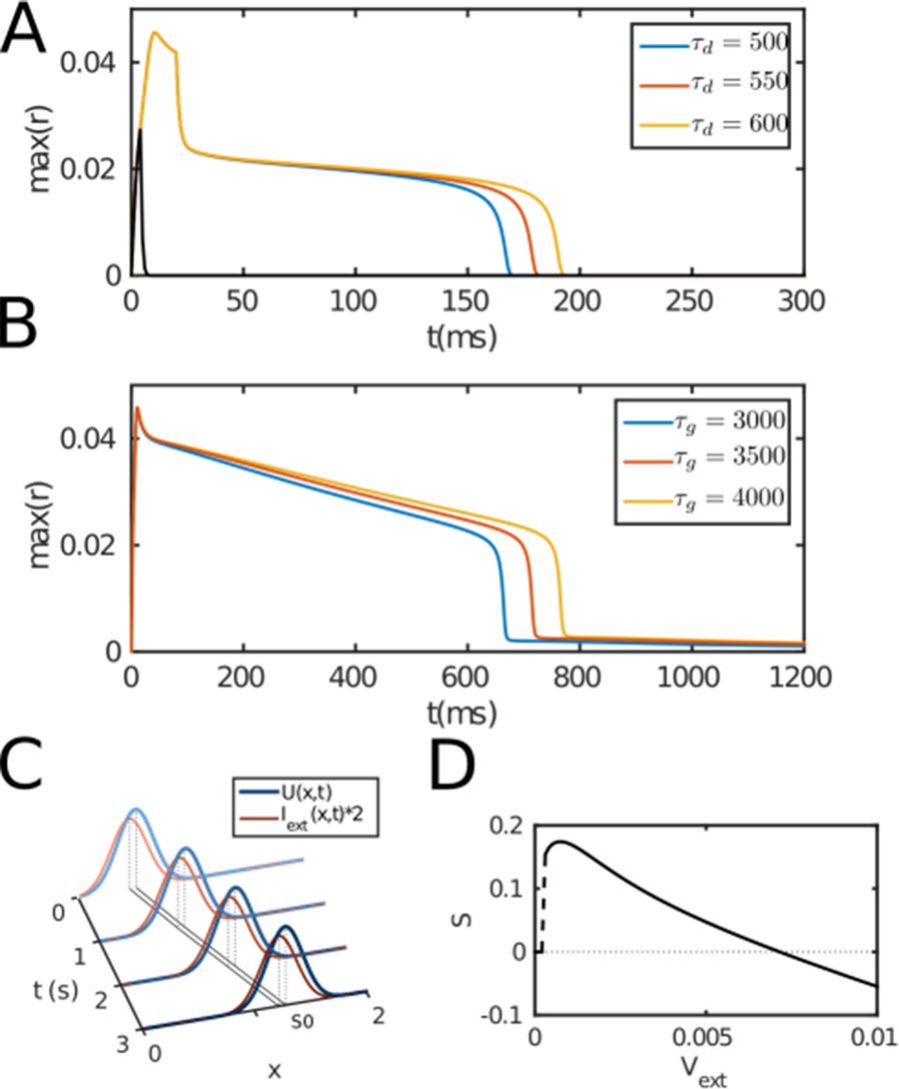
Networks implement different computations. **A** Persistent activity; network can sustain activity after removing stimulus. **B** Adaptation; network activity attenuates to background level given continuous stimulus. **C** Anticipative tracking; **D** network response leads moving stimulus in a certain speed

In this study, we propose that one benefit for having multiple dynamical features with varied time scales is that the brain can fully exploit the advantages of these features to implement which are otherwise contradictory computational tasks. To demonstrate this idea, we consider STF, SFA and STD with increasing time constants in the dynamics of a CANN. The potential brain regions with these parameter values are the sensory cortex, where the neuronal synapses are known to be STD-dominating. We show that the network is able to implement three seemingly contradictory computations, which are persistent activity, adaptation and anticipative tracking (see Fig. 19). Simply state, the role of STF is to hold persistent activity in the absence of external drive, the role of SFA is to support anticipative tracking for a moving input, and the role of STD is to eventually suppress neural activity for a static or transient input. Notably, the time constants of SFA and STD can be swapped with each other, since SFA and STD have the similar effects on the network dynamics. Nevertheless, we need to include both of them, since a single negative feedback modulation is unable to achieve both anticipative tracking and plateau decay concurrently. The implementation of each individual computational task based on a single dynamical feature has been studied previously. Here, our contribution is on revealing that these tasks can be realized concurrently in a single neural circuit by combined dynamical features with coordinated time scales. We hope that this study will shed light on our understanding of how the brain orchestrates its rich dynamics at various levels to realize abundant cognitive functions.

**Reference**

1. Benda J, Herz AVM. A universal model for spike-frequency adaptation. Neural Comput. 2003;15(11):2523–64.
2. Markram H, Wang Y, Tsodyks M. Differential signaling via the same axon of neocortical pyramidal neurons. Proc Natl Acad Sci. 1998;95(9):5323–28.

## P30 Maximum entropy models for 3D layouts of orientation selectivity

### Joscha Liedtke^1,2^, Manuel Schottdorf^1,2^, Fred Wolf^1,2^

#### ^1^Max Planck Institute for Dynamics and Self-Organization, Göttingen, Germany; ^2^Bernstein Center for Computational Neuroscience, Göttingen, Germany

##### **Correspondence:** Joscha Liedtke - joscha@nld.ds.mpg.de, Manuel Schottdorf - manuel@nld.ds.mpg.de

*BMC Neuroscience* 2016, **17(Suppl 1)**:P30

The neocortex is composed of 6 different layers. In the primary visual cortex (V1), the functional architecture of basic stimulus selectivity is experimentally found to be similar across these layers [1]. The organization in functional columns justifies the use of cortical models describing only two-dimensional layers and disregarding functional organization in the third dimension.

**Fig. 20:**
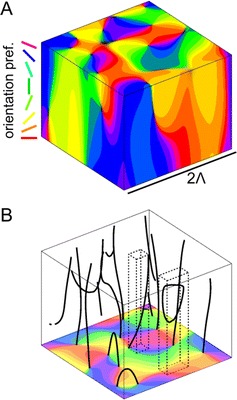
**A** Three-dimensional orientation domains with columnar correlation length of Λ. **B** String singularities of orientation domains in **A**. Typical scale of cats Λ ≈ 1 mm

Here we show theoretically that already small deviations from an exact columnar organization can lead to non-trivial three-dimensional functional structures (see Fig. 20). Previously, two-dimensional orientation domains were modeled by Gaussian random fields, the maximum entropy ensemble, allowing for an exact calculation of pinwheel densities [2]. Pinwheels are points surrounded by neurons preferring all possible orientations and these points generalize to pinwheel strings in three dimensions. We extend the previous two-dimensional model characterized by its typical scale of orientation domains to a three-dimensional model by keeping the typical scale in each layer and introducing a columnar correlation length. We dissect in detail the three-dimensional functional architecture for flat geometries and for curved gyri-like geometries with different columnar correlation lengths. The model is analyzed analytically complemented by numerical simulations to obtain solutions for its intrinsic statistical parameters. We find that (i) pinwheel strings are generally curved, (ii) for large curvatures closed loops and reconnecting pinwheel strings appear and (iii) for small columnar correlation lengths a novel transition to a rodent-like interspersed organization emerges.

This theory extends the work of [2, 3] by adding a columnar dimension and supplements the work of [4] by a rigorous statistical treatment of the three-dimensional functional architecture of V1. Furthermore, the theory sheds light on the required precision of experimental techniques for probing the fine structure of the columnar organization in V1.

**References**

1. Hubel DN, Wiesel TN. Receptive fields, binocular interaction and functional architecture in the cat’s visual cortex. J Physiol. 1962;160:106–54.
2. Schnabel M, Kaschube M, Löwel S, Wolf F. Random waves in the brain: symmetries and defect generation in the visual cortex. Eur Phys J Spec Topics. 2007;145(1):137–57.
3. Wolf F, Geisel T. Spontaneous pinwheel annihilation during visual development. Nature. 1998;395:73–8.
4. Tanaka S, Moon CH, Fukuda M, Kim SG: Three-dimensional visual feature representation in the primary visual cortex. *Neural Networks* 2011, 24(10):1022-1035.

## P31 A behavioral assay for probing computations underlying curiosity in rodents

### Yoriko Yamamura^1^, Jeffery R. Wickens^1^

#### ^1^Neurobiology Research Unit, Okinawa Institute of Science and Technology, Onna-son, Okinawa, 904-0412, Japan

##### **Correspondence:** Yoriko Yamamura - yoriko@oist.jp

*BMC Neuroscience* 2016, **17(Suppl 1)**:P31

Curiosity in humans appears to follow an inverted U-shaped function of unpredictability: stimuli that are neither too predictable nor too unpredictable evoke the greatest interest [1]. Rewarding moderate sensory unpredictability is an effective strategy for reinforcing explorations that improve our predictive models of the world [1, 2]. However, the computations and neural circuits underlying this unpredictability-dependence of curiosity remain largely unknown.

A rodent model of curiosity would be useful for elucidating its underlying neural circuitry, because more specific manipulation techniques are available than in humans. It has been shown that mice prefer unpredictable sounds to predictable ones when the sounds are paired with light [3]. However, frequency of stimulus presentation was a potential confound in this study. Furthermore, a more systematic sampling of stimulus unpredictability is necessary to determine whether a rodent analogue of the U-shaped curve indeed exists.

We have devised an operant conditioning paradigm building on [3], using sensory stimuli as “reward” to quantify the rewardingness of various levels of sensory predictability for rats. Rats (Long Evans, male) are placed in a soundproofed chamber with two nosepoke holes. A combination of sound and light stimuli is presented whenever the rat pokes the active hole; no stimulus is associated with the inactive hole (counterbalanced across subjects).

**Fig. 21:**
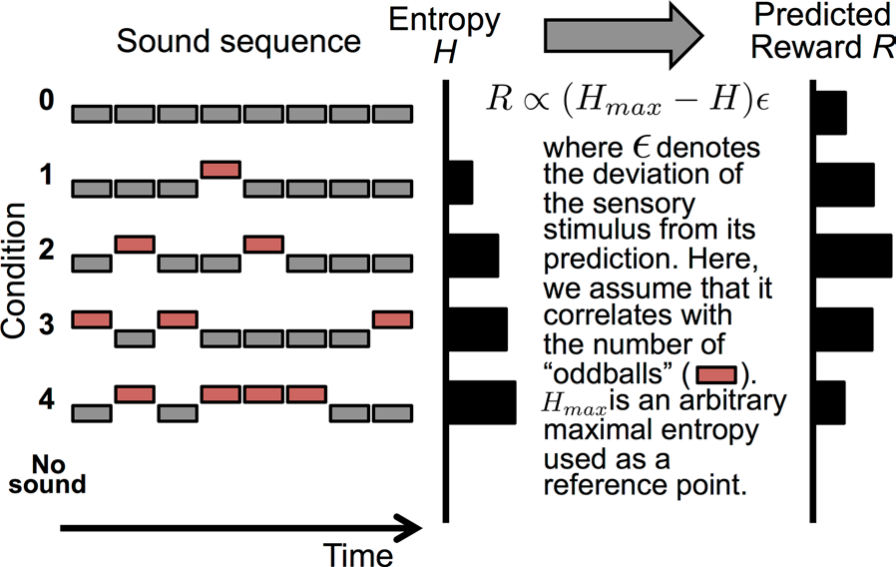
Schematic of the sound stimuli used in all conditions, and the predicted reward for each

We hypothesize that reward is also a U-shaped function of stimulus unpredictability in rats, and that this is due to a Bayesian precision weighting placing more importance on deviations from reliabile predictions. This departs from previous learning-based accounts [2]. There are five experimental conditions, systematically varied in unpredictability of the sound stimuli (as quantified by entropy *H*), and a control condition, in which a nosepoke in neither hole has any consequence (Fig. 21). Specifically, the sound stimuli are random sequences of two possible 125-ms sound snippets of equal value to the rat, with their frequencies of occurrence varied across conditions to vary *H*. Each sequence contains eight such snippets. Across all conditions, the light stimulus simply remains on while the sound is being played; it is added to enhance the rats’ responding to auditory stimuli [3]. We predict that the rats’ active nosepoke responses will be maximally increased at intermediate *H* (Fig. 21).

In preliminary experiments for conditions 0 and 2 (N = 3 each; three sessions), rats preferred the active hole to the inactive, replicating the earlier results in mice [3]. Moreover, as hypothesized, rats responded more to the active hole in condition 2 (mean = 15.0, SD = 5.32) than in condition 0 (mean = 11.3, SD = 4.05); t(22) = 1.91, p = 0.0345 (one-tailed t test). We note that in mice, most across-condition differences did not emerge until around session 7 [3].

The proposed assay quantifies the rewardingness of sensory unpredictability in rats. By systematically varying the entropy of the sound sequence, we can probe the computations behind the putative unpredictability-driven reward. The assay can furthermore be used to study the effect of pharmacological or genetic manipulations on unpredictability-driven reward, in order to validate mechanistic implementations of such computations.

**References**

1. Kidd C, Hayden BY. The psychology and neuroscience of curiosity. Neuron. 2015;88:449–60.
2. Oudeyer PY, Kaplan F. What is intrinsic motivation? A typology of computational approaches. Front Neurorobot. 2007;1:6.
3. Olsen CM, Winder DG. Stimulus dynamics increase the self-administration of compound visual and auditory stimuli. Neurosci Lett. 2012;511:8–11.

## P32 Using statistical sampling to balance error function contributions to optimization of conductance-based models

### Timothy Rumbell^1^, Julia Ramsey^2^, Amy Reyes^2^, Danel Draguljić^2^, Patrick R. Hof^3^, Jennifer Luebke^4^, Christina M. Weaver^2^

#### ^1^Computational Biology Center, IBM Research, Thomas J. Watson Research Center, Yorktown Heights, NY 10598; ^2^Department of Mathematics, Franklin and Marshall College, Lancaster, PA 17604; ^3^Fishberg Department of Neuroscience and Friedman Brain Institute, Icahn School of Medicine at Mount Sinai, New York, NY 10029; ^4^Department of Anatomy and Neurobiology, Boston University School of Medicine, Boston, MA 02118

##### **Correspondence:** Christina M. Weaver - christina.weaver@fandm.edu

*BMC Neuroscience* 2016, **17(Suppl 1)**:P32

**Fig. 22:**
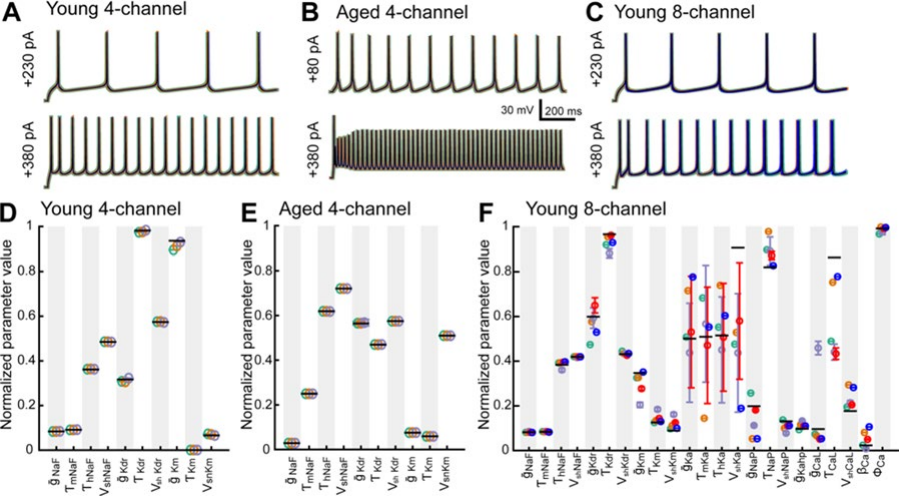
**A**–**C** Membrane potential of the synthetic target (*black*), and of randomly chosen members of the final population (*colors*, overlaid almost exactly), from three validation studies. Optimized 10 and 23 parameters in **A**–**C** respectively. **D–F** Parameter values used to generate synthetic data (black lines), and mean ± standard deviation of values recovered in the searches (colored circles), normalized to the range used in the optimization

Recently we developed a three-stage optimization method for fitting conductance-based models to data [1]. The method makes novel use of Latin hypercube sampling (LHS), a statistical space-filling design, to determine appropriate weights automatically for various error functions that quantify the difference between empirical target and model output. The method uses differential evolution to fit parameters active in the subthreshold and suprathreshold regimes (below and above action potential threshold). We have applied the method to spatially extended models of layer 3 pyramidal neurons from the prefrontal cortex of adult rhesus monkeys, in which in vitro action potential firing rates are significantly higher in aged versus young animals [2]. Here we validate our optimization method by testing its ability to recover parameters used to generate synthetic target data. Results from the validation fit the voltage traces of the synthetic target data almost exactly (Fig. 22A–C), whether fitting a model with 4 ion channels (10 parameters), or 8 ion channels (23 parameters). The optimized parameter values are either identical to, or nearby, the original target values (Fig. 22D–F), except for a few parameters that were not well constrained by the simulated protocols. Further, our LHS-based scheme for weighting error functions is significantly more efficient at recovering target parameter values than by weighting all error functions equally, or by choosing weights manually. We are now using the method to fit models to data from several young, middle-aged, and aged monkeys. Adding new conductances to the model, and allowing altered channel kinetics in the axon initial segment versus the soma, improves the quality of the model fits to data. We use published results from empirical studies of layer 3 neocortical pyramidal neurons to determine whether the optimized parameter sets are biologically plausible.

**References**

1. Rumbell T, Draguljić D, Luebke J, Hof P, Weaver CM. Prediction of ion channel parameter differences between groups of young and aged pyramidal neurons using multi-stage compartmental model optimization. BMC Neurosci. 2015;16(Suppl. 1):P282.
2. Chang YM, Rosene DL, Killiany RJ, Mangiamele LA, Luebke JI. Increased action potential firing rates of layer 2/3 pyramidal cells in the prefrontal cortex are significantly related to cognitive performance in aged monkeys. Cereb Cortex. 2005;15(4):409–18.

## P33 Exploration and implementation of a self-growing and self-organizing neuron network building algorithm

### Hu He^1^, Xu Yang^2^, Hailin Ma^1^, Zhiheng Xu^1^, Yuzhe Wang^1^

#### ^1^Institute of Microelectronics, Tsinghua University, Beijing, 100081, China; ^2^School of Software, Beijing Institute of Technology, Beijing, 100083, China

##### **Correspondence:** Xu Yang - yangxu@tsinghua.edu.cn

*BMC Neuroscience* 2016, **17(Suppl 1)**:P33

In this work, an algorithm to build self-growing and self-organizing neuron network according to external signals is presented, in attempt to build neuron network with high intelligence. This algorithm takes a bionic way to build complex neuron network. We begin with very simple external signals to provoke neurons.

**Fig. 23:**
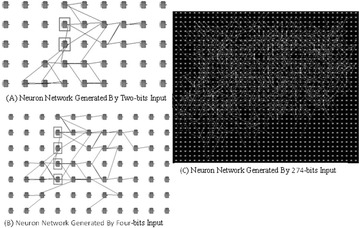
Neuron network generated by our algorithm

In order to propagate the signals, neurons will seek to connect to each other, thus building neuron networks. Those generated networks will be verified and optimized, and be treated as seeds to build more complex networks. Then we repeat this process, use more complex external signals, and build more complex neuron networks. A parallel processing method is presented, to enhance the computation efficiency of the presented algorithm, and to help build large scale of neuron network with reasonable time. The result shows that, neuron network built by our algorithm can self-grow and self-organize as the complexity of the input external signals increase. And with the screening mechanism, neuron network that can identify different input external signals is built successfully (Fig. 23).

**Acknowledgements:** This work is supported by the Core Electronic Devices, High-End General Purpose Processor, and Fundamental System Software of China under Grant No. 2012ZX01034-001-002, the National Natural Science Foundation of China under Grant No. 61502032, Tsinghua National Laboratory for Information Science and Technology (TNList), and Samsung Tsinghua Joint Laboratory.

## P34 Disrupted resting state brain network in obese subjects: a data-driven graph theory analysis

### Kwangyeol Baek^1,2^, Laurel S. Morris^1^, Prantik Kundu^3^, Valerie Voon^1^

#### ^1^Department of Psychiatry, University of Cambridge, Cambridge, CB2 0QQ, United Kingdom; ^2^Department of Biomedical Engineering, Ulsan National Institute of Science and Technology, Ulsan, South Korea; ^3^Departments of Radiology and Psychiatry, Icahn School of Medicine at Mount Sinai, New York City, 10029, USA

##### **Correspondence:** Kwangyeol Baek - kb567@cam.ac.uk

*BMC Neuroscience* 2016, **17(Suppl 1)**:P34

The efficient organization and communication of brain networks underlies cognitive processing, and disruption in resting state brain network has been implicated in various neuropsychiatric conditions including addiction disorder. However, few studies have focused on whole-brain networks in the maladaptive consumption of natural rewards in obesity and binge-eating disorder (BED). Here we use a novel multi-echo resting state functional MRI (rsfMRI) technique along with a data-driven graph theory approach to assess global and regional network characteristics in obesity and BED.

We collected multi-echo rsfMRI scans from 40 obese subjects (including 20 BED patients) and 40 healthy controls, and used multi-echo independent component analysis (ME-ICA) to remove non-BOLD noise. We estimated the normalized correlation across mean rsfMRI signals in 90 brain regions of the Automated Anatomical Labeling atlas, and computed global and regional network metrics in the binarized connectivity matrix with density threshold of 5–25 %. In addition, we confirmed the observed alterations in network metrics using the Harvard-Oxford atlas which was parcellated into 470 even-sized regions.

**Fig. 24:**
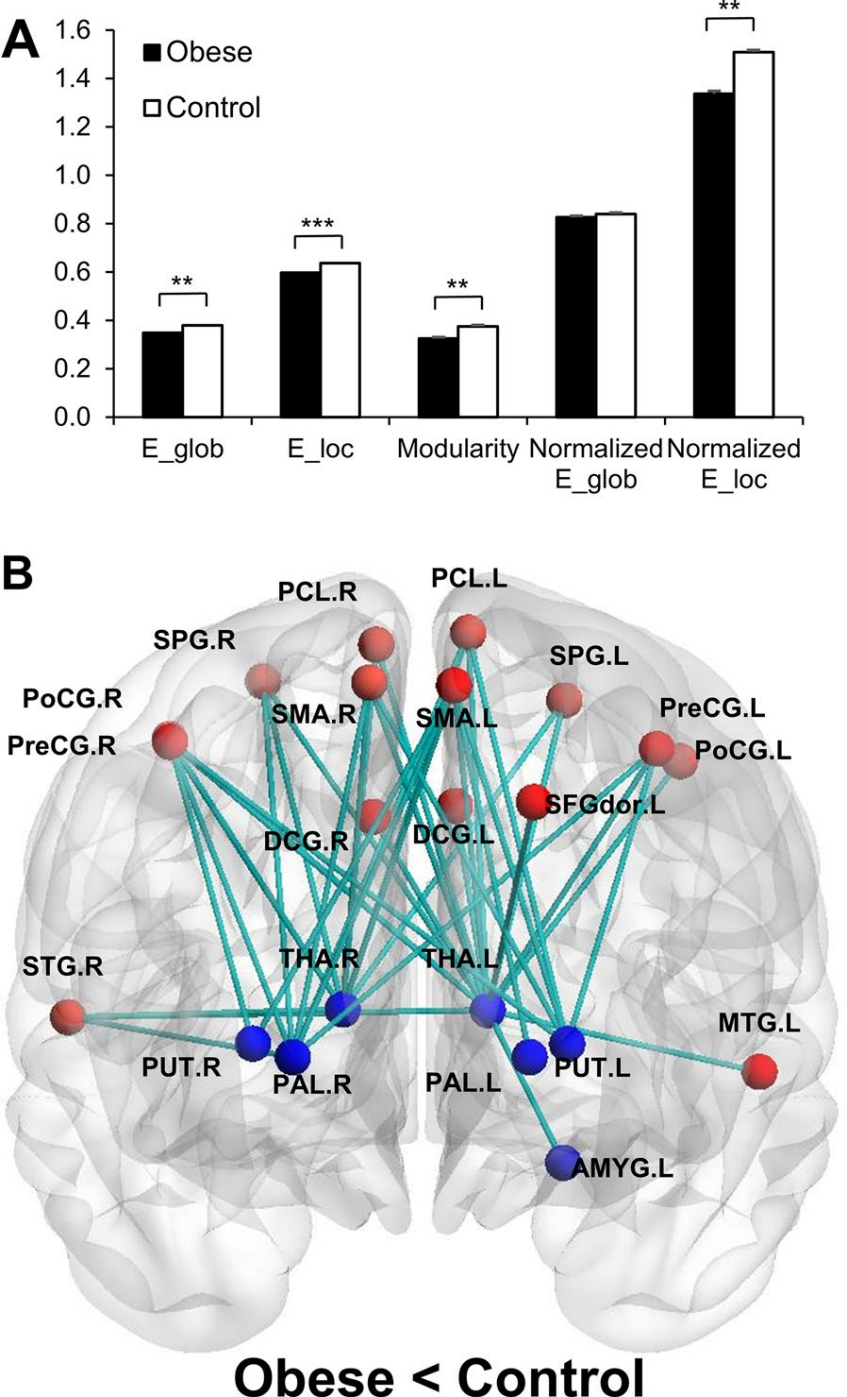
**A** Disrupted resting state brain network in obese subjects. **B** Global network properties network-based statistics

Obese subjects exhibited significantly reduced global and local efficiency as well as decreased modularity in the whole-brain network compared to healthy controls (Fig. 24). Both BED patients and the obese subjects without BED exhibited the same alteration of network metrics compared with healthy controls, but two obese groups did not differ from each other. In regional network metrics, bilateral putamen, thalamus and right pallidum exhibited profoundly decreased nodal degree and efficiency in obese subjects, and left superior frontal gyrus showed decreased nodal betweeness in obese subjects (all p < 0.05, Bonferroni correction). Network-based statistics revealed a cortico-striatal/cortico-thalamic network with significantly decreased functional connectivity which consisted of bilateral putamen, pallidum, thalamus, primary motor cortex, primary somatosensory cortex, supplementary motor area, paracentral lobule, superior parietal lobule, superior temporal cortex and left amygdala. Interestingly, when examining the same network properties but using only single-echo rsfMRI data analysis without ME-ICA, we find no significant differences between groups.

Therefore, using data-driven graph theory analysis of multi-echo rsfMRI data, we highlight more subtle impairments in cortico-striatal/cortico-thalamic networks in obesity that have previously been associated with substance addictions. We emphasize global impairments in network efficiency in obesity with disrupted local network organization closer to random networks. Mathematically capturing brain network alterations in obesity provides novel insights into potential biomarkers and therapeutic targets.

## P35 Dynamics of cooperative excitatory and inhibitory plasticity

### Everton J. Agnes^1^, Tim P. Vogels^1^

#### ^1^Centre for Neural Circuits and Behaviour, University of Oxford, Oxford, OX1 3SR, UK

##### **Correspondence:** Everton J. Agnes - everton.agnes@cncb.ox.ac.uk

*BMC Neuroscience* 2016, **17(Suppl 1)**:P35

**Fig. 25:**
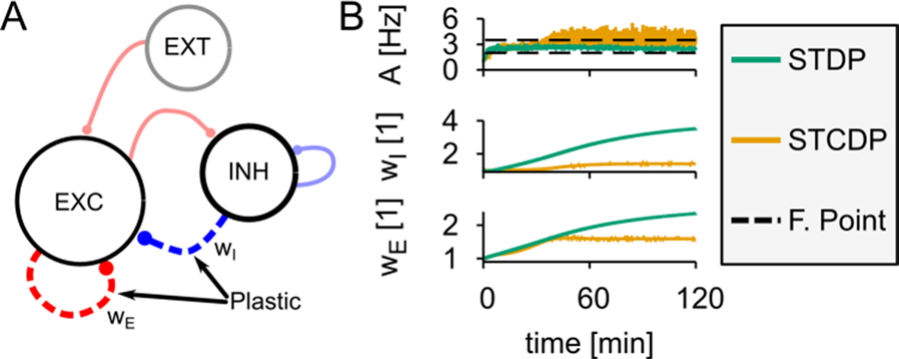
**A** Schematics representing the neuronal network. A group of 2000 excitatory neurons and 500 inhibitory neurons are recurrently connected with sparse connectivity and the excitatory neurons receive random input from an external pool of neurons. **B** Excitatory neurons’ mean firing-rate (*top*), mean excitatory weight onto excitatory neurons (*middle*) and mean inhibitory weight onto excitatory neurons (connections marked as plastic in **A**). Simulation of the neuronal network with a spike-based inhibitory learning rule is represented by *green lines* (STDP) while simulation with our novel spike-timing- and current-dependent learning rule is shown in yellow (STCDP). The *dashed lines* represent the fixed points imposed by the excitatory (*high*) and inhibitory (*low*) learning rules. The low fixed point only exists for the inhibitory STDP model (simulation represented by the *green lines*)

Neurons receive balanced excitatory and inhibitory inputs, a phenomenon thought to be essential for a variety of computations [1–3]. Inhibitory synaptic plasticity is an obvious candidate for imposing this balanced input regime [2,4], leaving excitatory synapses available to learn patterns and memories. Recent experimental work seems to agree with that notion of collaborative excitatory and inhibitory plasticity [4], but recent models do not take direct interactions into consideration. Instead, learning rules are usually tuned to indirectly but constructively interact via the firing-rates they elicit [3,5]. Without proper parameter tuning, this can be problematic because excitatory and inhibitory synaptic plasticity models may have different homeostatic set points, making synaptic weights fluctuate wildly (Fig. 25A, B; *green lines*). Here we present a hybrid model of inhibitory synaptic plasticity that combines the simplicity of spike-based models with the addition of a excitatory/inhibitory input dependence. It captures recent experimental findings showing that changes at inhibitory synapses are strongly correlated with the balance between excitation and inhibition and that inhibitory synapses do not change when excitatory input is blocked [4]. Essentially, our model is a symmetric spike-timing-dependent plasticity (STDP) rule in which the learning-rate is controlled by excitatory and inhibitory activities—a spike-timing- and current-dependent plasticity (STCDP) model. Balance is maintained, but the learning rule does not impose fixed-point attractor dynamics to post-synaptic neurons, because there is no change in inhibitory synapses once the total input is balanced. Inhibitory synapses change depending on excitatory synapses, which means that plasticity depends on at least three synaptic participants (trisynaptic) instead of only two (bisynaptic). We show that when combined with an excitatory synaptic plasticity model, both excitatory and inhibitory weights converge to stable values, as the firing-rate reaches the fixed-point imposed by the excitatory learning rule (Fig. 25B; *yellow lines*). More importantly, the learning rule allows efficient and stable learning of new weights when the balance is disrupted, opening the door for effective and stable learning of arbitrary synaptic patterns.

**Acknowledgements:** This work was partially funded by the Brazilian agency CNPq (Grant Agreement Number 235144/2014-2) and the Sir Henry Dale Fellowship (Grant Agreement WT100000).

**References**

1. Denève S, Machens CK. Efficient codes and balanced networks. Nat Neurosci. 2016;19:375–85.
2. Vogels TP, Froemke RC, Doyon N, Gilson M, Haas JS, Liu R, Maffei A, Miller P, Wierenga CJ, Woodin MA, et al. Inhibitory synaptic plasticity: spike timing-dependence and putative network function. Front Neural Circuits. 2013;7:119.
3. Zenke F, Agnes EJ, Gerstner W. Diverse synaptic plasticity mechanisms orchestrated to form and retrieve memories in spiking neural networks. Nat Commun. 2015;6:6922.
4. D’amour JA, Froemke RC. Inhibitory and excitatory spike-timing-dependent plasticity in the auditory cortex. Neuron. 2015;86:514–28.
5. Sprekeler H, Clopath C, Vogels TP. Interactions of excitatory and inhibitory synaptic plasticity. Front Comp.

## P36 Frequency-dependent oscillatory signal gating in feed-forward networks of integrate-and-fire neurons

### William F. Podlaski^1^, Tim P Vogels^1^

#### ^1^Centre for Neural Circuits and Behaviour, University of Oxford, Oxford, UK

##### **Correspondence:** William F. Podlaski - william.podlaski@cncb.ox.ac.uk

*BMC Neuroscience* 2016, **17(Suppl 1)**:P36

Neural oscillations—the periodic synchronisation of neuronal spiking—is a common feature of brain activity, with several hypothesised functions relating to information flow, attention and brain state [1]. Previous experimental work has shown that oscillatory activity correlates with moments of heightened attention, and that communication between different brain areas is often marked by an increase in oscillatory coherence between the regions [2]. Theoretical and modelling work has helped to explore the mechanisms behind neuronal oscillations, and some of their effects on neural coding and signal propagation [3]. Recently, theoretical studies have explored how resonance might affect signal processing [4, 5] and how information can be propagated along different pathways according to oscillatory phase and frequency [6].

We expand this work here by studying how resonance at the single neuron level might be used for frequency-dependent gating of information flow in neuronal networks. We show that in feed-forward spiking network simulations background oscillations can synchronise or desynchronise the spikes of a propagated signal, changing its content and emphasis from rate code to synfire code or vice versa. Such a mechanism can modulate information flow without rewiring the signal pathways themselves, allowing to select for specific downstream readout targets. Building on this idea, we can create entire pathways that can be selectively (in-)activated by different background oscillatory frequencies without changing the connectivity of the network. We hypothesise that neuronal resonance, combined with resonance in synapses and network motifs, can allow for precise oscillatory gating of information in cortex. Building on previous studies of resonance and oscillatory signal propagation [4,5,6] we propose a plausible mechanism for how fast and precise frequency-dependent gating might be achieved in the brain.

**Acknowledgements:** Research was supported by a Sir Henry Dale Royal Society and Wellcome Trust Research Fellowship (WT100000).

**References**

1. Buzsáki G. Rhythms of the brain. Oxford: Oxford University Press; 2011.
2. Engel AK, Fries P, Singer W. Dynamic predictions: oscillations and synchrony in top-down processing. Nat Rev Neurosci. 2001;2(10):704–16.
3. Wang XJ. Neurophysiological and computational principles of cortical rhythms in cognition. Physiol Rev. 2010;90:1195–1268.
4. Richardson MJE, Brunel N, Hakim V. From subthreshold to firing-rate resonance. J Neurophysiol. 2003;89:2538–54.
5. Hahn G, Bujan AF, Frégnac Y, Aertsen A, Kumar A. Communication through resonance in spiking neuronal networks. PLoS Comput Biol. 2014;10(8):e1003811.
6. Akam T, Kullmann DM. Oscillatory multiplexing of population codes for selective communication in the mammalian brain. Nat Rev Neurosci. 2014;15(2):111–22.

## P37 Phenomenological neural model for adaptation of neurons in area IT

### Martin Giese^1^, Pradeep Kuravi^2^, Rufin Vogels^2^

#### ^1^Section Computational Sensomotorics, CIN & HIH, Department of Cognitive Neurology, University Clinic Tübingen, Germany; ^2^Lab. Neuro en Psychofysiologie, Dept. Neuroscience, KU Leuven, Belgium

##### **Correspondence:** Martin Giese - martin.giese@uni-tuebingen.de

*BMC Neuroscience* 2016, **17(Suppl 1)**:P37

**Fig. 26:**
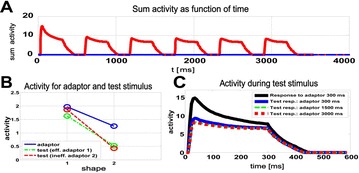
Simulation results. **A** Decay of neural activity for multiple repetitions of the same stimulus. **B** Experiment adapting with effective and ineffective stimuli. **C** Dependence of the PSTH on adaptor duration and unadapted response (*black*)

For repeated stimulation neurons in higher-level visual cortex show adaptation effects. Such effects likely influence repetition suppression paradigms in fMRI studies and the formation of high-level after-effects, e.g. for faces [1]. A variety of theoretical explanations has been discussed, which are difficult to distinguish without detailed electrophysiological data [2]. Meanwhile, detailed physiological experiments on the adaptation of shape-selective neurons in inferotemporal cortex (area IT) have provided constraints that help to narrow down possible neural processes. We propose a neurodynamical model that reproduces a number of these experimental observations by biophysically plausible neural circuits. Our model uses the mean-field limit and consists of a neural field of shape-selective dynamic linear-threshold neurons that are augmented several adaptation processes: (i) spike-rate adaptation; (ii) an input fatigue adaptation process, modeling adaptation in earlier hierarchy levels and of afferent synapses; (iii) a firing-rate fatigue adaptation process that models adaptation dependent on the output firing rates of the neurons. The model with a common parameter set is compared to results from several studies about adaptation in area IT. The model reproduces the following experimentally observed effects: (i) shape of the typical PSTHs of IT neurons; (ii) temporal decay for repeated stimulation of the same neurons with many repetitions of the same stimulus [3] (Fig. 26A); (iii) dependence of adaptation on efficient and ineffective adaptor stimuli, which stimulate the neuron strongly or only moderately [4] (Fig. 26B); (iv) dependence of the strength of the adaptation effect on the duration of the adaptor (Fig. 26C). A mean field model with several additional adaptive processes can account for the observed experimental effects, where all introduced processes were necessary to account for the results. Especially the observed dependence on the effectivity of the adaptor cannot be reproduced without an appropriate mixture if an input fatigue and a firing-rate fatigue mechanism. This suggests that adaptation in IT neurons is significantly influenced by several biophysical processes with different spatial and temporal scales.

**Acknowledgements:** Supported by EC Fp7-PEOPLE-2011-ITN PITN-GA-011-290011 (ABC), FP7-ICT-2013-FET-F/604102 (HBP), FP7-ICT-2013-10/611909 (Koroibot), BMBF, FKZ: 01GQ1002A, DFG GI 305/4-1 + KA 1258/15-1.

**References**

1. Leopold DA, O’Toole AJ, Vetter T, Blanz V: Prototype-referenced shape encoding revealed by high-level aftereffects. Nat Neurosci. 2001;4(1):89–94.
2. Grill-Spector K, Henson R, Martin A. Repetition and the brain: neural models of stimulus-specific effects. Trends Cogn Sci. 2006;10(1):14–23.
3. Sawamura H, Orban GA, Vogels R. Selectivity of neuronal adaptation does not match response selectivity: a single-cell study of the FMRI adaptation paradigm. Neuron. 2006;49(2):307–18.
4. De Baene W, Vogels R. Effects of adaptation on the stimulus selectivity of macaque inferior temporal spiking activity and local field potentials. Cereb Cortex. 2010;20(9):2145–65.

## P38 ICGenealogy: towards a common topology of neuronal ion channel function and genealogy in model and experiment

### Alexander Seeholzer^1,†^, William Podlaski^2,†^, Rajnish Ranjan^3^, Tim Vogels^2^

#### ^1^Laboratory of Computational Neuroscience, EPF Lausanne, Switzerland; ^2^Centre for Neural Circuits and Behaviour, University of Oxford, UK; ^3^The Blue Brain Project, EPF Lausanne, Switzerland

##### **Correspondence:** Alexander Seeholzer - alex.seeholzer@epfl.ch

^†^ These authors contributed equally to this work.

*BMC Neuroscience* 2016, **17(Suppl 1)**:P38

Ion channels are fundamental constituents determining the function of single neurons and neuronal circuits. To understand their complex interactions, the field of computational modeling has proven essential: since its emergence, thousands of ion channel models have been created and published as part of detailed neuronal simulations [1]. Faced with this large variety of models, it is difficult to determine how particular models relate to each other, to the interpretability of simulations and, importantly, to experimental data.

Here, we present a framework within which we analyzed a pilot set of 2378 voltage- or calcium-dependent published ion channel models for the NEURON simulator [1]. We extracted annotated metadata from all associated publications, helping identify their use in simulations (e.g. the animal type, neuron type or area of compartmental models) and the provenance of ion channel models as they were derived from other published work. This categorical and relational metadata is combined with quantitative evaluations of all channel models: individual channels are characterized by their responses to voltage clamp protocols. With subsequent cluster analysis, we extract topologies of ion channel similarity and genealogy, identifying redundancy and groups of common channel kinetics.

**Fig. 27:**
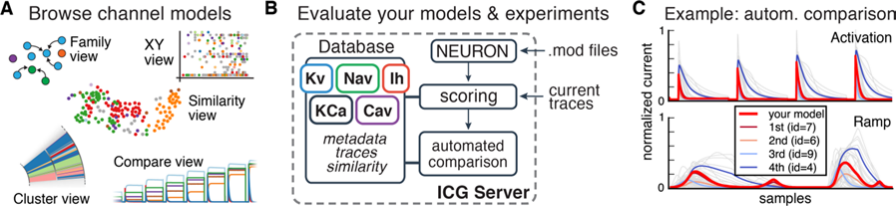
**A** Visualizations available on the web-resource [2] for model browsing. **B** Schematic of upload and evaluation. Both experimental current traces and mod files can be uploaded to our servers, where they are scored and compared to all models currently in the database. **C** Exemplary result of automated comparison: Current traces (recorded from “Ramp” and “Activation” voltage clamp protocols) of the uploaded model (*red*) together with mean (1st, 2nd, 3rd, 4th) and individual (*gray*) traces of the four most similar clusters of channel models in the database

The result of this large-scale assay of published work is freely accessible through interactive visualizations (see Fig. 27A) on the *Ion Channel Genealogy* (*ICG*) web-resource [2], providing a tool for model discovery and comparison. Bridging the gap between model and experiment, our resource allows classifying new channel models and experimental current traces within the topology of all models currently in the database (see Fig. 27B, C). The ICG framework thus allows for quantitative comparison of ion channel kinetics, experimental and model alike, aimed to facilitate field-wide standardization of experimentally-constrained modeling.

**Acknowledgements:** Research was supported by a Sir Henry Dale Royal Society & Wellcome Trust Research Fellowship (WT100000). A.S. was supported by the Swiss National Science Foundation (200020_147200). R.R. was supported by the EPFL Blue Brain Project Fund and the ETH Board funding to the Blue Brain Project.

**References**

1. Hines ML, Morse T, Migliore M, Carnevale NT, Shepherd GM. ModelDB. A database to support computational neuroscience. J Comput Neurosci. 2004;17:7–11.
2. ICGenealogy Project Website. http://icg.neurotheory.ox.ac.uk.

## P39 Temporal input discrimination from the interaction between dynamic synapses and neural subthreshold oscillations

### Joaquin J. Torres^1^, Fabiano Baroni^2^, Roberto Latorre^3^, Pablo Varona^3^

#### ^1^Departamento de Electromagnetismo y Física de la Materia, and Institute “Carlos I” for Theoretical and Computational Physics, University of Granada, Granada, Spain; ^2^School of Psychological Sciences, Faculty of Biomedical and Psychological Sciences, Monash University, Australia; ^3^Grupo de Neurocomputación Biológica, Dpto. de Ingeniería Informática, Escuela Politécnica Superior, Universidad Autónoma de Madrid, Spain

##### **Correspondence:** Pablo Varona - pablo.varona@uam.es

*BMC Neuroscience* 2016, **17(Suppl 1)**:P39

Neuronal subthreshold oscillations underlie key mechanisms of information discrimination in single cells while dynamic synapses provide channel-specific input modulation. Previous studies have shown that intrinsic neuronal properties, in particular subthreshold oscillations, constitute a biophysical mechanism for the emergence of non-trivial single-cell input/output preferences (e.g., preference towards decelerating vs. accelerating input trains of the same average rate) [1, 2]. It has also been shown that short-term synaptic dynamics, in the form of short-term depression and/or short-term facilitation, can provide a channel-specific mechanism for the enhancement of the post-synaptic effects of temporally specific input sequences [3, 4]. While intrinsic oscillations and synaptic dynamics are typically studied independently, it is reasonable to hypothesize that their interplay can lead to more selective and complex temporal input processing.

Here, we extend and refine our previous computational study on the interaction between subthreshold oscillations and synaptic depression [5]. In particular, we investigated whether, and under which conditions, the combination of intrinsic subthreshold oscillations and short-term synaptic dynamics can act synergistically to enable the emergence of robust and channel-specific selectivity in neuronal input–output transformations. We calculated analytically the voltage trajectories and spike output of generalized integrate-and-fire (GIF) model neurons in response to temporally distinct trains of input EPSPs. In particular, we considered triplets of input EPSPs in a range that covers intrinsic and synaptic time scales, and analyzed the model output as intrinsic and synaptic parameters were varied.

Our results show that intrinsic and synaptic dynamics interact in a complex manner for the emergence of specific input–output transformations. In particular, precise non-trivial preferences emerge from synergistic intrinsic and synaptic preferences, while broader selectivity is observed for mismatched intrinsic and synaptic dynamics. We discuss the conditions for robustness of the observed input/output relationships.

We conclude that the interaction of intrinsic and synaptic properties can enable the biophysical implementation of complex and channel-specific mechanisms for the emergence of selective neuronal responses. We further interpret our results in the light of experimental evidence describing distinct short-term synaptic dynamics in different afferents converging onto the same neuron, as in the case of parallel and climbing fiber inputs to cerebellar Purkinje cells, and advance specific hypotheses that link heterogeneous synaptic dynamics of distinct pathways onto the same post-synaptic target to their distinct computational function. We also discuss the impact of single-channel/single-neuron temporal input discrimination in the context of information processing based on heterogeneous elements.

**Acknowledgements:** We acknowledge support from MINECO FIS2013-43201-P, DPI2015-65833-P, TIN-2012-30883 and ONRG Grant N62909-14-1-N279.

**References**

1. Baroni F, Varona P. Subthreshold oscillations and neuronal input–output relationships. Neurocomputing. 2007;70:1611–14.
2. Baroni F, Torres JJ, Varona P. History-dependent excitability as a single-cell substrate of transient memory for information discrimination. PLoS One. 2010;5:e15023.
3. O’Donnell C, Nolan MF. Tuning of synaptic responses: An organizing principle for optimization of neural circuits. Trends Neurosci. 2011;34:51–60.
4. Torres JJ, Kappen HJ. Emerging phenomena in neural networks with dynamic synapses and their computational implications. Front Comp Neurosci. 2013;7.
5. Latorre R, Torres JJ, Varona P. Interplay between subthreshold oscillations and depressing synapses in single neurons. PLoS One. 2016;11:e0145830.

## P40 Different roles for transient and sustained activity during active visual processing

### Bart Gips^1,†^, Eric Lowet^1,2,†^, Mark J Roberts^1,2^, Peter de Weerd^2^, Ole Jensen^1^, Jan van der Eerden^1^

#### ^1^Radboud University, Donders Institute for Brain, Cognition and Behaviour, 6525 EN Nijmegen, The Netherlands; ^2^Faculty of Psychology and Neuroscience, Maastricht University, 6200 MD Maastricht, the Netherlands

##### **Correspondence:** Bart Gips - bart.gips@donders.ru.nl

† Authors have made equal contribution

*BMC Neuroscience* 2016, **17(Suppl 1)**:P40

**Fig. 28:**
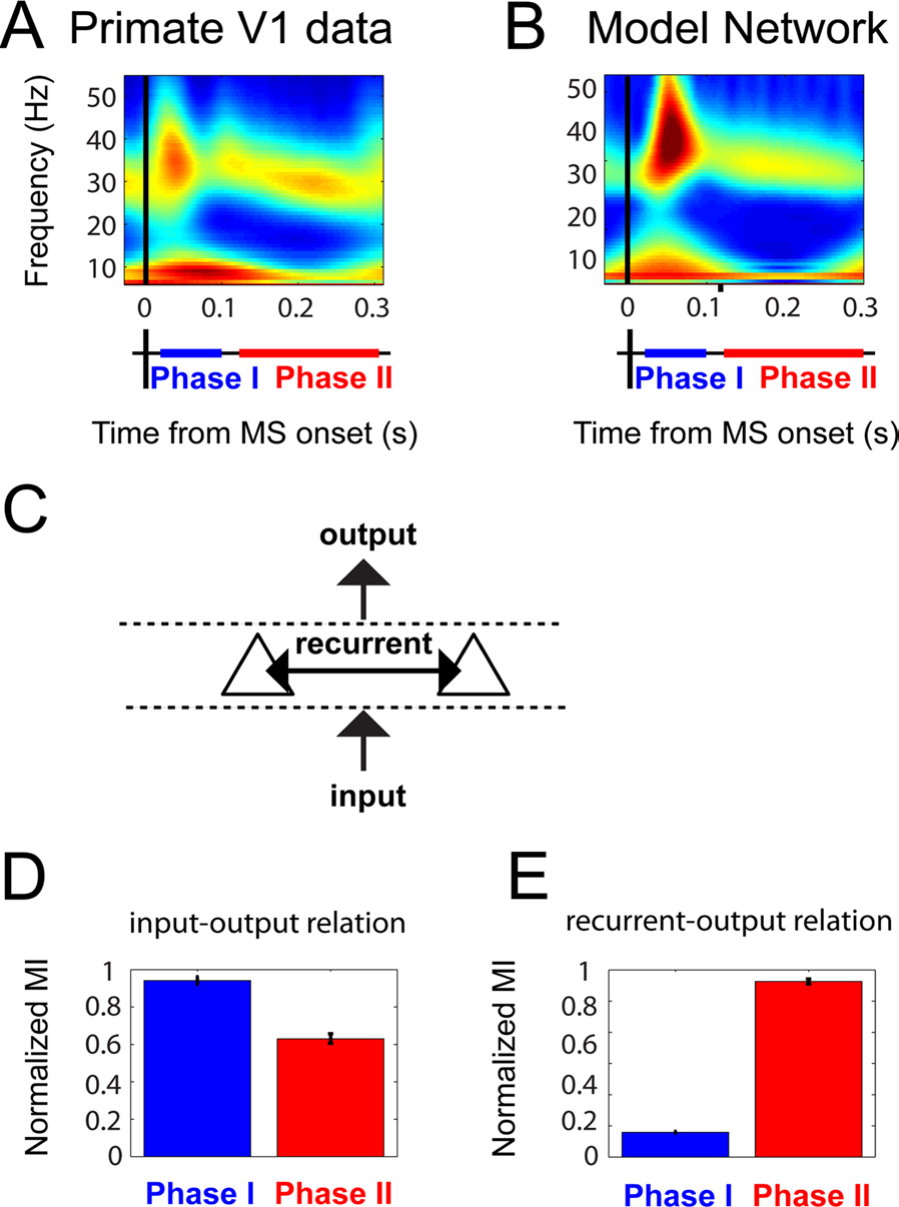
**A** Time–frequency representation of local field potential (LFP) locked to a microsaccade (MS) recorded in primate V1. **B** Time–frequency representation of simulated LFP. **C** Schematic representation of the model network illustrating input (injection current), recurrent connection pattern and output (spike trains). **D** The input to the neurons is best reflected in the simulated spike trains (output) during phase I, quantified by mutual information (MI). **E** Recurrent connection pattern is best reflected in the output during phase II

Neural activity in awake primate early visual cortex exhibits transients with intervals of 250-300 ms. Experimental work by us and others has shown that these transients are related to microsaccadic eye movements [1, 2]. These short transients are followed by periods of steady activity that last until the next microsaccade (Fig. 28A).

We found that computational models of excitatory-inhibitory spiking networks organized in a structure of columns and hypercolumns, are able to represent relevant stimulus information when subjected to 3–4 Hz saccade-like transients. The simulated networks expressed evoked responses with power in the alpha–beta band (~8–25 Hz) as well as gamma rhythmic activity (~25–80 Hz) similar to in vivo local field recordings in monkey V1 (Fig. 28A, B).

We show that in phase I, the model produces large-scale spatial synchrony and pronounced alpha–beta power. In phase II the model exhibits narrow-band gamma oscillations with spatially local synchrony. The activity in the model network (rate and timing coding) in phase I mainly reflects feedforward input (Fig. 28C, D), whereas, the network activity in phase II was dominated by recurrent connections (Fig. 28C, E).

The model network activity closely matches that found in experiments. The simulation results suggest that transient phase (phase I) allows for resetting the network and rapid feedfoward processing of novel information, whereas detailed processing and contextualization by recurrent activity take place in the period of steady gamma activity (phase II). Therefore we arrived at hypotheses on the functional interpretation of phases I and II that can be possibly tested in an experimental setup. First, because of the reset of network activity by a microsaccade, phase I is the optimal time window to switch information flow among competing networks through a top-down signal. This indicates that signals related to visual attention are most likely to occur just after a saccade. Second, the increased efficacy of recurrent connections during phase II indicate that contextualization operations such as figure-ground segregation [3] and contour completion occur in the steady phase ~100 ms after the onset of a (micro)saccade.

**References**

1. Lowet E, Roberts MJ, Bosman CA, Fries P, de Weerd P. Areas V1 and V2 show microsaccade-related 3–4 Hz covariation in gamma power and frequency. Eur J Neurosci. 2015.
2. Martinez-Conde S, Otero-Millan J, Macknik SL. The impact of microsaccades on vision: towards a unified theory of saccadic function. Nat Rev Neurosci. 2013;14:83–96.
3. Self MW, van Kerkoerle T, Supèr H, Roelfsema PR. Distinct roles of the cortical layers of area V1 in figure-ground segregation. Curr Biol. 2013:1–9.

## P41 Scale-free functional networks of 2D Ising model are highly robust against structural defects: neuroscience implications

### Abdorreza Goodarzinick^1^, Mohammad D. Niry^1,2^, Alireza Valizadeh^1,3^

#### ^1^Department of Physics, Institute for Advanced Studies in Basic Sciences (IASBS), Zanjan 45137-66731, Iran; ^2^Center for Research in Climate Change and Global Warming (CRCC), Institute for Advanced Studies in Basic Sciences (IASBS), Zanjan 45137-66731, Iran; ^3^School of Cognitive Sciences, Institute for Research in Fundamental Sciences (IPM), Tehran - Iran

##### **Correspondence:** Abdorreza Goodarzinick - a.goodarzinick@iasbs.ac.ir

*BMC Neuroscience* 2016, **17(Suppl 1)**:P41

In recent years, several experimental observations have confirmed the emergence of self-organized criticality (SOC) in the brain at different scales [1]. At large scale, functional brain networks obtained from fMRI data have shown that node-degree distributions and probability of finding a link versus distance are indicative of scale-free and small-world networks regardless of the tasks in which the subjects were involved [2]. At small scale, the study of neuronal avalanches in networks of living neurons revealed power-law behavior in both spatial and temporal scales [3]. It is also shown that functional networks of the brain are strikingly similar to those derived from the 2D Ising model at critical temperature [4] and the 2D abelian sandpile model [5].

**Fig. 29:**
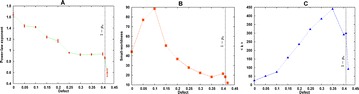
Relevant parameters of functional network of 2D Ising model at critical point versus fraction of defect to the structural cells. **A** Power-law exponent of degree-distribution, **B** small-worldness measure, **C** average degree

The importance to see whether brain network’s scaling properties associated with healthy conditions are altered under various pathologies and how structural defects of a system at criticality can affect its functional connectivity motivated us to study robustness of functional networks of 2D Ising model at critical point against elimination of structural sites. The results showed that the statistics of the functional network indicative of criticality (evident in healthy brain controls), such as power-law behavior and small-worldness remained robust against random elimination of structural sites up to percolation limit (see Fig. 29). The resulting functional network maintained its key properties orders of magnitude higher than those of the same system poised in a super-critical or sub-critical state. These results can show that self-organized critical behavior, besides having unique advantages like fasciliation of alteration of functional patterns, optimization of information transfer and maximization of correlation length, shows striking robustness against structural deficits. Taking into account brain’s long-range anatomical connections and compensatory mechanisms like neuroplasticity, if the results of this study are generalizable to the brain, they may help to explain the delay in clinical diagnosis of multiple neurodegenerative diseases in which possible deficit in functional connectivity among brain regions contribute to the cognitive dysfunctions.

**References**

1. Chialvo DR. Emergent complex neural dynamics. Nat Phys. 2010;6:744–50.
2. Eguíluz VM, Chialvo DR, Cecchi GA, Baliki M, Apkarian AV. Scale-free brain functional networks. Phys Rev Let.t 2005;94:018102.
3. Beggs J, Plenz D. Neuronal avalanches in neocortical circuits. J Neurosci. 2003;23:11167–77.
4. Fraiman D, Balenzuela P, Foss J, Chialvo D. Ising-like dynamics in large-scale functional brain networks. Phys Rev E. 2009;79:061922.
5. Zarepour M, Niry MD, Valizadeh A. Functional scale-free networks in the two-dimensional Abelian sandpile model. Phys Rev E. 2015;92:012822.

## P42 High frequency neuron can facilitate propagation of signal in neural networks

### Aref Pariz^1^, Shervin S Parsi^1^, Alireza Valizadeh^1,2^

#### ^1^Department of Physics, Institute for advanced studies in basic sciences, Zanjan, Iran; ^2^School of Cognitive Sciences, Institute for Studies in Theoretical Physics and Mathematics, Niavaran, Tehran, Iran

##### **Correspondence:** Aref Pariz - a.pariz@iasbs.ac.ir

*BMC Neuroscience* 2016, **17(Suppl 1)**:P42

Signal transmission is of interest from both fundamental and clinical perspective and has been well studied in nonlinear science and complex networks [1, 2]. In particular, in nervous systems, cognitive processing involves signal propagation through multiple brain regions and the activation of large numbers of specific neurons [3–6]. In information propagation through brain regions, each part, known as generator, activated locally as information comes to it from neighboring generators. Although the problem is well studied in the context of complex networks, our focus here is on the effect of the intrinsic dynamical properties of the reciprocal generators on the propagation of signal.

**Fig. 30:**
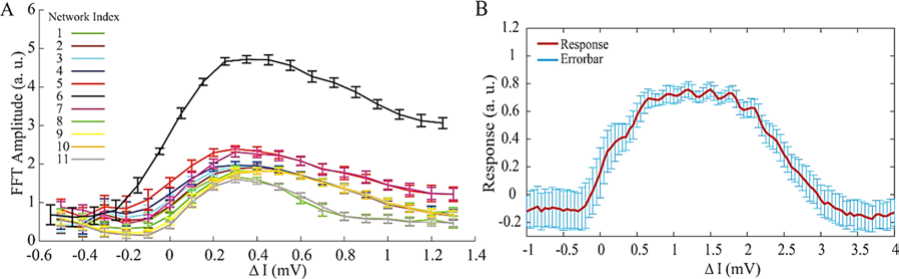
Inhomogeneity of input current on host network, increases the response of network. **A**, **B** Response of networks of neurons and chain of neurons, for different inhomogeneity on host network and host neuron, respectively

In this study we explored the propagation of information in a chain of neurons and networks. As signal propagate through the chain of networks, the firing rate of networks show a fluctuation as host network (the network which receive signal). Here the response is the amplitude of fast Fourier transform of firing rates of each network. If the host network has sufficiently higher intrinsic firing rate than others, signal can transfer with higher amplitude, otherwise, other networks will not get affected. As a result of propagation of signal, for the former case, all networks will show a peak in frequency domain at exactly the same frequency as input signal (Fig. 30A), but with different amplitude which show the efficacy of transmitted information. Also the same result can obtain by a chain of single LIF neurons (Fig. 30B). As phase response curve of the chain and it response to signal show, if the host neuron has higher firing rate (call it leader neuron), the propagation of information will be enhanced. But this higher firing rate has a limit which after that the whole chain will act asynchronously and results the loss of information was aimed to propagate.

**References**

1. Liang X, Liu Z, Li B. Weak signal transmission in complex networks and its application in detecting connectivity. Phys Rev E. 2010;80:046102.
2. Perc M. Stochastic resonance on weakly paced scale-free networks. Phys Rev E. 2008;78:036105.
3. Abeles M. Corticonics: neural circuits of the cerebral cortex. Cambridge: Cambridge UP; 1991.
4. Aertsen A, Diesmann M, Gewaltig MO. Propagation of synchronous spiking activity in feedforward neural networks. J Physiol. 1996;90:243–247.
5. van Rossum MC, Turrigiano GG, Nelson SB. Fast propagation of firing rates through layered networks of noisy neurons. J Neurosci. 2002;22:1956–66.
6. Vogels TP, Abbott LF. Signal propagation and logic gating in networks of integrate-and-fire neurons. J Neurosci. 2005;25(46):10786–95.

## P43 Investigating the effect of Alzheimer’s disease related amyloidopathy on gamma oscillations in the CA1 region of the hippocampus

### Julia M. Warburton^1^, Lucia Marucci^2^, Francesco Tamagnini^3,4^, Jon Brown^3,4^, Krasimira Tsaneva-Atanasova^5^

#### ^1^Bristol Centre for Complexity Sciences, University of Bristol, Bristol, BS8 1TR, UK; ^2^Department of Engineering Mathematics, University of Bristol, Bristol, BS8 1UB, UK; ^3^School of Physiology and Pharmacology, University of Bristol, Bristol, BS8 1TD, UK; ^4^Medical School, University of Exeter, Exeter, EX4 4PE, UK; ^5^Department of Mathematics, University of Exeter, Exeter, EX4 4QF, UK

##### **Correspondence:** Julia M. Warburton - julia.warburton@bristol.ac.uk

*BMC Neuroscience* 2016, **17(Suppl 1)**:P43

Alzheimer’s disease (AD) is the main form of dementia and is characterised clinically by cognitive decline and impairments to memory function. One of the key histopathological features of AD thought to cause this neurodegeneration is the abnormal aggregation of the protein amyloid-β (Aβ) [1]. Transgenic mouse models that overexpress Aβ are used to investigate the potential functional consequences of this amyloidopathy in AD. In this study we use in vitro electrophysiology data recorded from PDAPP transgenic mice (a mouse model of amyloidopathy) and their wild-type littermates to parameterise a hippocampal network model [2]. The aim of the study is to investigate how amyloidopathy alters gamma frequency oscillations within the hippocampus, which is one of the regions first affected in AD.

We use a synaptically connected network of excitatory pyramidal neurons and inhibitory interneurons to simulate the gamma frequency activity [3]. Each cell is described by a single-compartment Hodgkin–Huxley type equation, with the properties of the voltage-gated channels fit to the intrinsic properties measured experimentally, which included stimulated firing frequency data and the associated action potentials from CA1 pyramidal neurons and three-types of CA1 interneuron. Network activity is either driven deterministically via a direct stimulus, such as a step pulse or a theta wave, or via a stochastic input. We perform power spectral density analysis to analyse the oscillatory activity.

Our model focuses on gamma frequency oscillations, which lie in the 30–100 Hz range, because of the associations with attention, sensory processing and potentially of most relevance to AD, with learning and memory. It has been shown that within the hippocampus gamma oscillations enable cross-talk between distributed cell assemblies, with low frequency gamma associated with coupling between the CA1 and the CA3 region and fast frequency gamma associated with coupling between the CA1 and the medial entorhinal cortex [4]. EEG measurements from AD mouse models have identified network hypersynchrony alongside decreased gamma activity, with the role of interneurons in this process highlighted. [5]. By incorporating the pyramidal neuron and interneuron data in our model we aim to learn more about which parameters are most significant in these effects and to further understanding of the effects of amyloidopathy on oscillatory activity.

**Acknowledgements:** This work was supported by funding from the EPSRC.

**References**

1. Hardy J, Selkoe DJ. The amyloid hypothesis of Alzheimer’s disease: progress and problems on the road to therapeutics. Science. 2002;297:353–6.
2. Kerrigan TL, Brown JT, Randall TL. Characterization of altered intrinsic excitability in hippocampal CA1 pyramidal cells of the Aβ-overproducing PDAPP mouse. Neuropharmacology. 2014;79:515–24.
3. Kopell NJ, Borgers C, Pervouchine D, Maerba P, Tort A. Gamma and theta rhythms in biophysical models of hippocampal circuits. In: Hippocampal microcircuits: a computational modeler’s resource book, chap 15; p. 423–57.
4. Colgin LL, Denninger T, Fyhn M, Hafting T, Bonnevie T, Jensen O, Moser M-B, Moser EI. Frequency of gamma oscillations routes flow of information in the hippocampus. Nature. 2009;462:353–7.
5. Verret L, et al. Inhibitory interneuron deficit links altered network activity and cognitive dysfunction in Alzheimer model. Cell. 2012;149:708–21.

## P44 Long-tailed distributions of inhibitory and excitatory weights in a balanced network with eSTDP and iSTDP

### Florence I. Kleberg^1^, Jochen Triesch^1^

#### ^1^Frankfurt Institute for Advanced Studies, Frankfurt am Main, Hessen, Germany, 60438

##### **Correspondence:** Florence I. Kleberg - kleberg@fias.uni-frankfurt.de

*BMC Neuroscience* 2016, **17(Suppl 1)**:P44

The strengths of excitatory synapses in cortex and hippocampus have been shown to follow a rightward-skewed or long-tailed distribution [1,2]. Such distributions can be achieved in recurrent balanced networks [3, 4], after synaptic modification by spike-timing dependent plasticity (STDP) [5] and synaptic scaling [6]. Recently, long-tailed distributions have also been observed for inhibitory synapses in cultured cortical neurons [7], confirming early findings in hippocampal slices [8]. However, the conditions and plasticity mechanisms necessary for achieving long-tailed distributions of inhibitory synapses are unknown. Furthermore, different forms of inhibitory STDP have been reported, but their effect on the distribution of inhibitory synaptic efficacies are largely unknown [9-11].

Here we investigate how plasticity in the inhibitory synapses in a self-organised recurrent neural network (SORN [12]) with leaky integrate-and-fire neurons can lead to long-tailed distributions of synaptic weights. We examine different inhibitory STDP (iSTDP) rules and characterize the conditions under which right-skewed shapes of inhibitory synaptic weight distributions are obtained while a balance between excitation and inhibition is maintained. While the ratio of long-term potentiation to long-term depression in iSTDP affects the shape of the distribution, a variety of window shapes for iSTDP can each achieve long-tailed distributions of inhibitory weights. We find that a precise balance of excitation and inhibition can be achieved with a strongly right-skewed distribution of inhibitory weights. Our results suggest that long-tailed distributions of inhibitory weights could be a ubiquitous feature of neural circuits that employ different plasticity mechanism.

**References**

1. Bekkers, JM, Stevens, CF. NMDA and non-NMDA receptors are co-localized at individual excitatory synapses in cultured rat hippocampus. Nature. 1989;341:230–3.
2. Loewenstein Y, Kuras A, Rumpel S. Multiplicative dynamics underlie the emergence of the log-normal distribution of spine sizes in the neocortex in vivo. J Neurosci. 2011;31(26):9481–8.
3. Effenberger F, Jost J, Levina A. Self-organization in balanced state networks by STDP and homeostatic plasticity. PLoS Comput Biol. 2015;11(9):e1004420.
4. Miner D, Triesch J. Plasticity-driven self-organization under topological constraints accounts for non-random features of cortical synaptic wiring. PLoS Comput Biol. 2016;12(2):e1004759.
5. Bi G, Poo M. Synaptic modifications in cultured hippocampal neurons: dependence on spike timing, synaptic strength, and postsynaptic cell type. J Neurosci. 1998;18(24):10464–72.
6. Turrigiano GG, Leslie KR, Desai NS, Rutherford LC, Nelson SB. Activity-dependent scaling of quantal amplitude in neocortical neurons. Nature. 1998;391(6670):892–6.
7. Rubinski A, Ziv NE. Remodeling and tenacity of inhibitory synapses: relationships with network activity and neighboring excitatory synapses. PLoS Comput Biol. 2015;11(11):e1004632.
8. Miles R. Variation in strength of inhibitory synapses in the CA3 region of guinea-pig hippocampus in vitro. J Physiol. 1990;431:659–76.
9. Woodin MA, Ganguly K, Poo M. Coincident pre-and postsynaptic activity modifies GABAergic synapses by postsynaptic changes in Cl− transporter activity. Neuron. 2003;39(5):807–20.
10. Haas JS, Nowotny T, Abarbanel HDI. Spike-timing-dependent plasticity of inhibitory synapses in the entorhinal cortex. J Neurophysiol. 2006;96(6):3305–13.
11. D’Amour JA, Froemke RC. Inhibitory and excitatory spike-timing-dependent plasticity in the auditory cortex. Neuron. 2015;86(2):514–28.
12. Lazar A, Pipa G, Triesch J. SORN: a self-organizing recurrent neural network. Front Comp Neurosci. 2009;3:23.

## P45 Simulation of EMG recording from hand muscle due to TMS of motor cortex

### Bahar Moezzi^1^, Nicolangelo Iannella^1,4^, Natalie Schaworonkow^2^, Lukas Plogmacher^2^, Mitchell R. Goldsworthy^3^, Brenton Hordacre^3^, Mark D. McDonnell^1^, Michael C. Ridding^3^, Jochen Triesch^2^

#### ^1^Computational and Theoretical Neuroscience Laboratory, School of Information Technology and Mathematical Sciences, University of South Australia, Australia; ^2^Frankfurt Institute for Advanced Studies, Goethe-Universität, Germany; ^3^Robinson Research Institute, School of Medicine, University of Adelaide, Australia; ^4^School of Mathematical Sciences, University of Nottingham, UK

##### **Correspondence:** Bahar Moezzi - bahar.moezzi@unisa.edu.au

*BMC Neuroscience* 2016, **17(Suppl 1)**:P45

Single pulse transcranial magnetic stimulation (TMS) is a technique which (at moderate intensities) activates corticomotor neuronal output cells transynaptically and evokes a complex descending volley in the corticospinal tract. Rusu et al. developed a computational model of TMS induced I-waves that reproduced observed epidural recordings in conscious humans [1]. In humans, epidural responses can be recorded in anaesthetized subjects during surgery or conscious subjects with electrodes implanted for the treatment of chronic pain. Such opportunities are uncommon and invasive. The effects of TMS can be non-invasively studied using surface electromyography (EMG) recordings from the hand first dorsal interosseous (FDI) muscle.

We simulated the surface EMG signal due to TMS of motor cortex in the hand FDI muscle. Our model comprises a population of cortical layer 2/3 cells, which drive layer 5 cortico-motoneuronal cells with excitatory and inhibitory synaptic inputs as in [1]. The layer 5 cells in turn project to a pool of motoneurons, which are modeled as an inhomogeneous population of integrate-and-fire neurons to simulate motor unit recruitment and rate coding. The input to motoneurons from cortical layer 5 consists of TMS-induced spikes and baseline firing. We modeled baseline firing with a Poisson drive to layer 2/3 cells. Hermite-Rodriguez functions were used to simulate motor unit action potential shape. The EMG signal was obtained from the summation of motor unit action potentials of active motor units. Parameters were tuned to simulate recordings from the FDI muscle.

**Fig. 31:**
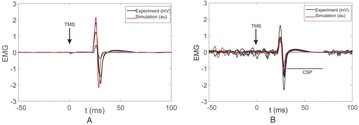
Comparison of simulated and experimental EMG during **A** rest, **B** 10 % maximum voluntary contraction

Our simulated EMG signals match experimental surface EMG recordings due to TMS of motor cortex in the hand FDI muscle in shape, size and time scale both at rest and during voluntary contraction (see Fig. 31). The simulated EMG traces exhibit cortical silent periods (CSP) that lie within the biological range.

**Reference**

1. Rusu CV, Murakami M, Ziemann U, Triesch J. A model of TMS-induced I-waves in motor cortex. Brain Stimul. 2014;7(3):401–14.

## P46 Structure and dynamics of axon network formed in primary cell culture

### Martin Zapotocky^1,2^, Daniel Smit^1,2,3^, Coralie Fouquet^3^, Alain Trembleau^3^

#### ^1^Institute of Physiology of the Czech Academy of Sciences, Prague, Czech Republic; ^2^Institute of Biophysics and Informatics, First Faculty of Medicine, Charles University in Prague, Czech Republic; ^3^IBPS, Neuroscience Paris Seine, CNRS UMR8246, Inserm U1130, UPMC UM 119, Université Pierre et Marie Curie, Paris, France

##### **Correspondence:** Martin Zapotocky - zapotocky@biomed.cas.cz

*BMC Neuroscience* 2016, **17(Suppl 1)**:P46

Axons growing in vivo or in culture may adhere to each other and form a connected network, which subsequently guides the paths of newly arriving axons. We investigated the development of such a network formed by growing axons in primary cell culture.

Olfactory epithelium explants from mouse embryos (day 13–14) were cultured on laminin substrate for 2 days and then recorded using DIC or phase contrast videomicroscopy for up to 24 h. The growing axons established a dense network within which large fascicles of axons were progressively formed. Within the recorded time period, the network remained stable, with limited further gowth of the axons but with ongoing rearrangement in the network structure. Based on segmentation of the recorded images, we determined the principal network characteristics (including the total length, the total number of vertices, and the network anisotropy) and their evolution in time.

This quantitative characterization permitted an analysis of the mechanisms of the observed network coarsening. We relate the network dynamics to the elementary processes of zippering, during which two axons or axon fascicles progressively adhere to each other [1]. We compare the structural features of the network (such as the distribution of vertex angles) with those reported in an electron microscopy investigation of a plexus of sensory neurites in Xenopus embryo [2]. We show that both our ex vivo study and the in vivo study of Ref. [2] support a similar underlying mechanism of the formation of the axon network.

**Acknowledgements:** Work supported by GAČR 14-16755S, GAUK 396213, MŠMT 7AMB12FR002, NIH 1RO1DCO12441 and ANR 2010-BLAN-1401-01.

**References**

1. Smit D, Fouquet C, Pincet F, Trembleau A, Zapotocky M. Axon zippering in neuronal cell culture and its biophysical modeling. BMC Neurosci. 2015;16(Suppl. 1):P298.
2. Roberts A, Taylor JSH. A scanning electron microscope study of the development of a peripheral sensory neurite network. J Embryol Exp Morph. 1982;69:237–50.

## P47 Efficient signal processing and sampling in random networks that generate variability

### Sakyasingha Dasgupta^1,2^, Isao Nishikawa^3^, Kazuyuki Aihara^3^, Taro Toyoizumi^2^

#### ^1^IBM Research - Tokyo, Tokyo, Japan; ^2^RIKEN Brain Science Institute, Tokyo, Japan; ^3^The University of Tokyo, Tokyo, Japan

##### **Correspondence:** Sakyasingha Dasgupta - sdasgup@jp.ibm.com

*BMC Neuroscience* 2016, **17(Suppl 1)**:P47

**Fig. 32:**
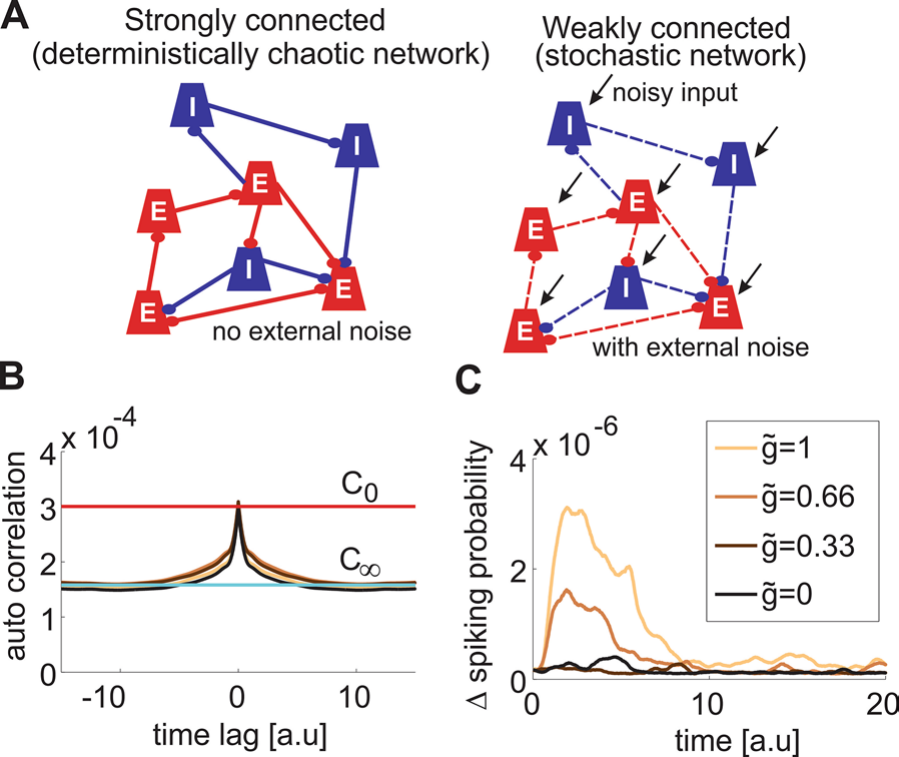
**A** Schematic illustrations of the two balanced QIF networks models considered in the present study. The left network consists of strongly coupled neurons without noise, while the right network consists of weak coupling among neurons with noisy input. **B** Nearly identical rate autocorrelation functions in the two networks. The *red line* (*C*_0_) represents the value of the autocorrelation at time 0 and cyan line (*C*_∞_) is the value of auto-correlation function in the limit of large *t*. **C** Change in spiking probability for different network connectivity strengths $(\tilde{g}),$ after being stimulated by a brief input at time t = 0

The source of cortical variability and its influence on signal processing remain an open question. We address the latter, by studying two types of randomly connected networks of quadratic integrate-and-fire neurons with balanced excitation-inhibition that produce irregular spontaneous activity patterns (Fig. 32A): (a) a deterministic network with strong synaptic interactions that actively generates variability by chaotic dynamics (internal noise) and (b) a stochastic network that has weak synaptic interactions but receives noisy input (external noise), e.g. by stochastic vesicle releases. These networks of spiking neurons are analytically tractable in the limit of a large network-size and slow synaptic-time-constant. Despite the difference in their sources of variability, spontaneous (baseline) activity patterns of these two models are indistinguishable unless majority of neurons are simultaneously recorded. We characterize the network behavior with dynamic mean field analysis and reveal a single-parameter family that allows interpolation between the two networks, sharing nearly identical spontaneous activity (Fig. 32B). Despite the close similarity in the spontaneous activity, the two networks exhibit remarkably different sensitivity to external stimuli. Input to the former network reverberates internally and can be successfully read out over long time. Contrarily, input to the latter network rapidly decays and can be read out only for short time. This is also observed in the significant changes in the spiking probability of evoked responses across this family (Fig. 32C). The difference between the two networks is further enhanced if input synapses undergo activity-dependent plasticity, producing significant difference in the ability to decode external input from neural activity. We show that, this difference naturally leads to distinct performance of the two networks to integrate spatio-temporally distinct signals from multiple sources. Unlike its stochastic counterpart, the deterministic chaotic network activity can serve as a reservoir to perform near optimal Bayesian integration and Monte-Carlo sampling from the posterior distribution. We describe implications of the differences between deterministic and stochastic neural computation on population coding and neural plasticity.

## P48 Modeling the effect of riluzole on bursting in respiratory neural networks

### Daniel T. Robb^1^, Nick Mellen^2^, and Natalia Toporikova^3^

#### ^1^Department of Mathematics, Computer Science and Physics, Roanoke College, Salem, VA 24153, USA; ^2^Department of Pediatrics, University of Louisville, Louisville, KY 40208, USA; ^3^Department of Biology, Washington and Lee University, Lexington, VA 24450, USA

##### **Correspondence:** Daniel T. Robb - robb@roanoke.edu

*BMC Neuroscience* 2016, **17(Suppl 1)**:P48

To accommodate constantly changing environmental and metabolic demands, breathing should be able to vary flexibly within a range of frequencies. The respiratory neural network in the pre-Botzinger complex of the ventrolateral medulla controls and flexibly maintains the breathing rhythm, coordinating network-wide bursting to signal the inspiratory phase of the breath. The frequency of this rhythmic activity is controlled by a number of neuromodulators, the majority of which are excitatory. Therefore, the central pattern generator for rhythmic respiratory activity should possess two seemingly contradictory properties: it has to be able to change frequency in response to excitatory input, but it also has to preserve stable rhythmic activity under a wide range of conditions.

A persistent sodium current (*I*_*NaP*_) been identified as one of the key currents for generation of inspiratory activity [1]. It has been shown that some of the neurons in Pre-BotC possess an intrinsic bursting mechanism, which relies on inactivation of this current. Higher expression of *I*_*NaP*_ correlates with higher burst frequency of a single pacemaker neuron [2]. However, the *I*_*NaP*_ pacemaker mechanism can only function within very narrow ranges of external excitation—NaP dependent pacemaker tends to switch to tonic firing after a small increase in depolarizing current [3].

**Fig. 33:**
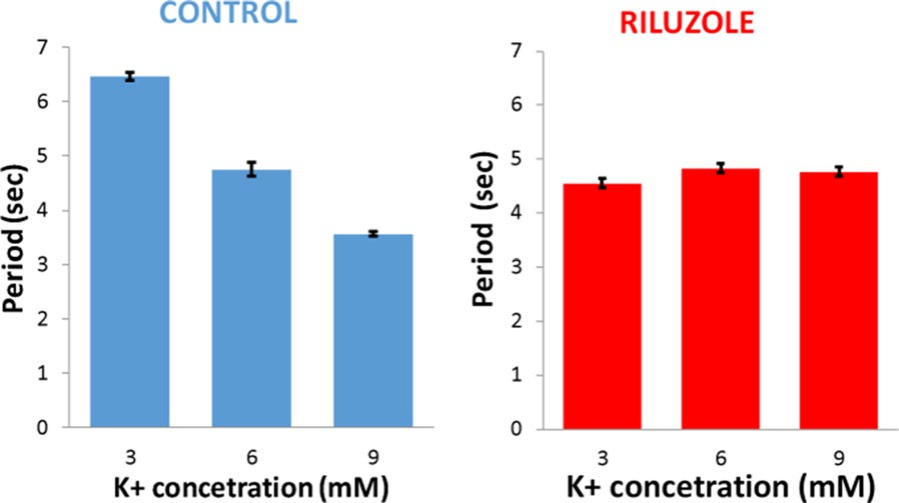
Summary of experiment on the effect of riluzole on the dependence of burst frequency on potassium concentration. Without riluzole (*left*), the frequency increases steadily with increasing potassium concentration. With riluzole present (*right*), the frequency remains essentially constant with increasing potassium concentration

In this combined experimental and computational study, we tested the effect of the persistent sodium blocker Riluzole (RIL) in several different levels of continuous depolarization, simulated by application of K+. Whereas increased potassium increases the bursting frequency of the control network, in the presence of RIL the increased potassium does not alter the bursting frequency (Fig. 33). These findings indicate that *I*_*NaP*_ is responsible for flexible modulation of respiratory rhythm, but there is another mechanism, which can sustain rhythmic activity in its absence. We developed a computational model which incorporates a Calcium sensitive Non-specific cationic current (*I*_*caN*_) in addition to *I*_*NaP*_. Our simulations indicate that *I*_*caN*_ and *I*_*NaP*_ can maintain the rhythm in respiratory neurons in the presence of RIL, and are capable of providing stable oscillations in the presence of tonic excitation by K+.

**References**

1. Butera RJ Jr, Rinzel J, Smith JC. Models of respiratory rhythm generation in the pre-Bötzinger complex. I. Bursting pacemaker neurons. J Neurophysiol. 1999;82:382–97.
2. Purvis LK, Smith JC, Koizumi H, Butera RJ. Intrinsic bursters increase the robustness of rhythm generation in an excitatory network. J Neurophysiol. 2007;97:1515–26.
3. Del Negro CA, Morgado-Valle C, Hayes JA, Mackay DD, Pace RW, Crowder EA, Feldman JL. Sodium and calcium current-mediated pacemaker neurons and respiratory rhythm generation. J Neurosci Off J Soc Neurosci. 2005;25:446–53.

## P49 Mapping relaxation training using effective connectivity analysis

### Rongxiang Tang^1^, Yi-Yuan Tang^2^

#### ^1^Department of Psychology, Washington University in St. Louis, St. Louis, MO 63130, USA; ^2^Department of Psychological Sciences, Texas Tech University, TX 79409, USA

##### **Correspondence:** Yi-Yuan Tang - yiyuan.tang@ttu.edu

*BMC Neuroscience* 2016, **17(Suppl 1)**:P49

Relaxation training (RT)is a behavioral therapy that has been applied in stress management, muscle relaxation and other health benefit. However, compared to short-term meditation training, previous studies did not show the significant differences in brain changes following same amount of RT [1,2]. One possible reason might derive from the insensitive correlation based routine functional connectivity method that could not reveal training-related changes in effective connectivity (directed information flow) among these distributed brain regions. Here, we applied a novel spectral dynamic causal modeling (spDCM) to resting state fMRI to characterize changes in effective connectivity.

Twenty-three healthy college students were recruited through campus advertisements and received 4 weeks of RT (10 h in total), previously reported in our randomized studies [1, 2]. All neuroimaging data were collected using an Allegra 3-Telsa Siemens scanner and processed using the Data Processing Assistant for Resting-State fMRI, which is based on SPM and Resting-State fMRI Data Analysis Toolkit [3]. For each participant, the subsequent standard procedures included slice timing, motion correction, regression of WM/CSF signals, and spatial normalization [3]. Based on previous literature, we specified four regions of interest within default mode network (DMN)—medial prefrontal cortex (mPFC), posterior cingulate cortex (PCC), and bilateral inferior parietal lobule (left IPL and right IPL), same coordinates as in previous spDCM studies [4]. A standard DCM analysis involves a specification of plausible models, which are then allows the model parameters to be estimated following Bayesian model selection. In both pre- and post-RT conditions, the procedure selected the fully connected model as the best model with a posterior probability of almost 1. The fully connected model had 24 parameters describing the extrinsic connections between nodes, the intrinsic (self-connections) within nodes and neuronal parameters describing the neuronal fluctuations within each node. We used Bayesian Parametric Average to quantify the differences between pre- and post-RT, and a classical multivariate test—canonical variate analysis to test for any significances in these differences [4]. Our results showed no significant differences in causal relationships among the above nodes following RT (all P > 0.05).

**Conclusions** Four weeks of RT could not induce significant changes in effective connectivity among DMN nodes. Long-term RT effect on brain changes warrants further investigation.

**Acknowledgements:** This work was supported by the Office of Naval Research.

**References**

1. Tang YY, Holzel BK, Posner MI. The neuroscience of mindfulness meditation. *Nat Rev Neurosci.* 2015;16:213–25.
2. Tang YY, Lu Q, Geng X, Stein EA, Yang Y, Posner MI. Short-term meditation induces white matter changes in the anterior cingulate. Proc Natl Acad Sci USA. 2010;107:15649–52.
3. Tang YY, Tang R, Posner MI. Brief meditation training induces smoking reduction. Proc Natl Acad Sci USA. 2013;110:13971–75.
4. Razi A, Kahan J, Rees G, Friston KJ. Construct validation of a DCM for resting state fMRI. Neuroimage. 2015;106:1–14.

## P50 Modeling neuron oscillation of implicit sequence learning

### Guangsheng Liang^1^, Seth A. Kiser^2,3^, James H. Howard, Jr.^3^, Yi-Yuan Tang^1^

#### ^1^Department of Psychological Sciences, Texas Tech University, TX 79409, USA; ^2^The Department of Veteran Affairs, District of Columbia VA Medical Center, Washington, DC 20420, USA; ^3^Department of Psychology, The Catholic University of America, Washington, DC 20064, USA

##### **Correspondence:** Yi-Yuan Tang - yiyuan.tang@ttu.edu

*BMC Neuroscience* 2016, **17(Suppl 1)**:P50

Implicit learning (IL) occurs without goal-directed intent or conscious awareness but has important influences on our everyday functioning and overall health such as environmental adaptation, developing habits and aversions. Most of IL studies used event-related potentials (ERPs) to study brain response by taking the grand average of all event-related brain signals. How neuron oscillation (EEG frequency band) involves in IL remains unknown. Moreover, ERP analysis requires brain signals that are not only time locked, but also phase locked to the event, therefore the information with phase locked signals are missed and not presented in potentials. To address this issue, we applied time–frequency analysis and cluster-based permutation test in this study.

Fifteen healthy participants were recruited to perform three sessions of triplets learning task (TLT), an IL task commonly used in the field [1]. Three successive cues were presented and participants were asked to observe the first two cues and only respond to the third cue (target) by pressing corresponding keys. During the task, EEG signals were recorded. Cluster based permutation on alpha and theta band is used to deal with family-wise error rate and in the same time, help to find out difference occurred in specific time range along with spatial information among different triplet types.

Base on the behavioral result, overall learning occurs in session1, while triplet-specific learning takes place in session2. We find significant difference in both alpha (8–13 Hz) and theta (4–8 Hz) frequency band. For alpha band, power modulation shows significant difference between high versus low frequency triplet group in session2 in the frontal cortex. For theta band, theta power shows significant difference between session1 and session3 in the frontal cortex. It started from as early as target onset until the end of the trial in high frequency triplet group. However, in the low frequency triplet group, the power differential occurs later, from around 1000 ms till the end of the next trial.

**Conclusions** Behavioral result showed that the brain learned the regularity of sequence implicitly. Alpha power modulation indicated that the brain allocated resource in attention among two different triplet types. Theta power modulation showed the difference of memory processing and retrieval among two different triplet types. Our results indicated that participants did not find the regularity of the triplet types till the end of the study, but the brain in fact reacts to these two different triplet types differently.

**Acknowledgements:** This work was supported by the Office of Naval Research.

**Reference**

1. Howard JH, Howard DV, Dennis N, Kelly AJ. Implicit learning of predictive relationships in three-element visual sequences by young and old adults. J Exp Psychol Learn Mem Cogn. 2008, 34: 1139–57.

## P51 The role of cerebellar short-term synaptic plasticity in the pathology and medication of downbeat nystagmus

### Julia Goncharenko^1^, Neil Davey^1^, Maria Schilstra^1^, Volker Steuber^1^

#### ^1^Centre for Computer Science and Informatics Research, University of Hertfordshire, Hatfield, AL10 9EJ, UK

##### **Correspondence:** Julia Goncharenko - i.goncharenko@herts.ac.uk

*BMC Neuroscience* 2016, **17(Suppl 1)**:P51

Downbeat nystagmus (DBN) is a common eye fixation disorder that is linked to cerebellar pathology. DBN patients are treated with 4-aminopyridine (4-AP), a K channel blocker, but the underlying mechanism is unclear. DBN is associated with an increased activity of floccular target neurons (FTNs) in the vestibular nuclei. It was previously believed that the reason for the increased activity of FTNs in DBN is a pathological decrease in the spike rate of their inhibitory Purkinje cell inputs, and that the effect of 4-AP in treating DBN could be mediated by an increased Purkinje cell activity, which would restore the inhibition of FTNs and bring their activity back to normal [1]. This assumption, however, has been questioned by in vitro recordings of Purkinje cells from tottering (tg/tg) mice, a mouse model of DBN. It was shown that therapeutic concentrations of 4-AP did not increase the spike rate of the Purkinje cells, but that they restored the regularity of their spiking, which is impaired in tg/tg mice [2].

Prompted by these experiments, Glasauer and colleagues performed computer simulations to investigate the effect of the regularity of Purkinje cell spiking on the activity of FTNs [3]. Using a conductance based FTN model, they found that changes in the regularity of the Purkinje cell input only affected the FTN spike rate when the input was synchronized. In this case, increasing the regularity of the Purkinje cell spiking resulted in larger gaps in the inhibitory input to the FTN and an increased FTN spike rate. These results predict that the increased irregularity in the Purkinje cell activity in DBN should lead to a decreased activity of the FTNs, rather than the increased activity that is found in experiments, and they are therefore unable to explain the therapeutic effect of 4-AP.

However, the model by Glasauer and colleagues does not take short-term depression (STD) at the Purkinje cell—FTN synapses into account. We hypothesized that this absence of STD could explain the apparent contradiction between the experimental [2] and computational [3] results. To study the role of STD in the pathology and 4-AP treatment of DBN, we used a morphologically realistic conductance based model of a cerebellar nucleus (CN) neuron [4, 5] as an FTN model to simulate the effect of irregular versus regular Purkinje cell input. The coefficients of variation of the irregular and regular Purkinje cell spike trains during DBN and after 4-AP treatment, respectively, were taken from recordings from wild-type and tg/tg mice [6], which served as a model system for DBN. We presented the FTN model with synchronized and unsynchronized input and found that, for both conditions, irregular (DBN) input trains resulted in higher FTN spike rates than regular (4-AP) ones. In the presence of unsynchronized Purkinje cell input, the acceleration of the FTN spike output during simulated DBN and the deceleration during simulated 4-AP treatment depended on STD at the Purkinje cell synapses. Our results provide a potential explanation for the pathology and 4-AP treatment of pathological nystagmus.

**References**

1. Glasauer S, Kalla R, Buttner U, Strupp M, Brandt T. 4-aminopyridine restores visual ocular motor function in upbeat nystagmus. J Neurol Neurosurg Psychiatry. 2005;76:451–3.
2. Alvina K, Khodakhah K. The therapeutic mode of action of 4-aminopyridine in cerebellar ataxia. J Neurosci. 2010;30:7258–68.
3. Glasauer S, Rössert C, Strupp M. The role of regularity and synchrony of cerebellar Purkinje cells for pathological nystagmus. Ann NY Acad Sci. 2011;1233:162–7.
4. Steuber V, Schultheiss NV, Silver RA, de Schutter E, Jaeger D. Determinants of synaptic integration and heterogeneity in rebound firing explored with data-driven models of deep cerebellar nucleus cells. J Comp Neurosci. 2011;30:633–58.
5. Luthman J, Hoebeek FE, Maex R, Davey N, Adams R, de Zeeuw CI, Steuber V. STD-dependent and independent encoding of input irregularity as spike rate in a computational model of a cerebellar nucleus neuron. Cerebellum. 2011;10:667–82.
6. Hoebeek FE, Stahl JS, van Alphen AM, Schonewille M, Luo C, Rutteman M, van den Maagdenberg AM, Molenaar PC, Goossens HH, Frens MA, et al. Increased noise level of Purkinje cell activities minimizes impact of their modulation during sensorimotor control. Neuron 2005, 45(6):953–965.

## P52 Nonlinear response of noisy neurons

### Sergej O. Voronenko^1,2^, Benjamin Lindner^1,2^

#### ^1^Department of Physics, Humboldt University, Berlin, 10099, Germany; ^2^Bernstein Center for Computational Neuroscience, Berlin, 10115, Germany

##### **Correspondence:** Sergej O. Voronenko - sergej@physik.hu-berlin.de

*BMC Neuroscience* 2016, **17(Suppl 1)**:P52

**Fig. 34:**
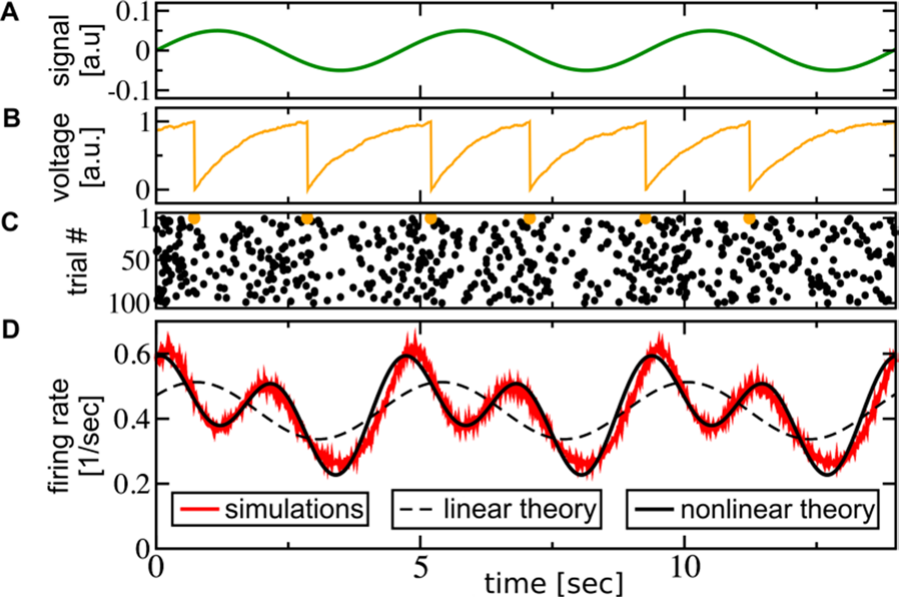
Nonlinear modulation of the firing rate by a cosine signal. **A** Signal, **B** subthreshold voltage, **C** rasterplot, **D** The time-dependent firing rate (*red*, noisy trace) is significantly different from the linear theory (*dashed line*) but is accurately described by the second-order response (*solid line*)

In many neuronal systems that exhibit high trial-to-trial variability the time-dependent firing rate is thought to be the main information channel for time-dependent signals. However, for nerve cells with low intrinsic noise and highly oscillatory activity synchronization, mode locking and frequency locking seem to be of major importance. Here, we present an extension to the linear response theory [1, 2] for the leaky integrate-and-fire neuron model to second order and demonstrate how the time-dependent firing rate can exhibit features that are reminiscent of mode-locking and frequency-locking. Although our theory allows to predict the response to general weak time-dependent signals, the second-order effects are best demonstrated using cosine signals as in Fig. 34A. We consider a leaky integrate-and-fire model for which the subthreshold voltage, Fig. 34B, is subject to the signal and to Gaussian white noise. Whenever the voltage hits the threshold, it is reset to zero and a spike time is recorded in the raster plot, Fig. 34C. The firing rate can be obtained numerically by averaging over the spike trains or via a perturbation approach similar to the weakly nonlinear analysis in [3]. We find that the firing rate can exhibit pronounced nonlinear behavior as can be seen from the excitation of a harmonic oscillation in Fig. 34D. Further effects that are not shown in Fig. 34 but are revealed by our analysis are a signal-dependent change of the mean firing rate and a pronounced nonlinear response to the sum of two cosine signals.

**Summary and conclusions** Here we demonstrate that the time-dependent firing rate (equivalent to the instantaneous population rate for neurons driven by a common stimulus) can exhibit pronounced nonlinearities even for weak signal amplitudes. The linear theory does not only give quantitatively wrong predictions but also fails to capture the timing of the modulation peaks. Hence, our theory has not only implications for sinusoidal stimulation that is commonly used to study dynamic properties of nerve cells but also demonstrates the relevance of the nonlinear response for the encoding of complex time-dependent signals.

**Acknowledgements:** This work was supported by the BMBF (FKZ: 01GQ1001A) and the DFG (research training group GRK1589/2).

**References**

1. Brunel N, Chance FS, Fourcaud N, Abbott LF. Effects of synaptic noise and filtering on the frequency response of spiking neurons. PRL. 2001;86(10):2186–9.
2. Lindner B, Schimansky-Geier L. Transmission of noise coded versus additive signals through a neuronal ensemble. PRL. 2001;86(14):2934–7.
3. Brunel N, Hakim V. Fast global oscillations in networks of integrate-and-fire neurons with low firing rates. Neural Comput. 1999;11(7):1621–71.

## P53 Behavioral embedding suggests multiple chaotic dimensions underlie *C. elegans* locomotion

### Tosif Ahamed^1^, Greg Stephens^1,2^

#### ^1^Biological Physics Theory Unit, Okinawa Institute of Science and Technology, Okinawa 904-0495, Japan; ^2^Department of Physics and Astronomy, Vrije Universiteit Amsterdam

##### **Correspondence:** Tosif Ahamed - tosif.ahamed@oist.jp

*BMC Neuroscience* 2016, **17(Suppl 1)**:P53

**Fig. 35:**
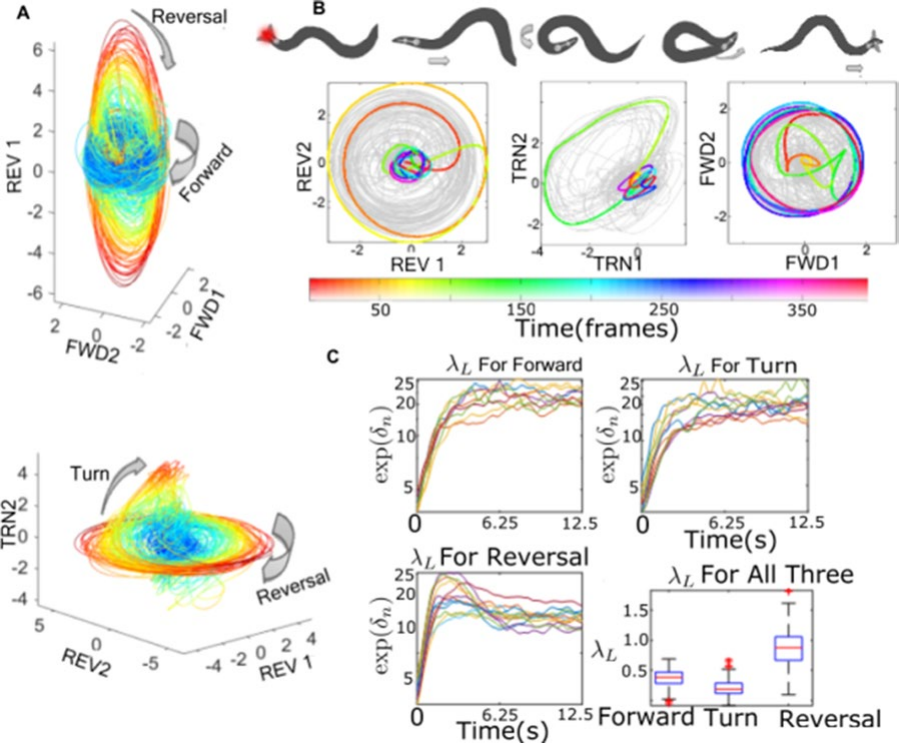
Phase space portrait and divergence of nearby trajectories. **A** The *top panel* shows the orthogonal relationship between the forward and reversal behaviors, while the *bottom panel* shows the transition from reversal to an omega turn in the phase space. To aid visualization color coding is done by radial distance from the origin. **B** Escape response visualized in the phase planes. When the worm is hit with a laser impulse, it makes a reversal, followed by an omega turn and then resumes forward crawling. *Color map* encodes time in frames. **C** Divergence curves for the three different attractors. Y-axis shows the exponential of the divergence between neighboring trajectories plotted on a semilog scale on the y axis, each curve corresponds to a single worm (n = 12). *λ*_*L*_ is estimated by calculating the slope of the linear region. *Boxplots* show the range of *λ*_*L*_ obtained from different animals

Behavior is the primary output of an organism; genetic and neural circuits, no matter how complex, seek to optimize this output. A quantitative understanding of behavior is therefore crucial to our understanding of biological processes. A key characteristic of natural behavior is variability; even the most stereotyped movements such as reaching to a target, which are similar in aggregate, can vary substantially from trial to trial. In motor control such variability is often ascribed to noise in the sensorimotor control circuit. On the other hand, deterministic dynamical systems can generate variability intrinsically when operating in a chaotic regime. Differentiating between the two is important as they generate separate mechanistic predictions about how variability is generated in the brain. Here, we use tools from nonlinear dynamics to understand behavioral variability in the movement of *C. elegans*. We reconstruct a 6-dimensional phase space by developing a novel extension of multivariate singular systems analysis [1] and applying it to a low-dimensional but complete representation of worm postures obtained from videos of freely foraging worms [2]. At a coarse level, the reconstructed phase space naturally separates into three stereotyped behaviors: forward locomotion, reversals and turns (Fig. 35A, B). However, there is also substantial variability at finer scales, which is reflected in positive maximal Lyapunov exponents (MLE) [3] within trajectories corresponding to each individual behavior (Fig. 35C). The MLEs calculated this way differ significantly from MLEs calculated from a random shuffle of the data that preserves its linear structure (or power spectrum). This implies that the positive MLEs, which indicate sensitive dependence to initial conditions in *C. elegans* behavior are a result of nonlinear structure present in the dynamics. These results are strengthened by the fact that we observe little inter-animal variability in the estimated values of MLE, additionally the values also agree well with the estimated time scales of the three behaviors. Based on these observations we propose that *C. elegans* behavior might be driven by the activity of multiple coupled chaotic attractors. We expect our analysis will also be relevant in understanding global neural dynamics, recently imaged in freely-moving worms [4].

**References**

1. Read PL. Phase portrait reconstruction using multivariate singular systems analysis. Phys D. 1993;69(3):353–65.
2. Stephens GJ, Johnson-Kerner, B, Bialek W, Ryu WS. Dimensionality and dynamics in the behavior of *C. elegans*. PLoS Comput Biol. 2008;4(4):e1000028.
3. Kantz H. A robust method to estimate the maximal Lyapunov exponent of a time series. Phys Lett A. 1994;185(1):77–87.
4. Nguyen JP, Shipley FB, Linder AN, Plummer GS, Liu M, Setru SU, Shaevitz JW, Leifer AM. Whole-brain calcium imaging with cellular resolution in freely behaving *Caenorhabditis elegans*. PNAS. 2015;201507110.

## P54 Fast and scalable spike sorting for large and dense multi-electrodes recordings

### Pierre Yger^1^, Baptiste Lefebvre^1^, Giulia Lia Beatrice Spampinato^1^, Elric Esposito, Marcel Stimberg et Olivier Marre^1^

#### ^1^Institut de la Vision, INSERM UMRS 968, CNRS UMR 7210, Paris

##### **Correspondence:** Pierre Yger - pierre.yger@inserm.com

*BMC Neuroscience* 2016, **17(Suppl 1)**:P54

Understanding how assemblies of neurons encode information requires recording of large populations of cells in the brain. In recent years, multi-electrode arrays and large silicon probes have been developed to record simultaneously from thousands of electrodes packed with a high density. However, these new devices challenge the classical way to do spike sorting. First, the large number of electrodes preclude approaches based on manual clustering. Even automatic approaches need to be fast enough to handle the amount of extracellular data. Second, the density of the electrodes is high enough so that a single spike will be detected on many electrodes. So the different channels must be processed simultaneously. Third, within a large and dense array of electrodes, overlapping spikes are rather the rule than the exception, and it is known that classical clustering methods cannot easily capture the synchronous occurrence of two spikes from two different cells [1].

Here we developed a new software to solve all these aforementioned issues, based on a highly automated algorithm to extract spikes from extracellular data, and show that this algorithm reached near optimal performance both in vitro and in vivo. The algorithm is composed of two main steps: (1) a “template-finding” phase to extract the cell templates, i.e. the pattern of activity evoked over many electrodes when one neuron fires an action potential; (2) a “template-matching” phase where the templates are matched onto the raw data to find the location of the spikes. The manual intervention by the user is reduced to the minimal, and the time spent on manual curation did not scale with the number of electrodes. For the template-finding phase, we start by detecting all the possible times in the raw data that could contain a spike. Spikes are then clustered into groups using a density-based clustering derived from [1], and we then extract the template corresponding to each group. In the fitting phase, we match the templates onto the raw data with a method that allows amplitude variation for each template [2]. The algorithm is written in Python and is entirely parallelized such that it can handle large amount of data. It also provides a graphical user interface so that the output of the algorithm can be checked, and to refine the sorting.

We tested our algorithm with large-scale data from in vitro and in vivo recordings, from 32 and up to 4225 electrodes. In all cases, we estimated its performance on data with ground truth, i.e. cases where the solution to the sorting problem is at least partially known. The performance was always close to the maximal expected performance. Therefore, our method appears as a general solution to sort spikes from large-scale extracellular recordings.

**References**

1. Einevoll GT, et al. Towards reliable spike-train recordings from thousands of neurons with multielectrodes. Curr Opin Neurobiol. 2012;22:11–17.
2. Rodriguez A, et al. Clustering by fast search and find of density peaks. Science. 2014;344(6191):1492–96.

## P55 Sufficient sampling rates for fast hand motion tracking

### Hansol Choi^1^, Min-Ho Song^2^

#### ^1^Bernstein Center Freiburg, Institute of Biology III, University of Freiburg, Germany, 79100; ^2^fourMs group, Dept. Musicology, University of Oslo, Norway, 0371

##### **Correspondence:** Min-Ho Song - minho.song@imv.uio.no

*BMC Neuroscience* 2016, **17(Suppl 1)**:P55

**Fig. 36:**
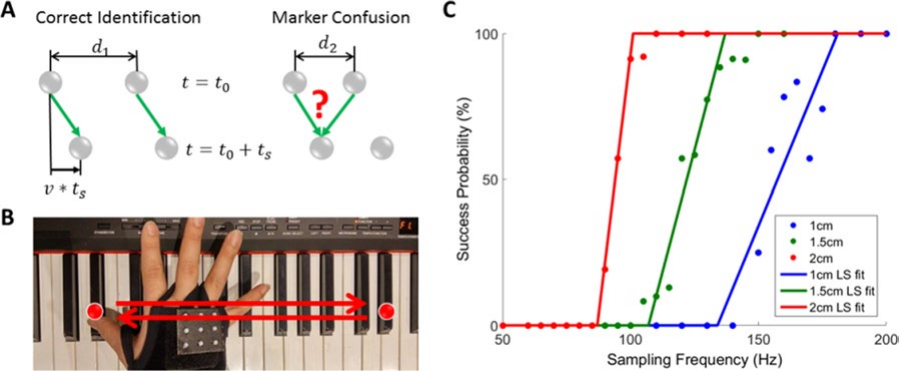
Experimental design and result. **A** Marker confusion. *Grey dots* are markers. *d*_1_, *d*_2_ are the distances between markers, *t*_s_ is the sampling latency, v is speed of marker. *Green lines* show the markers, which identified as same. *Left* correct identification *right* example of marker confusion. **B** Experimental set up. *Red dots* are keys to press by the thumb and the little finger during repeats. **C** The probabilities of continuous marker identification

When tracking fine motor behaviors in human body parts, passive marker-based tracking is one of the best-suited methods not only because of its high spatial precision and temporal resolution, but also allowing high degrees-of-freedom [1]. However, the passive marker approach suffers from identity confusion problem (Fig. 36A) between the markers. As the speed of motion increases, sufficient sampling rate is required to avoid the problem. In a recent study [2], we reported that the problem still occurs even with the sampling rate significantly higher than the Nyquist sampling rate. The study suggested a sampling rate criterion to avoid identity problem for the worst-case condition.

In this poster, the confusion problem is tested in more realistic human motor control behavior. Grids of 3 × 3 markers with different distances (1, 1.5 and 2 cm) were attached to a skilled piano player’s right hand (Fig. 36B). The experimental task was repeated right-hand alternative keystrokes between D#5 and D#7 (two octave) with a tempo of 176 bpm for 10 s. This is an excerpt from Liszt’s *La Campanella*, which requires fast horizontal jump of the right-hand. These motions were recorded with 7 optical motion capture cameras (Qualisys Ltd. Oqus 400) changing the sampling rates from 50 to 200 Hz. The maximum frequency components of these hand movements were lower than 8 Hz.

The probability of successful tracking is measured by counting the number of successful repetition of the center marker (Fig. 36C). Estimated required sampling rates for successful tracking (where the probabilities reach 100 %) were 101, 137z, and 181 Hz (fitted to piecewise linear functions by expectation maximization). The theoretically predicted values are 176, 235, and 353 Hz [2].

We found that the required sampling rates are lower than the theoretical criterion. This is because the theoretical prediction was developed to avoid the worst case where marker trajectories overlap from perfect periodic motion; not realistic for human movement, which has variability. Our results show that in practical situations involving human movements, the sampling criterion can be weakened considerably. But, it should be note that a motion slower than 10 Hz still requires more than 100 Hz, which far exceeds the Nyquist sampling rate.

**References**

1. Guerra-Filho G. Optical motion capture: theory and implementation. J Theor Appl Inf. 2005;12(2):61–8.9
2. Song M-H, Godøy RI. How fast is your body motion? Determining a sufficient frame rate for an optical motion tracking system using passive markers. PLoS One (in press).

## P56 Linear readout of object manifolds

### SueYeon Chung^1^, Dan D. Lee^2^, Haim Sompolinsky^1,3^

#### ^1^Center for Brain Science, Harvard University, Cambridge, MA 02138, USA; ^2^Department of Electrical and Systems Engineering, University of Pennsylvania, Philadelphia, PA 19104, USA; ^3^Edmond and Lily Safra Center for Brain Sciences, Hebrew University, Jerusalem 91904, Israel

##### **Correspondence:** SueYeon Chung - schung@fas.harvard.edu

*BMC Neuroscience* 2016, **17(Suppl 1)**:P56

**Fig. 37:**
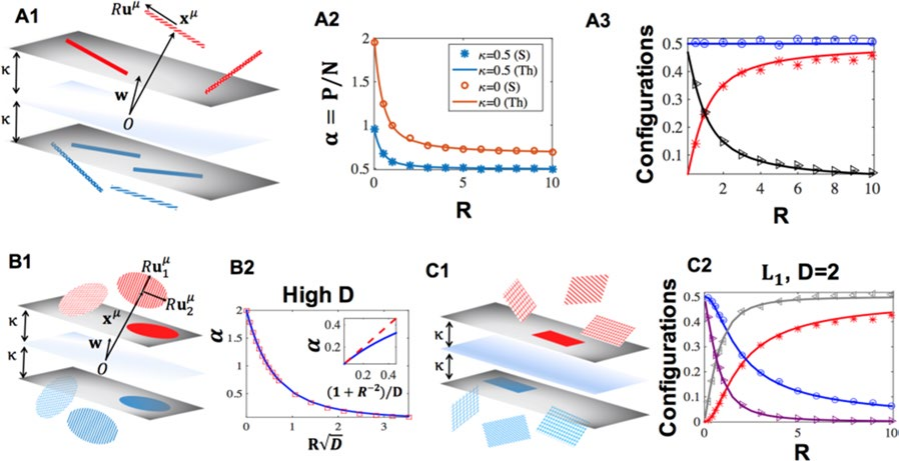
Theoretical predictions (*lines*) and numerical simulation (markers) are shown. **A1** Classification of line segments. (*Solid*) *lines* embedded in the margin, (*dotted*) *lines* touching the margin, (striped) interior lines .**A2** Capacity α = P/N of a network N = 200 as a function of R (line length) with margins κ = 0, 0.5. **A3** Fraction of configurations at capacity with κ = 0. (*red*) lines in the margin, (*blue*) touching the margin, (*black*) interior lines. **B1** D2 balls, **B2** capacity α = P/N for κ = 0 for large D = 50 and R $\propto$ D^−1/2^ as a function of ${\text{R}}\sqrt {\text{D}}$. (*Blue solid*) α_D_(0, R) compared with α_0_(${\text{R}}\sqrt {\text{D}}$) (*red square*). (*Inset*) capacity α at κ = 0 for 0.35 ≤ R ≤ 20 and D = 20: (*blue*) theoretical α compared with approximate form (1 + R^−2^)/D (*red dashed*). **C1** 2D L1 balls. **C2** Fraction of configurations as a function of radius R at capacity with κ = 0. (*red*) entire manifold embedded, (*blue*) touching margin at a single vertex, (*gray*) touching with two corners (one side), (*purple*) interior manifold

Objects are represented in sensory systems by continuous manifolds due to sensitivity of neuronal responses to changes in physical features such as location, orientation, and intensity [1]. It has been hypothesized that object identity can be decoded from high level representations, by simple downstream readout networks. What makes certain sensory representations better suited for invariant decoding of objects by downstream networks? We generalize Gardner’s statistical mechanical analysis of points [2, 3] and establish a replica theory of linear classification of manifolds synthesizing statistical and geometric properties of high dimensional signals. We show how changes in the dimensionality, size, and shape of the object manifolds affect the capacity and the distribution of configurations in downstream perceptrons (Fig. 37).

Our analysis shows how linear separability of the manifolds depends intimately upon the dimensionality, size and shape of the the manifolds. These properties are expected to differ at different stages in the sensory hierarchy. Thus, the present work enables systematic analysis of the degree to which this reformatting enhances the capacity for object classification in different sensory processing stages. The present work lays the groundwork for a computational theory of neuronal processing of objects in the presence of variability, providing quantitative measures for assessing the properties of representations in biological and artificial neural networks.

**References**

1. DiCarlo JJ, Cox DD. Untangling invariant object recognition. Trends Cogn Sci. 2007;11(8):333–41.
2. Gardner E. Maximum storage capacity in neural networks. EPL (Europhys Lett). 1987;4(4):481
3. Abbott LF, Kepler TB. Universality in the space of interactions for network models. J Phys A Math Gen. 1989;22(12):2031.

## P57 Differentiating models of intrinsic bursting and rhythm generation of the respiratory pre-Bötzinger complex using phase response curves

### Ryan S. Phillips^1,2^, Jeffrey Smith^1^

#### ^1^NINDS, NIH, Bethesda, MD 20892, USA; ^2^Department of Physics, University of New Hampshire, Durham, NH, 03824, USA

##### **Correspondence:** Ryan S. Phillips - Ryan.Phillips@nih.gov

*BMC Neuroscience* 2016, **17(Suppl 1)**:P57

The pre-Bötzinger complex (PBC) is an essential rhythmogenic brainstem nucleus located in the ventrolateral medulla. Rhythmic output from the PBC is relayed through premotor and motor neurons to the diaphragm and intercostal muscles to drive the active inspiratory phase of respiration. The specific biophysical mechanisms responsible for generating rhythmic bursting and network synchronization are not well understood and remain a highly controversial topic within the field. A wide variety of biophysical mechanisms have been proposed to explain the origins of intrinsic bursting and rhythmogenesis including persistent sodium currents [1, 2], calcium-activated nonspecific cation channels [2, 3], inositol trisphosphate (IP3) signaling [2], and synaptic mechanisms [4]. Computational simulations of these models produce similar patterns of bursting and network synchronization compared to each other and experimental recordings despite having different underlying mechanisms. In this theoretical study we demonstrate a method to differentiate between biophysically distinct models using phase response curves (PRCs). PRCs characterize the change in phase of an oscillator as a function of the timing of a perturbation to the system. Depolarizing and hyperpolarizing perturbations were generated by incorporating the light sensitive channels, Channelrhodopsin-2 and Archaerhodopsin, respectively, into our conductance based PBC models. A library of model PBC neurons was generated by varying the conductance of each ion channel over equally spaced intervals. PRCs were then calculated for each neuron capable of producing rhythmic bursting. Preliminary results found that in general depolarizing perturbations produced qualitatively similar PRCs and could both advance or delay the next cycle. Conversely, hyperpolarizing perturbations produced qualitatively distinct PRCs depending on the combination of conductance magnitudes. In conclusion, these PRCs provide a method for differentiating models of intrinsic bursting and rhythm generation based on underlying biophysical mechanisms and provide a means for interpreting experimentally derived PRCs from the PBC.

**References**

1. Butera RJ, Rinzel J, Smith JC. Models of respiratory rhythm generation in the pre-Bötzinger complex. I. Bursting pacemaker neurons. J Neurophysiol. 1999;82:382–97.
2. Jasinski PE, Molkov YI, Shevtsova NA, Smith JC, Rybak IA. Sodium and calcium mechanisms of rhythmic bursting in excitatory neural networks of the pre-Bötzinger complex: a computational modelling study. Eur J Neurosci. 2013;37:212–30.
3. Rubin JE, Hayes JA, Mendenhall JL, Del Negro CA. Calcium-activated nonspecific cation current and synaptic depression promote network-dependent burst oscillations. Proc Natl Acad Sci USA. 2009;106:2939–44.
4. Guerrier C, Hayes JA, Fortin G, Holcman D. Robust network oscillations during mammalian respiratory rhythm generation driven by synaptic dynamics. Proc Natl Acad Sci. 2015;201421997.

## P58 The effect of inhibitory cell network interactions during theta rhythms on extracellular field potentials in CA1 hippocampus

### Alexandra Pierri Chatzikalymniou^1,2^, Katie Ferguson^1,3^, Frances K. Skinner^1,2,4^

#### ^1^Krembil Research Institute, University Health Network, Toronto, ON, Canada; ^2^Department of Physiology, University of Toronto, Toronto ON, Canada; ^3^Department of Neuroscience, Yale School of Medicine, New Haven, CT, 06520, USA; ^4^Department of Medicine (Neurology), University of Toronto, Toronto ON, Canada

##### **Correspondence:** Alexandra Pierri Chatzikalymniou - alexandra.chatzikalymniou@mail.utoronto.ca

*BMC Neuroscience* 2016, **17(Suppl 1)**:P58

**Fig. 38:**
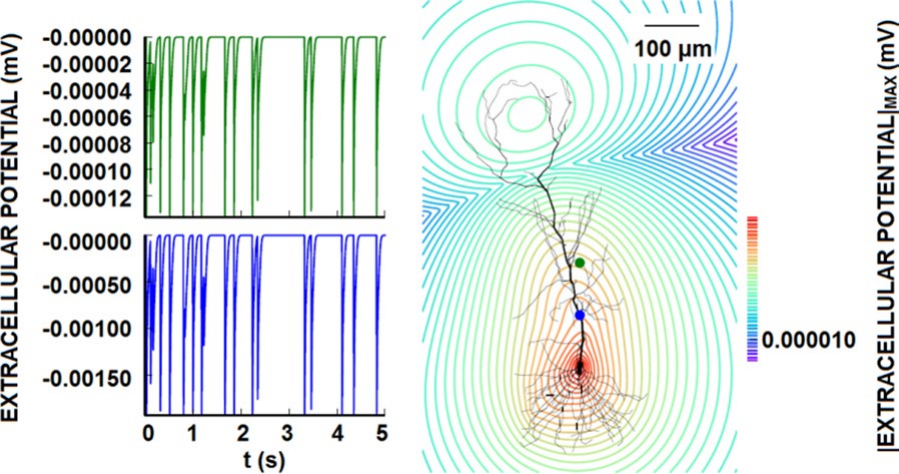
Example of the spatial attenuation of the extracellular potential signal for a particular set of inhibitory connections. The temporal traces at two electrode locations are represented with blue and green dots accordingly. Average over the absolute maximum extracellular potential amplitudes is shown in 2D space. According to the schematic the rate of the extracellular signal spatial attenuation generated by the pyramidal cell is approximately 400 μ

Oscillatory local field potentials (LFPs) are extracellularly recorded potentials with frequencies of up to ~500 Hz. They are associated with a number of physiological functions in health and disease and complement the information obtained by analysis of spikes. Because multiple neuronal processes contribute to the LFP, the signal is inherently ambiguous and more difficult to interpret than spikes [1]. However, the biophysical origin of LFPs is well understood in the framework of volume conductor theory [4]. Using “LFPy” [3], a python package that implements this framework, we construct a pyramidal cell model in CA1 hippocampus which generates extracellular potentials. Our pyramidal cell model receives inhibitory synaptic input from four different types of CA1 interneuron populations. These interneuron models are taken from a previous, experimentally constrained inhibitory network model developed to understand spontaneous theta (4–12 Hz) rhythms as expressed in an intact hippocampus preparation [2]. We investigate the contribution of the different inhibitory cell type interactions to the extracellular potential. In our current model we placed a virtual electrode probe along the vertical axis of the pyramidal cell to record its output in a layer dependent manner. We identified distinct regimes where specific interneuron cell type interactions distinctively affect the polarity, amplitude and frequency of the LFP signal (Fig. 38). We also distinguish between regimes where synaptic connection strengths preserve the extracellular potential frequency versus those that lead to lag or abolishment of the extracellular rhythm. In this way, our model helps us understand the cellular contributions to extracellular field patterns that arise in experimental recordings as a function of biologically relevant network states when the efficacy of inhibitory connections dynamically varies.

**Acknowledgements:** Supported by NSERC Canada, U. of T. Fellowship P.S.L., and the SciNet HPC Consortium.

**References**

1. Buzsáki G, Anastassiou CA, Koch C. The origin of extracellular fields and currents—EEG, ECoG, LFP and spikes. Nat Rev Neurosci. 2012;13:407–20.
2. Ferguson KA, Huh CYL, Amilhon B, Williams S, Skinner FK. Network models provide insight into how oriens-lacunosum-moleculare (OLM) and bistratified cell (BSC) interactions influence local CA1 theta rhythms. Front Syst Neurosci. 2015;9:110
3. Lindén H, Hagen E, Leski S, Norheim ES, Pettersen KH, Einevoll GT. LFPy: a tool for biophysical simulation of extracellular potentials generated by detailed model neurons. Front Neuroinform. 2014;7:41.
4. Rall W, Shepherd GM. Theoretical reconstruction of field potentials and dendrodendritic synaptic interactions in olfactory bulb. J Neurophysiol. 1968;31:884–915.

## P59 Expansion recoding through sparse sampling in the cerebellar input layer speeds learning

### N. Alex Cayco Gajic^1^, Claudia Clopath^2^, R. Angus Silver^1^

#### ^1^Department of Neuroscience, Physiology and Pharmacology, University College London, London, UK; ^2^Department of Bioengineering, Imperial College London, London, UK

##### **Correspondence:** N. Alex Cayco Gajic - natasha.gajic@ucl.ac.uk

*BMC Neuroscience* 2016, **17(Suppl 1)**:P59

Feed-forward networks often have many more output neurons than input neurons. This is thought to enable them to project neuronal activity patterns into a higher dimensional space. When such expansion recoding is combined with sparse representations it provides a powerful way to increase the separation between activity patterns [1–4]. In the input layer of the cerebellar cortex, granule cells (GCs) integrate sensorimotor input from the less numerous mossy fibre afferents (MFs), with each GC sampling from only 2 to 7 local MFs. Recent work has revealed that this sparse sampling of input by GCs provides an optimal tradeoff between information transmission and sparsification over a range of activity levels [4]. Moreover, theories of cerebellar function have linked expansion recoding under sparse regimes to pattern separation and associative learning [5, 6]. However, the relationship between the feedforward excitatory synaptic connectivity and the learning performance is poorly understood.

To investigate how the number of MF inputs per GC affects the performance we simulated a model of an 80µ ball of the MF-GC feedforward layer with either random or clustered MF activation. The connectivity profile of the network was constrained with recent anatomical data [4]. MF stimulation was modeled as random binary patterns with varying levels of population activity and correlation, and GCs as high-threshold rectified linear units. We then measured the speed at which granule cell population activity can be used to classify random patterns via backpropagation learning as the number of synaptic inputs was varied. We found that the largest speedup of learning in the GC activity (compared to learning based on the MF inputs) occurred when each GC received only a few synaptic inputs. We probed this result by analyzing the eigenvalues of the covariance matrix of the population-level activity, finding that sparse sampling of MF inputs allows GCs to both expand and decorrelate MF activity patterns. Interestingly, this feature is robustly preserved even in the presence of clustered inputs. In summary, we find that sparse sampling combined with sparsification of activity allows GCs to optimize both pattern expansion and pattern decorrelation.

**Acknowledgement:** This research is funded by the Wellcome Trust.

**References**

1. Laurent G. Olfactory network dynamics and the coding of multidimensional signals. Nat Rev Neurosci. 2001;3:884–95.
2. Olshausen BA, Field DJ. Sparse coding of sensory inputs. Curr Opin Neurobiol. 2004;14:481–7.
3. Billings G, Piasini E, Lorincz A, Nusser Z, Silver A. Network structure within the cerebellar input layer enables lossless sparse encoding. Neuron. 2014;83:960–74.
4. Babadi B, Sompolinksy H. Sparseness and expansion in sensory representations. Neuron. 2014;83:1–14.
5. Marr D. A theory of cerebellar cortex. J Physiol. 1969;202:437–70.
6. Tyrell T, Wilshaw D. Cerebellar cortex: its simulation and the relevance of Marr’s theory. Philos Trans R Soc B. 1992;336:239–57.

## P60 A set of curated cortical models at multiple scales on Open Source Brain

### Padraig Gleeson^1^, Boris Marin^1^, Sadra Sadeh^1^, Adrian Quintana^1^, Matteo Cantarelli^2^, Salvador Dura-Bernal^3^, William W. Lytton^3^, Andrew Davison^4^, R. Angus Silver^1^

#### ^1^Department of Neuroscience, Physiology and Pharmacology, University College London, London, UK; ^2^Metacell LLC, San Diego, CA, USA; ^3^State University of New York Downstate Medical Center, Brooklyn, NY, USA; ^4^Neuroinformatics group Unité de Neurosciences, Information et Complexité, CNRS, Gif sur Yvette, France

##### **Correspondence:** Padraig Gleeson - p.gleeson@ucl.ac.uk

*BMC Neuroscience* 2016, **17(Suppl 1)**:P60

Computational models of spiking cortical networks are implemented using a variety of approaches from large scale models with simplified point neurons and anatomically inspired connectivity, to networks on smaller scales with morphologically and biophysically detailed neurons. In between these scales many published models have used intermediate representations of neurons (e.g. conductance based with one compartment or abstract morphologies). These studies, and the associated modelling scripts, provide many potential starting points for experimental and theoretical neuroscientists wishing to use biologically constrained cortical models in their investigations. In addition, there are an increasing number of public neuroinformatics resources which are providing structured experimental data on the electrophysiology, connectivity and morphology of cortical neurons. While these modelling and experimental resources should lead to a proliferation in well constrained cortical models there remain a number of practical and technical barriers to more widespread development and use of such models among researchers.

The Open Source Brain (OSB) initiative (http://www.opensourcebrain.org) is a resource for collaborative development of models in computational neuroscience. Sharing of models in standardised representations such as NeuroML 2 [1] and PyNN is encouraged and actively supported on the site. Conversion of cell and network models to NeuroML allows them to be visualised and analysed in 3D in a standard web browser through the OSB website. We have recently added a feature to allow simulations to be executed on our servers (e.g. by conversion to NEURON) and the results displayed within the browser. We have been actively converting published cortical models to NeuroML format and making these available on OSB. These range from point neuron models [2, 3], to abstract [4] and detailed [5] multicompartmental models. We are working to develop these and others into a curated set of cortical models in a common format which can be used as the basis for new models. We have also developed frameworks for importing resources from neuroinformatics datasets such as the Allen Institute Cell Types database (http://celltypes.brain-map.org) and NeuroMorpho.org (http://neuromorpho.org). We have greatly improved compatibility between PyNN and NeuroML, allowing the modeller freedom to choose between procedural (Python) and declarative (XML) model specification. We have also extended a model optimisation framework (https://github.com/NeuralEnsemble/NeuroTune) facilitating generation of new NeuroML models from electrophysiological data. All of this work is aimed at making existing cortical models easier to access, visualise and simulate, simplifying development of new models based on these prototypes, and ensuring the latest experimental datasets can be used to constrain and validate complex models of cortical function.

**Acknowledgements:** This work has been primarily funded by the Wellcome Trust (101445/095667).

**References**

1. Cannon RC, Gleeson P, Crook S, Ganapathy G., Marin B, Piasini E, Silver RA. LEMS: a language for expressing complex biological models in concise and hierarchical form and its use in underpinning NeuroML2. Front Neuroinform. 2014;8:79.
2. Izhikevich E. Simple model of spiking neurons. IEEE Trans Neural Netw. 2003;14(6):1569–72.
3. Brunel N. Dynamics of sparsely connected networks of excitatory and inhibitory spiking neurons. J Comput Neurosci. 2000;8(3):183–208.
4. Traub RD, Contreras D, Cunningham MO, et al. Single-column thalamocortical network model exhibiting gamma oscillations, sleep spindles, and epileptogenic bursts. J Neurophysiol. 2005;93(4):2194–2232.
5. Markram H, Muller E, Ramaswamy S, et al. Reconstruction and simulation of neocortical microcircuitry. Cell. 2015;163(2):456–92.

## P61 A synaptic story of dynamical information encoding in neural adaptation

### Luozheng Li^1^, Wenhao Zhang^1^, Yuanyuan Mi^1^, Dahui Wang^1,2^, Si Wu^1^

#### ^1^State Key Laboratory of Cognitive Neuroscience & Learning, IDG/McGovern Institute for Brain Research, Beijing Normal University, Beijing 100875, China; ^2^School of System Science, Beijing Normal University, Beijing 100875, China

##### **Correspondence:** Si Wu - wusi@bnu.edu.cn

*BMC Neuroscience* 2016, **17(Suppl 1)**:P61

**Fig. 39:**
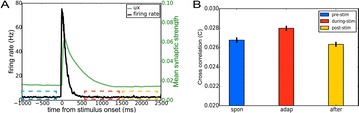
Firing rates, synaptic efficacy and cross-correlation change during the adaptation. **A** The time course of firing rates and the averaged synaptic efficacy of the network during the adaptation. $\left\langle {\text{ux}} \right\rangle$ is temporally enhanced during the adaptation due to the STF, but in the long term, strong STD drives the synaptic efficacy to background level. Stimulation is during 0–1500 ms. **B** The enhancement of cross-correlation between neurons during the adaptation

Adaptation refers to the general phenomenon that a neural system dynamically adjusts its response property according to the statistics of external inputs [1]. In response to a prolonged constant stimulation, neuronal firing rates always first increase dramatically at the onset of the stimulation; and afterwards, they decrease rapidly to a low level close to background activity (see Fig. 39A). This attenuation of neural activity seems to be contradictory to our experience that we can still sense the stimulus after the neural system is adapted [2]. Thus, it prompts a question: where is the stimulus information encoded during the adaptation? Here, we investigate a computational model in which the neural system employs a dynamical encoding strategy during the neural adaptation: at the early stage of the adaptation, the stimulus information is mainly encoded in the strong independent firings; and as time goes on, the information is shifted into the weak but concerted responses of neurons (see Fig. 39B). We find that short-term plasticity [3], a general feature of synapses, provides a natural mechanism to achieve this goal. Furthermore, we demonstrate that with balanced excitatory and inhibitory inputs, this correlation-based information can be read out efficiently. The implications of this study on our understanding of neural information encoding are discussed.

**Conclusions** We have explored a dynamical encoding strategy in neural adaptation. By constructing a computational model, we show that this can be achieved through varying the information encoder during the adaptation, that is, at the early stage of the adaptation, the stimulus information is mainly encoded in the strong and independent firings of neurons; and as time goes on, the stimulus information is shifted into the weak but concerted responses of neurons. This shift of information encoder can be naturally implemented via STP, a general feature of synapses.

**References**

1. Wark B, Lundstrom B N, Fairhall A. Sensory adaptation. Curr Opin Neurobiol. 2007;17(4):423–9.
2. Christopher deCharms R, Merzenich MM. Primary cortical representation of sounds by the coordination of action-potential timing. Nature. 1996;381:13.
3. Markram H, Wang Y, Tsodyks M. Differential signaling via the same axon of neocortical pyramidal neurons. Proc Natl Acad Sci. 1998;95(9):5323–28.

## P62 Physical modeling of rule-observant rodent behavior

### Youngjo Song^1^, Sol Park^1,2^, Ilhwan Choi^2^, Jaeseung Jeong^1^, Hee-sup Shin^2^

#### ^1^Bio and Brain Engineering, KAIST, Daejeon, 34141, Republic of Korea; ^2^Center for Cognition and Sociality, IBS, Daejeon, 34047, Republic of Korea

##### **Correspondence:** Jaeseung Jeong - jsjeong@kaist.ac.kr

*BMC Neuroscience* 2016, **17(Suppl 1)**:P62

There is training room for a mouse. In the room, there are two lights which is used as a cue for reward, and there are two reward zone. If a mouse go left reward zone when the left light cue turns on, the mouse gets reward, and if a mouse go right reward zone when the right light cue turns on, the mouse gets reward. The reward is given by brain stimulation from the electrode implanted in MFB. A pair of mice trained individually in the room in order to make them understand the meaning of two light cues. After individual training, the two mice released in the same training room at the same time. In this experiment, 15 out of 19 pairs showed tendency to separate their own reward zone.

In order to explain the rodent behavior, we made a computational rodent model. This model is based on Rescorla–Wagner Model. We defined a success rate as probability that a mouse is in the correct reward zone when any cue is given. In each trial, the model mouse learns the left success rate and the right success rate by reinforcement learning. In this model, we assume that success rates for the left cue and right cue are independent and we eliminate social interaction between two model mice.

**Fig. 40:**
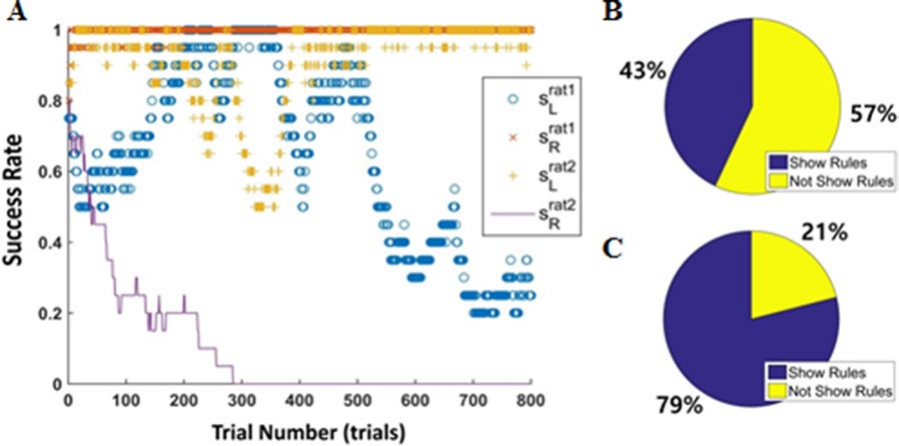
**A** Success rate of two model rats which shows rule between them (*blue dots* represent the left cue success rate of model rat1, orange plus represent the right cue success rate of model rat1, *yellow cross* represent the left cue success rate of model rat2, and *purple line* represent the right cue success rate of model rat2). **B** Simulation result (600 iterations). **C** Behavior experiment result (19 pairs)

**Results** The simulation result is given in below figures. Figure 40A is a graph of success rate of a pair of model mice which shows rule between them. You can see the right que success rate of mouse1 and the left que success rate of mouse2 converge to 1, but the left que success rate of mouse1 and the right que success rate of mouse2 converge below 0.4. It means that mouse1 tends to move only when the right cue is given, and mouse2 tends to move only when the left cue is given. Our model mice, however, shows this rule with less probability than actual behavior result. Figure 40B, C 43 % of model mice showed rule, but 79 % of actual mice showed rule in behavior experiment.

**Conclusion** If we assume the mouse as a simple independent creature which doesn’t have social element, they show rules with lower probability than the actual mouse in behavior experiment. It means that we can’t regard the mouse as a non-social creature. Therefore, we have to add the social factors such as empathy or cooperation to simulate actual rodent behavior.

**Reference**

1. Glimcher PW, Fehr E. Neuroeconomics, 2nd ed. Academic Press.

## P64 Predictive coding in area V4 and prefrontal cortex explains dynamic discrimination of partially occluded shapes

### Hannah Choi^1,2,3^, Anitha Pasupathy^2,3^, Eric Shea-Brown^1,3^

#### ^1^Department of Applied Mathematics, University of Washington, Seattle, WA 98195, USA; ^2^Department of Biological Structure, University of Washington, Seattle, WA 98195, USA; ^3^UW Institute for Neuroengineering, University of Washington, Seattle, WA 98195, USA

##### **Correspondence:** Hannah Choi - hannahch@uw.edu

*BMC Neuroscience* 2016, **17(Suppl 1)**:P64

**Fig. 41:**
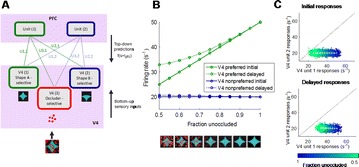
**A** Schematic of the V4-PFC network model. **B** Optimal representation of the shape-selective V4 responses as a function of occlusion level. **C** Neuronal responses with a noise projected onto the test shape-selective (unit 1)/non-selective (unit 2) V4 response plane, before (*top*) and after (*bottom*) the feedback inputs from PFC. Feedback inputs move the responses away from the unity line, improving shape discriminability under occlusion

The visual system recognizes objects in natural scenes without difficulty, even when most objects are partially occluded. The neural basis of this capacity is unknown. Recent results from primate area V4, an intermediate stage in the shape processing pathway, suggest that feedback from higher cortices may be important for the emergence of V4 shape selective signals [1] when animals are engaged in discriminating partially occluded shapes. Here we implement predictive coding, which has been previously applied to explain responses in early visual areas [2], to investigate possible mechanisms underlying robust discrimination of partially occluded shapes in V4. We propose that higher cortical areas such as prefrontal cortex (PFC) make predictions about V4 activities; when these PFC signals are relayed via feedback to V4, they can reproduce the delayed peak of V4 responses observed in experiments. With a model (Fig. 41A) composed of PFC and V4 units that are selective for different input features, we capture response characteristics of V4 and PFC measured in experiments, by combining feed-forward sensory inputs and feedback predictions to maximize the posterior probability of the responses. We found that inclusion of the feedback predictions results in stronger shape-selective responses across a range of occlusion levels (Fig. 41B), thus maintaining robust discrimination of partially occluded shapes (Fig. 41C).

**Acknowledgements:** This research was supported by the Washington Research Foundation Innovation Postdoctoral Fellowship in Neuroengineering (HC), National Science Foundation CRCNS Grant IIS1309725 (AP), and NEI Grant R01EY018839 (AP).

**References**

1. Kosai Y, El-Shamayleh Y, Fyall AM, Pasupathy A. The role of visual area V4 in the discrimination of partially occluded shapes. J Neurosci. 2014;34:8570–84.
2. Rao RPN, Ballard DH. Predictive coding in the visual cortex: a functional interpretation of some extra-classical receptive-field effects. Nature Neurosci. 1999;2:79–87.

## P65 Stability of FORCE learning on spiking and rate-based networks

### Dongsung Huh^1^, Terrence J. Sejnowski^1,2^

#### ^1^The Salk Institute for Biological Studies, La Jolla, CA 92037 USA; ^2^Division of Biological Sciences, University of California at San Diego, La Jolla, CA 92095 USA

##### **Correspondence:** Dongsung Huh - huh@salk.edu

*BMC Neuroscience* 2016, **17(Suppl 1)**:P65

Neurons in the brain often exhibit complex activity patterns, with fluctuations on time scales of several seconds. The generation of complex patterns is critical for directing movements, and is likely to be involved in processing time-varying input (such as speech). However, it is not yet understood how networks of spiking neurons, with time constants of only a few milliseconds, could exhibit such slow dynamics. This should be contrasted with rate-based neural networks, which can be easily trained to generate arbitrary complex activity patterns in a reservoir-based manner [1] by an iterative training method (FORCE learning [2]). So far, however, FORCE learning has not led to successful training of spiking neural networks.

Here, we analyze the stability of the networks that result from such learning schemes. For linear rate-based networks, we can analytically predict the full dynamic property of the networks. As the network’s recurrent connectivity reaches the “edge of chaos”, the neuronal activity exhibits a broad distribution of phase, providing appropriate basis for generating the fluctuations. For weaker recurrent connectivity, however, the phase distribution becomes much narrower. In this case, the trained network exhibits highly non-normal structure, which becomes unstable even under small perturbations. Our analysis also illuminates the source of instability in training spiking networks, which is mainly due to the rectified nature of the neuronal output. In numerical simulations, rectified-linear rate networks exhibit narrow phase distribution, even with strong recurrent connectivity near the edge of chaos. Moreover, introducing spiking-dynamics further reduces the width of the distribution, leading to highly unstable network dynamics. Our result reveals the limitation of the reservoir-based approaches, and may lead to more stable, alternative training methods.

**Acknowledgements:** Supported by HHMI.

**References**

1. Jaeger H, Haas H. Harnessing nonlinearity: predicting chaotic systems and saving energy in wireless communication. Science. 2004;304:78–80.
2. Sussillo D, Abbott LF. Generating coherent patterns of activity from chaotic neural networks. Neuron. 2009;63(4):544–57.

## P66 Stabilising STDP in striatal neurons for reliable fast state recognition in noisy environments

### Simon M. Vogt^1^, Arvind Kumar^2,3^, Robert Schmidt^1,2^

#### ^1^BrainLinks-BrainTools, Cluster of Excellence, University of Freiburg, Germany; ^2^Faculty of Biology and Bernstein Center Freiburg, University of Freiburg, Germany; ^3^Department of Computational Biology, Royal Institute of Technology Stockholm, Sweden

##### **Correspondence:** Simon M. Vogt - simonsunimail@gmail.com

*BMC Neuroscience* 2016, **17(Suppl 1)**:P66

The brain must be able to quickly identify environmental states based on sensory inputs and select appropriate actions. To obtain neurons that respond selectively to states with short response latencies, a neural plasticity rule is required. Spike timing dependent plasticity (STDP) is known to find the earliest predictors of spatiotemporal spike patterns without supervision. While this feature of STDP is often seen as a hindrance when aiming to reproduce an exact target output spike train, we exploit it to generate short-latency, reliable pattern recognition.

There are, however, some difficulties in using STDP in real-world continuous scenarios. In models, presynaptic firing rates are often assumed to be stationary or have constant correlation for simplification, and STDP rule parameters must be closely fitted to form a stable fixed point in the postsynaptic firing rate and normalise postsynaptic activity. Any deviation from these requirements can cause the postsynaptic neuron to become quiet before it is able to form strong selectivity, or exhibit a runaway effect that makes the neuron responsive to a large set of inputs. Soft-bound STDP with a weight-dependent attractor has been suggested as a means for stabilising postsynaptic activity, but this actively hinders separation of spatiotemporal patterns from background noise: as during learning the synaptic weight moves away from the attractor, noisy input becomes more likely to undo these weight changes. Furthermore, activity-dependent scaling also aims to keep the postsynaptic neuron active indefinitely, so neurons may lose any learned selectivity when enduring long periods of background noise. Interpolations between different types of STDP have been suggested, but a problem of balance between premature selectivity and longtime noise robustness remains.

We solve this dilemma by including a slow continuous potentiation in our model, which depends on metabolic cost of maintaining strong synapses and slowly vanishes as neurons become selective. It is independent of pre- or postsynaptic activity and can recover silent neurons if a metabolic cost function allows it to. Together with negative-integral STDP as taken from experimental data, it stabilises the activity of untrained neurons for a much wider range of rule parameters and heterogeneous input activity. Our model maintains a unimodal weight distribution while the postsynaptic neuron has not yet become selective, but does not impair the formation of selectivity to spatiotemporal patterns. Selectivity is quickly achieved as soon as patterns are present, even after enduring long periods of noise. Connections that only present noise, represent only other patterns, or present only late parts of a trained pattern become ineffective as the neuron becomes selective, and may be pruned. Any selectivity is hence ensured to represent actual spatiotemporal spike patterns that were at some point present in the postsynaptic neuron’s inputs. This makes the process of training neurons to detect environmental states encoded as spatiotemporal patterns more robust to variations in input statistics and rule parameters, thus easing application in larger-scale networks.

Our model of fast pattern detection may apply specifically to the striatum of the basal ganglia where fast reliable decisions need to be made within milliseconds. Unsupervised learning should coexist with rare dopaminergic reinforcement to continuously form new representations of environmental events and decide which of these events are behaviourally important and which can safely be ignored.

**Acknowledgements:** Cluster of Excellence BrainLinks-BrainTools funded by German Research Foundation (DFG, Grant Number EXC 1086).

## P67 Electrodiffusion in one- and two-compartment neuron models for characterizing cellular effects of electrical stimulation

### Stephen Van Wert^1^, Steven J. Schiff^1,2^

#### ^1^Center for Neural Engineering, Department of Engineering Science and Mechanics, The Pennsylvania State University, University Park, PA 16802, USA; ^2^Departments of Neurosurgery and Physics, The Pennsylvania State University, University Park, PA 16802, USA

##### **Correspondence:** Stephen Van Wert - szv124@psu.edu

*BMC Neuroscience* 2016, **17(Suppl 1)**:P67

Standard approaches for modeling the neuronal effects of electrical fields and currents (such as [1, 2]) apply transmembrane current to Hodgkin–Huxley membrane patches without regard to ion fluxes and conservation of ions inside and outside the cell. We propose cellular models that reflect polarization and preserve the biophysics of the spaces the neurons are embedded within. By including ion fluxes and maintaining conservation of mass and charge, the gradients of ionic concentrations both within and outside of the neuron can be accounted for. This requires characterizing the ionic fluxes with electrodiffusion, such that ionic charge gradients as well as ionic concentration gradients drive flux. This electrodiffusion mechanism, derived from Nernst-Planck flux equation, not only allows for more accurate modeling of physiological and pathophysiological conditions with substantial ionic and volume changes, but it also provides a means to model application of electrical stimulation. The model being developed here builds on recent one-compartment model development in [3] that extends the Hodgkin–Huxley formalism in several distinct ways, most notably in using conservation to track all ion fluxes and volume changes to determine the extra- and intra-cellular concentrations. The proposed model also extends the model in [3] to a two compartment model which allows for simulation of neuronal polarization with control applied in the direction of a soma-dendritic axis.

We first characterize the resultant model dynamics in the absence of any control stimulus and compare these to the dynamics seen with standard diffusion. In particular, the dynamics are characterized through trajectories of dynamically evolving variables, with a focus on bifurcation structure at points where the dynamics transition from different states such as normal firing, seizure, or spreading depression. We then simulate the effects of applying excitatory or inhibitory control on these dynamics and optimize the dynamics of the neuron to be consistent with experimental evidence. Such a model gives very different results from the customary approach to modeling the effects of electrical stimulation, where the stimulation is applied internally or externally to a neuron without taking the nature of the charge carriers present into account. There are a variety of effects of excitatory and inhibitory stimulation observed now that could not be possible before, and we can describe the trajectories of the effects of such stimulation in ways that shed light on multiple experimental scenarios.

This type of model development offers the ability to understand a wide variety of previously unexplained experimental observations for both excitatory and inhibitory stimulation. Doing so offers a platform for us to study electrical feedback control of neuronal systems and to offer model-based control strategies for pathological dynamics such as seizures and spreading depression.

**References**

1. Park E-H, Barreto E, Gluckman BJ, Schiff SJ, So P. A model of the effects of applied electric fields on neuronal synchronization. J Comput Neurosci. 2005;19: 53–70.
2. Berzhanskaya J, Chernyy N, Gluckman BJ, Schiff SJ, Ascoli GA. Modulation of hippocampal rhythms by subthreshold electric fields and network topology. J Comput Neurosci. 2013;34(3):369–89.
3. Wei Y, Ullah G, Schiff SJ. Unification of neuronal spikes, seizures, and spreading depression. J Neurosci. 2014;34(35):11733–43.

## P68 STDP improves speech recognition capabilities in spiking recurrent circuits parameterized via differential evolution Markov chain Monte Carlo

### Richard Veale^1^, Matthias Scheutz^2^

#### ^1^National Institute for Physiological Sciences, Okazaki, Aichi, Japan; ^2^Department of Computer Science, Tufts University, Medford, MA, USA

##### **Correspondence:** Richard Veale - richard@nips.ac.jp

*BMC Neuroscience* 2016, **17(Suppl 1)**:P68

**Fig. 42:**
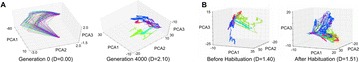
**A** Fitness evolution generation 0–4000 (first 3 principle components). *Each color* is a different word class, each line a different utterance token of the word. **B** Change in state space trajectory from STDP adaptation

A major issue in using spiking neural circuits for pragmatic tasks such as speech recognition is how to parameterize them. Here, we apply a hybrid Differential Evolution/Markov Chain Monte Carlo (DE/MCMC) [1, 2] approach to estimate optimal parameters for a spiking neural circuit that is used for real time speech recognition [3] from raw auditory input using PSWEEP2 (rveale.com/software.php). To avoid the expensive training step, we use a surrogate measure of word recognition performance. Specifically, we maximize average within-word similarity in the neural circuit’s state space trajectory, while simultaneously minimizing between-word similarity. We executed the algorithm for 7000 generations (48 h of runtime) using 2016 cores of the super computer Big Red II at Indiana university. The average fitness increases significantly with successive generations. Panel a is a visualization of the first 3 principle components of the state space for the exemplars of each different word category (shown as different colors). The different categories move to take more distant trajectories through the state space with successive generations. We verify that the state-space separation is a good surrogate measure of word recognition performance by taking the set of circuits with the highest fitness from the first, middle, and last 100 generations and training readout neurons for the 7-word corpus. Word recognition performance increases from 19 to 71 to 85 % (Fig. 42).

Finally, we evaluated the performance benefit of adaptation to sensory stimuli via synaptic plasticity mechanisms, to make pragmatic use of our previous work investigating auditory habituation [4]. We take the most performant parameter points that were found during the parameter sweep, and test their fitness before and after exposure to 100 presentations of the word stimuli while a nearest-neighbor temporally asymmetric Hebbian plasticity model of spike timing dependent plasticity (STDP) is implemented in all excitatory synapses. Although sensitive to STDP model parameters, Panel B shows that word recognition performance can be improved by as much as 8 % by familiarizing a neural circuit to the type of sensory stimulus that it will be used to compute. This follows previous findings by Triesch et al. [5], who reported similar effects in non-spiking neural networks.

**References**

1. Veale R, Isa T, Yoshida M. Applying differential evolution MCMC to parameterize large-scale spiking neural simulations. In: IEEE conference on evolutionary computation (CEC). IEEE. p. 1620–27.
2. Laloy E, Vrugt JA. High-dimensional posterior exploration of hydrologic models using multiple-try dream (zs) and high-performance computing, Water Resour Res. 2012;48(1).
3. Veale R, Scheutz M. Neural circuits for any-time phrase recognition. In: Proceedings of the 34th annual conference of the Cognitive Science Society; 2012. p. 1072–7.
4. Veale R, Scheutz M. Auditory habituation via spike-timing dependent. In: Proceedings of the international conference on development and learning and epigenetic robotics (ICDL), San Diego, CA; 2012.
5. Lazar A, Pipa G, Triesch J. Fading memory and time series prediction in recurrent networks with different forms of plasticity. Neural Netw. 2007;20(3):312–22.

## P69 Bidirectional transformation between dominant cortical neural activities and phase difference distributions

### Sang Wan Lee^1,2,3^

#### ^1^Department of Bio and Brain Engineering, Korea Advanced Institute of Science and Technology (KAIST), Daejeon, South Korea; ^2^Program of Brain and Cognitive Engineering, Korea Advanced Institute of Science and Technology (KAIST), Daejeon, South Korea; ^3^KAIST Institute for Health Science and Technology, Korea Advanced Institute of Science and Technology (KAIST), Daejeon, South Korea

##### **Correspondence:** Sang Wan Lee - sangwan@kaist.ac.kr

*BMC Neuroscience* 2016, **17(Suppl 1)**:P69

The brain is a complex nonlinear dynamic system comprising multiple different types of subsystems. Each subsystem encodes different types of information, and its states are context-dependent. Unlike sensory or motor processing occurring at relatively early or terminal stages of functional hierarchy, cognitive processes, including learning, inference, and top-down attention, require interactions between brain’s multiple subsystems. The associated neural dynamics inevitably leave an imprint on neural activity patterns over a wide areas of cortex.

Considerable progress has been made toward understanding such functional network dynamics. This includes the causal connectivity [1], its extension to distinguish causality from correlation within nonseparable weakly connected dynamic systems [2], and the integrated information theory to quantify the effect of a neuronal network connectivity on the increase in the amount of information above and beyond the capability of a single locally connected network [3, 4]. However, none of these methods is applicable to real-time analyses when the network size is large.

**Fig. 43:**
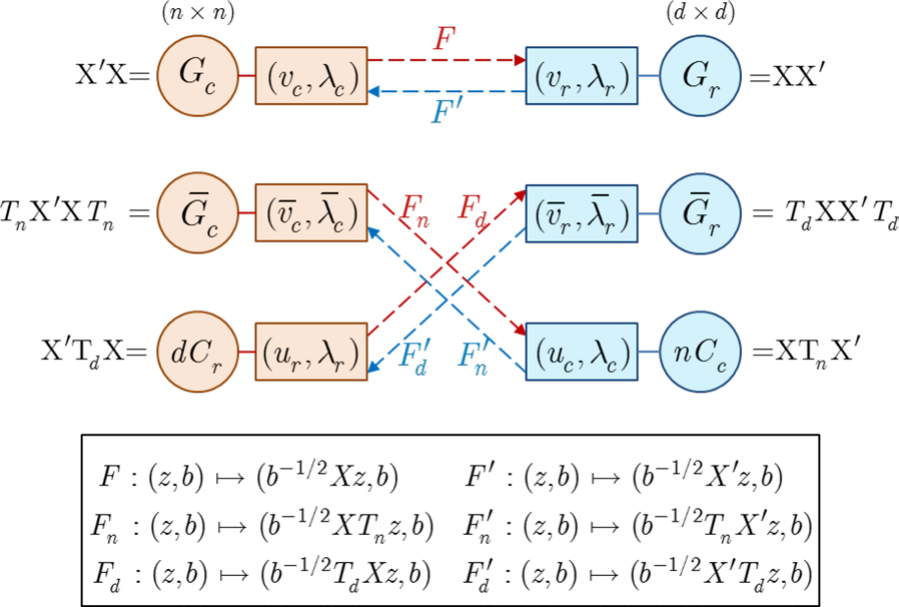
Computational framework for analyzing space–time cortical dynamics. *T* is an idempotent projection matrix

Here I develop a simple and efficient computational framework for analyzing cortical dynamics both in time and space, arising from complex interactions between brain’s multiple subsystems. Accommodating the fact that both a covariance and a gram matrix can be computed by using a combination of a certain idempotent matrix with a data matrix, I derive a set of matrix operators *F*, with which one set of eigenvectors associated with a covariance matrix of data and of mean-corrected data can be transposed to another set of eigenvectors associated with a gram matrix, and vice versa (see Fig. 43). For example, suppose that a *d*-by-*n* data matrix is a set of time-series data recorded from multiple locations of cortices where *d* and *n* refers to the number of electrodes and time points, respectively, and $d \ll n.$ One can then perform a singular value decomposition (SVD) for the *d*-by-*d* covariance or gram matrix to obtain an associated eigenvector set, followed by applying matrix operator *F*′ to the eigenvector set to convert it to the eigenvector set of their counterparts. There is no need to perform SVD for *n*-by-*n* matrix whose computational load is high. If $n \ll d,$ then one could simply start with performing the SVD for the *n*-by-*n* matrix, followed by applying the matrix operator *F* to its eigenvector set. It is noted that the acquired *d*-by-*1k* eigenvectors and *n*-by-1*k* eigenvectors correspond to dominant cortical neural activities and phase difference distributions, respectively.

**Conclusion** An efficient computational framework for analyzing cortical dynamics both in time and space is proposed, taking into account the relationship between a covariance and a gram matrix. For analyzing neural data acquired from multiple locations of cortices, the framework replaces the SVD with a simple matrix operator *F* so as to reduce a heavy computational load of performing SVD on large-size data matrices. In doing so, it allows efficient bidirectional transformation between dominant neural activities and phase difference distributions.

**Acknowledgements:** I thank Barclay Lee and Dae-Hyun Kim for their assistance. This work was supported by the research fund of the KAIST (Korea Advanced Institute of Science and Technology) (Grant code: G04150045).

**References**

1. Seth AK. Causal connectivity of evolved neural networks during behavior. Network. 2005;16:35–54.
2. Sugihara G, May R, Ye H, Hsieh C, Deyle E, Fogarty M, Munch S. Detecting causality in complex ecosystems. Science. 2012;338(6106):496–500.
3. Balduzzi D, Tononi G. Qualia: the geometry of integrated information. PLoS Comput Biol. 2009;5(8):e1000462.
4. Edlund JA, Chaumont N, Hintze A, Koch C, Tononi G, Adami C. Integrated information increases with fitness in the evolution of animats. PLoS Comput Biol. 2011;7(10):e1002236.

## P70 Maturation of sensory networks through homeostatic structural plasticity

### Júlia Gallinaro^1^, Stefan Rotter^1^

#### ^1^Bernstein Center Freiburg and Faculty of Biology, University of Freiburg, Freiburg, Baden-Württember, 79194, Germany

##### **Correspondence:** Simon M. Vogt - julia.gallinaro@bcf.uni-freiburg.de

*BMC Neuroscience* 2016, **17(Suppl 1)**:P70

Neurons in the adult visual cortex of mice prefer to make synapses with neurons responding to similar visual features. As such a bias in connectivity is not observed at the time of eye opening, it has been proposed that the functional subnetworks are formed through rewiring of recurrent synaptic connections, induced by visual experience [1]. However, it is not clear according to which rules this structure develops. The emergence of feature specific wiring was recently demonstrated in a balanced network model with appropriate rules of functional synaptic plasticity [2]. In this model, however, connectivity was evaluated based on the strength of already existing synapses, and the structure of the network remained unchanged throughout the simulation.

**Fig. 44:**
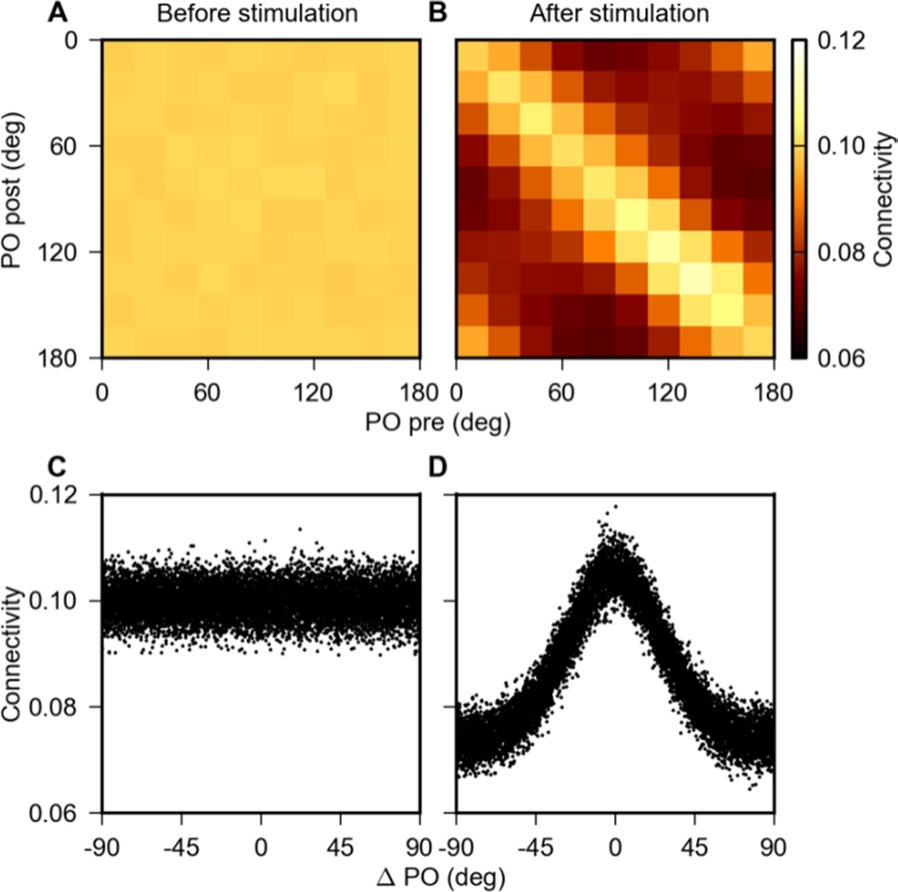
Network connectivity before and after sensory stimulation. **A**, **B**. Connectivity matrix, pre- and post-synaptic neurons are sorted according to their preferred orientation (PO) and subdivided into groups. **C**, **D**. Mean output connectivity plotted against the difference between pre and post PO

Referring to recent findings of homeostatic regulation of cortical activity in rodent visual cortex in vivo [3], we employ here a structural plasticity rule based on firing rate homeostasis described previously [4] for simulating network restructuring during sensory stimulation. We show that, next to other biologically meaningful properties, feature specific connectivity also emerges in a balanced network of changing structure (see Fig. 44), using a plasticity rule that does not depend on spike timing.

**Acknowledgements:** Supported by the Erasmus Mundus Joint Doctoral program EuroSPIN, the German Federal Ministry of Education and Research, grant 01GQ0830, and the state of Baden-Württemberg through bwHPC.

**References**

1. Ko H, Cossell L, Baragli C, Antolik J, Clopath C, Hofer SB, Mrsic-Flogel TD: The emergence of functional microcircuits in visual cortex. Nature. 2013;496:96–100.
2. Sadeh S, Clopath C, Rotter S. Emergence of functionalspecificity in balanced networks with synapticplasticity. PLoS Comput Biol. 2015;11:e1004307.
3. Bishop HI, Zito K. The downs and ups of sensorydeprivation: evidence for firing rate homeostasis in vivo. Neuron. 2013;80:247–9.
4. van Ooyen A: Using theoreticalmodels to analyseneuraldevelopment. Nat Rev Neurosci. 2011;12:311–26.

## P71 Corticothalamic dynamics: structure, number of solutions and stability of steady-state solutions in the space of synaptic couplings

### Paula Sanz-Leon^1,2^, Peter A Robinson^1,2^

#### ^1^School of Physics, University of Sydney, New South Wales, Australia; ^2^Center for Integrative Brain Function, University of Sydney, New South Wales, Australia

##### **Correspondence:** Paula Sanz-Leon - paula.sanz-leon@sydney.edu.au

*BMC Neuroscience* 2016, **17(Suppl 1)**:P71

**Fig. 45:**
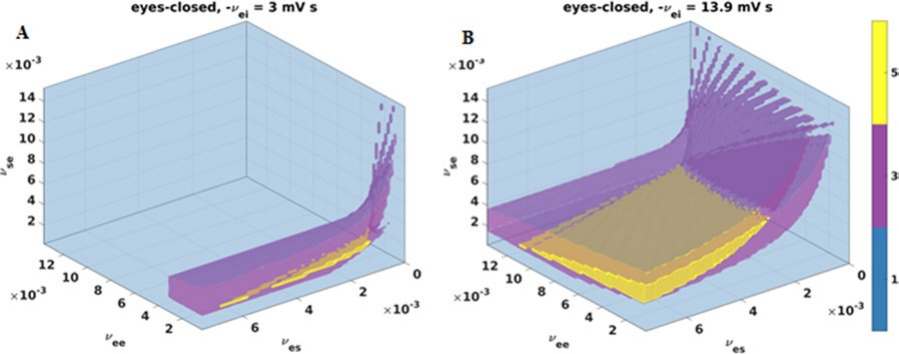
Three dimensional subsets of the 8D corticothalmic coupling space. **A**, **B** Regions with 1, 3 or 5 roots are enclosed by surfaces (*blue*, *violet* and *yellow* respectively). The sharp transition between zones of 1–3 roots along the v_ee_ axis (excitatory intracortical feedback) indicates the plane at which the total intracortical feedback (v_ee_ + v_ei_) changes sign. The difference between **A**, **B** is the value of v_ei_ (inhibitory intracortical feedback). In **a** the probability of having multiple roots is lower than in **B**

The interconnections of a model of the corticothalamic system [1] define an 8-dimensional parameter space where specific combinations of dimensions correspond to one of the three loops of the system (e.g., intracortical, corticothalamic and intrathalamic). The form of the steady-state equation of the corticothalamic system imposes an odd number of solutions, which in terms of dynamics correspond to fixed points of the system. Here, the structure of regions with different number of solutions is systematically investigated within physiologically valid ranges of synaptic couplings representing different brain states [2, 3]. For instance, Fig. 45A, Bdisplay the regions where the steady state equation has one, three or five solutions for two 3-dimensional subsets of the full space. These results show how small changes in the connectivity can cause additional roots of the steady state equation to appear or vanish. More importantly, they illustrate the effect of intracortical feedback: for more than one solution to exist the total intracortical feedback needs to be negative (inhibitory). The occurrence of multiple roots happens for parameter values that characterize normal arousal states [3], indicating that the approach presented here has a potential to (i) quantify and predict the existence of additional (abnormal) arousal states and (ii) categorize subtle differences in states such as anesthesia, coma [4].

**References**

1. Robinson PA, Rennie CJ, Wright JJ, Bourke PD. Steady states and global dynamics of electrical activity in the cerebral cortex. Phys Rev E. 1998;58:3557–71.
2. Robinson PA, Rennie CJ, Wright JJ, Bahramali H, Gordon E, Rowe DL. Prediction of electroencephalographic spectra from neurophysiology. Phys Rev E. 2001; 63:021903.
3. Abeysuriya RG, Rennie CJ, Robinson PA. Physiologically based arousal state estimation and dynamics. J Neurosci Methods. 2015;253:55–69.
4. Steyn-Ross ML, Steyn-Ross DA, Sleigh JW, Wilcocks LC. Toward a theory of the general-anesthetic-induced phase transition of the cerebral cortex. I. A thermodynamics analogy. Phys Rev E. 2001;64:942–53.

## P72 Optogenetic versus electrical stimulation of the parkinsonian basal ganglia. Computational study

### Leonid L. Rubchinsky^1,2^, Chung Ching Cheung^1^, Shivakeshavan Ratnadurai-Giridharan^1^

#### ^1^Department of Mathematical Sciences, Indiana University-Purdue University Indianapolis, Indianapolis, IN, USA; ^2^Stark Neurosciences Research Institute, Indiana University School of Medicine, Indianapolis, IN, USA

##### **Correspondence:** Leonid L. Rubchinsky - lrubchin@iupui.edu

*BMC Neuroscience* 2016, **17(Suppl 1)**:P72

Deep brain stimulation (DBS) is used as a therapeutic procedure to treat symptoms of several neurological and neuropsychiatric disorders. In particular it is used to treat motor symptoms of Parkinson’s disease (PD) by delivering high-frequency regular stimulation to subcortical targets. Hypokinetic symptoms of PD are associated with excessive oscillatory synchronized activity in the beta frequency band, and effective DBS is believed to suppress it. An alternative way to stimulate neural circuits is an emerging technology of optogenetics. It is an experimental technique and it is not clear if it eventually will be possible to implement it in clinical practice. However it is used as an experimental tool, and maybe, in time, it will be developed into safe therapeutic technique.

The goal of his study is to explore how effective an optogenetic stimulation in comparison with electrical stimulation in their network effects on elevated synchronized oscillatory activity. We use a model for the basal ganglia activity [1], which was developed to reproduce experimentally observed beta-band activity patterns [2]. We introduce electrical stimulation as well as optogenetic stimulation of two types: excitatory via channelrhodopsin and inhibitory via halorodopsin. We explore the effect of different stimulation types on oscillatory synchronized dynamics and consider the efficacy of stimulation for different kind of network’s dynamics.

All three modes of stimulation can decrease beta synchrony that is commonly associated with hypokinetic symptoms of Parkinson’s disease. Generally speaking, growing intensity of stimulation leads to larger suppression of the beta-band synchronized oscillatory activity. But the actions of different stimulation types on the beta activity may differ from each other. Electrical DBS and optogenetic excitation have somewhat similar effects on the network. Both of these stimulation types cause desynchronization and suppression of the beta-band bursting. As intensity of stimulation is growing, they synchronize the network at higher (non-beta) frequencies in a close to tonic spiking dynamics. Optogenetic inhibition effectively reduces spiking and bursting activity of the targeted neurons.

We compare the stimulation modes in terms of the minimal effective current delivered to basal ganglia neurons in order to suppress beta activity below a threshold: the less stimulation current is needed to suppress the activity, the more efficacious stimulation is. We found that optogenetic inhibition usually requires less effective current than electrical DBS to achieve beta suppression. Optogenetic excitation, while as not efficacious as optogenetic inhibition, still usually requires less effective current than electrical DBS to suppress beta activity.

Thus our results suggest that optogenetic stimulation may introduce less of effective currents to a neuron than conventional electrical DBS, but still achieve sufficient beta activity suppression. Optogenetics is presently not used in humans. However, it was implemented in the basal ganglia of non-human primates [3]. So we suppose our results may motivate further research into applicability of optogenetic technologies in humans. Optogenetic stimulation is also used as a research tool. Our results suggest that it may be more effective than electrical stimulation in control of synchronized oscillatory activity, because it does its job with less current injected into the neurons.

**Acknowledgements:** The study was supported by ICTSI and the Indiana University Health – Indiana University School of Medicine Strategic Research Initiative.

**References**

1. Park C, Worth RM, Rubchinsky LL. Neural activity in Parkinsonian brain: the boundary between synchronized and nonsynchronized dynamics. Phys Rev E. 2011;83:042901.
2. Park C, Worth RM, Rubchinsky LL. Fine temporal structure of beta oscillations synchronization in subthalamic nucleus in Parkinson’s disease. J Neurophysiol. 2010;103:2707–16.
3. Galvan A, Hu X, Smith Y, Wichmann T. In vivo optogenetic control of striatal and thalamic neurons in non-human primates. PLoS One. 2012;7(11):e50808.

## P73 Exact spike-timing distribution reveals higher-order interactions of neurons

### Safura Rashid Shomali^1^, Majid Nili Ahmadabadi^1,2^, Hideaki Shimazaki^3^, S Nader Rasuli^4,5^

#### ^1^School of Cognitive Sciences, Institute for Research in Fundamental Sciences (IPM), Tehran, 19395-5746, Iran; ^2^School of ECE, College of Engineering, University of Tehran, Tehran, 14155-6619, Iran; ^3^RIKEN Brain Science Institute, Wako, Saitama, 351-0198, Japan; ^4^Department of Physics, University of Guilan, Rasht, 41335-1914, Iran; ^5^School of Physics, Institute for Research in Fundamental Sciences (IPM), Tehran, 19395-5531, Iran

##### **Correspondence:** Safura Rashid Shomali - safura@ipm.ir

*BMC Neuroscience* 2016, **17(Suppl 1)**:P73

It has been suggested that variability in spike patterns of individual neuron is largely due to noisy fluctuations caused by asynchronous synaptic inputs balanced near the threshold regime [1–3]. In this regime, small fluctuations in synaptic inputs to a neuron do cause output spikes; because the membrane potential is maintained below but close enough to the threshold potential. To successfully transfer signals under such noisy conditions, it is proposed that a few relatively stronger synapses and/or an assembly of nearly synchronous ones form “signaling inputs” [4]. Thus one fundamental question is how such relatively strong signaling input modifies the spiking activity of a post-synaptic neuron which receives noisy background inputs balanced near the threshold regime. Nonetheless, analytical studies on the effect of the signaling input under such conditions are scarce even with the popular leaky integrate-and-fire (LIF) neuron model. Here we analytically study the impact of a specified signaling input on spike timing of the postsynaptic LIF neuron which receives noisy inputs at the threshold regime. To this end, we first revisit Fokker–Planck analysis of a first spike-timing distribution when the LIF neuron receives noisy synaptic inputs, but no signaling input, at the threshold regime. We then perform perturbation analysis to investigate how a signaling input modifies this first spike-timing distribution. Fortunately, we could solve all terms of perturbation analytically and find the exact first spike-timing distribution of the postsynaptic neuron; it is applicable to not only excitatory but also inhibitory input. This analytical solution allows us to describe the statistics of output spiking activity as a function of background noise, membrane dynamics, and signaling input’s timing and amplitude.

The proposed analysis of signaling input provides a powerful framework for studying information transmission, neural correlation, and timing-dependent synaptic plasticity. Among them, we investigate the impact of common signaling inputs on population activities of postsynaptic neurons. Using mixture models based on our analytical first spike-timing distribution, we calculate the higher-order interactions [5] of postsynaptic neurons in different network architectures. Comparing these results with higher-order interactions, measured from experimental data in monkey V1 [6], we try to answer whether one can reveal network architecture, responsible for the ubiquitously observed sparse activities.

**References**

1. Vreeswijk C, Sompolinsky H. Chaos in neuronal networks with balanced excitatory and inhibitory activity. Science. 1996;274: 1724–6.
2. Renart A, De La Rocha J, Bartho P, Hollender L, Parga N, Reyes A, Harris KD. The asynchronous state in cortical circuits. Science. 2010;327:587–90.
3. Tan AY, Chen Y, Scholl B, Seidemann E, Priebe NJ. Sensory stimulation shifts visual cortex from synchronous to asynchronous states. Nature. 2014;509:226–9.
4. Teramae Jn, Tsubo Y, Fukai T. Optimal spike-based communication in excitable networks with strong-sparse and weak-dense links. Sci Rep. 2:485.
5. Nakahara H, Amari S. Information-geometric measure for neural spikes. Neural Comput. 2002;14:2269–316.
6. Ohiorhenuan IE, Victor JD. Information-geometric measure of 3-neuron firing patterns characterizes scale dependence in cortical networks. J Comput Neurosci. 2011;30:125–41.

## P74 Neural mechanism of visual perceptual learning using a multi-layered neural network

### Xiaochen Zhao^1^, Malte J. Rasch^1^

#### ^1^State Key Lab of Cognitive Neuroscience and Learning, IDG/McGovern Institute for Brain Research, Beijing Normal University, Beijing 100875, China

**Correspondence:** Malte J. Rasch - malte.rasch@bnu.edu.cn

*BMC Neuroscience* 2016, **17(Suppl 1)**:P74

Recently, a study [1] has found by recording the activities of neurons in monkeys performing a contour detection task that the response properties of the primary visual cortex (V1) change continuously during perceptual learning. In particular, the figure-background contrast was continuously enhanced in the course of learning. However, the exact neural circuit mechanisms that causes the V1 responses to change during perceptual learning remain unclear. In order to understand how the underlying neural network needs to change, we here train a multi-layered neural network model to perform the contour detection task on the very same visual stimuli as in the experiments and investigate the network’s performance and the resulting synaptic weight structure.

**Fig. 46:**
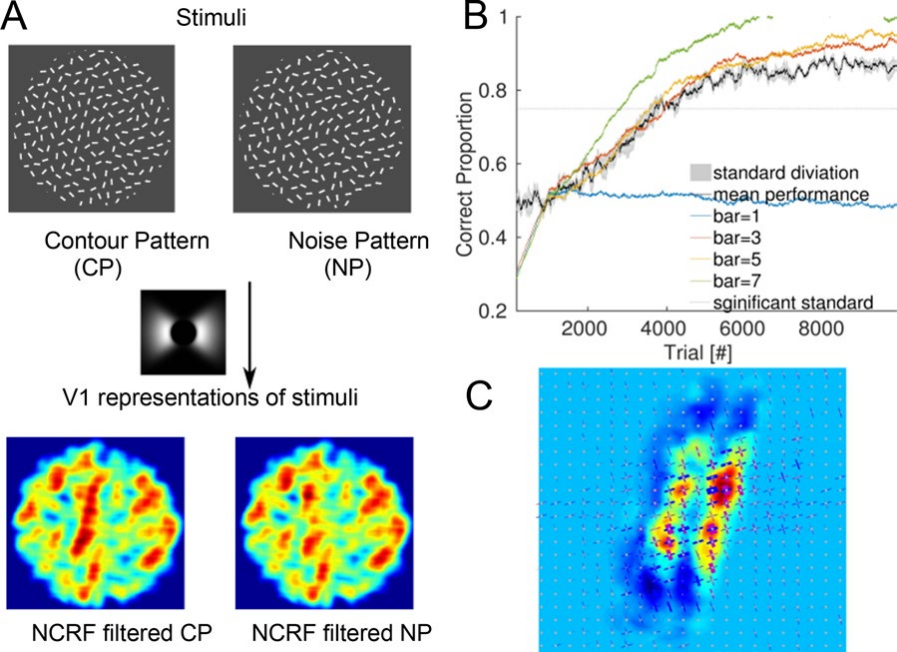
V1 representations of the stimuli and simulation results of the model. **A** V1 representations of the stimuli by using NCRF model. **B** Model performance increases with perceptual training. **C** Synaptic weight changes after perceptual learning

In this study, we first model the V1 representation of each visual stimulus by using a non-classical receptive field model (NCRF) which takes into account orientation selective inhibition [3]. We further assume that the higher visual areas (up to a decision unit) are hierarchically structured, read out the V1 activity, and learn to change their synaptic weights to optimally perform the contour detection task. AGREL (attention-gated reinforcement learning) algorithm [2], which considers feedback connections and biologically plausible local synaptic adjustments, is applied to train the network (see Fig. 46). We found that the multi-layered model trained with AGREL could replicate the behavioral performance increase in a contour detection task as observed in experiments. Moreover, learning the network model structure showed enhanced synaptic weights in the region of the detected contour. It further demonstrated that “predictive” feedback signals from higher layers facilitate the responses of V1 neurons to the contour and thus increased the figure-background contrast in V1 with improved behavioral performance. The results suggest that the experimental observed V1 response facilitation could be caused by selective synaptic strengthening of feed-forward and feed-back pathways.

**References**

1. Yan Y, Rasch MJ, Chen M, et al. Perceptual training continuously refines neuronal population codes in primary visual cortex. Nat Neurosci. 2014;17(10):1380–7.
2. Roelfsema PR, van Ooyen A. Attention-gated reinforcement learning of internal representations for classification. Neural Comput. 2005;17(10):2176–214.
3. Zeng C, Li Y, Yang K, et al. Contour detection based on a non-classical receptive field model with butterfly-shaped inhibition subregions. Neurocomputing. 2011;74(10):1527–34.

## P75 Inferring collective spiking dynamics from mostly unobserved systems

### Jens Wilting^1^, Viola Priesemann^1,2^

#### ^1^Max-Planck-Institute for Dynamics and Self-Organization, D-37077 Göttingen, Germany; ^2^Bernstein Center for Computational Neuroscience, University of Göttingen, D-37075 Göttingen, Germany

##### **Correspondence:** Jens Wilting - jwilting@nld.ds.mpg.de

*BMC Neuroscience* 2016, **17(Suppl 1)**:P75

What can we know about a high-dimensional dynamical system if we can only observe a very small part of it? This problem of spatial subsampling is common to almost every area of research where spatially extended, time evolving systems are investigated, and is particularly severe when assessing population spiking dynamics in neuroscience. Previous studies have shown that subsampling can lead to spurious results when assessing the dynamical state of spiking activity, in particular when discriminating whether neural networks operates at criticality [1, 2]. Here we present further insight why the distance to criticality is systematically overestimated, and introduce a novel estimator which for the first time allows to correctly infer the distance to criticality even under strong subsampling.

Neuronal systems have been proposed to operate close to criticality, because in models criticality maximizes information processing capacities [e.g. 3]. Indeed, power-law distributions of the avalanche size, an indication of criticality, have been found for local field potentials from in vitro systems [1] to humans in vivo [4]. However, for neuronal systems criticality also comes with the risk of spontaneous runaway activity, which may lead to pathological states like epilepsy. Experiments indeed indicate that spiking activity in rats, cats, and monkeys is in a sub-critical regime, thereby keeping a safety-margin from criticality [5]. Quantifying the precise distance to criticality may help to shed light on how the brain maximizes its information processing capacities without risking runaway activity.

In neural systems, critical dynamics is typically compared to dynamics from models that resemble branching processes [1]. Their dynamics are controlled by a single parameter, the expected number σ of postsynaptic spikes generated by one individual spike, showing either stationary dynamics (sub-critical, σ < 1) or transient growth (super-critical, σ > 1). For σ = 1 branching processes are critical and produce heavy tailed avalanche size distributions. We used a driven branching process, which allows to exactly match the model neuron firing rate to that observed in experiments for any σ. We propose a stochastic representation of subsampling and show that under subsampling established approaches to inferring σ are substantially biased. We derived a novel approach based on multistep regression [6], which for the first time allows to quantify the distance to criticality even under strong subsampling. Our method generalizes to auto-regressive processes with both additive and multiplicative noise, making it widely applicable in diverse fields of research. We validate our method by applying subsampling to simulated branching networks with invasion, and also to a network of integrate-and-fire neurons.

We applied this method to spike recordings from awake macaque monkeys prefrontal cortex, cat visual cortex, and rat hippocampus. We found that neuronal population activity operates close to criticality, but in a subcritical regime with 0.94 < σ < 0.995. These results point at a novel universal organization principle: spiking dynamics in vivo is in a subcritical regime which does not yield maximum, but sufficient information processing capacity, and at the same time keeps a safety-margin from unstable supercritical states.

**References**

1. Beggs J, Plenz D. Neuronal avalanches in neocortical circuits. J Neurosci. 2003;23(35):11167–77.
2. Priesemann V, Munk MHJ, Wibral M. Subsampling effects in neuronal avalanche distributions recorded in vivo. BMC Neurosci. 2009;10:40.
3. Boedecker J, Obst O, Lizier JT, Mayer NM, Asada M. Information processing in echo state networks at the edge of chaos. Theory Biosci. 2012;131:205–13.
4. Priesemann V, Valerrame M, Wibral M, Le Van Quyen M. Neuronal avalanches differ from wakefulness to deep sleep—evidence from intracranial depth recordings in humans. PloS Comput Biol. 2013;9(3):e1002985.
5. Arviv O, Goldstein A, Shriki O. Near-critical dynamics in stimulus-evoked activity of the human brain and its relation to spontaneous resting-state activity. J Neurosci. 2015;35(41):13927–42.
6. Priesemann V, et al. Spike avalanches in vivo suggest a driven, slightly subcritical brain state. Front Syst Neurosci. 2014;8:108.
7. Wilting J, Priesemann V. Quantifying the distance to criticality under subsampling. BMC Neurosci. 2015;16:O3.

## P76 How to infer distributions in the brain from subsampled observations

### Anna Levina^1^, Viola Priesemann^2^

#### ^1^IST Austria, Klosterneuburg, 3400, Austria; ^2^BCCN & MPI for Dynamics and Self-Organization, Göttingen, 37077, Germany

##### **Correspondence:** Anna Levina - anna.levina@ist.ac.at

*BMC Neuroscience* 2016, **17(Suppl 1)**:P76

Inferring the dynamics of a system from observations is a challenge, even if one can observe all system units or components. The same task becomes even more challenging if one can sample only a small fraction of the units at a time. As a prominent example, spiking activity in the brain can be accessed only for a very small fraction of all neurons in parallel. These limitations do not affect our ability to infer single neuron properties, but it influences our understanding of the global network dynamics or connectivity: Subsampling can hamper inferring whether a system shows scale-free topology or scale-free dynamics (criticality) [1, 2]. Criticality is a dynamical state that maximizes information processing capacity in models, and therefore is a favorable candidate state for brain function. Experimental approaches to test for criticality extract spatio-temporal clusters of spiking activity, called avalanches, and test whether they followed power laws. Avalanches can propagate over the entire system, thus observations are strongly affected by subsampling. We developed a formal ansatz to infer avalanche distributions in the full system from spatial subsampling using both analytical and numerical approaches.

**Fig. 47:**
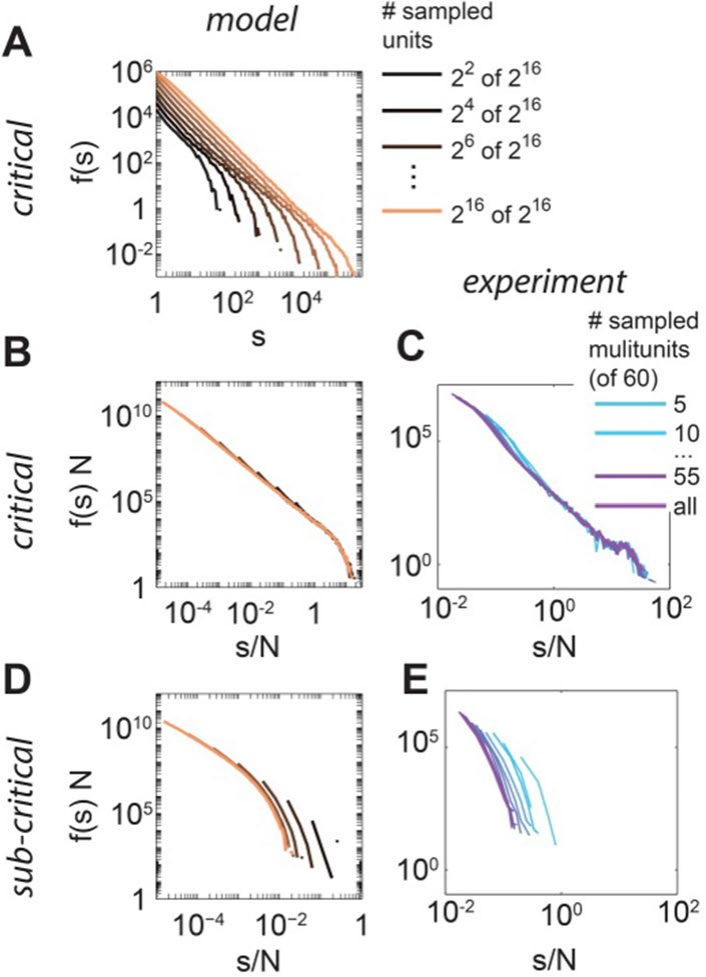
Subsampling scaling in model and experiment. *Left* branching process model; right: experiments on developing cultures **A** Avalanche size counts *f*(*s*) from the full and the subsampled *critical* model; N: number of sampled neurons. **B** Under subsampling scaling, all *f*(*s*) collapse. **C** Collapse of subsampled avalanche-size distribution from the culture at the age of 21 days. **D** For *subcritical* models, the same scaling ansatz does not result in a collapse. **E** No collapse of *f*(*s*) from the culture at age 7 days

In the mathematical model subsampling from exponential distribution does not change the class of distribution, but only its parameters. In contrast, power law distributions, despite their alias “scale-free”, do not manifest as power laws under subsampling [2]. We study changes in distributions to derive “subsampling scaling” that allows to extrapolate the results from subsampling to a full system: P(s) = p_sub_P_sub_(s/p_sub_) where P(s) is the original distribution, P_sub_ is the one under subsampling, and ${\text{p}}_{\text{sub}} = \frac{\text{N}}{\text{M}}$ is the probability to sample a unit, *N*—number of sampled units, *M*—system size. In the model with critical avalanches, subsampling scaling collapses distributions for any *N* (Fig. 47B). However, for subcritical models, no distribution collapse is observed (Fig. 47D). Thus we demonstrate that subsampling scaling allows to distinguish critical from non-critical systems. With the help of this novel method we studied dissociated cortical cultures. For these we artificially subsampled recordings by considering only fraction of all 60 electrodes. We find that in the first days subsampling scaling does not collapse distributions well, whereas mature cultures (~from day 21) allow for a good collapse, indicating development toward criticality (Fig. 47C, E).

**Acknowledgements:** AL received funding from the Marie Curie Actions (FP7/2007–2013) under REA Grant Agreement No. (291734). VP received funding from BMBF Bernstein 01GQ1005B.

**References**

1. Stumpf, MPH, Wiuf C, May RM. Subnets of scale-free networks are not scale-free: sampling properties of networks. PNAS 2005;102(12):4221–24.
2. Priesemann V, Munk MH, Wibral M. Subsampling effects in neuronal avalanche distributions recorded in vivo. BMC Neurosci. 2009;10(1):40.
3. Uhlig M, Levina A, Geisel T, Herrmann JM. Critical dynamics in associative memory networks. Front Comp Neurosci. 2013;7.

## P77 Influences of embedding and estimation strategies on the inferred memory of single spiking neurons

### Lucas Rudelt^1^, Joseph T. Lizier^2^, Viola Priesemann^1^

#### ^1^Department of Non-linear Dynamics, Max Planck Institute for Dynamics and Self-Organization, Göttingen, Germany; ^2^School of Civil Engineering, The University of Sydney, Sydney, NSW, Australia

##### **Correspondence:** Lucas Rudelt - l.rudelt@gmail.com

*BMC Neuroscience* 2016, **17(Suppl 1)**:P77

**Fig. 48:**
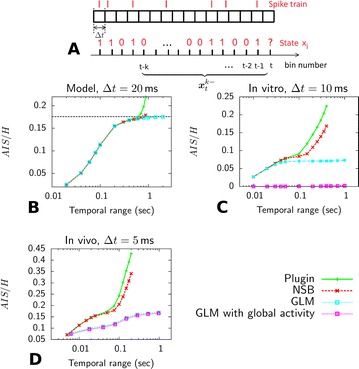
Relative active information storage as a function of the time range of the past state for different estimators

Information theory provides a generic framework for studying statistical dependencies, and is widely used in neuroscience. However, a correct estimation of the involved quantities can be challenging. For example, a correct estimation of transfer entropy (TE), TE(X → Y) = I(**X**^−^, Y|**Y**^−^), or active information storage (AIS), AIS(X) = I(**X**^−^, X), requires past state variables **X**^−^ and **Y**^−^ that encode all information about the past that is relevant when predicting X or Y [1]. For a spiking neuron, states can be defined by transforming the spike train into a binary sequence of spike counts in sufficiently small, equally spaced bins with some bin size Δ*t* (Fig. 48A). For neurons, however, it is unclear how many past bins a sufficient state variable typically comprises. In practice, past states have often been limited to only one time bin to reduce the complexity of estimation. This points at the main challenge one faces when estimating TE and AIS: A reliable estimation of probabilities from recorded data becomes more and more difficult with increasing complexity of the state variables, i.e. with considering more past bins. We used AIS for single spiking neurons to estimate (a) how much memory there is, (b) how long it reaches typically into the past, (c) the (non-)linear contributions of the memory. To this end, we first examined the performance of different estimators for a realistic model neuron [2] whose AIS can be directly computed (dashed line, Fig. 48B). Using constant external drive we simulated a recording of 12 h. In a model-free approach, probabilities were directly estimated from relative frequencies using the standard ‘plugin’ estimator or the ‘NSB’ estimator [3]. In addition, we fitted a generalized linear model (GLM) whose predictions constitute an estimator that is constrained to linear contributions. For all these estimation strategies, the number of past bins k and thus the time range was systematically varied (Fig. 48B). We then applied the same estimators to in vitro and in vivo recordings (Fig. 48c, d) of 3 h and 1 h duration.

Considering a very small number k of past bins can lead to a substantial underestimation of AIS. Increasing the number of past bins, however, leads to severe positive bias of the model-free estimators when the complexity of the past state becomes too large. This manifests in the estimators exceeding the true AIS (Fig. 48B). Assuming a point process with linear contributions (GLM), in contrast, allows a robust estimation but does not capture non-linear effects. This approach can also be used to take the global past activity of the neural population into account, thereby unveiling redundancies in the activity of the single neuron with the population activity. While the model neuron intrinsically has only linear dependencies on its past, it is surprising that, in vitro, there also seem to be very little non-linear contributions and a lot of redundancy. In vivo, the non-linear contributions are more prominent and the memory is clearly non-redundant.

We thus showed that appropriate embedding is necessary, otherwise AIS is underestimated and likewise, TE might be mis-estimated. Furthermore, our results suggest that in vivo the information processing is more evolved.

**References**

1. Wibral M, Lizier J, Priesemann V. Bits from brains for biologically-inspired computing. Comput Intell. 2015;2:5.
2. Pozzorini, C, Naud R, Mensi S, Gerstner W. Temporal whitening by power-law adaptation in neocortical neurons. Nat Neurosci. 2013;16(7):9428.
3. Nemenman I, Shafee F, Bialek W. Entropy and inference, revisited. Adv Neural Inf Process Syst. 2002;14:471–478.

## P78 A nearest-neighbours based estimator for transfer entropy between spike trains

### Joseph T. Lizier^1^, Richard E. Spinney^1^, Mikail Rubinov^2,3^, Michael Wibral^4^, Viola Priesemann^5,6^

#### ^1^Complex Systems Research Group, Faculty of Engineering & IT, The University of Sydney, NSW 2006, Australia; ^2^Janelia Research Campus, Howard Hughes Medical Institute, Ashburn, VA 20147, USA; ^3^Department of Psychiatry, University of Cambridge, Cambridge, UK; ^4^MEG Unit, Brain Imaging Center, Goethe University, 60528 Frankfurt am Main, Germany; ^5^Department of Nonlinear Dynamics, Max Planck Institute for Dynamics & Self-Organization, Göttingen, Germany; ^6^Bernstein Center for Computational Neuroscience, Göttingen, Germany

##### **Correspondence:** Joseph T. Lizier - joseph.lizier@sydney.edu.au

*BMC Neuroscience* 2016, **17(Suppl 1)**:P78

The nature of a directed relationship (or lack thereof) between brain areas is a fundamental topic of inquiry in computational neuroscience [1]. A particular focus of such inquiry is in regards to the analysis of information flows in a network [2], and such investigations take place at all levels of analysis, from interregional connectivity in fMRI imaging data [3] down to directed relationships between spike trains at the neuronal level [4].

In all of the aforementioned studies, information theory provides the primary tool, transfer entropy (TE) [5], for analysis of such directed relationships. TE measures the predictive gain about state transitions in a target time-series from observing some source time-series. While the TE has been used extensively to analyse recordings from fMRI, MEG and EEG for example [1–3], fewer applications [4] have been made to spiking time-series. Although one can apply temporal binning on such time-series before measuring TE on the resultant binary time-series [4], it remains unclear: (a) how to set parameters for this approach (e.g. bin sizes), (b) whether an estimate can be achieved by avoiding temporal binning and instead working directly with continuous-valued time stamps of spikes, and (c) whether such an estimate would actually improve on binning approaches.

Recent theoretical developments have pointed to how transfer entropy may be derived from continuous-valued time-stamps of spikes directly, using spike rates conditioned on previous spike histories [6]. Yet, it is not immediately obvious how an estimator for this form would be constructed, and indeed construction of such an estimator has previously defaulted to a binning or discretisation of time [7]. Here, we propose an estimator for this continuous-time point-process formulation of TE that remains in the continuous-time regime by harnessing a nearest-neighbours approach [8] to matching (rather than binning) inter-spike interval (ISI) histories and future spike-times. By retaining as much information about ISIs as possible, this estimator is expected to improve on properties of TE such as robustness to noise and undersampling, bias removal, and sensitivity, etc. We are currently implementing the proposed estimation algorithm in open-source code (i.e. contributing to JIDT [9] and TRENTOOL [10]), and evaluating the properties of the algorithm particularly in comparison to temporal binning approaches.

**References**

1. Wibral M, Vicente R, Lizier JT, editors. Directed information measures in neuroscience. Berlin: Springer-Verlag; 2014.
2. Vicente R, Wibral M, Lindner M, Pipa G. Transfer entropy—a model-free measure of effective connectivity for the neurosciences. J Comp Neurosci. 2011;30(1):45–67.
3. Lizier JT, Heinzle J, Horstmann A, Haynes J-D, Prokopenko M. Multivariate information-theoretic measures reveal directed information structure and task relevant changes in fMRI connectivity. J Comp Neurosci. 2011;30(1):85–107.
4. Ito S, Hansen ME, Heiland R, Lumsdaine A, Litke AM, Beggs JM. Extending transfer entropy improves identification of effective connectivity in a spiking cortical network model. PLoS One. 2011;6(11):e27431.
5. Schreiber T. Measuring information transfer. Phys Rev Lett. 2000;85:461–4.
6. Bossomaier T, Barnett L, Harré M, Lizier JT. An introduction to transfer entropy: information flow in complex systems. Berlin: Springer; 2016 (in press).
7. Kim S, Putrino D, Ghosh S, Brown EN. A Granger causality measure for point process models of ensemble neural spiking activity. PLoS Comput Biol. 2011;7(3):e1001110.
8. Kraskov A, Stögbauer H, Grassberger P. Estimating mutual information. Phys Rev E. 2004;69(6):066138.
9. Lizier JT. JIDT: an information-theoretic toolkit for studying the dynamics of complex systems. Front Robot AI. 2014;1:11.
10. Lindner M, Vicente R, Priesemann V, Wibral M. TRENTOOL: a Matlab open source toolbox to analyse information flow in time series data with transfer entropy. BMC Neurosci. 2011;12(1):119.

## P79 Active learning of psychometric functions with multinomial logistic models

### Ji Hyun Bak^1^, Jonathan Pillow^2^

#### ^1^Department of Physics & Lewis-Sigler Institute for Integrative Genomics, Princeton University, Princeton, NJ 08544, USA; ^2^Department of Psychology & Princeton Neuroscience Institute, Princeton University, Princeton, NJ 08544, USA

##### **Correspondence:** Ji Hyun Bak - jhbak@princeton.edu

*BMC Neuroscience* 2016, **17(Suppl 1)**:P79

**Fig. 49:**
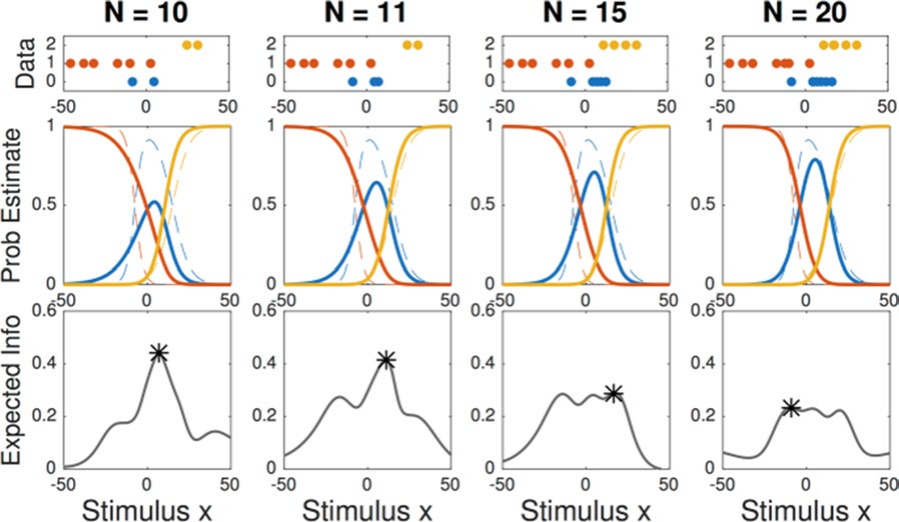
Example of active learning, simulated with a three-alternatives model on 1D stimulus. After each observation, the psychometric functions are estimated based on the accumulated data, and the next stimulus is chosen to maximize the expected information gain. The estimated psychometric functions (*solid lines*) quickly approach the true functions (*dashed lines*) through the adaptive and optimal choice of stimuli

As new technologies expand the capacity for making large-scale measurements of neural activity, there is a growing need for methods to rapidly characterize behavior and its dependence on stimuli. In typical experiments, an animal is presented with a stimulus on each trial and has to select a response among several options. Since such experiments are costly, a problem of practical importance is to learn the animal’s psychometric choice functions from a minimal amount of data. Here we show that one can achieve substantial speedups over traditional randomized designs via active learning, in which stimuli are selected adaptively on each trial according to an information-theoretic criterion, as shown in Fig. 49. Specifically, we model behavior with a multinomial logistic regression model, in which the probability of each choice given a stimulus depends on a set of linear weights. Our work extends previous work on this problem [1–3] in several important ways. First, we incorporate an explicit lapse rate to account for the fact that observers may occasionally make errors on “easy” trials due to lapses in concentration or memory [4]. Second, we develop an efficient method based on Markov Chain Monte Carlo (MCMC) sampling that is accurate in settings in which the log-likelihood is not concave, for example as in the presence of lapse rates. Third, we extend consideration for multiple-alternative responses, extending previous work for binary responses. We compare the performance of our sampling-based method to one based on a local (Laplace) approximation to the posterior [5], and show that failure to incorporate lapse rates can have deleterious effects on the accuracy of inferred parameters under both methods. We test our method on simulated data, as well as on an experimental dataset concerning the multiple-alternative choice behavior of monkeys [6], demonstrating that active sampling of the stimulus space facilitates the learning of the psychometric function significantly, as well as suggesting that the full range of the multi-dimensional stimulus could have been exploited more efficiently using our active learning framework. Finally, we discuss the comparative advantages and disadvantages of the different methods, and how one might adapt these algorithms to achieve best results.

**Acknowledgements:** We thank Anne Churchland for providing the data. JP was supported by the McKnight Foundation, Simons Global Brain Initiative, NSF CAREER Award IIS-1150186, and NIMH grant MH099611. JHB was supported by NSF grant PHY-1521553.

**References**

1. Kontsevich LL, Tyler CW. Bayesian adaptive estimation of psychometric slope and threshold. Vis Res. 1999;39:2729–37.
2. Zocchi SS, Atkinson AC. Optimum experimental designs for multinomial logistic models. Biometrics. 1999;55:437–44.
3. DiMattina C. Fast adaptive estimation of multidimensional psychometric functions. J Vis. 2015;15:1–20.
4. Kuss M, Jakel F, Wichmann FA. Bayesian inference for psychometric functions. J Vis. 2005;5:478–92.
5. Lewi J, Butera R, Paninski L. Sequential optimal design of neurophysiology experiments. Neural Comput. 2009;21:619–87.
6. Churchland AK, Kiani R, Shadlen MN. Decision-making with multiple alternatives. Nat Neurosci. 2008;11:693–702.

## P81 Inferring low-dimensional network dynamics with variational latent Gaussian process

### Yuan Zaho^1,2^, Il Memming Park^1,3^

#### ^1^Department of Neurobiology and Behavior, Stony Brook University, Stony Brook, NY 11794, USA; ^2^Department of Applied Mathematics and Statistics, Stony Brook University, Stony Brook, NY 11794, USA; ^3^Institute for Advanced Computational Science, Stony Brook University, Stony Brook, NY 11794, USA

##### **Correspondence:** Il Memming Park - memming.park@stonybrook.edu

*BMC Neuroscience* 2016, **17(Suppl 1)**:P81

Surprisingly, large-scale population recordings often show signatures of low-dimensional dynamics, that is, variations in a small number of common factors explain most of the dependence among neurons [1–3]. This supports the idea that a large neuronal network is implementing necessary computations described by continuous low-dimensional nonlinear dynamics. Sufficient amount of redundancy in the population activity would allow us access to the internal computation process of interest even when we only observe a small subset of neurons. Thus, it is necessary to deduce the latent dynamics from neural time series in order to understand if and how neural systems operate in this regime. There are several latent variable models that aim at recovering the latent dynamics, howerver, they make inadequate assumptions in favor of fast inference [4]. Here we describe an approximate inference method that recovers the latent dynamics under a natural generative model with minimal assumptions.

We implemented a probabilistic method to extract shared low-dimensional latent dynamics from multi-channel neural recordings (LFP and spike trains) to reveal how neural population encodes information, and how multiple functional neural populations dynamically interact with each other. Key assumptions of our model are: (1) each neural signal represent a noisy mixture of common latent dynamics, and (2) latent dynamics are independent and temporally smooth (with possibly different time scales). We use autoregressive generalized linear model driven by latent dynamics. Unlike most of the literature [5], we do not impose linear dynamics as a prior on the latent process, instead we use a general gaussian process prior which provides a flexible framework for imposing structure such as smoothness. However, as a result, the exact posterior inference is intractable, thus we developed a variational method to find a Gaussian approximation to the posterior [6]. Our inference algorithm is memory-efficient and fast: both linear in time using a low-rank approximation of the covariance. We compare our method on both simulated systems and real data from V1 driven by drifting gratings. For a population of 148 V1 neurons, 11.4 % of the variance was explained by a shared 4-dimensional latent process, while 10 % of the variance was explained by independent variability of each neuron. We recovered orientation dependent embedding that faithfully encode the stimulus drive on average, and the population-wide trial-to-trial modulation. In conclusion, we present an efficient and scalable method to recover underlying dynamics from noisy partial observations to study neural code and computation.

**References**

1. Okun M, Steinmetz NA, Cossell L, Iacaruso MF, Ko H, Barthó P, Moore T, Hofer SB, Mrsic-Flogel TD, Carandini M, Harris KD. Diverse coupling of neurons to populations in sensory cortex. Nature. 2015;521(7553):511–15.
2. Goris RLT, Movshon JA, Simoncelli EP. Partitioning neuronal variability. Nat Neurosci. 2014;17(6):858–65.
3. Luczak A, Bartho P, Harris KD. Gating of sensory input by spontaneous cortical activity. J Neurosci. 2013;33(4):1684–95.
4. Yu BM, Cunningham JP, Santhanam G, Ryu SI, Shenoy KV, Sahani M. Gaussian-process factor analysis for low-dimensional single-trial analysis of neural population activity. J Neurophysiol. 2009;102(1):614–35.
5. Paninski L, Ahmadian Y, Ferreira DGG, Koyama,S, Rahnama Rad K, Vidne M, Vogelstein J, Wu W. A new look at state-space models for neural data. Journal of Comput Neurosci. 2010;29(1–2):107–26.
6. Blei DM, Kucukelbir A, McAuliffe JD. Variational inference: a review for statisticians. arXiv 2016 1601.00670 [http://arxiv.org/abs/1601.00670 arXiv]

## P82 Computational investigation of energy landscapes in the resting state subcortical brain network

### Jiyoung Kang^1^, Hae-Jeong Park^2^

#### ^1^Graduate School of Life Science, University of Hyogo, 3-2-1 Koto, Kamigori, Ako, Hyogo, 678-1297, Japan; ^2^Department of Nuclear Medicine, Radiology and Psychiatry, Yonsei University College of Medicine, Department of Cognitive Science, Yonsei University, 50 Yonsei-ro, Sinchon-dong Seodaemoon-gu, Seoul, 120-752, Republic of Korea

##### **Correspondence:** Hae-Jeong Park - parkhj@yuhs.ac

*BMC Neuroscience* 2016, **17(Suppl 1)**:P82

Recently, energy landscapes of the resting state functional brain network have been researched using the pairwise maximum entropy model (MEM). This approach considers not only activities of nodes but also interactions among nodes in modeling brain networks and thus estimating energy landscapes of the brain state [1, 2]. From the energy landscape models, we can identify major stable states (local minima) and estimate transition rates among stable states.

The brain networks of the resting states are known to be affected by brain diseases or treatments. In the pairwise MEM, such effects correspond to changes in the parameters of the baseline activities and pairwise interactions.

In the present study, we investigated the energy landscape and its robustness of the subcortical human brain network that plays a central role in the human brain. The subcortical brain regions we examined were 15 regions of interests (ROIs); hippocampus, amygdala, caudate, putamen, pallidum, thalamus, nucleus accumbens, and brainstem. To construct a pairwise MEM for spontaneous interactions among subcortical brain regions, we used resting state fMRI (rs-fMRI) data of the human connectome projects, which contains 468 people’s data. The blood oxygen level-dependent signals in the ROI were first binarized to represent states (zero for inactive, one for active states) of the ROI, and thereby 2^15^ brain states were considered. The parameters of the MEM were fitted to reproduce observed activation patterns of the rs-fMRI data. The constructed MEM showed high accuracy of fit (~92.6 %) and reliability (~99.9 %).

We found symmetric properties for the left and right hemispheres, and confirmed estimated parameters grossly reflecting the known anatomical connectivity of the subcortical brain. We further investigated the robustness of the system by perturbing the global weight for interactions, parameters for baseline activities of ROI and parameters for interactions between pairs of all ROIs (1906 edges), one by one from the original MEM. Alteration of the energy landscapes after perturbation was measured with respect to the number of local minima. We found that the number of the local minima of the subcortical system without any perturbation is very high. This implies that the subcortical brain system is optimal in the sense of its largest coverage of local minima (maximal number of local minima). This result suggests that brain was built to have multiple stable states. We also found different categories of parameters that affect the energy landscape of the resting state. For example, small increase in the pairwise parameter between the caudate and putamen dramatically reduced the numbers of the local minima while reduction in this parameter did not change the energy landscape.

In conclusion, MEM analysis of resting state functional network would be an important tool to understand principles of the brain organization and could be useful in researching brain disease.

**References**

1. Watanabe T, Hirose S, Wada H, Imai Y, Machida T, Shirouzu I, Konishi S, Miyashita Y, Masuda N. A pairwise maximum entropy model accurately describes resting-state human brain networks. Nat Commun. 2013;4:1370.
2. Watanabe T, Hirose S, Wada H, Imai Y, Machida T, Shirouzu I, Konishi S, Miyashita Y, Masuda N. Energy landscapes of resting-state brain networks. Front Neuroinform. 2014;8:12.

## P83 Local repulsive interaction between retinal ganglion cells can generate a consistent spatial periodicity of orientation map

### Jaeson Jang^1^, Se-Bum Paik^1,2^

#### ^1^Department of Bio and Brain engineering, Korea Advanced Institute of Science and Technology, Daejeon 34141, Republic of Korea; ^2^Program of Brain and Cognitive Engineering, Korea Advanced Institute of Science and Technology, Daejeon 34141, Republic of Korea

##### **Correspondence:** Jaeson Jang - jaesonjang@kaist.ac.kr

*BMC Neuroscience* 2016, **17(Suppl 1)**:P83

**Fig. 50:**
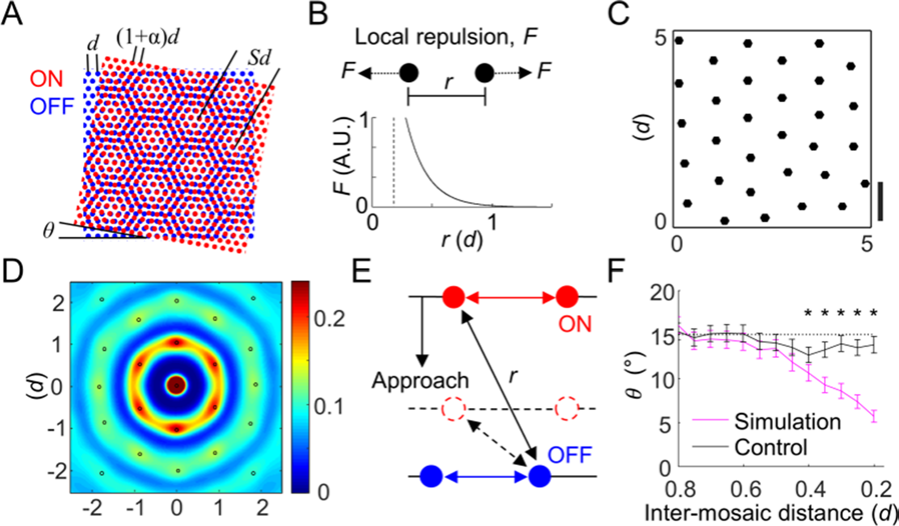
Local repulsive interaction develops a consistent interference between mosaics. **A** Moiré pattern of RGC. **B** Developmental model of RGC mosaic with local repulsive interaction between nearby cells. **C** Developed cell mosaic. **D** Autocorrelation of developed mosaics. **e** Approach between ON and OFF mosaics induces a gradual reinforcement of heterotypic interaction. **F** Angle alignment between mosaics (θ) is limited to low angles as mosaics approach (*: p < 0.05, Ranksum test, error bar: SE)

Orientation map in the primary visual cortex (V1) is of great interest among functional maps in the brain, but its developmental mechanism has been under debate. A recently suggested idea is that a moiré interference pattern between ON and OFF retinal ganglion cells (RGCs) can develop a quasi-periodic structure of orientation map [1] (Fig. 50A). In this model, the mosaics of ON and OFF RGCs that are in hexagonal lattice patterns generate a periodic interference pattern and induce a cortical orientation preference map. This model successfully explains the mechanism of map development, but two questions remain unanswered yet; (1) How does the hexagonal pattern of RGC mosaic develop and (2) how is the angle alignment (θ) between ON and OFF RGC mosaics (Fig. 50A) restricted to seed the consistent spatial periodicity of orientation map? Here, we suggest that a local repulsive interaction between the nearby cells is enough to develop hexagonal RGC mosaics and consistent alignment of ON and OFF mosaics.

To validate this idea of developmental process of cell mosaic, we assumed a local repulsive force between the nearby cells as a function of distance between two cells (Fig. 50B), which induces a gradual shift of cell position. In our model simulations, we confirmed that this model could develop a hexagonal pattern in the monotypic RGC mosaic (Fig. 50C, D). Next, we examined how the angle alignment between ON and OFF mosaics can be achieved by homotypic (ON–ON or OFF–OFF) and heterotypic (ON–OFF) interaction between RGCs. We simulated the development of ON and OFF mosaics as we allow a heterotypic interaction and gradually reduce the distance between two mosaics (Fig. 50E). When two mosaics get closer enough, we observed that the angle alignment between ON and OFF mosaics was limited to low angles (Fig. 50F). Finally, we analyzed previously reported cat RGC mosaic data [2, 3] and concluded that the data suggests the existence of heterotypic repulsive interaction between ON and OFF mosaics, as our model predicted.

Our results suggest that a local repulsive interaction between RGCs can develop a hexagonal pattern in mosaics and restrict the angle alignment between ON and OFF RGC mosaics to generate a constant spatial period of orientation map. This model may provide a complementary mechanism of the retinal origin of periodic functional maps in the brain.

**References**

1. Paik S-B, Ringach DL. Retinal origin of orientation maps in visual cortex. Nat Neurosci. 2011;14:919–925.
2. Zhan XJ, Troy JB. Modeling cat retinal beta-cell arrays. Vis Neurosci. 2000;17:23–39.
3. Wassle H, Boycott BB, Illing R-B. Morphology and mosaic of ON- and OFF-beta cells in the cat retina and some functional considerations. Proc R Soc B Biol Sci. 1981;212:177–95.

## P84 Phase duration of bistable perception reveals intrinsic time scale of perceptual decision under noisy condition

### Woochul Choi^1,2^, Se-Bum Paik^1,2^

#### ^1^Department of Bio and Brain Engineering, KAIST, Daejeon 34141, Republic of Korea; ^2^Program of Brain and Cognitive Engineering, KAIST, Daejeon 34141, Republic of Korea

##### **Correspondence:** Woochul Choi - choiwc1128@kaist.ac.kr

*BMC Neuroscience* 2016, **17(Suppl 1)**:P84

When we see an ambiguous visual stimulus such as Necker cube, our perceived state switches periodically between two possible interpretations. This phenomenon, called bistable perception, is considered important to the study of dynamic mechanism of sensory perception. In particular, the time duration of each perceptual state, termed “phase duration”, seems to be a crucial factor to understanding the temporal features of underlying neural activity during sensory perception under this condition, which has not been studied intensively yet.

**Fig. 51:**
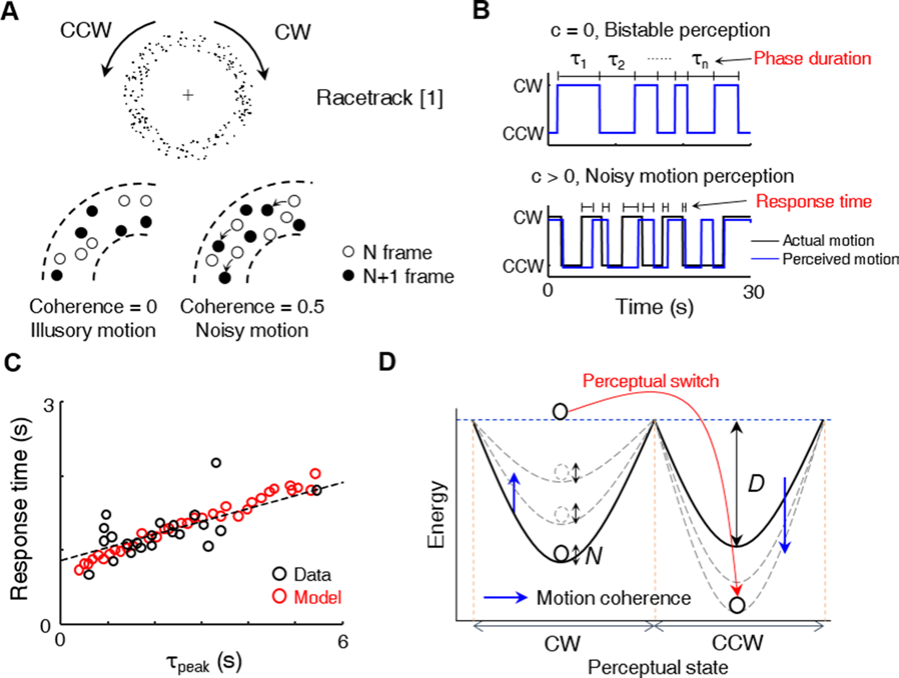
Correlation between bistable perception and perception under ambiguous signal. **A** Racetrack stimulus. Rotational motion can be either illusory or ambiguous depending on coherence. **B** Example response of racetrack. Perceived motion can be bistable (*top*) or follows actual motion with response time (bottom). **C** Subjects’ (*black*) and model’s (*red*) phase duration and response time are highly correlated. **D** Double-well energy model to describe behavior during bistable perception and perceptual decision making task

In this study, we assume that phase duration is intrinsically correlated with time delay in cognitive tasks, such as decision making from sensory information. Our hypothesis is that the periodic switching in bistable perception is a repeated process of decision making and reveals the time scale required for this decision task. To confirm our hypothesis, we performed a human psychophysics experiment using the “racetrack” type stimulus [1], which can induce a motion perception from both bistable illusion and real motion by varying the coherence parameter, c (Fig. 51A). We examined the relationship between the phase duration, τ, under illusory motion (c = 0) and the response delay for coherent motion with different degrees of ambiguity (c > 0, Fig. 51B). Our result showed that the response delay in the coherent motion detection task (c > 0) was highly correlated with the phase duration in the bistable illusory motion perception (c = 0) (Fig. 51C, N = 19, R = 0.61, p < 0.01, Pearson’s correlation coefficient). For a systematic analysis of subjects’ performance for these two tasks, we designed a theoretical model of simple double-well energy potential [2] (Fig. 51D). The model could successfully replicate the correlation between phase duration and response delay in each task, suggesting that bistable perception and perceptual decision making processes may share a common neural mechanism (Fig. 51C).

**Conclusions** Our findings show that the phase duration of bistable perception is highly correlated with the response time of a cognitive task. Our simple model suggests that the bistable perception can be interpreted as perceptual decision making process under highly ambiguous condition and share similar temporal dynamics.

**References**

1. Jain S. Performance characterization of Watson Ahumada motion detector using random dot rotary motion stimuli. PLoS One. 2009;4.
2. Kornmeier J, Bach M. Ambiguous figures—what happens in the brain when perception changes but not the stimulus. Front Hum Neurosci. 2012;6:51.

## P85 Feedforward convergence between retina and primary visual cortex can determine the structure of orientation map

### Changju Lee^1^, Jaeson Jang^1^, Se-Bum Paik^1,2^

#### ^1^Department of Bio and Brain Engineering, Korea Advanced Institute of Science and Technology, Daejeon 34141, Republic of Korea; ^2^Program of Brain and Cognitive Engineering, Korea Advanced Institute of Science and Technology, Daejeon 34141, Republic of Korea

##### **Correspondence:** Changju Lee - lcj110808@kaist.ac.kr

*BMC Neuroscience* 2016, **17(Suppl 1)**:P85

**Fig. 52:**
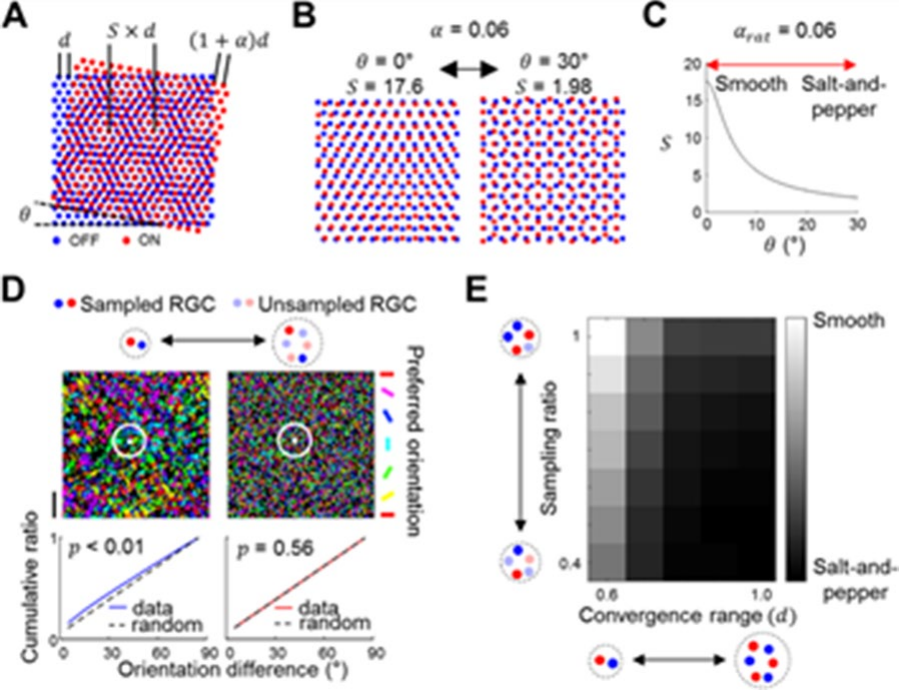
The simulation model for developmental mechanism of salt-and-pepper map by feedforward convergence between retina and V1. **A** Moiré interference between ON and OFF RGC mosaics. **B** Moiré interference with small and large alignment angles can generate various range of periodicity, *S*. **C** From rat RGC mosaics, both smooth and salt-and-pepper map can be developed. **D** Spatial distribution of preferred orientations of V1 cells by different convergence conditions; Smooth map model (high sampling ratio and large convergence range), Salt-and-pepper map model (low sampling ratio and short convergence range). **E** The structure of orientation map depending on convergence range and sampling ratio

Orientation map in the primary visual cortex (V1) is one of the most studied functional maps in the brain. In higher mammals such as monkeys and cats, preferred orientation of each V1 neuron appears continuous and periodic across cortical space. On the other hand, in rodents, it appears completely discontinuous, forming a structure called salt-and-pepper map. However, the developmental mechanism of salt-and-pepper orientation maps remains unclear. Previously, a model study suggested that a moiré interference pattern between ON and OFF retinal ganglion cell (RGC) mosaics can seed a periodic orientation maps (Fig. 52A), and a salt-and-pepper map can also be developed when the spatial periodicity of interference pattern is very short [1]. However, our analysis suggests that the spatial periodicity of map, estimated from rat RGC mosaics data, is not small enough to generate a salt-and-pepper structure (Fig. 52B, C). To address this issue, here we suggest that feedforward convergent wiring between retina and V1 is a crucial factor that decides the structure of orientation map.

To find a convergence condition that develops salt-and-pepper map, we modulated two parameters in our simulations: (1) the convergence range of V1 cells and (2) the sampling ratio of RGCs within the range. The regularity of orientation map was estimated from the measurement of the preferred orientation difference between local neurons (Fig. 52D). We found that a salt-and-pepper map was developed with low sampling ratio and large convergence range, while a smooth map was developed when convergence range was relatively small and sampling ratio was high (Fig. 52E). To further analyze the map structure generated by our model, we compared the profile of correlation between local receptive fields structure in our simulated V1 map to the previous observation in animal experiment [2]. We confirmed that our salt-and-pepper map model well matched the statistics of observed experimental data.

**Conclusions** Our result suggests that a salt-and-pepper map can be developed by sparse and long-range convergence in feedforward wiring, while smooth map can be developed by localized convergence. We suggest that the condition of feedforward convergence between retina and V1 is a critical factor to determine the structure of orientation map.

**References**

1. Paik S-B, Ringach DL. Retinal origin of orientation maps in visual cortex. Nat Neurosci. 2011;14:919–25.
2. Bonin V, Histed MH, Yurgenson S, Reid RC. Local diversity and fine-scale organization of receptive fields in mouse visual cortex. J Neurosci. 2011;31:18506–21.

## P86 Computational method classifying neural network activity patterns for imaging data

### Min Song^1,2^, Hyeonsu Lee^1^, Se-Bum Paik^1,2^

#### ^1^Department of Bio and Brain Engineering, KAIST, Daejeon 34141, Republic of Korea; ^2^Program of Brain and Cognitive Engineering, KAIST, Daejeon 34141, Republic of Korea

##### **Correspondence:** Min Song - night@kaist.ac.kr, Hyeonsu Lee - hslee9305@kaist.ac.kr

*BMC Neuroscience* 2016, **17(Suppl 1)**:P86

In neural imaging data, various types of spatio-temporal activity patterns are observed which may reflect dynamic features of information processing in the brain [1, 2]. Classification of these patterns is required to analyze the brain activity further. However, development of analysis tool for spatio-temporal neural activity pattern has been regarded difficult because of highly complex connections between neurons and nonlinearity of activity patterns. In this study, we suggest a novel method of classifying activity patterns in two aspects: spatial geometry and temporal dynamics. We show that our method efficiently categorizes complicated spatio-temporal patterns in brain.

**Fig. 53:**
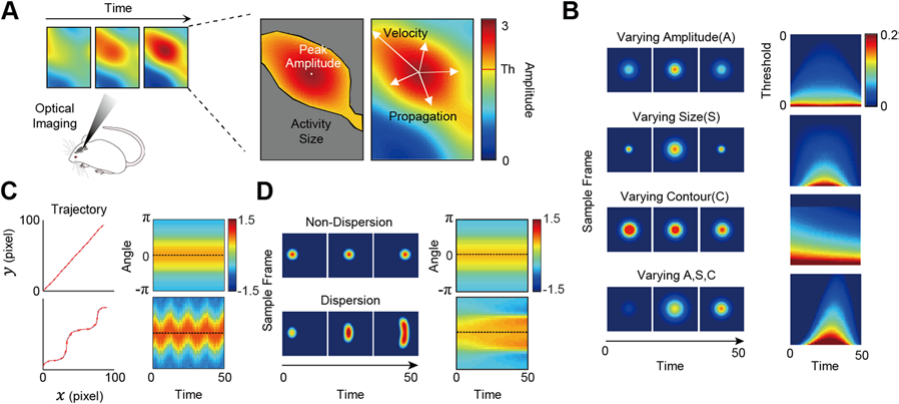
A novel index effectively describes different neural activity patterns obtained from imaging. **A** Neural activity obtained from optical imaging could be analyzed with appearance and propagation. **B** Appearance index of four distinct sample. **C** Propagation index of straight trajectory (*top*) and curved trajectory (*bottom*). **D** Propagation Index of non-dispersive sample (*top*) and dispersive sample (*bottom*)

First, we defined meaningful activity as salient distribution of highly activated parts. Its spatial feature could be described by size and peak amplitude (Fig. 53A), temporal feature by velocity and dispersion of activity (Fig. 53A). Thus, we designed geometric profile as two-dimensional profile of instantaneous neural activity, by measuring the topography of supra-threshold area with a shifting threshold. This profile contains the information of size, peak amplitude, and geometric contours of meaningful activity. With this method, we could readily estimate similarity or correlation of different activities in terms of size, peak amplitude, and amplitude contour (Fig. 53B).

Next, we defined propagation profile as a characteristic of temporal displacement of activity on each direction against time and angle axis. We measured trajectory and speed of activity using a normalized cross-correlation. This profile intuitively shows dominant trajectory, speed change and dispersion of the activity: how disperse to every direction. So we can compare activities: whether the activity is moving straight or curved trajectories, or whether the activity propagation is accelerating or not (Fig. 53C).

Our new method can easily perform not only classification of overall dynamics in brain, but also a simplified description of complex patterns (Fig. 53D), that may be applicable to the analysis of various kinds of brain imaging data.

## P87 Symmetry of spike-timing-dependent-plasticity kernels regulates volatility of memory

### Youngjin Park^1^, Woochul Choi^1,2^, Se-Bum Paik^1,2^

#### ^1^Department of Bio and Brain Engineering, Korea Advanced Institute of Science and Technology, Daejeon 34141, Republic of Korea; ^2^Program of Brain and Cognitive Engineering, Korea Advanced Institute of Science and Technology, Daejeon 34141, Republic of Korea

##### **Correspondence:** Youngjin Park - yodamaster@kaist.ac.kr

*BMC Neuroscience* 2016, **17(Suppl 1)**:P87

**Fig. 54:**
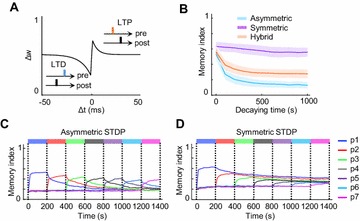
Different learning rules reproduce volatile/nonvolatile memory system. **A** Spike timing dependent plasticity. **B** Memory decaying properties of different learning rule. Poisson spikes are given for 1000 s to simulate decaying environment. **C**, **D** Multiple patterns was given to the system every 200 s. **C** Memory performance of each pattern in AS memory system. **D** Memory performance of each pattern in SS memory system

Synaptic plasticity is considered the core mechanism of learning and memory [1]. However, how plasticity can specifically modulate synaptic connections to generate short term or long term memory has not been understood completely. Here we introduce a theoretical model which suggests that a key mechanism of short term and long term memory can be implemented by a small difference in spike-timing-dependent-plasticity (STDP) rule. (Fig. 54A).

To test our idea, we designed simulations using a model feedforward neural network where two types of synaptic plasticity are implemented; asymmetric STDP (AS) [2] and symmetric STDP (SS). We defined the memory as the ability of a system to retrieve a consistent response spike pattern when we repeatedly introduce an identical pattern of spikes, and then we examined the performance of the system in terms of memory sustainability and appendability.

In our simulations, a network with AS showed performances similar to short-term memory while a network with SS showed long-term memory like properties. Memory in an AS Network decayed as a function of time, while memory in a SS network did not show a noticeable decay (Fig. 54B). Moreover, when a new input pattern was given to the network in addition to old memory, AS system replaced old memory with new memory pattern (Fig. 54C), while SS system maintained the old memory together with a newly trained memory (Fig. 54D).

Based on our findings, we suggest a new memory system called hybrid memory that is capable of showing intermediate properties between a long-term memory and a short-term memory (Fig. 54B, hybrid). This model suggests that transition between short term and long term memory might not be discrete but gradual.

**Conclusions** We have shown that our model network can implement different types of memory performance from the variation of plasticity, or learning rule. Our results imply that the various types of memory may be originated from a small difference in the shape of STDP kernel.

**References**

1. Bliss TV, Collingridge GL. A synaptic model of memory: long-term potentiation in the hippocampus. Nature. 1993;361(6407): 31–9.
2. Gütig R, Aharonov R, Rotter S, Sompolinsky H. Learning input correlations through nonlinear temporally asymmetric Hebbian plasticity. J Neurosci. 2003;23(9):3697–3714.

## P88 Effects of time-periodic coupling strength on the first-spike latency dynamics of a scale-free network of stochastic Hodgkin–Huxley neurons

### Ergin Yilmaz^1^, Veli Baysal^1^, Mahmut Ozer^2^

#### ^1^Department of Biomedical Engineering, Bülent Ecevit University, Zonguldak 67100, Turkey; ^2^Department of Electrical and Electronics Engineering, Bülent Ecevit University, Zonguldak 67100, Turkey

##### **Correspondence:** Ergin Yilmaz - erginyilmaz@yahoo.com

*BMC Neuroscience* 2016, **17(Suppl 1)**:P88

**Fig. 55:**
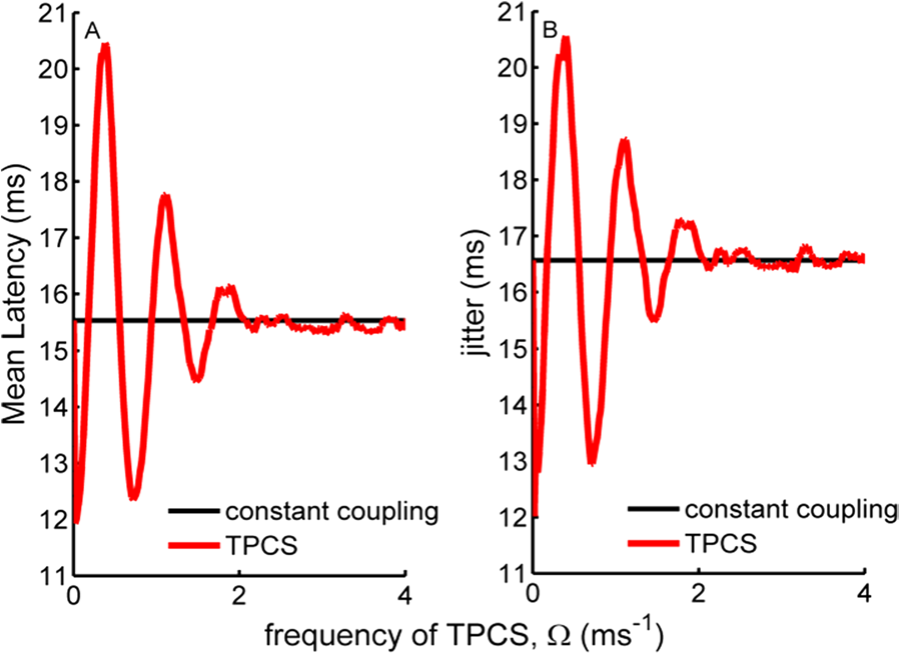
The statistics of the first-spike occurrence times (amplitude of TPCS ε_0_ = 0.2, cell size S = 100 μm^2^, frequency of suprathreshold signal f = 20 Hz and amplitude of it A = 4μA/cm^2^). **A** Mean latency of the network, **B** jitter of the network

It is still not known clearly which encoding mechanism that neurons utilize for coding of sensory information. One of the proposed encoding mechanism is latency coding which suppose that first-spike latency conveys much of the information about the stimulus. In this context, Pankratova et al. [1] studied the effects of noise on the first-spike latency dynamics of stochastic the Hodgkin–Huxley (HH) neuron, and obtained a bell-shaped dependence of mean response time of the neuron on noise intensity, emerging a phenomenon called “noise delayed decay” (NDD). Later, this finding have been extensively studied by using complex neuronal networks [2]. On the other hand, neurons exchange information via coupling at the special location called synapse. Thus, coupled neurons in networks play a decisive role on the phenomenon occurring in neuronal networks. Majority of the studies examining the NDD effect assume that coupling strength among coupled neurons is constant and the fact that synapses are plastic, that is, coupling strength among neurons can change with time, have been neglected. To present the effects of plasticity or time-varying-coupling strength Birzu et al. [3] studied the effects of time-periodic coupling strength (TPCS) on the firing dynamics of a globally coupled array of FHN neurons. Here, our aim is to present the effects of the frequency of TPCS on the NDD phenomenon in a scale-free network of HH neurons. We construct the network with N = 200 neurons modeled by a stochastic HH equation including ion channel noise, and average degree of k_avg_ = 4. We consider that the coupling strength among coupled units changes with time-periodic fashion as proposed in [3]. To measure the mean latency and jitter of the network, the first-spike times of each neuron are recorded. For the comparison purpose, we give the constant coupling strength effect on the latency dynamics of scale-free network. Obtained result are depicted in Fig. 55.

**Conclusions** It is seen that mean latency and jitter of the first-spike times exhibit a damped sine wave dependence on the frequency of TPCS, indicating that TPCS can significantly increase or decrease the latency time which passes until sensing of the suprathreshold stimulus by each neuron at fixed intensity of channel noise (S = 100 μm^2^). The frequencies of TPCS greater than ω = 2 m s^−1^ are not significantly affect the mean latency and jitter of the network, as compared to the constant coupling strength. As a result, with finely tuned values of the frequency of TPCS input signal detection performance of the scale-free network can be prominently increased by mitigating the response time of the each neuron.

**References**

1. Pankratova EV, Polovinkin AV, Mosekilde E. Resonant activation in a stochastic Hodgkin–Huxley model: interplay between noise and suprathres-hold driving effects. Eur Phys J B. 2005;45:391–7.
2. Yilmaz E. Impacts of hybrid synapses on the noise-delayed decay in scale-free neural networks. Chaos Solitons Fractals. 2014;66:1–8.
3. Birzu A, Krischer K. Resonance tongues in a system of globally coupled FitzHugh-Nagumo oscillators with time-periodic coupling strength. Chaos. 2010;20(4):043114.

## P89 Spectral properties of spiking responses in V1 and V4 change within the trial and are highly relevant for behavioral performance

### Veronika Koren^1,2^, Klaus Obermayer^1,2^

#### ^1^Institute of Software Engineering and Theoretical Computer Science, Technische Universitaet Berlin, Berlin, 10587, Germany; ^2^Bernstein Center for Computational Neuroscience Berlin, Humboldt-Universitaet zu Berlin, Berlin, 10115, Germany

##### **Correspondence:** Veronika Koren - veronika.koren@bccn-berlin.de

*BMC Neuroscience* 2016, **17(Suppl 1)**:P89

Linking sensory coding and behavior is a fundamental question in neuroscience. We have addressed this issue in behaving monkey visual cortex (areas V1 and V4) while animals were trained to perform a visual discrimination task in which two successive images (target and test stimuli, with a delay period in between) were either rotated with respect to each other or were the same. We hypothesized that animal’s performance in the visual discrimination task depends on the quality of stimulus coding in visual cortex. We tested this hypothesis by investigating the power spectral density of spiking signal from single neurons (spectra) and of pairs of neurons (cross-spectra) in relation to correct and incorrect behavioral responses. Our analysis shows that spectral properties systematically change with behavioral performance. Correct responses are associated with significantly higher spectra during the delay period. Cross-spectra of correct responses are significantly lower during the target period but significantly higher afterwards (delay period and test period). Spectral properties of single neurons and even more of pair-wise interactions therefore change within the trial, presumably following functional demands of stimulus processing in different epochs of the trial. Interestingly, differential dynamics in visual cortex sustains successful versus unsuccessful behavioral performance.

Preprocessing methods are used in order to avoid biases due to limited measurement time. The spike train is multiplied with Hanning window for low frequencies up to 22 Hz and with Slepian multitapers for frequencies between 24 and 140 Hz [1]. We use 300 ms window of sustained activity during stimulus periods and 500 ms window of activity during the delay period. Spectrum and cross spectrum are computed with Fast Fourier transform (Matlab, Mathworks). The cross-spectrum being a complex function, we consider its absolute value. Spectra are averaged in bins of 6 Hz. The variance and the covariance of the spiking signal are computed as sums over frequencies of auto and cross-spectra, respectively, up to the cut-off frequency (140 Hz).

Auto spectra are significantly higher in correct compared to incorrect trials in most of frequency bands in both V1 and V4 areas (p < 0.05 in 21 and 22 out of 24 frequency bands in V1 and V4, respectively, one-tailed sign-rank test). Consistently, the variance is significantly higher for correct responses (p < 10^−4^ in V1 and p = 0.0014 in V4. Cross-spectra are lower in correct trials during target period (18 and 10 out of 24 frequency bands are significant in V1 and V4, respectively, no significant effect in remaining bands) but higher in delay period (11 and 20 bands are significant in V1 and V4, respectively, no effect in remaining bands) and test period (19 and 13 significant bands in V1 and V4, respectively, no effect in remaining bands). Consistently, the covariance is significantly lower for correct responses during target stimulus (p = 0.0003, p = 0.0002 in V1 and V4) and higher during the delay (p < 10^−4^ in V1 and V4) and the test stimulus (p < 10^−4^ in V1 and V4). Our results show that spectra and cross spectra change during behavioral task and that spectral information in visual cortex might be highly relevant for behavioral performance.

**References**

1. Fries P, Womelsdorf T, Oostenwald R, Desimone R. The effects of visual stimulation and selective visual attention on rhythmic neuronal synchronization in Macaque area V4. J Neurosci. 2008;28(18):4823–35.
2. Womelsdorf T, Schoffelen JM, Oostenveld R, Singer W, Desimone R, Engel AK, Fries P. Modulation of neuronal interactions through neuronal synchronization. Science. 2007;316:1609–12.
3. Appel W. Mathematics for physics & physicists. Oxfordshire: Princeton University Press; 2007.
4. Gutnisky DA, Dragoi V. Adaptive coding of visual information in neural populations. Nature. 2008;452(7184):220–4.

## P90 Methods for building accurate models of individual neurons

### Daniel Saska^1^, Thomas Nowotny^1^

#### ^1^School of Engineering and Informatics, Sussex Neuroscience, University of Sussex, Falmer, Brighton BN1 9QJ, UK

##### **Correspondence:** Daniel Saska - research@saska.io

*BMC Neuroscience* 2016, **17(Suppl 1)**:P90

Formulating predictive models of single neuron dynamics has become a challenge taken up by many researchers ever since Hodgkin and Huxley published their widely accepted phenomenological model of electrophysiological dynamics of the squid giant axon [1]. Advances include, amongst others, modelling complex cells (such as cells of the stomatogastric ganglion (STG) in lobster and crab [2]) or increasingly automated modelling methods [3]. However, for each problem solved, new ones emerge. One such problem has been pointed out by Golowasch et al. [4] who discovered that averaging multiple measurements from the same cell type can produce models that fail to reproduce the behaviour of the target cells. This issue does not only affect methods that rely on averaging to achieve better signal-to-noise ratio but more generally all methods that examine ion channels in separate preparations. This includes classical voltage clamp, in which different ionic conductances are measured in separate individual cells because many pharmacological blockers cannot be fully reversed.

We here propose a different approach for parameter estimation aiming to build a model based on data from a single, individual cell. The proposed method consists of the consecutive use of a voltage clamp like protocol and parameter estimation in a current clamp mode. For the ‘voltage clamp protocol’ we use genetic algorithms (GA) to evolve a set of ‘highlighting’ voltage waveforms and specific observation windows, so that the resulting currents within the windows depend on a highlighted parameter but not so much on the values of other parameters. These parameter-specific waveforms are then applied to a live neuron (so far in simulation) and the resulting currents are observed and fitted with another GA, focusing on the highlighted parameters for each of the voltage waveforms. The resulting model is then transferred into current clamp, where the parameters are again estimated using a GA. The neuron models in the GA population are coupled to the observed cell to achieve a degree of synchronization and so smooth the error landscape. The coupling is reduced adiabatically until the model neurons and experimental cell remain synchronized with (virtually) no coupling.

**Fig. 56:**
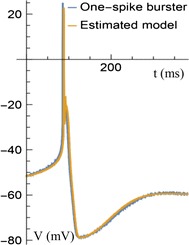
One-spike burster and estimated model as in Table 1

We found that combining voltage and current clamp works particularly well since the fitness landscape in voltage clamp has few local minima but is fairly shallow whereas the opposite is true for the current clamp mode. We can hence find approximate parameter values from arbitrary initial guesses in our ‘voltage clamp’ mode and once the parameters are in the right area, they can be refined in current clamp. Our method can produce accurate models of cells in the crab STG for the cases of one, two, three and four-spike bursters. Table 1 shows the resulting parameter values for the example of a one-spike burster cell illustrated in Fig. 56.

**Table 1:** Estimated parameters for one-spike burster STG cell

fPar	C	g_Na_	E_Na_	g_Kd_	E_Kd_	g_A_	E_A_	g_Ca_	Ca_0_	Ca_f_	Ca_t_	g_KCa_	E_KCa_	g_leak_	E_leak_
Min	0.1	0	0	0	−100	0	−100	0	0.01	14	20	0	−100	0	−100
Max	10	800	100	200	0	75	0	5	0.1	16	250	300	0	1	0
Real	0.628	50	50	100	−80	5	−80	4	0.05	14.96	200	250	−80	0.01	−50
Estim.	0.613	62.07	39.05	95.77	−76.09	4.622	−88.59	3.912	0.0438	15.59	194.7	246.2	−80.22	0.0106	−50.15

**References**

1. Hodgkin AL, Huxley AF. A quantitative description of membrane current and its application to conduction and excitation in nerve. J Physiol. 1952;117(4):500–44.
2. Liu Z, Golowasch J, Marder E, Abbott LF. A model neuron with activity-dependent conductances regulated by multiple calcium sensors. J Neurosci. 1998;18(7):2309–20.
3. Willms AR. NEUROFIT: software for fitting Hodgkin–Huxley models to voltage-clamp data. J Neurosci Methods. 2002;121(2):139–50.
4. Golowasch J, Goldman MS, Abbott LF, Marder E. Failure of averaging in the construction of a conductance-based neuron model. J Neurophysiol. 2002;87(2):1129–31.

## P91 A full size mathematical model of the early olfactory system of honeybees

### Ho Ka Chan^1^, Alan Diamond^1^, Thomas Nowotny^1^

#### ^1^School of Engineering and Informatics, University of Sussex, Falmer, Brighton, BN1 9QJ, UK

##### **Correspondence:** Ho Ka Chan - hc338@sussex.ac.uk

*BMC Neuroscience* 2016, **17(Suppl 1)**:P91

Experimental measurements often can only provide limited data from animals’ sensory systems. As a result, data driven models are similarly limited. However, in order to make biologically relevant predictions, it is important to consider inputs representative of the full sensory input space. Here we present a full size model of the early olfactory system of honeybees that extrapolates inputs from the limited subset of available experimental observations.

Our model comprise olfactory receptor neurons (ORNs), local neurons (LNs) and projection neurons (PNs) organized in 160 glomeruli. The ORN response patterns are generated using a set of ordinary differential equations describing the binding and activation of receptors as in [2]. The parameters for these processes are chosen to match the statistical distribution of experimental observed quantities in [3, 4] as well as the statistics of asymptotic responses to time-invariant odours at high concentration observed in calcium imaging of glomeruli with bath-applied Ca dyes [5]. To generate the PN responses, we considered a network in which PNs and LNs both receive excitatory input from ORNs in the same glomerulus and inhibitory input from LNs in all glomeruli. The connectivity between PNs and LNs is based on the correlation between the activities of their respective glomeruli as in [5]. A rate model, derived using the leaky integrate-and-fire model with the assumption of constant input, is used to determine the input–output relationship.

**Fig. 57:**
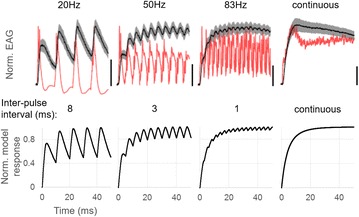
Experimental ORN responses to stimulus measured by electro-antennogram recordings in [5] (*top*, *black line*) is qualitative similar to the average normalized ORN responses to stimulus (1-hexanol at concentration 0.1 M) predicted by our model (*bottom*)

We tested our ORN model with continuous stimuli and short pulses. The average normalized ORN responses to a chemical stimulus are qualitatively similar to that of biological ORNs measured by electro-antennogram recordings [4] as shown in Fig. 57, except that the time scale of response latency is a little smaller in the model. This can be explained by the lack of temporal filtering of input conductance to output spiking in our rate model. The responses of PNs driven by ORN activity can be compared to calcium imaging data with back-filled PNs [6], which confirms that the responses of our model PNs can replicate key features of those of biological PNs. With appropriate data, our model can be generalized to the early olfactory systems of other insects. It hence provides a possible basis for future numerical studies of olfactory processing in insects.

**Acknowledgements:** This work was supported by HFSP, RGP0053/2015 and EPSRC, EP/J019690/1.

**References**

1. Galizia CG, Sachse S, Rappert A, Menzel R. The glomerular code for odor representation is species specific in the honeybee *Apis mellifera*. Nat Neurosci. 1999;2(5):473–8.
2. Grémiaux A, Nowotny T, Martinez D, Lucas P, Rospars J-P. Modelling the signal delivered by a population of first-order neurons in a moth olfactory system. Brain Res. 2012;1434:123–35.
3. Rospars J-P, Lansky P, Chaput M, Duchamp-Viret P. Competitive and noncompetitive odorant interactions in the early neural coding of odorant mixtures. J Neurosci. 2008;28(10):2659–66.
4. Szyszka P, Gerkin RC, Galizia CG, Smith BH. High-speed odor transduction and pulse tracking by insect olfactory receptor neurons. Proc Natl Acad Sci USA. 2014;111(47):16925–30.
5. Linster C, Sachse S, Galizia CG. Computational modeling suggests that response properties rather than spatial position determine connectivity between olfactory glomeruli. J Neurophysiol. 2005;93(6):3410–17.
6. Ditzen M. Odor concentration and identity coding in the antennal lobe of the honeybee *Apis mellifera* (PhD thesis). Berlin: Freie Universität Berlin; 2005.

## P92 Stimulation-induced tuning of ongoing oscillations in spiking neural networks

### Christoph S. Herrmann^1^, Micah M. Murray^2^, Silvio Ionta^2^, Axel Hutt^3^, Jérémie Lefebvre^4^

#### ^1^Research Center Neurosensory Science, Carl-von-Ossietzky University Oldenburg, Oldenburg, Germany; ^2^The Laboratory for Investigative Neurophysiology (The LINE), Department of Clinical Neurosciences and Department of Radiology, University Hospital Center and University of Lausanne, Lausanne 1011, Switzerland; ^3^Deutscher Wetterdienst, 63067 Offenbach, Germany; ^4^ Krembil Research Institute, University Health Network, Toronto, Ontario M5T 2S8, Canada

##### **Correspondence:** Jérémie Lefebvre - jeremie.lefebvre@uhnresearch.com

*BMC Neuroscience* 2016, **17(Suppl 1)**:P92

Rhythmic neural activity is believed to play a central role in neural computation. Oscillatory brain activity has been associated with myriad functions such as homeostasis, attention, and cognition as well as neurological and psychiatric disorders, including Parkinson’s disease, schizophrenia, and depression [1]. Numerous studies have shown that that non-invasive stimulation, such as repetitive transcranial magnetic stimulation (rTMS) and Transcranial Alternating Direct Current Stimulation (TACS), provide the means of modulating large-scale oscillatory brain dynamics by perturbing and/or entraining both resting state and task activity [2]. These stimulation-induced perturbations of neural oscillations have been shown to alter cognitive performance and perception, effects that are further known to depend on brain state prior and during stimulation [3]. Yet, the surge of interest in these approaches is compromised by the existence of complex interference patterns between exogenous and endogenous dynamics.

To better understand oscillatory responses evoked during rhythmic stimulation, we simulated a spiking cortical network built of excitatory and inhibitory cells, expressing resting state alpha synchrony and subjected to pulsatile forcing at frequencies in the range of 1–100 Hz. Varying stimulation parameters—such as frequency and amplitude—we evaluated the influence of stimulation on the spectral properties of the network’ global neuroelectric output. The network was composed of recurrently connected Poisson neurons with propagation delays, linear adaptation, spatially profiled and sparse synaptic connections and noisy inputs. To model exogenous influences, we used continuous trains of phasic pulses and stimulated the network globally (all neurons identically), to mimic TMS-like signals. For every stimulation condition, we also measured the neurons mean firing rate, the mean network spike coherence and non-linearity metric. Multiple spectral patterns could be observed in the network’s responses, both in the power and frequency domains, indicating a plurality of responses to shifts in stimulation frequency and/or amplitude. Network responses to slower/weaker stimulation were expectedly found to be shaped by entrainment and resonance: resonance curves defining the amplitude of the system’s responses were revealed, alongside the characteristic Arnold tongues, where stimulus-locking can be achieved. The individual firing rates of the neurons and resulting spike coherence (assessing the degree of spiking synchronization) were both strongly tied to the stimulation forcing. In contrast, for stimulation frequencies higher than 50 Hz, a different mechanism was found to dominate the network dynamics: stimulation pulses shaped the system’s response via a *non*-*linear acceleration* (NLA) on ongoing oscillatory activity. The network peak frequency was gradually shifted, leading to a transition from the alpha to the beta band, and for forcing parameters that did not recruit neither resonance nor entrainment. Also, NLA led the network in a state of weak oscillatory power, where individual neurons were found in a state of intense, irregular spiking. By investigating closely the network non-linear interactions for each stimulation conditions, we found that high-frequency forcing induces synergetic and non-linear, large-scale effects [4]. Our results provide new computational perspectives about the response of synchronous spiking neural networks in which firing rates, spike coherence and emergent oscillatory activity can be exogenously modulated using dynamic inputs. Taken together, our results suggest that the action of forcing on oscillating neural systems must be regarded as strongly non-linear, and input features must be considered as control parameters.

**References**

1. Wang X-J. Neurophysiological and computational principles of cortical rhythms in cognition. Physiol Rev. 2010;90:1195–1268.
2. Thut G, et al. The functional importance of rhythmic activity in the brain. Curr Biol. 2012;22:658–63.
3. Neuling T, et al. Orchestrating neuronal networks: sustained after-effects of transcranial alternating current stimulation depend upon brain states. Front Hum Neurosci. 2013;7:161.
4. Lefebvre J, et al. Stimulus statistics shape oscillations in non-linear recurrent neural networks. J Neurosci. 2015;35(7):2895–903.

## P93 Decision-specific sequences of neural activity in balanced random networks driven by structured sensory input

### Philipp Weidel^1^, Renato Duarte^1,4,5^, Abigail Morrison^1,2,3,4^

#### ^1^Institute of Advanced Simulation (IAS-6) & Institute of Neuroscience and Medicine (INM-6) & JARA BRAIN Institute I, Jülich Research Center, 52425 Jülich, Germany; ^2^Institute of Cognitive Neuroscience, Faculty of Psychology, Ruhr-University Bochum, 44801 Bochum, Germany; ^3^Simulation Laboratory Neuroscience – Bernstein Facility for Simulation and Database Technology, Institute for Advanced Simulation, Jülich Aachen Research Alliance, Jülich Research Center, Jülich, Germany; ^4^Bernstein Center Freiburg, Albert-Ludwig University of Freiburg, Freiburg im Breisgau, 79104, Germany; ^5^Institute for Adaptive and Neural Computation, School of Informatics, University of Edinburgh, EH8 9AB, UK

##### **Correspondence:** Philipp Weidel - p.weidel@fz-juelich.de

*BMC Neuroscience* 2016, **17(Suppl 1)**:P93

Perceptual decision-making is an intricate process implicating the coordinated activity of multiple brain areas [1, 2]. Recent experimental studies demonstrate the existence of a complex interplay between decision-related neural events and transient working memory processes [1], implemented by distributed circuits where specific sub-populations appear to be differentially involved in the evidence accumulation process and subsequent behavioral outcomes [2]. This results in observable divergences in choice-specific neuronal dynamics, unfolding as reproducible trajectories throughout the network’s state-space [1] and hinting at the dissipative nature of the underlying dynamical system, which executes cognitively relevant processing through transient trajectories.

Despite this evidence, the majority of modeling studies addressing reward-modulated decision-making tend to simplify the formalization of environmental representations in the cortex as stable, attractor states corresponding to discrete environmental states [3]. Even models involving transient-based computations often simplify sensory stimuli to a discrete set of inputs transduced as stochastic point processes [4]. These simplifications potentially draw an incomplete picture of neural dynamics and therefore provide limited insights into the true nature of computation in neural circuits.

To overcome this issue, we take one step towards realistic in silico experimental settings by using structured virtual environments to obtain rich sensory input to drive model neural systems using the ROS-MUSIC toolchain [5]. It allows us to simulate robotic agents in virtual 3D environments performing a realistic perceptual decision task, which can be directly equated to experimental data. The robotic simulation generates realistic and structured sensory data which is encoded to spiking neural activity using a nonlinear encoding process, as formalized in [6]. The encoded sensory data is then used as input to a balance recurrent neural circuit.

In this study, we investigate the emergent dynamical features of neural activity when the agent is navigating a virtual T-maze. We observe decision-specific sequences of neural activity akin to experimental evidence [1], revealing possible processing strategies employed by the neural substrate. Furthermore, we investigate the role of different adaptation/plasticity mechanisms in shaping the system’s dynamics. In order to equate our results with those of other studies, we attempt to partition the network state-space into discrete activity clusters, which carry relevant information that could potentially be used to drive reinforcement learning algorithms.

**Acknowledgements:** We acknowledge partial support the Helmholtz Alliance through the Initiative and Networking Fund of the Helmholtz Association and the Helmholtz Portfolio theme “Supercomputing and Modeling for the Human Brain”, EuroSPIN and the German Federal Ministry for Education and Research (BMBF Grant 01GQ1343).

**References**

1. Harvey CD, Coen P, Tank DW. Choice-specific sequences in parietal cortex during a virtual-navigation decision task. Nature. 2012;484(7392):62–8.
2. Shadlen MN, Kiani R. Decision making as a window on cognition. Neuron. 2013;80(3):791–806.
3. Jitsev J, Morrison A, Tittgemeyer M Learning from positive and negative rewards in a spiking neural network model of basal ganglia. In: The 2012 international joint conference on neural networks (IJCNN). IEEE; 2012.
4. Duarte R, Morrison A. Dynamic stability of sequential stimulus representations in adapting neuronal networks. Front Comput Neurosci. 2014;8(124).
5. Weidel P, Duarte R, Djurfeldt M, Morrison A. ROS-MUSIC toolchain (in preparation).
6. Eliasmith C, Anderson CH. Neural engineering: computation, representation, and dynamics in neurobiological systems. MIT Press; 2004.

## P94 Modulation of tuning induced by abrupt reduction of SST cell activity

### Jung H. Lee^1^, Ramakrishnan Iyer^1^, Stefan Mihalas^1^

#### ^1^Allen Institute for Brain Science, Seattle, WA 98109, USA

##### **Correspondence:** Jung H. Lee - jungl@alleninstitute.org

*BMC Neuroscience* 2016, **17(Suppl 1)**:P94

Inhibitory interneurons have been considered pivotal in orchestrating pyramidal neurons. Indeed, the optogenetic perturbation of inhibitory cell types confirmed its validity. Recent studies [1, 2] have found that the optogenetic stimulation of somatostatin positive (SST) interneurons, one of the three major inhibitory types, sharpens the tuning of visual neurons, but its effect was conspicuous only when the optogenetic activation of SST cells was turned off abruptly. Specifically, with 4-s presentation of visual stimuli, the 1-s activation of SST cells resulted in a sharper tuning, whereas 4-s activation did not induce significant sharpening [2], which leads to a question: “Why does the length of optogenetic stimulation render such a striking difference?” Lee et al. suggested that the 1-s activation sharpens the tuning curve due to the rebound activity of PV cells, and El-Boustani et al. suggested the reduction of co-activation between PV and Pyr cell activity; see Ref. [2] for the details.

In our study, we investigate the potential mechanisms underlying the disparate effects between short and long activations of SST cells by using the firing rate equations that expresses the interactions among Pyr, SST and PV cells conveyed via cell-type specific connections reported by Pfeffer et al. [3]. Our model consists of five populations: two pyramidal populations (Pyr1, 2), two PV cell populations (PV1, 2) and SST cell population. We assume that Pyr1 and Pyr2 in close proximity respond to preferred and non-preferred stimuli, respectively. The two pyramidal populations excite the shared SST cell population which sends inhibition back to them. Since SST cells are known to be connected to distant presynaptic pyramidal cells via long-horizontal connections [4], the two SST populations in close proximity would receive (almost) identical inputs, making the two SST populations redundant. PV1 and PV2, which receive identical external background inputs, interact with Pyr1 and Pyr2, respectively. Pyr1 and Pyr2 are not directly connected, but they can indirectly interact with each other through SST cell population.

Our model replicates the paradoxical finding that not 4-s activation of SST cells but 1-s activation leads to the sharper responses of V1 neurons. In our model, PV cells provide synchronized inhibition to pyramidal cells despite their distinctive receptive fields when SST cell activation is abruptly turned off. If SST cells are stimulated during the entire period of simulations (4 s), the induced synchronous inhibition from PV cells to pyramidal cells is not strong enough to induce sharper responses. We also found that this synchronous inhibition can be induced by the activation of VIP cells, raising the possibility that VIP cells regulate V1 neural responses with the proposed mechanism.

**Acknowledgements:** We wish to thank the Allen Institute founders, Paul G. Allen and Jody Allen, for their vision, encouragement and support.

**References**

1. Wilson NR, Runyan CA, Wang FL, Sur M. Division and subtraction by distinct cortical inhibitory networks in vivo. Nature. 2012;488(7411):343–8. doi:10.1038/nature11347.
2. Lee S-H, Kwan AC, Dan Y. Interneuron subtypes and orientation tuning. Nature. 2014;508(7494):E1–2. doi:10.1038/nature13128.
3. Pfeffer CK, Xue M, He M, Huang ZJ, Scanziani M. Inhibition of inhibition in visual cortex: the logic of connections between molecularly distinct interneurons. Nat Neurosci. 2013;16(8):1068–76. doi:10.1038/nn.3446.
4. Adesnik H, Bruns W, Taniguchi H, Huang ZJ, Scanziani M. A neural circuit for spatial summation in visual cortex. Nature. 2012;490(7419):226–31. doi:10.1038/nature11526.

## P95 The functional role of VIP cell activation during locomotion

### Jung H. Lee^1^, Ramakrishnan Iyer^1^, Christof Koch^1^, Stefan Mihalas^1^

#### ^1^Allen Institute for Brain Science, Seattle, WA 98109, USA

##### **Correspondence:** Jung H. Lee - jungl@alleninstitute.org

*BMC Neuroscience* 2016, **17(Suppl 1)**:P95

Vasoactive intestinal polypeptide positive (VIP) inhibitory interneurons are commonly found in the superficial layers of cortices [1]. They are distinct from other major cortical inhibitory cell types in terms of connectivity and cellular mechanisms and exclusively inhibit somatostatin (SST) cells in visual cortex. This property is consistent with the interneuron-selective interneuron group, which has been proposed recently [2]. Indeed, bitufted cells in this group express VIP. Moreover, VIP cells have nicotinic receptors rarely found in SST and parvalbumin positive (PV) cells [3]. These recent studies lead to the hypothesis that VIP cells play unique functions in cortical areas, which can be supported with evidence. The optogenetic activation of cingulate cortex of mouse elicited a strong response in VIP cells of V1, suggesting the central roles of VIP cells in top-down gain control [4]. Also, VIP cells are nonspecifically depolarized when a mouse runs [5].

VIP cells disinhibit pyramidal cells by suppressing SST cell activity. That is, when VIP cells are activated, pyramidal cell activity increases due to reduction of inhibition from SST cells, which accounts for the gain modulation. However, the advantage of VIP cell activation induced by locomotion is not clear. We hypothesized that VIP cell activation leads to better perception of moving objects since all visual objects would appear to be in motion when a mouse runs. The strong surround suppression could prevent visual neurons from responding to those effective movements. In this sense, VIP cell activation may be beneficial to the mouse running, as the enhanced VIP cell activity reduces surround suppression.

Here we use a computational model of V1 consisting of multiple cortical columns to address if VIP cell activation can enhance perception on moving objects. Our computational model is based on an earlier multiple column model [6], and we refined it by incorporating VIP, SST and PV cells into the superficial layers of the model. To build this refined model, we used two strategies. First, we inferred the time course of synaptic events and the number of connections from both experimental data [7] and parameters from the earlier model. Second, we identified the minimal set of intercolumnar connections necessary for reproducing lateral interactions observed in visual cortex [8]. To examine whether the enhanced VIP cells could enhance responses to moving objects, we simulated a single moving object by stimulating the columns sequentially in the model. Our simulation results support our hypothesis: the column responses during sequential stimulation increase as we increase inputs to VIP cells.

**Acknowledgements:** We wish to thank the Allen Institute founders, Paul G. Allen and Jody Allen, for their vision, encouragement and support.

**References**

1. Rudy B, Fishell G, Lee S, Hjerling-Leffler J. Three groups of interneurons account for nearly 100 % of neocortical GABAergic neurons. Dev Neurobiol. 2011;71:45–61.
2. Jiang X, Shen S, Cadwelll C, Berence P, Sinz F, Ecker AS, et al. Principles of connectivity among morphologically defined cell types in adult neocortex. Science. 2015;350:62–4.
3. Kepecs A, Fishell G. Interneuron cell types are fit to function. Nature. 2014;505:318–26.
4. Zhang S, Xu M, Kamigaki T, Hoang Do JP, Chang W-C, Jenvay S, et al. Long-range and local circuits for top-down modulation of visual cortex processing. Science. 2014;345:660–5.
5. Fu Y, Tucciarone JM, Espinosa JS, Sheng N, Darcy DP, Nicoll RA, et al. A cortical circuit for gain control by behavioral state. Cell. 2014;156:1139–52.
6. Wagatsuma N, Potjans TC, Diesmann M, Sakai K, Fukai T. Spatial and feature-based attention in a layered cortical microcircuit model. PLoS One. 2013;8:e80788.
7. Pfeffer CK, Xue M, He M, Huang ZJ, Scanziani M. Inhibition of inhibition in visual cortex: the logic of connections between molecularly distinct interneurons. Nat Neurosci. 2013;16:1068–76.
8. Xu S, Jiang W, Poo M-M, Dan Y. Activity recall in a visual cortical ensemble. Nat Neurosci. 2012, 15:449–455.

## P96 Stochastic inference with spiking neural networks

### Mihai A. Petrovici^1,†^, Luziwei Leng^1,†^, Oliver Breitwieser^1,†^, David Stöckel^1,†^, Ilja Bytschok^1^, Roman Martel^1^, Johannes Bill^1^, Johannes Schemmel^1^, Karlheinz Meier^1^

#### Kirchhoff-Institute for Physics, University of Heidelberg, Germany

##### **Correspondence:** Mihai A. Petrovici - mpedro@kip.uni-heidelberg.de

^†^ Authors with equal contributions

*BMC Neuroscience* 2016, **17(Suppl 1)**:P96

Brains are adept at creating an impressively accurate internal model of their surrounding based on incomplete and noisy sensory data. Understanding this inferential prowess is not only interesting for neuroscience, but may also inspire computational architectures and algorithms for solving hard inference problems. Here, we give an overview of our work on probabilistic inference with brain-inspired spiking networks, their advantages compared to classical neural networks and their implementation in neuromorphic hardware.

In the neural sampling framework, we interpret spiking activity as sampling from distributions over binary random variables. By exploiting the dynamics of spiking neurons with conductance-based synapses, we have shown that their activation function can become symmetric in the high-conductance state, which in turn enables Glauber-like dynamics in ensembles of noise-driven LIF networks [1, 2]. This allows the straightforward construction of LIF networks that sample from previously defined probability distributions.

When the parameters of the distribution are not well-defined, they need to be learned from data. Due to their analogy to classical neural networks such as Boltzmann machines, LIF networks are amenable to the same learning algorithms and can be shown to match the performance of their equally-sized abstract counterparts when trained on classical machine-learning datasets such as MNIST. However, spiking neural networks endowed with short-term plasticity can travel more efficiently through their associated state space, allowing them to simultaneously become good generative and discriminative models of learned data, which is notoriously difficult with conventional techniques such as Gibbs sampling. This finding points towards a distinct advantage of spike-based computation and communication, which is relevant in any scenario where spiking neural networks need to be able to escape local attractors.

This computational advantage of spiking sampling networks can be further bolstered by emulation on an accelerated neuromorphic substrate. The core idea behind these devices is the direct emulation of biological neuronal dynamics in VLSI circuits. Such hardware can far surpass simulators running on conventional computing architectures both in terms of speed and power consumption, but with the caveat of having limited parameter precision, as well as other sources of disruptive noise [3, 4]. With some additional modifications, we have shown how LIF networks can become robust to certain types of parameter noise—both during training and during operation– thereby making them amenable to a neuromorphic implementation with an acceleration factor of 10^4^ compared to biological real-time.

An even more compelling argument for neuromorphic spike-based inference can be made when considering that learning (in particular, the simulation of synaptic plasticity) is by far the most time-consuming factor in simulations. In an effort to make expectation–maximization learning compatible with existing neuromorphic devices, we have developed a network model that can use double-exponential STDP with 4–6 bit weight resolution for learning and spike-based homeostasis for stabilization and robustness.

This research was supported by EU grants #269921 (BrainScaleS), #237955 (FACETS-ITN), #604102 (Human Brain Project) and the Manfred Stärk Foundation.

**References**

1. Petrovici MA, Bill J, Bytschok I, Schemmel J, Meier K. Stochastic inference with deterministic spiking neurons. arXiv preprint arXiv:1311.3211, 2013.
2. Petrovici MA, Bytschok I, Bill J, Schemmel J, Meier K. The high-conductance state enables neural sampling in networks of LIF neurons. BMC Neurosci. 20150;16(Suppl. 1):O2.
3. Petrovici MA, Vogginger B, Müller P, Breitwieser O, Lundqvist M, Muller L, Ehrlich M, Destexhe A, Lansner A, Schüffny R, et al. Characterization and compensation of network-level anomalies in mixed-signal neuromorphic modeling platforms. PloS One. 2014;9(10):e108590.
4. Pfeil T, Grübl A, Jeltsch S, Müller E, Müller P, Petrovici MA, Schmuker M, Brüderle D, Schemmel J, Meier K. Six networks on a universal neuromorphic computing substrate. Front Neurosci. 2013;7:11. ISSN 1662-453X. doi:10.3389/fnins.2013.00011.

## P97 Modeling orientation-selective electrical stimulation with retinal prostheses

### Timothy B. Esler^1^, Anthony N. Burkitt^1^, David B. Grayden^1^, Robert R. Kerr^2^, Bahman Tahayori^3^, Hamish Meffin^4^

#### ^1^NeuroEngineering Laboratory, Electrical & Electronic Engineering, The University of Melbourne, Parkville VIC 3010, Australia; ^2^IBM Research, Melbourne, Australia; ^3^Monash Institute of Medical Engineering, Monash University, Melbourne, Australia; ^4^National Vision Research Institute, Melbourne, Australia

##### **Correspondence:** Timothy B. Esler - tesler@student.unimelb.edu.au

*BMC Neuroscience* 2016, **17(Suppl 1)**:P97

One present challenge in electrical stimulation for epiretinal prostheses is how to avoid stimulating axons of passage in the nerve fiber layer (NFL) that originate from distant regions of the ganglion cell layer (GCL). Co-stimulation of target retinal ganglion cells and overlying axons results in irregular visual percepts, which can significantly limit perceptual efficacy [1, 2]. This research explores how the characteristic distributions of fiber orientation in different retinal layers result in differences between the activation of the axon initial segment and axons of passage. Specifically, axons of passage of retinal ganglion cells are characterized by a narrow distribution of fiber orientations, dominated by the direction of passage towards the optic disk. In contrast, proximal axons in the GCL tend to have a wider distribution of orientations.

A model of extracellular stimulation that captures the effects of neurite orientation has been developed using a modified version of the standard volume conductor model, known as the cellular composite model [3], embedded in a four layer model of the retina. The cellular composite model is used in this analysis as it addresses a number of limitations of conventional volume conductor models and more accurately captures the spatiotemporal properties of neural tissue.

By generalizing the model to allow for analysis of fibers with arbitrary orientations, simulations have been conducted to investigate the interaction of neural tissue orientation, electrode placement, and stimulation pulse duration and amplitude.

**Fig. 58:**
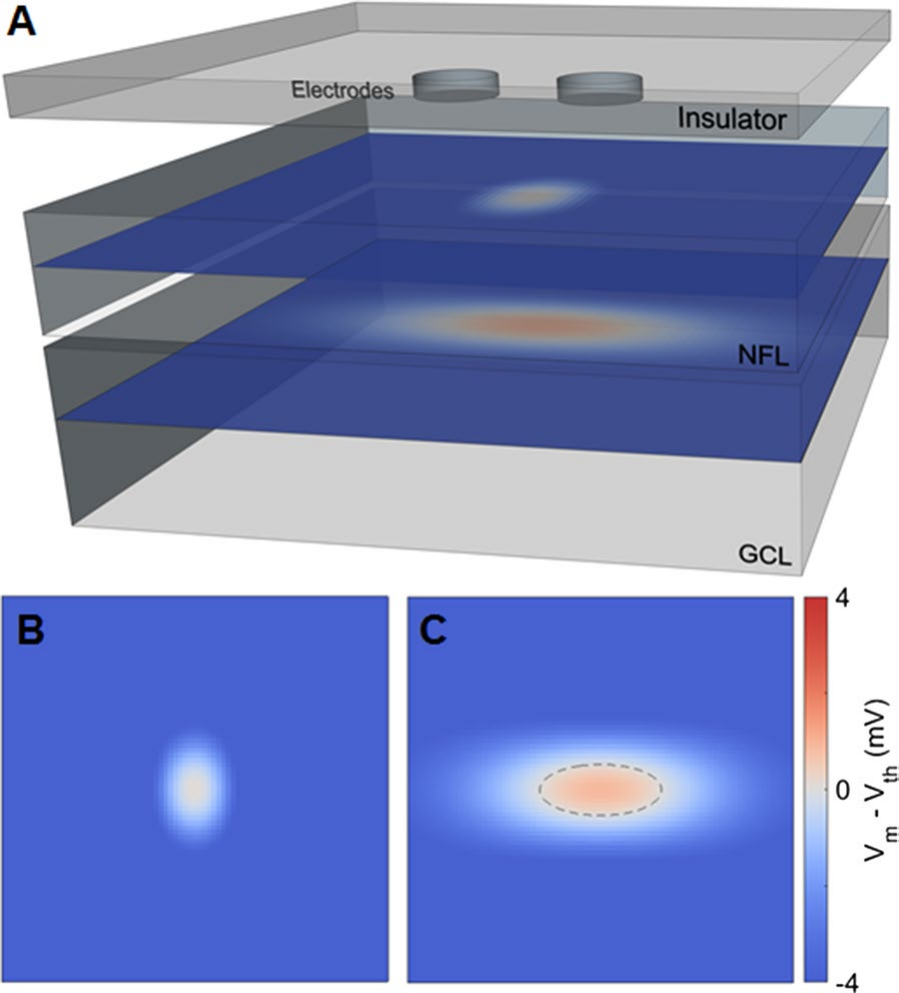
**A** Simulation geometry showing the four modeled layers: insulator (glass), vitreous, NFL, and GCL. Distance from membrane threshold in mV for **B** parallel axons in a plane in the NFL and **C** perpendicular axon initial segments in a plane in the GCL, when stimulated with a 300 µs biphasic pulse with electrode-retina separation of 400 µm. *Dotted contour* marks the threshold level

Through an exhaustive parameter search, a set of stimulation pulse durations, amplitudes and electrode positions are proposed to achieve selective activation of axon initial segments. Using appropriate multiple electrode configurations and higher frequency stimulation, preferential activation of the axon initial segment is shown to be possible for a range of realistic electrode-retina separation distances (Fig. 58).

These results establish a quantitative relationship between the time-course of stimulation and physical properties of the tissue, such as fiber orientation.

**References**

1. Fried SI, Lasker ACW, Desai NJ, Eddington DK. Axonal sodium-channel bands shape the response to electric stimulation in retinal ganglion cells. J Neurophysiol. 2009;101(4):1972–87.
2. Rattay F, Resatz S. Effective electrode configuration for selective stimulation with inner eye prostheses. IEEE Trans Biomed Eng. 2004;51(9):1659–64.
3. Meffin H, Tahayori B, Sergeev EN, Mareels IMY, Grayden DB, Burkitt AN. Modelling extracellular electrical stimulation: III. Derivation and interpretation of neural tissue equations. J Neural Eng. 2014;11(6):065004.

## P98 Ion channel noise can explain firing correlation in auditory nerves

### Bahar Moezzi^1^, Nicolangelo Iannella^1,2^, Mark D. McDonnell^1^

#### ^1^Computational and Theoretical Neuroscience Laboratory, School of Information Technology and Mathematical Sciences, University of South Australia, Australia; ^2^School of Mathematical Sciences, University of Nottingham, Nottingham, UK

##### **Correspondence:** Bahar Moezzi - bahar.moezzi@unisa.edu.au

*BMC Neuroscience* 2016, **17(Suppl 1)**:P98

Neural spike trains are commonly characterized as a Poisson point process. However, the Poisson assumption is a poor model for spiking in auditory nerve fibers because it is known that interspike-intervals display positive correlation over long time scales and negative correlation over shorter time scales. It has been suggested that ion channel opening and closing might not be well described by Markov models. Instead, fractal ion channel gating could be used to take into account the involvement of proteins in the conformational changes of sub-states in the channel gating kinetics. Using a detailed biophysical model, we tested the hypothesis that fractal ion channel gating is responsible for short and long term correlations in the auditory nerve spike trains.

We developed a biophysical model based on the well-known Meddis model of the peripheral auditory system [1]. We introduced biophysically realistic ion channel noise to an inner hair cell membrane potential model that includes (i) fractal fast potassium channels, (ii) deterministic slow potassium channels, and (iii) a stochastic Markov model for noisy calcium channels. We used Fano factor as a measure of firing correlation.

We showed that the resulting simulated Fano factor time curves have all the common attributes of the Fano factor of experimentally recorded spike trains in the auditory nerve fibers, except the time scale of corelation. Our model thus replicates macro-scale stochastic spiking statistics in the auditory nerve fibers due to modeling stochasticity at the micro-scale of potassium and calcium ion channels.

**Reference**

1. Meddis R. Auditory-nerve first-spike latency and auditory absolute threshold: a computer model. J Acoust Soc Am. 2006;119(1):406–17.

## P99 Limits of temporal encoding of thalamocortical inputs in a neocortical microcircuit

### Max Nolte^1^, Michael W. Reimann^1^, Eilif Muller^1^, Henry Markram^1^

#### ^1^Blue Brain Project, École Polytechnique fédérale de Lausanne (EPFL), Geneva, Switzerland

##### **Correspondence:** Max Nolte - max.nolte@epfl.ch

*BMC Neuroscience* 2016, **17(Suppl 1)**:P99

During naturalistic whisker motion, subsets of neurons in the same barreloid of the rat ventroposterior medial thalamus (VPM) respond synchronously with temporal precision to different kinetic features of whisker movement (spike-time coding) [1]. Multiple synchronously firing VPM cells can trigger temporally precise responses in the somatosensory cortex, such as those observed during full whisker deflection or active touch, but the minimum number of synchronously firing VPM cells needed to reliably drive the spiking of cortical cells is not known.

**Fig. 59:**
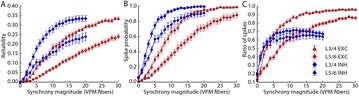
**A** Mean spike-timing reliability (similar correlation-based measure as in [3], but with firing rate adaption). The reliability of the VPM input is 0.55. **B** Mean probability of firing within 2–12 ms after the initial input VPM spike in each trial. **C** Mean ratio of spikes occurring within 2–12 ms after a VPM spike, out of all spikes. Mean of 30 (L3/4 excitatory), 50 (L5/6 exc.), 40 (L3/4 inhibitory) and 30 cells (L5/6 inh.) respectively

In this study, we use the Blue Brain Project’s digital reconstruction of a somatosensory microcircuit of a juvenile rat [2] to characterize how many synchronously firing VPM cells are needed to reliably drive individual cells of different morphological types in the rat somatosensory cortex. We activate an increasing number of synchronously firing VPM fibers (with in vivo VPM spike trains from experiments published in [1]) in both simulations of single cells, and simulations of the whole reconstructed microcircuit with only a small number of active VPM fibers. We find that inhibitory neurons in layers 3 and 4 quickly approach maximum spike-timing reliability when receiving input from 10 to 15 synchronously firing VPM neurons. Excitatory neurons in layers 3 and 4 require substantially more synchronous VPM fibers, but less than excitatory neurons in layers 5 and 6 (see Fig. 59). With an average of eight synapses per connection, these numbers are significantly higher than what has been observed in a previous in silico study in the cat visual cortex [3]. In addition to the difference in animal and sensory system, we show that this decrease of reliability can be partly explained by a lower synaptic release probability in vivo than in vitro caused by a lower extracellular calcium concentration in vivo [4], which is taken into account in our simulations [2].

Finally, we describe how the requirement for synchronous, redundant VPM inputs limits the maximum amount of asynchronous, temporally precise VPM activity (in subsets of synchronous VPM neurons) that can be reliably encoded in a neocortical microcircuit.

**Acknowledgements:** This work was supported by funding from the ETH Domain for the Blue Brain Project (BBP). The BlueBrain IV IBM BlueGene/Q system is financed by ETH Board Funding to the Blue Brain Project and hosted at the Swiss National Supercomputing Center (CSCS). We thank M. Bale and R. Petersen for providing the VPM spike trains.

**References**

1. Bale MR, Ince RAA, Santagata G, Petersen RS. Efficient population coding of naturalistic whisker motion in the ventro-posterior medial thalamus based on precise spike timing. Front Neural Circuits. 2015;50.
2. Markram H, et al. Reconstruction and simulation of neocortical microcircuitry. Cell. 2015;163(2):456–92.
3. Wang H, Spencer D, Fellous J, Sejnowski T. Synchrony of thalamocortical inputs maximizes cortical reliability. Science. 2010;328:106–9.
4. Borst JGG. The low synaptic release probability in vivo. Trends Neurosci. 2010;33(6):259–66.

## P100 On the representation of arm reaching movements: a computational model

### Antonio Parziale^1^, Rosa Senatore, Angelo Marcelli^1^

#### Department of Information and Electrical Engineering, University of Salerno, 84084, Fisciano (SA), Italy

##### **Correspondence:** Antonio Parziale - anparziale@unisa.it

*BMC Neuroscience* 2016, **17(Suppl 1)**:P100

Experimental studies on the spinal cord (SC) have shown that SC is not a simple relay station for transmitting information to and from supraspinal centers but “it is a highly evolved and complex part of the CNS that has considerable computational ability” [1]. Limb movements are planned and initiated by the brain but they cannot be performed without a spinal cord and the intricate feedback systems that reside within it [2]. In the last years, computational models have been devised in order to explain the role of the spinal cord in the translation from motor intention to motor execution [3], in sensorimotor control and learning of movements [4], in investigating how the supraspinal centers can control the cord [5], for providing evidence that CNS can plan and control movements without a representation of complex bodily dynamics because the creation and coordination of dynamic muscle forces is entrusted to the spinal feedback mechanisms [6], for investigating how the central nervous system coordinates the activation of both α and γ motoneurons during movement and posture [7].

Here we propose a computational model of the local interneuron networks within SC to evaluate how spinal and supraspinal centers can interact for performing a movement. We model a one-degree of freedom system representing an arm learning and executing reaching movements. The model incorporates the key anatomical and physiological features of the neurons in SC, namely interneurons Ia, Ib and PN and Renshaw cells, and their interconnections [2]. The model envisages descending inputs coming from both rostral and caudal M1 motor cortex and cerebellum (through the rubro- and reticulo-spinal tracts), local inputs from both Golgi tendon organs and spindles, and its output is directed towards α motoneurons, which also receive descending inputs from the cortex and local inputs from spindles. The model envisages virtual muscle [8] for modeling musculoskeletal mechanics and proprioceptors.

Our simulations show that the CNS may produce elbow flexion movements with different properties by adopting different strategies for the recruitment and the modulation of interneurons and motoneurons. One interesting results is that the speed-accuracy tradeoff predicted by the Fitts’ law [9] does not follow from the structure of the system, that is capable of performing fast and precise movements, but arises from the strategy adopted to produce faster movements, by starting from a pre-learned set of motor commands useful to reach the target position and by modifying only the activations of the PN and α neurons.

Other simulations show that when a suddenly variation of the target position happens after the onset of a learned movement, the descending inputs from the cerebellum can be exploited for the online correction of the movement trajectory by regulating the activity of PN cells. This result agrees with the experimental studies suggesting that the CNS modulates interneurons networks to execute a visually guided online correction.

**References**

1. Burke RE. Spinal cord. *Scholarpedia.* 2008;3(4):1925.
2. Pierrot-Deseilligny E, Burke DJ. The circuitry of the human spinal cord: neuroplasticity and corticospinal mechanisms. Cambridge: Cambridge University Press; 2012.
3. Bullock D, Grossberg S. VITE and FLETE: Neural modules for trajectory formation and tension control. Volitional Action. 1989;253–97.
4. Tsianos GA, Goodnes J, Loeb GE. Useful properties of spinal circuits for learning and performing planar reaches. J Neural Eng. 2014;11:1–21.
5. Raphael G, Tsianos GA, Loeb GE. Spinal-like regulator facilitates control of a two-degree-of-freedom wrist. J Neurosci. 2010;30:9431–44.
6. Buhrmann T, Di Paolo EA. Spinal circuits can accommodate interaction torques during multijoint limb movements. Front Comput Neurosci. 2014;8:1–18.
7. Li S, Hao M, He X, Marquez JC, Niu CM, Lan N. Coordinated alpha and gamma control of muscles and spindles in movement and posture. Front Comput Neurosci. 2015;9:1–15.
8. Cheng EJ, Brown IE, Loeb GE: Virtual Muscle: a computational approach to understanding the effects of muscles properties on motor control. J Neurosci Methods. 2000;101:117–30.
9. Fitts PM. The information capacity of the human motor system in controlling the amplitude of movement. J Exp Psychol. 1954;47(6):381–91.

## P101 A computational model for investigating the role of cerebellum in acquisition and retention of motor behavior

### Rosa Senatore^1,2^, Antonio Parziale^1^, Angelo Marcelli^1^

#### ^1^Department of Information and Electrical Engineering and Applied Mathematics, University of Salerno, Fisciano (SA), 81100, Italy; ^2^Laboratory of Neural Computation, Istituto Italiano di Tecnologia, Rovereto (TN), 38068, Italy

##### **Correspondence:** Rosa Senatore - rsenatore@unisa.it

*BMC Neuroscience* 2016, **17(Suppl 1)**:P101

Experimental studies on the cerebellum (CB) have provided a large body of knowledge about its anatomical and physiological features, the neural processes and the phenomena of synaptic plasticity occurring within both the cerebellar cortex and nuclei [1]. The emerging picture is that the CB plays a crucial role in the acquisition and/or retention of motor behaviors and is involved in several cognitive functions [2, 3], therefore several CB models and simulations of its neural processes have been proposed [3–5]. Here we investigated, through a modeling approach, the role of the CB in three different behaviors: vestibulo-ocular reflex (VOR) adaptation, motor learning, and eyeblink conditioning. Different cerebellar areas are involved in these functions: the control of the amplitude and timing of the VOR involves the vestibulocerebellum, learning novel limb movements involves the lateral cerebellar cortex and its connections to the dentate nucleus and acquisition of the eyeblink conditioned responses involves cerebellar cortex areas (lobule HVI) connected to the interposed nucleus[1, 3]. It is noteworthy that the CB is characterized by the remarkable regularity and geometrical structure of its circuits: cerebellar neurons are arranged in a highly regular manner as repeating units, the cerebellar microcomplexes [1]. Therefore the uniform structure of the CB and the contribution of different cerebellar areas to specific behaviors raise the possibility that different behaviors are based on a common ‘neural computation within the cerebellum’.

We developed a model (using the Leabra framework in emergent neural simulation software [6]) that incorporates the key anatomical and physiological features of the cerebellar microcomplex, whose behavior was analyzed for investigating the neural processes occurring during the acquisition of novel motor behaviors, classically conditioned responses and VOR adaptation. Since the neural circuits involved in these behaviors present some differences, in terms of the input/output areas sending signals to or receiving signals from the CB, we developed three models, which share the same core network, made up of a set of cerebellar microcomplexes (comprising cerebellar cortex neurons and their connections to nuclear and olivary neurons), but which include different anatomical connections from/to different extra cerebellar regions: (a) “VOR model”, comprising the vestibulocerebellum (flocculus and vestibular nucleus) and its connections with the dorsal cap region of the inferior olive and oculomotor nuclei; (b) “Motor model”, comprising the lateral cerebellum (lateral cerebellar cortex and dentate nucleus) and its anatomical connections with the inferior olive, thalamus and motor cortex; (c) “Conditioning model”, comprising the lobule HVI of the cerebellar cortex and its connections to the interposed nucleus, and their external connections with the dorsal accessory olive, red nucleus and oculomotor nuclei.

Our simulations suggest that the CB performs the same computational operation on whichever afferent information it receives, that the appearance of the ‘teaching’ signal conveyed by the climbing fibers could be the explanation for functional differentiation and that different types and sites of synaptic plasticity are involved in different behaviors.

References

1. Ghez C, Thach WT. The cerebellum. In: Kandel ER, Schwartz JH, Jessell TM, editors. Principles of neural science. McGraw-Hill; 2000. p. 832–52.
2. Koziol LF, Budding D, Andreasen N, D’Arrigo S, Bulgheroni S, Imamizu H, Ito M, Manto M, Marvel C, Parker K, et al. Consensus paper:the cerebellum’s role in movement and cognition. Cerebellum. 2014;13(1):151–77.
3. Manto M, Bower JM, Conforto AB, Delgado-Garcia JM, da Guarda SN, Gerwig M, Habas C, Hagura N, Ivry RB, Marien P, et al. Consensus paper: roles of the cerebellum in motor control–the diversity of ideas on cerebellar involvement in movement. Cerebellum. 2012;11(2):457–87.
4. Houk JC, Buckingham JT, Barto AG. Models of the cerebellum and motor learning. Behav Brain Sci. 1996;19(3):368–83.
5. Medina JF, Mauk MD. Computer simulation of cerebellar information processing. Nat Neurosci. 2000;3(Suppl.):1205–11.
6. Aisa B, Mingus B, O’Reilly R. The emergent neural modeling system. Neural Netw. 2008;21(8):1146–52.

## P102 The emergence of semantic categories from a large-scale brain network of semantic knowledge

### K. Skiker^1^, M. Maouene^2^

#### ^1^LIST Laboratory, FST, Abdelmalek Essaadi’s University, Tangier, Morocco; ^2^Department of computer science, ENSAT, Abdelmalek Essaadi’s University, Tangier, Morocco

##### **Correspondence:** K. Skiker - skiker.kaoutar85@gmail.com

*BMC Neuroscience* 2016, **17(Suppl 1)**:P102

In cognitive neuroscience, the issue of how semantic categories (e.g. animals, tools, fruits/vegetables) are organized in the brain is still debated (Caramazza and Mahon 2006). Some authors postulate that semantic categories are explicitly represented in specific brain areas developed through evolutionary pressure for rapid classification and categorization of animals, tools and foods (Caramazza and Shelton 1998). Other researches argue that semantic categories are not explicitly represented, instead emerge from distributed semantic knowledge (Martin 2007; Tyler and Moss 2001). However, a little is known about how semantic knowledge is structured within the brain for fast and efficient emergence of semantic categories. In this paper, we hypothesize that semantic knowledge is supported by a large-scale brain network that shows the properties of segregation and integration. To test this hypothesis, we first examine where semantic knowledge is nested in the brain; we present functional neuroimaging studies suggesting that semantic knowledge (e.g. visual, auditory, tactile; action, olfactory/gustatory) is grounded in modality specific association brain areas (e.g. visual association areas, auditory association areas, somatosensory association areas) (Barsalou 2008; Goldberg et al. 2006). Then, we derive the connectivity between brain areas where semantic knowledge is nested from Hagmann’s connectivity matrix (Hagmann et al. 2008) freely available. Finally, we examine the properties of the connectivity matrix using graph measures including clustering coefficient and characteristic path length. Our findings show that a large-scale brain network of features exhibit the small world property with high clustering coefficient (C = 0.48) and low path length (L = 2.49).These properties indicate a balance between segregation (high clustering) and integration (low path length) that are essential for the fast and efficient emergence of semantic categories from distributed semantic knowledge.

**References**

1. Barsalou, Lawrence W. Grounded cognition. Annu Rev Psychol. 2008;59: 617–45.
2. Caramazza A, Mahon BZ. The organisation of conceptual knowledge in the brain: the future’s past and some future directions. Cogn Neuropsychol. 2006;23(1):13–38.
3. Caramazza A, Shelton JR. Domain-specific knowledge systems in the brain the animate-inanimate distinction. J Cogn Neurosci. 1998;10(1):1–34.
4. Goldberg RF, Perfetti CA, Schneider W. Perceptual knowledge retrieval activates sensory brain regions. J Neurosci. 2006;26(18):4917–21.
5. Hagmann P, Cammoun L, Gigandet X, Meuli R, Honey CJ, Wedeen VJ, Sporns O. Mapping the structural core of human cerebral cortex. PLoS Biol. 2008;6(7): e159.
6. Martin A. The representation of object concepts in the brain. Annu Rev Psychol. 2007;58:25–45.
7. Tyler LK, Moss HE. Towards a distributed account of conceptual knowledge. Trends Cogn Sci. 2001;5(6):244–52.

## P103 Multiscale modeling of M1 multitarget pharmacotherapy for dystonia

### Samuel A. Neymotin^1,2^, Salvador Dura-Bernal^1^, Alexandra Seidenstein^1,3^, Peter Lakatos^4^, Terence D Sanger^5,6^, and William W Lytton^1,7^

#### ^1^Department Physiology & Pharmacology, SUNY Downstate, Brooklyn, NY 11203, USA; ^2^Department Neuroscience, Yale University School of Medicine, New Haven, CT, USA; ^3^Departmentt of Chemical and Biomedical Engineering, Tandon School of Engineering, NYU, Brooklyn, NY, USA; ^4^Nathan Kline Institute for Psychiatric Research, Orangeburg, NY, USA; ^5^Department Biomedical Engineering, University of Southern California, Los Angeles, CA, USA; ^6^Div Neurology, Child Neurology and Movement Disorders, Children’s Hospital Los Angeles, LA, CA, USA; ^7^Department Neurology, Kings County Hospital Center, Brooklyn, NY 11203, USA

##### **Correspondence:** Samuel A. Neymotin - samn@neurosim.downstate.edu

*BMC Neuroscience* 2016, **17(Suppl 1)**:P103

Dystonia is a movement disorder that produces involuntary sustained muscle contractions. Different types of dystonia likely involve primary or induced pathologies across multiple brain areas including basal ganglia, thalamus, cerebellum, and sensory and motor cortices. Due to lack of therapeutic alternatives, much current treatment involves paralyzing affected muscles directly with painful injections of botulinum toxin. Primary motor cortex (M1) represents a potential target for therapy. M1 pathological dynamics in some forms of dystonia include hyperexcitability and altered beta oscillations. In order to further develop understanding of motor cortex involvement in this disease and to look at potential drug cocktails (multitarget polypharmacy), we developed a multiscale model of M1 across spatial scales, ranging from molecular interactions, up to cellular and network levels. The model contains 1715 compartmental model neurons with multiple ion channels and intracellular molecular dynamics [1, 2]. Wiring and arrangements of cellular layers of the model was based on previously recorded electrophysiological data obtained from mouse M1 circuit mapping experiments. Simulations were run in the NEURON simulator and intracellular dynamics utilized the reaction–diffusion module [3]. The chemophysiological component of the simulation focused on calcium (Ca) handling, and Ca regulation of hyperpolarization-activated cyclic nucleotide-gated (HCN) channels. The Ca signaling was modeled in conjunction with intracellular cytosolic and endoplasmic reticulum (ER) volumes, inositol triphosphate (IP3) production via a metabotropic glutamate receptor signaling cascade, and ER IP3 and ryanodine receptors (RYR) which release ER Ca into the cytosol. The model reproduced the pathological dynamics providing hyperexcitability and synchronous beta oscillations across cortical layers. We applied independent random variations to multiple ion channel densities (multiple cell membrane channels: HCN, channels for Na, K, Ca; RYR, IP3 channels in ER), to identify pathological and physiological simulation sets. Experiments with these models demonstrated degeneracy, with multiple routes that produced the pathological syndrome. In most cases, there was no single parameter alteration which would induce the change from pathological to physiological dynamics. We used support vector machines to assess the high dimensional parameter space to provide overall direction for passage from an overall pathological to an overall physiological region of parameter space, enabling prediction of multitarget drug cocktails that would be likely to move the system from dystonic to physiological dynamics.

**Acknowledgements:** Research supported by NIH grant R01 MH086638, NIH grant U01 EB017695, NIH grant R01 NS064046, NIH grant R01 DC012947.

**References**

1. Neymotin SA, McDougal RA, Bulanova AS, Zeki M, Lakatos P, Terman D, Hines ML, Lytton WW. Calcium regulation of HCN channels supports persistent activity in a multiscale model of neocortex. Neuroscience. 2016;316:344–66.
2. Neymotin SA, McDougal RA, Sherif MA, Fall CP, Hines ML, Lytton WW. Neuronal calcium wave propagation varies with changes in endoplasmic reticulum parameters: a computer model. Neural Comput. 2015;27:898–924.
3. McDougal RA, Hines ML, Lytton WW. Reaction–diffusion in the NEURON simulator. Front Neuroinform. 2013;7:28.

## P104 Effect of network size on computational capacity

### Salvador Dura-Bernal^1^, Rosemary J. Menzies^2^, Campbell McLauchlan^2^, Sacha J. van Albada^3^, David J. Kedziora^2^, Samuel Neymotin^1^, William W. Lytton^1^, Cliff C. Kerr^2^

#### ^1^Department of Physiology & Pharmacology, SUNY Downstate Medical Center, Brooklyn, NY 11023, USA; ^2^Complex Systems Group, School of Physics, University of Sydney, Sydney, NSW 2006, Australia; ^3^Institute of Neuroscience and Medicine (INM-6), Jülich Research Centre and JARA, Jülich, Germany

##### **Correspondence:** Cliff C. Kerr - cliff@thekerrlab.com

*BMC Neuroscience* 2016, **17(Suppl 1)**:P104

There is exceptionally strong circumstantial evidence that organisms with larger nervous systems are capable of performing more complex computational tasks. Yet relatively few studies have investigated this effect directly, instead typically treating network size as a fixed property of a simulation while exploring the effects of other parameters. Recently, Diehl and Cook [1] found that network performance did increase modestly with network size; however, larger networks also required longer training times to achieve a given performance. In this work, we directly addresses the relationship between network size and computational capacity by using a biomimetic spiking network model of motor cortex to direct a virtual arm towards a target via reinforcement learning [2]. The reaching task was performed by a two-joint virtual arm controlled by four muscles (flexor and extensor muscles for shoulder and elbow joints). These muscles were controlled by a neural model that consisted of excitatory and inhibitory Izhikevich neurons in three cortical populations: a proprioceptive population, which received input from the current arm position; a motor population, which was used to drive the arm muscles; and a sensory population, which served as the link between the proprioceptive and motor populations. The model was trained to reach the target using exploratory movements coupled with reinforcement learning and spike-timing dependent plasticity (STDP). The model was implemented using NEURON.

A major challenge in scaling network size is that not all properties of the network can be held constant. As shown by van Albada et al. [3], while first-order properties (such as average firing rate) can be maintained, there are limitations in preserving second- and higher-order statistical properties (such as noise correlations). Thus, we explored multiple different ways of scaling the connectivity of the network, including (a) preserving connection probability, scaling connection weight to be inversely proportional to model size, and increasing the variance of the external drive; and (b) reducing connection probability to preserve average node degree and leaving other parameters unchanged. In addition, we explored scaling each of the neuronal population groups versus only scaling the sensory (processing) population group. Large differences were observed in network dynamics and statistics based on different scaling choices. However, the relationship between network size and task performance was significant only for certain specific choices of model parameters. Overall, task performance is highly sensitive to the network’s metaparameters, such as STDP learning rates. We found that these must be optimized specifically for different network sizes; otherwise, differences in suitability of these parameters overwhelm the intrinsic advantages of larger networks. In conclusion, while network size does affect computational capacity, the relationship is strongly dependent on the manner in which the scaling is implemented.

**References**

1. Diehl PU, Cook M. Unsupervised learning of digit recognition using spike-timing-dependent plasticity. Front Comp Neurosci. 2015;9:99.
2. Dura-Bernal S, Li K, Neymotin SA, Francis JT, Principe JC, Lytton WW. Restoring behavior via inverse neurocontroller in a lesioned cortical spiking model driving a virtual arm. Front Neurosci. 2016;10:28.
3. Van Albada SJ, Helias M, Diesmann M. Scalability of asynchronous networks is limited by one-to-one mapping between effective connectivity and correlations. PLoS Comput Biol. 2015;11:e1004490.

## P105 NetPyNE: a Python package for NEURON to facilitate development and parallel simulation of biological neuronal networks

### Salvador Dura-Bernal^1^, Benjamin A. Suter^2^, Samuel A. Neymotin^1^, Cliff C. Kerr^3^, Adrian Quintana^4^, Padraig Gleeson^4^, Gordon M. G. Shepherd^2,^ William W. Lytton^1^

#### ^1^Department Physiology & Pharmacology, SUNY Downstate, Brooklyn, NY 11203, USA; ^2^Department Physiology, Northwestern University, Chicago, IL 60611, USA; ^3^Complex Systems Group, School of Physics, University of Sydney, Sydney, NSW 2006, Australia; ^4^Department of Neuroscience, Physiology & Pharmacology, University College London, London WC1E6BT, UK

##### **Correspondence:** Salvador Dura-Bernal - salvadordura@gmail.com

*BMC Neuroscience* 2016, **17(Suppl 1)**:P105

NEURON is a widely used neuronal simulator, with over 1600 published models. It enables multiscale simulation ranging from the molecular to the network level. However, learning to use NEURON, especially running parallel simulations, requires much technical training. NetPyNE (Network development Python package for NEURON) greatly facilitates the development and parallel simulation of biological neuronal networks in NEURON, potentially bringing its benefits to a wider audience, including experimentalists. It is also intended for experienced modelers, providing powerful features to incorporate complex anatomical and physiological data into models.

NetPyNE seamlessly converts a set of high-level specifications into a NEURON model. Specifications are provided in a simple, standardized, declarative format, based solely on Python’s lists and dictionaries. The user can define network populations and their properties, including cell type, number or density. For each cell type, the user can define morphology, biophysics and implementation, or choose to import these from existing files (HOC templates or Python classes). Cell models for each population can be easily changed, and several models can be combined to generate efficient hybrid networks, e.g. composed of Hodgkin–Huxley multicompartment cells and Izhikevich point neurons. NetPyNE provides an extremely flexible format to specify connectivity, with rules based on pre- and post-synaptic cell properties, such as cell type or location. Multiple connectivity functions are available, including all-to-all, probabilistic, convergent or divergent. Additionally, connectivity parameters (e.g. weight, probability or delay) can be specified as a function of pre/post-synaptic spatial properties. This enables implementation of complex biological patterns, such as delays or connection probabilities that depend on distance between cells, or weights that depend on the post-synaptic neuron’s cortical depth. The subcellular distribution of synapses along the dendrites can be specified, and is automatically adapted to the morphology of each model neuron. Learning mechanisms, including spike-timing dependent plasticity and reinforcement learning, can be readily incorporated.

Using the high-level network specifications, NetPyNE instantiates the full model (all cells and connections) as a hierarchical Python structure including the NEURON objects necessary for simulation. Based on a set of simulation options (e.g. duration, integration step), NetPyNE runs the model in parallel using MPI, eliminating the burdensome task of manually distributing the workload and gathering data across computing nodes. Optionally NetPyNE plots output data, such as spike raster plots, LFP power spectra, connectivity matrix, or intrinsic time-varying variables (e.g. voltage) of any subset of cells. To facilitate data sharing, the package saves and loads the high-level specifications, instantiated network, and simulation results using common file formats (Pickle, Matlab, JSON or HDF5). NetPyNE can convert instantiated networks to and from NeuroML, a standard data format for exchanging models in computational neuroscience.

NetPyNE has been used to develop a variety of multiscale models: primary motor cortex with cortical depth-dependent connectivity; the claustrum; and sensorimotor cortex that learns to control a virtual arm. The package is open source, easily installed, and includes comprehensive online documentation, a step-by-step tutorial and example networks (www.neurosimlab.org/netpyne). We believe this tool will strengthen the neuroscience community and encourage collaborations between experimentalists and modelers.

**Acknowledgements:** Research supported by NIH grant U01 EB017695 and DARPA grant N66001-10-C-2008.

## P107 Inter-areal and inter-regional inhomogeneity in co-axial anisotropy of Cortical Point Spread in human visual areas

### Juhyoung Ryu^1^, Sang-Hun Lee^1^

#### ^1^Brain and Cognitive Science, Seoul National University, Seoul 151-742, Republic of Korea

##### **Correspondence:** Juhyoung Ryu - jh67753737@snu.ac.kr, visionsl@snu.ac.kr

*BMC Neuroscience* 2016, **17(Suppl 1)**:P107

A focal visual stimulus can evoke widespread neural activation far beyond the directly stimulated site, a phenomenon referred to as the “cortical point spread” (CPS). The lateral connections among neurons in the early visual cortex have been proposed as a likely anatomical conduit for the CPS, and recent functional studies on humans [1] and non-human primates [2] demonstrated that the CPS is spatially anisotropic, spread preferentially along with the axis of stimulus orientation, dubbed as ‘coaxial anisotropy.’ Although these two seminal studies documented the coaxial anisotropy robustly in two different species, there are several remaining questions to be further explored. First, previous human psychophysical studies reported substantial degrees of inhomogeneity in association field characteristics over the visual space (e.g., crowding effects), which implies the presence of corresponding inhomogeneity of coaxial anisotropy. Second, the animal study [2] examined the coaxial anisotropy only from V1 of two monkeys, and the human study [1] reported a substantial degree of individual differences and inter-areal differences. The current study is set out to address these two aspects of coaxially anisotropic CPS.

**Fig. 60:**
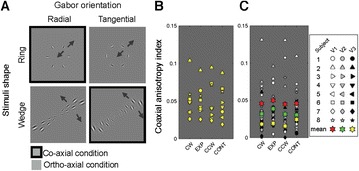
Stimuli and fMRI results. **A** The snapshot of traveling Gabors are shown for the four different conditions. The *black arrows* represent a moving direction of wedge or ring. **B** Significant (*yellow*, t test p < 0.001) coaxial anisotropy in all subjects. **C** Coaxial anisotropy across visual areas (V1, V2, V3)

We acquired time series of functional magnetic resonance imaging (fMRI) measurements in V1 while human individuals viewed a ring or wedge of Gabor patches that slowly drifted along the radial or tangential axis over a spatially extended (up to 8° in radius) region of retinotopic space (Fig. 60A). The orthogonal combination of two different drifting direction and stimulus orientation generated two interesting viewing conditions: coaxial and orthoaxial conditions (boxed and unboxed panels, respectively, in Fig. 60A). For individual gray matter units (2 mm iso volume voxels) in the early visual cortex (V1, V2, V3), we quantified the degree and sign of coaxial anisotropy by comparing the width of fMRI response profiles between the coaxial and orthoaxial conditions. In specific, we first estimated the width of CPS at the half of its maximum response respectively for two viewing conditions − coaxial condition (Wc) and orthoaxial condition (Wo), then computed coaxial anisotropy index by taking the singed contrast between these two width estimates: CAI = (Wc − Wo)/(Wc + Wo).

**Results** The results replicated those in the previous study [1]: in all of the subjects inspected, the width of CPS was significantly greater along the coaxial axis than along the orthoaxial axis (Student’s t test, p < 0.001), and the CAIs ranged from +0.05 to +0.15 (Fig. 60B). In addition, we found two interesting new findings: first, coaxial anisotropy tended to decrease along the processing hierarchy (V1 > V2 > V3; Fig. 60C); second, coaxial anisotropy tended to be more pronounced along the cardinal axes (horizontal meridians in particular) in retinotopic space.

**References**

1. Park SH, Cha K, Lee SH. Coaxial anisotropy of cortical point spread in human visual areas. J Neurosci. 2013; 33(3):1143–56a.
2. Michel MM, Chen Y, Geisler WS, Seidemann E. An illusion predicted by V1 population activity implicates cortical topography in shape perception. Nat Neurosci. 2013; 16(10):1477–83.

## P108 Two bayesian quanta of uncertainty explain the temporal dynamics of cortical activity in the non-sensory areas during bistable perception

### Joonwon Lee^1^, Sang-Hun Lee^1^

#### ^1^Department of Brain and Cognitive Sciences, Seoul National University, Seoul 151-742, Korea

##### **Correspondence:** Joonwon Lee - jwl89@snu.ac.kr; visionsl@snu.ac.kr

*BMC Neuroscience* 2016, **17(Suppl 1)**:P108

**Fig. 61:**
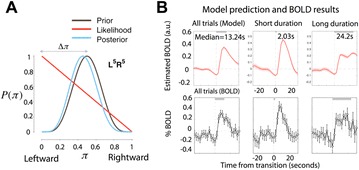
Bayesian estimation to predict BOLD dynamics around switch. **A** Bayesian inference model of iteratively updated prior, input likelihood, and combined posterior. **B** (*Upper*) Uncertainty-driven BOLD estimated from Bayesian model locked to transition under different duration conditions. Time-series is built purely from real behavior history. (*Lower*) Average % BOLD signal of ACC region in 8 subjects (14 sessions)

Bistable perception—involuntary fluctuation over time in perceptual appearance despite unchanging physical stimulation on sensory organs—has been a popular tool for exploring neural loci substantiating transitions in perceptual awareness. To track observers’ ongoing percepts, experimenters make the observers actively report binary states of their perception. Unfortunately, observers’ engagement in perceptual tracking is likely to invite the brain activities that are not associated directly with perceptual transition per se, but rather reflect the cognitive processes ensuing from temporal sequences of perceptual transitions. We reasoned that, in a computational perspective, the latter, post-transition component of bistable perception is majorly driven by two separate quanta of uncertainty: unexpected (UU) and expected uncertainty (EU), which have been proposed to relate to neuromodulatory systems of norepinephrine and acetylcholine, respectively [1]. We found the Dynamic Belief Model (DBM) [2] particularly relevant to the concurrent measurement of those two quanta of uncertainty (i) because it is capable of updating the moment-to-moment, expected probability of binary events based on a recent history of those events (Fig. 61A) and (ii) because this probability directly estimates EU whereas the disparity between the expected probability and perceived outcome quantize UU.

With the DBM in hand, we predicted the time courses of UU and EU (red curves in Fig. 61B), and explored the cortical loci substantiating those two kinds of uncertainty by acquiring fMRI measurements while human observers viewed a ‘structure-from-motion (SfM)’ display, in which ambiguous 2D motion of coherently moving dots gives perceptual alternations in 3D motion perception between bistable states, clockwise vs counterclockwise rotational motion. To compensate for the temporal resolution of fMRI activity, we slowed down the dynamcis of bistable perception using the intermittent stimulation technique, which allowed us to identify gray-matter units (voxels) whose variability in fMRI time course can be explained by UU or EU.

As expected from previous studies, cortical activity increased substantially during the transition periods in many distributed brain regions. More importantly, the fMRI time series in these transition-locked regions were explained by the weighted linear sum of the time series of UU and EU quantity, some exhibiting greater weights for UU and others greater weights for EU. In additions, the time series of pupil size of the observers resembled the predicted time courses of UU, consistent with the previously reported tight linkage between UU and the LC-NE system. We conclude that the cortical activities previously claimed as being responsible for triggering perceptual transition are likely to reflect two post-transition cognitive quanta of uncertainty.

**References**

1. Yu AJ, Dayan P. Uncertainty, neuromodulation, and attention. Neuron. 2005;46:681–92.
2. Yu AJ, Cohen JD. Sequential effects: superstition or rational behavior? NIPS. 2009;21:1873–80.

## P109 Optimal and suboptimal integration of sensory and value information in perceptual decision making

### Hyang Jung Lee^1^, Sang-Hun Lee^1^

#### ^1^Department of Brain and Cognitive Neuroscience, Seoul National University, Gwanak-gu, South Korea

##### **Correspondence:** Hyang Jung Lee - hyangjung.lee@snu.ac.kr, visionsl@snu.ac.kr

*BMC Neuroscience* 2016, **17(Suppl 1)**:P109

Optimization of decision-making often requires the effective integration of sensory and value information, particularly when sensory inputs are ambiguous and the criterion for the successful decision changes stochastically over time (e.g., a hitter making ‘strike’ or ‘ball’ decisions by integrating visual information and an umpire’s calls). We adapted this ‘sensory-value integration’ situation to a laboratory, where 30 human subjects classified ring stimuli into ‘small’ or ‘large’ based on the perceived ring size and the trial-by-trial feedback (‘correct’ or ‘incorrect’) for judgment. The key manipulation was, unbeknownst to subjects, to induce a slight amount of bias, favoring either ‘small’ or ‘large’ choices, or staying ‘unbiased’. Inspired by previous animal studies (Corrado et al. 2005; Lau and Glimcher 2005; Busse et al. 2011), we developed a Linear-Nonlinear-Poisson model to describe the dynamics of how humans adapt their moment-to-moment perceptual decision to subtle, yet volatile environmental feedbacks. The integration process takes place at the Linear stage where a decision variable is formed by combining the sensory information in the current trial and the reward/choice information histories. This is followed by the nonlinear stage where softmax rule is applied to translate the decision variable into probability for the ensuing Poisson stage. Fitting the model to the data set of each individual allowed us to explore the individual differences in optimal sensory-value integration in our task. Our L–N–P model effectively depicted, and generated as well, the temporal dynamics of human subjects’ perceptual choices made in an environment with volatile and stochastic feedbacks. The correlation analysis identified a set of latent model parameters (e.g., reward kernel weight) that are tightly linked to the individual differences in ability to adapt their decision to abrupt changes in feedback. The ideal decision-maker analysis indicated that human subjects are generically suboptimal in forming an effective reward kernel for translating feedbacks in the past trials into action values in the upcoming trials.

**Acknowledgements:** Supported by National Research Foundation of Korea, NRF-2013R1A2A2A03017022.

**References**

1. Busse L, Ayaz A, Dhruv, NT, Katzner S, Saleem, AB, Schölvinck ML, Carandini M. The detection of visual contrast in the behaving mouse. J Neurosci. 2011;31(31):11351–61.
2. Lau B, Glimcher PW. Dynamic response-by-response models of matching behavior in rhesus monkeys. J Exp Anal Behav. 2005;84(3):555–79.
3. Corrado GS, Sugrue LP, Seung, HS, Newsome, WT. Linear–nonlinear–Poisson models of primate choice dynamics. J Exp Anal Behav. 2005;84(3):581.

## P110 A Bayesian algorithm for phoneme Perception and its neural implementation

### Daeseob Lim^1^, Sang-Hun Lee^1^

#### ^1^Department of Brain and Cognitive Sciences, Seoul National University, Seoul, 08826, South Korea

##### **Correspondence:** Daeseob Lim - daeseob@snu.ac.kr, visionsl@snu.ac.kr

*BMC Neuroscience* 2016, **17(Suppl 1)**:P110

Human listeners seem effortless in recognizing a rapid stream of speech sounds uttered by their fellow speakers, thus being capable of readily participating in conversation. However, it remains poorly understood how the brain represents the basic processing unit of such fluent speech perception, phoneme. In a computational perspective, phoneme perception is a reverse engineering of speech production, where the goal is to infer from noisy acoustic signal which phonetic gesture is the one that was most probably intended by a speaker. This ‘probabilistic inferential’ nature of computations makes the Bayesian framework attractive. Here we developed a Bayesian algorithm that captures the two characteristic phenomena of phoneme perception, (1) sharp transitions in perception (categorization) and (2) enhanced discriminability (differentiation) at around phoneme boundaries, and then explored how this algorithm can be implemented in plastic human brains.

Our model posits (i) that the brain has the probabilistic knowledge of frequencies of phonetic stimuli prior to forming the likelihood of phoneme stimuli based on noisy sensory signals (prior and likelihood beliefs), (ii) that the brain combines these two knowledges to form a posterior distribution of probability (posterior belief), and (iii) that the brain ‘optimally’ utilizes this single posterior belief to concurrently perform the categorization and discrimination tasks. The major latent variables of our interest were the shape of the prior probability distribution function (prior PDF) and the width of the likelihood PDF. The parameters for these two PDFs were estimated by fitting the model to the behavioral data in a pair of psychophysical experiments, where human subjects both categorized and discriminated the acoustic stimuli comprising the cyclic transition among three voiced stop consonant–vowel syllables, /ba/-/da/-/ga/, varying in the place of articulation (labial-alveolar-velar). The behaviorally constrained models revealed the prior PDF with the three modes whose peaks correspond to the three prototypical phoneme syllables and the posterior PDF with large variance.

**Fig. 62:**
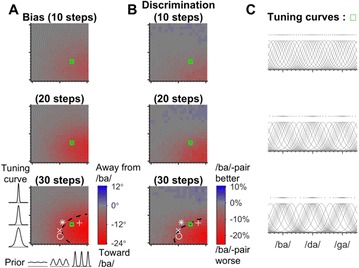
Interaction between tuning curve and prior in population tuning. **A** Bias map for combinations of tuning curve width and prior sharpness. Negative bias means that perception of a near-/ba/stimulus was biased toward/ba/, namely categorical perception. Three *inset* plots on ordinate and abscissa show the cases of the lowest/median/highest concentration parameters of tuning curve and prior peak, respectively. **B** Discrimination difference map. *Negative number* indicates that between-phoneme condition outperformed near-/ba/condition. **C** Tuning curves of population neurons that was marked as green squares in **A**, **B**. Location of tuning centers were marked as dots for 60 neurons, and tuning curves of 30 out of those 60 neurons were drawn below

Having identified the prior and likelihood PDFs used by the optimal Bayesian listener, we explored plausible neural mechanisms for implementing those PDFs. Going beyond previous attempts, our proposal of Bayesian implementation offers a formal account for how the unequal frequency of acoustic stimuli, i.e., stimulus prior, is developmentally translated into an unequal distribution of sensory neurons via well-known canonical principles of neural plasticity (‘neural remapping’). Specifically we propose that sensory neurons, stimulus tuning preferences of which were equally distributed initially, iteratively shift their tuning curves toward an experienced stimulus (‘attractive shift’) as a function of their current responsitivity to that stimulus (Fig. 62). The population distribution of sensory tuning curves that were shaped by this remapping scenario, when plugged into typical probabilistic population coding schemes, reproduced qualitatively the human listeners’ performances in the both phoneme tasks. Our model exercise on tuning width also shed new light on how the optimal tuning width of sensory neurons (broad tuning in our case) can be constrained by the task requirements (categorical perception) and the stimulus environments (biased prior) imposed on a given sensory system (speech perception).

## P111 Complexity of EEG signals is reduced during unconsciousness induced by ketamine and propofol

### Jisung Wang^1^, Heonsoo Lee^1^

#### ^1^Physics department, Pohang University of Science and Technology, Pohang, South Korea

##### **Correspondence:** Heonsoo Lee - beafool@postech.ac.kr

*BMC Neuroscience* 2016, **17(Suppl 1)**:P111

**Fig. 63:**
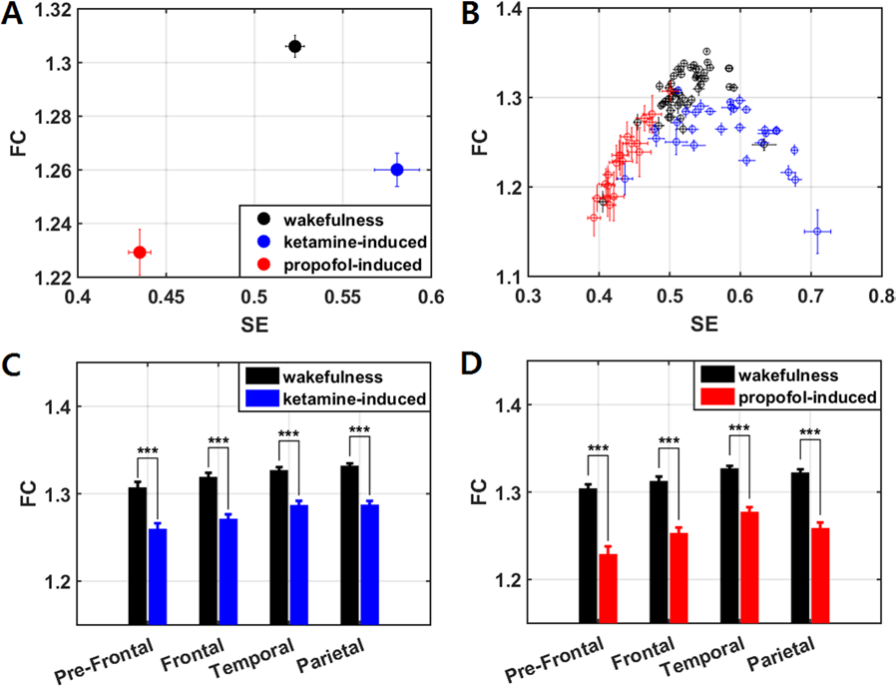
**A** SE and FC values of Fp1 channel for three different states, which are wakeful, ketamine-induced, and propofol-induced states, are averaged over subjects (n = 29 for ketamine-induced, n = 20 for propofol-induced and n = 49 for wakeful states). *Error bars* represent standard errors. Wakeful state has the intermediate SE value between ones of two other states. For FC value, however, wakeful state has the highest one and both anesthetized states have smaller ones, forming a concave relationship between three states. **B** *Each dot* manifests averaged SE and FC values of one subject over 23 10 s-long epochs overlapping 5 s each other. *Error bars* here also indicate standard errors. Most wakeful states have higher FC values compared to ones of unconscious states and have intermediate SE values. Ketamine-induced states are mainly located in the lower right part when propofol-induced states are clustered at the lower left part of the area in SE–FC plot. **C** FC values of EEG signals from the whole brain area, covering pre-frontal, frontal, temporal, and parietal regions, significantly decreased during ketamine-induced loss of consciousness (p < 0.0001). **D** All FC values of the signals from different regions were also significantly reduced during propofol-induced unconscious state (p < 0.0001)

Identifying a universal feature of brain dynamics during anesthetic-induced unconsciousness has been an important work for both practical use of monitoring depth of general anesthesia and scientific knowledge about the nature of consciousness. However, it is difficult because anesthetics with different mechanism of action (MOA) induce distinct brain dynamics. From the perspective of complex system science, we claim that the dynamics generated by conscious brain is more complex compared to one from anesthetic-induced unconscious brain regardless of anesthetic types. To test the hypothesis, we used ketamine and propofol which fall into two distinct anesthetic groups [1]. Disorder and complexity of electroencephalogram (EEG) signals were analyzed before and after bolus injection of drugs. For the analysis, we employed Shannon entropy (SE) and fluctuation complexity (FC), which are information theory-based measures quantifying disorder and complexity, respectively [2]. The study shows that ketamine and propofol both reduced the complexity (p < 0.00001 for both) of EEG signals from the whole brain area (Fp1, F3, T3, P3) while each respectively increased (p = 0.000112) and decreased (p < 0.00001) disorder of the signal (Fig. 63). The finding supports our claim and suggests considering the EEG complexity as a common measure of consciousness.

**References**

1. Lee U, Ku S, Noh G, Baek S, Choi B, Mashour GA. Disruption of frontal–parietal communication by ketamine, propofol, and sevoflurane. J Am Soc Anesthesiol. 2013;118:1245–6.
2. Bates JE, Shepard HK. Measuring complexity using information fluctuation. Phys Lett A. 1993;172:416–25.

## P112 Self-organized criticality of neural avalanche in a neural model on complex networks

### Nam Jung^1^, Le Anh Quang^1^, Seung Eun Maeng^1^, Tae Ho Lee^1^, Jae Woo Lee^1^

#### ^1^Department of Physics, Inha University, Namgu, Incheon 22212, Korea

##### **Correspondence:** Jae Woo Lee - jaewlee@inha.ac.kr

*BMC Neuroscience* 2016, **17(Suppl 1)**:P112

**Fig. 64:**
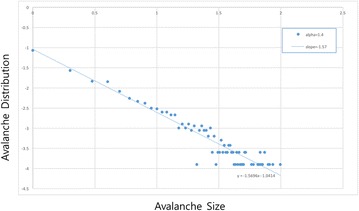
Distribution of avalanche size for LHG model on the fully-connected network. The distribution function of avalanche size shows the power law with exponent −1.57

The concept of the self-organized criticality is applied to many natural and economical systems [1–4]. The distribution of neural avalanche in neural models obeys a power law with exponents of the mean-field theory. Neural avalanches in cultured neocortical network show self-organized criticality over long stable period with exponent −1.5 of the power law for the distribution of the neural avalanche [1]. We consider a modified integrate-and-fire model introduced by Levina, Herrmann and Geisel (LHG model) [2]. We extend the LHG model on the complex networks such as fully-connected network, random network, small-world network, and scale-free networks. In the LHG model the membrane potential of a neuron is accumulated from input potential and random external input. In a fully connected network we observed the power law with exponent −1.57 as shown in Fig. 64. The exponent of the power law depends on the network structure of the neural systems.

**Acknowledgements:** This research was supported by the Basic Science Research Program through the National Research Foundation of Korea (NRF) funded by the Ministry of Science, ICT and Future Planning (NRF-2014R1A2A1A11051982).

**References**

1. Beggs J, Plenz D. Neural avalanche in neocortical circuits. J Neurosci. 2003;23:11167–77.
2. Levina A, Herrmann JM, Geisel T. Dynamical synapses causing self-organized criticality in neural networks. Nat Phys. 2007;3:857–60.
3. Li X, Small M. Neuronal avalanches of a self-organized neural network with active-neuron-dominant structure. Chaos. 2012;22:023104.
4. Liu H, Song Y, Xue F, Li X. Effects of bursting dynamic features on the generation of multi-clustered structure of neural network with symmetric spike-timing-dependent plasticity learning rule. Chaos. 2015;25:113108.

## P113 Dynamic alterations in connection topology of the hippocampal network during ictal-like epileptiform activity in an in vitro rat model

### Chang-hyun Park^1,2^, Sora Ahn^3^, Jangsup Moon^1,2^, Yun Seo Choi^2^, Juhee Kim^2^, Sang Beom Jun^3,4^, Seungjun Lee^3^, Hyang Woon Lee^1,2^

#### ^1^Departments of Neurology, Ewha Womans University School of Medicine, Seoul, Korea; ^2^Department of Medical Science, Ewha Womans University School of Medicine, Seoul, Korea; ^3^Department of Electronics Engineering, Ewha Womans University College of Engineering, Seoul, Korea; ^4^Brain & Cognitive Sciences, Ewha Womans University College of Scranton, Seoul, Korea

##### **Correspondence:** Chang-hyun Park - park.changhyun@gmail.com

*BMC Neuroscience* 2016, **17(Suppl 1)**:P113

**Fig. 65:**
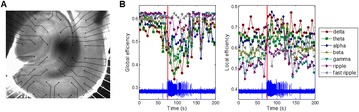
**A** Microelectrodes placed to cover both entorhinal cortex and hippocampus. **B** Temporal changes in global and local efficiency around the time of ictal-like epileptiform activity. The *red vertical line* indicates the initiation of ictal-like events and the time series in *blue* displays recorded field potentials

The experimental approach using in vitro slices of the rat limbic system has been applied to identify mechanisms underlying epileptiform activity in ictal-like events [1]. We prepared combined slices of the rat hippocampus-entorhinal cortex and placed them in artificial corticospinal fluid that contained 4-aminopyridine (4AP). Field potential recordings were made with a microelectrode array composed of 6 × 10 microelectrodes with inter-electrode spacing of 500 μm (see Fig. 65A) when synchronous activity was induced by 4AP. Channels with high artifact rates were rejected, and the signal for each remaining channel was divided into 10 s windows without overlap. For each window, adjacency matrices, or binary networks, were estimated via inter-channel connections based on spectral coherence in different frequency bands, including delta, theta, alpha, beta, gamma, ripple, and fast ripple. Topological properties of the inter-channel networks were assessed by calculating global and local efficiency [2]. As ictal-like events were initiated, global efficiency started to decrease and local efficiency started to increase, and the changes were maintained during ictal-like epileptiform activity (see Fig. 65B). Such changes related to a shift in connection topology to a regularized pattern are in line with the findings for the whole brain in a rat model [3].

**Conclusions** Although the initiation and propagation of epileptiform activity may not be fully appreciated due to the spatially isolated structure in the in vitro slice preparation, the pattern of ictal-like synchronous activity in the limbic system was related to changes in connection topology that may reflect a shift in brain states.

**Acknowledgements:** This research was supported by Basic Science Research Program through the National Research Foundation of Korea (NRF) funded by the Ministry of Science, ICT & Future Planning (2015R1C1A1A01052438 to C. Park and 2014R1A2A1A11052103 to H. W. Lee), and by the Korea Health Technology R&D Project through the Korea Health Industry Development Institute (KHIDI) funded by the Ministry of Health & Welfare (HI14C1989 to H. W. Lee).

**References**

1. Avoli M, Barbarosle M, Lücke A, Nagao T, Lopantsev V, Köhling R: Synchronous GABA-mediated potentials and epileptiform discharges in the rat limbic system in vitro. J Neurosci. 1996;16(12):3912–24.
2. Latora V, Marchiori M. Efficient behavior of small-world networks. Phys Rev Lett. 2001;87(19):198701.
3. Otte WM, Dijkhuizen RM, van Meer MPA, van der Hel WS, Verlinde SAMW, van Nieuwenhuizen O, Viergever MA, Stam CJ, Braun KPJ. Characterization of functional and structural integrity in experimental focal epilepsy: reduced network efficiency coincides with white matter changes. PLoS One. 2012;7(7):e39078.

## P114 Computational model to replicate seizure suppression effect by electrical stimulation

### Sora Ahn^1^, Sumin Jo^1^, Eunji Jun^1^, Suin Yu^1^, Hyang Woon Lee^2^, Sang Beom Jun^1^, Seungjun Lee^1^

#### ^1^Department of Electronics Engineering, Ewha Womans University, Seoul, 120-750, Korea; ^2^Department of Neurology, Ewha Womans University, Seoul, 120-750, Korea

##### **Correspondence:** Seungjun Lee - slee@ewha.ac.kr

*BMC Neuroscience* 2016, **17(Suppl 1)**:P114

Deep brain stimulation (DBS) method for suppression of epileptic seizure is being developed mostly based on clinical experiences because the suppression mechanism by electrical stimulation is still unclear. As such, it is difficult to improve efficacy of the DBS method. The study of computational models allows to predict and analyze the effect of electrical stimulation by computer simulation such that it can help to determine optimum stimulation parameters to suppress seizure activity in various conditions.

In this paper, we propose a hippocampal network model which portrays propagation characteristics of seizure-like events (SLEs) and suppression phenomena by electrical stimulation. The model is composed of four sub-networks representing EC, DG, CA3 and CA1 and well-known synaptic pathways between sub-networks. Each sub-network consists of excitatory and inhibitory neurons which are described by Izhikevich’s model [1]. Besides synaptic transmission [2], electrical field transmission [3] between neurons is also considered. Input gains of neurons are controlled by interaction strengths between sub-networks which are calculated by Granger causality analysis. We adopt the “potassium accumulation hypothesis” in order to replicate suppression effect by electrical stimulation [4, 5]. The effectiveness of the model is confirmed by comparing the simulation results with experimental data which were measured in rat hippocampal slice (horizontal, 400um) in bicuculline bath application. Local field potentials are recorded using micro-electrode array (MEA) and electrical stimulation (130 Hz, 500 µA, biphasic, 3–5 s) is applied manually in EC by an additional depth electrode when SLE is initiated.

**Fig. 66:**
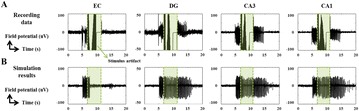
Recording data (**A**) and simulation results (**B**) of SLE suppression effect by electrical stimulation

Following Fig. 66 shows time domain signals recorded in in vitro measurement and generated from the computer model, respectively. After stimulation, the SLE in EC is suppressed immediately, while SLEs in other areas still remained. The simulation results show similar waveforms with experimental data.

**Acknowledgements:** This work was supported by the National Research Foundation of Korea (No. 2014R1A2A1A11052763).

**References**

1. Izhikevich EM. Simple model of spiking neurons. IEEE Trans Neural Netw. 2003;14(6): 1569–72.
2. Izhikevich EM, Gally JA, Edelman GM. Spike-timing dynamics of neuronal groups. Cereb Cortex. 2004;14(8):933–44.
3. Fröhlich F, McCormick DA. Endogenous electric fields may guide neocortical network activity. Neuron. 2010;67(1):129–43.
4. Fertziger AP, Ranck JB. Potassium accumulation in interstitial space during epileptiform seizures. Exp Neurol. 1970;26(3):571–85.
5. Beurrier C, Bioulac B, Audin J, Hammond C. High-frequency stimulation produces a transient blockade of voltage-gated currents in subthalamic neurons. J Neurophysiol. 2001;85(4):1351–56.

## P115 Identifying excitatory and inhibitory synapses in neuronal networks from spike trains using sorted local transfer entropy

### Felix Goetze^1,2^, Pik-Yin Lai^1^

#### ^1^Department of Physics, National Central University, Chung-Li, Taiwan, ROC; ^2^Taiwan International Graduate Program for Molecular Science and Technology, Institute for Atomic and Molecular Sciences, Academia Sinica, Taipei, Taiwan, ROC

##### **Correspondence:** Felix Goetze - afgoetze@gmail.com

*BMC Neuroscience* 2016, **17(Suppl 1)**:P115

Transfer entropy [1] is the established method for quantifying the effective connectivity among neurons.

It has been shown in simulations [2] that measuring it from simultaneously recorded spike trains of neurons can detect the underlying connections and therefore reconstruct a neuronal network from its observed dynamics. Being interpreted as the predicted information transfer, it quantifies the directed non-linear interactions between time series as a model-free method regardless of the underlying interaction type, which could be either inhibitory or excitatory.

Making the distinction between excitatory and inhibitory synapses, however is important in order to understand the underlying principles of spatiotemporal patterns in functional networks.

In our study we describe a method for the measuring of interaction types, based on the concept of local transfer entropies [3]. In contrast to the averaging across all configurations of variables of the source process and the target process as in the Transfer Entropy estimation, the local transfer entropy quantifies the effect of a specific configuration of variables on how they either inform or misinform on the future of the target process. For example observing the presynaptic neuron fire and then observing the postsynaptic neuron fire is informative for an excitatory connection, but misinformative for an inhibitory connection. On average, knowing the past of the source process of an inhibitory or excitatory connection are both predictive of the future of the target process, but local transfer entropies of specific variable configurations have opposite signed values for each interaction type respectively. Sorting the local entropies according to interaction type yields the quantity we call sorted local transfer entropy that aims to identify inhibitory and excitatory synapses from recorded spike trains.

We validate this method with simulated spike trains from the Izhikevich model [4] of cortical neuronal networks, by following a previous paper [2]. The random network is noise-driven and consists of 800 excitatory and 200 inhibitory neurons. Synapses have random delays and the synaptic strengths evolved according to a spike-timing plasticity rule, before the recordings for the analysis are collected. Using Transfer Entropy and the new quantity Sorted Local Transfer Entropy, we reconstruct the networks and distinguish inhibitory from excitatory synapses. The use of two decision boundaries for classifying inhibitory and excitatory synapses separately improves the overall network reconstruction.

**References**

1. Schreiber T. Measuring information transfer. Phys Rev Lett. 2000;85:461.
2. Ito S, Hansen ME, Heiland R, Lumsdaine A, Litke AM, Beggs JM, Zochowski M. Extending transfer entropy improves identification of effective connectivity in a spiking cortical network model. PLoS One. 2011;6:e27431
3. Lizier JT. Measuring the dynamics of information processing on a local scale in time and space. In: Directed information measures in neuroscience. Berlin: Springer; 2014. p. 161–93.
4. Izhikevich EM. Polychronization: computation with spikes. Neural Comput. 2006;18(2):245–82.

## P116 Neural network model for obstacle avoidance based on neuromorphic computational model of boundary vector cell and head direction cell

### Seonghyun Kim^1^, Jeehyun Kwag^1^

#### Department of Brain and Cognitive Engineering, Korea University, Seoul, Korea

##### **Correspondence:** Jeehyun Kwag - jkwag@korea.ac.kr

*BMC Neuroscience* 2016, **17(Suppl 1)**:P116

Developing robots that can perform autonomous exploration in unfamiliar environment is one of the challenges in robotics. Autonomously navigating robots are generally equipped with an obstacle avoidance (OA) system based on sensors such as Light Detection and Ranging (LIDAR) and camera to detect obstacles as well as complex algorithms to correct the noise of sensors [1]. Interestingly, rodent brain shows remarkable reliability to noise in OA [2] through using neurons specialized in processing spatial orientation and spatial boundary called head direction cell (HDC) [3] and boundary vector cell (BVC) [4], respectively. Therefore, building a bioinspired OA system with neural network that consists of neuromorphic HDC and BVC may help increase the efficiency of autonomous navigation. Hence, we built a neural network for OA consisting of all-to-all synaptic connections between six HDCs, four BVCs, and two motor neurons where HDC and BVC were constructed as multi-compartment Hodgkin–Huxley models using the NEURON based on full morphology and electrophysiological properties in vitro [5, 6]. Each HDC was modeled to spike at specific preferred directions separated by 60°, BVCs were modeled to spike at boundaries of cardinal directions and motor neurons were modeled to spike with Gaussian white noise as a background noise. We also built a virtual rat that navigated within a 1 m × 1 m environment whose trajectory was controlled by spikes of motor neurons receiving synaptic inputs from: (1) HDCs, (2) BVCs and (3) both HDCs and BVCs. Number of obstacle detection (detection number: DN) and the time spent during obstacle collision (collision time: CT) were analyzed to compare the efficiency of neural network for OA.

We first verified that our neuromorphic HDC and BVC models could mimic the experimentally recorded electrophysiological properties in vitro and in vivo: HDCs reached maximum firing rate at each preferred direction, and BVCs increased their firing rate as the virtual rat approached boundaries. Using such neuromorphic HDC and BVC models, we investigated the roles of HDC and BVC in neural network for OA. Firstly, we performed the control simulation where virtual rat was controlled with neural network without neither HDC nor BVC and observed that DN was 40 and CT was 137 s. When HDC was added to the neural network, the result was similar to control simulation, (DN = 38 and CT = 139 s), indicating HDC alone cannot perform OA efficiently. When BVC alone was included in the neural network, DN substantially increased and CT decreased compared to control model (DN = 110 and CT = 73 s), indicating that OA efficiency increased. Finally, when both HDC and BVC were included in the neural network, the OA performance was most efficient (DN = 139 and CT = 39 s). These results suggest that our neural network model composed of neuromorphic HDC and BVC neurons can successfully perform OA even with background noise. Therefore, here we suggest the bioinspired neural network that consists of neuromorphic computational model of HDC and BVC could serve as a new approach to build an efficient OA system.

**Acknowledgements:** This study was supported by the Basic Science Research Program through the National Research Foundation of Korea funded by the Ministry of Science, ICT and Future Planning (NRF-2013R1A1A2053280).

**References**

1. Zohaib M, Pasha M, Riaz R, Javaid N, Ilahi M, Khan R. Control strategies for mobile robot with obstacle avoidance. J Basic Appl Sci Res. 2013;3(4):1027–36.
2. Vorhees CV, Williams MT. Assessing spatial learning and memory in rodents. ILAR J. 2014;55(2):310–32.
3. Taube JS, Muller RU, Ranck JB Jr. Head-direction cells recorded from the postsubiculum in freely moving rats. I. Description and quantitative analysis. J Neurosci. 1990;10(2):420–35.
4. Lever C, Burton S, Jeewajee A, O’Keefe J, Burgess N: Boundary vector cells in the subiculum of the hippocampal formation. J Neurosci. 2009;29(31):9771–77.
5. Yoder RM, Taube JS. Projections to the anterodorsal thalamus and lateral mammillary nuclei arise from different cell populations within the postsubiculum: implications for the control of head direction cells. Hippocampus. 2011;21(10):1062–73.
6. Menendez de la Prida L, Suarez F, Pozo MA. Electrophysiological and morphological diversity of neurons from the rat subicular complex in vitro. Hippocampus. 2003;13(6):728–44.

## P117 Dynamic gating of spike pattern propagation by Hebbian and anti-Hebbian spike timing-dependent plasticity in excitatory feedforward network model

### Hyun Jae Jang^1^, Jeehyun Kwag^1^

#### Department of Brain and Cognitive Engineering, Korea University, Seoul, Korea

##### **Correspondence:** Jeehyun Kwag - jkwag@korea.ac.kr

*BMC Neuroscience* 2016, **17(Suppl 1)**:P117

Precise timings of spikes within in vivo spike train are believed to carry information critical for neural computation [1]. For such neural information to be effective, temporal patterns of spike train should be able to propagate across multiple neuronal layers in the feedforward network (FFN) of the brain without dissipation [2]. To support such reliable propagation of spike patterns, preferential and selective strengthening of synaptic pathways through which the spike patterns are routed may be necessary. Asymmetric Hebbian and anti-Hebbian spike timing-dependent plasticity (STDP), where synaptic strengths are strengthened or weakened depending on the precise relative timing and the order between pre- and postsynaptic spikes [3, 4], may serve as a good candidate for dynamically routing the propagation of spike patterns.

Hence, we investigated the role of Hebbian and anti-Hebbian STDP in spike pattern propagation using a six-layered FFN model composed of 200 Hodgkin–Huxley excitatory neurons in each layer. Asymmetric and symmetric/Hebbian and anti-Hebbian STDP were modeled at excitatory synapses using exponential functions [5]. In vivo spike train obtained from public database (crcns.org) was used as an input spike pattern (T_IN_) in layer 1, which was simulated in a small subset of excitatory neurons in layer 1 of FFN model, while the rest were made to spontaneously spike with spike frequencies showing log-normal distribution to mimic in vivo background noise. The propagation of temporal spike pattern was quantified by analyzing the similarity ratio (SR) between T_IN_ and output spike pattern in layer 6 (T_OUT_), which calculates how instantaneous inter-spike intervals of T_IN_ and T_OUT_ are similar.

In FFN model without STDP, the spike pattern of T_IN_ in layer 1 became dissipated in noise as it propagated across layers, and consequently failed to preserve its spike pattern to layer 6 with low SR (0.49). When asymmetric anti-Hebbian STDP was included in FFN model, T_IN_ also failed to propagate to layer 6 with low SR (0.17). However, in the presence of asymmetric Hebbian STDP, T_IN_ successfully propagated to the final layer with high SR (0.87), indicating that asymmetric Hebbian STDP preferentially enhanced T_IN_ propagation in FFN model. Further analysis revealed that asymmetric Hebbian STDP selectively strengthened the synaptic weights of the synaptic pathways routing T_IN_ while it weakened the synaptic weights of that routing noise, effectively serving as an open-gate for propagating T_IN_. In contrast, asymmetric anti-Hebbian STDP curve selectively weakened the synaptic weights of the synaptic pathways routing T_IN_, serving as a close-gate for propagating T_IN_. We also tested the effect of symmetric Hebbian STDP which induces only LTP or symmetric anti-Hebbian STDP which induces only LTD, and found that both types of symmetric STDP failed to propagate T_IN_ with low SR (symmetric Hebbian = 0.23, symmetric anti-Hebbian = 0.14). Our results demonstrate that only asymmetric Hebbian STDP facilitates the reliable propagation of in vivo temporal pattern while asymmetric and symmetric anti-Hebbian STDP blocks temporal pattern propagation, suggesting that different types of STDP may dynamically gate the propagation of neural information.

**Acknowledgements:** This study was supported by Human Frontier Science Program (RGY0073/2015) and the Basic Science Research Program through the National Research Foundation of Korea funded by the Ministry of Science, ICT and Future Planning (NRF-2013R1A1A2053280).

**References**

1. Mainen ZF, Sejnowski TJ. Reliability of spike timing in neocortical neurons. Science. 1995;268(5216):1503–6.
2. Kumar A, Rotter S, Aertsen A. Spiking activity propagation in neuronal networks: reconciling different perspectives on neural coding. Nat Rev Neurosci. 2010;11(9):615–27.
3. Bi GQ, Poo MM. Synaptic modifications in cultured hippocampal neurons: dependence on spike timing, synaptic strength, and postsynaptic cell type. J Neurosci. 1998;18(24):10464–72.
4. Feldman DE. The spike-timing dependence of plasticity. Neuron. 2012;75(4):556–71.
5. Song S, Miller KD, Abbott LF. Competitive Hebbian learning through spike-timing-dependent synaptic plasticity. Nat Neurosci. 2000;3(9):919–26.

## P118 Inferring characteristics of input correlations of cells exhibiting up-down state transitions in the rat striatum

### Marko Filipović^1,2^, Ramon Reig^3^, Ad Aertsen^1,2^, Gilad Silberberg^4^, Arvind Kumar^1,5^

#### ^1^Bernstein Center Freiburg, Freiburg, Germany; ^2^Faculty of Biology, University of Freiburg, Freiburg, 79104, Germany; ^3^Instituto de Neurociencias de Alicante, University of Alicante, Alicante, Spain; ^4^Department of Neuroscience, Karolinska Institute, Stockholm, 17177, Sweden; ^5^Department of Computational Science and Technology, School of Computer Science and Communication, KTH Royal Institute of Technology, Stockholm, 10040, Sweden

##### **Correspondence:** Marko Filipović - marko.filipovic@bcf.uni-freiburg

*BMC Neuroscience* 2016, **17(Suppl 1)**:P118

Rat striatal projection neurons (SPNs) recorded under ketamine anesthesia exhibit slow oscillations, with transitions between depolarized and hyperpolarized membrane potential also referred to as up and down states, respectively. It is presumed that the activity during hyperpolarized down-states is determined by intracellular processes, whereas the large membrane voltage fluctuations during up-states are a product of increased synaptic input. Because local striatal activity during an up-state is weak, the statistics of the up-state fluctuations mainly reflect cortical feedforward input to the SPNs.

To infer the statistics of the cortical input to SPNs we measured the statistics and spectrum of the membrane potential of SPNs in up and down states. The spectrum of the membrane potential reflects the filtering properties of the membrane and can be used to estimate the effective time constant (τ_eff_) of the neuron. Our analysis showed that SPNs have significantly smaller τ_eff_ in the up-state than in the down-state, consistent with the assumption that the barrage of synaptic input causes an increase in membrane conductance during the up state. However, this observation is inconsistent with the idea that depolarization of SPNs should increase the membrane time constant because of the closing of some of the voltage dependent ion channels (e.g. the Kir) channels [1].

The mean (μ_up_) and variance (σ_up_) of the membrane potential during up states varied in a correlated manner. At the same time, for a given SPN, μ_up_ and σ_up_ of individual up-state membrane potentials were highly variable across different up states, indicating a corresponding variability in the cortical inputs. Using a point neuron model of an SPN, we show that the correlation and variability of the up-state mean and variance could be explained if we assume that SPNs receive correlated inputs.

Across different SPNs, each recorded in a different animal, we observed a high variability in the correlation (*ρ*) between μ_up_ and σ_up_. This variability could arise from the heterogeneity in the neuron morphology, intracellular properties, conductance state of the neurons, synaptic weights and the input rate and correlations. Using a point neuron model we tested the dependence of *ρ* on each of these properties. Our analysis showed that the variability of the correlation between μ_up_ and σ_up_ arises because of the diversity of synaptic weights and input correlations, and not because of intrinsic properties of SPNs. This suggests that neuronal heterogeneity could be obscured by the statistics of the synaptic inputs and synaptic weights.

In summary, our analysis of up-down states allows us to make general inferences about characteristics of correlated synaptic input, such as strength of correlations and input firing rate, solely based on membrane potential recordings of SPNs exhibiting up and down states.

**Acknowledgements:** This work was supported in parts by the Erasmus Mundus Joint Doctorate Programme EUROSPIN (MF), an ERC starting grant (GS), the Knut and Alice Wallenberg Academy Fellowship, the Karolinska Institutet Strategic Research program in Neuroscience (StratNeuro; GS, AK), and the Swedish Medical Research Council (GS).

**Reference**

1. Nisenbaum ES, Wilson CJ. Potassium currents responsible for inward and outward rectification in rat neostriatal spiny projection neurons. J Neurosci. 1995, 15: 4449–63.

## P119 Graph properties of the functional connected brain under the influence of Alzheimer’s disease

### Claudia Bachmann^1^, Simone Buttler^1^, Heidi Jacobs^2,3,4^, Kim Dillen^5^, Gereon R Fink^5,6^, Juraj Kukolja^5,6^, Abigail Morrison^1,7,8^

#### ^1^Institute of Neuroscience and Medicine (INM-6) and Institute for Advanced Simulation (IAS-6) and JARA BRAIN Institute I, Jülich Research Centre, Jülich, Germany; ^2^Faculty of Health, Medicine and Life Science, School for Mental Health and Neuroscience (MHeNS), Alzheimer Centre Limburg, Maastricht University Medical Centre, PO Box 616, 6200 MD Maastricht, The Netherlands; ^3^Department of Radiology & Athinoula A. Martinos Center for Biomedical Imaging, Massachusetts General Hospital, Harvard Medical School, Boston, MA 02114, USA; ^4^Faculty of Psychology and Neuroscience, Department of Cognitive Neuroscience, Maastricht University, PO BOX 616, 6200 MD Maastricht, The Netherlands; ^5^Cognitive Neuroscience, Institute of Neuroscience and Medicine (INM-3), Jülich Research Centre, Jülich, Germany; ^6^Department of Neurology, University Hospital of Cologne, Cologne, Germany; ^7^Computational Neuroscience, Bernstein Center Freiburg, Freiburg, 79104, Germany; ^8^Institute of Cognitive Neuroscience, Faculty of Psychology, Ruhr-University Bochum, 44801 Bochum, Germany

##### **Correspondence:** Claudia Bachmann - c.bachmann@fz-juelich.de

*BMC Neuroscience* 2016, **17(Suppl 1)**:P119

Diagnosing Alzheimer’s disease (AD), especially in the early stage, is costly and burdensome for the patients, since it comprises a battery of psychological tests and an extraction of disease specific biomarkers from the cerebrospinal fluid. A cheaper and more convenient procedure would be a diagnosis based on images obtained through fMRI. Based on previous polymodal studies demonstrating disrupted inter- and intra-cortical connectivity in AD [1], we argue that the functional connectivity of the whole cortex might be a good predictor for the cause of the disease. In resting state fMRI, previous attempts to analyze graph properties of whole brain networks contradict each other [2]. In our opinion there are two general critical points in the methodology of these studies that are likely to contribute to the variability of the results. First, we criticize that the activities of the brain areas (graph nodes) that are used to calculate the functional connectivities (weights of the graph edges) are composed of functionally inhomogeneous signals, as individual brains are often mapped onto a standard atlas brain of known functional coherent areas [2, 3]. The second problem consists in converting the resulting weighted graphs into simple graphs, by setting weights above an arbitrary threshold w_min_ to 1, and those below it to 0 [2]. The drawback here is that there is no validation for an optimal threshold, and information that might be relevant in AD may be lost. In this work we address the first problem by applying an activity-driven, region-growing clustering algorithm derived from image processing [4]. In order to guarantee functionally homogeneous clusters, the threshold for inclusion of a voxel in a region is regulated by a heterogeneity criterion [3]. Applying this algorithm, we end up with undirected weighted graphs with varying numbers of nodes for three sets of data: healthy elderly controls, mild cognitive impairment and Alzheimer’s disease. Targeting the second problem, we analyze the dependence of graph theoretic measures (shortest path length, in- and out-degree distribution, clustering coefficient, modularity and minimal spanning tree [5]) on w_min_. Finally, we investigate the distribution of these measures for each data set to determine candidates for a predictive measure.

**Acknowledgements:** We acknowledge partial support by the Helmholtz Alliance through the Initiative and Networking Fund of the Helmholtz Association and the Helmholtz Portfolio theme “Supercomputing and Modeling for the Human Brain”.

**References**

1. Bokde AL, Ewers M, Hampel H.: Assessing neuronal networks: understanding Alzheimer’s disease. Prog Neurobiol. 2009;89:125–33.
2. Tijms BM, Wink AM, de Haan W, van der Flier WM, Stam CJ, Scheltens P, Barkhof F. Neurobiol: Alzheimer’s disease: connecting findings from graph theoretical studies of brain networks. Aging. 2013;34: 2023–36.
3. Marrelec G, Fransson P. Assessing the influence of different ROI selection strategies on functional connectivity analyses of fMRI data acquired during steady-state conditions. PLoS One. 2011;6(4):e14788.
4. Lu Y, Jiang T, Zang Y. Region growing method for the analysis of functional MRI data. *Neuroimage.* 2003;20(1):455–65.
5. Wang J, Zuo X, Dai Z, Xia M, Zhao Z, Zhao X, Jia J, Han Y, He Y. Disrupted functional brain connectome in individuals at risk for Alzheimer’s disease. Biol Psychiatry. 2013;73(5):472–81.

## P120 Learning sparse representations in the olfactory bulb

### Daniel Kepple^1^, Hamza Giaffar^1^, Dima Rinberg^2^, Steven Shea^1^, Alex Koulakov^1^

#### ^1^Cold Spring Harbor Laboratory, Cold Spring Harbor, NY 11724, USA; ^2^NYU Neuroscience Institute, New York, NY 10016, USA

##### **Correspondence:** Daniel Kepple - akula@cshl.edu

*BMC Neuroscience* 2016, **17(Suppl 1)**:P120

**Fig. 67:**
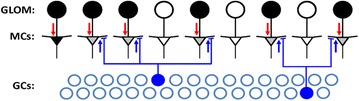
Sparse incomplete representations (SIR). In our previously formulated model of the main olfactory bulb network [1], MCs receive inputs from receptor neurons in the glomeruli (*black circles*) and interact with GCs through dendrodendritic synapses. GCs build representations of MC glomerular inputs (*red arrows*). The representations are contained in the inhibitory inputs returned by the GCs to the MCs (*blue arrows*). Because GCs inhibit each other through second-order inhibitory interactions, only a few GCs respond to an odorant (full *blue circles* with a dendrite shown). The vast majority of GCs do not change their firing rate in response to an odorant (*empty circles*). Thus, the responses of GCs are sparse. Because some MCs manage to retain the responses to odorants, the representation by GCs is called *incomplete*. According to this model, MCs transmit to higher areas the errors in the GC representation

In mouse olfaction, olfactory receptor responses are aggregated in spherical structures of the main olfactory bulb (MOB) called ‘glomeruli.’ These signals are then received by mitral cells (MC) and communicated to the cortex. MC responses are modified by local inhibitory interneurons called granule cells GC, which vastly outnumbered MCs (50fold–100fold). In our previous work [1], we proposed a model in which GCs inhibit MC to form a sparse, incomplete representation (SIR) of odors (Fig. 67). Here, we reason that since sparse representations are efficient, sparseness may increase with learning. We extend the SIR model to allow network synaptic weights to be adjusted, increasing representational sparseness and increasing stimulus discriminability. We derive learning rules for dendrodendritic connectivity between GCs and MCs and also for centrifugal cortico-granule synapses. We computationally test these learning rules and make several predictions of GC and MC plasticity. Specifically, we predict that a minority of GCs outcompete the rest of the population to generate a negative image of a learned odor. Additionally, we predict that participation of the GC network will confer combination selectivity and the ability to discriminate overlapping input patterns. Finally, we experimentally validate these predictions for the dynamics of GCs during locus coeruleus-induced MOB plasticity.

**Reference**

1. Koulakov AA, Rinberg D. Sparse incomplete representations: a potential role of olfactory granule cells. Neuron. 2011;72(1):124–36

## P121 Functional classification of homologous basal-ganglia networks

### Jyotika Bahuguna^1,2,3^, Tom Tetzlaff^1^, Abigail Morrison^1,2^, Arvind Kumar^2,3^, Jeanette Hellgren Kotaleski^3^

#### ^1^Institute of Neuroscience and Medicine (INM-6), Institute for Advanced Simulation (IAS-6) and JARA BRAIN Institute I, Jülich Research Centre, Jülich, Germany; ^2^Computational Neuroscience, Bernstein Center Freiburg, Freiburg, 79104, Germany; ^3^Computational Brain Science, Department of Computational Science and Technology, School of Computer Science and Communication, KTH, Royal Institute of Technology, Stockholm, Sweden

##### **Correspondence:** Jyotika Bahuguna - j.bahuguna@fz-juelich.de

*BMC Neuroscience* 2016, **17(Suppl 1)**:P121

The basal ganglia (BG) are a set of nuclei that play an important role in motor and cognitive functions. Indeed many brain diseases such as Parkinson’s disease (PD) can be attributed to dysfunction of one or more BG nuclei. The classical model of basal ganglia has been regularly updated with discoveries of new sub-populations within a nucleus or new projections from existing nuclei in recent years. It is unclear how these new insights on the structure of the BG network foster our understanding of its function. The effective connectivities among these recently identified BG sub-populations are only partially known. In the framework of a simple firing-rate model subjected to a genetic algorithm, we identified effective BG connectivities which are consistent with experimentally established firing-rate and phase relationships in Subthalamic Nucleus (STN) and two GPe subpopulations (arkypallidal [GPe-TA] and prototypical [GPe -TI]) in both healthy and PD states [1]. This is in extension to an earlier model that identified effective connectivities for the STN-TA-TI-sub circuit [2].

As expected, we found that multiple parameter combinations can fit the data [1]. We re-classified these homologous networks that reproduced the healthy and PD state, on the basis of two dynamical features: suppression of GPi activity and susceptibility of the BG network to oscillate in the presence of cortical input. These features were chosen because task execution requires GPi suppression while oscillations in the STN-GPe subnetwork are characteristic of PD. We found that most putative pathological networks showed insufficient suppression of GPi activity and high susceptibility to oscillations whereas most putative healthy networks showed sufficient suppression of GPi activity and low susceptibility to oscillations. This is consistent with experimental data that shows that lack of GPi suppression [3] or oscillations [4, 5] is correlated with Parkinsonian symptoms such as stymied movement and tremor. A small fraction of networks, however, in both cases show deficiency in only one of the features. This could indicate the configurations of healthy networks that might be more pathology prone and in contrast configurations of pathological networks that might be easier to push into a healthy state. Further analysis of estimated BG connectivity revealed that transitions between the putative PD and healthy networks were possible by modifying the strength of the relevant projections. Most of the transitions involved changes in corticostriatal, striatopallidal and pallidopallidal projections. Finally, the variance observed in the functional classification of putative pathological and healthy networks might hint at the variance observed in manifestation of Parkinson’s disease (PD).

**Acknowledgements:** Klinische Forschergruppe (KFO219, TP12) of the Deutsche Forschungsgemeinschaft; Helmholtz Association, EuroSPIN and Erasmus Mundus Joint Doctorate Programme.

**References**

1. Mallet A, Pogosyan A, Márton LF, Bolam JP, Brown P, Magill PJ. Parkinsonian beta oscillations in the external globus pallidus and their relationship with subthalamic nucleus activity. J Neurosci. 2008;28(52):14245–58.
2. Nevado-Holgado AJ, Mallet N, Magill PJ, Bogacz R. Effective connectivity of the subthalamic nucleus-globus pallidus network during Parkinsonian oscillations. J. Physiol. 2014;592(7):1429–55.
3. Boraud T, Bezard E, Bioulac B, Gross CE. Ratio of inhibited-to-activated pallidal neurons decreases dramatically during passive limb movement in the MPTP-treated monkey. J Physiol. 2000;83(3):1760–63.
4. Chen CC, Litvak V, Gilbertson T, Kühn A, Lu CS, Lee ST, Tsai CH, Tisch S, Limousin P, Hariz M, Brown P. Excessive synchronization of basal ganglia neurons at 20 Hz slows movement in Parkinson’s disease. Exp Neurol. 2007;205(1):214–21.
5. Moran A, Bergman H, Israel Z, Bar-Gad I. Subthalamic nucleus functional organization revealed by parkinsonian neuronal oscillations and synchrony. Brain. 2008;131(Pt-12):3395–409.

## P122 Short term memory based on multistability

### Tim Kunze^1,2^, Andre Peterson^3^, Thomas Knösche^1^

#### ^1^Max Planck Institute for Human Cognitive and Brain Sciences, Leipzig, Germany; ^2^Institute of Biomedical Engineering and Informatics, Ilmenau University of Technology, Ilmenau, Germany; ^3^Department of Medicine, University of Melbourne, Melbourne, Australia

##### **Correspondence:** Tim Kunze - tkunze@cbs.mpg.de

*BMC Neuroscience* 2016, **17(Suppl 1)**:P122

Neural circuits can be formally described by modeling the collective behavior of relatively homogeneous neural populations, so-called neural masses [1]. Some neural mass models consider the minimum set of one excitatory and one inhibitory subpopulation each. In contrast, three-population models make a distinction between excitatory pyramidal cells (PC), projecting to distant areas, and excitatory interneurons (EIN), providing local feedback. We investigate a three-population neural mass model, driven by input to the EIN, with respect to its input/output behavior. We find that such a circuit exhibits, for sufficiently salient inputs, a memory effect based on multi-stability. Furthermore, we test the hypothesis that this mechanism essentially depends on the separation between input and output neurons and is thus not captured in the simpler two-population model.

We use a neural mass model [2], where a pyramidal cell subpopulation receives negative feedback from an inhibitory interneuron subpopulation and positive feedback either directly through self-connections or indirectly via a secondary excitatory subpopulation of interneurons. The respective feedback topology of interest (including which subpopulation is targeted by external input) is controlled by a single parameter. We systematically applied transient sensory inputs, modeled by pulses of various magnitude and duration, as external inputs to the EIN and monitored the behavior of the PC.

**Fig. 68:**
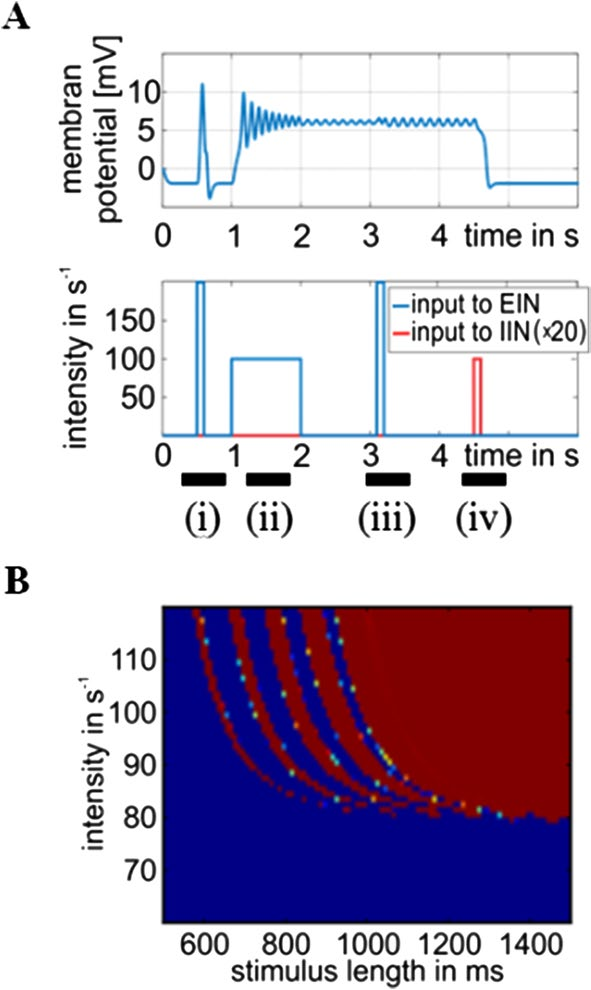
**A** Response of pyramidal cells to transient input to excitatory interneurons shows different modes. **B** Depending on intensity and duration of the stimulus

Depending on the duration and intensity of the applied stimuli (see Fig. 68A), the output either transiently follows the input (i) or it jumps to a more depolarized state, where it remains oscillating with a higher mean membrane potential even after the stimulus has ceased (ii) and where further input does not effect the output any more (iii). This state can be terminated by an impulse to the inhibitory interneurons (iv). The accessibility of this memory effect depends on the saliency of the stimulus in terms of duration and intensity (see Fig. 68B) and disappears in case of direct feedback in a structurally similar two population model.

The identified short-term memory mechanism would be important for temporal integration in cortical processing, potentially applicable in predictive coding schemes. The distinction between the input receiving excitatory subpopulation and the output sending excitatory subpopulation appears to be crucial for the described mechanism, which is further modulated by inhibitory feedback. The further examination of the ratio between excitation and inhibition, governing this mechanism, thus represents an important step to elucidate how the topology between excitatory and inhibitory neural populations affects emerging dynamics on a mesoscopic scale with potential effects on brain states and higher-order brain functionality.

**References**

1. Freeman WJ. Mass action in the nervous system. New York: Academic Press; 1975.
2. Spiegler A, Kiebel SJ, Atay FM, Knösche TR. Bifurcation analysis of neural mass models: Impact of extrinsic inputs and dendritic time constants. NeuroImage. 2010;52:1041–58.

## P123 A physiologically plausible, computationally efficient model and simulation software for mammalian motor units

### Minjung Kim^1^, Hojeong Kim^1^

#### ^1^Division of IoT and Robotics Convergence Research, DGIST, Daegu, 42988, Korea

##### **Correspondence:** Hojeong Kim - hojeong.kim03@gmail.com

*BMC Neuroscience* 2016, **17(Suppl 1)**:P123

**Background** A spinal motoneuron contacts a bunch of muscle fibers forming a motor unit that underlies all mammalian movements. The essential role of the motor unit is the transduction of synaptic inputs from descending and reflex pathways into muscle force. Since the input–output properties of both motoneurons and muscle fibers are non-linear, it has been difficult to make predictions on how changes in synaptic inputs to motoneuron, cellular properties of the motoneuron and muscle fibers and muscle length may affect motor output [1].

**Methods** To tackle this fundamental issue in the field of motor neuroscience, we developed a physiologically plausible but computationally efficient model of the motor unit and a software package that allows for virtual experiments on the input–output properties of the motor unit over a full range of physiological inputs and biophysical parameters.

**Results** The computational model of motor unit was first built in this study coupling the motoneuron model and the muscle unit model with a simplified axon model. The motoneuron model was developed using the recently reported two-compartment modeling approach [2]. The key feature of the new reduced motoneuron model is that all cable parameters of the reduced model are analytically determined based on the system properties such as input resistance, membrane time constant and electrical coupling properties between the soma and the dendrites, which are all empirically measurable from real motoneurons.

For the muscle unit, the recently developed muscle modeling approach was employed that consists of three sub-modules representing [3]: (1) the transformation of the spike signals from motoneurons into the dynamics of calcium concentration in the sarcoplasm, (2) the conversion of the calcium concentration to the muscle activation level, and (3) the transformation of the muscle activation level into the muscle force using Hill-type muscle mechanics. The new muscle model was constructed in this study to reflect all experimentally identified dependencies of muscle activation dynamics on muscle length and movement over a full range of stimulation frequencies in cat soleus muscles.

Then, to enhance the usability and extendibility the software package for simulating and analyzing the developed motor unit model was designed and implemented based on the object-oriented programing paradigm and open source Python language along with graphic user interfaces (GUI). The software package developed in this study provides a GUI-based simulation environment in which a single motoneuron, muscle unit, and motor unit can be individually simulated and analyzed in a wide range of experimental conditions reflecting biological realisms.

**Conclusions** Our model of the motor unit and user-friendly simulation software may provide not only a computational framework to gain systemic insights into motor control by the central nervous system in a cellular perspective but also a basis on which to build biologically realistic large-scale neuro-musculo-skeletal models.

**Acknowledgements:** This work was supported by the DGIST R&D Program of the Ministry of Science, ICT and Future Planning of Korea (15-RS-02 and 16-RS-02).

**References**

1. Heckman CJ, Enoka RM: Motor unit. Compr Physiol. 2012;2(4):2629–82.
2. Kim H, Jones KE, Heckman CJ. Asymmetry in signal propagation between the soma and dendrites plays a key role in determining dendritic excitability in motoneurons. PLoS One. 2014;9(8):e95454.
3. Kim H, Sandercock TG, Heckman CJ. An action potential-driven model of soleus muscle activation dynamics for locomotor-like movements. J Neural Eng. 2015;12(4).

## P125 Decoding laser-induced somatosensory information from EEG

### Ji Sung Park^1^, Ji Won Yeon, Sung-Phil Kim^1^

#### ^1^Department of Human Factors Engineering, Ulsan National Institute of Science and Technology, Ulsan 689-798, South Korea

##### **Correspondence:** Sung-Phil Kim - spkim@unist.ac.kr

*BMC Neuroscience* 2016, **17(Suppl 1)**:P125

**Fig. 69:**
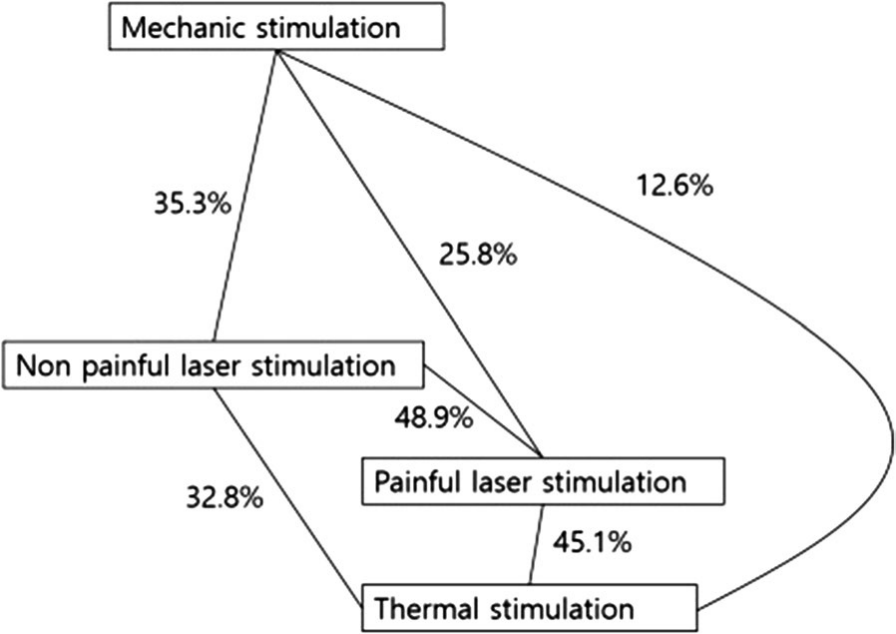
*Each node* represents stimulation type and each edge means classification error rate. Length of edge shows similarity between a pair of stimulation

Recently, our research group has proposed a new way of providing a non-nociceptive tactile sensation with laser [1]. In this study, we aimed to investigate laser-induced somatosensory information represented in cortical activity using the human EEG. The EEG data were acquired using the V-Amp amplifier (Brain Products GmbH, Gilching, Germany) with 16 wet electrodes that were placed on the scalp following the international 10–20 system. Twenty one subjects participated in the study (7 female and mean age of 22.4 years). During the experiment, a mechanical stimulus, a laser stimulus and a heat stimulus were given in a random order to subjects sixty times per stimulus. Subjects described the feeling of laser stimulation as non-painful sensation, painful sensation and no sensation. As described in the previous study, 56.3, 12.3 and 31.4 % of the subjects reported laser stimulation as non-painful, painful and no sensation, respectively [1]. To examine similarity of cortical activity in response to different stimuli, we employed a decoding analysis of the EEG data. In the decoding analysis, we used the linear discriminant analysis (LDA) method to classify the beta (21–28 Hz) event-related desynchronization/synchronization (ERD/S) patterns of EEG into one of the two classes representing every pair of stimuli (a total of six pairs from four stimuli) [2]. Classification error indicated how similar beta ERD/S patterns were between two stimuli: a larger error reflected more difficulty in discriminating patterns and consequently a greater similarity between patterns. The beta ERD/S patterns were estimated using the short time Fourier transform. Baseline correction was implemented using the 0.5 s period before stimulus onset. For each pair of stimuli, one-way ANOVA was used to select four channels that exhibited the most differences in beta ERD/S patterns between classes and classification accuracy was assessed by the leave-four-out cross validation [3] (see Fig. 69 for the classification error between every stimulus pair). The classification results showed that to the beta ERD/S pattern induced by mechanical stimulation, the pattern by non-painful laser stimulation was most similar. Also, the results indicated closeness of cortical activities between non-painful and painful laser stimulations as well as painful laser and thermal stimulations (see Fig. 69). These results suggest that laser might induce similar beta responses whether it evoked painful or non-painful feelings but non-painful laser might share presumably non-nociceptive somatosensory information with mechanical stimulation whereas painful laser shared presumably nociceptive somatosensory information with thermal stimulation. We expect that further information theoretical analyses may reveal more details about somatosensory information encoded in cortical rhythms induced by laser.

**References**

1. Jun J-H, Park J-R, Kim S-P, Bae YM, Park J-Y, Kim H-S, Choi S, Jung SJ, Park SH, Yeom D-I. Laser-induced thermoelastic effects can evoke tactile sensations. Sci Rep. 2015;5.
2. Pfurtscheller G, Lopes Da Silva FH. Event-related EEG/MEG synchronization and desynchronization: basic principles. Clin Neurophysiol. 1999;110(11):1842–57.
3. Celisse A, Robin S. Nonparametric density estimation by exact leave-p-out cross-validation. Comput Stat Data Anal. 2008;52(5):2350–68.

## P126 Phase synchronization of alpha activity for EEG-based personal authentication

### Jae-Hwan Kang^1^, Chungho Lee^1^, Sung-Phil Kim^1^

#### ^1^Department of Human and Systems Engineering, Ulsan National Institute of Science and Technology, Ulsan

##### **Correspondence:** Sung-Phil Kim - spkim@unist.ac.kr

*BMC Neuroscience* 2016, **17(Suppl 1)**:P126

**Fig. 70:**
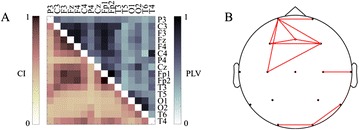
Overall characteristics of alpha phase synchronization for PA. **A** The upper triangle of association matrix indicates the PLV calculated by grand mean phase coherence. The lower triangle of association matrix indicates the its CI values in pairs. **B** Topographical connections with the 12 top-ranked CI from the lower triangle of **a**

There has been a growing interest in the EEG-based biometric system as an alternative approach to personal authentication (PA). In this study, we focused on the potentialities of functional connectivity, especially phase synchronization in the alpha rhythm represented by the phase locking value (PLV) as a novel EEG signature for PA. We analyzed an EEG dataset of 39 trials from 7 subjects who participated in the 5–7 sessions repeatedly on different days. In the sessions, a total of 16 EEG signals were acquired by a portable EEG device, when the subjects were in a resting state with their eyes closed for 2 min. The characteristics of alpha phase synchronization were estimated by the following procedures. (1) The alpha rhythm was extracted from the EEG signals using a band-pass filter of 8–13 Hz. (2) We randomly selected 20 2-s time segments of the alpha rhythm and calculated the mean phase coherence [1] between channels within each time segment. From all possible pairs of 16 EEG channels, a total of 120 mean alpha phase coherence values were extracted. 3) From these mean alpha phase coherence values, we calculated a criteria index (CI) of each of them where the CI calculated the ratio of an inter-subject variability to an intra-subject variability, which was developed to discriminate critical EEG features for PA in our previous study [2]. 4) Using the mean alpha phase coherence and its CI values, we constructed the association matrix of phase coherence and extracted the 12 top-ranked CI connections (Fig. 70). The topographical result showed that there were apparently two functional connectivity networks of the alpha rhythm in the brain for PA. The first network was distributed over the anterior regions including the pre-frontal, frontal and left central regions. The second one was located in the posterior region covering the occipital region. It should be noted that two regions have been well known as main sources of the alpha rhythm from many studies on EEG alpha rhythms. Our results suggest an important role of the alpha rhythm in the EEG-based biometrics system.

**Acknowledgements:** This work was supported by Institute for Information and communications Technology Promotion (IITP) grant funded by the Korea government (MSIP) (R0190-15-2054, Development of Personal Identification Technology based on Biomedical Signals to Avoid Identity Theft).

**References**

1. Mormann F, Lehnertz K, David P, Elger CE. Mean phase coherence as a measure for phase synchronization and its application to the EEG of epilepsy patients. Phys D. 2000;144:358–69.
2. Kang J-H, Lee C, Kim S-P. EEG Feature Selection and the use of Lyapunov exponents for eeg-based biometrics. In: IEEE international conference on biomedical and health informatics, Las Vegas, NV, USA; 2016. p. 1–4.

## P129 Investigating phase-lags in sEEG data using spatially distributed time delays in a large-scale brain network model

### Andreas Spiegler^1^, Spase Petkoski^1,2^, Matias J. Palva^3^, Viktor K. Jirsa^1^

#### ^1^INSERM UMR 1106 Institut de Neurosciences des Systèmes - Aix-Marseille Université, Marseille, France; ^2^Aix-Marseille Université, CNRS, ISM UMR 7287, 13288, Marseille, France; ^3^Neuroscience Center, University of Helsinki, Helsinki 00014, Finland

##### **Correspondence:** Andreas Spiegler - Andreas.Spiegler@univ-amu.fr

*BMC Neuroscience* 2016, **17(Suppl 1)**:P129

On a large scale, the brain appears as a network composed of white matter tracts connecting brain areas within and between cerebral hemispheres. The finite transmission speed delays the interaction of areas via these pathways. The delays are on the same scale as the brain oscillates, that is, 10–250 ms [1], and have been suggested to play a role in the functional organization. One potential key mechanism is synchronization [2], which could explain the phase-lags of brain signals. In humans, stereotactical EEG (sEEG) revealed frequency-specific inter-areal synchronization often associated with nearly zero phase-lag, or with variable phase-lags between ±π.

**Fig. 71:**
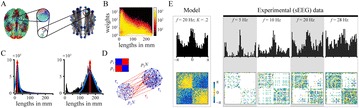
**A** Model with local (*left*) and long-range connections (*right*). **B, C** Averaged tract lengths and weights from 4 connectomes **B** Joint distribution, and **C** histogram of weighted lengths for intra- and inter-hemispheric links. **D** Sketch of the spatial delay structure. **E** Phase-lag distributions (*top*) and phase-lags between areas (*bottom rows*)

With the advance of non-invasive imaging techniques, large-scale modeling of the entire brain has become feasible using realistic connectivity and time delays [3], Fig. 71A. From structural and diffusion MRI, we obtained human connectomes composed of the strength and length of connections among 68 cortical areas. We approximated the bimodal tract length distribution (Fig. 71B) by Dirac deltas (Fig. 71C). Intra-hemispheric connections are in the 1st, and inter-hemispheric ones are in the 2nd mode. The delay of a connection was determined from its length divided by the speed of 5 m/s. The Kuramoto phase oscillator described the activity in each area. The phase difference of areas was analyzed and compared with the map of inter-areal phase lags obtained from resting state sEEG of epileptic patients.

The model of fixed oscillators (e.g., *f* = 20 Hz) switched from global incoherence to alternating in- and anti-phase coherence with increasing coupling strength. Increasing the natural frequencies for constant coupling resulted in alternating switching from in- to anti-phase coherence, but also to incoherence. Intra-hemispheric links were in-phase (phase-lags ~0), and inter-hemispheric links were either in- or anti-phase (±π), see clusters in Fig. 71E. Links among areas of low in-strength (sum of all the weights for that node) showed flatly distributed phase-lags. For *f* = 20 Hz, we found the phase-lags in the sEEG in the regime of in- and anti-phase coherence in the model, Fig. 71E.

We demonstrated that it is not simply the connectivity strength that matters in oscillatory large-scale brain networks, but time delays are of equal importance. The spatial structure in the time delays is reflected in the clustering of phase-lags. The model captured the statistics of the phase-lags as observed in the experimental data. The phase-lag structure of links at *f* = 20 Hz is explained in the model by a spatial organization of in- and anti-phase coherence.

**References**

1. Buzsáki G, Draguhn A. Neuronal oscillations in cortical networks. Science. 2004;304(5679):1926–29.
2. Varela F, Lachaux J, Rodriguez E, Martinerie J. The brainweb: phase synchronization and large-scale integration. Nat Rev Neurosci. 2001;2(4): 229–39.
3. Deco G, Jirsa V, McIntosh AR, Sporns O, Kötter R. Key role of coupling, delay, and noise in resting brain fluctuations. Proc Natl Acad Sci USA. 2009;106(25):10302–07.

## P130 Epileptic seizures in the unfolding of a codimension-3 singularity

### Maria L. Saggio^1^, Silvan F. Siep^1^, Andreas Spiegler^1^, William C. Stacey^2^, Christophe Bernard^1^, Viktor K. Jirsa^1^

#### ^1^INSERM UMR 1106 Institut de Neurosciences des Systèmes - Aix-Marseille Université, Marseille, France; ^2^Department of Neurology, ^2^Department of Biomedical Engineering, University of Michigan, Ann Arbor, MI 48109, USA

##### **Correspondence:** Maria L. Saggio - marisa.saggio@gmail.com

*BMC Neuroscience* 2016, **17(Suppl 1)**:P130

Seizures can arise under a variety of conditions. Despite this fact, there are invariant features resulting in a characteristic electrophysiological signature. Investigations of these universal properties lead to a classification of a planar description of point-cycle fast-slow bursters [1] and a taxonomy of seizures [2]. The phenomenological model, the epileptor [2], is able to reproduce the main features of the predominant class of human seizure (~80 % of all cases), according to data from epileptic patients. We aim at generalizing this model to include other bursting classes of the taxonomy.

We extended the work by [3] on bursters, living in the unfolding of high codimension singularities, and systematically investigated the unfolding of the codimension-3 degenerate Takens–Bogdanov bifurcation (focus, elliptic, saddle and cusp cases) [4]. The biological relevance of this codimension-3 bifurcation has been highlighted by other authors, and several classes appearing in the context of neuronal spiking have been identified in its unfolding (e.g., [5]). However, a systematic search in this unfolding for all the planar point-cycle bursting classes predicted by [1] was still missing. The existence of 16 bursters of the type *slow*-*wave* (self-oscillating slow subsystem), and 16 of type *hysteresis*-*loop* (slow-subsystem oscillating thanks to feedback from the fast one) was predicted.

We could find all slow-wave bursters in the unfolding together with seven of the hysteresis-loop ones. With regard to these hysteresis-loop bursters, we propose a model able to reproduce each of them depending only on the initial and final points of the path in the unfolding’s parameter space. This model is based on the known normal form of the codimension-three bifurcation [4], therefore we can readily describe the role of all its variables and how the tuning of its parameters affects the models activity. We found that the codimension-three model incorporates not only the repertoire (80 % of seizure) of the model proposed by [2] but also the classes that account for the remaining 20 % of seizures. Moreover, based on an ultra-slow modulation of the bursting path (see also [6]) in the model, possible transitions between bursting classes and, more importantly, transitions to regimes (in the parameter space) where bursting behavior is not possible at all could be predicted. These predictions could be tested using data from epileptic patients for whom different types of seizures coexist.

Overall, the main points of the present work are threefold: (i) a model description comprising the complete set of slow-wave bursters and seven (out of 16) hysteresis-loop bursters predicted by Izhikevich [1], (ii) a generalization of the model proposed by [2] to include the missing seizure types found in human data and to make prediction about their robustness, (iii) a framework to investigate the coexistence of different seizure types in the same patient and the transitions between them. The possibility of describing different seizure types with a unique model, thus with a unique set of variables and parameters, will facilitate the search for physiological correlates and treatments.

**References**

1. Izhikevich EM. Neural excitability, spiking and bursting. IJBC. 2000;10(6):1171–266.
2. Jirsa VK, Stacey WC, Quilichini PP, Ivanov AI, Bernard C. On the nature of seizure dynamics. Brain. 2014;137(8):2210–30.
3. Golubitsky M, Josic K, Kaper TJ. An unfolding theory approach to bursting in fast-slow systems. In: Krauskopf B, Broer HW, Vegter G, editors. Global analysis of dynamical systems; 2001. p. 227–308.
4. Dumortier F,Roussarie R, Sotomayor J, Żaładek H. Bifurcations of planar vector fields—nilpotent singularities and abelian integrals. Berlin: Springer; 1991.
5. Osinga HM, Sherman A, Tsaneva-Atanasova K. Cross-currents between biology and mathematics: the codi-mension of pseudo-plateau bursting. DCDS-A. 2012;32(8):2853–77.
6. Franci A, Drion G, Sepulchre R. Modeling the modulation of neuronal bursting: a singularity theory approach. SIAM J Appl Dyn Syst. 2014;13(2):798–829.

## P131 Incremental dimensional exploratory reasoning under multi-dimensional environment

### Oh-hyeon Choung^1^, Yong Jeong^1^

#### ^1^Department of Bio and Brain Engineering, KAIST, Daejeon, 34141, South Korea

##### **Correspondence:** Oh-hyeon Choung - iohyeonki@kaist.ac.kr

*BMC Neuroscience* 2016, **17(Suppl 1)**:P131

In our daily life, we encounter thousands of complex problems, which are not ‘one dimensional’. However, multi-dimensional problems were known to suffer from “curse of dimensionality” [1]. Therefore, the researches of reward learning and goal-directed behavior were mostly focused on single dimensional environment for a decade [2]. Even a few researches on multi-dimensional tasks was emphasizing that human representation learning is done by reducing the dimensionality, but not focusing on multiple compositional reasoning under multi-dimensional environment [3].

**Fig. 72:**
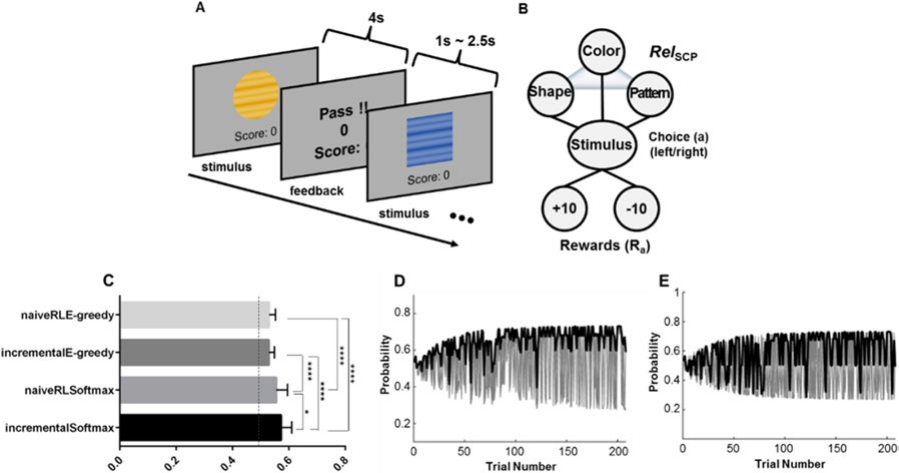
Multidimensional decision making task design and model comparison. **A** Multidimensional decision making task schematics. **B** Systemic structure of the task. **C** The result of model comparison, proposed model has significantly high accuracy on prediction. **D**, **E** The models’ prediction accuracy of proposed model (**D**) and naïve model (**E**)

Here, the multi-dimensional decision task was conducted (Fig. 72A, B) and the framework of Reinforcement Learning (RL) was used for analysis. We investigated that the reasoning under multi-dimensional environment is processed in incremental order, rather than one-shot learning. Also, the exploration of the best strategy occurs depends more on internal value, that is exploring under low value and exploiting under high value (softmax decision rule) rather than random exploration (randomized ɛ-greedy algorithm). Functional MRI were taken on each subject, while conducting the behavioral task. Brain regions of the incremental learning and the value sensitive explorative behavior will be verified.

We demonstrated that incremental learning rule can explain the multidimensional reasoning process better than other models (Fig. 72C–E). This result indicate that people deal with the complicated multi-dimensional problem, we solve them by adding dimensional information one by one.

**References**

1. Sutton RS, Barto AG. Reinforcement learning: An introduction. MIT Press; 1998.
2. Niv Y, Daniel R, Geana A, Gershman SJ, Leong YC, Radulescu A, Wilson RC. Reinforcement learning in multidimensional environments relies on attention mechanisms. J Neurosci. 2015;8145–57.
3. Lee SW, Shimojo S, O’Doherty JP. Neural computations underlying arbitration between model-based and model-free learning. Neuron. 2014;687–99.

## P132 A low-cost model of eye movements and memory in personal visual cognition

### Yong-il Lee^1,2^, Jaeseung Jeong^1,2^

#### ^1^Department of Bio and Brain Engineering, College of Engineering, Korea Advanced Institute of Science and Technology (KAIST), Daejeon, 34141, South Korea; ^2^Program of Brain and Cognitive Engineering, College of Engineering, Korea Advanced Institute of Science and Technology (KAIST), Daejeon, 34141, South Korea

##### **Correspondence:** Jaeseung Jeong - jsjeong@kaist.ac.kr

*BMC Neuroscience* 2016, **17(Suppl 1)**:P132

Eye movements are the most useful and clearest signal of our body to understand cognitive process and memory mechanism. Because it is convenient to measure the stream signal and quantify. Other sensory inputs, like auditory, gustatory, olfactory, and kinesthetic stimuli, are hard to estimate, but visual stimuli are easy by the eye-tracker [1]. And people are primarily visually oriented. Every day, people get over 80 percent of information from their own eye.

Until now, eye movements data has been measured and analyzed by very expensive eye-trackers mostly. The major companies’ devices price, including SMI, Tobii and EyeLink, are at least 10,000$ with their analysis tool SW. Of course, there have been many substitution trials in open SW and open HW area [2]. But their performance is certainly lower than major brands. Also, their data representation doesn’t have standard. Therefore, open SWs are difficult to apply for other utility services and scientific researchers have hesitated to use them to analyze result data to understand complex cognitive process. A few research results, which is investigating the relation between eye movement and cognitive model based on open eye-tracker platform, have reported by this time.

**Fig. 73:**
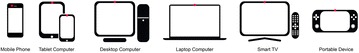
Low-cost eye movement tracker using front cameras on the each devices

However, eye-tracker is not only for science, and other areas need the usability of eye movement, for instance, UI/UX, healthcare, driving, game, learning consulting, TV viewer rating, market research and so on [3]. If there is a more general and low-cost eye-tracker which is confirmed cognitive model, above areas would be effective and we could do better decision making. This research implements a low-cost eye-tracker using a front camera (webcam) and a pin camera (Fig. 73, If the pc or laptop has laptop has a front camera, it doesn’t need more pin camera). The implementation includes the auto detection and classifying of useful memory based on eye movement of visual information on the device’s display. To do this function, the camera measures the saccade variation spectrum, as the X–Y axis acceleration, and categorize individual pattern while the user is taking train session. It is developed using OpenCV library and C#. XLabs Inc., already has made the gaze/head tracker using front camera without cognitive pattern analysis [4]. In the future, we will try this function on the mobile devices, which are cellular phone, tablet pc, and game interface. These devices have more sensors, like GPS, illumination, and activity accelerator. Combination of sensors input would make more precise prediction for memory cognition.

**References**

1. Wedel M, Pieters R: Eye tracking for visual marketing. Now Publishers Inc; 2008.
2. Dalmaijer ES, Mathôt S, Van der Stigchel S. PyGaze: an open-source, cross-platform toolbox for minimal-effort programming of eyetracking experiments. Behav Res Methods. 2014;46(4):913–21.
3. Pannasch S, Helmert JR, Velichkovsky BM. Eye tracking and usability research: an introduction to the special issue. J Eye Mov Res. 2008;2(4):1–4.
4. https://xlabsgaze.com/about/.

## P133 Complex network analysis of structural connectome of autism spectrum disorder patients

### Su Hyun Kim^1,2^, Mir Jeong^1^, Jaeseung Jeong^1,2^

#### ^1^Department of Bio and Brain Engineering, College of Engineering, Korea Advanced Institute of Science and Technology (KAIST), Daejeon, 34141, Korea; ^2^Program of Brain and Cognitive Engineering, College of Engineering, Korea Advanced Institute of Science and Technology (KAIST), Daejeon, 34141, Korea

##### **Correspondence:** Jaeseung Jeong - jsjeong@kaist.ac.kr

*BMC Neuroscience* 2016, **17(Suppl 1)**:P133

**Background** Human connectome which is the map of full connection of neuronal network in the human brain exhibits the characteristics of complex network [1]. The human connectome is known to be wired in a way that neurons efficiently transmit and communicate information. Statistical measures of complex network describe the topological features of a certain network and this enables researchers to compare effectiveness of information processing within a network. Autism spectrum disorder (ASD) subjects exhibit repetitive behaviors, impaired social communication skills, and sensory problems. Those symptoms of neurodevelopmental disorder is doubted to be originated from genetic causes [2]. Also, recent investigations find that ASD is a ‘connection problem’. But still exact cause of ASD is unknown. The aim of the research to be conducted is to reveal the genetic cause of (ASD) by the statistical analysis of network measures of ASD patients and normal groups’ structural connectome data using diffusion tensor imaging (DTI).

**Methods** The method to be used in the research is to compare the network measure values of various subject groups’ connectome and relate the difference to the genetic mutations in common. DTI data describes the neuronal connection of the brain regions in the mesoscale level. Structural connectome that is constructed from DTI information.

Expected result is that there are genotypic changes of genes which affect development of neuronal connection in ASD subjects. This finding will shade a new light on the investigation of ASD diagnosis and treatment.

**References**

1. Bullmore E, Sporns O. Complex brain networks: graph theoretical analysis of structural and functional systems. Nat Rev Neurosci. 2009;10(3):186–98.
2. Freitag CM, Staal W, Klauck SM, Duketis E, Waltes R. Genetics of autistic disorders: review and clinical implications. Eur Child Adolesc Psychiatry. 2010;19(3):169–78.

## P134 Cognitive motives and the neural correlates underlying human social information transmission, gossip

### Jeungmin Lee^1,2^, Jaehyung Kwon^1^, Jerald D. Kralik^1,2^, Jaeseung Jeong^1,2^

#### ^1^Department of Bio and Brain Engineering, College of Engineering, Korea Advanced Institute of Science and Technology (KAIST), Daejeon, 34141, Republic of Korea; ^2^Program of Brain Engineering, College of Engineering, Korea Advanced Institute of Science and Technology (KAIST), Daejeon, 34141, Republic of Korea

##### **Correspondence:** Jaeseung Jeong - jsjeong@kaist.ac.kr

*BMC Neuroscience* 2016, **17(Suppl 1)**:P134

Gossip is a specific example of human conversation containing social factors and has been considered as malicious, useless idle-talk by general population. Several researchers have suggested the role of gossip as social police that control the members of social groups to behave cooperative rather than selfish. However, there is not enough data to explain the actual cognitive motives that drive people to spread gossip. Throughout this study, human gossiping behavior is defined as transmission of social information about an absent third-party (i.e. the target of the gossip). In order to define the types of gossip, various scenarios containing social information are divided into 48 different categories by the third-party identity, valence and contents. Big five personality inventory, prosocial personality battery, cultural orientation scale and moral foundations questionnaire were used to measure personal traits that may influence the gossiping behavior of individuals. We found out that people, regardless to the scores of their personal traits, tend to spread gossip about in-group and celebrities more than out-group members. We also found out that positive gossip about in-group members is spread with significantly higher rates than in-group negative gossip, whereas the spread pattern was the opposite when the gossip is about celebrities. With such findings, we conducted fMRI study using in-group and out-group gossip with positive and negative valence. Increased activity in various brain regions including medial frontal gyrus, dorsolateral prefrontal cortex and precuneus was found when participants made decisions whether or not to spread gossip. With the obtained data, we tried to construct a computational model that may be used for classification of spread gossip.

## P135 EEG hyperscanning detects neural oscillation for the social interaction during the economic decision-making

### Jaehwan Jahng^1,2^, Dong-Uk Hwang^3^, Jaeseung Jeong^1,2^

#### ^1^Department of Bio and Brain Engineering, College of Engineering, Korea Advanced Institute of Science and Technology (KAIST), Daejeon, 34141, South Korea; ^2^Program of Brain and Cognitive Engineering, College of Engineering, Korea Advanced Institute of Science and Technology (KAIST), Daejeon, 34141, South Korea; ^3^Division of Computational Mathematics, National Institute for Mathematical Sciences (NIMS), Daejeon, 34047, South Korea

##### **Correspondence:** Jaehwan Jahng - jahngjh.627@gmail.com

*BMC Neuroscience* 2016, **17(Suppl 1)**:P135

**Fig. 74:**
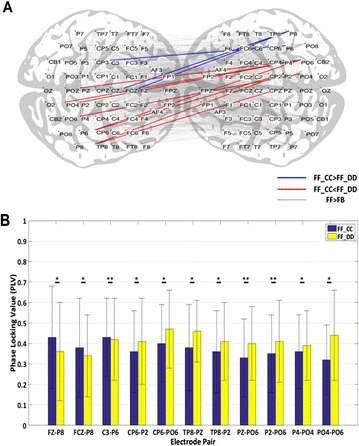
**A** Brain synchrony analyses. Intra-brain and inter-brain phase synchronies in alpha band [0.5, 1] s. Links between electrodes means that the phase activities there are synchronized. All synchronies here were higher in FF groups than in FB groups (*gray line*). *Blue line* denotes the synchronies that were higher in CC epochs compared with DD epochs of FF groups (CC > DD) whereas *red line* denotes the synchronies that were higher in DD epochs compared with CC epochs of FF groups (CC < DD). Intra-brain synchronies are drawn in both brains and only one of each pair of inter-brain synchronies are drawn. Significant level was at p < 0.05, Bonferroni corrected. **B** Magnitudes of phase synchronies that showed significant strategical differences. These correspond to the links depicted as *blue and red line* in **A**. * p < 0.05; ** p < 0.01, Bonferroni corrected

Social interaction is an important feature of the economic exchange. However, little is known about the varying neural mechanism during the economic decision-making depending on the different degrees of the social interaction. In this study, we used an iterated version of Prisoner’s dilemma game (PDG) with an EEG hyperscanning to investigate how the presence of face-to-face interaction modulates the social interactions and in turn the aspects of an economic decision-making. Participants played the game either face-to-face (FF) or face-blocked (FB). On the behavioral level, face-to-face interaction led both participants to choose cooperative strategies more often. On the neural level, FF groups showed significantly different alpha power during the first 0.5 s after seeing each outcome compared with FB groups in right temporo-parietal region. By computing the phase locking value (PLV), we measured the brain synchrony and found that the inter-brain phase synchronies across right temporo-parietal area were significantly associated with both the group differences and strategical differences of both players (Fig. 74). These results suggest that inter-brain alpha synchronies across right temporo-parietal area might serve as an implicit neural marker for both the social interaction level and intention to either cooperate or defect. Moreover, our results warrant the future hyperscanning studies on the social interactions of autism spectrum disorder (ASD) patients as all neural substrates revealed are known to be deeply associated with their social traits.

**Acknowledgements:** This research was supported by the CHUNG Moon Soul Research Center for Bio Information and Bio Electronics (CMSC) in KAIST and a Korea Science and Engineering Foundation (KOSEF) grant funded by the Korean government (No. 2006-2005399). The funders had no role in study design, data collection and analysis, the decision to publish, or the preparation of the manuscript.

## P136 Detecting purchase decision based on hyperfrontality of the EEG

### Jae-Hyung Kwon^1,2^, Sang-Min Park^1,2^, Jaeseung Jeong^1,2^

#### ^1^Department of Bio and Brain Engineering, Korea Advanced Institute of Science and Technology (KAIST), Daejeon, 34141, South Korea; ^2^Program of Brain and Cognitive Engineering, Korea Advanced Institute of Science and Technology (KAIST), Daejeon, 34141, South Korea

##### **Correspondence:** Jae-Hyung Kwon - jh2393@kaist.ac.kr

*BMC Neuroscience* 2016, **17(Suppl 1)**:P136

Understanding and predicting purchase decision process is one of the fundamental issues in economics, marketing, decision sciences, yet an easily accessible means to monitoring purchase decisions has not been developed yet [1–3]. Using event-related functional fMRI, such purchase behavior with a shopping task was investigated [4] but it has limit on potential practical uses due to the cost and portability of the MRI. The electroencephalogram (EEG) has been suggested to have many advantages for applications in marketing due to its relatively low cost, portability, and high temporal resolution. The aim of the current study was to determine the possibility of the EEG as a tool for detecting and predicting purchase decision in potential consumers. Twenty-three participants were recruited to record their EEGs as they saw the pictures of products followed by the products’ prices and made the choice of whether to buy them or not. We estimated the power spectra and approximate entropy (ApEn), an information-theoretic measure to quantify the complexity [5], of their EEGs and compared them for purchase and non-purchase trials. The support vector machine (SVM) method was to predict their purchase decisions. We found that the relative spectral powers and ApEn values of the EEG significantly differed between purchase and non-purchase trials, in particular frontal regions. SVM could distinguish and predict purchase and non-purchase decisions based on the spectral powers and ApEn values of the EEGs in frontal regions prior to the decision moment with a high accuracy (>87 %). This finding suggests that relatively inexpensive, portable EEG recording technique has great potential as a neural predictor of purchase behavior in neuromarketing and neuroeconomics.

**References**

1. Lee N, Broderick AJ, Chamberlain L. What is “neuromarketing”? A discussion and agenda for future research. Int J Psychophysiol. 2007;63:199–204.
2. Mirja Hubert PK. A current overview of consumer neuroscience. J Consum Behav. 2008;7:272–92.
3. Ariely D, Berns GS. Neuromarketing: the hope and hype of neuroimaging in business. Nat Rev Neurosci. 2010;11:284–92.
4. Knutson B, Rick S, Wimmer GE, Prelec D, Loewenstein G. Neural predictors of purchases. Neuron. 2007;53:147–56.
5. Gu F, Meng XIN, Shen E, Cai Z. Can we measure consciousness with EEG complexities? Int J Bifurc Chaos. 2003;13:733–42.

## P137 Vulnerability-based critical neurons, synapses, and pathways in the *Caenorhabditis elegans* connectome

### Seongkyun Kim^1^, Hyoungkyu Kim^1^, Jerald D. Kralik^1^, Jaeseung Jeong^1^

#### Department of Bio and Brain Engineering, Program of Brain and Cognitive Engineering, College of Engineering, Korea Advanced Institute of Science and Technology (KAIST), Daejeon, 34141, South Korea

##### **Correspondence:** Jerald D. Kralik - jerald.kralik@raphe.kaist.ac.kr, Jerald D. Kralik - jsjeong@kaist.ac.kr

*BMC Neuroscience* 2016, **17(Suppl 1)**:P137

Determining the fundamental architectural design of complex nervous systems will lead to significant medical and technological advances. Yet it remains unclear how nervous systems evolved highly efficient networks with near optimal sharing of pathways that yet produce multiple distinct behaviors to reach the organism’s goals. To determine this, we investigated the vulnerability of the nematode roundworm *Caenorhabditis elegans* connectome [1] by attacking each of 279 individual neurons and 6393 chemical synapses and 890 electrical junctions in the connectome, and quantifying the lethality of the network in terms of global information processing using graph-theoretic measures: i.e., examining vulnerability with respect to clustering (*C*), efficiency (*E*), and betweenness (*B*).

**Fig. 75:**
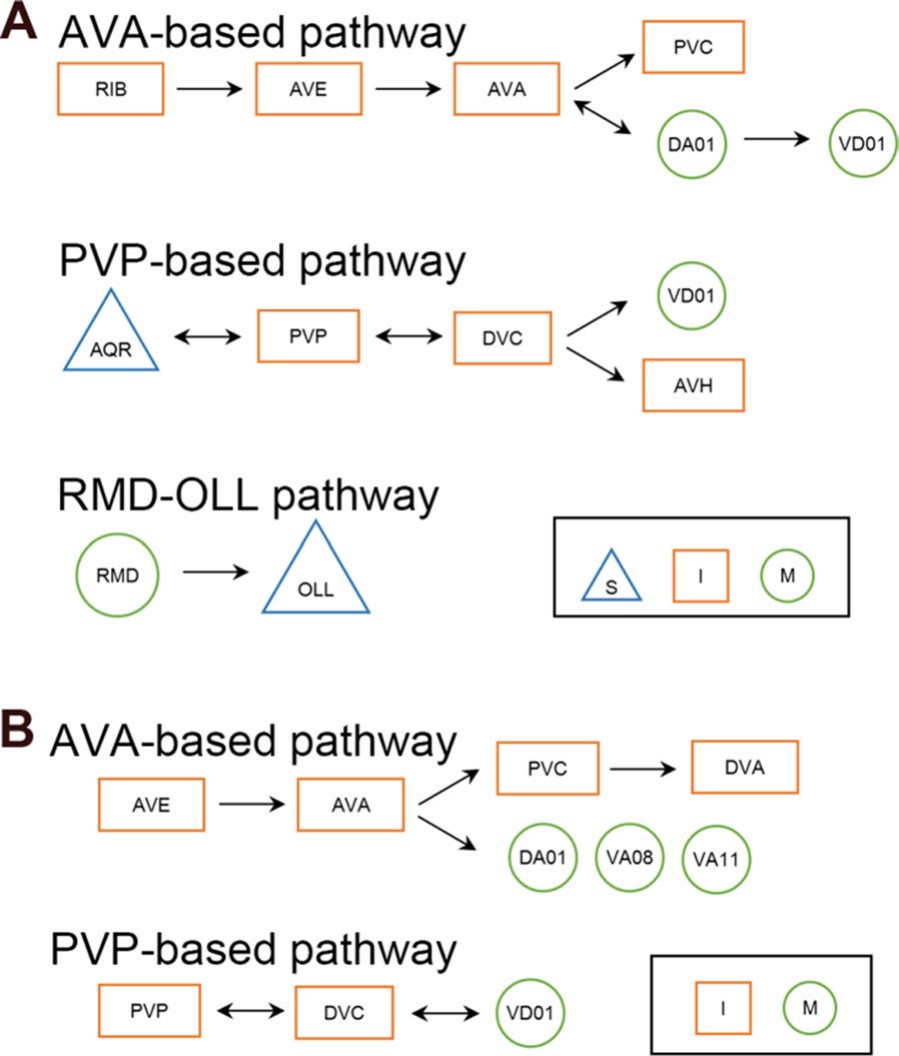
Three critical pathways emerged from the results. **A** For *V*_*B*_ they were: (1) AVA-based; (2) PVP-based; and (3) RMD → OLL. **B** Two of these pathways were again implicated for *V*_*E*_: the AVA-based and the PVP-based pathways

The vulnerability analyses, *V*_*C*_, *V*_*E*_, *V*_*B*_, identified 12 critical neurons and 29 critical synapses that are the most important components for establishing fundamental network properties. These critical elements were found to be control elements—i.e., those with the most influence over multiple underlying pathways. In addition, we found that the critical synapses formed into circuit-level control units, suggesting fractal-like control in the connectome. More specifically, three main critical pathways emerged from the results (Fig. 75A, B).

**Conclusions** The critical pathways that emerged from our computational analysis provide evidence for (a) the importance of backward locomotor control, avoidance behavior, and social feeding to the organism; (b) the potential roles of specific neurons whose functions have been unclear; and (c) both parallel and serial design elements in the connectome—i.e., specific evidence for a mixed architectural design. This design structure may be fundamental to nervous systems, providing necessary building blocks for the evolution of higher intelligence.

**Acknowledgements::** Supported by the National Research Foundation of Korea (NRF-2013R1A1A2011570).

**Reference**

1. Altun Z, Hall D. Worm Atlas; 2002. http://www.wormatlas.org.

## P138 Motif analysis reveals functionally asymmetrical neurons in *C. elegans*

### Pyeong Soo Kim^1^, Seongkyun Kim^1^, Hyoungkyu Kim^1^, Jaeseung Jeong^1^

#### ^1^Department of Bio and Brain Engineering, Korea Advanced Institute of Science and Technology (KAIST), Daejeon 305-701, South Korea

##### **Correspondence:** Jaeseung Jeong - jsjeong@kaist.ac.kr

*BMC Neuroscience* 2016, **17(Suppl 1)**:P138

Majority of animal species have bilaterally symmetrical nervous system. Symmetric and asymmetric features among their morphological symmetric nervous system have been interesting issue for long time. The simplest bilaterally symmetrical organism is nematode called *Caenorhabditis elegans*. Previously, symmetry for *C. elegans* has only been thoroughly studied in morphological and functional manner [1]. According to previous observation, there are 92 bilaterally symmetrical neuronal pairs and remaining 95 neurons are mostly located on the axis of symmetry. Functionally there are only 2 neuronal pairs that show asymmetrical gene expression among 92 pairs of symmetrical neurons. We examined the symmetry of *C. elegans* nervous network which has not been studied.

Total of 279 neurons and 2990 links in *C. elegans* were used. Neurons were classified into bilaterally symmetrical neurons, unlateral neurons, and unilateral neurons. According to the neuronal positions, we could define the symmetry of each individual link and expand that definition to define the symmetry of motif [2]. After defining symmetry of nervous network, we suggest a novel approach to classify asymmetric neurons of *C. elegans* nervous system by examining asymmetric network topology for every node. We defined 5 explicit locally topological parameters for a neuron; (1) the degree is defined as the number of asymmetric links attached to the neuron, (2) the motif is defined as distribution of the numbers of asymmetric motifs for a neuron, (3) the degree ratio is defined as ratio of asymmetric links over totally attached links to the neuron including both of symmetric links and asymmetric links, (4) the motif ratio is distribution of the rates for asymmetric motifs over total motifs including both of symmetric and asymmetric motifs, and (5) the relative distance is defined by the difference of asymmetric motif fingerprint of bilaterally symmetrical neurons. Thresholds were defined using mean and standard deviation (SD) values of asymmetries to find statistically asymmetric components. Neurons with asymmetry value over the threshold were considered as asymmetric neurons (asymmetric neurons > SD from the mean values). We checked our asymmetric neurons with ASE and AWC neurons that are only known to show bilaterally asymmetrical gene expression. As a result, our study suggested that (4) ratio of asymmetric motif and (5) relative distance measures successfully classified ASE and AWC as asymmetric neurons. Except for ASE and AWC neurons, BDU, PLM, and PVW neurons are classified asymmetric in both measures. These results could be interpreted that BDU neurons, PLM neurons, and ALN neurons might possess asymmetric features that have not been discovered.

**References**

1. Oliver H, Johnston RJ, Chang S. Left–right asymmetry in the nervous system: the *Caenorhabditis elegans* model. Nat Rev Neurosci. 2002;3(8):629–40.
2. Sporns O, Kötter R. Motifs in brain networks. PLoS Biol. 2004;2(11):e369.

## P139 Computational approach to preference-based serial decision dynamics: do temporal discounting and working memory affect it?

### Sangsup Yoon^1,2^, Jaehyung Kwon^1,2^, Sewoong Lim^1,2^, Jaeseung Jeong^1,2^

#### ^1^Department of Bio and Brain Engineering, College of Engineering, Korea Advanced Institute of Science and Technology (KAIST), Daejeon, 34141, South Korea; ^2^Program of Brain and Cognitive Engineering, College of Engineering, Korea Advanced Institute of Science and Technology (KAIST), Daejeon, 34141, South Korea

##### **Correspondence:** Jaeseung Jeong - jsjeong@kaist.ac.kr

*BMC Neuroscience* 2016, **17(Suppl 1)**:P139

When we face the multiple options (such as different taste of chocolates in a box) and choose one by one sequentially, there is no reason to prefer particular order of choice than any other possible choice strategies since all the items will eventually be consumed by ourselves. Recent studies have, however, revealed that there are distinct patterns of choice strategy in this preference-based serial decision tasks. Interestingly, there are two opposite choice patterns (favorite-first and favorite-last) in human subjects [1], while non-human animals (rhesus monkeys) only chose their favorite options at first [2]. Although several hypotheses for underlying neural mechanisms have been suggested to explain about how these distinct choice strategies appeared, they are not directly tested yet.

The goal of the current study was to examine whether temporal discounting and working memory affect choice strategy of serial decision-making and if so, to examine how they influence it. To measure the choice strategy, we used the modified version of ‘the sushi problem’ task [1], which use the pictures of opposite sex as a reward instead of the sushi [3]. We also measured the temporal discounting parameters and working memory performance by using the same set of opposite-sex pictures. The whole pictures were rated by each subject twice before the main experiment, the average rating score were subsequently used to divide the whole picture set into four groups based on the difference of subject specific preference. In ‘sushi’ task, subjects were asked to choose among four options (squares contain different number of stars; 1 ~ 4), each of which represents following short presentation (1.5 s) of pictures right after their choice. Each trial ends when subject choose all of four options, so they couldn’t skip or miss any options. The temporal discounting parameter was measured by the subject choice between sooner-small reward and later-larger reward, in this case, the magnitude of reward was the number of stars which indicate the subject-specific attractiveness of each pictures, the delay of reward was relatively shorter (1–30 s) than typical temporal discounting task since our task offered actual outcome (see the picture) of each choices [4]. The picture version of n-back task was used to measure the working memory performance.

Consistent with previous studies, we observed distinct patterns of choice order in ‘sushi’ task, favorite-last strategy was most dominant (58 %) and favorite-first was second (31 %). We also found the relationship of both temporal discounting and working memory with choice strategies. The favorite-last group showed significantly lower rate of temporal discounting and higher performance of working memory than favorite-first group. The effects of working memory and temporal discounting parameters on choice strategy were examined by logistic regression analysis, which revealed how the propensity to discount future events and the memory effect about recent events predicted the pattern of serial choice. We also constructed simple computational models using support vector machine and naïve Bayes classifier to predict their decision patterns based on working memory performance and temporal discounting parameters. We showed that these computational models successfully predicted preference-based decision patterns.

**References**

1. Jaeseung J, Younmin O, Miriam C, Jerald DK. Preference-based serial decision dynamics: your first sushi reveals your eating order at the sushi table. Plos One. 2014;9(5):e96653
2. Kanghoon J, Jerald DK. Get it while it’s hot: a peak-first bias in self-generated choice order in rhesus macaques. Plos One. 2013;8(12):e83814.
3. Itzhak A, Nancy E, Dan A, Christopher FC, Ethan O, Hans CB. Beautiful faces have variable reward value: fMRI and behavioral evidence. Neuron. 2001;32:537–51.
4. Benjamin YH, Purak CP, Robert OD, Michael LP. Economic principles motivating social attention in humans. Proc R Soc B. 2007;274:1751–56.

## P141 Social stress induced neural network reconfiguration affects decision making and learning in zebrafish

### Choongseok Park^1^, Thomas Miller^2^, Katie Clements^2^, Sungwoo Ahn^3^, Eoon Hye Ji^4^, Fadi A. Issa^2^

#### ^1^Department of Mathematics, North Carolina A&T State University, Greensboro, NC, 27411, USA; ^2^Department of Biology, East Carolina University, Greenville, NC, 27858, USA; ^3^Department of Mathematics, East Carolina University, Greenville, NC, 27858, USA; ^4^David Geffen School of Medicine, UCLA, Los Angeles, CA, 90095, USA

##### **Correspondence:** Choongseok Park - issaf14@ecu.edu, Fadi A. Issa - cpark@ncat.edu

*BMC Neuroscience* 2016, **17(Suppl 1)**:P141

In many social species, behavioral mechanisms of how social hierarchies formed and maintained have been studied extensively [1]. However, the neural bases underlying behavioral decisions and dynamics of neural circuits that permit animals to adapt to changes in social rank are poorly understood. In this study we focused on two social stress induced behaviors in zebrafish [the Mauthner cell (M-cell) mediated startle escape response and swimming behavior] to investigate how social regulation affects intrinsic cellular and network properties that result in the behavioral differences between dominant (DOMs) and subordinate (SUBs) animals. We utilized a non-invasive technique that allowed us to monitor the activation pattern of the two neural circuits in freely behaving animals.

High behavioral responsiveness and a low stimulus threshold for the initiation of escape in M-cell were observed in SUBs while DOMs showed the quicker habituation to repeated auditory stimulation compared to SUBs. We also observed that on average SUBs generated significantly less number of swim bursts compared to DOMs. These results suggest that social status induced stress can modify the startle plasticity as well as the local swimming circuit. The change in M-cell’s excitability due to the change in the presynaptic inhibitory drive may be responsible for the lowered threshold. On the other hand, the local neural circuits and their intrinsic modulatory components (motor neurons and interneurons) may be configured differently according to social status to produce status-dependent swim patterns [2].

To test these ideas, we developed a biologically-based mathematical model whose network architecture is based on recent experimental data [3]. The model is able to reproduce several hallmarks of social status induced behavioral differences that were experimentally observed between DOMs and SUBs, as well as some inherent activity patterns. Changing some intrinsic synaptic and network parameters was sufficient to obtain the transition between DOMs and SUBs activity patterns while maintaining the network architecture.

Recent experiments show that the startle plasticity in M-cell can be modulated by endocannabinoids, 2-AG [3]. We chose the availability of 2-AG in M-cell as one of main parameters for the simulation, whose dynamics is governed by the intracellular calcium level in M-cell. Model simulation shows that high behavioral responsiveness in SUBs results from the increased excitability in M-cell, which can be interpreted as the reduced inhibitory input to M-cell. To reproduce less swimming activity in SUBs, the hallmark of social status induced behavioral difference observed in our experiments, we chose another intrinsic parameter, the availability of 2-AG in inhibitory interneurons to represent 2-AG modulated local network property. Model simulation shows that less swimming activity in SUBs is produced by the increased inhibitory input to the swimming neural circuit via the 2-AG driven elevated interneuron activity.

**Acknowledgements:** This work was partially supported by Simons Foundation Collaboration Grants for Mathematicians (#317566) to CP and East Carolina University, Department of Biology fund to FAI.

**References**

1. Bergman TJ, Beehner JC, Cheney DL, Seyfarth RM. Hierarchical classification by rank and kinship in baboons. Science. 2003;302(5648):1234–36.
2. Issa FA, Drummond J, Cattaert D, Edwards DH. Neural circuit reconfiguration by social status. J Neurosci. 2012;32(16):5638–45.
3. Song J, Ampatzis K, Ausborn J, Manira AE. A hardwired circuit supplemented with endocannabinoids encodes behavioral choice in zebrafish. Curr Biol. 2015;25:2610–20.

## P142 Descriptive, generative, and hybrid approaches for neural connectivity inference from neural activity data

### JeongHun Baek^1^, Shigeyuki Oba^1^, Junichiro Yoshimoto^2,3^, Kenji Doya^2^, Shin Ishii^1^

#### ^1^Graduate School of Informatics, Kyoto University, Yoshidahonmachi 36-1, Sakyo, Kyoto, Japan; ^2^Neural Computation Unit, Okinawa Institute of Science and Technology Graduate University, 1919-1 Tancha, Onna-son, Kunigami-gun, Okinawa, Japan; ^3^Graduate School of Information Science, Nara Institute of Science and Technology,8916-5 Takayama, Ikoma, Nara, Japan

##### **Correspondence:** JeongHun Baek - ku21fang@gmail.com

*BMC Neuroscience* 2016, **17(Suppl 1)**:P142

Identification of the connectivity between neurons is important not only for elucidating neural bases for various functions but also for reconstructing the dynamics emerged in the connectivity. This thesis considers efficient methods for estimating synaptic connections from neural activity data. There are two kinds of approaches to neural connectivity inference: analytic one based on descriptive statistics and reproductive one based on statistical generative models. Analyses based on descriptive statistics, such as Pearson correlation, can identify neural connectivity based on activity data, with low computational cost. It, however, cannot reproduce the dynamic behaviors of the underlying connectivity, and hence, it is not suited for simulating the identified network.

Reproductive approach based on statistical generative models, such as generalized linear model, can naturally simulate the dynamic behaviors of the identified network, once we determine the network parameters from activity data. Contrary to this advantage, the computational cost of reproductive approach is often much heavier than analytic one. To utilize the preferable characters of the two approaches, in this study, we propose a hybrid approach of using a descriptive statistic for prescreening of existing connections and then performing generative model inference for dynamic model construction. We applied the hybrid approach to artificially generated spike data of various network sizes.

**Fig. 76:**
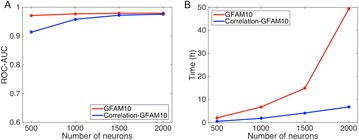
**A** Comparison of the prescreening accuracy in terms of ROC-AUC value (higher is better accuracy). **B** Comparison of the computation time. ‘GFAM10 [1]’, regarded as the original method, denotes generative functional additive model which is extended version of generalized linear model. ‘Correlation-GFAM10’ denotes a hybrid approach which performs the Pearson correlation for prescreening and then performs GFAM10

**Results and conclusions** Figure 76A shows the accuracy of functional connectivity analysis, in terms of ROC-AUC of binary classification, presence/absence of connectivity, where we see the hybrid approach performed slightly worse than the GFAM10. Note that the hybrid approach performed almost same with GFAM10 when the number of neurons was 2000. Figure 76B shows the computation time of the two methods, where we see that GFAM10 took five times as much time as the hybrid approach. Our hybrid approach successfully reduced computational time, into about one-fifth of that of the sole reproductive approach based on the GFAM, while maintaining the estimation accuracy of the response functions within the identified functional connectivity.

**Reference**

1. Song D, Wang H, Tu CY, Marmarelis VZ, Hampson RE, Deadwyler SA, Berger TW. Identification of sparse neural functional connectivity using penalized likelihood estimation and basis functions. J Comput Neurosci. 2013;35(3):335–57.

## P145 Divergent-convergent synaptic connectivities accelerate coding in multilayered sensory systems

### Thiago S. Mosqueiro^1^, Martin F. Strube-Bloss^2^, Brian Smith^3^, Ramon Huerta^1^

#### ^1^University of California San Diego, La Jolla CA, USA; ^2^Biocenter University of Würzburg, Würzburg, Germany; ^3^School of Life Sciences, Arizona State University, Tempe, AZ, USA

##### **Correspondence:** Brian Smith - brian.h.smith@asu.edu

*BMC Neuroscience* 2016, **17(Suppl 1)**:P145

**Fig. 77:**
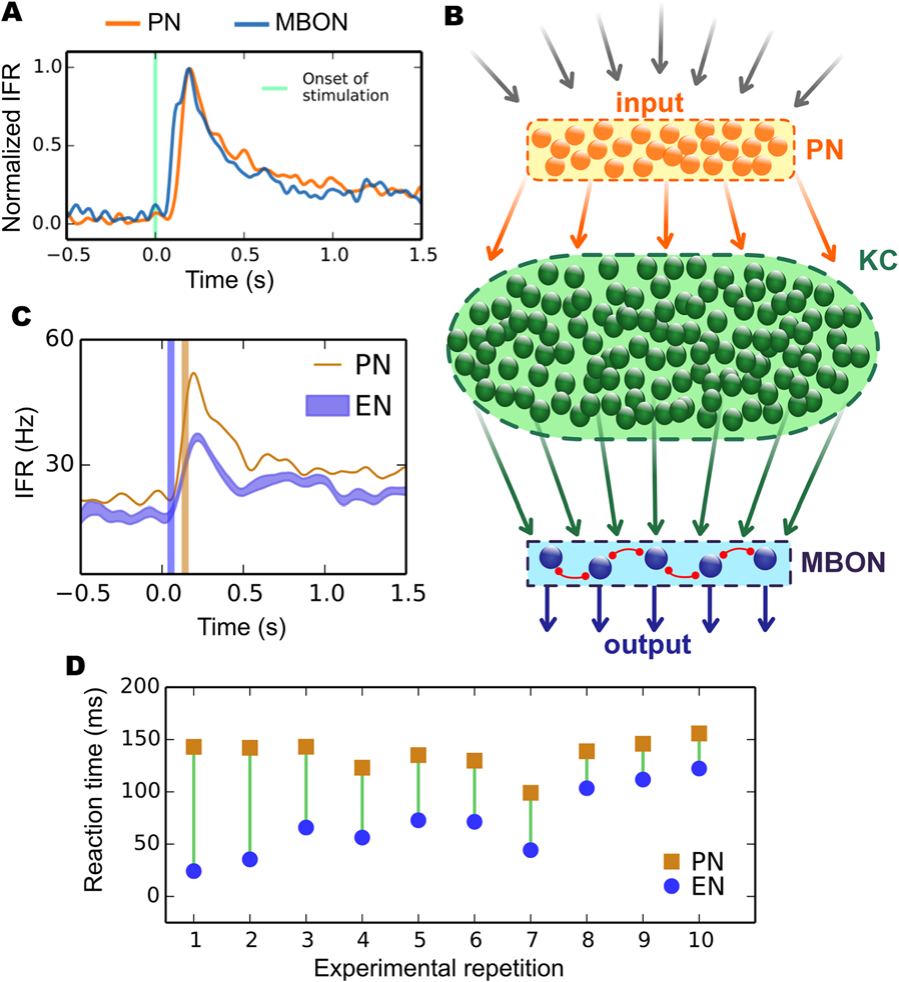
Early discrimination of stimulus in the MBs. **A** Recordings of PNs and MBONs activities from untrained honey bees to odor stimulation. At t = 0 s (*green bar*), an odor stimulation is presented. **B** Connectionist blueprint of the MBs, emphasizing synapses and population size. Note the divergence present between PN and KC layers, followed by convergence onto MBONs. **C** We reproduced in silico the early response (*blue bar*) of MBONs in the vertical lobe with respect to the PNs (*orange bar*) using spiking neuron networks. **D** Time differences in our simulations for each experiment repetition. Difference in response time between is on average 50 ms (one tail Mann–Whitney test, p < 0.025)

A central dogma in perception postulates that a minimal number of higher-order neurons provide the coding basis required for decision making and survival [1]. However, sensory information must travel through several neural layers before converging onto a smaller number of neurons in a premotor decision layer [2]. This multi-layered processing and convergence induces a time lag between peripheral input and adaptive behavior, which is inconsistent with the need for reaction speed. We propose that the divergent–convergent organization often occurring in multilayered neuropils enhances processing speed. Insect olfactory processing is a good model for investigating perceptual timing [3], where effective classification in the 4th layer ‘anticipates’ classification in input layers by 50 ms (Fig. 77A, B) [4]. Here we show that this anticipation emerges from divergent-convergent connectivity and the relative sizes of the layers, which rapidly amplifies subtle input signals and improves precision (Fig. 77C). We reproduced experimental results of peak classification in MBONs anticipating PNs by 50 ms on average (Fig. 77D). This becomes more pronounced as the KC layer grows, although increased noise is also observed. For an oversized KC layer, thus, this anticipation becomes lower and the signal is eventually destroyed by the emphasized noise. Interestingly, the key feature to this anticipation is indeed the ratio between KCs and PNs, showing that larger brains may balance these populations to achieve jointly higher pattern recognition capabilities and fasts discrimination times. We have analyzed fast coding properties of fan-out/fan-in structures that are ubiquitous in the brain. We developed a model to reproduce experimental data and analyze the optimal reaction times of the network model, finding a balance between fast information transmission and high accuracy in pattern recognition. Our contribution improves understanding of the role of divergent convergent feedforward networks on the stability of fast and accurate decision-making.

**References**

1. Barlow HB. Single units and sensation: a neuron doctrine for perceptual psychology? Perception. 2009;38:371–94.
2. Shepherd GM. The synaptic organization of the brain. Oxford: Oxford Press; 2003.
3. Mosqueiro TS, Huerta R. Computational models to understand decision making and pattern recognition in the insect brain. Curr Opin Insect Sci. 2014;6:80–5.
4. Strube-Bloss MF, Herrera-Valdez MA, Smith BH. Ensemble response in mushroom body output neurons of the honey bee outpaces spatiotemporal odor processing two synapses earlier in the antennal lobe. PLoS One. 2012;7:e50322.

## P146 Swinging networks

### Michal Hadrava^1,2,3^, Jaroslav Hlinka^2,3^

#### ^1^Department of Cybernetics, Faculty of Electrical Engineering, Czech Technical University in Prague, Prague, 166 27, Czech Republic; ^2^Department of Nonlinear Dynamics and Complex Systems, Institute of Computer Science, The Czech Academy of Sciences, Prague, 182 07, Czech Republic; ^3^National Institute of Mental Health, Klecany, 250 67, Czech Republic

##### **Correspondence:** Michal Hadrava - hadrava@cs.cas.cz

*BMC Neuroscience* 2016, **17(Suppl 1)**:P146

Nature is a powerful illusionist who, unfortunately for life sciences, hates revealing her secrets. One of her most rewarding tricks involves interconnecting a bunch of non-oscillatory neurons in such a way that they collectively behave like an oscillator [1]. Contemporary neuroscience strives to decipher this magic and does so not out of mere curiosity: the trick can go wrong, causing myriads of neurons to march to the deadly rhythm of epileptic seizure. It is of vital importance to determine which connectivity patterns promote and suppress epileptiform activity if surgery is to be effective when nothing else is [2]. On a lighter note, oscillatory dynamics explains many aspects of musical experience [3–5]. A question comes up again and again in ethnomusicological discourse as to whether these aspects are learned or not. In the context of oscillatory dynamics on networks, we might ask whether a single connectivity leads to the emergence of dynamics relevant to each musical culture or a different (learned) connectivity is at work each time. In conclusion, establishing a link between connectivity and oscillatory dynamics on networks seems to be an important problem with repercussions in such diverse fields as epileptology and ethnomusicology.

The mainstream approach to the problem can be characterized as follows: first, choose a dynamical model of single unit—e.g. neuron, synapse, or population thereof. Next, connect units of the selected type(s) in a network. Finally, study the effect of connectivity parameters on the global dynamics analytically, computationally, or using a combination of both. The major drawback of any analysis performed in this way is that the validity of its results is put in doubt whenever that of the single unit model is. Needless to say, none of the ever-growing variety of models has gained a wide acceptance yet. The mainstream approach could be dubbed the “object-oriented” one. The alternative approach, advocated by category theorists and adopted by us, could be called the “relational” one: instead of analysing a particular dynamical system, one investigates a whole class of dynamical systems on a particular manifold characterized only by its relations to classes of dynamical systems on different manifolds. This latter approach is epitomized by a recently introduced algebraic structure [6] which relates global network dynamics to its connectivity. We are currently trying to prove the existence of global periodic solutions in selected classes of simple networks with a given structure using this new theory.

**Acknowledgements:** This work was supported by the Grant Agency of the Czech Technical University in Prague, grant No. SGS14/192/OHK3/3T/13, the Czech Science Foundation Project No. P303-14-02634S, and the Czech Health Research Council Project No. NV15-29835A.

**References**

1. Buzsáki G. Rhythms of the brain. New York: Oxford University Press; 2006.
2. Dixit AB, Banerjee J, Tripathi M, Chandra PS. Presurgical epileptogenic network analysis: a way to enhance epilepsy surgery outcome. Neurol India. 2015;63(5):743–50.
3. Cartwright JHE, Gonzalez DL, Piro O. Pitch perception: a dynamical-systems perspective. Proc Natl Acad Sci USA. 2001;98(9):4855–59.
4. Large EW: A dynamical systems approach to musical tonality. In: Huys R, Jirsa VK, editors. Studies in computational intelligence: nonlinear dynamics in human behavior, vol 328. Berlin: Springe; 2011. p. 193–211.
5. Large EW, Snyder JS. Pulse and meter as neural resonance. In: DallaBella S, Kraus N, Overy K, Pantev C, Snyder JS, Tervaniemi M, Tillmann B, Schlaug G, editors. Neurosciences and music III: disorders and plasticity, vol 1169; 2009. p. 46–57.
6. Lerman E, Spivak DI. An algebra of open continuous time dynamical systems and networks. arXiv:1602.01017v1 [math.DS].

## P147 Inferring dynamically relevant motifs from oscillatory stimuli: challenges, pitfalls, and solutions

### Hannah Bos^1^, Moritz Helias^1,2^

#### ^1^Institute of Neuroscience and Medicine (INM-6) and Institute for Advanced Simulation (IAS-6) and JARA BRAIN Institute I, Jülich Research Centre, 52425 Jülich, Germany; ^2^Department of Physics, Faculty 1, RWTH Aachen University, 52074 Aachen, Germany

##### **Correspondence:** Hannah Bos - h.bos@fz-juelich.de

*BMC Neuroscience* 2016, **17(Suppl 1)**:P147

Applications of oscillatory stimuli in optogenetical studies have been used to gather evidence that γ oscillations are generated by the interaction of inter-neurons (also termed the inter-neuron γ or ING mechanism) [1, 2]. We elaborate the pitfalls of inferring the origin of the oscillation from absolute (response spectra) as well as relative (power ratios) changes in spectra of neural activity induced by oscillatory input. We consider minimalistic models that isolate the difficulties and limitations arising in the interpretation of response spectra. The described effects generalize to more realistic models. This is demonstrated in simulations of a multi-laminar model of V1 composed of leaky-integrate-and-fire (LIF) model neurons [3], where the ground truth regarding the sub-circuits generating the oscillations is known [4]. In this structured model these effects combine and yield misleading results. By extending mean-field theoretical descriptions of population dynamics [5] by oscillatory input, we can close the loop to the condensed models.

We identify three main complications: First, the input can modify the excitability of the population in a linear or non-linear fashion, yielding significantly different changes in the spectra. Second, depending on the properties of the system, the input to the populations is potentially low pass filtered before it enters the system. Since this low pass filter is reflected in the response spectra, without revealing information regarding the internal dynamics of the network, we propose a stimulation protocol counteracting this effect by emphasizing high frequencies. Third, in general, the stimulation of a single population excites a mixture of dynamical modes. One frequency is generated by one dynamical mode that can be mapped to its anatomical origin [4]. Since the observable response is composed of an inseparable mixture of modes, the mode generating the oscillation cannot easily be isolated. Hence reconstructing the underlying connectivity as well as identifying the role of the stimulated population in the generation of the rhythm is not straightforward. Instead, the stimulus vector needs to reflect the structure of the circuit generating the oscillation in order to allow insights into the dynamically relevant components of the system.

These problems can be regarded as a sub-set of challenges that need to be faced when interpreting the results of circuits composed of more complex units. The proposed solutions may be used to construct new experimental stimulation protocols.

**Acknowledgements:** We acknowledge funding by the Helmholtz Association: portfolio theme SMHB and Young Investigator’s Group VH-NG-1028, and 604102 (Human Brain Project). All network simulations were carried out with NEST (http://www.nest-initiative.org).

**References**

1. Cardin JA, Carlé M, Meletis K, Knoblich U, Zhang F, Deisseroth K, Tsai L-H, Moore CI. Driving fast-spiking cells induces gamma rhythm and controls sensory responses. Nature. 2009;459:663–7.
2. Buzsáki G, Wang XJ. Mechanisms of gamma oscillations. Annu Rev Neurosci. 2012;35:203–25.
3. Potjans TC, Diesmann M. The cell-type specific cortical microcircuit: relating structure and activity in a full-scale spiking network model. Cereb Cortex. 2014;24:785–806.
4. Bos H, Diesmann M, Helias M. Identifying anatomical origins of coexisting oscillations in the cortical microcircuit. 2015, arXiv preprint arXiv:1510.00642.
5. Brunel N. Dynamics of sparsely connected networks of excitatory and inhibitory spiking neurons. J Comput Neurosci. 2000;8:183–208.

## P148 Spatiotemporal mapping of brain network dynamics during cognitive tasks using magnetoencephalography and deep learning

### Charles M. Welzig^1^, Zachary J. Harper^1,2^

#### ^1^Departments of Neurology and Physiology, Medical College of Wisconsin, Milwaukee, WI 53226, USA; ^2^College of Engineering and Applied Science, University of Wisconsin-Milwaukee, Milwaukee, WI 53211, USA

##### **Correspondence:** Charles M. Welzig - welzig@mcw.edu

*BMC Neuroscience* 2016, **17(Suppl 1)**:P148

**Fig. 78:**
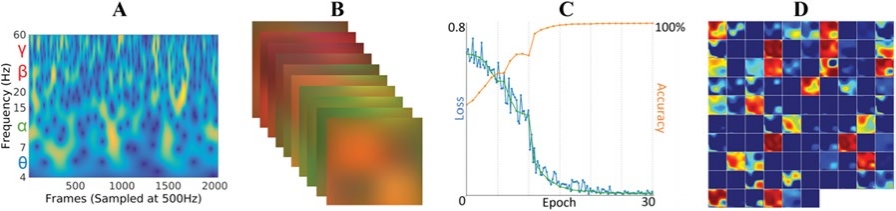
Visualisations of processing and deep learning stages. **A** Wavelet decomposition across bands of interest. **B** Progression of image-encoded oscillatory synchronization in BA10. **C** Deep learning network improvement across training epochs. **D** One trained network layer displaying parcel dynamics that mediate classification

Magnetoencephalography (MEG) offers the high spatiotemporal resolution necessary to capture dynamic mesoscale cortical activation features [1] for spatiotemporal mapping of brain networks. In order to adapt such data for effective pathological or cognitive state classifiers, novel techniques are required to extract complex connectivity dynamics that vary in duration and latency. We have developed an advanced deep-learning system to explore such network dynamics through parcellated connectomes in individual subjects. The machine learning system produces transparent classifiers that can define spatiotemporal characteristics of state-specific connectivity, model neurophysiological pathology and expand understanding of connectivity dynamics. Our implementation uses source localized activation patterns extracted from event related epochs to classify cognitive states corresponding to working memory tasks. Subject-wise MEG data is mapped to segmented morphology from magnetic resonance imaging for source localization, then preprocessed to optimize neural network performance. The following steps minimize dimensionality and accommodate the deep-learning system’s input requirements. First, spatiotemporal data are encoded using a wavelet transformation that extracts oscillatory data in the theta, alpha and beta/gamma bands per parcel (Fig. 78A). Next, synchronicity between parcels is calculated to populate 2D connectivity matrices respective to the frequency bands. These matrices are normalized and combined into frame images where theta, alpha and beta/gamma synchronicity is encoded as blue, green and red intensity values respectively. These pixel grids are smoothed and expanded using gridded, cubic interpolation (Fig. 78B). The deep learning system consists of a recursive neural network utilizing a long-short term memory (LSTM) architecture [2] that preserves temporal input characteristics (Fig. 78C). LSTM presents dynamically changing oscillatory patterns to the deep learning system by integrating features of a specified range of contiguous frames relative to each training frame. As this classification system allows for visualization of activation at each layer, we are able to identify specific patterns that mediate the classification process (Fig. 78D) [3].

Our classification methods have demonstrated significantly low error rate of 0.236 ± 0.425 (mean ± SD) in binary working memory state classification after 3 min of GPU-accelerated training. Additionally, weight patterns at specific layers within the deep learning network highlighted relevant parcel interactions with significant effect on functional connectivity dynamics within classified cognitive states. This project represents an advancement in preserving critical spatiotemporal information required to classify complex cognitive states that characterize dynamically changing oscillatory and synchronous functional activity patterns across the connectome.

**References**

1. Larson-Prior LJ, Oostenveld R, Della Penna S, Michalareas G, Prior F, Babajani-Feremi A, Schoffelen JM et al. Adding dynamics to the human connectome project with MEG. NeuroImage. 2013;80:190–201.
2. Donahue J, Hendricks LA, Guadarrama S, Rohrbach M, Venugopalan S, Saenko K, Darrell T. Long-term recurrent convolutional networks for visual recognition and description; 2014. arXiv:14114389.
3. Plis SM, Hjelm D, Salakhutdinov R, Allen EA, Bockholt HJ, Long JD, Johnson HJ, Paulsen J, Turner JA, Calhoun VD. Deep learning for neuroimaging: a validation study. Front Neurosci. 2014;8.

## P149 Multiscale complexity analysis for the segmentation of MRI images

### Won Sup Kim^1^, In-Seob Shin^1^, Hyeon-Man Baek^2^, Seung Kee Han^1^

#### ^1^Department of Physics, Chungbuk National University, Cheongju, Chungbuk 28644, Republic of Korea; ^2^Korea Basic Science Institute, Cheongju, Chungbuk 28119, Republic of Korea

##### **Correspondence:** Seung Kee Han - skhan@chungbuk.ac.kr

*BMC Neuroscience* 2016, **17(Suppl 1)**:P149

Segmentation of human brain images into regions with homogeneous intensity or texture is very crucial for the diagnosis of various brain diseases. However, the presence of noises or artifacts remains as one of the biggest obstacles for the successful segmentation. Here we propose a novel method of segmentation based on the multiscale complexity analysis. The idea is to characterize the complexity in visual images by the multiscale profile representing the scale dependence of compositional complexity. Our claim is that the multiscale profile of human brain images combining scale dependent information on intensity and texture information could be effectively utilized for the segmentation of human brain images.

We have applied the multiscale complexity analysis for the segmentation of two dimensional MRI images. Our method consists of three steps. (I) An MRI image is partitioned into homogeneous regions utilizing the information bottleneck method. (II) Multiscale complexity profiles of individual pixels are computed from the partitioned image of the MRI. (III) Feature vectors combining both intensity and texture information are extracted for the segmentation. For the segmentation, the feature vectors of individual pixels are clustered using a simple K-mean clustering algorithm.

**Fig. 79:**
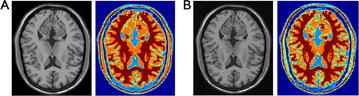
**A** An MRI image from the BrainWeb [1] and the result of segmentation into five clusters. **B** An MRI image with 7 % noise added and the result of segmentation into five clusters

Using the simulated MRI images provided the BrainWeb database [1], the performance of the segmentation was tested. The performance shown in Fig. 79 indicates that the multiscale complexity analysis is very robust against noise. Details will be presented during the meeting.

**Reference**

1. Cocosco C, Kollokian V, Kwan RS, Evan A. BrainWeb: on line interface to a 3D MRI simulated brain database. NeuroImage. 1997;5:S425.

## P150 A neuro-computational model of emotional attention

### René Richter^1^, Julien Vitay^1^, Frederick Beuth^1^, Fred H. Hamker^1,2^

#### ^1^Department of Computer Science, Chemnitz University of Technology, Chemnitz, Germany; ^2^Bernstein Center for Computational Neuroscience, Charité University Medicine, Berlin, Germany

##### **Correspondence:** René Richter - rene.richter@cs.tu-chemnitz.de

*BMC Neuroscience* 2016, **17(Suppl 1)**:P150

Emotional stimuli attract attention so the brain can focus its processing resources on them. The questions that arise is how these stimuli acquire their emotional value and how they can influence attentional processes. Evidence suggests that this association might be learned through conditioning in the amygdala, more specifically the basal lateral amygdala (BLA). Furthermore, feedback connections from the BLA to the visual cortex seem to enhance the activation of neural representations which is a possible top-down attention mechanism of the emergent attention hypothesis. While neuro-computational models of attention mechanisms attract increasing interest due to their importance for the focused processing of information in the brain, the possible emotional feedback from the amygdala is to date largely unexplored. Therefore, we propose a rate-coded, biological realistic neuro-computational model constructed of 3 smaller functional models. First, we combined a model of the visual processing pathway for object recognition [1] that includes the retina, the lateral geniculate nucleus, the visual areas V1, V2 and V4 as well as the frontal eye field with an amygdala model for the associative conditioning of a visual stimulus with a bodily reaction representing a particular emotional state. Second, in order to provide the model with realistic temporal learning properties, a reward-timing model [2] simulating the afferent system to the dopaminergic area VTA has been integrated to temporally adjust the learning process through dopamine-mediated modulation of plasticity. This timing model includes a number of brain areas, most prominently the ventral tegmental area, the nucleus accumbens, the lateral hypothalamus, the ventral medial prefrontal cortex and the amygdala. In order to enable emotional attention, 2 simulation phases were implemented: (1) a conditioning phase to learn the association between an important stimulus and the body reaction, and (2) an attention phase where the representation of the visual stimulus activates the BLA which then sends back a feedback to enhanced this specific stimulus. Afterwards, the enhanced representation in V4 suppresses the competing ones and allows the frontal eye field to initiate a saccade in its direction. As a result of the biologically based connectivity and the realistic learning process, the model outcomes are coherent with several experimental findings and increase our understanding of the brain network’s interaction. In the future, the model could furthermore be used for facial analysis and the process of learning the importance of specific facial features for emotional expressions.

**References**

1. Beuth F, Hamker FH. A mechanistic cortical microcircuit of attention for amplification, normalization and suppression. Vis Res. 2015;116:241–57.
2. Vitay J, Hamker FH. Timing and expectation of reward: a neuro-computational model of the afferents to the ventral tegmental area. Front Neurorobot. 2014;8:1–25.

## P151 Multi-site delayed feedback stimulation in parkinsonian networks

### Kelly Toppin^1^, Yixin Guo^1^

#### ^1^Department of Mathematics, Drexel University, Philadelphia, PA 19104, USA

##### **Correspondence:** Yixin Guo - yixin@math.drexel.edu

*BMC Neuroscience* 2016, **17(Suppl 1)**:P151

**Fig. 80:**
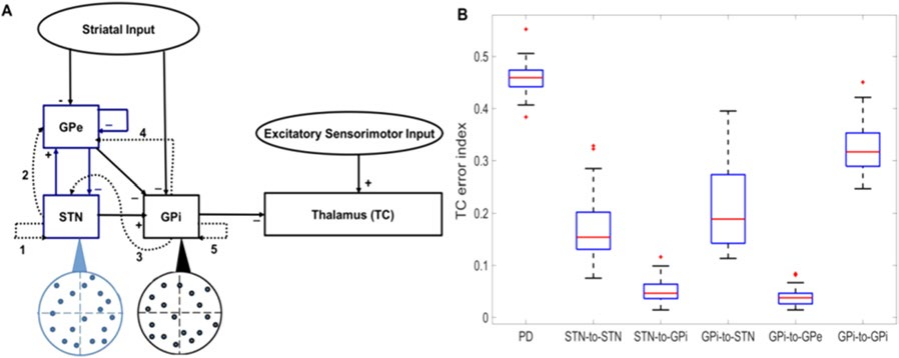
**A** The network model. *Plus symbol* indicates excitatory connection, and *minus symbol* indicates inhibitory connection. LFP is computed from GPi and STN populations separately. These two LFP signals are used as the source of the five MDFS stimulations (*dashed*-*arrows*): STN-to-STN, STN-to-GPi, GPi-to-STN, GPi-to-GPe and GPi-to-GPi, shown by arrow labeled 1–5. **B** Error index values for 80 different model TC neurons in an intermittent network. Comparison of MDFS among different stimulation targets using either STN or GPi LFP signal. *Whisker plots* show mean (*red line*), 25–75 percentile range (*blue box*), 95 % confidence interval (*black lines*) and outliers (*red plus signs*)

The conventional deep brain stimulation (DBS), as a surgical procedure to alleviate debilitating and disrupting symptoms of Parkinson’s disease (PD), has several drawbacks. Multi-site delayed feedback stimulation (MDFS) has been proposed as a feasible alternative to overcome the drawbacks of the conventional DBS [2, 3]. We first build two types of large scale biophysical networks to explore the effectiveness of MDFS. The persistent parkinsonian network has strongly synchronized bursting clusters with elevated firing rates present in subthalmic nucleus (STN), internal and external segments of globus pallidus (GPi and GPe) neurons. However, the brain of a PD patient may not be in a constant strong synchronized and clustered state, and short desynchronized events may present when the brain is in between high synchronization [1]. We build an intermittent parkinsonian network that can transit between synchronized and desynchronized dynamics. Using both parkinsonian networks, we compute the TC error index, the fraction of miss responses and excessive responses when a TC neuron relays multiple excitatory inputs, in five different stimulation settings: MDFS from STN to STN, from STN to GPe, from GPi to STN, from GPi to GPe and from GPi to GPi, shown by the dashed-arrows labeled 1–5 in Fig. 80A. Each “to” population is stimulated by the signal based on the LFP calculated at the “from” population. Our results of lower TC relay errors with the five different stimulations in Fig. 80B show that MDFS improves the fidelity of the TC relay neurons’ communication and responses to the input motor signal in both persistent and intermittent parkinsonian networks. We also find that MDFS with STN or GPe as a stimulation target is more effective in reducing TC relay errors.

**Acknowledgements:** The study was supported by NSF grant DMS-1226180 awarded to Yixin Guo.

**References**

1. Ahn S, Rubchinsky L. Short desynchronization episodes prevail in synchronous dynamics of human brain rhythms. Chaos. 2013;23:013138.
2. Guo Y, Rubin JE. Multi-site stimulation of subthalamic nucleus diminishes thalamocortical relay errors in a biophysical network model. Neural Netw. 2011;24(6):602-16.
3. Hauptmann C, Omel’Chenko O, Popovych V, Maistrenko Y, Tass PA. Control of spatially patterned synchrony with multisite delayed feedback. Phys Rev E. 2007;76(6):066209.

## P152 Bistability in Hodgkin–Huxley-type equations

### Tatiana Kameneva^1^, Hamish Meffin^2^, Anthony N. Burkitt^1^, David B Grayden^1,3^

#### ^1^NeuroEngineering Laboratory, Department of Electrical & Electronic Engineering, University of Melbourne, Parkville, VIC 3010, Australia; ^2^National Vision Research Institute, Australian College of Optometry, Carlton, VIC 3053, Australia; ^3^Centre for Neural Engineering, University of Melbourne, Parkville, VIC 3010, Australia

##### **Correspondence:** René Richter - tkam@unimelb.edu.au

*BMC Neuroscience* 2016, **17(Suppl 1)**:P152

**Background** Purkinje cells have two states of the resting membrane potential: a hyperpolarized quiescent state (down state) and a depolarized spiking state (up state) [1]. This bistability has been observed in in vitro and in vivo recordings, in anesthetized animals, and in slices. It has been proposed that bistability in Purkinje cells play a key role in the short-term processing and storage of sensory-motor information.

**Methods** To investigate bistability of the neuronal resting state, we use computer simulations in neuron. We simulate single compartment neurons and use the Hodgkin–Huxley-type formalism to study how initial conditions and a combination of ionic channels affect neuronal response. We systematically apply intracellular current pulse stimulation to set the membrane potential to different levels and observe the neuronal dynamics after the stimulation is released.

**Fig. 81:**
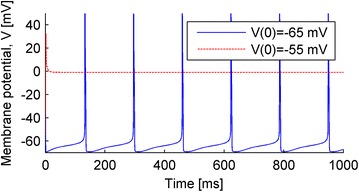
Response of a modeled neuron for different initial conditions, V(0). Sodium, three types of potassium, calcium, and hyperpolarisation-activated currents are included in the model

**Results** We show that the neural response after release of the pulse stimulation depends on the amplitude of the current pulses. For some stimulation levels, the cells return to the level of the activity prior to stimulation, while for other levels, the neuronal dynamics are different to prior activity levels for a long time post stimulation. We show that different initial conditions lead to different neuronal dynamics even when all other parameters in the Hodgkin–Huxley-type model are set the same (Fig. 81). We explore the region of attraction for two stable states and find that they differ for different parameters of the model, in particular that different ionic channel combinations do not change our qualitative results.

**Conclusions** This work demonstrates a potential method to explore the mechanisms underlying bistability in Purkinje cells. In particular, the proposed methodology allows the exploration of the circumstances under which Purkinje cells transit from the down state to the up state and return. This work implies that results obtained using the Hodgkin–Huxley formalism should be carefully considered since the choice of initial conditions may significantly affect the final outcome.

**Acknowledgements:** This research was supported by the Australian Research Council (ARC). TK acknowledge support through ARC Discovery Early Career Researcher Award (DE120102210).

**Reference**

1. Loewenstein Y, Mahon S, Chadderton P, Kitamura K, Sompolinsky H, Yarom Y, Hausser. Bistability of cerebella Purkinje cells modulated by sensory stimulation. Nat Neurosci. 2005;8(2):202–11.

## P153 Phase changes in postsynaptic spiking due to synaptic connectivity and short term plasticity: mathematical analysis of frequency dependency

### Mark D. McDonnell^1^, Bruce P. Graham^2^

#### ^1^Computational and Theoretical Neuroscience Laboratory, School of Information Technology and Mathematical Sciences, University of South Australia, Mawson Lakes, SA, 5095, Australia; ^2^Computing Science and Mathematics, School of Natural Sciences, University of Stirling, Stirling, FK9 4LA, UK

##### **Correspondence:** Mark D. McDonnell - mark.mcdonnell@unisa.edu.au

*BMC Neuroscience* 2016, **17(Suppl 1)**:P153

**Fig. 82:**
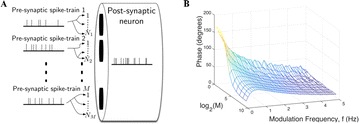
The phase of post-synaptic firing in response to frequency-modulated inhomogenous Poisson pre-synaptic spike trains depends on the configuration of input synapses. **A** The connectivity involves M independent pre-synaptic neurons each with NM synaptic release sites at a post-synaptic neuron. Vesicles are released probabilistically when activated by a pre-synaptic action-potential, if one is available at that site. The post-synaptic neuron depolarizes and produces action potentials after arrival of neurotransmitter according to standard models. **B** The phase lead of output spiking relative to periodic modulation at frequency f, for M active-zones is both frequency-dependent and configuration-dependent. The figure is from simulations but we also derived the same result mathematically

We examined how short-term synaptic depression due to vesicle depletion [1] interacts with the configuration of the synaptic pathways onto an output neuron. Using both simulations and mathematical analysis, we found significant frequency-dependent phase-shifts of the spiking response of a neuron driven by independent frequency-modulated Poisson input signals. The synaptic inputs to the neuron are assumed to consist of a fixed number of release sites that are divided between active zones, with each active zone being the presynaptic axonal target of a single input neuron (Fig. 82A). For the same number of release sites, at one extreme the output neuron receives input from a large number of neurons through independent active zones, each containing a single release site, similar to cortical cells. At the other extreme (similar to a Calyx of Held in the auditory brainstem), the neuron is driven by a single input neuron through a giant synapse containing a single active zone with a very large number of release sites.

Using standard stochastic models of short term depression due to vesicle depletion [2], and post-synaptic current dynamics, we found strong phase dependencies for input modulation rates up to 5 Hz. The phase shift also depends strongly on the configuration (Fig. 82B). However, the phase shift otherwise remains invariant for a wide range of post-synaptic conditions, such as for Hodgkin–Huxley or leaky integrate-and-fire models, and whether or not the dynamics of post synaptic currents included rise-times, or longer or shorter decay times.

**Acknowledgements:** M. D. McDonnell was supported by an Australian Research Fellowship from the Australian Research Council (project DP1093425). B. P. Graham’s contribution was supported by the BBSRC project grant BB/K01854X/1.

**References**

1. Abbott LF, Regehr WG. Synaptic computation. Nature. 2004;431:796–803.
2. McDonnell MD, Mohan A, Stricker C. Mathematical analysis and algorithms for efficiently and accurately implementing stochastic simulations of short-term synaptic depression and facilitation. Front Comput Neurosci. 2013;7:58.

## P154 Quantifying resilience patterns in brain networks: the importance of directionality

### Penelope J. Kale^1^, Leonardo L. Gollo^1^

#### ^1^Systems Neuroscience Group, QIMR Berghofer Medical Research Institute, Brisbane, QLD, 4006, Australia

##### **Correspondence:** Penelope J. Kale - Penelope.Kale@qimr.edu.au

*BMC Neuroscience* 2016, **17(Suppl 1)**:P154

Defining how interactions take place, directionality is major feature of network connections. Brain networks are intrinsically directed because of the nature of chemical synapses, which comprise most of the neuronal connections. The specific fingerprint of the interactions between cortical regions and neurons thereof are crucial to the neuronal dynamics. The neuronal ability to synchronize is extremely sensitive to the presence of reciprocal connections in neuronal motifs and circuits [1]. The type of synchronization (or the phase relation between phase locked neurons and cortical regions) also depends on the relation between the synaptic strengths between these regions [2, 3]. Moreover, whole brain network dynamics is also shaped by reciprocal connections, which stabilizes the network dynamics and reduce transitions between metastable states [4]. However, due to limitations in current brain imaging techniques, the directionality of edges between structurally connected regions of the human brain cannot be confirmed. Additionally, despite the demonstrated importance of synaptic direction, its effect over main network features is not yet elucidated.

Comparing several directed brain networks from different species (macaque, cat, mouse, and *C. elegans*) and with variable node size (parcellation), we estimate the error that is made in characterizing and identifying brains as complex network when undirected networks are assumed. We use different approaches to turn directed networks undirected: (i) remove unidirectional links; (ii) add reciprocal links; (iii) add one reciprocal for each removed unidirectional link thus keeping the same network density. We find that directionality plays a major role in shaping the brain networks. All regions are affected, including hub nodes, which have large degree and enhanced importance in information integration for cognitive functions [5]. We compute and rank graph theoretical measures and determine their resilience with respect to the loss of directionality of the network. Overall, our results suggest that the characterization of connectomes can be compromised in the absence of data regarding the directionality of brain networks.

**References**

1. Gollo LL, Mirasso C, Sporns O, Breakspear M. Mechanisms of zero-lag synchronization in cortical motifs. PLoS Comput Biol. 2014;10(4):e1003548.
2. Matias FS, Carelli PV, Mirasso CR, Copelli M. Anticipated synchronization in a biologically plausible model of neuronal motifs. Phys Rev E. 2011;84(2):021922.
3. Matias FS, Gollo LL, Carelli PV, Bressler SL, Copelli M, Mirasso CR. Modeling positive Granger causality and negative phase lag between cortical areas. NeuroImage. 2014;99:411–8.
4. Gollo LL, Zalesky A, Hutchison RM, van den Heuvel M, Breakspear M. Dwelling quietly in the rich club: brain network determinants of slow cortical fluctuations. Philos Trans R Soc Lond B Biol Sci. 2015;370(1668):20140165.
5. van den Heuvel MP, Sporns O. Network hubs in the human brain. Trends Cogn Sci. 2013;17(12):683–96.

## P155 Dynamics of rate-model networks with separate excitatory and inhibitory populations

### Merav Stern^1^, L.F. Abbott^2^

#### ^1^Faculty of Medicine, Technion, Haifa, Israel; ^2^Department of Neuroscience and Department of Physiology and Cellular Biophysics, Columbia University, New York, NY, USA

##### **Correspondence:** Merav Stern - merav.stern@mail.huji.ac.il

*BMC Neuroscience* 2016, **17(Suppl 1)**:P155

Randomly connected networks of rate-model neurons have a rich dynamics [1], a feature that has been exploited to model a variety of phenomena [2]. These model networks typically do not distinguish between excitatory and inhibitory neuron classes. Doing this requires constraining the network connectivity matrix to have columns with exclusively positive entries, representing input from excitatory neurons, and with negative entries, representing input from inhibitory neurons. The eigenvalue spectra of random matrices satisfying this constraint have a number of interesting properties [3, 4].

Here we study the dynamics of rate-model networks that result from using such connectivity matrices. We find that neural activity is correlated across all neurons, including both excitatory and inhibitory subpopulations. This correlation depends on the difference between the mean strengths of the excitation and inhibition connections and it increases as this difference is increased. For very large values of this difference, the network reaches a stable fixed point, otherwise it is chaotic. Chaos arises from the residual activity deviating from the correlated mean network activity and it acts to reduce these correlations. The magnitude of the residual chaotic activity is determined by the variances of the synaptic strengths within the excitatory and inhibitory populations.

In summary, unlike models with a single mixed excitatory/inhibitory population, in which the activity between pairs of neurons is uncorrelated for every value of synaptic gain, networks with distinct excitatory and inhibitory subpopulations exhibit strongly correlated activity across the entire network reminiscent of the up/down states seen in neural recordings [5].

**References**

1. Sompolinsky H, Crissanti A, Sommers HJ. Chaos in random neural networks. Cerebral Phys Rev Lett. 1988;61:259–62.
2. Sussillo D. Neural circuits as computational dynamical systems. Curr Opin Neurobiol. 2014;25:156–63.
3. Rajan K, Abbott LF. Eigenvalue spectra of random matrices for neural networks. Phys Rev Lett. 2006;97:188104.
4. Tao T. Outliers in the spectrum of IID matrices with bounded rank perturbations. *arXive*: 1012.4818v6.
5. Steriade M, Nunez A, Amzica F. A novel slow (<1 Hz) oscillation of neo-cortical neurons in vivo: depolarizing and hyperpolarizing components. J. Neurosci. 1993;13:3252–65.

## P156 A model for multi-stable dynamics in action recognition modulated by integration of silhouette and shading cues

### Leonid A. Fedorov^1,2^, Martin A Giese^1,2^

#### ^1^Section for Computational Sensomotorics, Department of Cognitive Neurology, CIN&HIH, Tübingen, Germany; ^2^GTC, International Max Planck Research School, University of Tübingen, Tübingen, Germany

##### **Correspondence:** Leonid A. Fedorov - leonid.fedorov@uni-tuebingen.de

*BMC Neuroscience* 2016, **17(Suppl 1)**:P156

**Fig. 83:**
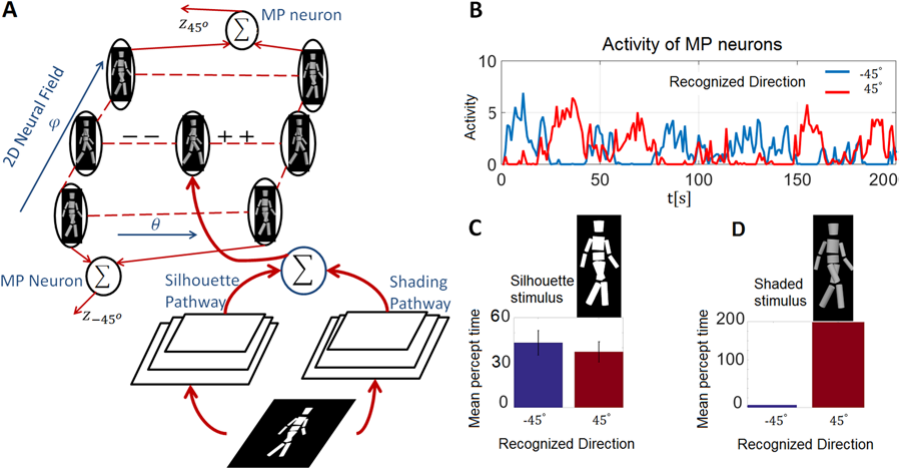
**A** Model architecture with 2D neural field that receives input from two hierarchical path-ways. **B** Response traces of MP neurons for silhouette stimulus without shading during a 200 s simulation. **C** Corresponding average response times of the output neurons. **D** Response times for shaded stimulus

The visual perception of body motion can show interesting multi-stability. For example, a walking body silhouette (bottom inset Fig. 83A) is seen alternately as walking in two different directions [1]. For stimuli with minimal texture information, such as shading, this multi-stability disappears. Existing neural models for body motion perception [2–4] do not reproduce perceptual switching. Extending the model [2], we developed a neurodynamic model that accounts for this multi-stability (Fig. 83A). The core of the model is a two-dimensional neural field that consists of recurrently coupled neurons with selectivity for instantaneous body postures (‘snapshots’). The dimensions of the field encode the keyframe number *θ* and the view of the walker *ϕ*. The lateral connectivity of the field stabilizes two competing traveling pulse solutions that encode the perceived temporally changing action patterns (walking in the directions ±45°). The input activity of the field is generated by two visual pathways that recognize body postures from gray-level input movies. One pathway (‘silhouette pathway’) was adapted from [2] and recognizes shapes, mainly based on the contrast edges between the moving figure and the background. The second pathway is specialized for the analysis of luminance gradients inside the moving figure. Both pathways are hierarchical (deep) architectures, built from detectors that reproduce known properties of cortical neurons. Higher levels of the hierarchies extract more complex features with higher degree of position/scale invariance. The field activity is read out by two Motion Pattern (MP) neurons, which encode the two possible perceived walking directions. Testing the model with an unshaded silhouette stimulus, it produces randomly switching percepts that alternate between the walking directions (±45°) (Fig. 83B, C). Addition of shading cues disambiguates the percept and removes the bistability (Fig. 83D). The developed architecture accounts for the disambiguation by shape-from shading.

**Acknowledgements:** Supported by EC Fp7-PEOPLE-2011-ITN PITN-GA-011-290011 (ABC), FP7-ICT-2013-FET-F/604102 (HBP), FP7-ICT-2013-10/611909 (Koroibot), BMBF, FKZ: 01GQ1002A, DFG GI 305/4-1 + KA 1258/15-1.

**References**

1. Vangeneugden J, et al. Activity in areas MT+ and EBA, but not pSTS, allows prediction of perceptual states during ambiguous biological motion. Soc Neurosci Meet. 2012;127(04).
2. Giese MA, Poggio T. Neural mechanisms for the recognition of biological movements and action. Nat Rev Neurosci. 2003;4:179–92.
3. Lange J, Lappe M. A model of biological motion perception from configural form cues. J Neurosci. 2006;26:2894–906.
4. Jhuang H, et al. A biologically inspired system for action recognition. In: ICCV 2007. p. 1–8.

## P157 Spiking model for the interaction between action recognition and action execution

### Mohammad Hovaidi Ardestani^1,2^, Martin Giese^1^

#### ^1^Section Computational Sensomotorics, CIN & HIH, Department of Cognitive Neurology, Tübingen, 72076, Germany; ^2^IMPRS for Cognitive and Systems Neuroscience, University Clinic Tübingen, Tübingen, 72076, Germany

##### **Correspondence:** Mohammad Hovaidi Ardestani - Mohammad.Hovaidi-Ardestani@uni-tuebingen.de

*BMC Neuroscience* 2016, **17(Suppl 1)**:P157

**Fig. 84:**
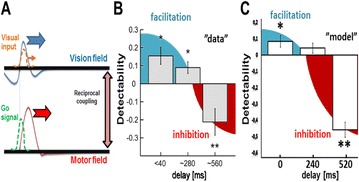
**A** Model architecture consisting of two coupled neural fields, implemented with biophysically realistic neurons. **B** Psychophysical results from [1] showing the dependence of the detectability of visual point-light stimuli in dependence of the delay between a visually observed and the concurrently executed action. **C** Simulated detectability derived from the model for the same experimental conditions

Action perception and the control of action execution are intrinsically linked in the human brain. Experiments show that the concurrent motor execution influences the visual perception of actions and biological motion (e.g. [1]). This interaction likely is mediated by action-selective neurons in the STS, premotor and parietal cortex. We have developed a model based on biophysically realistic spiking neurons that accounts for the observed interactions between action perception and motor planning. The model is based on two dynamic representation levels (Fig. 84A), one modeling a representation of perceived action patters (vision field), and one representing associated motor programs (motor field). Both levels are modeled by recurrent spiking networks that approximate neural fields, where each field consists of 30 coupled neural ensembles, each consisting of 80 excitatory and 20 inhibitory adaptive Exponential Integrate-and-Fire (aEIF) neurons [2]. Within each field asymmetric recurrent connections between the ensembles stabilize a traveling pulse solution, which is stimulus-driven in the visual field and autonomously propagating in the motor field after initiation by a go-signal. Both fields are coupled by interaction kernels that results in mutual excitation between the fields of the traveling pulse propagate synchronously and in mutual inhibition otherwise. We used the model to reproduce the result of a psychophysical experiment that tested the detection of point-light stimuli in noise during concurrent motor execution [1]. The point-light patterns showed arm movements of the observer, which were synchronized with varying time delays with the executed movements. Compared to a baseline without concurrent motor execution, the detectability of the visual stimulus was higher for very small time delays between the visual stimulus and the executed arm movement, and it was lower when the observed movement was strongly delayed (>300 ms) against the executed motor patterns (Fig. 84B). The same pattern arises from the detectability of the visual stimulus as predicted from our model, where we assumed that the level of neural activity (compared to a noise level) provides a measure for the detectability of the stimulus (Fig. 84C). The proposed model, which is derived by simplification from physiologically-inspired neural models for action execution and motor planning, reproduces correctly the modulation of visual detection by the synchrony of the stimulus with executed motor behavior. Present work extends the model by a full visual pathway and an effector model, allowing for the simulation of a broader spectrum of experimental results.

**Acknowledgements:** Supported by EC FP7-ICT-2013-FET-F/604102 (HBP), Fp7-PEOPLE-2011-ITN PITN-GA-011-290011 (ABC), FP7-ICT-2013-10/611909 (Koroibot), BMBF, FKZ: 01GQ1002A, DFG GI 305/4-1 + KA 1258/15-1.

**References**

1. Christensen A, Ilg W, Giese MA. Spatiotemporal tuning of the facilitation of biological motion perception by concurrent motor execution. J Neurosci. 2011;31:3493–9.
2. Brette R, Gerstner W. Adaptive exponential integrate-and-fire model as an effective description of neuronal activity. J Neurophysiol. 2005;94:3637–42.

## P158 Surprise-modulated belief update: how to learn within changing environments?

### Mohammad Javad Faraji^1^, Kerstin Preuschoff^2^, Wulfram Gerstner^1^

#### ^1^School of Life Sciences, Brain Mind Institute and School of Computer and Communication Sciences, Ecole Polytechnique Federal de Lausanne (EPFL), CH-1015 Lausanne, Switzerland; ^2^Geneva Finance Research Institute (GFRI) and Swiss Center for Affective Sciences (CISA), University of Geneva, CH-1211 Geneva, Switzerland

##### **Correspondence:** Mohammad Javad Faraji - mohammadjavad.faraji@epfl.ch

*BMC Neuroscience* 2016, **17(Suppl 1)**:P158

Surprise is informative because it drives attention [1] and modifies learning [2]. Correlates of surprise have been observed at different stages of neural processing, and found to be relevant for learning and memory formation [3]. Although surprise is ubiquitous, there is neither a widely accepted theory that quantitatively links surprise to observed behavior, such as the startle response, nor an agreement on how surprise should influence learning speed or other parameters in iterative statistical learning algorithms. Building on and going beyond earlier surprise measures [4–6], we propose a novel information theoretic measure for calculating surprise in a Bayesian framework so as to capture uncertainty of the world as well as imperfections of the subjective model of the world, two important aspects of surprise. The principle of future surprise minimization leads to a learning rule that can be interpreted as a surprise modulated belief update suitable for learning within changing environments. Importantly, we do not need an assumption on how quickly the world changes. We apply our surprise-modulated learning rule to an exploration task in a maze-like environment. Our results are consistent with the behavioral finding that surprising events induce humans and animals to learn faster and to adapt more quickly to changing environments. Information content [5] captures the inherent unexpectedness of a piece of data for a given set of models (uncertainty of the world), whereas Bayesian surprise [4] measures the change in belief caused by a new data point (observer dependent). These are two complementary approaches for calculating surprise. In our approach both aspects are combined with a third aspect: if we are uncertain about what to expect, receiving a low-probability data sample is less surprising than in a situation when we are almost certain about the world. A surprise minimizing learning (SMiLe) rule is derived by solving a constrained optimization problem defined as follows: the objective is to maximally reduce surprise when facing the same data again in the not so far future, under the constraint that the posterior belief (after the update step) is not too different from the prior. The resulting SMiLe rule balances the influence of newly acquired data with prior knowledge where the balance depends on surprise. In case of a fundamental change in the world signaled by surprising samples, data acquired before the change is downgraded as less informative about the current state of the world. A simultaneous increase of the influence of newly acquired data on learning leads to a fast adaptation of the model to an environmental change. While in a stationary environment our algorithm approaches the known Bayesian update rule, it also allows the model to react to changes in the environment. In summary, surprising data increases the uncertainty we have about our current model of the world and gives a bigger influence of newly acquired data on belief update. The interaction between surprise and uncertainty is important for modeling the behavior of humans and animals in changing environments. The surprise signal could be broadly transmitted in the brain by a neuromodulator with widespread axonal ramifications (e.g., norepinephrine (NE) released from locus coeruleus (LC) neurons) and influence synaptic plasticity rules.

**Acknowledgements:** This research was supported by the European Research Council (Grant Agreement No. 268 689).

**References**

1. Itti L, Baldi P. Bayesian surprise attracts human attention. Vis Res. 2009;49(10):1295–1306.
2. Schultz W, Dickinson A. Neuronal coding of prediction errors. Annu Rev Neurosci. 2000;23(1):473–500.
3. Ranganath C, Rainer G. Neural mechanisms for detecting and remembering novel events. Nat Rev Neurosci. 2003;4(3):193–202.
4. Baldi P, Itti L. Of bits and wows: a bayesian theory of surprise with applications to attention. Neural Netw. 2010;23(5):649–66.
5. Shannon CE. A mathematical theory of communication. ACM SIGMOBILE Mobile Comput Commun Rev. 2001;5(1):3–55.
6. Palm G. Novelty, information and surprise. Berlin: Springer; 2012.

## P159 A fast, stochastic and adaptive model of auditory nerve responses to cochlear implant stimulation

### Margriet J. van Gendt^1^, Jeroen J. Briaire^1^, Randy K. Kalkman^1^, Johan H. M. Frijns^1,2^

#### ^1^ENT-Department, Leiden University Medical Centre, Leiden, 2300 RC, the Netherlands; ^2^Leiden Institute for Brain and Cognition, Leiden, 2300 RC, the Netherlands

##### **Correspondence:** Margriet J. van Gendt - m.j.van_gendt@lumc.nl

*BMC Neuroscience* 2016, **17(Suppl 1)**:P7

Cochlear implants (CI) rehabilitate hearing impairment through direct electrical stimulation of the auditory nerve. In many modern CIs sound is coded through the continuous interleaved sampling (CIS) strategy. Although many different sound-coding strategies have been introduced in the last decade, no major advances have been made since the introduction of the CIS strategy [1]. New stimulation strategies are commonly investigated by means of psychophysical experiments and clinical trials, which is time-consuming for both patient and researcher. Alternatively, strategies can be evaluated using computational models. In this study a computationally efficient model that accurately predicts auditory nerve responses to CI pulse train input is developed.

The model includes the 3D volume conduction and active nerve model developed in the Leiden University Medical Center [2], and is extended with stochasticity, adaptation and accommodation. This complete model includes spatial as well as temporal characteristics of both the cochlea and the auditory nerve. The stochastic and adaptive auditory nerve model is used to investigate full-nerve responses to amplitude modulated long duration stimulation. Understanding responses to amplitude modulation is important because current speech coding strategies are based on the principle of speech information distribution through amplitude modulation of the input pulse trains. The model is validated by comparison to experimentally measured single fiber action potential (SFAP) responses to pulse trains published in literature [3–6]. The effects of different pulse-train parameters such as pulse rate, pulse amplitude and amplitude modulation are investigated.

The neural spike patterns produced in response to CI stimulation are very similar to spike patterns obtained with single fiber action potential measurements in animal experiments in response to CI stimulation. Besides predicting single fiber responses to constant amplitude pulse trains, the model also very well predicts single fiber responses to amplitude modulated pulse trains. Response alterations seen over the duration of the stimulus are similar to those seen in animal experiments. Modeled effects of stimulus amplitude, pulse rate and amplitude modulation is similar to the effects seen in animal experiments. Adaptation is found to be an important factor in modeling nerve outcomes to amplitude modulated pulse trains and their spatial effects.

The model is shown to accurately predict spike timings in response to long duration pulse trains as observed in animal experiments. The model can be used to predict full auditory nerve responses to electrical pulse trains, and thus to different sound coding strategies. The next step will be to apply this model to evaluate complete auditory nerve responses to different sound coding strategies.

**Acknowledgements:** This study was financially supported by Advanced Bionics Corporation.

**References**

1. Zeng FG, Rebscher S, Harrison WV. Cochlear implants: system design, integration and evaluation. IEEE Rev Biomed Eng. 2008;115–42.
2. Kalkman RK, Briaire JJ, Dekker DMT, Frijns JHM. Place pitch versus electrode location in a realistic computational model of the implanted human cochlea. Hear Res. 2014;315:10–24.
3. Miller CA, Hu N, Zhang F, Robinson BK, Abbas PJ. Changes across time in the temporal responses of auditory nerve fibers stimulated by electric pulse trains. J Assoc Res Otolaryngol. 2008;9:122–37.
4. Litvak L, Delgutte B, Eddington D. Auditory nerve fiber responses to electric stimulation: modulated and unmodulated pulse trains. J Acoust Soc Am. 2001;110:368.
5. Zhang F, Miller CA, Robinson BK, Abbas PJ, Hu N. Changes across time in spike rate and spike amplitude of auditory nerve fibers stimulated by electric pulse trains. JARO J Assoc Res Otolaryngol. 2007;8:356–72.
6. Hu N, Miller CA, Abbas PJ, Robinson BK, Woo J. Changes in auditory nerve responses across the duration of sinusoidally amplitude-modulated electric pulse-train stimuli. J Assoc Res Otolaryngol. 2010;11:641–56.

## P160 Quantitative comparison of graph theoretical measures of simulated and empirical functional brain networks

### Won Hee Lee^1^, Sophia Frangou^1^

#### Department of Psychiatry, Icahn School of Medicine at Mount Sinai, New York, NY 10029, USA

##### **Correspondence:** Won Hee Lee - wonhee.lee@mssm.edu

*BMC Neuroscience* 2016, **17(Suppl 1)**:P160

Graph theoretical approaches to resting-state fMRI have been widely used to quantitatively characterize functional network organization in the resting brain, but mechanistic explanations for how resting-state brain works are still lacking. Whole-brain computational models have shown promise in enriching our understanding of mechanisms contributing to the formation and dissolution of resting-state functional patterns [1]. It is therefore important to determine the degree to which computational models reproduce the topological features of empirical functional brain networks. Here, we focused on the performance of the Kuramoto model [2] as it is considered most representative model of coupled phase oscillators and is widely used in the literature.

Empirical and simulated functional networks were defined based on 66 brain anatomical regions (nodes). Simulated resting-state functional connectivity (FC) was generated using the Kuramoto model constrained by empirical structural connectivity. The simulated FC matrix was tuned to best fit empirical FC matrix. In order to improve stability and reliability, we simulated 10 runs of fMRI BOLD time series (obtained from 320 s simulations, discarding 20 s initial transients) with varying random initial conditions, and generated the best-fit simulated FC matrix for each run. We applied graph theoretical approaches to optimally simulated FC and empirical FC data to characterize key topological features of brain networks [3]. Finally, we quantified and compared the difference, in terms of relative error, in graph theoretical measures between the simulated and empirical functional networks.

**Fig. 85:**
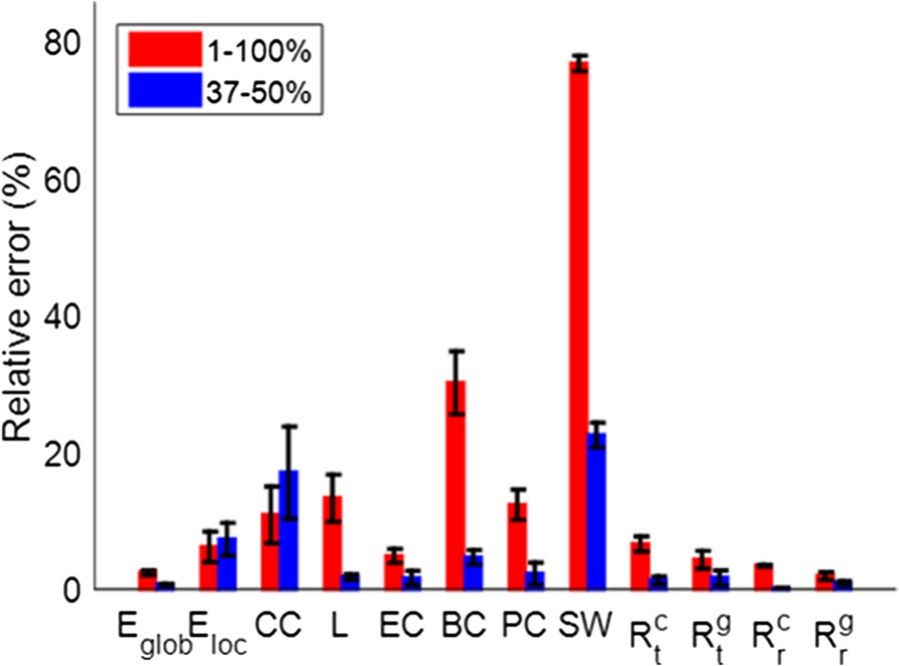
Relative error (RE) in percentage between graph theoretical measures of simulated FC versus empirical FC for the entire (1–100 %) and selected range of connection densities (37–50 %). *Bars* and *error bars* correspond respectively to the averages and standard deviations across the ten RE values. *E*_*glob*_ global efficiency, *E*_*loc*_ local efficiency, *CC* clustering coefficient, *L* characteristic path length, *EC* eigenvector centrality, *PC* participation coefficient, SW: small-worldness, ${\text{R}}_{\text{t}}^{\text{c}}$ and ${\text{R}}_{\text{t}}^{\text{g}}$ represent resilience to targeted attack in the size of largest connected component and global efficiency, respectively, ${\text{R}}_{\text{r}}^{\text{c}}$ and ${\text{R}}_{\text{r}}^{\text{g}}$ represent resilience to random failure in the size of largest connected component and global efficiency, respectively

Figure 85 shows the quantitative difference in graph theoretical measures between the empirical FC and the simulated FC over the entire (1–100 %) and selected range of connection densities (37–50 %). The averaged relative differences were found to be 2–77 % over the entire range of connection densities as well as 0.1–22 % over a range of 37–50 % connection densities. We found that simulated functional data can be used with confidence to model graph measures of global and local efficiency, characteristic path length, eigenvector centrality, and resilience to targeted attack and random failure. Our results also highlight the critical dependence of the solutions obtained in simulated data on the specified connection density.

This study demonstrates the value of computational models in assessing whole-brain network connectivity, and provides a method for the quantitative evaluation and external validation of graph theory metrics derived from simulated data that can be used to inform future study designs.

**References**

1. Deco G, Jirsa VK, McIntosh AR: Emerging concepts for the dynamical organization of resting-state activity in the brain. Nat Rev Neurosci. 2011;12(1):43–56.
2. Kuramoto Y. Chemical oscillations, waves, and turbulence. Berlin: Springer; 1984.
3. Rubinov M, Sporns O. Complex network measures of brain connectivity: uses and interpretations. Neuroimage. 2010l52:1059–69.

## P161 Determining discriminative properties of fMRI signals in schizophrenia using highly comparative time-series analysis

### Ben D. Fulcher^1^, Patricia H. P. Tran^1^, Alex Fornito^1^

#### ^1^Monash Institute of Cognitive and Clinical Neurosciences, Monash University, Clayton, Vic 3168, Australia

##### **Correspondence:** Ben D. Fulcher - ben.fulcher@monash.edu

*BMC Neuroscience* 2016, **17(Suppl 1)**:P161

Analysis of fMRI data typically focuses on inter-regional functional connectivity, measured as pairwise correlations, or through multivariate decompositions (e.g., ICA). Relatively little attention is given to the univariate time-series properties of BOLD signals within a specific brain region, despite a broad scientific literature on time-series analysis (including power spectral techniques, information theoretic methods, model fitting, nonlinear time-series analysis, and fractal scaling). Here we undertake the largest systematic comparison of over 7000 such measures of temporal structure to identify the temporal features of individual BOLD signals, and their locations in the brain, that are most discriminative of people with schizophrenia.

MRI data were obtained from the open *COBRE* database [4] for 72 people with schizophrenia (SCZ) and 74 healthy controls (CON). For each subject, we extracted 7779 temporal features from the BOLD time series recorded in each of 264 brain regions using the publicly available highly comparative time-series analysis framework, *hctsa* (http://benfulcher.github.io/hctsa/) [1].

*Spatial analysis* ROIs that were most discriminative of SCZ versus CON were identified by training a separate linear support vector machine (SVM) classifier for each ROI, across all features, using tenfold cross validation. We identified 23 ROIs with a classification accuracy exceeding chance levels (*p* < 0.05, FDR-corrected) with some individual ROI accuracies reaching 69.5 %. These discriminative brain regions were mostly located in the frontal and parietal cortices.

*Temporal analysis* The most discriminative temporal features were deduced using t-tests in each ROI, and then averaging across all ROIs. P values were computed using permutation tests with 1000 shuffles. We identified over 100 time-series features of the BOLD signal with statistically significant separability between SCZ and CON (p < 0.05). These features were mostly measures of time series ‘predictability’, including autocorrelation, local prediction error (using exponential smoothing, Gaussian Processes, and AR models), the SD1 measure from the heart rate variability literature, and low frequency power. This emergent class of discriminative properties of BOLD dynamics is consistent with the use of the ALFF metric in existing work using fMRI data [3].

We present the first systematic comparison of thousands of interdisciplinary time-series analysis measures to fMRI data and use machine learning to uncover characteristic BOLD signatures of schizophrenia, in both space and time. In a completely data-driven manner, we identify informative brain regions and time-series analysis techniques that best discriminate people with schizophrenia from healthy controls, using just the properties of BOLD signals in individual ROIs. The framework presented here represents a general and powerful data-driven means of identifying discriminative time-series features from neuroscience data.

**References**

1. Fulcher BD, Little MA, Jones NS. Highly comparative time-series analysis: the empirical structure of time series and their methods. J R Soc Interface. 2013;10:20130048.
2. Power JD, Cohen AL, Nelson SM, Wig GS, Barnes KA, Church JA, Vogel AC, Laumann TO, Miezin FM, Schlaggar BL, Petersen SE. Functional network organization of the human brain. Neuron. 2011;72:665–78.
3. Yu-Feng Z, Yong H, Chao-Zhe Z, Qing-Jiu C, Man-Qiu S, Meng L, Li-Xia T, Tian-Zi J, Yu-Feng W. Altered baseline brain activity in children with ADHD revealed by resting-state functional MRI. Brain Dev. 2007;29:83–91.
4. COBRE database. http://fcon_1000.projects.nitrc.org/indi/retro/cobre.html.

## P162 Emergence of narrowband LFP oscillations from completely asynchronous activity during seizures and high-frequency oscillations

### Stephen V. Gliske^1^, William C. Stacey^1,2^, Eugene Lim^3^, Katherine A. Holman^4^, Christian G. Fink^3,5^

#### ^1^Department of Neurology, University of Michigan, Ann Arbor, MI 48104, USA; ^2^Department of Biomedical Engineering, University of Michigan, Ann Arbor, MI 48104, USA; ^3^Department of Physics, Ohio Wesleyan University, Delaware, OH 43015, USA; ^4^Department of Physics, Towson University, Towson, MD 21252, USA; ^5^Neuroscience Program, Ohio Wesleyan University, Delaware, OH 43015, USA

##### **Correspondence:** Christian G. Fink - cgfink@owu.edu

*BMC Neuroscience* 2016, **17(Suppl 1)**:P162

Recent experimental studies have demonstrated the emergence of narrowband local field potential oscillations during epileptic seizures in which the underlying neural activity appears to be completely asynchronous [1]. We derive a mathematical model explaining how this counterintuitive phenomenon may occur, showing that a population of independent, completely asynchronous neurons may produce narrowband oscillations if each neuron fires quasi-periodically. This quasi-periodicity can occur through cells with similar frequency–current (f–I) curves receiving a similar, high amount of uncorrelated synaptic noise. Thus, this source of oscillatory behavior is distinct from the usual cases (pacemaker cells entraining a network, or oscillations being an inherent property of the network structure), as it requires no oscillatory drive nor any specific network or cellular properties other than cells that repetitively fire with continual stimulus.

**Fig. 86:**
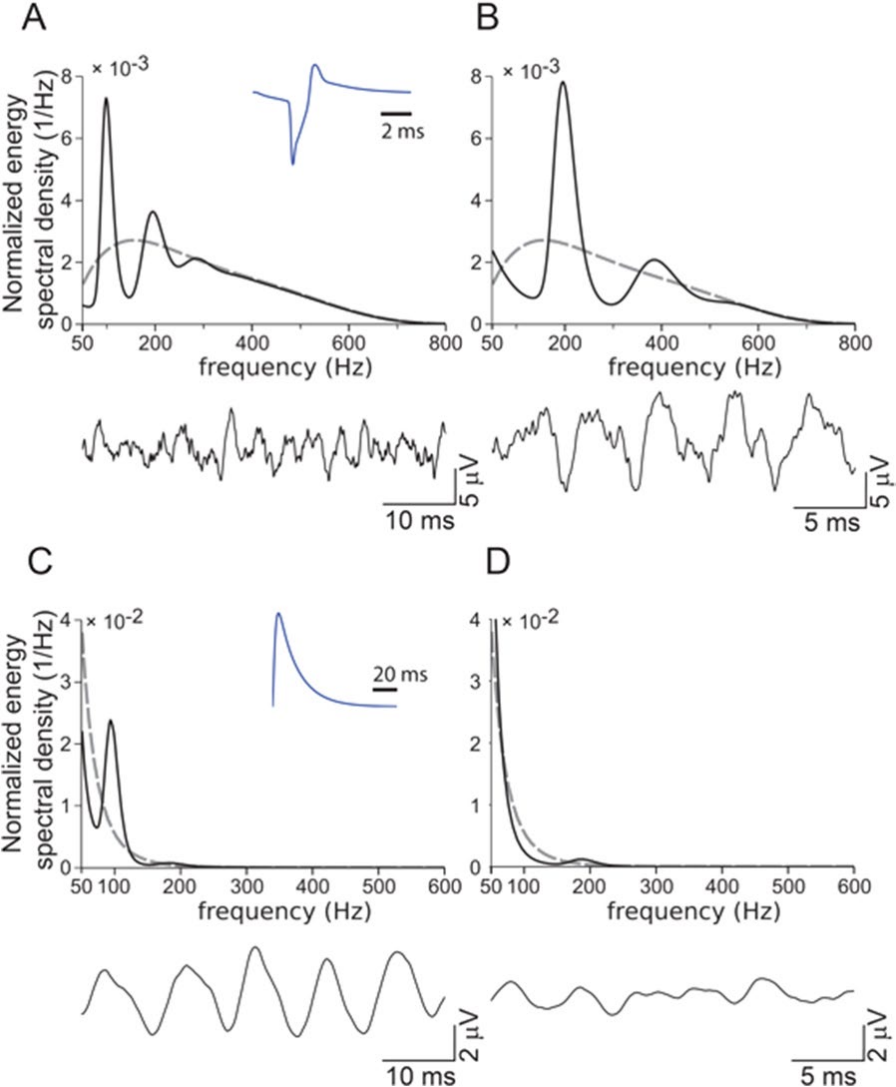
Normalized energy spectra and voltage traces resulting from asynchronous neural activity. **A**, **B** Results of superimposed, asynchronous action potential waveforms for quasi-periodic frequencies of 100 Hz (**A**) and 200 Hz (**B**). **C**, **D** Results of superimposed, asynchronous postsynaptic potential waveforms for quasi-periodic frequencies of 100 Hz (**A**) and 200 Hz (**B**). *Gray dashed lines* represent energy spectra that would result from Poisson process spike trains convolved with AP/PSP waveforms

We deduce bounds on the degree of variability in neural spike-timing which will permit the emergence of such oscillations, both for action potential- and postsynaptic potential-dominated LFPs. (See Fig. 86 for example voltage traces and energy spectra resulting from asynchronous neural activity, demonstrating how our model naturally explains why PSPs tend to dominate the LFP at low frequency, while APs dominate at high frequency.) These results suggest that even an uncoupled network may generate collective rhythms, implying that the breakdown of inhibition and high synaptic input often observed during epileptic seizures may generate narrowband oscillations. We propose that this mechanism may explain why so many disparate epileptic pathologies can produce similar high frequency oscillations [2].

**References**

1. Truccolo W, et al. Neuronal ensemble synchrony during human focal seizures. J Neurosci. 2014;34:9927–44.
2. Engel Jr J, Bragin A, Staba R, Modi I. High-frequency oscillations: what is normal and what is not? Epilepsia. 2009;50:598–604.

## P163 Neuronal diversity in structure and function: cross-validation of anatomical and physiological classification of retinal ganglion cells in the mouse

### Jinseop S. Kim^1,2^, Shang Mu^2^, Kevin L Briggman^3^, H. Sebastian Seung^2,4^, and the EyeWirers^5^

#### ^1^Department of Structure and Function of Neural Networks, Korea Brain Research Institute, Daegu 41068, Republic of Korea; ^2^Princeton Neuroscience Institute, Princeton University, Princeton, NJ 08544, USA; ^3^Circuit Dynamics and Connectivity Unit, National Institute of Neurological Disorders and Stroke, National Institutes of Health, Bethesda, MD 20824, USA; ^4^Computer Science Department, Princeton University, Princeton, NJ 08544, USA; ^5^http://eyewire.org

##### **Correspondence:** Jinseop S. Kim - jinseop.s.kim@kbri.re.kr

*BMC Neuroscience* 2016, **17(Suppl 1)**:P163

The neural computation of visual perception begins in the retina. The retinal neural circuits receive inputs from the photoreceptors, spread out along interneurons, and converge to retinal ganglion cells (RGCs). The axons of RGCs are the only output of the retina and carry all the visual information from the retina to the rest of the brain. Each type of RGCs is thought to be associated with one microcircuit and to process distinct visual information. Therefore, classifying the types is an important step towards understanding the neural computation in the retina and retina’s role in vision [1, 2].

We anatomically classified roughly 400 RGCs based mainly on dendritic stratification profiles [3]. The RGC dendritic arbors were reconstructed from serial electron microscope (EM) images of a (0.3 mm)^2^ slice of the inner plexiform layer of the mouse retina [4]. The reconstruction was carried out on EyeWire, a web-based EM reconstruction pipeline that combines artificial intelligence of deep learning and human intelligence of a community of ‘citizen neuroscientists’ [5]. This is the first time EM reconstruction was done on a large enough area to potentially sample and identify all RGC types.

For cross-validation of the anatomical classification, we compared it with the visual responses of the same cells recorded by calcium imaging performed before EM preparation. The comparison confirmed that our classification recovered all well-known ganglion cell types including on–off direction selective ganglion cells (DSGCs), sustained/transient On DSGCs, asymmetric Off DSGC types, sustained/transient and On/Off alpha cells, and local edge detectors. We also found orientation selective or direction selective responses in some cell types that were not previously well-characterized or were previously unknown. In all, our classification includes over 40 types of RGCs.

**References**

1. Sanes JR, Masland RH. The types of retinal ganglion cells: current status and implications for neuronal classification. Annu Rev Neurosci. 2015;38:221–46.
2. Baden T, Berens P, Franke K, Rosón MR, Bethge M, Euler T. The functional diversity of retinal ganglion cells in the mouse. Nature. 2016;529:345–50.
3. Sümbül U, Song S, McCulloch K, Becker M, Lin B, Sanes JR, Masland RH, Seung HS. A genetic and computational approach to structurally classify neuronal types. Nat Commun. 2014;5:3512.
4. Briggman KL, Helmstaedter M, Denk W. Wiring specificity in the direction-selectivity circuit of the retina. Nature. 2011;471:183–88.
5. Kim JS, Greene MJ, Zlateski A, Lee K, Richardson M, Turaga SC, Purcaro M, Balkam M, Robinson A, Behabadi BF, et al. Space–time wiring specificity supports direction selectivity in the retina. Nature. 2014;509:331–6.

## P164 Analysis and modelling of transient firing rate changes in area MT in response to rapid stimulus feature changes

### Detlef Wegener^1^, Lisa Bohnenkamp^1,2^, Udo A. Ernst^2^

#### ^1^Brain Research Institute, University of Bremen, 28334 Bremen, Germany; ^2^Institute for Neurophysics, University of Bremen, 28334 Bremen, Germany

##### **Correspondence:** Detlef Wegener - wegener@brain.uni-bremen.de

*BMC Neuroscience* 2016, **17(Suppl 1)**:P164

**Fig. 87:**
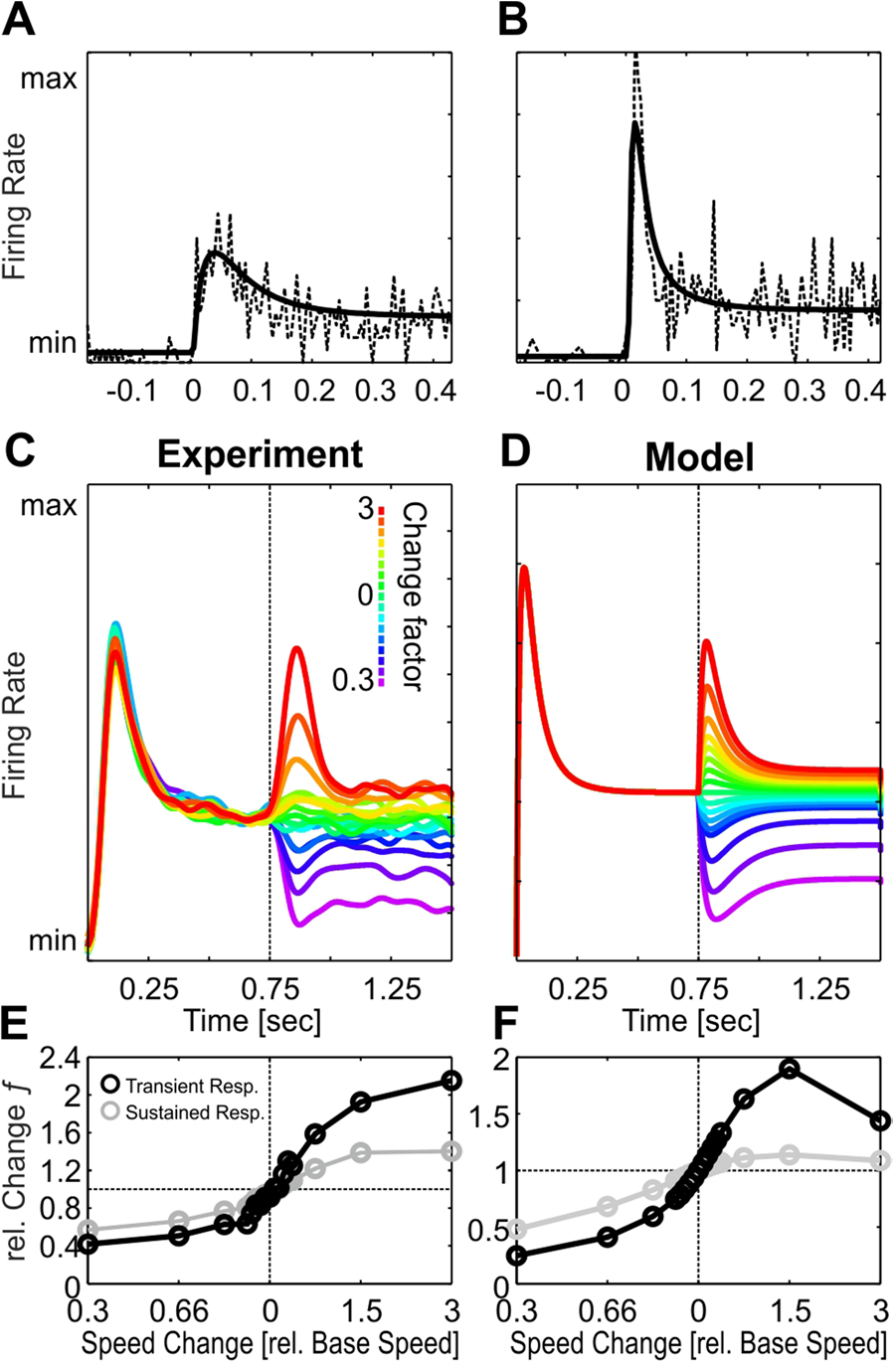
**A**, **B** Fits to motion onset responses to estimate each neuron’s kinetics. **C** Experimentally estimated MT transients to positive and negative speed changes of various magnitude. **D** Transient response amplitudes as derived from the model. **E**, **F** Relation between transient and sustained MT responses as a function of speed change magnitude as estimated experimentally (**E**) and by the model (**F**)

Neurons in area MT of the primate visual system are strongly tuned to the direction and speed of moving stimuli, and they exhibit pronounced transients in their firing rates after changes in visual stimulation. These transients increase the sensitivity of neurons and they are closely correlated to behavioral performance. For example, arbitrary instantaneous speed changes are associated with transients of different sign and amplitude, which closely correlate with the sign and magnitude of the preceding stimulus change and with behavioral performance [1, 2]. Interestingly, the transients’ size cannot be directly referred from the neuron’s underlying speed tuning, and is significantly more pronounced if the base speed before the change is far from the neuron’s preferred speed. Understanding the neural dynamics shaping these responses, and their effects on information transmission of arbitrary time-varying signals, is key to understanding how the visual system copes with dynamic scenes. We here present a dynamical model for MT neurons that reproduces detailed characteristics of experimentally observed transients (Fig. 87). The model takes the single cell’s kinetics and its speed tuning into account. Based on divisive inhibition of excitation, it is capable to reproduce and explain the specific transients of single neurons. Single direction column are made up of one excitatory and one inhibitory population, with the inhibitory population providing divisive inhibition onto the excitatory population. By combining multiple direction columns to one hypercolumn, the model consists of N × 2 populations, with the excitatory populations receiving different input depending on their tuning parameters and the stimulus, and the inhibitory populations receiving an input averaged over the neighboring columns’ input by a Gaussian kernel plus a fixed offset. Using an optimization procedure, the model reliably reproduces MT cell responses to arbitrary accelerations and decelerations of a moving stimulus, starting from both low and high base speeds, reproducing recently unexplained experimental data. If the inhibitory time constant is a multiple of the excitatory time constant, the model is analytically tractable for a piecewise constant input current: The analytical solution allows quantifying the transients’ magnitude as a function of general neuron parameters such as response gain and time constants, providing precise predictions for population responses to brief events of arbitrary contrast.

**References**

1. Galashan FO, Saßen HC, Kreiter AK, Wegener D. Monkey area MT latencies to speed changes depend on attention and correlate with behavioral reaction times. Neuron. 2013;78(4):740–50.
2. Traschütz A, Kreiter AK, Wegener D: Transient activity in monkey area MT represents speed changes ind is correlated with human behavioral performance. *J Neurophysiol* 2015, 113(3):890-903.

## P165 Step-wise model fitting accounting for high-resolution spatial measurements: construction of a layer V pyramidal cell model with reduced morphology

### Tuomo Mäki-Marttunen^1^, Geir Halnes^2^, Anna Devor^3,4^, Christoph Metzner^5^, Anders M. Dale^3,4^, Ole A. Andreassen^1^, Gaute T. Einevoll^2,6^

#### ^1^NORMENT, Institute of Clinical Medicine, University of Oslo, Norway; ^2^Department of Mathematical Sciences and Technology, Norwegian University of Life Sciences, Ås, Norway; ^3^Department of Neurosciences, University of California San Diego, La Jolla, CA, USA; ^4^Department of Radiology, University of California San Diego, La Jolla, CA, USA; ^5^Biocomputation Research Group, University of Hertfordshire, Hatfield, UK; ^6^Department of Physics, University of Oslo, Norway

##### **Correspondence:** Tuomo Mäki-Marttunen - tuomomm@uio.no

*BMC Neuroscience* 2016, **17(Suppl 1)**:P165

Novel imaging methods such as intracellular Ca^2+^ imaging and voltage-sensitive dye measurements provide ever finer spatiotemporal data about single-neuron activity. The challenge for model fitting methods is to incorporate these data in order to describe the neuron behavior in a manner that faithfully preserves the signal propagation and membrane potential dynamics across the neuronal dendrites. A difficulty in this task is the evidently large number of different ion channels residing along the dendritic and perisomatic locations: Unless extra care is taken, the role of specific species of ion channel could be under- or overestimated at the expense of another type of ion channel.

In this work, we propose an automatic step-wise model fitting procedure as a solution to this challenge. Our approach resembles that of [1], but our objective functions are designed to account for correct membrane potentials not only at soma but also along the dendrites. In addition, we replace the need for spatial occlusion of parts of dendrite (“pinching”) [2] in the experimental setup by a cumulative use of ion channel blockers.

We apply this procedure to construct a reduced-morphology version of the layer V pyramidal cell model of [3]. We simulated the cumulative blocking of ion channels by setting the corresponding ion channel conductances to zero in the full model, and measured the membrane potential (and Ca^2+^ concentrations when needed) along the soma and dendrites at each step. We then fitted the maximal conductances in the model with reduced morphology in four steps, starting with passive parameters (1st step), continuing with *I*_*h*_ current conductances (2nd step), Ca^2+^ dynamics and related conductances (3rd step), and ending with ion channel conductances that are in charge of the spiking behavior (4th step).

**Fig. 88:**
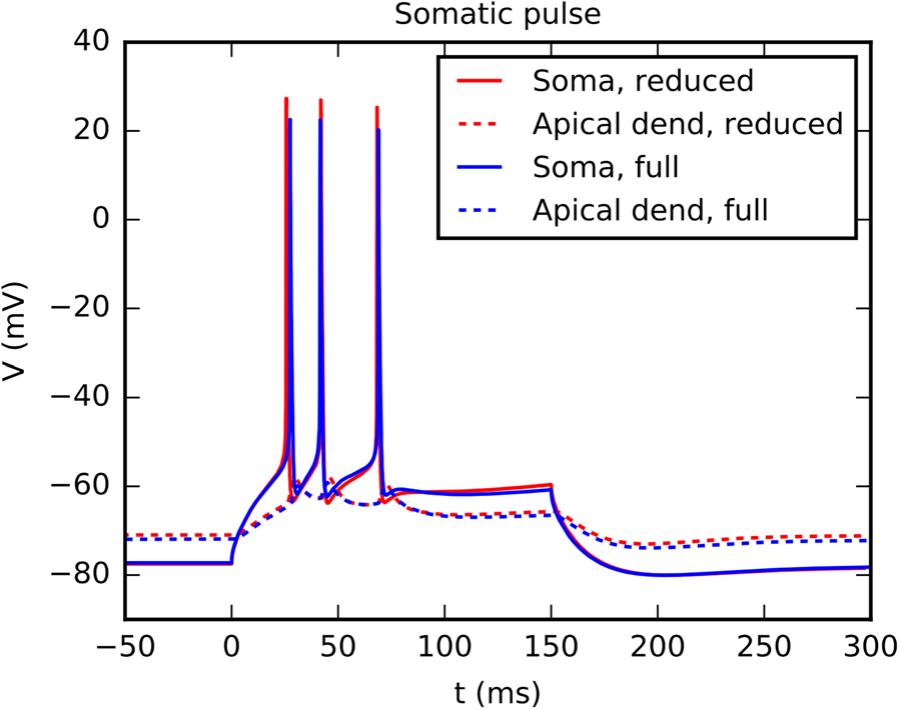
Comparison of model with reduced (*red*) morphology to the model with full (*blue*) morphology. The y-axis shows the membrane potential at soma (*solid*) and apical dendrite (*dashed*) as a response to a somatic 200-ms DC pulse

We show that our model with reduced morphology correctly reproduces important aspects of the membrane potential dynamics across the neuron, both in the control condition (see Fig. 88), and under the effect of the abovementioned ion channel blockers. In the final step of our study, we present and apply a method for reducing the number of synaptic contacts (from 1000s to a few 100s) yet maintaining the spatio-temporal activation pattern of the neuron. The obtained network model is cost-efficient in terms of both simulation time and memory requirements. Our model is publicly accessible in ModelDB, accession number 187474, as NEURON and NeuroML-2 descriptions (https://senselab.med.yale.edu/ModelDB/showModel.cshtml?model=187474).

**References**

1. Bahl A, Stemmler MB, Herz AV, Roth A. Automated optimization of a reduced layer 5 pyramidal cell model based on experimental data. J Neurosci Methods. 2012;210(1):22–34.
2. Bekkers JM, Häusser M. Targeted dendrotomy reveals active and passive contributions of the dendritic tree to synaptic integration and neuronal output. Proc Natl Acad Sci. 2007;104(27):11447–52.
3. Hay E, Hill S, Schürmann F, Markram H, Segev I. Models of neocortical layer 5b pyramidal cells capturing a wide range of dendritic and perisomatic active properties. PLoS Comput Biol. 2011;7:e1002107.

## P166 Contributions of schizophrenia-associated genes to neuron firing and cardiac pacemaking: a polygenic modeling approach

### Tuomo Mäki-Marttunen^1^, Glenn T. Lines^2^, Andy Edwards^2^, Aslak Tveito^2^, Anders M. Dale^3^, Gaute T. Einevoll^4^, Ole A. Andreassen^1^

#### ^1^NORMENT, Institute of Clinical Medicine, University of Oslo, Norway; ^2^Simula Research Laboratory and Center for Cardiological Innovation, Oslo, Norway; ^3^Multimodal Imaging Laboratory, UC San Diego, La Jolla, CA, USA; ^4^Department of Mathematical Sciences and Technology, Norwegian University of Life Sciences, Ås, Norway

##### **Correspondence:** Tuomo Mäki-Marttunen - tuomomm@uio.no

*BMC Neuroscience* 2016, **17(Suppl 1)**:P166

A recent genome-wide association study (GWAS) of schizophrenia (SCZ) has identified more than a hundred genetic loci exceeding genome-wide significance, confirming the polygenic nature of the disorder [1]. The loci implicate genes that encode numerous ion channel subtypes and calcium transporters, and are major contributors not only to the function of brain cells, but also to the functioning of organs outside the central nervous system, such as heart. Meta-studies have reported a 2.5-fold–threefold increase in mortality rates in schizophrenic patients, and majority of these excess deaths are natural and mostly due to cardiovascular disease [2]. In agreement with this observation, GWASs of cardiac phenotypes, such as electrocardiographic (ECG) measures, highlight a set of genes that overlaps with the one discovered in GWASs of SCZ. Nevertheless, both the genetic and mechanistic connections between cardiac and neural phenotypes in SCZ patients remain poorly understood.

In this work, we use computational modeling to study the contribution of SCZ-associated genes to cardiac and neuronal excitability. We focus our analyses on two central, well-studied cell types, namely, layer V pyramidal cells (L5PCs) in the cortex and sinoatrial node cells (SANCs) in the myocardium. The apical tuft of an L5PC serves as an integration hub for non-local synaptic inputs, and is considered a biological substrate for cortical associations providing high-level “context” for low-level (e.g., sensory) inputs that arrive to the perisomatic compartment. Therefore, the ability of L5PC to integrate the apical and perisomatic inputs has been proposed as one of the mechanisms that could be impaired in hallucinating patients. The SANCs, in turn, have a key role in controlling the heart rate as the primary pacemakers of the mammalian heart. Both of these cell types are well described in terms of biophysical modeling, and are therefore a suitable target for a detailed computational studies incorporating genetic effects. We apply two recent multicompartmental L5PC models and two recent SANC models to argue for the generality of our findings.

We show that small changes in the parameters governing the voltage-dependence and time constants of activation and inactivation of different ion channels caused observable effects in both L5PC and SANC function. In the case of Ca^2+^ channel gene variants, these changes typically had opposite effects on cell excitability in L5PCs compared to SANCs (higher L5PC firing frequency ↔ lower SANC pacemaking frequency), while in the case of Na^+^ or HCN channel variants, the effects were mostly similar (higher L5PC firing frequency ↔ higher SANC pacemaking frequency). Furthermore, many of the studied variants showed an impact on signal propagation in a chain of coupled SANCs. Our results may help explain some of the cardiac comorbidity in schizophrenia, and may facilitate generation of effective antipsychotic medications with less arrythmia side-effects.

**References**

1. Ripke S, Sanders AR, Kendler KS, Levinson DF, Sklar P, Holmans PA, Lin DY, Duan J, Ophoff RA, Andreassen OA et al.: Genome-wide association study identifies five new schizophrenia loci. Nat Gen. 2011;43:969–76.
2. Laursen TM, Munk-Olsen T, Vestergaard M. Life expectancy and cardiovascular mortality in persons with schizophrenia. Curr Opin Psychiatry. 2012;25(2):83–8.
3. Hay E, Hill S, Schürmann F, Markram H, Segev I: models of neocortical layer 5b pyramidal cells capturing a wide range of dendritic and perisomatic active properties. PLoS Comput Biol. 2011;7:e1002107.
4. Almog M, Korngreen A. A quantitative description of dendritic conductances and its application to dendritic excitation in layer 5 pyramidal neurons. J Neurosci. 2014;34(1):182–96.
5. Kharche S, Yu J, Lei M, Zhang H. A mathematical model of action potentials of mouse sinoatrial node cells with molecular bases. Am J Physiol Heart Circ Physiol. 2011;301(3):H945–63.
6. Severi S, Fantini M, Charawi LA, DiFrancesco D. An updated computational model of rabbit sinoatrial action potential to investigate the mechanisms of heart rate modulation. J Physiol. 2012;590(18):4483–99.

## P167 Local field potentials in a 4 × 4 mm^2^ multi-layered network model

### Espen Hagen^1^, Johanna Senk^1^, Sacha J van Albada^1^, Markus Diesmann^1,2,3^

#### ^1^Institute of Neuroscience and Medicine (INM-6) and Institute for Advanced Simulation (IAS-6) and JARA BRAIN Institute I, Jülich Research Centre, Jülich, 52425, Germany; ^2^Department of Psychiatry, Psychotherapy and Psychosomatics, Medical Faculty, RWTH Aachen University, Aachen, 52074, Germany; ^3^Department of Physics, Faculty 1, RWTH Aachen University, Aachen, 52074, Germany

##### **Correspondence:** Espen Hagen - e.hagen@fz-juelich.de

*BMC Neuroscience* 2016, **17(Suppl 1)**:P167

**Fig. 89:**
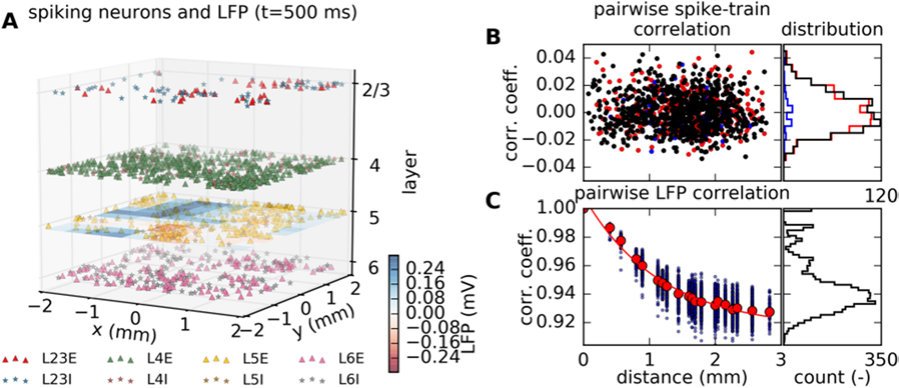
**A** Instantaneous spiking and LFP in a 4-layer network model covering 4 × 4 mm^2^ at realistic cell and synapse density with distance-dependent connectivity. **B** Pairwise correlations between spike trains of exc. (E) and inh. (I) layer 5 neurons as function of distance (*red*: E–E, *blue*: I–I, *black*: E–I). **C** Distance-dependent LFP correlation computed for a 10 × 10 electrode grid in layer 5 (0.4 mm between contacts)

The local field potential (LFP), the low-frequency part of extracellular potentials in neural tissue, is routinely recorded as a measure of population activity. LFPs reflect correlated activity of both local and remote neurons and depend on the anatomy and electrophysiology of neurons near the recording location. While forward models have shed light on various aspects of LFPs, e.g., their spatial reach [1], such models often ignore network interactions. Large-scale network models commonly use point neurons for tractability (see, e.g., [2]). However, predicting the LFP signal from such models is not straightforward, as point neurons do not generate extracellular potentials. In [3] we provided methods to compute extracellular potentials from point-neuron networks incorporating the biophysical principles of LFP generation using multicompartment neurons. This hybrid scheme uses spike times of point neurons as spatially dependent synaptic input with layer specificity of connections from anatomical data. The methods were demonstrated using a laterally homogeneous, layered point-neuron network representing 1 mm^2^ of early sensory cortex at full cell and synapse density [4]. Preserving biological cell and connection densities is critical: networks may not be strongly downscaled without affecting correlations [5], and diluted LFP-generating populations fail to preserve the effect of correlations on the LFP [3]. Even small network correlations dominate in the compound LFP spectrum due to the different scaling of average single-cell LFP spectra and average pairwise coherence of single-cell LFP. Here, we extend this work to a network covering 4 × 4 mm^2^ (Fig. 89A) accounting for connection probabilities falling off with lateral distance. Even for low pairwise spike-train correlations (Fig. 89B), the model accounts for highly correlated LFPs across lateral distance (Fig. 89C) as observed experimentally. Further we show that such features strongly depend on network state.

**Acknowledgements:** EU FP7 grant 604102 (HBP); Helmholtz Portfolio Supercomputing and Modeling for the Human Brain (SMHB).

**References**

1. Lindén H, Tetzlaff T, Potjans TC, Pettersen KH, Gruen S, Diesmann M, Einevoll GT. Modeling the spatial each of the LFP. Neuron. 2011;72:859–72. 
2. Bos H, Diesmann M, Helias M. Identifying anatomical origins of coexisting oscillations in the cortical microcircuit. arXiv:1510.00642 [q-bio.NC] 2016.
3. Hagen E, Dahmen D, Stavrinou ML, Lindén H, Tetzlaff T, van Albada SJ, Grün S, Diesmann M, Einevoll GT. Hybrid scheme for modeling local field potentials from point-neuron networks. arXiv:1511.01681 [q-bio.NC] 2016. http://inm-6.github.io/hybridLFPy.
4. Potjans TC, Diesmann M. The cell-type specific cortical microcircuit: relating structure and activity in a full-scale spiking network model. Cereb Cortex. 2014;24:785–806.
5. Van Albada SJ, Helias M, Diesmann M. Scalability of asynchronous networks is limited by one-to-one mapping between effective connectivity and correlations. PLoS Comput Biol. 2015;11(9):e1004490.

## P168 A spiking network model explains multi-scale properties of cortical dynamics

### Maximilian Schmidt^1^, Rembrandt Bakker^1,2^, Kelly Shen^3^, Gleb Bezgin^4^, Claus-Christian Hilgetag^5,6^, Markus Diesmann^1,7,8^, Sacha Jennifer van Albada^1^

#### ^1^Institute of Neuroscience and Medicine (INM-6) and Institute for Advanced Simulation (IAS-6), and JARA BRAIN Institute I, Jülich Research Centre, Jülich, Germany; ^2^Donders Institute for Brain, Cognition and Behavior, Radboud University, Nijmegen, Netherlands; ^3^Rotman Research Institute, Baycrest, Toronto, Ontario M6A 2E1, Canada; ^4^McConnell Brain Imaging Centre, Montreal Neurological Institute, McGill University, Montreal, Canada; ^5^Department of Computational Neuroscience, University Medical Center Eppendorf, Hamburg, Germany; ^6^Department of Health Sciences, Boston University, Boston, MA, USA; ^7^Department of Psychiatry, Psychotherapy and Psychosomatics, Medical Faculty, RWTH Aachen University, Aachen, Germany; ^8^Department of Physics, Faculty 1, RWTH Aachen University, Aachen, Germany

##### **Correspondence:** Maximilian Schmidt - max.schmidt@fz-juelich.de

*BMC Neuroscience* 2016, **17(Suppl 1)**:P168

Neural networks in visual cortex are structured into areas, layers, and neuronal populations with specific connectivity at each level. Cortical dynamics can similarly be characterized on different scales, from single-cell spiking statistics to the structured patterns of interactions between areas. A challenge of computational neuroscience is to investigate the relation of the structure of cortex to its dynamics. Network models are promising tools, but for technical and methodological reasons, they have been restricted to detailed models of one or two areas or large-scale models that reduce the internal structure of areas to a small number of differential equations.

We here present a multi-scale spiking network model of all vision-related areas of macaque cortex that represents each area by a full-scale microcircuit with area-specific architecture based on a model of early sensory cortex [1]. The layer- and population-resolved network connectivity integrates axonal tracing data from the CoCoMac database with recent quantitative tracing data, and is systematically refined using dynamical constraints [2]. Gaps in the data are bridged by exploiting regularities of cortical structure such as the exponential decay of connection densities with inter-areal distance and a fit of laminar patterns versus logarithmized ratios of neuron densities.

Simulations reveal a stable asynchronous irregular ground state with heterogeneous activity across areas, layers, and populations. In the presence of large-scale interactions, the model reproduces longer intrinsic time scales in higher compared to early visual areas, similar to experimental findings [3]. Activity propagates preferentially in the feedback direction, mimicking experimental results associated with visual imagery [4]. Cortico-cortical interaction patterns agree well with fMRI resting-state functional connectivity [5]. The model bridges the gap between local and large-scale accounts of cortex, and clarifies how the detailed connectivity of cortex shapes its dynamics on multiple scales.

**Acknowledgements:** VSR computation time Grant JINB33, Helmholtz Portfolio SMHB, EU Grant 269921 (BrainScaleS), EU Grant 604102 (Human Brain Project, HBP), SFB936/A1, Z1 and TRR 169/A2.

**References**

1. Potjans TC, Diesmann M. The cell-type specific cortical microcircuit: relating structure and activity in a full-scale spiking network model. Cereb Cortex. 2014;24:785–806.
2. Schuecker J, Schmidt M, van Albada SJ, Diesmann M, Helias M. Fundamental activity constraints lead to specific interpretations of the connectome. arXiv preprint 2015, arXiv:1509.03162.
3. Murray JD, Bernacchia A, Freedman DJ, Romo R, Wallis JD, Cai X, Padoa-Schioppa C, Pasternak T, Seo H, Lee D, Wang X-J. A hierarchy of intrinsic timescales across primate cortex. Nat Neurosci. 2014;17:1661–3.
4. Dentico D, Cheung BL, Chang J-Y, Guokas J, Boly M, Tononi G, Van Veen B. Reversal of cortical information flow during visual imagery as compared to visual perception. Neuroimage. 2014;100:237–243.
5. Shen K, Bezgin G, Hutchison RM, Gati JS, Menon RS, Everling S, McIntosh AR. Information processing architecture of functionally defined clusters in the macaque cortex. J Neurosci. 2012;32:17465–76.

## P169 Using joint weight-delay spike-timing dependent plasticity to find polychronous neuronal groups

### Haoqi Sun^1,2,3,5^, Olga Sourina^2,5^, Guang-Bin Huang^3,5^, Felix Klanner^4,5^, Cornelia Denk^5^

#### ^1^Energy Research Institute @ NTU (ERI@N), Interdisciplinary Graduate School, Nanyang Technological University, Singapore 639798; ^2^Fraunhofer IDM @ NTU, Nanyang Technological University, Singapore 639798; ^3^School of Electrical and Electronic Engineering, Nanyang Technological University, Singapore 639798; ^4^School of Computer Engineering, Nanyang Technological University, Singapore 639798; ^5^Future Mobility Research Lab, A Joint Initiative of BMW Group & NTU, Nanyang Technological University, Singapore 639798

##### **Correspondence:** Haoqi Sun - hsun004@e.ntu.edu.sg

*BMC Neuroscience* 2016, **17(Suppl 1)**:P169

It is known that polychronous neuronal groups (PNGs), i.e. neuron groups having reproducible time-locked but not synchronous firing patterns, can function as representative entities [1]. They have huge capacity by sharing neurons. They compete between each other to represent sensory inputs. Therefore, PNG is considered as one of the potential, yet elusively difficult to analyze, hypothetical mechanisms of memory in the brain.

In computational models, the difficulties of finding PNGs mainly come from (1) low percentage of spikes from PNGs (about 4 % [1]) when driven by random inputs; and (2) combination explosion to enumerate all possible PNGs for template-matching (possible PNGs triggered by 3 neurons in a 1000-neuron network is 3C1000 = 1.66 × 10^8^).

Here we aim at solving the second difficulty without template-matching by connecting PNG readout neurons with joint weight-delay spike-timing dependent plasticity (joint STDP) to the network. The joint STDP consists of (1) weight STDP with the conventional exponential learning window; and (2) (axonal) delay STDP with learning window of shape te^−t/τ^, scaled by weight-related gains. The joint STDP strengthens and pulls together spikes arriving before postsynaptic firing, on the other hand weakens and postpones spikes after postsynaptic firing. In this way, we can recover the PNG by looking at (1) the strengthened synapses, which tells which neurons belong to the PNG; and (2) the delays of the strengthened synapses, which are complementary to the spike timing inside the PNG, because the presynaptic spike arrival times for the readout neuron (=spike timing + delay) are pulled close to each other.

**Fig. 90:**
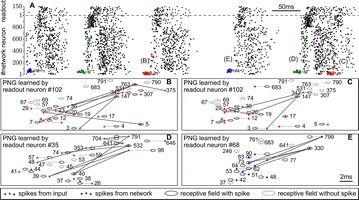
**A** The spike raster plot showing 0.6 s of simulation. The *vertical axis* shows neuron index. Neurons from index 1 to 100 receive structured inputs. *Colored spikes* refer to PNGs founded by the same *colored* spikes of readout neurons (above the *dash line*), where the letter-marked ones are shown in *other panels*. **B** A recovered PNG with the predicted spike timing (receptive field) and the actual spikes. The numbers are neuron indices. **C** The same PNG in **B** but activated at another time. **D**, **E** Another PNG

In the experiment, we repeatedly fed structured inputs to a sparsely connected network of 800 excitatory and 200 inhibitory neurons. There were 150 readout neurons connected to the network with lateral inhibition between them. After 405 s of simulation, we used the incoming weights and delays of the readout neurons to find PNGs (see Fig. 90). It turned out that the readout neurons can learn the subsets of the persistently activated PNGs. The readout neurons do not rely on template-matching. Instead, they become differentiated members of the PNG to indicate the actual activation of its subsets.

**Reference**

1. Izhikevich EM. Polychronization: computation with spikes. Neural Comput. 2006;18(2):245–82.

## P170 Tensor decomposition reveals RSNs in simulated resting state fMRI

### Katharina Glomb^1^, Adrián Ponce-Alvarez^1^, Matthieu Gilson^1^, Petra Ritter^2,3,4,5^, Gustavo Deco^1,6^

#### ^1^Center for Brain and Cognition, Universitat Pompeu Fabra, 08018 Barcelona, Spain; ^2^Minerva Research Group Brain Modes, Max Planck Institute for Human Cognitive and Brain Sciences, 04103 Leipzig, Germany; ^3^Department of Neurology, Charité - University Medicine, 10117 Berlin, Germany; ^4^Bernstein Focus State Dependencies of Learning & Bernstein Center for Computational Neuroscience, 10115 Berlin, Germany; ^5^Berlin School of Mind and Brain & Mind and Brain Institute, Humboldt University, 10117 Berlin, Germany; ^6^Catalan Institution for Advanced Studies (ICREA), Universitat Barcelona, 08010 Barcelona, Spain

##### **Correspondence**: Won Hee Lee - katharina.glomb@upf.edu

*BMC Neuroscience* 2016, **17(Suppl 1)**:P170

The subject of this study is the temporal dynamics of functional connectivity (FC) in human resting state (RS) as measured with BOLD fMRI. In spite of rising interest in the topic [1], it remains unclear whether observed FC is stationary or if state switching is present, nor is it clear what constitutes these putative states. Modelling is an invaluable tool for answering these questions: here we combine a dynamic mean field model of the cortex with data analysis in order to determine whether and to what extent spatio-temporal FC patterns found in empirical data can be mimicked by a stationary model as described in [2]. To this end, we cast our data into tensor form by computing time-dependent FC inside of sliding windows (dynamic FC, dFC), comparing three methods to compute dFC (two correlation based, and mutual information). We employ canonical polyadic decomposition (also known as parallel factor analysis) with or without non-negativity constraint to decompose the tensors, which allows us to simultaneously consider the temporal and spatial dimensions [3]. First, we decompose such tensors obtained from empirical data of 24 subjects [4] and cluster resulting spatial features (i.e., communities) in order to obtain a small number of templates. These templates are used in a second step to compare to simulated data that is processed in the same way. We find that even on a very low level of spatial resolution (66 cortical regions), and using only the 2 % biggest dFC values in terms of region pairs and time windows, we succeed in extracting communities that generalize across subjects and can be found in the simulated data. Furthermore, we show that using model-based effective connectivity to inform the model [5] leads to more realistic and stable communities than diffusion weighted MRI-based structural connectivity alone. The method shown here is widely applicable to compare patient groups, data obtained from different tasks as well as mental states, and opens the door to understanding the differences between the temporal dynamics of these conditions.

**Acknowledgements:** KG is funded by the Marie Curie Initial Training Network INDIREA, grant agreement no ITN-2013-606901. APA was supported by SEMAINE ERA-Net NEURON Project. MG acknowledges funding from FP7 FET ICT Flagship Human Brain Project (604102). GD was supported by the ERC Advanced Human Brain Project (n. 604102) and the Plan Estatal de Fomento de la investigación Científica y Técnica de Excelencia (PSI2013-42091-P).

**References**

1. Hutchison RM, Womelsdorf T, Allen EA, Bandettini PA, Calhoun VD, Corbetta M, Della Penna S, Duyn JH, Glover GH, Gonzalez-Castillo J, Handwerker DA, Keilholz S, Kiviniemi V, Leopold DA, de Pasquale F, Sporns O, Walter M, Chang C. Dynamic functional connectivity: promise, issues, and interpretations. NeuroImage. 2013;80:360–78.
2. Deco G, Ponce-Alvarez A, Hagmann P, Romani G, Mantini D, Corbetta M. How local excitation–inhibition ratio impacts the whole brain dynamics. *J Neurosci.* 2014;34(23):7886–98.
3. Cichocki A. Tensor decompositions: a new concept in brain data analysis? *arxiv Prepr.* 2013, arXiv1305.0395, 507–17.
4. Schirner M, Rothmeier S, Jirsa VK, McIntosh AR, Ritter P. An automated pipeline for constructing personalized virtual brains from multimodal neuroimaging data. NeuroImage. 2015;117:343–57.
5. Gilson M, Moreno-Bote R, Ponce-Alvarez A, Ritter P, Deco G. Estimation of directed effective connectivity from fMRI functional connectivity hints at asymmetries in cortical connectome. PLoS Comput Biol. 2016.

## P171 Getting in the groove: testing a new model-based method for comparing task-evoked versus resting-state activity in fMRI data on music listening

### Matthieu Gilson^1,†^, Maria A. G. Witek^2,†^, Eric F. Clarke^3^, Mads Hansen^4^, Mikkel Wallentin^5^, Gustavo Deco^1^, Morten L Kringelbach^2,5,6^, Peter Vuust^2,5^

#### ^1^Center for Brain Cognition, Universitat Pompeu Fabra, Barcelona, Spain; ^2^Center for Music in the Brain, Aarhus University & Royal Academy of Music, Aarhus/Aalborg, Denmark; ^3^Faculty of Music, University of Oxford, UK; ^4^Department of Psychology and Behavioural Sciences, Aarhus University, Denmark; ^5^Center of Functionally Integrative Neuroscience, Aarhus University, Denmark; ^6^Department of Psychiatry, University of Oxford, UK

##### **Correspondence:** Matthieu Gilson - matthieu.gilson@upf.edu

*BMC Neuroscience* 2016, **17(Suppl 1)**:P171

^†^ Equal contribution.

Much of present neuroimaging studies have used fMRI to simply measure the activity in brain regions and computing the functional connectivity (FC) between regions and behaviour. This has provided important insights into the task-evoked activity compared to rest and thus the flow of information between functional regions (e.g., sensory, multimodal integration, memory). Yet, this does not capture all of the complex spatiotemporal patterns of brain activity and in particular not been the underlying effective connectivity. The present study provides evidence for application of a novel method of determining the functional roadmap of effective connectivity (EC), which measures the strengths of dynamic cortical interactions. We use a whole-brain dynamical model that combines fMRI data with anatomical information obtained using diffusion-tensor imaging (DTI) [1]. Our recently developed method [2] provides estimates for the EC as well as the local excitability and stimulus load in a study of groove-based music. The brain is divided into 90 areas and the input noise is shaped by the EC to generate the FC. This model allows us to explore the role of the network parameters in shaping FC: after constraining the model to reproduce resting-state activity, we examine the effect of an arbitrary change in individual inputs and EC strengths on FC. Our method focuses on spatio-*temporal* FC, meaning covariances of BOLD signals with possible time shifts. The estimated EC and inputs are taken as fingerprints of the brain dynamics. We analyze fMRI data of participants listening to 15 rhythms with three levels of syncopation: Low, Medium and High (five drum-breaks in each level) [3]. In accordance with other studies, we find behaviourally that the Medium level—with more “groove”—elicits the most pleasure and wanting to move. We tune the model to reproduce the FC recorded for each syncopation level, as well as rest. We analyze significant changes for each groove condition as compared to rest. FC for Medium syncopation exhibits a faster shuffling between successive brain patterns of activity, linked to more metastability and corresponding maybe to the subjective experience of higher pleasure. In addition, our model gives a detailed functional neuroanatomy of dynamical changes in the brain networks. Interestingly, Medium syncopation induces changes in excitability in the basal ganglia, such as the pallidum and the caudate nucleus, which may be related to the increased desire for moving. Interestingly, significant changes are also observed in regions of the orbitofrontal and anterior cingulate cortices, which have been strongly implicated in the pleasure network [4]. Overall, our new method has for the first time allowed us to uncover the network and the corresponding effective connectivity of a highly pleasurable state of groove; possibly even revealing the brain topography of eudaimonia, the sense of well-being.

**Acknowledgements:** MG and GD were supported by the FP7 FET ICT Flagship Human Brain Project (604102). GD was supported by the ERC Advanced Grant: DYSTRUCTURE (n. 295129), by the Spanish Research Project SAF2010-16085. MLK was supported by the ERC Consolidator Grant: CAREGIVING (n. 615539). MLK, MW and PV were supported by the Center for Music in the Brain, funded by the Danish National Research Foundation (DNRF117).

**References**

1. Deco G, Jirsa V, McIntosh A. Emerging concepts for the dynamical organization of resting-state activity in the brain. Nat Rev Neurosci. 2011;12:43–56.
2. Gilson M, Moreno-Bote R, Ponce-Alvarez A, Ritter P, Deco G. Estimation of directed effective connectivity from fmri functional connectivity hints at asymmetries in cortical connectome. PLoS Comput Biol. 2016 (in press).
3. Witek MAG, Clarke E, Wallentin M, Kringelbach ML, Vuust P. Syncopation, body-movement and pleasure in Groove music. PLoS One. 2014;9:e94446.
4. Berridge KC, Kringelbach ML. Pleasure systems in the brain. Neuron. 2015;86:646–64.

## P172 Stochastic engine for pathway simulation (STEPS) on massively parallel processors

### Guido Klingbeil^1^, Erik De Schutter^1^

#### ^1^Computational Neuroscience Unit, Okinawa Institute of Science and Technology, 1919-1 Tancha, Onna-son, Kunigami-gun, Okinawa 904-0495, Japan

##### **Correspondence:** Guido Klingbeil - guido.klingbeil@oist.jp

*BMC Neuroscience* 2016, **17(Suppl 1)**:P172

STEPS is a stochastic reaction–diffusion simulator. Its emphasis is on accurately simulating signaling pathways in realistic morphologies [1].

It is becoming apparent that larger computational models are demanded to either capture more such morphologies or to simulate more complex systems. As an example, the dendrite calcium burst model presented by Anwar et al. [2] requires approximately 285,000 sub-volumes with 15 diffusing molecular species and 20 reactions per sub-volume. It required several weeks to compute a simulation of 500 ms.

Thus it is desirable to reduce the computational burden. Accelerators such as graphics processing units (GPU) offer unprecedented computing performance and are now common amongst the fastest super computers [3]. This project enables STEPS to benefit from the computational power of GPUs.

GPUs are massively parallel co-processors aggregating thousands of simplified processing cores onto a single chip. They share many characteristics with vector computers and a key challenge is that the processing cores are not independent. Similar to vector computers an operation is applied to a group of data elements rather than to the individual data element. Furthermore, the programmer has to mitigate the memory hierarchy of GPUs. While memory with a high access latency is, in general, abundant, fast memory space shared between threads is small which may limit the size of the reaction system one is able to simulate.

Previous research has shown that we can exploit the computational power of GPUs to accelerate spatially homogenous stochastic simulations by two orders of magnitude while avoiding the limitation imposed to the size of the reaction system to be simulated by the small fast memory space [4].

STEPS implements a spatial version of Gillespie’s stochastic simulation algorithm (SSA) computing reaction–diffusion systems on a mesh of tetrahedral sub-volumes [1, 5]. Currently a parallelised multi-processor version of STEPS is under development. Operator splitting techniques allow to separate the reaction of molecules within a sub-volume from the diffusion of molecules between them. This prevents computationally costly rollbacks in case of molecules diffusing between sub-volumes handled by different processors.

We develop a layered hybrid software architecture using both, the classic central processing unit as well as GPUs, integrated into STEPS applying GPU acceleration at the sub-volume level and integrating them into a coherent spatial simulation using operator splitting.

Our architecture will be a plug-in solution to STEPS not requiring any changes to the interfaces towards the user or other software systems of STEPS itself.

**References**

1. Hepburn I, et al.: STEPS: efficient simulation of stochastic reaction–diffusion models in realistic morphologies. BMC Syst Biol. 2012;6:36.
2. Anwar H, et al.: Stochastic calcium mechanisms cause dendritic calcium spike variability. J Neurosci. 2013;33(40):15848–67.
3. TOP500 Supercomputer Site [http://www.top500.org].
4. Klingbeil, et al. *S*tochastic simulation of chemical reactions with cooperating threads on GPUs (in preparation).
5. Gillespie DT. Exact stochastic simulation of coupled chemical reactions. J Phys Chem. 1977;81(25):2340–61.

## P173 Toolkit support for complex parallel spatial stochastic reaction–diffusion simulation in STEPS

### Weiliang Chen^1^, Erik De Schutter^1^

#### Computational Neuroscience Unit, Okinawa Institute of Science and Technology, Okinawa 904-0411, Japan

##### **Correspondence:** Weiliang Chen - w.chen@oist.jp

*BMC Neuroscience* 2016, **17(Suppl 1)**:P173

The studies of large neuronal pathway models with complex morphologies, such as our previous work on the stochastic effects of calcium dynamics in Purkinje cells [1], present a great challenge to currently available spatial stochastic reaction–diffusion simulators, for example, STEPS [2], as the model scales and complexities quickly surpass the capability any serial simulator can achieve.

One possible solution for this challenge is parallelization. At CNS2015 we reported a parallel implementation of STEPS which demonstrated great speedup when simulating a reduced calcium burst model with a tetrahedral cylinder [3], but it is clear that to explore the full potential of our implementation, a larger scale simulation with more complex geometry is required.

Here we extend our work by simulating the reduced calcium burst model with a reconstructed Purkinje dendrite tree branch mesh. Comparing to previous simulations with regular cylinder meshes, the simulation with dendrite tree mesh requires several new support routines from the simulator. First of all, the simple axis based partitioning approach used in the cylinder simulations is no longer a good partitioning solution due to the complex tree structure of the mesh. A sophisticated mesh partitioning and validation solution is therefore necessary for the new simulation. Second, the new simulation demands good regional annotation and data collection support as calcium concentration, the main focus of the simulation, varies both spatially and temporally. The parallel environment further increases the difficulty of such support as the simulation is distributed over a massive number of processors, and each annotated region may not be completely simulated within a single processor. Furthermore, it is necessary to minimize the user interface difference between serial and parallel STEPS solvers and extend the STEPS visualization toolkit to facilitate comparison with results from previous work.

In this poster we demonstrate the general procedure of converting a serial STEPS simulation to its parallel counterpart, using the reduced calcium burst model with complex tree mesh as example, and showcase new supporting toolkits developed for the procedure. We believe that the presented procedure and toolkits will be helpful to STEPS users in their future research.

**References**

1. Anwar H, Hepburn I, Nedelescu H, Chen W, De Schutter E. Stochastic calcium mechanisms cause dendritic calcium spike variability. J Neurosci. 2013;33(40):15488–867.
2. Hepburn I, Chen W, Wils S, De Schutter E. STEPS: efficient simulation of stochastic reaction–diffusion models in realistic geometries. BMC Syst Biol. 2012;6:36.
3. Chen W, Hepburn I, De Schutter, E. Implementation of parallel spatial stochastic reaction–diffusion simulation in STEPS. BMC Neurosci. 2015;16(Suppl 1):P54.

## P174 Modeling the generation and propagation of Purkinje cell dendritic spikes caused by parallel fiber synaptic input

### Yunliang Zang^1^, Erik De Schutter^1^

#### Computational Neuroscience Unit, Okinawa Institute of Science and Technology Graduate University, Onna-son, Okinawa, Japan

##### **Correspondence:** Yunliang Zang - yunliang.zang@oist.jp

*BMC Neuroscience* 2016, **17(Suppl 1)**:P174

The dendrite of Purkinje cell (PC) has been shown to express different types of voltage gated ion channels. After strong parallel fiber (PF) stimulus, calcium currents can cause dendritic spikes to occur in the spiny dendrite [1]. Different with climbing fiber caused calcium signals that propagate throughout the dendritic tree, PF caused dendritic spikes are local. The elevated calcium concentration due to the local dendritic spike may trigger local synaptic plasticity, possibly playing a significant role in information processing. However, until now, how these dendritic spikes originate and propagate is not well understood.

In this work, we built a new PC dendrite model, which can generate local dendritic calcium spikes. The generated spike by model shows similar properties to experimental observations [1], including spike threshold, amplitude and latency. We identify the role of P type Ca^2+^ current, A type K^+^ current, high threshold K^+^ current (Kv3), calcium activated K^+^ current and axial current on the depolarization and repolarization of the spike. In the model, the required threshold synaptic input to trigger local dendritic spikes decreases with distance from soma, which facilitates the occurrence of spikes in the spiny dendrite by PF synaptic input. This model can also successfully replicate the failure of propagation of PF caused dendritic spikes at the parent branch point. By analyzing the spatial spread of the dendritic spikes and EPSP signal to soma, we identify the relative contribution of active currents and impedance mismatch on the signal decay. Because dendritic spikes can robustly propagate over an entire branchlet in the direction away from the soma, dendritic branchlets may be the basic organization unit for integrating synaptic input [2, 3].

**References**

1. Rancz EA, Hausser M: Dendritic calcium spikes are tunable triggers of cannabinoid release and short-term synaptic plasticity in cerebellar Purkinje neurons. J Neurosci. 2006;26(20):5428–37.
2. De Schutter E, Bower JM. Simulated responses of cerebellar Purkinje cells are independent of the dendritic location of granule cell synaptic inputs. PNAS. 1994;91(11):4736–40.
3. Branco T, Hausser M. The single dendritic branch as a fundamental functional unit in the nervous system. Curr Opin Neurobiol. 2010;20(4):494–502.

## P175 Dendritic morphology determines how dendrites are organized into functional subunits

### Sungho Hong^1^, Akira Takashima^1^, Erik De Schutter^1^

#### ^1^Computational Neuroscience Unit, Okinawa Institute of Science and Technology Graduate University, Onna-son, Okinawa 904-0495, Japan

##### **Correspondence:** Sungho Hong - shhong@oist.jp

*BMC Neuroscience* 2016, **17(Suppl 1)**:P175

Studies have established that dendrites are not simple cables that deliver synaptic inputs to a spike initiation zone in a neuron but can also perform active transformation, which is termed “dendritic computation” [1]. In particular, it has been claimed that individual dendritic branch should function as a local computational subunit [2] and therefore single neurons (especially pyramidal neurons) can act like two-layer neural networks [3]. Evidence supporting these hypotheses is largely based on existence of active membrane mechanisms in dendrites that give rise to their rich computational capabilities (e.g. [4]) and independent operations [5]. However, dendritic morphology is also known to play a significant role: For example, spike backpropagation is effectively prevented in the cerebellar Purkinje cells mostly due to morphology, even when artificial active mechanisms supporting propagation are embedded in simulations [6]. Nevertheless, to our best knowledge, there has been only few studies that quantified how the real morphological structure can control the functional properties of dendrites by forming subunits.

Here we address this question by combining a data-driven statistical analysis and computational modeling approach: First, we simulated central neurons of diverse morphological types with the passive membranes where localized inputs were injected. Response patterns in the dendritic membrane were collected as “features” corresponding to the input sites. Then, our dimensionality reduction/clustering procedure grouped them into clusters, which we call “subunits”. We found that those subunits usually consist of a few nearby branches in many neuron types, containing 2.12 ± 0.13 dendritic terminals per subunit (mean ± SEM), whereas they consist of one or more branchlets in the cerebellar Purkinje cells (12.9 ± 0.82 terminals). We also found that the subunits are comparable with other functional properties such as sublinear summation of multiple synaptic inputs and spreading of a dendritic spike.

**Conclusions** The morphological branching pattern of a neuronal dendritic tree determines how dendrites are organized into functional subunits. This implies that principles governing synaptic integration and active events, such as dendritic spiking, can widely vary depending on the morphological type of the neuron.

**References**

1. London M, Häusser M. Dendritic computation. Annu Rev Neurosci. 2005;28:503–32.
2. Branco T, Häusser M. The single dendritic branch as a fundamental functional unit in the nervous system. Curr Opin Neurobiol. 2010;20:494–502.
3. Poirazi P, Brannon T, Mel BW. Pyramidal neuron as two-layer neural network. Neuron. 2003;37:989–99.
4. Branco T, Clark BA, Häusser M. Dendritic discrimination of temporal input sequences in cortical neurons. Science. 2010;329:1671–5.
5. Behabadi BF, Mel BW. Mechanisms underlying subunit independence in pyramidal neuron dendrites. Proc Natl Acad Sci USA. 2014;111:498–503.
6. Vetter P, Roth A, Häusser M. Propagation of action potentials in dendrites depends on dendritic morphology. J Neurophysiol. 2001;85:926–37.

## P176 A model of Ca^2+^/calmodulin-dependent protein kinase II activity in long term depression at Purkinje cells

### Criseida Zamora^1^, Andrew R. Gallimore^1^, Erik De Schutter^1^

#### Computational Neuroscience Unit, Okinawa Institute of Science and Technology Graduate University, Okinawa 904-0895, Japan

##### **Correspondence:** Won Hee Lee - criseida.chimal@oist.jp

*BMC Neuroscience* 2016, **17(Suppl 1)**:P176

Cerebellar long-term depression (LTD) is a form of synaptic plasticity involved in motor learning. It is characterized as a robust and persistent decrease in the synaptic transmission between parallel fibers (PF) and Purkinje cells (PC), which is expressed as a reduction in the number of synaptic AMPA receptors (AMPAR). LTD signaling network includes a PKC-ERK-cPLA_2_ positive feedback loop and mechanism of AMPAR trafficking. Previous studies suggest that Ca^2+^/calmodulin-dependent protein kinase II (CaMKII) is required for the LTD induction [1]. However, the molecular mechanism of how CaMKII contributes to LTD is not fully understood. Noise in the signaling networks plays an important role in cellular processes. LTD models including the CaMKII pathway have been developed [2], but they have not included the intrinsic stochasticity of molecular interactions.

Our lab recently developed a stochastic model of the LTD signaling network including a PKC-ERK-cPLA_2_ feedback loop and AMPAR trafficking [3]. In this work, we have extended the model by adding the molecular network regulating CaMKII activity, which is known to influence LTD. This new model was solved stochastically by STEPS (STochastic Engine for Pathway Simulation) to simulate the influence of noise in the LTD signaling network [4]. Some of the most important new components of this network include phosphatase 2A (PP2A), phosphodiesterase 1 (PDE1), cGMP/protein kinase G (PKG) and nitric oxide (NO) pathway.

Through stochastic modeling we showed that the requirement of CaMKII activity for LTD induction is controlled by its indirect inhibition of PP2A activity, with PP2A markedly suppressing the activation of LTD when CaMKII activity is decreased. The impairment of LTD could be rescued by the additional PDE1 reduction when CaMKII is reduced. In addition, the cGMP/PKG pathway supports LTD through its activation by NO. These results are congruent with previous studies of CaMKII activity [2] and make our stochastic model a potential tool to study the effects of CaMKII, phosphatases and phosphodiesterases in LTD molecular network.

**References**

1. Hansel C, de Jeu M, Belmeguenai A, Houtman SH, Buitendijk GH, Andreev D, De Zeeuw CI, Elgersma Y. αCaMKII is essential for cerebellar LTD and motor learning. Neuron. 2006;51:835–43.
2. Kawaguchi SY, Hirano T. Gating of long-term depression by Ca^2+^/calmodulin-dependent protein kinase II through enhanced cGMP signalling in cerebellar Purkinje cells. J Physiol. 2013;591(7):1707–30.
3. Antunes G, De Schutter E. A stochastic signaling network mediates the probabilistic induction of cerebellar long-term depression. J Neurosci. 2012;32(27):9288–300.
4. Hepburn I, Chen W, Wils S, De Schutter E. STEPS: efficient simulation of stochastic reaction–diffusion models in realistic morphologies. BMC Syst Biol. 2012;6:36.

## P177 Reward-modulated learning of population-encoded vectors for insect-like navigation in embodied agents

### Dennis Goldschmidt^1^, Poramate Manoonpong^2^, Sakyasingha Dasgupta^3,4^

#### ^1^Champalimaud Neuroscience Programme, Champalimaud Center for the Unknown, Lisbon, Portugal; ^2^Center of Biorobotics, Mærsk Mc-Kinney Møller Institute, University of Southern Denmark, Odense, Denmark; ^3^Riken Brain Science Institute, 2-1 Hirosawa, Wako, Saitama, Japan; ^4^IBM, IBM Research - Tokyo, Tokyo, 103-8510, Japan

##### **Correspondence:** Dennis Goldschmidt - dennis.goldschmidt@neuro.fchampalimaud.org

*BMC Neuroscience* 2016, **17(Suppl 1)**:P177

Many insects exhibit robust and efficient visual-based navigation in complex environments [1]. Specifically, behavioral studies on ants and bees showed that they are guided by orientation vectors based on a process called path integration. This process allows them to estimate their current location by integrating cues from odometry and a sun-based compass. While it is mainly applied to return back to the nest, it also guides learning of so-called vector memories for subsequent foraging [2, 3]. Vector memories can be anchored globally to the nest or locally to landmarks. Recent neurophysiological studies revealed that the central complex, an insect neuropil, contains neural representations of compass [4] and odometric cues [5]. However, it is still unclear, how these representations are involved in path integration and vector memories, and how they produce goal-directed navigation. Computational modeling has been powerful in testing hypotheses about the underlying neural substrates and their generated behavior, and to predict further experimental data. Previous models [6, 7] sufficiently produced insect-like vector navigation, but they neglected biologically plausible explanations about underlying neural mechanisms that could generate this behavior.

We present here a novel computational model of neural mechanisms in closed-loop control for vector navigation in embodied agents. It consists of a path integration mechanism, reward-modulated learning of global and local vectors, random search, and action selection. The path integration mechanism computes a vectorial representation of the agent’s current location. The vector is encoded in the activity pattern of circular arrays, where the angle is population-coded and the distance is rate-coded. We apply a reward-modulated learning rule for global and local vector memories, which associates the local food reward with the path integration state. A motor output is computed based on the combination of vector memories and random exploration. We show that the modeled neural mechanisms enable robust homing and localization in a simulated agent, even in the presence of external sensory noise. The proposed learning rules produce goal-directed navigation and route formation under realistic conditions. This provides an explanation for, how view-based navigational strategies are guided by path integration. As such, the model is the first to link behavioral observations to their possible underlying neural substrates in insect vector navigation.

**Acknowledgements:** We thank Florentin Wörgötter at the Department of Computational Neuroscience in Göttingen, where most of this work was conducted. SD acknowledges funding from the RIKEN Brain Science Institute.

**References**

1. Wehner R. Desert ant navigation: how miniature brains solve complex tasks. J Comp Physiol A. 2003;189(8):579–88.
2. Collett M, Collett TS, Bisch S, Wehner R. Local and global vectors in desert ant navigation. Nature. 1998;394(6690):269–72.
3. Collett TS, Collett M. Route-segment odometry and its interactions with global path-integration. J Comp Physiol A. 2015;201(6):617–30.
4. Seelig JD, Jayaraman V. Neural dynamics for landmark orientation and angular path integration. Nature. 2015;521(7551):186–91.
5. Martin JP, Guo P, Mu L, Harley CM, Ritzmann RE. Central-complex control of movement in the freely walking cockroach. Curr Biol. 2015;25(21):2795–803.
6. Cruse H, Wehner R. No need for a cognitive map: decentralized memory for insect navigation. PLoS Comput Biol. 2011;7(3):e1002009.
7. Kubie JL, Fenton AA. Heading‐vector navigation based on head‐direction cells and path integration. Hippocampus. 2009;19(5):456–79.

## P178 Data-driven neural models part II: connectivity patterns of human seizures

### Philippa J. Karoly^1^, Dean R. Freestone^1,2^, Daniel Soundry^2^, Levin Kuhlmann^3^, Liam Paninski^2^, Mark Cook^1^

#### ^1^Department of Medicine, The University of Melbourne, Parkville VIC, 3010, Australia; ^2^Department of Statistics, Columbia University, New York, NY, USA; ^3^Swinburne University of Technology, Hawthorn, VIC 3122, Australia

##### **Correspondence:** Philippa J. Karoly - pkaroly@student.unimelb.edu.au

*BMC Neuroscience* 2016, **17(Suppl 1)**:P178

Here we present a model-based estimation framework for electrocorticography (ECoG) data that provides insight into mechanisms of seizures; and can be used as a clinical tool to monitor and design new treatment strategies on a patient-specific basis.

**Fig. 91:**
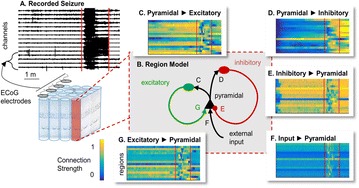
Example estimation of a seizure recording. **A** Sixteen channel electrocortiography (ECoG) of seizure (*red lines* indicate start and end points). **B** The ECoG channels are modelled as cortical regions, each with three coupled populations. **C**–**G** Estimation results of coupling strength (proportional to *color*) between neural populations for 16 cortical regions (*vertical axis*), over the time span of the seizure (*horizontal axis*)

Seizures are brief periods of abnormal, hypersynchronous neural firing that spreads across multiple cortical regions. People with epilepsy experience recurrent seizures, which are often untreatable and of unknown cause. The data-driven estimation framework, shown in Fig. 91, describes dynamic neural connectivity patterns during patient seizures. Data were obtained from a clinical trial for an implantable seizure warning device [1], which captured thousands of seizures. We estimated mean membrane potentials and connectivity strengths between excitatory, inhibitory and pyramidal populations using a non-linear, assumed density filter for the neural mass equations [2].

Estimated parameters provide insights into the mechanisms of the seizure, which are not apparent from ECoG alone. For instance, Panel E shows the seizure is preceded by a focal decrease in inhibition compared to the surrounding channels, with widespread disinhibition during the seizure. Joint state and parameter estimation was repeated for every seizure, and the results showed consistent, stereotypical effective connectivity patterns that differed between short (<20 s) and long seizures. This is an important finding, as understanding the regulatory factors implicated in stopping seizures can guide new pharmaceutical treatments and electrical counter-stimulation strategies. The successful application of the neural mass model to study epileptic seizures supports the use of data-driven estimation for the clinical management of epilepsy.

**References**

1. Cook MJ, et al. Prediction of seizure likelihood with a long-term, implanted seizure advisory system in patients with drug-resistant epilepsy: a first-in-man study. Lancet Neurol. 2013;12(6):563–71.
2. Freestone et al.: Data-driven neural models part I CNS 2016 abstract submission.

## P179 Data-driven neural models part I: state and parameter estimation

### Dean R. Freestone^1^, Philippa J. Karoly^1,2^, Daniel Soundry^3^, Levin Kuhlmann^4^, Mark Cook^1^

#### ^1^Department of Medicine, The University of Melbourne, Parkville VIC, 3010, Australia; ^2^Department of Electrical and Electronic Engineering, The University of Melbourne, Parkville VIC, 3010, Australia; ^3^Department of Statistics, Columbia University, New York, NY, USA; ^4^Swinburne University of Technology, Hawthorn, VIC 3122, Australia

##### **Correspondence:** Dean R. Freestone - deanrf@unimelb.edu.au

*BMC Neuroscience* 2016, **17(Suppl 1)**:P179

This work describes a novel algorithm for inferring neural activity and effective cortical connectivity from neuroimaging data. The ability to infer cortical network structure from data is an important step towards understanding and treating neurological disorders, such as epilepsy. However, statistical measures for correlation in neuroimaging data are ambiguous and bear little or no relation to physiology. On the other hand, estimating physiologically realistic connectivity is highly challenging due to the complex, non-linear dynamics of the brain. The algorithm we present overcomes this challenge by providing an exact solution to non-linear inversion for a class of biologically inspired neural network models.

**Fig. 92:**
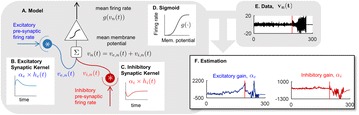
Data-driven model estimation. **A** The basic unit of a neural model is described by the mean membrane potential, v_n_(t), of a neural ensemble and synaptic inputs. **B**, **C** Pre-synaptic firing rates are convolved with the excitatory/inhibitory kernel to generate membrane potential fluctuations. **D** The resulting membrane potential is converted to an output firing rate via a sigmoidal transform. **E** Electrical recording of a seizure. **F** Estimated gain parameters during seizure

The presented algorithm performs joint state and parameter estimation for a class of neural model that represents interacting cortical regions as coupled nodes (shown in Fig. 92). The states of the model represent mean cortical activity [population membrane potentials, v(t)], and the parameters are the effective connectivity (synaptic gain kernels, α_i,e_). The output voltage, v_n_(t), represents the electrophysiology recording, which is inverted using a novel formulation of the Kalman filter equations for neural models [1]. The novelty of this method is the derivation of an exact solution to the integral over the distribution of hidden model states conditioned on previous data.

We provide results showing that the new algorithm demonstrates higher estimation accuracy and greater computational tractability than existing inference methods for neural models. We also show example estimation results from an electrical recording of a human seizure (shown in Fig. 92). This new method for data-driven inference represents an important contribution to online diagnostic applications, in particular for the treatment of epilepsy [2].

**References**

1. Freestone DR, et al. Estimation of effective connectivity via data-driven neural modeling. Front Neurosci. 2014;8:383
2. Karoly, et al. Data-driven neural models part II: connectivity patterns of human seizures. CNS 2016 abstract.

## P180 Spectral and spatial information processing in human auditory streaming

### Jaejin Lee^1^, Yonatan I. Fishman^2^, Yale E. Cohen^1^

#### ^1^Department of Otorhinolaryngology – Head and Neck Surgery, University of Pennsylvania, Philadelphia, PA 19104, USA; ^2^Department of Neurology, Albert Einstein College of Medicine, Bronx, NY 10461, USA

##### **Correspondence:** Jaejin Lee - jaejin@mail.med.upenn.edu

*BMC Neuroscience* 2016, **17(Suppl 1)**:P180

The purpose of auditory system is to transform acoustic stimuli from the external environment to sound perception. To achieve this goal, the auditory system needs to analyze a mixture of stimuli that originate from independent sources and distinguish individual sound sources in the auditory scene. It is believed that the auditory system groups and segregates auditory stimuli based on their regularities, but the neural basis of how regularities relate to sound perception is not well known. The ventral pathway in the brain is involved in auditory perception whereas the dorsal pathway is involved in spatial processing and audiometer processing. We are interested in how the spatial information is represented in the ventral pathway during perceptual auditory streaming tasks that use spatial information.

We first developed a novel task based on [1] in which human listeners can segregate streams using spectral or spatial information and detect the deviant tone. An array of 13 free field speakers with different spatial distributions were used to play the stimuli. The frequency difference between streams and the spatial separation were varied to explore how the spectral and spatial information interplay in the auditory streaming task. We also manipulated other acoustic features of the stimuli to understand how different acoustic cues can affect the auditory streaming performance. We found that the ability to segregate the streams is vastly improved when there is spatial information available in addition to spectral information. Also, we further analyzed the behavioral data to get psychophysical kernels and fit the data to variants of sequential sampling models related to the drift diffusion model(DDM) [2] to quantify the effects of sequence coherence on the decision making process.

**References**

1. Sussman E, Steinschneider M. Attention effects on auditory scene analysis in children. Neuropsychologia. 2009;47:771–85.
2. Liu A, Tsunada J, Gold J, Cohen Y. Temporal integration of auditory information is invariant to temporal grouping cues. eNeuro. 2015;2(2):e0077-14.

## P181 A tuning curve for the global effects of local perturbations in neural activity: mapping the systems-level susceptibility of the brain

### Leonardo L. Gollo^1^, James A. Roberts^1^, Luca Cocchi^1^

#### ^1^Systems Neuroscience Group, QIMR Berghofer Medical Research Institute, Herston, QLD 4006, Australia

##### **Correspondence:** Leonardo L. Gollo - leonardo.l.gollo@gmail.com

*BMC Neuroscience* 2016, **17(Suppl 1)**:P182

**Fig. 93:**
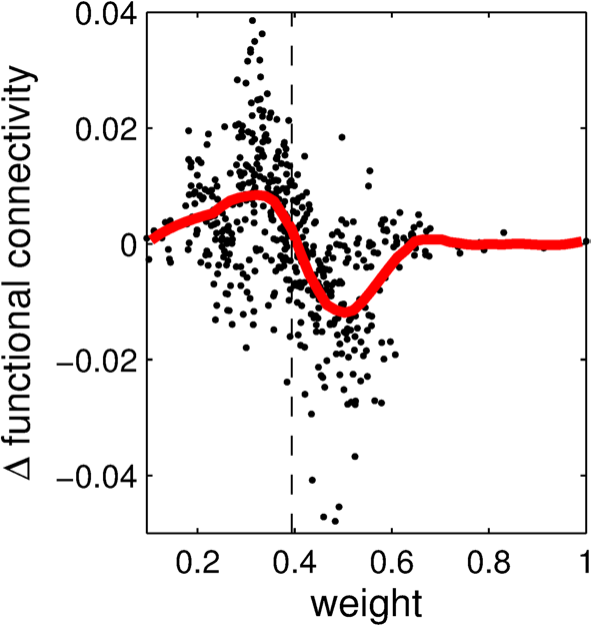
Changes in functional connectivity with respect to baseline after inhibitory stimulation as a function of cortical weight of the structural connectivity matrix. *Red line*: mean uniform bins curve smoothed; *dashed line*: mean weight

The activity of the human brain in a state of rest exhibits a defined pattern of functional connectivity, and a small set of functional networks, which comprise regions that are highly correlated and are mostly distinctive from one another [1]. However, despite recent efforts [2, 3], the effects of local perturbations into endogenous whole brain dynamics are not yet clearly understood [4]. To gain insights into the global effects of a focal perturbation, we simulate the human brain dynamics using a weighted high-resolution connectome of 513 cortical regions [5]. The cortical dynamics is modelled by a canonical oscillatory model, introducing heterogeneous dynamics between cortical regions as a function of the anatomical nodal strength (sum of weights). Such heterogeneity leads to a hierarchy of time scales of cortical regions recapitulating the known anatomical hierarchy, with peripheral regions having fast time scales and core regions with slow time scales [6]. Results showed that nodal diversity is not just a crucial element to improve the model’s performance [7, 8], but also to reproduce the experimental data of variations in functional connectivity following local inhibitory transcranial magnetic stimulation (TMS). We find a large variation in the overall effect of functional connectivity following local stimulation. Specifically, the inhibition of hub nodes causes increased anti-correlated activity, whereas inhibition of peripheral nodes caused increased correlated activity with the rest of the brain. The intensities of the variations in functional connectivity with respect to baseline were also highly variable and stronger for intermediary nodes that were not hubs or peripheral regions. Moreover, depending on the weights of the cortical regions, changes in functional connectivity form a tuning curve (Fig. 93). Overall, our findings suggest a key role of local temporal dynamics to explain the widespread effects of focal perturbations in neural activity.

**References**

1. Power JD, Cohen AL, Nelson SM, Wig GS, Barnes KA, Church JA, Vogel AC, Laumann TO, Miezin FM, Schlaggar BL, Petersen SE. Functional network organization of the human brain. Neuron. 2011;72:665–78.
2. Cocchi L, Sale MV, Lord A, Zalesky A, Breakspear M, Mattingley JB. Dissociable effects of local inhibitory and excitatory theta-burst stimulation on large-scale brain dynamics. J Neurophysiol. 2015;113:3375–85.
3. Kunze, T, Hunold A, Haueisen J, Jirsa V, Spiegler A. Transcranial direct current stimulation changes resting state functional connectivity: a large-scale brain network modeling study. NeuroImage. doi:10.1016/j.neuroimage.2016.02.015.
4. Sale MV, Mattingley JB, Zalesky A, Cocchi L. Imaging human brain networks to improve the clinical efficacy of non-invasive brain stimulation. Neurosci Biobehav Rev. 2015;57:187–98.
5. Roberts JA, Perry A, Lord AR, Roberts G, Mitchell PB, Smith RE, Calamante F, Breakspear M. The contribution of geometry to the human connectome. NeuroImage. 2016;124:379–93.
6. Gollo LL, Zalesky A, Hutchison RM, van den Heuvel M, Breakspear M. Dwelling quietly in the rich club: Brain network determinants of slow cortical fluctuations. Philos Trans R Soc B Biol Sci. 2015;370:20140165.
7. Mejias JF, Longtin A. Optimal heterogeneity for coding in spiking neural networks. Phys Rev Lett. 2012;108:228102.
8. Gollo LL, Copelli M, Roberts JA. Diversity improves performance in excitable networks. arXiv preprint arXiv:1507.05249. 2015 Jul 19.

## P182 Diverse homeostatic responses to visual deprivation mediated by neural ensembles

### Yann Sweeney^1^, Claudia Clopath^1^

#### ^1^Department of Bioengineering, Imperial College London, UK

##### **Correspondence:** Yann Sweeney - y.sweeney@imperial.ac.uk

*BMC Neuroscience* 2016, **17(Suppl 1)**:P182

Visual deprivation paradigms provide crucial insight into the homeostatic response in visual cortex. We explore how neurons within functional ensembles may exhibit correlated homeostatic responses to visual deprivation, and how the source of common inputs to these ensembles determine the extent of their homeostatic recovery. We hypothesise that common inputs from non-visual stimuli are responsible for driving recovery from visual deprivation.

We simulate development during spontaneous and evoked activity in a recurrent network model of visual cortex in which Hebbian and homeostatic synaptic plasticity is implemented. This leads to the emergence of highly interconnected ensembles of neurons driven by either common visual or common non-visual inputs. When we then deprive the developed network of visual input, the homeostatic response is a strengthening of activity within ensembles which share common non-visual inputs. A broad reduction in inhibition across the network is also observed. Interestingly, the magnitude of the homeostatic response depends on the size of these ensembles, with larger ensembles more likely to fully recover from visual deprivation. Our results demonstrate the importance of investigating functional plasticity of ensembles triggered by sensory deprivation paradigms.

**Acknowledgements:** This research was supported by the Engineering and Physical Sciences Research Council (EPSRC), the Leverhulme Trust and Google Faculty Award.

## P183 Opto-EEG: a novel method for investigating functional connectome in mouse brain based on optogenetics and high density electroencephalography

### Soohyun Lee^1,3^, Woo-Sung Jung^1,2^, Jee Hyun Choi^3^

#### ^1^Department of Physics, POSTECH, Pohang, 37673, South Korea; ^2^Department of Industrial and Management Engineering, POSTECH, Pohang, 37673, South Korea; ^3^Center for Neuroscience, KIST, Seoul, 02792, South Korea

##### **Correspondence:** Jee Hyun Choi - jeechoi@kist.re.kr

*BMC Neuroscience* 2016, **17(Suppl 1)**:P183

**Fig. 94:**
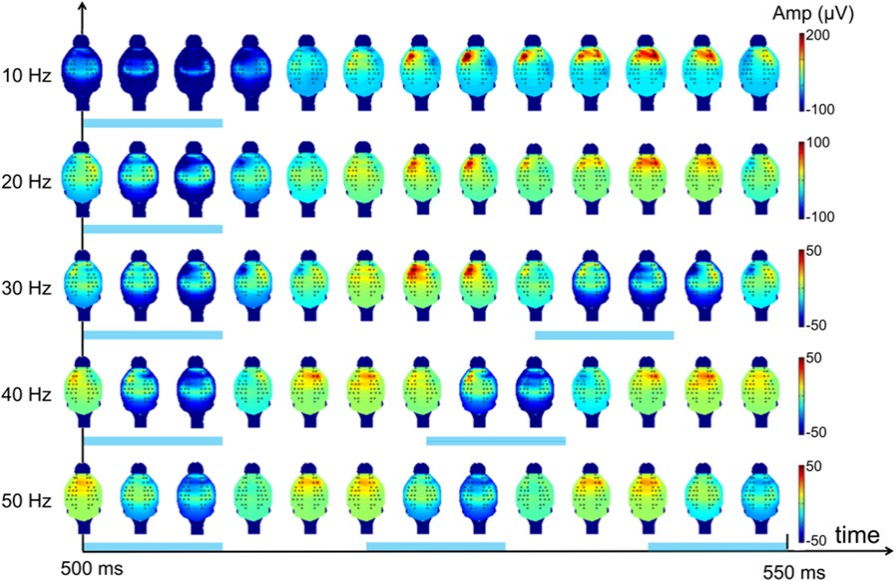
Propagation patterns of optical stimulation at each frequency in thalamocotical circuit. Beta frequency stimulus propagated S1–M1 strongly, but gamma frequency case, contralateral propagation is dominant. *Blue bars* indicate optical stimulus in left VPM

Connectome, comprehensive structural description of the network of elements and connections forming the brain [1], is fundamental for understanding the brain functions. Recent advances in optical imaging techniques allow us to be feasible to structural connectivity. But differently from structural connectivity, the functional connectivity is altered by condition such as brain states, input types and pathological conditions. To construct functional connectome, the techniques to map individual functional circuit and control specific neuronal activity have been needed. However, the current functional brain mapping techniques have limitations to obtain the map of the functionally correlated brain activity in freely moving mouse model. Here, we introduce novel functional brain mapping technique for mouse model by high density electroencephalography [2] under optogenetic stimulus, which we referred as opto-EEG. Opto-EEG tool enables us to investigate the functionally connected neuronal circuit with high spatial and temporal resolution. We stimulated ventral posterioromedial thalamic nucleus (VPM) with various frequencies for verifying different frequency dependency of functional connectome. Stimulation of VPM induced sequential activations of ipsilateral somatosensory cortex (S1) followed by ipsilateral motor cortex (M1), contralateral M1 and contralateral S1. The power based analysis result showed information flow between S1 and M1 was maximized under beta frequency stimulus. On the other hand, latency-based result showed minimized interhemispheric transfer latency under gamma band (Fig. 94). This example indicates that opto-EEG makes it possible to be used to characterize the functional connectivity under temporally precise control of specific neuronal circuits, provide new insights into brain exploration capabilities of functional connectome, and be applied to discover neuromodulation method for treatment of disease or pathologies.

**References**

1. Sporns O, Tononi G, Kotter R. The human connectome: A structural description of the human brain. PLoS Comput Biol. 2005;1(4):e42.
2. Lee M, Kim D, Shin HS, Sung HG, Choi JH. High-density EEG recordings of the freely moving mice using polyimide-based microelectrode. J Vis Exp. 2011;(47).

## P184 Biphasic responses of frontal gamma network to repetitive sleep deprivation during REM sleep

### Bowon Kim^1,2^, Youngsoo Kim^3^, Eunjin Hwang^1^, Jee Hyun Choi^1,2^

#### ^1^Center for neuroscience, Korea Institute of Science and Technology, Seoul, South Korea; ^2^Department of Neuroscience, University of Science and Technology, Daejon, South Korea; ^3^Department of Psychiatry, VA Boston Healthcare System & Harvard Medical School, Brockton, MA, USA

##### **Correspondence:** Jee Hyun Choi - jeechoi@kist.re.kr

*BMC Neuroscience* 2016, **17(Suppl 1)**:P184

**Fig. 95:**
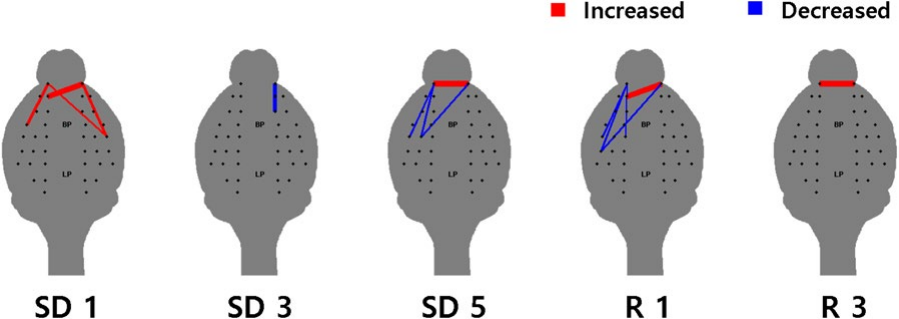
The pairs with statistically significantly increased (*red*) or decreased (*blue*) PSI of gamma oscillations (Student t test, p < 0.05). Only the pairs from the prefrontal cortex were depicted here. SD and R stand for sleep deprivation and recovery days, respectively

Prefrontal cortex has been known to be less activated [1] and decoupled from the other cortical area in REM sleep [2]. In our previous study of chronic sleep deprivation (SD) in mice model, we observed that the 5 successive days of SD (SD 1–5, 18 h sleep deprivation in each day) induced a monotonic increase of the prefrontal gamma oscillation (30–40 Hz) in REM sleep as the sleep pressure increased. However, the functional role of this increased gamma oscillation was not answered. Here, we investigated the functionality of the increased prefrontal gamma in sleep deprived nights by calculating the connectivity between the prefrontal cortex and the other cortical regions [3]. Phase synchrony index (PSI) was employed to minimize the volume conduction in high density EEG microarray. In the first day of the sleep deprivation (SD 1), we observed statistically significant increases in gamma connectivity within the bilateral prefrontal regions and between prefrontal and ipsilateral somatosensory cortex. However, as the sleep deprivation continued, an opposite response of prefrontal-somatosensory gamma connectivity was observed in a way that the PSI between these two areas become insignificant in SD 3 and statistically significantly decreased in the SD 5, which remained even after the 3rd day of recovery after the sleep deprivation (R 3). The area of decreased gamma connectivity became broader as well. On the other hand, the intracortical connectivity within prefrontal connectivity remained elevated throughout the sleep deprivation and recovery days. This result implies that the increased prefrontal gamma oscillation due to the homeostatic response of REM sleep does not participate in the information transfer from prefrontal to the other cortical area (Fig. 95).

**Acknowledgements:** This research was supported by the Korean National Research Council of Science and Technology (No. CRC-15-04-KIST).

**References**

1. Maquet P, et al. Functional neuroanatomy of human rapid-eye-movement sleep and dreaming. Nature. 1996;383(6596):163–6.
2. Castro S, et al. Coherent neocortical 40‐Hz oscillations are not present during REM sleep. Eur J Neurosci. 2013;37(8):1330–9.
3. Hwang E, McNally JM, Choi JH. Reduction in cortical gamma synchrony during depolarized state of slow wave activity in mice. Front Syst Neurosci. 2013;7.

## P185 Brain-state correlate and cortical connectivity for frontal gamma oscillations in top-down fashion assessed by auditory steady-state response

### Younginha Jung^1,2^, Eunjin Hwang^1^, Yoon-Kyu Song^2^, Jee Hyun Choi^1,3^

#### ^1^Center for Neuroscience, Korea Institute of Science and Technology, Seoul 02792, Korea; ^2^Program in Nano Science and Technology, Seoul National University, Seoul 08826, Korea; ^3^Department of Neuroscience, University of Science and Technology, Daejon 34113, Korea

##### **Correspondence:** Jee Hyun Choi - jeechoi@kist.re.kr

*BMC Neuroscience* 2016, **17(Suppl 1)**:P185

Cortical gamma rhythm, particularly in the frequency range of 30–50 Hz, has received intensive attention as neural correlates of cognitive process [1]. On the other hand, diminished cognitive flexibility, one of the typical symptoms in psychiatric disorders, is closely associated with disturbances in neural oscillations, specifically gamma band [2]. To quantify gamma-band oscillation, auditory steady-state response (ASSR) evoked by repetitive auditory stimulus given at a rate of 40 Hz has been used as a prominent approach which reflects neural efficiency for maintaining gamma oscillation [3]. Despite its diagnostic advantages, there is less discussion whether ASSRs are modulated by endogenous top-down effect. The present research attempts to investigate top-down influences on ASSR by analyzing in vivo mouse data.

Experimental data in this study were obtained from 38-channel mouse epidural electroencephalogram during auditory steady-state stimulus. Interestingly, there were two distinctive topographic maps of EEG spectral power and the notable difference between topographies was the presence or absence of frontal responses. By comparing topographic results, we hypothesized that frontal ASSRs reflect top-down functioning. The analytic approaches taken in this work are based on brain-state alteration and regional connectivity. The first research question in the data analysis is that frontal ASSRs switch states of arousal via top-down control. Video-based behavior analysis was adapted to classify arousal states into wakefulness and drowsiness and the proportion of arousal behavior in two topographies were determined. In addition, comparison of delta spectral power for topographic patterns could explain frontal engaged sleep state modulation. The second study question is about early stages of auditory ascending pathway in each topographic pattern. Magnitude and latency of auditory evoked potentials and gamma spectral power were analyzed in inferior colliculus and primary auditory cortex. The third question in data analysis is functional connectivity among cortical regions and, in detail, phase-locking value and directed phase lag index were calculated in frontotemporal and inter-frontal coupling.

Overall, the current results show that frontal lobe contributes substantially to ASSR and imply that it is important to consider the frontal involvement in auditory steady-state signal processing. Together, these methodologies could provide important insights to clinical research by demonstrating top-down modulation. Investigating gamma oscillatory activity across cortical regions potentially provides deeper understanding for dysfunction in neurological disorders and furthermore gives clues to determine neural circuit disruption.

**References**

1. Fries P, Reynolds JH, Rorie AE, Desimone R: Modulation of oscillatory neuronal synchronization by selective visual attention. Science. 2001;291(5508):1560–3.
2. Kwon JS, O’Donnell BF, Wallenstein GV, Greene RW, Hirayasu Y, Nestor PG, Hasselmo ME, Potts GF, Shenton ME, McCarley RW. Gamma frequency-range abnormalities to auditory stimulation in schizophrenia. Arch Gen Psychiatry. 1999;56(11):1001–5.
3. Picton TW, John MS, Dimitrijevic A, Purcell D. Human auditory steady-state responses. Int J Audiol. 2003;42(4):177–219.

## P186 Neural field model of localized orientation selective activation in V1

### James Rankin^1^, Frédéric Chavane^2^

#### ^1^Center for Neural Science, New York University, 4 Washington Place, 10003 New York, NY, USA; ^2^Institut de Neuroscienes de la Timone (INT), CNRS & Aix-Marseille University, 27 Boulevard Jean Moulin, 13005 Marseille, France

##### **Correspondence:** James Rankin - james.rankin@nyu.edu

*BMC Neuroscience* 2016, **17(Suppl 1)**:P186

Voltage imaging experiments in primary visual cortex [1] have shown that local, oriented visual stimuli elicit stable orientation-selective activation within the stimulus retinotopic footprint. The cortical activation dynamically extends far beyond the retinotopic footprint, but the peripheral spread stays non-selective—a surprising finding given a number of studies showing the orientation specificity of long-range connections, e.g. [2]. We study the dynamics of these input-driven localized states in a planar neural field model building on an earlier theoretical study using radially symmetric inputs [3]. Here we use a new anatomically-motivated connectivity profile and extend the model to multiple sub-populations encoding orientation. For canonical choices of connectivity profile (such as a radial difference of Gaussians), localized orientation selectivity arises. However, unlike the experimental observations, the selective activation is unstable during transient dynamics. In the new connectivity profile defined in our study, the range of excitatory and inhibitory connections and the orientation selectivity of those connections are controlled with separate parameters. We demonstrate how peaks in the number of excitatory connections at each hyper-column distance [4] are crucial in stabilizing the transient, local orientation selective activation. If these peaks in excitation are non-realistically exaggerated, we demonstrate that spurious selectivity (not matching preference map) could arise in the peripheral spread. Furthermore, although orientation selectivity of connections increases accuracy of the selective activation within the retinotopic footprint, it can also lead to orientation selective activation in the periphery. Our parameter exploration shows that with a balance in the sharpness of peaks in long-range excitatory connections and the selectivity of these connections, we can capture the correct localized selective activation, the non-selective peripheral spread and the stable transient dynamics.

**Conclusions** Typical choices of connectivity profile in planar models of cortex fail to produce important aspects of the observed cortical spread of activation. We developed a more realistic connectivity profile inspired by anatomical data that, used in conjunction with our planar multiple sub-population model, captures all key spatial and temporal aspects of the cortical spread of activation. For the first time, our study shows that the unexpected experimental findings of [1] can be accounted for with a realistic balance between the sharpness of peaks in long-range excitation and orientation selectivity of connections.

**References**

1. Chavane F, Sharon D, Jancke D, Marre O, Frégnac Y, Grinvald A. Lateral spread of orientation selectivity in V1 is controlled by intracortical cooperativity. Front Syst Neurosci. 2011;5:4
2. Bosking WH, Zhang Y, Schofield B, Fitzpatrick D. Orientation selectivity and the arrangement of horizontal connections in tree shrew striate cortex. J Neurosci. 1997;17:2112–27.
3. Rankin J, Avitabile D, Baladron J, Faye G, Lloyd DJ. Continuation of localized coherent structures in nonlocal neural field equations. SIAM J Sci Comput. 2014;36:B70–93.
4. Buzás P, Eysel U, Adorján P, Kisvárday Z. Axonal topography of cortical basket cells in relation to orientation, direction, and ocular dominance maps. J Comp Neurol. 2001;437:259–85.

## P187 An oscillatory network model of Head direction and Grid cells using locomotor inputs

### Karthik Soman^1^, Vignesh Muralidharan ^1^, V. Srinivasa Chakravarthy^1^

#### ^1^Department of Biotechnology, Indian Institute of Technology Madras, Chennai, Tamil Nadu, India

##### **Correspondence:** V. Srinivasa Chakravarthy - schakra@iitm.ac.in

*BMC Neuroscience* 2016, **17(Suppl 1)**:P187

**Fig. 96:**
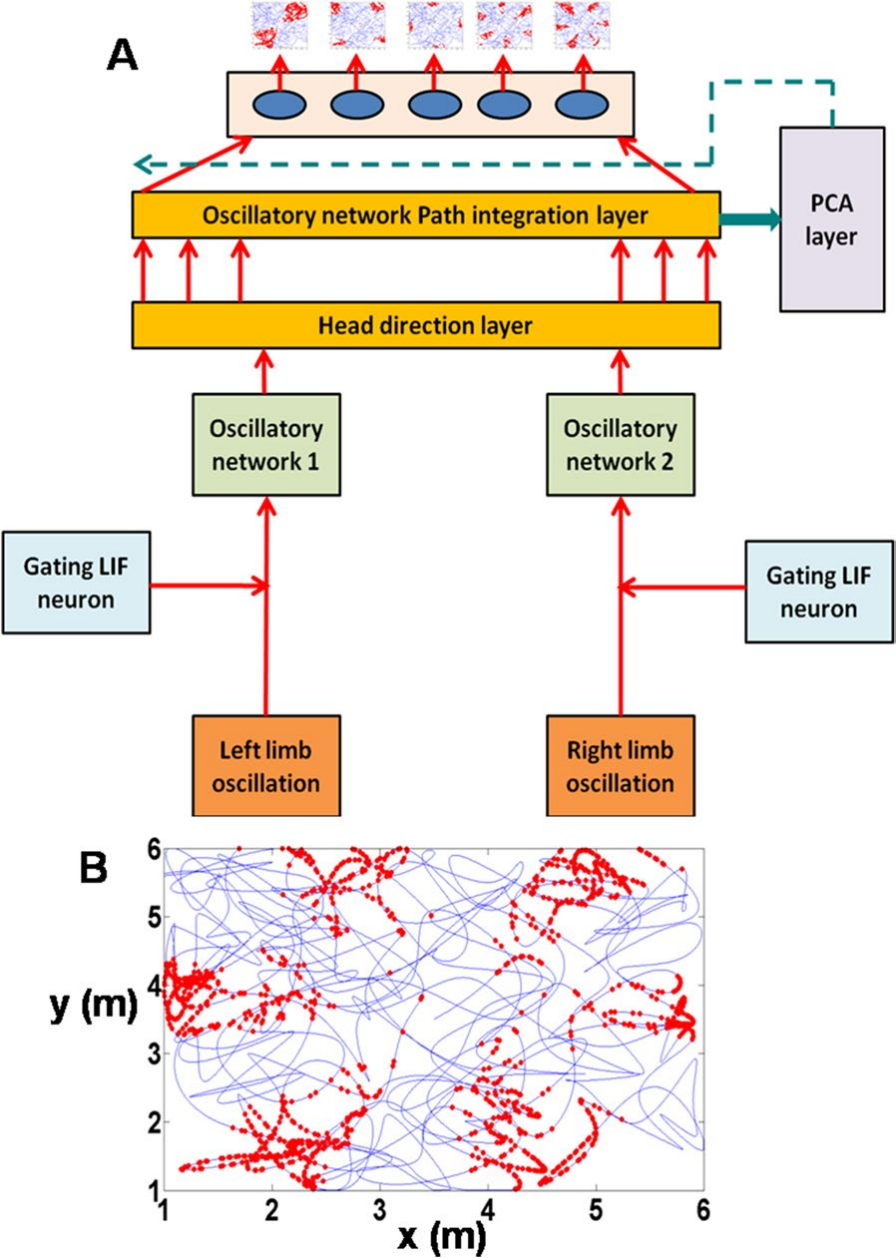
**A** The model architecture. **B** Hexagonal firing field of a single neuron in the outer layer of the model while remaping its response on the visual space

The model (Fig. 96A) takes proprioceptive inputs coming from the joint angles of the two limbs. Locomotor rhythms are modeled as two sinusoidal oscillators whose amplitudes are modulated by the curvature and the speed of simulated animal. These inputs are gated using a leaky integrate and fire (LIF) neuron that spikes at a fixed frequency so that the curvature and speed information from respective limb oscillations are extracted out and given to two oscillatory neural networks separately. The oscillatory neural networks are modeled as Kuramoto networks in which the phase is modulated by the curvature of the path traversed by the simulated animal. Synchrony between the two clusters of oscillators is quantified in terms of phase coherence and phase difference. The synchrony parameters are further used to train a one dimensional self organizing map (SOM), whose neurons display head direction-tuned responses. Each HD response is transformed to a cosine response which is further given to path integration (PI) layer. PI layer is again a network of Kuramoto oscillators whose phase is integrated as a function of HD responses. PCA is done on the PI values. The top few principal components (PC) corresponding to the largest Eigen values are rearranged in increasing order and taken as the weight connections from the PI layer to an outer 1-D layer of neurons. While remapping the neural response of the third and fourth PCs, square grid fields were observed while the fifth and sixth PCs gave

We present a model of head direction (HD) and grid cells formed purely from idiothetic (locomotor) inputs. Grid cells are a class of spatial cells located in the medial Entorhinal Cortex which is assumed to perform path integration and characterized by its unique hexagonal firing fields [1]. Empirically it is proven that HD cells, another class of spatial cells which encode the heading direction of an animal, form the major input to the grid cell. Existing computational models of grid cells make artificial assumptions like existence of HD cells with a phase differences that are integral multiples of 60° [2]. The aim of the study is to model grid cell firing without imposing these special constraints. Hexagonal grid fields (Fig. 96B). Further analysis showed that PCs were sinusoidal vectors. Investigation of the correlation values between the adjacent rows of the covariance matrix of PI pointed out its similarity to circulant matrices. This was in corroboration with the circulant matrix theorem which states that a circulant matrix of any size gives rise to sinusoidal Eigen vectors. Hence this is a generalized model which provides a theoretical basis for the formation of both hexagonal and non hexagonal grid fields and possibly other spatial cells which are actually projections of the PI values onto sinusoidal orthonormal basis vectors.

**References**

1. Hafting T, Fyhn M, Molden S, Moser M-B, Moser EI. Microstructure of a spatial map in the entorhinal cortex. Nature. 2005;436(7052):801–6.
2. Burgess N, Barry C, O’Keefe J. An oscillatory interference model of grid cell firing. Hippocampus. 2007;17(9):801–12.

## P188 A computational model of hippocampus inspired by the functional architecture of basal ganglia

### Karthik Soman^1,*^, Vignesh Muralidharan^1,*^, V. Srinivasa Chakravarthy^1^

#### ^1^Department of Biotechnology, Indian Institute of Technology Madras, Chennai, Tamil Nadu- 600036, India

##### **Correspondence:** V. Srinivasa Chakravarthy - schakra@iitm.ac.in

*BMC Neuroscience* 2016, **17(Suppl 1)**:P188

* Both authors have equal contribution.

**Fig. 97:**
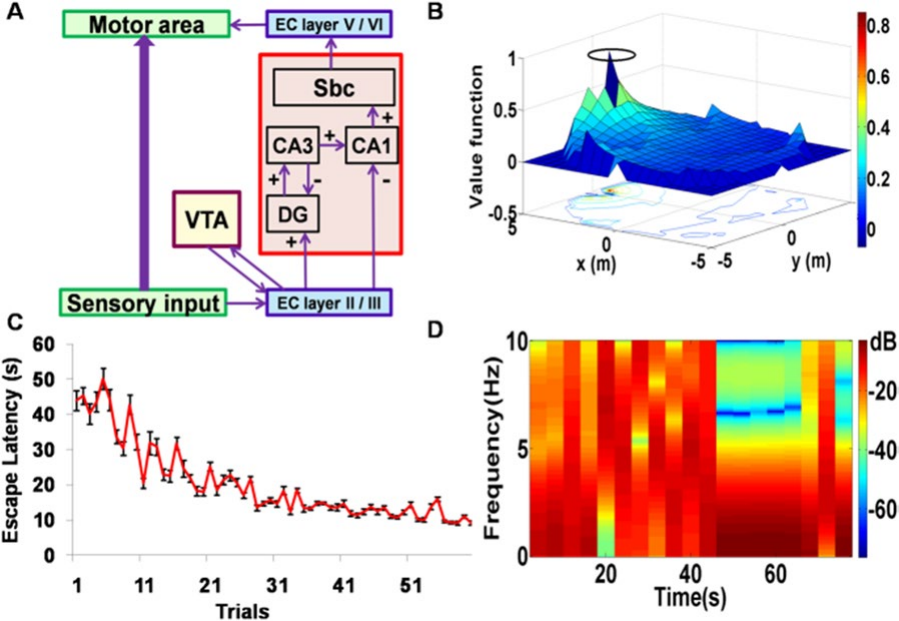
The model architecture (**A**) used to simulate the water maze task indicating the notion of a direct and an indirect pathway. The value function (**B**) developed after training the agent for 10 trials, the value peaks near to the platform. The escape latency (**C**) through trials shows that the agent has learnt the task with increased hippocampal dependence in the earlier stages and cortical dependence in the later stages of learning. The spectrogram (**D**) of the activity of CA3 as the function of time shows desynchronization while active exploration of the maze (0–45 s) and synchronized activity upon reaching the platform (45–65 s)

We present a networkmodel of hippocampus (HC) inspired by the functional architecture of the basal ganglia (BG). The model describes the role of hippocampus in spatial navigation and is cast in reinforcement learning (RL) framework (Fig. 97A). There is a corpus of literature which states that hippocampus is a key player in spatial learning because of the enriched sensory information that arrives at the portals of HC, the entorhinal cortex (EC), from the sensory cortical areas [1]. The model simulates the Morris water maze task wherein a virtual agent navigates inside a circular pool to find an invisible platform using the spatial context from the environment.

In order to model the ability of HC to learn spatial context, we simulate a circular pool surrounded by six distinguishable poles of equal heights. As the agent/animal navigates, the size of retinal image of each pole varies with distance between the agent and the pole. Reward is given to the agent when it reaches the platform. The abstract form of the visual input is given to EC which has afferent and efferent projections from ventral tegmental area (VTA), one of the dopamine centers in the mid brain. Temporal difference (TD) error generated from VTA is used to update the synaptic weights for value computation of the sensory input in EC. Additionally an action vector defining the direction of the agent’s next step also forms a feedback input to the EC. We describe the functional anatomy of HC in terms of two pathways: a direct pathway between EC and CA1 and an indirect pathway between EC and CA1 via dentate gyrus (DG), and CA3. A quantity known as Value difference, that represents afferent dopamine signals in EC, is thought to control switching between these pathways. Desynchronized activity generated by the DG–CA3 loop in the indirect pathway aids the agent to explore the space. Direct pathway facilitates the agent’s navigation. The difference in the responses from these two pathways is computed in CA1 and is relayed to subiculum (Sbc) which computes the direction of the next step. Output of Sbc is communicated to higher motor areas (MC), modeled as a 1-D Continuous attractor neural network, via deeper layers of EC. MC also receives direct inputs from sensory areas so that the output of MC is the weighted sum of responses from the sensory cortical areas and HC respectively. MC response is used to update the next step. Cortico-cortical pathway (CCP) connections are updated using the TD error as well as the velocity generated from HC as a target. As the value function matures (Fig. 97B), contribution from HC declines; the CCP connections gain the upper hand and the agent reaches the platform faster (Fig. 97C). Thus, after training, the CCP can drive navigation without the involvement of HC. Analysis of CA3 activity shows desynchronization during active exploration and synchronizationupon reaching the platform (Fig. 97D). This resonates with experimental results suggesting that low-amplitude theta waves correspond to desynchronized activity during exploration, whereas the sharp waves during non-exploratory states correspond to synchronized activity [2].

**References**

1. Sukumar D, Rengaswamy M, Chakravarthy VS. Modeling the contributions of Basal ganglia and Hippocampus to spatial navigation using reinforcement learning. PloS One. 2012;7(10):e47467.
2. Buzsáki G. Theta oscillations in the hippocampus. Neuron. 2002;33(3):325–40.

## P189 A computational architecture to model the microanatomy of the striatum and its functional properties

### Sabyasachi Shivkumar^1^, Vignesh Muralidharan^1^, V. Srinivasa Chakravarthy^1^

#### ^1^Department of Biotechnology, Indian Institute of Technology Madras, Chennai, Tamil Nadu, India-600036

##### **Correspondence:** V. Srinivasa Chakravarthy - schakra@iitm.ac.in

*BMC Neuroscience* 2016, **17(Suppl 1)**:P189

**Fig. 98:**
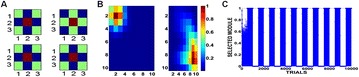
**A** Centre surround mapping in striosomes and matrisomes. The striosomes highlighted by *red*, map the states and the matrisomes highlighted by *green*, map the actions. **B** Value function map in the multiple context setting where the reward is present at the *top left* corner and the *bottom right* corner. **C** Switching of the modules based on the environmental contexts. The reward changes every 1000 episodes and the corresponding change in module with episode is shown

We propose a computational model of the functional architecture of the striatum. Anatomical and physiological evidence suggests that the microstructure of the striatum maps the sensory-motor information from the cortex in complex patterns. The dorsal striatum can be differentiated into centre surround regions called striosomes and matrisomes [1]. In the proposed striatum model, striosomes map the state space and the matrisomes map the action space. The model consists of a hierarchical two-level self organizing map (SOM), wherein the higher level SOM is trained on the state values and a sub-SOM layer containing multiple smaller SOMs are trained on action values. Neurons of ‘action SOMs’ are activated by neurons of ‘state SOMs’ The scheme of mapping of state space and action space onto the proposed architecture is given in Fig. 98A where the red area represents the striosomes and green area represents the matrisomes. We have also shown previously that such feature representation in a SOM layer can be used to develop value functions for sensory state spaces [2]. Thus to compute the state and action values, the activities of the respective SOMs were mapped to individual neurons by state and action weight vectors respectively. These weights were trained by the temporal difference error which represents the dopamine signals from the Substantia Nigra pars compacta (SNc) based on the reward from the environment. Action selection was performed by using the action values with exploration.

The model was further extended to reflect striatal modularity, which could also be exploited to solve modular RL tasks with varying contexts [3]. This is done by using multi-SOMs, where multiple SOMs compete with each other to represent the input space. Biologically, this competition between different local striatal maps can be thought to be carried out by striatal interneurons. Using the above described striatal model as a single module, multiple modules were created. The higher level SOMs in these modules generate a responsibility signal, which represent the ability of the module to best represent that context. This was used to select the modules, a selection process which is probably carried out by the tonically active neurons (TANs). To validate this overall architecture, we tested this on the gridworld problem with a 10 × 10 grid and 4 actions. The reward is present at the corner of the grid in the first case and in subsequent case of modular RL framework, the reward is placed at one of two opposite corners. The value function map is built between the two modules and their switching at regular intervals is given in Fig. 98B, C respectively.

**References**

1. Graybiel A, Flaherty A, Gimenez-Amaya J-M. Striosomes and matrisomes. In: The basal ganglia III edn. Berlin: Springer; 1991. p. 3–12.
2. Krishnan R, Ratnadurai S, Subramanian D, Chakravarthy VS, Rengaswamy M. Modeling the role of basal ganglia in saccade generation: Is the indirect pathway the explorer? Neural Networks. 2011;24(8):801–13.
3. Amemori K-I, Gibb LG, Graybiel AM. Shifting responsibly: the importance of striatal modularity to reinforcement learning in uncertain environments; 2011.

## P190 A scalable cortico-basal ganglia model to understand the neural dynamics of targeted reaching

### Vignesh Muralidharan^1^, Alekhya Mandali^1^, B. Pragathi Priyadharsini^1^, Hima Mehta^1^, V. Srinivasa Chakravarthy^1^

#### ^1^Department of Biotechnology, Indian Institute of Technology Madras, Chennai, Tamil Nadu-600036, India

##### **Correspondence:** V. Srinivasa Chakravarthy - schakra@iitm.ac.in

*BMC Neuroscience* 2016, **17(Suppl 1)**:P190

**Fig. 99:**
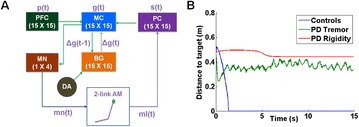
The model architecture (**A**) with the different modules aiding in reaching movements. The comparison of controls and PD’s approach to a target (**B**) and the appearance of PD symtoms including tremor and rigidity as a function of distance to the target

We present a scalable network model of the basal ganglia (BG) to highlight its role in performing simple reaching movements. The model consists of the following components: a 2-joint arm model (AM), a layer of motor-neurons in the spinal cord (MN), the proprioceptive cortex (PC), the motor cortex (MC), the prefrontal cortex (PFC) and the BG (Fig. 99A). The arm model has two joints each consisting of an agonist and an antagonist muscle pair innervated by a pair of motor neurons; the muscles in turn control the position of the arm in 2D space. The PC receives information about the muscle length and tension, thought to be originating from muscle spindles and Golgi tendon organs of the muscle. The MC then uses the sensory map information from the PC to develop a motor map of the arm. The MC activity is also modulated by the BG which uses reward information to make the arm learn to reach the target. The MC then sends these signals to respective muscles of the arm via the motor neurons (MN) to perform the movement. Since the existence of maps has been well established in the cortex, the sensory map of the PC, and the map from PC to MC were modelled using the self-organizing map (SOM) algorithm [1]. The motor command is thought to arise from the PFC, which specifies the goal to be reached. The MC therefore combines inputs from three sources: the PC, the prefrontal cortex (PFC), and the BG (from GPi via the thalamus). To enable this summation dynamically, MC was modelled as a continuous attractor neural network (CANN), wherein stable activity in CANN space corresponds to an equilibrium position of the arm in the workspace.

Training of the model proceeds as follows. A target is chosen by activating corresponding neurons in the PFC. The arm makes exploratory movements driven by the Indirect Pathway of BG and gets rewarded when it reaches the target. Now BG uses this reward information and the corresponding arm position to transform it into a value profile over the arm’s workspace such that the trained value peaks at the target positions. As the training of model proceeds, the arm reaches the goal position faster and faster as BG stochastically climbs over the trained value function [2]. Furthermore, the connections from PFC and MC are also trained on successful reach, so that the motor command can directly activate the motor cortex thereby producing rapid movement avoiding the slow search conducted by the BG. The model exhibits all stages of motor learning i.e., slow movements dominated by the BG during early stages and cortically driven fast movements at later stages. The simulation results show PD symptoms like tremor which could be attributed to synchronized oscillations in STN-GPe (Fig. 99B).

**References**

1. Chen Y, Reggia JA. Alignment of coexisting cortical maps in a motor control model. Neural Comput. 1996;8(4):731–55.
2. Magdoom K, Subramanian D, Chakravarthy VS, Ravindran B, Amari S-I, Meenakshisundaram N. Modeling basal ganglia for understanding parkinsonian reaching movements. Neural Comput. 2011;23(2):477–516.

## P191 Emergence of radial orientation selectivity from synaptic plasticity

### Catherine E. Davey^1^, David B. Grayden^1,2^, Anthony N. Burkitt^1^

#### ^1^Department of Electrical and Electronic Engineering, University of Melbourne, Victoria, 3010, Australia; ^2^Centre for Neural Engineering, University of Melbourne, Victoria, 3010, Australia

##### **Correspondence:** Catherine E. Davey - cedavey@unimelb.edu.au

*BMC Neuroscience* 2016, **17(Suppl 1)**:P191

The ability to learn and recall are primary functions of the brain. Synaptic plasticity is one of the key mechanisms by which we learn and adapt to our environment, and describes the process by which neuronal connection strengths are modified in response to environmental inputs [1]. There has been significant research effort invested into identifying the general principles of plasticity in neural networks, in order to garner insight into the learning process. The ability for animals to see and hear prior to birth is evidence of learning before exposure to ongoing external sensory signals. Consequently, cortical structure can be created, to some extent, in the absence of structured input. In a three-paper series, Linsker outlined a process by which cortical learning may occur prior to birth [2–4]. Linsker’s model identified particular spatial distributions of synaptic connectivity that are sufficient to induce the development of circularly symmetric cells in a system driven only by noisy input [2]. Furthermore, Linsker [3] revealed that orientation selective cells may develop by the sixth layer of processing. However, the resulting preferred orientation was a random function of stochastic weight initialisations [5].

Radial selectivity describes a tendency for cells to have the preferred orientation biased towards a central point, and has been observed in several cortical structures, including the visual cortex [6] and the auditory cortex [7]. In this study we reveal the mechanism by which radial orientation selectivity can emerge from synaptic plasticity in the absence of structured input. Linsker’s model assumed that cells within a laminar had an identical distribution of synaptic connection densities. This assumption is modified in this study to allow synaptic connection densities to change as a function of a cell’s radial distance to the centre of the laminar. The proposed network provides for spatially larger receptive fields as cells become progressively distal in the laminar, which is in keeping with electrophysiological and anatomical results. We show, both analytically and computationally, that this slightly modified network prompts the evolution of orientation selective cells with a predictable radial preference, in the third layer of neural processing. Importantly, this proposal maintains Linsker’s intent for a minimal set of model assumptions, ensuring that the resulting structure is robust to details and parameter values of the model used, and that general principles of plasticity are established. Consequently, our results are applicable to cortical learning generally. The mechanisms developed in this study could play a central role in the development of radial orientation selectivity in the visual cortex.

**Acknowledgements:** This research was supported under Australian Research Council’s Discovery Projects funding scheme (Project Number DP140102947).

**References**

1. Hughes JR. Post-tetanic potentiation. Phys Rev. 1958;38(1):91–113.
2. Linsker R. From basic network principles to neural architecture: emergence of spatial-opponent cells. Proc Natl Acad Sci USA. 1986;83:7508–12.
3. Linsker R. From basic network principles to neural architecture: emergence of orientation-selective cells. Proc Natl Acad Sci USA. 1986;83:8390–4.
4. Linsker R. From basic network principles to neural architecture: emergence of orientation columns. Proc Natl Acad Sci USA. 1986;83:8779–83.
5. Domany E, Hemmen JL van, Schulten K: Models of neural networks III. Berlin: Springer; 2012.
6. Schall JD, Vitek DJ, Leventhal AG. Retinal constraints on orientation specificity in cat visual cortex. J Neurosci. 1986;6(3):823–36.
7. Wang Y, Brzozowska-Prechtl A, Karten HJ. Laminar and columnar auditory cortex in avian brain. Proc Natl Acad Sci USA. 2010;107:12676–81.

## P192 How do hidden units shape effective connections between neurons?

### Braden A. W. Brinkman^1,2^, Tyler Kekona^1^, Fred Rieke^2,3^, Eric Shea-Brown^1,2,4^, Michael Buice^4^

#### ^1^Department of Applied Mathematics, University of Washington, Seattle, WA 98195, USA; ^2^Department of Physiology and Biophysics, University of Washington, Seattle, WA 98195, USA; ^3^Howard Hughes Medical Institute, University of Washington, Seattle, WA 98195, USA; ^4^Allen Institute for Brain Science, Seattle, WA, 98109, USA

##### **Correspondence:** Braden A. W. Brinkman - bradenb@uw.edu

*BMC Neuroscience* 2016, **17(Suppl 1)**:P192

A major challenge in neuroscience is understanding how “hidden units”—neurons or other influences not observed in an experiment—influence the behavior of the observed neurons. Much work has been done on the inferring network interactions from data [1, 2], but it remains unknown how hidden neurons shape the network interactions inferred. Using techniques from non-equilibrium statistical physics, we have developed a theoretical framework to predict how effective connections in subsampled networks depend on the true connections in the full network. Beyond calculating effective connections, this approach can be systematically expanded to study how hidden units generate effective noise in subsampled networks.

**Fig. 100:**
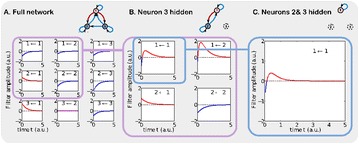
**A** Self-history filters (diagonal) and directed coupling filters between neurons (off-diagonal) in the full 3-neuron network. Neuron 1 is excitatory and its couplings to the other neurons are strictly positive. Neurons 2 and 3 are inhibitory and make strictly negative couplings to other neurons. There is no coupling from neuron 2–3. **B** Effective self-history filters (diagonals) and coupling filters (off-diagonals) when neuron 3 is hidden. The *bottom row* is unaltered because neuron 2 makes no coupling to neuron 3. The filters in the *top row* are changed due to the influence of signals neuron 1 sends to itself through neuron 3 and to neuron 2 through neuron 3. Although neuron 1’s true self-history filter and coupling from neuron 2 are negative the effective filters change sign. **C** The effective self-history filter of neuron 1 when both neurons 2 and 3 are hidden. Times and filter amplitudes are given in arbitrary units (a.u.)

As an example, we apply this framework to a network of three spiking neurons described by a generalized linear model (GLM) with rates driven by a neuron’s own filtered spiking activity and those from which it receives input. By approximating the subsampled network as a GLM with effective spike-filters corrupted by Gaussian noise, we can analytically calculate how hidden units transform the filters (Fig. 100) and give rise to correlations in the effective noise (not shown). Based on our 3-neuron results, we conjecture that for general networks within this framework the filter between neurons *i* and *j* is modified by corrections from every path that neuron *i* can send a signal to neuron *j* through hidden units.

**Acknowledgements:** Support provided by the Sackler Scholar Program in Integrative Biophysics (BAWB), CRCNS grant DMS-1208027 (ESB, FR), NSF-DMS-1056125 (ESB), NIH grant EY11850 (FR), HHMI (FR). ESB and MB thank the Allen Institute founders, Paul G. Allen and Jody Allen, for their vision, encouragement and support.

**References**

1. Pillow JP, Shlens J, Paninski L, Sher A, Litke AM, Chichilnisky EJ, Simoncelli EP. Spatio-temporal correlations and visual signalling in a complete neuronal population. Nature. 2008;454:995–9.
2. Pillow JW, Latham P. Neural characterization in partially observed populations of spiking neurons. Adv Neural Inf Process Syst. 2007;3256:1–8.

## P193 Characterization of neural firing in the presence of astrocyte-synapse signaling

### Maurizio De Pittà^1,2^, Hugues Berry^2,3^, Nicolas Brunel^1,3^

#### ^1^Department of Neurobiology, University of Chicago, Chicago, IL 60637, USA; ^2^Project-Team BEAGLE, INRIA Rhône-Alpes, Villeurbanne, F-69603, France; ^3^Department of Statistics, University of Chicago, Chicago, IL 60637, USA

##### **Correspondence:** Maurizio De Pittà - maurizio.depitta@gmail.com

*BMC Neuroscience* 2016, **17(Suppl 1)**:P194

We study analytically the dynamics of neural activity in the presence of synaptic inputs modulated by astrocyte-released neurotransmitters (i.e. the so-called “gliotransmitters”). We start with the simple scenario of gliotransmitter-mediated modulation of synaptic release probability at *N* excitatory synapses impinging on a single postsynaptic neuron as well as on the same astrocyte domain. In this scenario, release from pre-synaptic terminals leads to activation of the astrocyte, that in turn modulates synaptic release through gliotransmitter release. In the limit of *N* → ∞ synapses, we derive equations relating gliotransmitter release to the instantaneous presynaptic rate, identify conditions for co-existence of multiple states of synaptic release, and study their stability. In the bistable regime, long-lasting potentiation of synaptic release by gliotransmission accounts for the emergence of persistent postsynaptic firing. Analysis of the coefficient of variation (CV) of the ensuing interspike interval distribution reveals increased firing variability following stimulation and in the presence of gliotransmission, in close analogy with increased CV values experimentally observed during the delay period in working-memory related tasks. We then extend our analysis to the scenario of a balanced neural network coupled with a network of astrocytes, and demonstrate the existence of an analogous mechanism for persistent neural firing by mean field theory. Taken together, our analysis suggests a novel astrocyte-based mechanism for persistent activity, and provides experimentally testable hypotheses on the possible involvement of astrocytes in cognitive tasks related to working memory.

## P194 Metastability of spatiotemporal patterns in a large-scale network model of brain dynamics

### James A. Roberts^1^, Leonardo L Gollo^1^, Michael Breakspear^1^

#### ^1^Systems Neuroscience Group, QIMR Berghofer Medical Research Institute, Brisbane, QLD 4006, Australia

##### **Correspondence:** James A. Roberts - james.roberts@qimrberghofer.edu.au

*BMC Neuroscience* 2016, **17(Suppl 1)**:P194

**Fig. 101:**
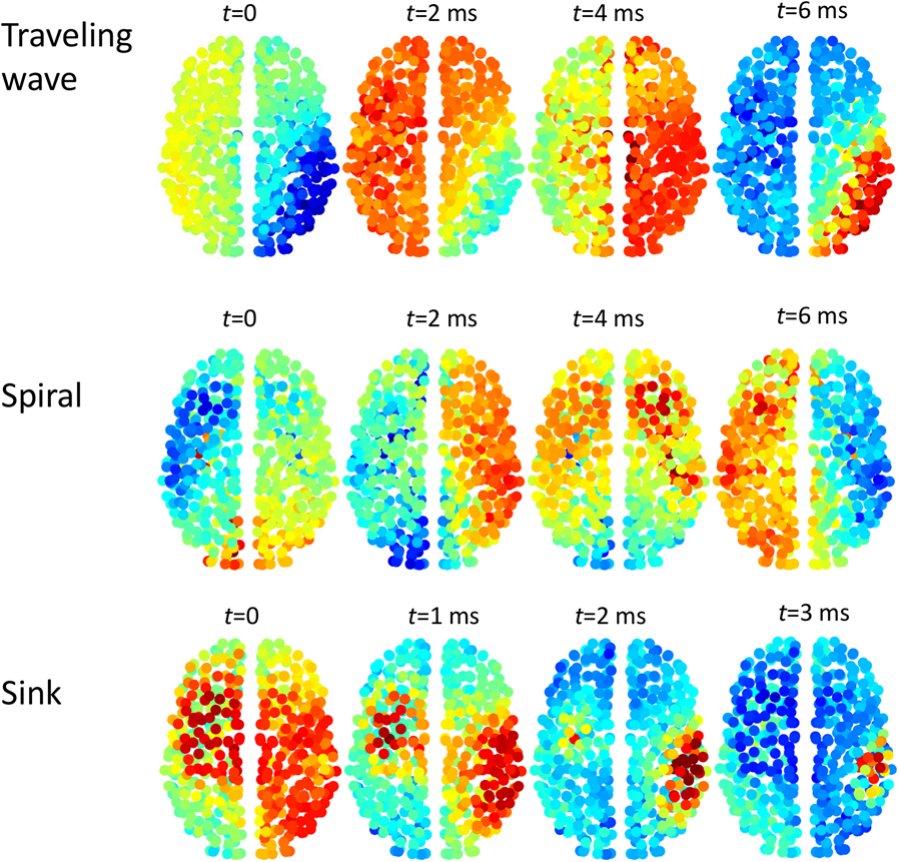
Large-scale wave patterns for strong coupling, showing four time snapshots for a traveling wave (*top*), a spiral wave (*middle*), and a sink pattern (*bottom*). *Warmer colors* denote higher amplitudes

Advances in mapping the human connectome have yielded increasingly-detailed descriptions of large-scale brain networks, prompting growing interest in the dynamics that emerge from this structural connectivity. Moreover, there is a desire to move beyond simple static functional connectivity measures to better describe and understand the more complex repertoire of brain dynamics, which unfolds on multiple time scales. Here, we analyze the dynamics that emerge from a neural mass model [1, 2] with network connectivity derived from densely-seeded probabilistic tractography on human diffusion imaging data [3]. We find a rich array of three-dimensional wave patterns, including traveling waves, spiral waves, sources, and sinks (Fig. 101). These patterns are metastable, with the dynamics cycling between several relatively long-lived states. Varying the overall coupling strength and coupling delay reveals a complex parameter space, with other emergent patterns such as cycling between strongly-correlated clusters and multistability between different regimes (as distinct from metastability within a single regime). These dynamics accord with empirical data from multiple imaging modalities, including observations of electrical waves in cortical tissue [4] and the presence of sequential spatiotemporal patterns in resting state MEG data [5]. By characterizing the dynamic states and time scales in our simulated data, we demonstrate the richness of dynamics that emerge from the human connectome. This work lays a platform for detailed analyses of large-scale functional neuroimaging data and their mechanistic underpinnings.

**References**

1. Breakspear M, Terry JR, Friston KJ: Modulation of excitatory synaptic coupling facilitates synchronization and complex dynamics in a biophysical model of neuronal dynamics. Network. 2003;14:703–32.
2. Gollo LL, Zalesky A, Hutchison RM, van den Heuvel M, Breakspear M. Dwelling quietly in the rich club: Brain network determinants of slow cortical fluctuations. Philos Trans R Soc Lond B Biol Sci. 2015;370:20140165.
3. Roberts JA, Perry A, Lord AR, Roberts G, Mitchell PB, Smith RE, Calamante F, Breakspear M. The contribution of geometry to the human connectome. NeuroImage. 2016;124:379–93.
4. Townsend RG, Solomon SS, Chen SC, Pietersen AN, Martin PR, Solomon SG, Gong P. Emergence of complex wave patterns in primate cerebral cortex. J Neurosci. 2015;35:4657–62.
5. Baker AP, Brookes MJ, Rezek IA, Smith SM, Behrens T, Smith PJ, Woolrich M. Fast transient networks in spontaneous human brain activity. eLife. 2014;3:e01867.

## P195 Comparison of three methods to quantify detection and discrimination capacity estimated from neural population recordings

### Gary Marsat^1^, Jordan Drew^1^, Phillip D. Chapman^1^, Kevin C. Daly^1^, Samual P. Bradley^1^

#### ^1^Department of Biology, West Virginia University, Morgantown, WV 26506, USA

##### **Correspondence:** Gary Marsat - gary.marsat@mail.wvu.edu

*BMC Neuroscience* 2016, **17(Suppl 1)**:P195

The responses of sensory neurons carry information about the presence and the identity of relevant external events [1]. The pattern of activity in populations of such neurons must be decoded by post synaptic networks for the information to contribute to the elaboration of behavioral responses. For two stimuli to be discriminated, or a stimulus discriminated from background (i.e. detected), the patterns of activity it elicits in the encoding neural population must be different enough that target decoders are activated differentially [2]. In this research we used recording from electrosensory neurons in Gymnotid fish [3] and recordings from the antennal lobe of moth [4] to compare three methods that can be used to quantify how accurately the neural responses can support detection and discrimination tasks. The first two methods, namely Euclidian distances [5] and spike metrics distances [3], have traditionally been used by neurophysiologist to characterize the information carried by spike trains and to compare the responses to different stimuli. The third method we explored relies on the clustering neural network provided in Matlab toolboxes that uses unsupervised learning. Neural networks of this type are widely used by engineers to perform practical tasks but are rarely used by neuroscientist to study actual neural systems. The clustering tool relies on a quantification similar in many ways to Euclidian distances or spike distance metrics. However, since it learns to weight the inputs to allow optimal clustering, the weight patterns of networks that cluster accurately can reveal features that actual neural networks ought to have (including synaptic facilitation and depression or amount of convergence of inputs). We show that, both for the olfactory system of moth and the electrosensory system of fish, the neural network decoder outperforms the other two decoding analyses by weighting more heavily information rich inputs and more weakly noisy ones. We argue that this tool can be advantageously used to quantify neural coding, can make testable prediction regarding the characteristics of the decoding network, and, most importantly, can be easily used and implemented by researchers who have little training in neural modeling.

**Acknowledgements:** This work was supported by NSF Grant IOS-1557846 to G.M.

**References**

1. Ollerenshaw DR, Zheng HJV, Millard DC, Wang Q, Stanley GB. The adaptive trade-off between detection and discrimination in cortical representations and behavior. Neuron. 2014;81(5):1152–64.
2. Clemens J, Ronacher B. Feature extraction and integration underlying perceptual decision making during courtship behavior. J Neurosci. 2013;33(29):12136–45.
3. Marsat G, Maler L. Neural heterogeneity and efficient population codes for communication signals. J Neurophysiol. 2010;104(5):2543–55.
4. Staudacher EM, Huetteroth W, Schachtner J, Daly KC. A 4-dimensional representation of antennal lobe output based on an ensemble of characterized projection neurons. J Neurosci Methods. 2009;180(2):208–23.
5. Daly KC, Bradley S, Chapman PD, Staudacher EM, Tiede R, Schachtner J. Space takes time: concentration dependent output codes from primary olfactory networks rapidly provide additional information at defined discrimination thresholds. Front Cell Neurosci. 2016;9.

## P196 Quantifying the constraints for independent evoked and spontaneous NMDA receptor mediated synaptic transmission at individual synapses

### Sat Byul Seo^1^, Jianzhong Su^1^, Ege T. Kavalali^2^, Justin Blackwell^1^

#### ^1^Department of Mathematics, University of Texas at Arlington, Arlington, TX 76019, USA; ^2^Department of Neuroscience, University of Texas Southwestern Medical Center, Dallas, TX 75390, USA

##### **Correspondence:** Sat Byul Seo - satbyul.seo@mavs.uta.edu

*BMC Neuroscience* 2016, **17(Suppl 1)**:P196

Presynaptic terminals release neurotransmitters either in response to action potentials or spontaneously independent of presynaptic activity. In the case of glutamate, released neurotransmitters activate N-methyl-D-asparate (NMDA) receptors within a single postsynaptic site and give rise to miniature postsynaptic currents. In this study, we used a mathematical model to simulate spontaneous and evoked neurotransmission processes resulting from glutamate release within a synapse and evaluate the quantitative constraints that determine their degree of overlap independent signaling mediated by spontaneous and evoked release events. First we simulated isotropic diffusion of 4000 glutamates molecules release from a point source. We then simulated release of the glutamate molecules through a vesicle by addition of two compartments that one modeled the vesicle and the other represented the fusion pore. After we obtains the glutamate concentration from the standard heat equation then determine the opening probability of individual receptor using a state model (3C2O). Those two problems in MATLAB are solved.

**Fig. 102:**
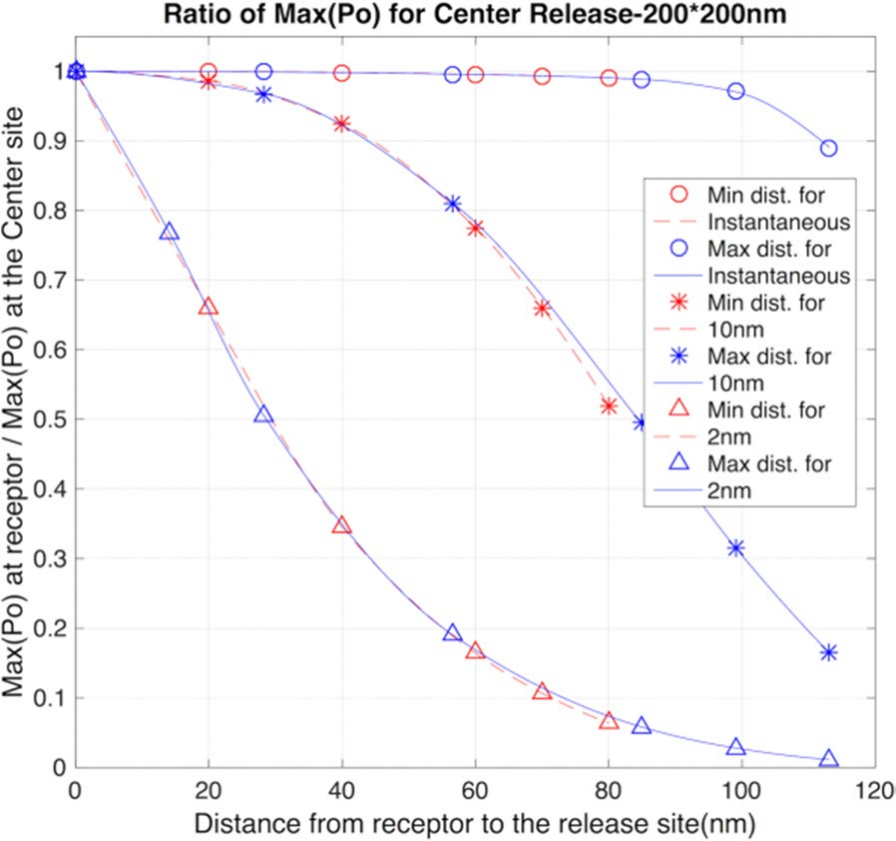
In small synapses (200 nm × 200 nm), Ratios of maximum NMDA receptor opening probabilities as functions of receptor distance for different release speed (slow, 2 nm fusion pore—*triangle*, regular, 10 nm fusion pore—*asterisk*, and instantaneous—*circle*) of glutamate vesicle release. The open probabilities were calculated by the kinetics equation, when glutamates are released above the center location

If we assume a fivefold–tenfold ratio as a good indicator for independent currents, then we cannot assure independency with the structure for medium and small synapses in our current hypothesis. Figure 102 shows that small synapse (200 nm × 200 nm) might not have independent signaling when glutamate release instantaneously because evoked and spontaneous receptors are not far away from each other and thus not far from the release site in either evoked or spontaneous releases, the ratio of open probability is close to 1. The open probability is consistent up to 90 nm far from the release site as in Fig. 102. However for small synapses, as glutamate release through 10 and 2 nm vesicle fusion pore, the open probability ratio decreases more drastically and become close to zero, and in 2 nm pore, the ratio achieves 10-fold reduction at 90 nm distance, giving plausibility for independent signaling.

**Conclusion** From the results we conclude that peak open value is most sensitive to the distance from the receptor to the release site. Glutamate release speed or fusion pore size is relevant but to a lesser degree. The calculation was first performed for a large synapse of 0.36 µm^2^ (with R6 near the center for evoked neurotransmission, and R16 for spontaneous neurotransmission) which established in theory that two non-overlapping domains that give rise to independent signaling in large synapses [1]. Then calculations in medium size 0.16 µm^2^ and small size 0.04 µm^2^ push the biophysical envelope for independent currents, as the degree of independence decreases when the size of synapse gets smaller, or the distances from evoked and spontaneous receptors to the release site are closer together.

**Reference**

1. Atasoy D, Ertunc M, Moulder KL, et al. Spontaneous and evoked glutamate release activates two populations of NMDA receptors with limited overlap. J Neurosci. 2008;28:10151–166.

## P199 Gamma oscillation via adaptive exponential integrate-and-fire neurons

### LieJune Shiau^1^, Laure Buhry^2^, Kanishka Basnayake^3^

#### ^1^Department of Mathematics, University of Houston, Clear Lake, Houston, TX, 77059, USA; ^2^Department of Computational Neurosciences, University of Lorraine, Nancy, 54600, France; ^3^Computational Neurosciences Laboratory, Ecole Polytechnique Federale de Lausanne, CH-1015, Switzerland

##### **Correspondence:** LieJune Shiau - shiau@uhcl.edu

*BMC Neuroscience* 2016, **17(Suppl 1)**:P199

Coherent oscillation of neuronal spiking in the brain is known related to cognitive functions, including perception, attention, and memory. It is therefore important to determine the properties of neurons and network architectures in emerging the coherent activities that influence the network collective behaviors. It is known that, in local cortical circuits, the probability for any pair of pyramidal cells to be connected is low and about 0.1–0.2 [1]. Wang and Buzsaki [2] numerically demonstrate that, in a heterogeneous and inhibitory network with sparse and random connection, the *minimal* connection required per neuron to observe coherence oscillation is approximately 60 with Hodgkin–Huxley (H–H) type interneurons in certain parameter regime. More importantly, this minimal number is relatively independent to the network size. In contrast, Golomb and Hanel [3] theoretically show that, identical inhibitory neurons in a sparse and random network, the minimal connection required per neuron to exhibit coherence oscillation is about 360 with integrate-and-fire (IF) neurons. The minimal connection required in either study depends on the intrinsic and synaptic properties of the neurons.

It is shown that hippocampal CA1 neurons make an average of about 60 contacts to other neurons within a spatial span of approximately 500 μm [4]. Hence, to study rhythmic oscillation in hippocampal networks, H–H type neurons, instead of IF neurons, could produce Gamma rhythm through sparsely connected network in a population of interneurons. These findings are believed to address the importance of detailed physiological properties of single neurons in determining collective network behaviors.

We adopt an increasingly popular two-dimensional *adaptive exponential integrate*-*and*-*fire* (aEIF) model [5] which is equipped with a *subthreshold adaptation* coupling the voltage and a slow current, and a *spike*-*triggered adaptation* regulated through each spike. To demonstrate that aEIF neurons can provide adequate networks in inducing Gamma frequency as in hippocampus effectively, we establish the minimal synaptic contacts required in sparse and random networks of aEIF neurons to exhibit coherent oscillations, and the impacts of neuronal and synaptic properties have on the minimal value. The aEIF neuron provides more physiological neuronal details than IF neuron, but much less than the H–H neurons. Intuitively, it may be anticipated that the minimal synaptic contacts of aEIF required in such networks lies somewhere between that of the IF and H–H neurons.

We demonstrate that the minimal synaptic contacts required in such networks of aEIF neurons to exhibit Gamma rhythm is also surprisingly low with an approximation of 60 in certain parameter regime. More specifically, the minimal connection required per neuron for the onset of network synchrony is not a faction of the total number of network neurons. It either remains constant or only depends weakly on the total network neurons. This study indicates that the inclusion of subthreshold and spike-triggered adaptations provides aEIF neuron with features to compensate for the lack of physiological details, as supposed to its H–H neuron counterpart, in studying Gamma rhythm in the brain. Our result is very encouraging in building neural network studies through a simple two-dimensional model.

**References**

1. Song S, Sjostrom P, Reigl M, Nelson S, Chklovskii D. Highly nonrandom features of synaptic connectivity in local cortical circuits. PLoS Biol. 2005;3(3):0507–19.
2. Wang X, Buzsaki G. Gamma oscillation by synaptic inhibition in a hippocampal interneuronal network model. J Neurophysiol. 1996;20:6402–13.
3. Golomb D, Hansel D. The number of synaptic inputs and the synchrony of large sparse neuronal networks. Neural Comput. 1999;12(5):1095–1139.
4. Sik A, Penttonen M, Ylinen A, Buzsaki G. Hippocampal CA1 interneurons: an in vivo intracellular labeling study. J Neurosci. 1999;24:49–65.
5. Brette R, Wulfram G. Adaptive exponential integrate-and-fire model as an effective description of neuronal activity. J Neurophysiol. 2005;94:3637–42.

## P200 Visual face representations during memory retrieval compared to perception

### Sue-Hyun Lee^1,2^, Brandon A. Levy^3^, Chris I. Baker^3^

#### ^1^Department of Bio and Brain Engineering, Korea Advanced Institute of Science and Technology (KAIST), Daejeon 34141, Republic of Korea; ^2^ Program of Brain and Cognitive Engineering, Korea Advanced Institute of Science and Technology (KAIST), Daejeon 34141, Republic of Korea; ^3^Laboratory of Brain and Cognition, National Institute of Mental Health, National Institutes of Health, Bethesda, MD 20892, USA

##### **Correspondence:** Sue-Hyun Lee - suelee@kaist.ac.kr

*BMC Neuroscience* 2016, **17(Suppl 1)**:P200

In our daily life, we can easily discriminate and recognize familiar faces. Much evidence suggests that the fusiform face area (FFA) and the occipital face area (OFA) are involved in face processing [1–3]. However, it remains unclear how individual face information is represented in the visual cortex during retrieval compared to perception. To address this question, we performed an event-related functional magnetic resonance imaging (fMRI) experiment, comprising separate perception, learning and retrieval sessions. During the perception session, which took place inside the scanner, participants were presented with fixed pairings of six auditory cues (pseudowords) with six face images, and six auditory cues with six shoe images. During the learning session, which took place on a separate day outside the scanner, participants were trained to memorize the pseudoword-image associations for about 1 h. Finally, 1 day after the learning session, participants were scanned again and instructed to retrieve each image in response to auditory presentation of the paired pseudoword cue. To test the veracity of the retrieved visual information, participants were asked to perform forced-choice tests after the retrieval scan session, in which they heard one of the pseudoword cues and chose the paired category or image. Every participant showed near perfect performance in the forced-choice test. We focused on the patterns of response in face-selective cortical areas. Using multivoxel pattern analyses, we found that FFA showed more discriminable patterns of response to individual faces during retrieval compared to those elicited during perception. In contrast object-selective areas, which respond well to images of shoes, did not show any significant difference between perception and retrieval for individual shoe images. To determine whether the increased discrimination reflected a difference between perceived and retrieved face information and not an effect of learning, we conducted a similar fMRI experiment in which the second session was also perception and not retrieval. Importantly, there was no difference in face discrimination between the first and second perception sessions in FFA. Taken together, these results suggest that retrieval of face information generates more discriminative neural responses for individual faces than that evoked by perception of the very same faces.

**Acknowledgements:** This work was supported by the US National Institutes of Health Intramural Research Program of the National Institute of Mental Health, and a NARSAD Young Investigator Grant from the Brain & Behavior Research Foundation.

**References**

1. Kanwisher N, McDermott J, Chun MM. The fusiform face area: a module in human extrastriate cortex specialized for face perception. J Neurosci. 1997;17:4302–11.
2. Tarr MJ, Gauthier I. FFA: a flexible fusiform area for subordinate-level visual processing automatized by expertise. Nat Neurosci. 2000;3:764–9.
3. Kanwisher N, Yovel G. The fusiform face area: a cortical region specialized for the perception of faces. Philos Trans R Soc Lond B Biol Sci. 2006;361:2109–28.

## P201 Top-down modulation of sequential activity within packets modeled using avalanche dynamics

### Timothée Leleu^1^, Kazuyuki Aihara^1^

#### ^1^Institute of Industrial Science, the University of Tokyo, Tokyo, Japan

##### **Correspondence:** Timothée Leleu - timothee@sat.t.u-tokyo.ac.jp

*BMC Neuroscience* 2016, **17(Suppl 1)**:P201

Recent experiments show that short activity packets are triggered by external stimuli or internal spontaneous events during which the temporal order of spikes is only partially stereotypical [1]. Moreover, it has been suggested that the timing of neurons during these packets depends on top-down modulatory inputs that “gate” the sensory information and represents either the replay of previously stored patterns or information about ongoing external stimuli [1]. Finally, it has been observed that spontaneous activity consists in the superposition of multiple overlapping packets [1]. We propose a simple model of cortical neural networks that reproduces these experimental observations [1] and an analytical description of the top-down modulation of packets using avalanche dynamics [2]. The proposed theory allows predicting the average size of packets using the synaptic weight matrix and vice versa.

The model describes the neural activity within a cortical area that receives top-down modulatory and bottom-up sensory inputs from higher-order areas and thalamic projections, respectively. The activity of excitatory neurons is simulated using the leaky integrate-and-fire model. Excitatory synaptic connections are modified on shorter and longer time-scales by short-term depression and spike-timing dependent plasticity, respectively. Sequential patterns are stored within the recurrent connections of the middle area by repeated presentation of the external stimuli.

**Fig. 103:**
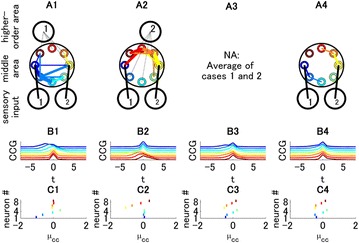
**A1–A4** Network structure. Neurons of the first and second stored pattern are represented by colors ranging from *blue* to *green* and *yellow* to *red*, respectively. Effective synaptic connections can be calculated and are shown by colored segments. **B1**–**B4** Cross-correlograms (CCG) of single neuron activity with the summed activity of other neurons (see [1]). **C1**–**C4** Center of mass of CCGs, noted μ_CC_

Figure 103A1–C1, A2–C2 show that only the first and second stored sequences are replayed when the first and second top-down input is active, respectively, although the bottom-up inputs trigger the starting neurons of both sequential patterns. When there is no top-down input, the spontaneous activity is composed of the time-compressed superposition of both sequential patterns (see Fig. 103A4–C4).

**Acknowledgements:** This research was supported by ImPACT Program of Council for Science, Technology and Innovation (Cabinet Office, Government of Japan).

**References**

1. Luczak A, Bartho P, Harris KD. Gating of sensory input by spontaneous cortical activity. J Neurosci. 2013;33(4):1684–95.
2. Leleu T, Aihara K. Unambiguous reconstruction of network structure using avalanche dynamics. Phys Rev E. 2015;91:022804.

## Q28 An auto-encoder network realizes sparse features under the influence of desynchronized vascular dynamics

### Ryan T. Philips^1^, Karishma Chhabria^1^, V.Srinivasa Chakravarthy^1^

#### ^1^Department of Biotechnology, Indian Institute of Technology, Madras, Chennai 600036, India

##### **Correspondence:** V. Srinivasa Chakravarthy - schakra@iitm.ac.in

*BMC Neuroscience* 2016, **17(Suppl 1)**:Q28

Please note that this abstract was presented at the previous year’s 24th Annual Computational Neuroscience Meeting: CNS-2015.

Cerebral vascular dynamics are generally thought to be controlled by neural activity in a unidirectional fashion. However, both computational modeling and experimental evidence points to the feedback effects of vascular activity on neural dynamics [1, 2]. Vascular feedback in the form of glucose and oxygen controls neuronal ATP, which in turn can control the threshold of neural firing. We present a computational model of a neuro-vascular system in which a network of ‘vascular units’ supply ‘energy’ to a neural network (NN), which reduces neural firing threshold. The vascular network (VN) is modeled by a network of oscillators as in [3]. Neuronal pools fed by the complex dynamics of VN are turned ON and OFF randomly. We show that such a feedback mechanism results in sparse weight matrix, thereby enhancing the performance of an auto-encoder NN.

**Fig. 104:**
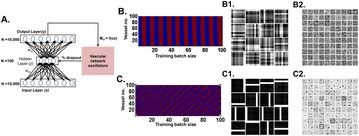
**A** Auto-encoder NN coupled to the VN. **B**, **C** Depict desynchronized (*ɛ* = 1) and synchronized (*ɛ* = 0) states of VN respectively. The corresponding output weight patterns learnt by the auto-encoder, driven by the VN, trained on bar pattern data (**B1**, **C1**) and MNIST data (**B2**, **C2**)

In the proposed NN model, the hidden layer is coupled to a vascular network in a one-to-one fashion (Fig. 104A) and is trained using back-propagation. The cross-entropy (*ce*) error measure is used to update the energy demand parameter (*M*_*d*_) which is fed back to the VN. *M*_*d*_ in turn governs the state of the vascular units, which determines if the neuron should be turned ON/OFF. This paradigm of randomly turning neural units ON/OFF is adapted from [4]. High *M*_*d*_ results in an increase in *ce* as the neuronal dropout level is too low; similarly low *M*_*d*_ results in an increase in *ce* due to high dropout. The time scale of vascular dynamics (*M*_*d*_) is much longer than that of neural dynamics (*ce*), reflecting physiology. The network was trained on two datasets: overlapping bar patterns and MNIST data. The settled weight matrices corresponding to both the synchronized (Fig. 104B) and desynchronized vascular dynamics (Fig. 104C) are shown in the Fig. 104B1, B2, C1, C2, respectively. Our earlier modeling study highlighted the link between desynchronized vascular dynamics and efficient energy delivery in skeletal muscle [3]. We now show that desynchronized vascular dynamics leads to efficient training in an auto-encoder NN.

**References**

1. Chander BS, Chakravarthy VS. A computational model of neuro-glio-vascular loop interactions. PloS One. 2012;7(11):e48802.
2. Moore CI, Cao R: The hemo-neural hypothesis: on the role of blood flow in information processing. J Neurophysiol. 2008;99(5):2035.
3. Pradhan RK, Chakravarthy V, Prabhakar A. Effect of chaotic vasomotion in skeletal muscle on tissue oxygenation. Microvasc Res. 2007;74(1):51–64.
4. Srivastava N, Hinton G, Krizhevsky A, Sutskever I, Salakhutdinov R. Dropout: a simple way to prevent neural networks from overfitting. J Mach Learn Res. 2014;15(1):1929–58.

